# Supplementary material for: Enantioselective construction of ortho-sulfur- or nitrogen-substituted axially chiral biaryls and asymmetric synthesis of isoplagiochin D
Source: Nat Commun. 2022 Aug 5;13:4577. doi: 10.1038/s41467-022-32360-7 (PMC9355965; doi:10.1038/s41467-022-32360-7)
Supplement: Supplementary file 1 — Supplementary Information [file 41467_2022_32360_MOESM1_ESM.pdf]

Supplementary Information

Enantioselective Construction of *ortho*-Sulfur- or Nitrogen-Substituted Axially Chiral Biaryls and Asymmetric Synthesis of Isoplagiochin D

He Yang[†] and Wenjun Tang[*,†,‡]

[†]State Key Laboratory of Bio-Organic and Natural Products Chemistry, Shanghai Institute of Organic Chemistry, Chinese Academy of Sciences

[‡]Hangzhou Institute for Advanced Study, University of Chinese Academy of Sciences

*E-mail:tangwenjun@sioc.ac.cn

Table of contents

1. Supplementary Methods……………………..……………………………………S1

1.1 General Information ……………………………..…………………….………S1

1.2 Preparation of MeO-PEG-BaryPhos…………..…………….………..………....S2

1.3 Preparation of Substrates………………………………………….………..……....S8

1.4 Asymmetric Cross-Coupling for Chiral Biaryl Synthesis ………………….…..…..S16

1.5 Recycling experiment with MeO-PEG-BaryPhos ………………………….........S53

1.6 Comparison of Represent Chiral Ligands in Enantioselective Cross-Coupling....S55

1.7 Proposed stereochemical model and structure feature of substrates………...…..S56

1.8 Determination of racemization barrier of related products……………….……...S57

1.9 Transformation of Axially Chiral Biaryl Products…………………..………......S59

1.10 Enantioselective Synthesis of Isoplagiochin D……….…………...………...S71

1.11 Determination of the Absolution Configuration of **6a** and *P*-**17**….…………..…S78

1.12 Photophysical Property Studies…….……………………………………...….S81

2. Supplementary Figures………………………………………...…………S83

2.1 NMR Spectra……………………………………….……………………….…..S83

3. Supplementary References …………..……………………………………..S189

1. Supplementary Methods

1.1 General Information

Most of reactions were carried out under nitrogen atmosphere using Schlenk techniques. Reagents were purchased at the highest commercial quality and used without further purification, unless otherwise stated. Tris(dibenzylideneacetone)dipalladium was purchased from J&K Chemical Co. Ltd.. Anhydrous THF and toluene were distilled from sodium/benzophenone immediately prior to use. All other solvents and reagents were used as received from commercial sources (Bidepharmatech and Titan sci.), unless otherwise specified. Analytical thin layer chromatography (TLC) was performed on precoated silica gel 60 GF254 plates. Flash column chromatography was performed using Tsingdao silica gel (200-300 mesh). Visualization on TLC was achieved by use of UV light (254 nm), iodine or basic $KMnO_4$ dip.

^{1}H NMR, ^{31}P NMR, ^{19}F NMR and ^{13}C NMR data were recorded on a Bruker-Ultrashield PLUS 400, a 500 MHz Agilent or a Bruker Avance 600 AV spectrometer with $CDCl_3$ or DMSO-d_6 as the solvent. ^{1}H chemical shifts were referenced to $CDCl_3$ at 7.26 ppm or DMSO-d_6 at 2.50 ppm. ^{13}C chemical shifts were referenced to $CDCl_3$ at 77.16 ppm or DMSO-d_6 at 39.52 ppm, and obtained with ^{1}H decoupling. Multiplicities are abbreviated as follows: singlet (s), doublet (d), triplet (t), quartet (q), doublet-doublet (dd), multiplet (m), and broad (br). MS was measured on Agilent 7890A/5975C Series GC/MSD mass spectrometer or Agilent 1100 Series LC/MSD mass spectrometer. The molecular weights of PEG supported ligands were determined with a Waters 1515 GPC. Chiral HPLC analyses were performed on an Agilent 1100 Series using a Daicel Chiralpak column with hexane/*i*PrOH or MeCN/H_2O as the eluent. Optical rotations were measured on an Anton Paar Modular Circular Polarimeter. Absorption spectra were measured with a LAMBDA 950 UV-Vis spectrophotometer. Emission spectra were measured with a Perkinelmer LS55 spectrometer. Circular dichroism (CD) spectra were measured on an APPLIED PHOTOPHYSICS Chirascan CD spectrometer. Circular polarized luminescence (CPL) were measured on a JASCO CPL-300 spectrometer.

Abbreviations are as follows: Ac, acetyl; Ar, aryl; Bn, benzyl; dba, dibenzylideneacetone; DCM, dichloromethane; DMF, *N*,*N*-dimethylformamide; EA, ethyl acetate; LDA, lithium diisopropylamide; PE, petroleum ether; PEG, polyethylene glycol.

1.2 Preparation of MeO-PEG-BaryPhos

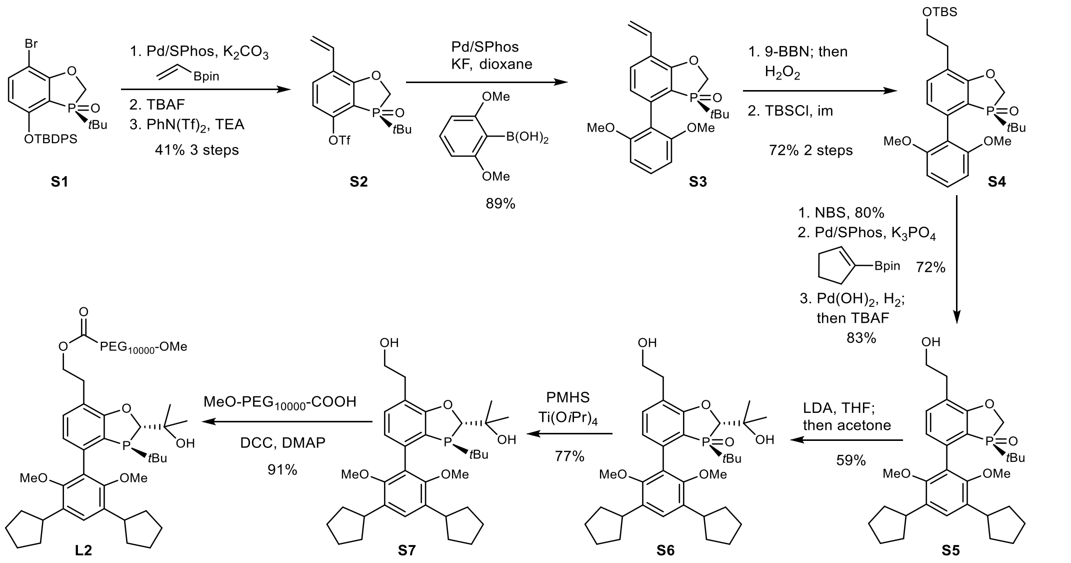

Supplementary Figure 1 Preparation of MeO-PEG-BaryPhos. Synthetic route of supported BaryPhos.

(*R*)-3-(tert-butyl)-3-oxido-7-vinyl-2H-benzo[d][1,3]oxaphosphol-4-yl trifluoromethanesulfonate (S2)

(*R*)-7-bromo-3-(tert-butyl)-4-((tert-butyldiphenylsilyl)oxy)-2H-benzo[d][1,3]oxaphosphole 3-oxide (**S1**) was prepared according to reported procedure.[1]

To a Schlenk tube was charged with **S1** (3.90 g, 7.18 mmol), $Pd_2(dba)_3$ (65.8 mg, 0.072 mmol), SPhos (59.0 mg, 0.144 mmol) and K_3PO_4 (3.81 g, 18.0 mmol). The Schlenk tube was evacuated and back-filled with nitrogen for 3 times before the addition of 2-vinyl-4,4,5,5-tetramethyl-1,3,2-dioxaborolane (2.21 g, 14.4 mmol), degassed toluene (30 mL) and H_2O (6 mL). The Schlenk tube was sealed and the mixture was stirred at 80 °C for 15 h. Upon completion of the reaction, the mixture was diluted with EA (30 mL) and filtered through a layer of celite. The Pd catalyst was wash twice with EA (10 mL). The filtrate was washed with brine (10 mL×2), separated, dried over Na_2SO_4, filtered and concentrated under reduced pressure to afford crude vinylated product, which was dissolved in THF (20 mL) and treated with TBAF (8.62 mL, 8.62 mmol, 1.0 M). The reaction solution was stirred at rt for 3 h before being quenched with saturated NH_4Cl (15 mL). The resulting mixture was extracted with EA (15 mL ×3). The combined organic phases were washed with brine (20 mL), dried over Na_2SO_4, filtered and concentrated under reduced pressure to afford a pale solid.

The above crude intermediate was dissolved in DCM (25 mL). To the solution was added $PhN(Tf)_2$ (3.08 g, 8.62 mmol) and trimethylamine (1.50 mL, 10.77 mmol). The mixture was stirred at rt for 12 h. Upon completion, the mixture was partitioned between DCM (20 mL) and H_2O (20 mL). The organic phase was separated and the aqueous layer was extracted with DCM (15 mL ×3). The organic phases were combined, dried over Na_2SO_4, filtered and concentrated under reduced pressure. The crude product was purified by column chromatography (eluent: EA/PE = 1/2) to give **S2** (1.13 g, 41% yield over 3 steps) as a white foam. $[\alpha]^{25}_D$ = -27.7 (c = 1, $CHCl_3$); ^1H NMR (400 MHz,

$CDCl_3$) δ 7.65 (d, *J* = 8.5 Hz, 1H), 7.05 (dd, *J* = 8.5, 3.8 Hz, 1H), 6.78 (dd, *J* = 17.8, 11.3 Hz, 1H), 5.89 (d, *J* = 17 .7 Hz, 1H), 5.44 (d, *J* = 11.7 Hz, 1H), 4.72 (dd, *J* = 14.1, 2.3 Hz, 1H), 4.54 (dd, *J* = 14.1, 11.0 Hz, 1H), 1.27 (d, *J* = 16.8 Hz, 9H); ^{13}C NMR (101 MHz, $CDCl_3$) δ 163.0 (d, *J* = 16.4 Hz), 148.4, 133.2 (d, *J* = 1.1 Hz), 128.5, 124.3 (d, *J* = 5.1 Hz), 118.4 (q, *J* = 322.2 Hz), 118.2, 113.6, 107.9 (d, *J* = 85.7 Hz), 66.2 (d, *J* = 59.4 Hz), 34.1 (d, *J* = 72.4 Hz), 23.9; ^{19}F NMR (376 MHz, $CDCl_3$) δ -73.3; ^{31}P NMR (162 MHz, $CDCl_3$) δ 61.9; HRMS (ESI) Calcd. for $C_{14}H_{17}F_3O_5PS$ $[M+H]^+$: 385.0486; Found: 385.0487.

(*R*)-3-(tert-butyl)-4-(2,6-dimethoxyphenyl)-7-vinyl-2H-benzo[d][1,3]oxaphosphole 3-oxide (S3)

In the glovebox, to a flame-dried Schlenk tube was added **S1** (2.20 g, 5.73 mmol), $Pd_2(dba)_3$ (53.1 mg, 0.058 mmol), SPhos (47.0 mg, 0.115 mmol), KF (1.00 g, 17.2 mmol), 2,6-dimethoxyphenylboronic acid (2.09 g, 11.5 mmol) and 1,4-dioxane (10 mL). The Schlenk tube was sealed and taken out of the glovebox. The mixture was stirred at 100 °C for 12 h. Upon completion of the reaction, the mixture was diluted with EA (30 mL) and filtered through a layer of Celite. The Pd catalyst was wash twice with EA (10 mL). The filtrate was washed with brine (10 mL×2), separated, dried over Na_2SO_4, filtered and concentrated under reduced pressure. The crude product was purified by column chromatography (eluent: EA/PE = 1/1) to give **S3** (1.90 g, 89% yield) as a white foam. $[\alpha]^{25}_D$ = -20.3 (*c* = 1, $CHCl_3$); ^1H NMR (500 MHz, $CDCl_3$) δ 7.61 (d, *J* = 7.7 Hz, 1H), 7.29 (t, *J* = 8.4 Hz, 1H), 6.89 (td, *J* = 12.2, 8.8 Hz, 2H), 6.65 (d, *J* = 8.4 Hz, 1H), 6.56 (d, *J* = 8.3 Hz, 1H), 5.84 (dd, *J* = 17.8, 1.2 Hz, 1H), 5.34 (dd, *J* = 11.2, 1.2 Hz, 1H), 4.52 (dd, *J* = 13.8, 1.8 Hz, 1H), 4.39 (dd, *J* = 13.8, 10.5 Hz, 1H), 3.77 (s, 3H), 3.71 (s, 3H), 0.87 (d, *J* = 15.9 Hz, 9H); ^{13}C NMR (126 MHz, $CDCl_3$) δ 162.5 (d, *J* = 19.1 Hz), 158.7, 157.5, 137.6 (d, *J* = 5.7 Hz), 131.1 (d, *J* = 2.0 Hz), 130.4, 130.1, 125.2 (d, *J* = 9.2 Hz), 122.8 (d, *J* = 6.2 Hz), 117.3, 115.9, 114.9 (d, *J* = 91.2 Hz), 104.6, 103.1, 65.5 (d, *J* = 60.9 Hz), 56.1, 55.5, 33.7 (d, *J* = 71.9 Hz), 23.8 (d, *J* = 0.6 Hz); ^{31}P NMR (162 MHz, $CDCl_3$) δ 62.1; HRMS (ESI) Calcd. for $C_{21}H_{26}O_4P$ $[M+H]^+$: 373.1563; Found: 373.1560.

(*R*)-3-(tert-butyl)-7-(2-((tert-butyldimethylsilyl)oxy)ethyl)-4-(2,6-dimethoxyphenyl)-2H-benzo[d][1,3]oxaphosphole 3-oxide (S4)

To a solution of **S3** (1.76 g, 4.73 mmol) in anhydrous THF (25 mL) at 0 °C was added 9-BBN dimer (0.68 g, 2.84 mmol) and then the mixture was allowed to warm to rt and stirred for 10 h. Upon fully consumption of **S3**, saturated NH_4Cl (15 mL) was added to quench the reaction and the mixture was extracted with EA (15 mL ×3). The organic phases were combined, washed once with brine (15 mL), dried over Na_2SO_4, filtered and concentrated under reduced pressure. The obtained residue was dissolved in MeOH (10 mL) and H_2O (10 mL). The solution was cooled to 0 °C before the addition of NaOH (1.35 g, 33.84 mmol). 30% H_2O_2 (3.42 mL, 27.07 mmol) was added dropwise to the solution and the resulting mixture was allowed to warm to rt and stirred for 6 h. Saturated $Na_2S_2O_3$ (aq) (15 mL) was added and the mixture was extracted with EA (10 mL ×3). The organic phases were combined, dried over Na_2SO_4, filtered and

concentrated under reduced pressure. The crude product was dissolved in DCM (15 mL). To the solution was added imidazole (0.39 g, 5.68 mmol) and TBSCl (0.86 g, 5.68 mmol). The resulting mixture was stirred at rt for 15 h. H_2O (15 mL) was added and the mixture was extracted with DCM (10 mL ×3). The organic phases were combined, dried over Na_2SO_4, filtered and concentrated under reduced pressure. The crude product was purified by column chromatography (eluent: EA/PE = 1/2) to afford **S4** as a white foam (1.72 g, 72% yield over 2 steps). $[\alpha]^{25}_{D}$ = -15.7 (*c* = 1.0, $CHCl_3$); ^{1}H NMR (400 MHz, $CDCl_3$) δ 7.28 (d, *J* = 7.7 Hz, 1H), 7.20 (t, *J* = 8.4 Hz, 1H), 6.75 (dd, *J* = 7.5, 3.7 Hz, 1H), 6.57 (d, *J* = 8.4 Hz, 1H), 6.47 (d, *J* = 8.4 Hz, 1H), 4.41 (dd, *J* = 13.8, 1.5 Hz, 1H), 4.28 (dd, *J* = 13.7, 10.4 Hz, 1H), 3.76 (t, *J* = 6.9 Hz, 2H), 3.70 (s, 3H), 3.61 (s, 3H), 2.79 (t, *J* = 6.8 Hz, 2H), 0.81 (s, 9H), 0.79 (d, *J* = 15.9 Hz, 9H), -0.07 (d, *J* = 2.3 Hz, 6H); ^{13}C NMR (126 MHz, $CDCl_3$) δ 163.1 (d, *J* = 18.7 Hz), 158.4, 157.3, 135.7 (d, *J* = 5.8 Hz), 135.3, 129.6, 124.5 (d, *J* = 9.2 Hz), 122.9 (d, *J* = 5.7 Hz), 117.3 (d, *J* = 2.0 Hz), 113.7 (d, *J* = 91.6 Hz), 104.2, 102.8, 65.1 (d, *J* = 61.0 Hz), 62.3, 55.6, 55.1, 33.2 (d, *J* = 71.8 Hz), 33.0, 25.8, 23.6, 18.1, -5.5, -5.6; ^{31}P NMR (162 MHz, $CDCl_3$) δ 63.0; HRMS (ESI) Calcd. for $C_{27}H_{42}O_5PSi$ $[M+H]^+$: 505.2534; Found: 505.2531.

(*R*)-3-(tert-butyl)-4-(3,5-dicyclopentyl-2,6-dimethoxyphenyl)-7-(2-hydroxyethyl)-2H-benzo[d][1,3]oxaphosphole 3-oxide (S5)

To a solution of **S4** (1.30 g, 2.58 mmol) in MeCN (30 mL) was added NBS (1.15 g, 6.45 mmol) and the mixture was stirred at rt for 15 h. Upon completion of the reaction, the mixture was concentrated and partitioned between EA (20 mL) and H_2O (15 mL). The organic phase was separated and the aqueous layer was extracted with EA (15 mL ×3). The organic phases were combined, dried over Na_2SO_4, filtered and concentrated under reduced pressure. The crude brominated product **S8** was purified by column chromatography (eluent: EA/PE = 1/2) to give a colorless oil which turned to be a white foam under vacuum (1.36 g, 80% yield).

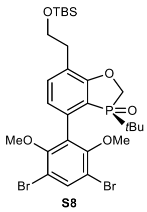

S8

(*R*)-3-(tert-butyl)-7-(2-((tert-butyldimethylsilyl)oxy)ethyl)-4-(3,5-dibromo-2,6-dimethoxyphenyl)-2H-benzo[d][1,3]oxaphosphole 3-oxide (S8)

$[\alpha]^{25}_{D}$ = -9.6 (*c* = 1.0, $CHCl_3$); ^{1}H NMR (400 MHz, $CDCl_3$) δ 7.72 (s, 1H), 7.34 (d, *J* = 7.5 Hz, 1H), 6.77 (dd, *J* = 7.5, 3.7 Hz, 1H), 4.53 (dd, *J* = 13.9, 1.7 Hz, 1H), 4.30 (dd, *J* = 13.8, 10.7 Hz, 1H), 3.78 (t, *J* = 6.2 Hz, 2H), 3.64 (s, 3H), 3.61 (s, 3H), 2.82 (dd, *J* = 12.5, 6.3 Hz, 2H), 0.96 (d, *J* = 16.2 Hz, 9H), 0.79 (s, 9H), -0.09 (s, 3H), -0.10 (s, 3H); ^{13}C NMR (101 MHz, $CDCl_3$) δ 163.5 (d, *J* = 18.4 Hz), 155.9, 154.8, 136.3, 135.3, 134.5 (d, *J* = 5.7 Hz), 131.5 (d, *J* = 2.0 Hz), 125.1 (d, *J* = 5.4 Hz), 123.5 (d, *J* = 8.6 Hz), 114.1 (d, *J* = 89.3 Hz), 112.9, 112.1, 65.7 (d, *J* = 60.8 Hz), 62.0, 61.6, 61.4, 33.8 (d, *J* = 70.9 Hz), 33.0, 25.9, 23.7, 18.2, -5.4, -5.5; ^{31}P NMR (162 MHz, $CDCl_3$) δ 63.6; HRMS (ESI) Calcd. for $C_{27}H_{40}Br_2O_5PSi$ $[M+H]^+$: 661.0744; Found: 661.0742.

A Schlenk tube was charged with **S8** (0.74 g, 1.12 mmol), $Pd_2(dba)_3$ (20.5 mg, 0.022 mmol), SPhos (18.0 mg, 0.044 mmol), K_3PO_4 (0.71 g, 3.36 mmol) and 2-(1-cyclopenten-1-yl)-4,4,5,5-tetramethyl-1,3,2-dioxaborolane (1.09 g, 5.60 mmol). The Schlenk tube was evacuated and back-filled with nitrogen for 3 times before the

addition of degassed 1,4-dioxane (10 mL) and H_2O (2 mL). The Schlenk tube was sealed and the mixture was stirred at 80 ºC for 20 h. Upon completion of the reaction, the mixture was diluted with EA (20 mL) and filtered through a layer of celite. The Pd catalyst was wash twice with EA (10 mL). The filtrate was washed with brine (10 mL), separated, dried over Na_2SO_4, filtered and concentrated under reduced pressure to afford a brown foam which was dissolved in THF (20 mL). To the mixture was added 15% $Pd(OH)_2$ (0.10 g, 15 wt%) and the system was stirred at 30 ºC under 1 atm of H_2 for 2 days. Upon completion (monitored by ^{1}H NMR), the mixture was filtered through a layer of Celite and the catalyst was wash twice with THF (15 mL). To the combined organic solvent was added TBAF (1.34 mL, 1.34 mmol, 1.0 M) and the mixture was stirred at rt for 5 h before being quenched with saturated NH_4Cl (15 mL). The resulting mixture was extracted with EA (15 mL ×3). The combined organic phases were washed with brine (20 mL), dried over Na_2SO_4, filtered and concentrated under reduced pressure. The crude product was purified by column chromatography (eluent: EA, then MeOH/EA = 1/10) to give **S5** (0.35 g, 60% yield from **S8**) as a colorless film. $[\alpha]^{25}_{D}$ = -11.1 (c = 1, $CHCl_3$); ^{1}H NMR (400 MHz, $CDCl_3$) δ 7.33 (d, J = 7.5 Hz, 1H), 7.12 (s, 1H), 6.90 (dd, J = 7.4, 3.7 Hz, 1H), 4.46 (dd, J = 13.8, 2.5 Hz, 1H), 4.33 (dd, J = 13.6, 10.6 Hz, 1H), 3.77 (dd, J = 11.1, 6.7 Hz, 2H), 3.57 (s, 3H), 3.42 (s, 3H), 3.33-3.14 (m, 2H), 3.02 (s, 1H), 2.87 (dq, J = 13.7, 6.9 Hz, 2H), 2.15-1.87 (m, 4H), 1.87-1.42 (m, 11H), 1.39-1.23 (m, 1H), 0.89 (d, J = 16.2 Hz, 9H); ^{13}C NMR (126 MHz, $CDCl_3$) δ 163.7 (d, J = 18.3 Hz), 155.1, 154.1, 137.3 (d, J = 6.1 Hz), 135.6, 134.5, 134.3, 127.6, 125.5, 124.3 (d, J = 9.0 Hz), 123.5 (d, J = 5.5 Hz), 115.3, 115.0 (d, J = 89.9 Hz), 114.6, 66.0, 65.7 (d, J = 60.6 Hz), 65.5, 62.5, 61.7, 38.7, 38.5, 35.5, 34.7, 34.3, 34.1, 33.8, 33.3, 33.1, 25.7, 25.6, 25.6 (dd, J = 17.0, 6.9 Hz), 25.6, 25.5, 24.0; ^{31}P NMR (162 MHz, $CDCl_3$) δ 63.6; HRMS (ESI) Calcd. for $C_{31}H_{44}O_5P$ $[M+H]^+$: 527.2921; Found: 527.2918.

(2*S*,3*R*)-3-(tert-butyl)-4-(3,5-dicyclopentyl-2,6-dimethoxyphenyl)-7-(2-hydroxyethyl)-2-(2-hydroxypropan-2-yl)-2H-benzo[d][1,3]oxaphosphole 3-oxide (S6)

To a solution of **S5** (0.13 g, 0.25 mmol) in anhydrous THF (6 mL) was added dropwise a solution of LDA in THF (2.5 M, 0.25 mL, 0.63 mmol) at -78 ºC. The reaction mixture was stirred for 1 h at -78 ºC before the addition of freshly distilled acetone (0.18 mL, 2.50 mmol) at the same temperature. The reaction system was warmed to rt within 2 h and stirred for 1 h at rt. The reaction was quenched by addition of H_2O (10 mL) and the resulting mixture was extracted with EA (10 mL ×3). The organic phases were combined, dried over Na_2SO_4, filtered and concentrated under reduced pressure. The crude product was purified by column chromatography (eluent: EA) to give **S6** (86 mg, 59% yield) as a white foam. $[\alpha]^{25}_{D}$ = 63.8 (c = 1, $CHCl_3$); ^{1}H NMR (400 MHz, $CDCl_3$) δ 7.38 (d, J = 7.5 Hz, 1H), 7.15 (s, 1H), 6.94 (dd, J = 7.5, 3.8 Hz, 1H), 4.25 (d, J = 3.3 Hz, 1H), 3.93 (t, J = 6.6 Hz, 2H), 3.59 (s, 3H), 3.43 (s, 3H), 3.38-3.27 (m, 1H), 3.27-3.15 (m, 1H), 3.04-2.88 (m, 2H), 2.60 (brs, 1H), 2.14-1.98 (m, 3H), 1.98-1.48 (m, 12H), 1.45 (s, 3H), 1.36 (s, 3H), 1.39-1.27 (m, 1H), 0.97 (d, J = 16.2 Hz, 9H); ^{13}C NMR (151 MHz, $CDCl_3$) δ 162.9 (d, J = 18.8 Hz), 154.9, 154.1, 138.1 (d, J = 5.6 Hz), 135.6, 134.6,

134.5, 127.1, 125.7, 125.0 (d, *J* = 9.0 Hz), 122.7 (d, *J* = 5.6 Hz), 114.4 (d, *J* = 88.7 Hz), 78.3 (d, *J* = 60.2 Hz), 73.63 (d, *J* = 1.7 Hz), 62.5, 62.3, 61.4, 38.7, 38.6, 35.6, 35.0, 34.6, 34.5, 34.3, 33.9, 33.3, 28.7 (d, *J* = 5.3 Hz), 25.9, 25.8, 25.7, 25.7, 25.0, 23.7; ^{31}P NMR (162 MHz, $CDCl_3$) δ 66.4; HRMS (ESI) Calcd. for $C_{34}H_{49}NaO_6P$ $[M+Na]^+$: 607.3159; Found: 607.3155.

2-((2*S*,3*S*)-3-(tert-butyl)-4-(3,5-dicyclopentyl-2,6-dimethoxyphenyl)-7-(2-hydroxyethyl)-2,3-dihydrobenzo[d][1,3]oxaphosphol-2-yl)propan-2-ol (S7)

At 0 ºC, to a solution of **S6** (0.10 g, 0.17 mmol) in anhydrous THF (5.0 mL) was added Ti(O*i*Pr)$_4$ (0.50 mL, 1.70 mmol), followed by PMHS (0.20 mL, 3.40 mmol). The mixture was warmed to 60 ºC and stirred for 12 h. Upon completion of the reduction (monitored by TLC), the reaction system was cooled to 0 ºC and a solution of degassed 30% NaOH (aq) (10 mL) was added. The resulting mixture was allowed to warm to rt and stirred until the two phases separated clearly (around 1.5 h). The organic layer was separated and the aqueous phase was extracted with degassed MeTHF (5 mL×3). The organic phases were combined, dried over Na_2SO_4, filtered and concentrated under reduced pressure at rt. The crude product was purified by flash column chromatography under N_2 (eluent: hexane, then EA/PE = 1:3) to afford **S7** (74 mg, 77% yield) as a white solid. $[\alpha]^{25}_D$ = -9.8 (*c* = 1.0, $CHCl_3$); ^{1}H NMR (600 MHz, $CDCl_3$) δ 7.17 (d, *J* = 7.6 Hz, 1H), 7.12 (s, 1H), 6.95 (dd, *J* = 7.5, 3.4 Hz, 1H), 4.64 (s, 1H), 3.98-.87 (m, 2H), 3.70 (s, 3H), 3.35-3.21 (m, 2H), 3.16 (s, 3H), 3.02-2.90 (m, 2H), 2.12-2.02 (m, 2H), 2.01-1.90 (m, 2H), 1.90-1.74 (m, 5H), 1.73-1.59 (m, 7H), 1.26 (s, 3H), 1.25 (s, 3H), 0.72 (d, *J* = 12.3 Hz, 9H); ^{13}C NMR (151 MHz, $CDCl_3$) δ 162.9, 154.6, 154.0, 138.2 (d, *J* = 17.7 Hz), 135.4, 134.7, 131.4, 129.1, 124.7, 124.5 (d, *J* = 15.9 Hz), 123.4 (d, *J* = 4.1 Hz), 120.0, 91.3 (d, *J* = 29.9 Hz), 73.7 (d, *J* = 18.8 Hz), 63.0, 62.8, 60.9, 39.1, 38.8, 35.7, 34.6, 34.5, 34.3, 34.2, 30.8 (d, *J* = 18.8 Hz), 27.0 (d, *J* = 14.8 Hz), 25.9, 25.8, 25.7, 25.6, 25.2 (d, *J* = 7.3 Hz); ^{31}P NMR (162 MHz, $CDCl_3$) δ -0.93; HRMS (ESI) Calcd. for $C_{34}H_{50}O_5P$ $[M+H]^+$: 569.3390; Found: 569.3388.

Preparation of MeO-PEG$_{10000}$-BaryPhos (L2)

To a flame-dried Schlenk tube was charged with **S7** (0.13 g, 0.23 mmol), MeO-PEG-COOH (Mn 10000, 1.20 g, 0.12 mmol), DCC (47 mg, 0.23 mmol) and DMAP (14 mg, 0.12 mmol). The Schlenk tube was evacuated and back-filled with nitrogen for 3 times before the addition of degassed DCM (8 mL). The Schlenk tube was sealed and the mixture was stirred at rt for 30 h. The mixture was filtered through a layer of Celite under N_2 to remove the precipitated urea and most of the filtrate was concentrated under reduced pressure until 1 mL of solvent remained. To the solution was slowly added degassed Et_2O (20 mL) and the white precipitate was isolated by filtration. The solid was washed twice with degassed Et_2O (3 mL×2) and dried *in vacuo* (1.11 g, 91% yield). ^{31}P NMR (162 MHz, $CDCl_3$) δ 0.09; Mn: 11162; Mw: 11969; MP: 12095.

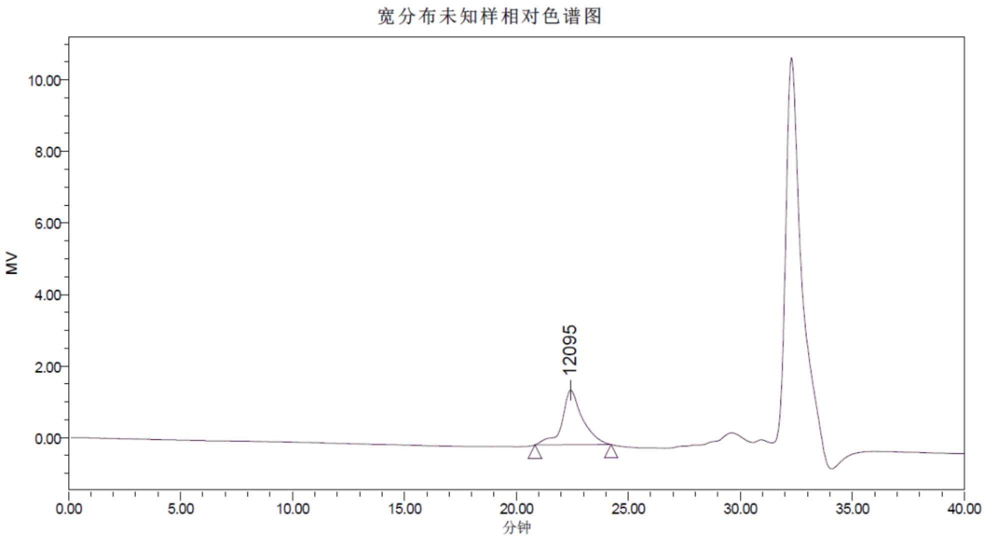

宽分布未知样相对峰表

	分布名	Mn (道尔顿)	Mw (道尔顿)	MP	Mz (道尔顿)	Mz+1 (道尔顿)	多分散性	Mz/Mw	Mz+1/Mw
1		11162	11969	12095	12885	13944	1.072335	1.076549	1.165001
2									
3									

MeO-PEG$_{2000}$-BaryPhos (**L3**) was prepared following a similar procedure. ^{31}P NMR (162 MHz, $CDCl_3$) δ 0.09; Mn: 2698; Mw: 2960; MP: 2991.

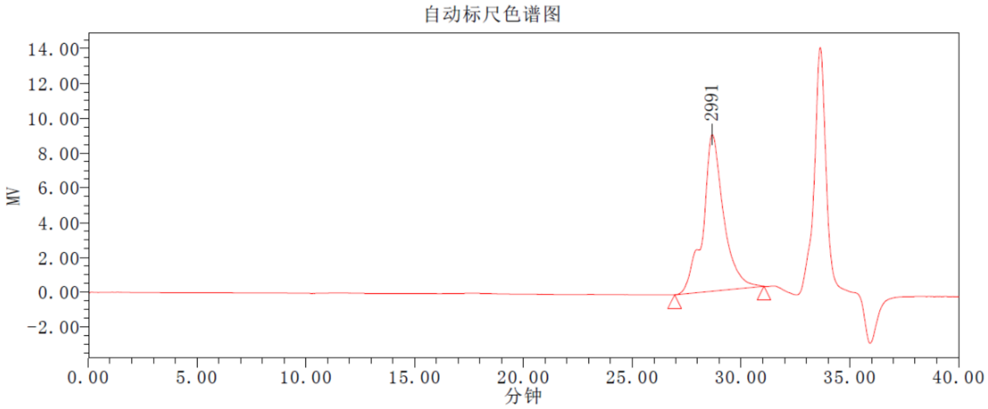

GPC 结果

	分布名	Mn (道尔顿)	Mw (道尔顿)	MP	Mz (道尔顿)	Mz+1 (道尔顿)	多分散性	MW 标记 1 (道尔顿)
1		2698	2960	2991	3201	3435	1. 096936	

GPC 结果

	MW 标记 2 (道尔顿)
1	

1.3 Preparation of Substrates

Preparation of (5-bromonaphthalen-1-yl)(morpholino)methanone (S9)

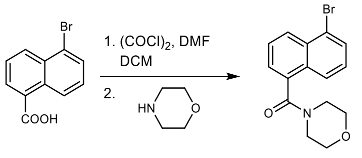

S9

To a solution of 5-bromo-1-naphthoic acid (3.0 g, 12.0 mmol) in anhydrous DCM (15 mL) at 0 °C was added oxalyl chloride (3.0 mL, 35.4 mmol) dropwise over a period of 15 min. Then one drop of DMF was added and the resulting mixture was stirred at 0 °C for 30 min. The mixture was warmed to room temperature and stirred for 4 h. The mixture was concentrated under reduced pressure and residual organic solvents were removed under high vacuum to afford the corresponding acyl chloride as a pale solid. The solid was dissolved in DCM (15 mL) and morpholine (2.6 mL, 29.9 mmol) was added at room temperature. The reaction solution was stirred at the same temperature for 8 h. Upon completion, the reaction mixture was successively washed with 2 N HCl (aq) (10 mL) and brine (10 mL). The organic phase was separated, dried over Na_2SO_4, filtered and concentrated under reduced pressure. The crude product was purified by column chromatography (eluent: EA/PE = 1/2) to afford amide **S9** as a white solid (3.5 g, 92% yield). ^{1}H NMR (400 MHz, $CDCl_3$) δ 8.29 (d, *J* = 8.6 Hz, 1H), 7.81 (t, *J* = 7.8 Hz, 2H), 7.63-7.54 (m, 1H), 7.46 (d, *J* = 6.9 Hz, 1H), 7.36 (t, *J* = 7.9 Hz, 1H), 3.99 (dt, *J* = 8.7, 4.6 Hz, 1H), 3.92-3.74 (m, 3H), 3.57-3.37 (m, 2H), 3.24-3.03 (m, 2H); ^{13}C NMR (101 MHz, $CDCl_3$) δ 168.8, 134.2, 132.0, 130.8, 130.7, 128.5, 127.5, 126.7, 124.8, 124.6, 123.4, 67.0, 66.9, 47.6, 42.2; HRMS (ESI) Calcd. for $C_{15}H_{14}BrNNaO_2$ $[M+Na]^+$: 342.0106; Found: 342.0103.

Preparation of (5-(morpholine-4-carbonyl)naphthalen-1-yl)boronic acid (S10)

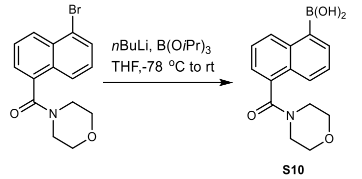

S10

To a solution of (5-bromonaphthalen-1-yl)(morpholino)methanone **S9** (3.5 g, 10.9 mmol) and B(O*i*Pr)$_3$ (6.0 mL, 26.0 mmol) in anhydrous THF (60 mL) at -78 °C was added *n*BuLi (2.5 M, 6.6 mL, 16.4 mmol) dropwise over a period of 30 min. The mixture was stirred at -78 °C for 2 h before being warmed up to room temperature over 1 h. After being further stirred at room temperature for 3 h, the mixture was cooled to 0 °C and the reaction was quenched by addition of 2 *N* HCl (aq) (20 mL). The mixture was extracted with EA (20 mL×3) and the organic phases were combined, washed with brine (20 mL), dried over Na_2SO_4, filtered and concentrated under reduced pressure. The crude product was purified by column chromatography (eluent: EA) to afford **S10** as a white solid (2.7 g, 87% yield). ^{1}H NMR (600 MHz, DMSO-*d*$_6$) δ 8.43 (d, *J* = 3.4 Hz, 2H), 8.40 (d, *J* = 8.5 Hz, 1H), 7.78 (dd, *J* = 11.9, 7.5 Hz, 2H), 7.54 (dt, *J* = 8.4, 6.9

Hz, 2H), 7.45-7.39 (m, 1H), 3.90-3.80 (m, 1H), 3.73 (t, J = 9.5 Hz, 3H), 3.47 (dd, J = 10.1, 6.4 Hz, 2H), 3.13-3.08 (m, 1H), 3.00 (dd, J = 11.2, 4.2 Hz, 1H); ^{13}C NMR (151 MHz, DMSO-d_6) δ 168.8, 135.8 134.7, 132.8, 130.1, 129.2, 126.6, 126.1, 125.4, 123.6, 66.7, 66.6, 47.6, 42.1; HRMS (ESI) Calcd. for $C_{15}H_{16}BNNaO_4$ [M+Na]$^+$: 308.1070; Found: 308.1074.

Preparation of 2-(2-(2,2-difluorovinyl)-4-(trifluoromethyl)phenyl)-4,4,5,5-tetramethyl-1,3,2-dioxaborolane (S11)

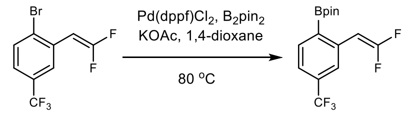

S12 S11

A flame-dried Schlenk tube was charged with 1-bromo-2-(2,2-difluorovinyl)-4-(trifluoromethyl)benzene **S12**[2] (1.66 g, 5.78 mmol), Pd(dppf)Cl_2 (0.20 g, 0.27 mmol), B_2pin_2 (2.20 g, 8.66 mmol) and KOAc (1.70 g, 17.3 mmol). The Schlenk tube was evacuated and back-filled with nitrogen for 3 times before the addition of degassed 1,4-dioxane (40 mL). The Schlenk tube was sealed and the mixture was stirred at 80 °C for 20 h. Upon completion of the reaction, the mixture was diluted with EA (30 mL) and filtered through a layer of Celite. The Pd catalyst was wash twice with EA (10 mL). The filtrate was washed with brine (10 mL×2), separated, dried over Na_2SO_4, filtered and concentrated under reduced pressure. The crude product was purified by column chromatography (eluent: EA/PE = 1/20) to afford **S11** as a colorless oil (1.68 g, 87% yield). ^{1}H NMR (500 MHz, $CDCl_3$) δ 7.94 (d, J = 7.8 Hz, 1H), 7.77 (s, 1H), 7.45 (d, J = 7.8 Hz, 1H), 6.29 (dd, J = 26.1, 4.8 Hz, 1H), 1.36 (s, 12H); ^{13}C NMR (151 MHz, $CDCl_3$) δ 156.5 (dd, J = 299.2, 288.5 Hz), 137.0 (dd, J = 7.9, 5.7 Hz), 136.8, 133.0 (q, J = 32.1 Hz), 124.2 – 123.8 (m), 123.9 (q, J = 272.6 Hz), 122.6 – 122.3 (m), 84.4, 81.4 (dd, J = 31.0, 10.5 Hz), 30.8 (d, J = 187.5 Hz); ^{19}F NMR (376 MHz, $CDCl_3$) δ -63.3, -81.7 (t, J = 27.0 Hz), -82.0 (dd, J = 28.1, 4.8 Hz); HRMS (ESI) Calcd. for $C_{15}H_{17}BF_5O_2$ [M+H]$^+$: 335.1242; Found: 335.1240.

Preparation of 3-bromo-1,2-dimethoxy-4-nitro-5-vinylbenzene (S14)

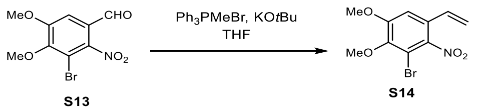

S13 S14

To a suspension of Ph_3PMeBr (0.47 g, 1.32 mmol) in anhydrous THF (20 mL) at 0 °C was added KO*t*Bu (0.15 g, 1.32 mmol). The mixture was stirred at the same temperature for 30 min before addition of a solution of aldehyde **S13**[3] (0.35 g, 1.21 mmol) in THF (5 mL). The mixture was allowed to warm to room temperature and stirred for 8 h. Upon completion of the reaction, saturated NH_4Cl (aq) (20 mL) was added and the mixture was extracted with EA (20 mL×3). The organic phases were combined, dried over Na_2SO_4, filtered and concentrated under reduced pressure. The crude product was purified by column chromatography (eluent: EA/PE = 1/6) to afford **S14** as a white solid (0.30 g, 86% yield). ^{1}H NMR (500 MHz, $CDCl_3$) δ 7.01 (s, 1H), 6.51 (dd, J = 17.1, 11.1 Hz, 1H), 5.75 (d, J = 17.2 Hz, 1H), 5.43 (d, J = 10.9 Hz, 1H), 3.93 (s, 3H),

3.86 (s, 3H); ^{13}C NMR (126 MHz, $CDCl_3$) δ 154.4, 146.7, 144.1, 129.2, 127.2, 119.6, 109.6, 108.0, 60.9, 56.3; HRMS (ESI) Calcd. for $C_{10}H_{11}BNO_4$ $[M+H]^+$: 287.9871; Found: 287.9875.

Preparation of 2-bromo-4-fluoro-1-methoxy-3-vinylbenzene (S15)

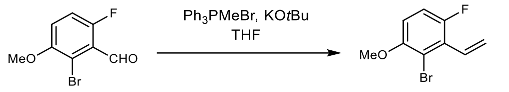

S16 **S15**

Substrate **S15** was prepared from **S16**[4] following similar procedure to that of **S14** and obtained in 91% yield). ^{1}H NMR (400 MHz, $CDCl_3$) δ 7.00-6.91 (m, 1H), 6.77 (dd, J = 17.8, 11.9 Hz, 1H), 6.67 (dd, J = 9.1, 4.4 Hz, 1H), 5.89 (dt, J = 17.9, 1.6 Hz, 1H), 5.69-5.55 (m, 1H), 3.80 (s, 2H); ^{13}C NMR (126 MHz, $CDCl_3$) δ 155.1 (d, J = 245.9 Hz), 152.4 (d, J = 2.6 Hz), 130.5 (d, J = 2.0 Hz), 126.8 (d, J = 15.3 Hz), 122.2 (d, J = 11.6 Hz), 114.7, 114.5, 110.3 (d, J = 9.2 Hz), 56.6; ^{19}F NMR (376 MHz, $CDCl_3$) δ -122.0; HRMS (EI) Calcd. for C_8H_8BrFO $[M]^+$: 229.9737; Found: 229.9734.

Preparation of (5-methoxy-2-vinylphenyl)boronic acid (S17)

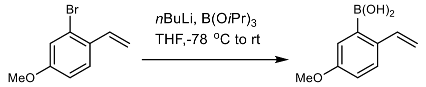

S17

Boronic acid **S17** was reported as a known compound and was prepared following the reported procedure.[5] ^{1}H NMR (500 MHz, $CDCl_3$) δ 7.81 (dd, J = 17.5, 10.9 Hz, 1H), 7.70 (s, 1H), 7.60 (dd, J = 8.4, 3.6 Hz, 1H), 7.11-7.02 (m, 1H), 5.62 (d, J = 17.5 Hz, 1H), 5.24 (d, J = 10.9 Hz, 1H), 3.86 (d, J = 3.9 Hz, 3H); ^{13}C NMR (126 MHz, $CDCl_3$) δ 158.5, 138.4, 137.3, 127.2, 120.7, 119.1, 113.5, 55.1; HRMS (ESI) Calcd. for $C_9H_{11}BNaO_3$ $[M+Na]^+$: 201.0699; Found: 201.0704.

Preparation of (6-vinylbenzo[*d*][1,3]dioxol-5-yl)boronic acid (S18)

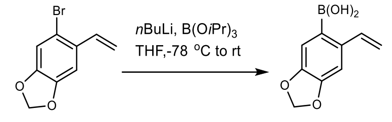

S18

Boronic acid **S18** was prepared following a similar procedure to the synthesis of **S17**. ^{1}H NMR (500 MHz, DMSO-d_6) δ 8.09 (s, 2H), 7.20 (s, 1H), 7.14 (dd, J = 17.5, 11.0 Hz, 1H), 6.95 (s, 1H), 5.99 (s, 2H), 5.60 (d, J = 17.5 Hz, 1H), 5.14-5.03 (m, 1H); ^{13}C NMR (126 MHz, DMSO-d_6) δ 148.4, 146.3, 137.3, 135.9, 112.4, 112.3, 104.2, 100.8; HRMS (ESI) Calcd. for $C_9H_9BNaO_4$ $[M+Na]^+$: 215.0492; Found: 215.0496.

Preparation of 1-(3-bromo-4,5-dimethoxy-2-nitrobenzyl)piperidine (S19)

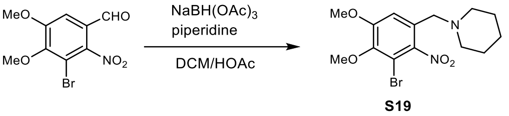

S19

A mixture of piperidine (0.23 mL, 2.59 mmol), 3-bromo-4,5-dimethoxy-2-nitrobenzaldehyde (0.50 g, 1.72 mL), and HOAc (50 μL) in DCM (10 mL) was stirred

at room temperature for 1 h. To the reaction solution was added $NaBH(OAc)_3$ (0.55 g, 2.59 mmol) and the resulting mixture was stirred for 20 h at room temperature. Upon completion of the reaction, saturated $NaHCO_3$ (aq) (20 mL) was added and the mixture was extracted with EA (20 mL×3). The organic phases were combined, dried over Na_2SO_4, filtered and concentrated under reduced pressure. The crude product was purified by column chromatography (gradient eluent: EA/PE = 1/3 to 1/1) to afford **S19** as a yellow oil (0.51 g, 82% yield). ^{1}H NMR (400 MHz, $CDCl_3$) δ 7.03 (s, 1H), 3.91 (s, 3H), 3.85 (s, 3H), 3.39 (s, 2H), 2.30 (s, 4H), 1.51 (dt, *J* = 10.5, 5.4 Hz, 4H), 1.39 (d, *J* = 4.7 Hz, 2H); ^{13}C NMR (126 MHz, $CDCl_3$) δ 154.2, 145.7, 144.7, 129.3, 112.1, 110.0, 109.9, 60.8, 59.0, 56.3, 54.4, 25.8, 24.1; HRMS (ESI) Calcd. for $C_{14}H_{20}BrN_2O_4$ $[M+H]^+$: 359.0606; Found: 359.0610.

Preparation of (1R,2S)-1-((3-bromo-4,5-dimethoxy-2-nitrobenzyl)amino)-2,3-dihydro-1H-inden-2-ol (S20)

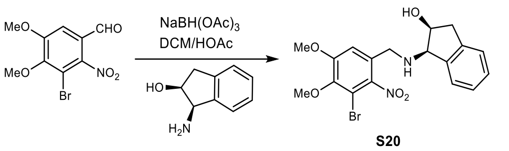

S20

Compound **S20** was prepared from 3-bromo-4,5-dimethoxy-2-nitrobenzaldehyde (0.30 g, 1.03 mmol) and (1*R*,2*S*)-1-amino-2,3-dihydro-1H-inden-2-ol (0.23 g, 1.55 mmol) following a similar procedure to the synthesis of **S19** and was obtained as a colorless oil (0.29 g, 66% yield). $[\alpha]^{25}_D$ = 9.96 (*c* = 0.5, $CHCl_3$); ^{1}H NMR (500 MHz, $CDCl_3$) δ 7.30-7.27 (m, 1H), 7.22 (dd, *J* = 7.1, 3.1 Hz, 3H), 7.07 (s, 1H), 4.44 (td, *J* = 5.2, 2.6 Hz, 1H), 4.05 (d, *J* = 5.1 Hz, 1H), 3.98-3.90 (m, 5H), 3.87 (d, *J* = 6.2 Hz, 3H), 3.05 (dd, *J* = 16.4, 5.2 Hz, 1H), 2.94 (dd, *J* = 16.4, 2.5 Hz, 1H); ^{13}C NMR (126 MHz, $CDCl_3$) δ 154.7, 146.4, 145.0, 141.8, 140.7, 129.5, 128.3, 127.0, 125.7, 124.1), 112.2, 110.3, 71.2, 65.6, 61.0, 56.5, 48.6, 39.8; HRMS (ESI) Calcd. for $C_{18}H_{20}BrN_2O_5$ $[M+H]^+$: 423.0556; Found: 423.0566.

Preparation of (5-aminonaphthalen-1-yl)boronic acid (S21)

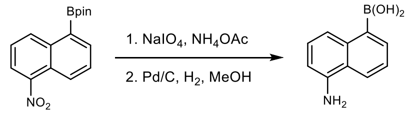

S21

To a solution of 4,4,5,5-tetramethyl-2-(5-nitronaphthalen-1-yl)-1,3,2-dioxaborolane[6] (0.45 g, 1.51 mmol) in acetone (10 mL) and H_2O (3 mL) was added NH_4OAc (0.23 g, 3.01 mmol) and $NaIO_4$ (0.96 g, 4.52 mmol), and the resulting mixture was stirred at room temperature for 12 h. Another portion of $NaIO_4$ (0.96 g, 4.52 mmol) was added and the reaction suspension was stirred for another 12 h. Saturated $NaHCO_3$ (aq) (10 mL) was added and the mixture was extracted with EA (20 mL×3). The organic phases were combined, washed once with brine (10 mL), dried over Na_2SO_4, filtered and concentrated under reduced pressure. The crude boronic acid was dissolved in methanol (15 mL) and 10 wt% Pd/C (45.0 mg) was added to the solution. The mixture was stirred under 1 atm H_2 at room temperature for 8 h. Upon completion of the reaction, the

mixture was diluted with EA (20 mL) and filtered through a layer of Celite. The Pd catalyst was wash twice with EA (10 mL). The filtrate was concentrated under reduced pressure and the crude product was purified by column chromatography (gradient eluent: EA to EA/MeOH = 8:1) to afford **S21** as a yellow solid (0.15 g, 54% yield over two steps). ^{1}H NMR (500 MHz, DMSO-*d*$_6$) δ 8.19 (s, 2H), 8.04 (d, *J* = 8.4 Hz, 1H), 7.56 (d, *J* = 6.5 Hz, 1H), 7.47 (d, *J* = 8.3 Hz, 1H), 7.31 (dd, *J* = 8.4, 6.8 Hz, 1H), 7.16 (t, *J* = 7.9 Hz, 1H), 6.65 (d, *J* = 7.3 Hz, 1H), 5.59 (s, 2H); ^{13}C NMR (151 MHz, DMSO-*d*$_6$) δ 144.8, 136.4, 135.6, 130.9, 126.2, 123.1, 122.8, 122.4, 116.8, 107.2; HRMS (ESI) Calcd. for $C_{10}H_{10}BNNaO_2$ [M+Na]$^+$: 210.0702; Found: 210.0701.

Preparation of 2-bromo-3-methoxybenzenethiol (S22)

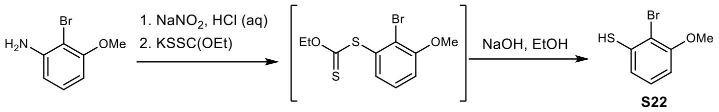

S22

To a suspension of 2-bromo-3-methoxyaniline (2.50 g, 12.4 mmol) in 3 N HCl (20 mL) at 0 °C was added a solution of $NaNO_2$ (0.95 g, 13.6 mmol) in H_2O (2.0 mL) over a period of 20 min. The resulting mixture was stirred at the same temperature for 1 h and then transferred dropwise to a preheated solution of KSSC(OEt) (4.0 g, 24.8 mmol) in H_2O (10 mL) at 60 °C. Upon completion of transfer, the resulting mixture was stirred at 80 °C for 2 h and then cooled to room temperature. The mixture was extracted with EA (20 mL×3). The organic phases were combined, washed with brine (15 mL), dried over Na_2SO_4, filtered and concentrated under reduced pressure. The obtained brown residue was dissolved in EtOH (18.0 mL)/H_2O (2.0 mL) and NaOH (1.20 g, 37.2 mmol) was added. The mixture was stirred at 60 °C for 6 h and then cooled to room temperature. The pH of the reaction solution was adjusted by addition of 3 N HCl (aq) to 3-4 and the mixture was extracted with EA (15 mL×3). The organic phases were combined, washed with brine (15 mL), dried over Na_2SO_4, filtered and concentrated under reduced pressure. The crude product was purified by column chromatography (eluent: EA/PE = 1:20) to afford **S22** as a yellow oil (1.71 g, 63% yield). ^{1}H NMR (400 MHz, $CDCl_3$) δ 7.80 (dt, *J* = 11.6, 5.8 Hz, 1H), 7.52-7.44 (m, 1H), 7.16 (dd, *J* = 8.3, 1.3 Hz, 1H), 3.95 (d, *J* = 10.0 Hz, 3H), 3.29 (d, *J* = 9.5 Hz, 3H); ^{13}C NMR (126 MHz, $CDCl_3$) δ 157.1, 140.9, 128.7, 122.4, 116.7, 110.4, 57.0, 42.3; HRMS (EI) Calcd. for C_7H_7BrOS [M]$^+$: 217.9401; Found: 217.9405.

Preparation of 2-bromo-3-methoxybenzenesulfonyl fluoride (S23)

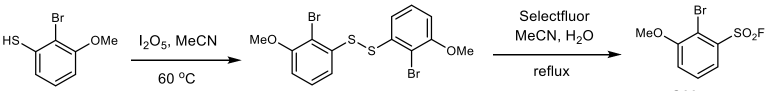

S23

To a solution of 2-bromo-3-methoxybenzenethiol (4.50 g, 20.5 mmol) in MeCN (50 mL) was added I_2O_5 (6.90 g, 20.5 mmol) and the mixture was stirred at 60 °C for 2 h. Upon completion, the mixture was filtered through a layer of Celite and the filtrate was concentrated. The residue was partitioned between EA (20 mL) and H_2O (15 mL). The organic layer was separated and the aqueous phase was extracted with EA (15 mL×3). The organic phases were combined, dried over Na_2SO_4, filtered and concentrated under

reduced pressure. The obtained disulfide was dissolved in MeCN (30 mL)/H_2O (3 mL) and the mixture was stirred for 1.5 h at reflux. Upon full consumption of disulfide, the reaction system was cooled to room temperature and concentrated under reduced pressure. The residue was partitioned between EA (20 mL) and H_2O (15 mL). The organic layer was separated and the aqueous phase was extracted with EA (15 mL×3). The organic phases were combined, dried over Na_2SO_4, filtered and concentrated under reduced pressure. The crude product was purified by column chromatography (eluent: EA/PE = 1:10) to afford **S23** as a yellow solid (3.35 g, 61% yield). ^{1}H NMR (500 MHz, $CDCl_3$) δ 7.67 (dd, J = 8.0, 1.0 Hz, 1H), 7.48 (td, J = 8.2, 1.3 Hz, 1H), 7.25-7.20 (m, 1H), 3.96 (s, 3H); ^{13}C NMR (126 MHz, $CDCl_3$) δ 157.8, 135.2 (d, J = 23.1 Hz), 128.9, 123.3, 118.1, 110.9, 57.1; ^{19}F NMR (376 MHz, $CDCl_3$) δ -208.8; HRMS (EI) Calcd. for $C_7H_6BrFO_3S$ $[M]^+$: 267.9205; Found: 267.9209.

Preparation of 4-amino-2-bromo-3-methoxybenzenesulfonyl fluoride (S25)

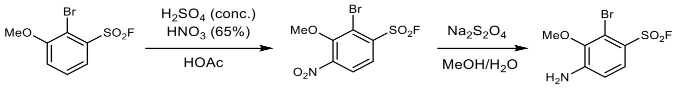

S24 S25

To a solution of 2-bromo-3-methoxybenzenesulfonyl fluoride (0.70 g, 2.60 mmol) in HOAc (2 mL) and conc. H_2SO_4 (4 mL) at 0 °C was added 65% HNO_3 (2.4 mL, 26.0 mmol). The resulting mixture was allowed to warm to rt and stirred for 3 h. The reaction system was poured into icy water (15 mL) and the mixture was extracted with DCM (10 mL×2). The organic phases were combined and washed successively with brine (10 mL) and saturated $NaHCO_3$ (10 mL). The organic phase was separated, dried over Na_2SO_4, filtered and concentrated under reduced pressure. The crude product was purified by column chromatography (eluent: EA/PE = 1:6) to afford **S24** as a yellow solid (0.69 g, 86% yield). ^{1}H NMR (500 MHz, $CDCl_3$) δ 7.72 (d, J = 9.0, 1H), 7.27 (d, J = 9.0 Hz, 1H), 4.06 (s, 3H); ^{13}C NMR (126 MHz, $CDCl_3$) δ 159.5, 144.2, 128.8 (d, J = 25.0 Hz), 125.0, 116.0, 114.7 (d, J = 1.5 Hz), 57.9; ^{19}F NMR (376 MHz, $CDCl_3$) δ 64.3; HRMS (EI) Calcd. for $C_7H_5BrFNO_5S$ $[M]^+$: 312.9056; Found: 312.9048.

To a solution of **S24** (0.69 g, 2.20 mmol) in MeOH (10 mL)/H_2O (2 mL) was added $Na_2S_2O_4$ (1.53 g, 8.80 mmol) and the mixture was stirred at room temperature for 10 h. Upon completion of the reaction, the mixture was diluted with EA (20 mL) and washed with brine (10 mL×2). The organic phase was separated, dried over Na_2SO_4, filtered and concentrated under reduced pressure. The crude product was purified by column chromatography (eluent: EA/PE = 1:6) to afford **S25** as a yellow oil (0.54 g, 87% yield). ^{1}H NMR (600 MHz, $CDCl_3$) δ 7.10 (d, J = 9.2 Hz, 1H), 6.74 (dd, J = 9.2, 1.3 Hz, 1H), 5.25 (s, 2H), 3.85 (s, 3H); ^{13}C NMR (151 MHz, $CDCl_3$) δ 149.1, 144.3, 122.8, 118.8 (d, J = 1.2 Hz), 114.3 (d, J = 20.8 Hz), 112.0 (d, J = 2.6 Hz), 58.2; ^{19}F NMR (376 MHz, $CDCl_3$) δ 69.5; HRMS (EI) Calcd. for $C_7H_7BrFNO_3S$ $[M]^+$: 282.9314; Found: 282.9310.

Preparation of 4-formyl-2-methoxy-6-nitrophenyl trifluoromethanesulfonate (S27)

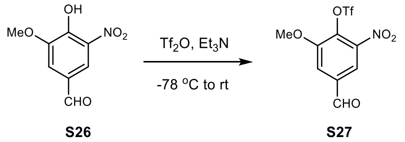

S26 S27

Compound **S26** was prepared following a reported procedure.[7] ^{1}H NMR (500 MHz, $CDCl_3$) δ 11.26 (s, 1H), 9.89 (s, 1H), 8.22 (s, 1H), 7.64 (s, 1H), 4.02 (s, 3H); ^{13}C NMR (151 MHz, $CDCl_3$) δ 189.0, 151.3 (d, J = 18.6 Hz), 133.6, 128.1, 121.5, 113.6, 57.1; HRMS (ESI) Calcd. for $C_8H_8NO_5$ $[M+H]^+$: 198.0402; Found: 198.0400.
To a solution of phenol **S26** (7.0 g, 35.5 mmol) in DCM (40 mL) at -78 °C was added Tf_2O (7.8 mL, 46.2 mmol), followed by Et_3N (6.5 mL, 46.2 mmol). The resulting solution was allowed to warm to room temperature over 1 h and then stirred at rt for 3 h. Upon completion, the reaction system was diluted with DCM (30 mL) and the mixture was washed successively with NH_4Cl (aq) (20 mL) and brine (20 mL). The organic phase was separated, dried over Na_2SO_4, filtered and concentrated under reduced pressure. The crude product was purified by column chromatography (eluent: EA/PE = 1:8) to afford **S27** as a yellow solid (10.2 g, 87% yield). ^{1}H NMR (500 MHz, $CDCl_3$) δ 10.03 (s, 1H), 8.14 (s, 1H), 7.82 (s, 1H), 4.09 (s, 3H); ^{13}C NMR (126 MHz, $CDCl_3$) δ 188.2, 153.8, 143.3, 135.6, 135.4, 119.4, 118.6 (q, J = 321.1 Hz), 115.7, 57.6; ^{19}F NMR (376 MHz, $CDCl_3$) δ 73.0; HRMS (ESI) Calcd. for $C_9H_7F_3NO_7S$ $[M+H]^+$: 329.9895; Found: 329.9901.

Preparation of 2-bromo-1-methoxy-3-(methylsulfonyl)benzene (S29)

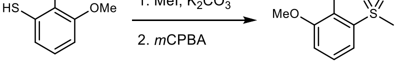

S28 **S29**

To a solution of 2-bromo-3-methoxybenzenethiol (0.45 g, 2.05 mmol) in MeCN (15 mL) was added K_2CO_3 (0.42 g, 3.08 mmol) and MeI (0.25 mL, 4.10 mmol). The mixture was stirred at rt for 12 h and then concentrated under reduced pressure. The residue was partitioned between EA (20 mL) and water (15 mL). The organic phase was separated and the aqueous phase was extracted with EA (10 mL×2). The organic layers were combined, dried over Na_2SO_4, filtered and concentrated under reduced pressure. The obtained oil was dissolved in DCM (15 mL) and mCPBA (85%, 1.25 g, 6.15 mmol) was added in three portions over 15 min. The mixture was stirred at rt for 1 h. Upon completion, the reaction system was diluted with DCM (15 mL) and the solution was washed successively with saturated $NaHCO_3$ (10 mL) and brine (10 mL). The organic layer was separated, dried over Na_2SO_4, filtered and concentrated under reduced pressure. The crude product was purified by column chromatography (eluent: EA/PE = 1:6) to afford **S29** as a white solid (0.49 g, 91% yield). ^{1}H NMR (500 MHz, $CDCl_3$) δ 7.77 (dd, J = 7.9, 1.1 Hz, 1H), 7.46 (t, J = 8.1 Hz, 1H), 7.19-7.12 (m, 1H), 3.94 (s, 3H), 3.28 (s, 3H); ^{13}C NMR (126 MHz, $CDCl_3$) δ 157.3, 141.1, 128.8, 122.6, 116.7, 110.7, 57.1, 42.4; HRMS (EI) Calcd. for $C_8H_9BrO_3S$ $[M]^+$: 263.9456; Found: 263.9459.

Preparation of 4-((2-bromo-3-methoxyphenyl)sulfonyl)morpholine (S30)

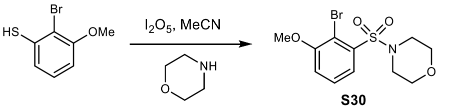

S30

To a solution of 2-bromo-3-methoxybenzenethiol (1.80 g, 8.22 mmol) in MeCN (30

mL) was added I_2O_5 (2.70 g, 8.22 mmol) and morpholine (1.50 mL, 16.44 mmol). The mixture was stirred at 60 °C for 12 h and then concentrated under reduced pressure. The residue was partitioned between EA (30 mL) and water (15 mL). The organic phase was separated and the aqueous phase was extracted with EA (20 mL×2). The organic layers were combined, dried over Na_2SO_4, filtered and concentrated under reduced pressure. The crude product was purified by column chromatography (eluent: EA/PE = 1:5) to afford **S30** as a pink solid (2.41 g, 87% yield). ^{1}H NMR (500 MHz, $CDCl_3$) δ 7.29 (t, J = 8.0 Hz, 1H), 7.20 (dd, J = 8.0, 1.2 Hz, 1H), 3.88 (s, 3H), 3.82-3.75 (m, 4H), 3.12-3.06 (m, 4H); ^{13}C NMR (126 MHz, $CDCl_3$) δ 156.2, 142.6, 128.2, 116.7, 108.3, 105.7, 68.0, 56.5, 56.1; HRMS (ESI) Calcd. for $C_{11}H_{15}BrNO_4S$ $[M+H]^+$: 335.9905; Found: 335.9902.

1.4 Asymmetric Cross-Coupling for Chiral Biaryl Synthesis

General procedure for the asymmetric Suzuki-Miyaura coupling.

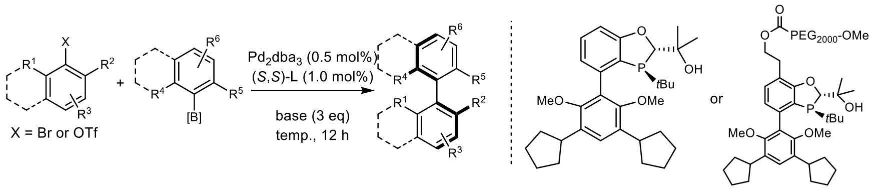

To a mixture of aryl halide or triflate (0.20 mmol), arylboronic acid or ester (0.30 mmol), base (0.60 mmol), $Pd_2(dba)_3$ (1.0 μmol) and (*S*,*S*)-L (2.0 μmol, Pd/ligand mol ratio: 1/1) under N_2 was charged degassed solvent. The mixture was stirred at noted temperature for noted time. Ethyl acetate (15 mL) was added and the organic phase was washed sequentially with water (5 mL) and brine (5 mL). The organic layer was separated, dried over Na_2SO_4, filtered and concentrated. The crude enantioenriched chiral biaryl product was purified by silica gel flash column chromatography. A mixture of DCE/H_2O (5:1) was used as solvent for sulfonyl-substituted substrates and the reaction was conducted at 35 °C for 48 h unless otherwise specified. H_2O was used as solvent for other substrates and the reaction was conducted at 30 °C for 12 h unless otherwise specified.

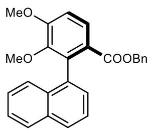

3a

benzyl (*R*)-3,4-dimethoxy-2-(naphthalen-1-yl)benzoate (3a)
Compound **3a** was obtained from the coupling reaction between benzyl 2-bromo-3,4-dimethoxybenzoate[8] and 1-naphthalene boronic acid in 79% yield and 93% ee. $[\alpha]^{25}_D$ = -32.0 (*c* = 0.5, $CHCl_3$); ^1H NMR (500 MHz, $CDCl_3$) δ 7.90 (d, *J* = 8.7 Hz, 1H), 7.85 (d, *J* = 7.9 Hz, 1H), 7.79 (d, *J* = 8.2 Hz, 1H), 7.47-7.41 (m, 3H), 7.32 (t, *J* = 7.6 Hz, 1H), 7.28 (d, *J* = 7.0 Hz, 1H), 7.19 (t, *J* = 7.3 Hz, 1H), 7.13 (t, *J* = 7.4 Hz, 2H), 7.05 (d, *J* = 8.8 Hz, 1H), 6.72 (d, *J* = 7.4 Hz, 2H), 4.76 (s, 2H), 3.98 (s, 3H), 3.39 (s, 3H); ^{13}C NMR (126 MHz, $CDCl_3$) δ 166.8, 156.3, 147.4, 136.3, 135.5, 135.2, 133.3, 132.7, 128.3, 128.2, 128.0, 127.8, 127.6, 127.6, 126.3, 125.9, 125.8, 125.5, 125.1, 124.5, 111.1, 66.6, 60.9, 56.0.; HRMS (ESI) Calcd. for $C_{26}H_{23}O_4$ $[M+H]^+$: 399.1596; Found: 399.1594; Enantiomeric excess was determined by chiral HPLC (Chiralcel OD-3, 25 °C, flow rate: 0.7 mL/min, hexanes/isopropanol: 90/10, 214 nm, 14.70 min (major isomer), 17.39 min (minor isomer)).

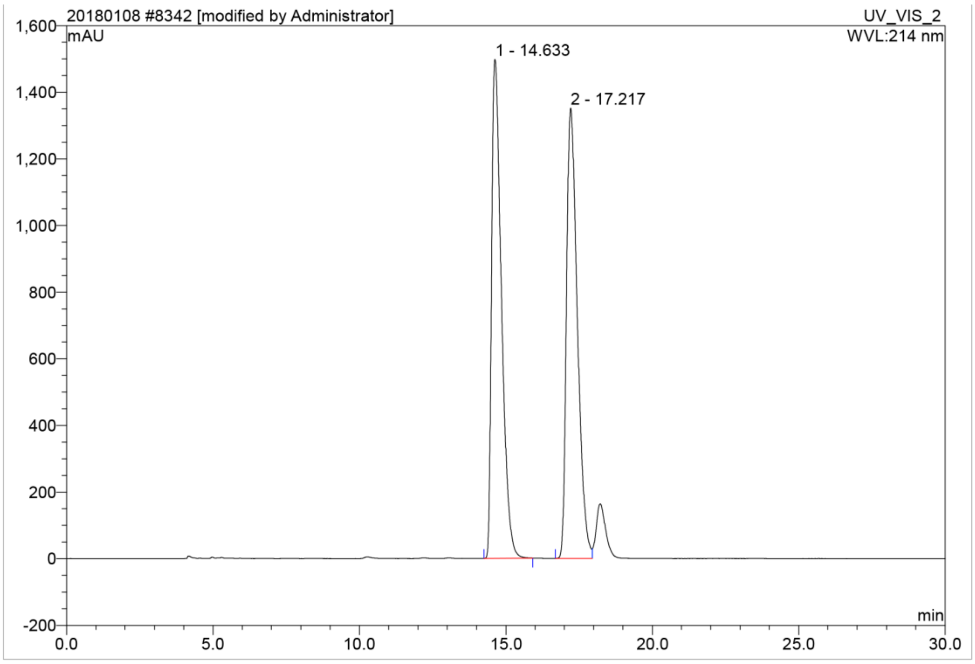

No.	Ret.Time min	Peak Name	Height mAU	Area mAU*min	Rel.Area %	Amount	Type
1	14.63	n.a.	1497.995	583.524	50.01	n.a.	BMB
2	17.22	n.a.	1351.884	583.263	49.99	n.a.	BM *
Total:			2849.879	1166.788	100.00	0.000	

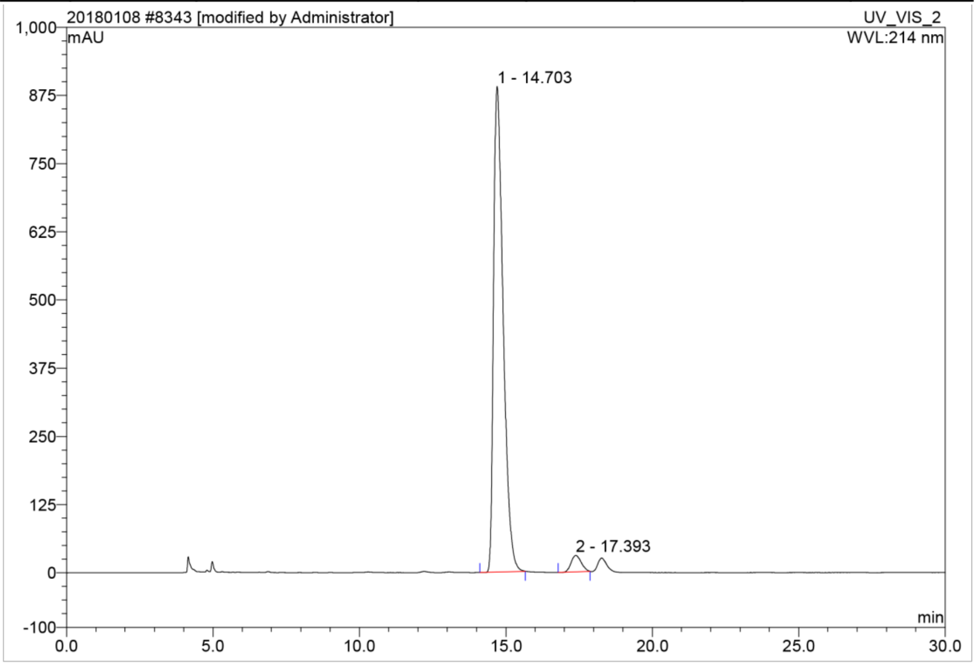

No.	Ret.Time min	Peak Name	Height mAU	Area mAU*min	Rel.Area %	Amount	Type
1	14.70	n.a.	890.311	338.773	96.58	n.a.	BMB*
2	17.39	n.a.	30.162	12.002	3.42	n.a.	BMB*
Total:			920.473	350.775	100.00	0.000	

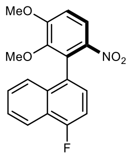

3b

(*R*)-1-(2,3-dimethoxy-6-nitrophenyl)-4-fluoronaphthalene (3b)

Compound **3b** was obtained from the coupling reaction between 2-bromo-3,4-dimethoxy-1-nitrobenzene and 4-Fluoronaphthalene-1-boronic acid in 87% yield and 95% ee. $[\alpha]^{25}_{D}$ = -110.9 (c = 0.3, $CHCl_3$); ^1H NMR (500 MHz, $CDCl_3$) δ 8.17 (d, J = 8.4 Hz, 1H), 7.97 (d, J = 9.1 Hz, 1H), 7.54 (t, J = 7.5 Hz, 1H), 7.43 (dt, J = 17.2, 8.4 Hz, 2H), 7.21 (dt, J = 17.9, 7.8 Hz, 2H), 7.08 (d, J = 9.2 Hz, 1H), 4.01 (s, 3H), 3.44 (s, 3H); ^{13}C NMR (126 MHz, $CDCl_3$) δ 158.7 (d, J = 252.6 Hz), 157.1, 147.7, 143.0, 133.4 (d, J = 4.8 Hz), 130.2, 127.5 (d, J = 4.6 Hz), 127.3, 126.2 (d, J = 1.8 Hz), 125.8 (d, J = 8.4 Hz), 125.1 (d, J = 2.7 Hz), 123.7 (d, J = 16.7 Hz), 121.5, 120.9 (d, J = 5.4 Hz), 110.9, 108.9 (d, J = 20.5 Hz), 61.0, 56.3; ^{19}F NMR (282 MHz, $CDCl_3$) δ -123.4; HRMS (ESI) Calcd. for $C_{18}H_{15}NFO_4$ $[M+H]^+$: 328.0985; Found: 328.0990; Enantiomeric excess was determined by chiral HPLC (Chiralcel OD-3, 25 ºC, flow rate: 0.7 mL/min, hexanes/isopropanol: 80/20, 214 nm, 9.84 min (major isomer), 10.70 min (minor isomer)).

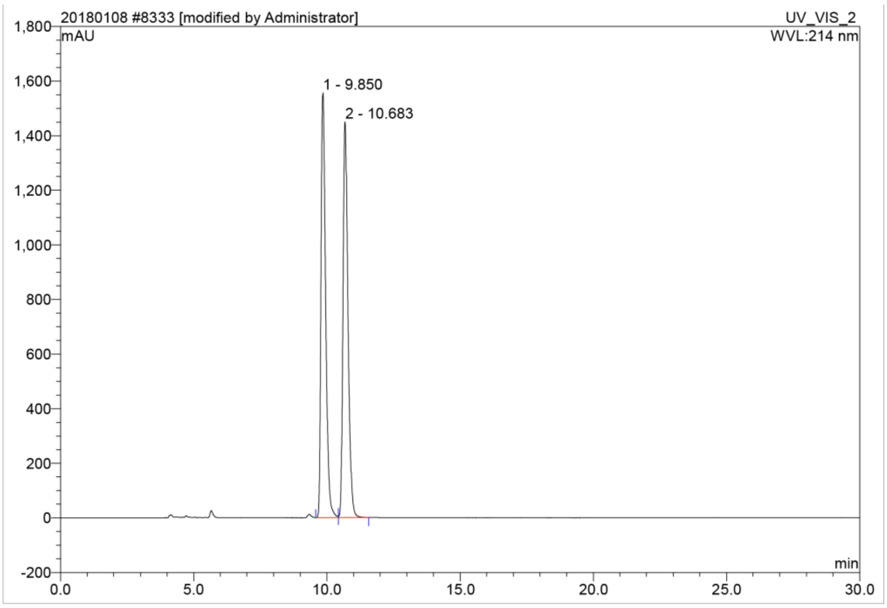

No.	Ret.Time min	Peak Name	Height mAU	Area mAU*min	Rel.Area %	Amount	Type
1	9.85	n.a.	1556.700	315.164	50.04	n.a.	BM *
2	10.68	n.a.	1450.098	314.674	49.96	n.a.	MB*
Total:			3006.798	629.838	100.00	0.000	

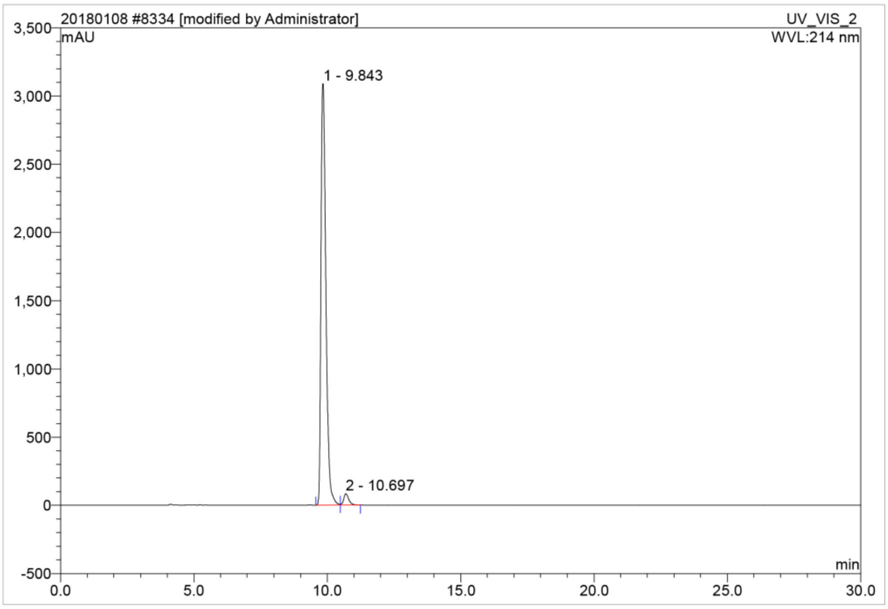

No.	Ret.Time min	Peak Name	Height mAU	Area mAU*min	Rel.Area %	Amount	Type
1	9.84	n.a.	3090.537	660.416	97.26	n.a.	BM
2	10.70	n.a.	82.980	18.596	2.74	n.a.	MB
Total:			3173.517	679.012	100.00	0.000	

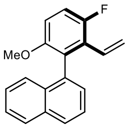

3c

(*R*)-1-(3-fluoro-6-methoxy-2-vinylphenyl)naphthalene (3c)

Compound **3c** was obtained from the coupling reaction between **S15** and 1-naphthaleneboronic acid in 94% yield and 96% ee. $[\alpha]^{25}_{D}$ = 37.2 (*c* = 0.2, $CHCl_3$); ^{1}H NMR (400 MHz, $CDCl_3$) δ 7.89 (dd, *J* = 8.1, 5.1 Hz, 2H), 7.54 (dd, *J* = 8.2, 7.1 Hz, 1H), 7.47 (ddd, *J* = 8.1, 5.6, 2.5 Hz, 1H), 7.37 (dd, *J* = 5.6, 1.1 Hz, 2H), 7.28 (dd, *J* = 7.0, 1.0 Hz, 1H), 7.14 (dd, *J* = 10.8, 9.1 Hz, 1H), 6.87 (dd, *J* = 9.0, 4.1 Hz, 1H), 5.98 (dd, *J* = 17.9, 11.9 Hz, 1H), 5.70 (dt, *J* = 17.9, 1.6 Hz, 1H), 5.15 (ddd, *J* = 11.9, 2.6, 1.9 Hz, 1H), 3.58 (s, 3H); ^{13}C NMR (151 MHz, $CDCl_3$) δ 156.9, 155.3, 153.8 (d, *J* = 2.0 Hz), 134.3 (d, *J* = 1.9 Hz), 133.6, 132.4, 130.0 (d, *J* = 3.5 Hz), 129.8 (d, *J* = 1.1 Hz), 128.4, 128.0 (d, *J* = 5.3 Hz), 126.2, 126.1 (d, *J* = 12.3 Hz), 125.9, 125.9, 125.6, 120.6 (d, *J* = 12.6 Hz), 115.5 (d, *J* = 24.9 Hz), 110.6 (d, *J* = 9.2 Hz), 56.5; ^{19}F NMR (376 MHz, $CDCl_3$) δ -124.5; HRMS (EI) Calcd. for $C_{19}H_{15}FO$ $[M]^+$: 278.1107; Found: 278.1103; Enantiomeric excess was determined by chiral HPLC (Chiralcel OJ-3, 25 ºC, flow rate: 0.8 mL/min, hexanes/isopropanol: 90/10, 250 nm, 6.28 min (minor isomer), 6.79 min (major isomer).

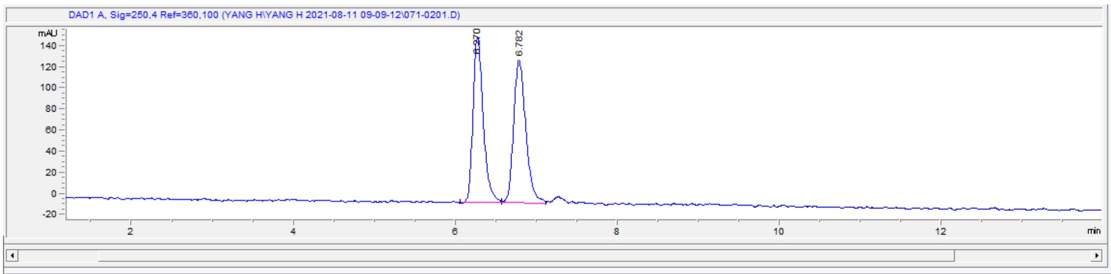

#	Time 时间	Area 峰面积	Height 峰高	Width 峰宽	Symmetry factor 对称因子	Area% 峰面积 %	Area%
1	6.27	1392.4	157.5	0.1331	0.732	49.491	49.491
2	6.782	1421	136.2	0.1598	0.758	50.509	50.509

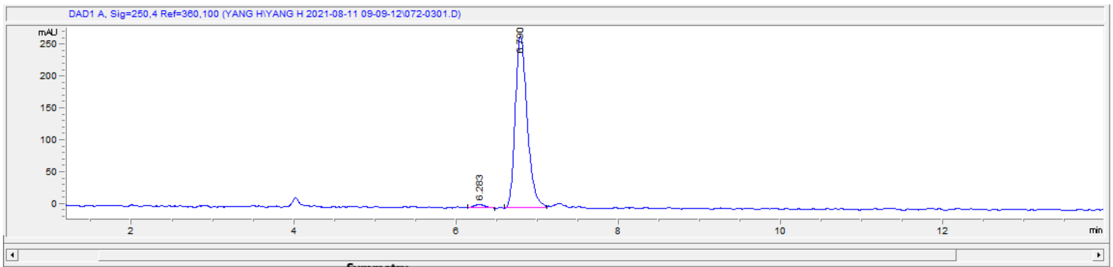

#	Time 时间	Area 峰面积	Height 峰高	Width 峰宽	Symmetry factor 对称因子	Area% 峰面积 %	Area%
1	6.283	62.2	6.1	0.1332	0.866	2.197	2.197
2	6.79	2768.2	269.9	0.1557	0.73	97.803	97.803

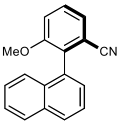

3d

(*R*)-3-methoxy-2-(naphthalen-1-yl)benzonitrile (3d)

Compound **3d** was obtained from the coupling reaction between 2-bromo-3-methoxybenzonitrile and 1-naphthaleneboronic acid in 93% yield and 88% ee. $[\alpha]^{25}_{D}$ = -45.2 (*c* = 0.2, $CHCl_3$); ^1H NMR (600 MHz, $CDCl_3$) δ 7.93 (dd, *J* = 15.8, 8.2 Hz, 2H), 7.58 (dd, *J* = 8.2, 7.1 Hz, 1H), 7.50 (dt, *J* = 8.0, 4.6 Hz, 2H), 7.45-7.37 (m, 3H), 7.34 (d, *J* = 8.4 Hz, 1H), 7.25 (d, *J* = 8.4 Hz, 1H), 3.70 (s, 3H); ^{13}C NMR (151 MHz, $CDCl_3$) δ 157.9, 133.7, 133.5, 132.6, 131.9, 129.8, 129.3, 128.6, 127.9, 126.4, 126.1, 125.5, 125.4, 125.0, 117.9, 115.4, 115.3, 56.1; HRMS (ESI) Calcd. for $C_{18}H_{14}NO$ $[M+H]^+$: 260.1070; Found: 260.1068; Enantiomeric excess was determined by chiral HPLC (Chiralcel OD-3, 25 ºC, flow rate: 0.7 mL/min, hexanes/isopropanol: 90/10, 230 nm, 10.53 min (major isomer), 11.82 min (minor isomer).

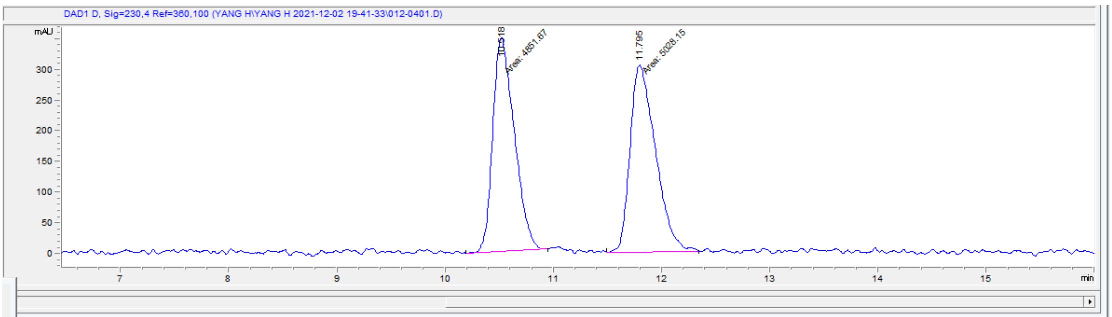

#	Time 时间	Area 峰面积	Height 峰高	Width 峰宽	Symmetry factor 对称因子	Area% 峰面积 %	Area%
1	10.518	4851.7	349.3	0.2315	0.674	49.107	49.107
2	11.795	5028.1	306.5	0.2734	0.601	50.893	50.893

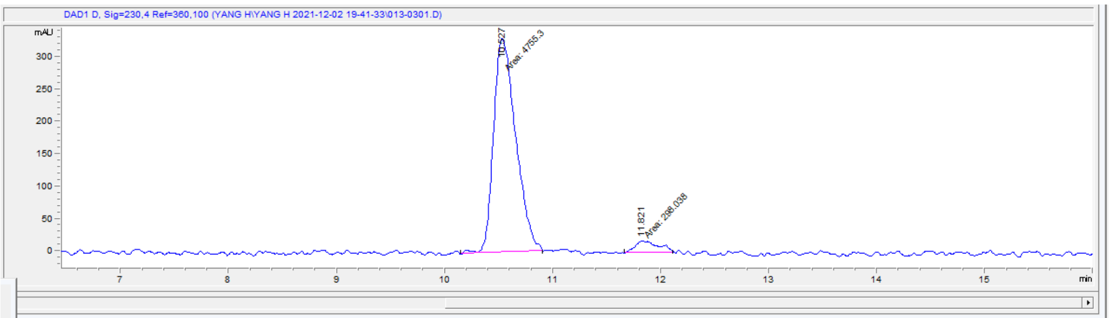

#	Time 时间	Area 峰面积	Height 峰高	Width 峰宽	Symmetry factor 对称因子	Area% 峰面积 %	Area%
1	10.527	4755.3	330.9	0.2395	0.589	94.102	94.102
2	11.821	298	18.9	0.2635	0.407	5.898	5.898

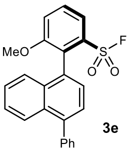

3e

(*S*)-3-methoxy-2-(4-phenylnaphthalen-1-yl)benzenesulfonyl fluoride (3e)

Compound **3e** was obtained from the coupling reaction between **S23** and 4-phenylnaphthalene-1-boronic acid in 91% yield and 97% ee. $[\alpha]^{25}_{D}$ = -42.2 (*c* = 0.5, $CHCl_3$); ^{1}H NMR (500 MHz, $CDCl_3$) δ 7.97 (d, *J* = 8.0 Hz, 1H), 7.92-7.87 (m, 1H), 7.68 (td, *J* = 8.3, 1.0 Hz, 1H), 7.62-7.56 (m, 2H), 7.54-7.48 (m, 3H), 7.47-7.34 (m, 5H), 7.30 (d, *J* = 7.7 Hz, 1H), 3.71 (s, 3H); ^{13}C NMR (126 MHz, $CDCl_3$) δ 159.2, 141.3, 140.7, 135.0 (d, *J* = 20.4 Hz), 132.7, 131.6, 130.4, 130.3, 129.8, 128.4, 127.6, 127.5, 126.6, 126.2, 126.1, 125.9, 125.7, 121.8, 117.6, 56.7; ^{19}F NMR (376 MHz, $CDCl_3$) δ 65.1; HRMS (EI) Calcd. for $C_{23}H_{17}FO_3S$ $[M]^+$: 392.0882; Found: 392.0878; Enantiomeric excess was determined by chiral HPLC (Chiralcel OD-3, 25 °C, flow rate: 0.6 mL/min, hexanes/isopropanol: 99/1, 254 nm, 20.54 min (minor isomer), 22.12 min (major isomer)).

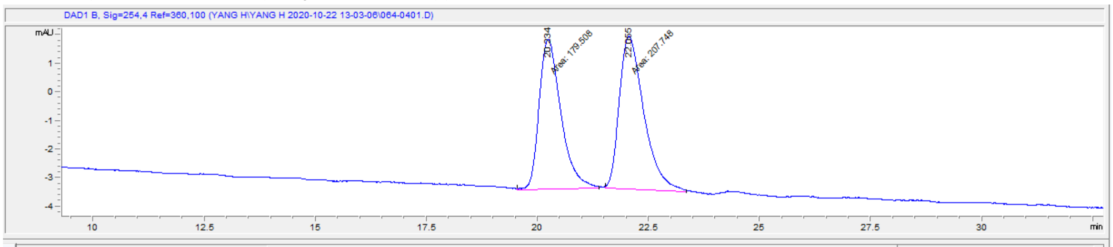

#	Time 时间	Area 峰面积	Height 峰高	Width 峰宽	Symmetry factor 对称因子	Area% 峰面积 %	Area%
1	20.234	179.5	5.2	0.5703	0.67	46.354	46.354
2	22.055	207.7	5.4	0.6461	0.619	53.646	53.646

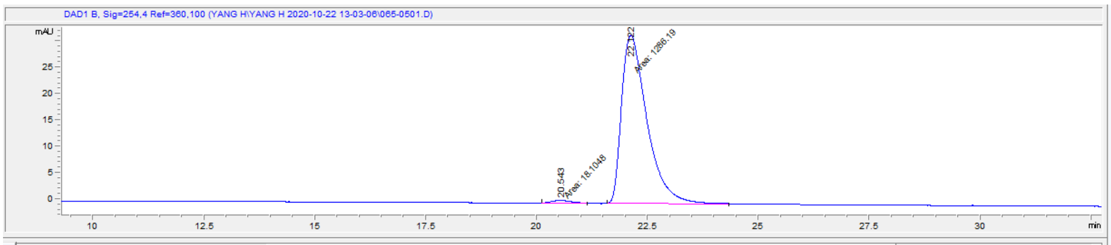

#	Time 时间	Area 峰面积	Height 峰高	Width 峰宽	Symmetry factor 对称因子	Area% 峰面积 %	Area%
1	20.543	18.1	5.4E-1	0.5537	0.751	1.388	1.388
2	22.122	1286.2	32	0.6694	0.548	98.612	98.612

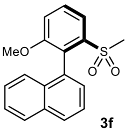

3f

(*S*)-1-(2-methoxy-6-(methylsulfonyl)phenyl)naphthalene (3f)

Compound **3f** was obtained from the coupling reaction between **S29** and 1-naphthalene boronic acid in 77% yield and 98% ee. $[\alpha]^{25}_{D}$ = -25.6 (*c* = 0.2, $CHCl_3$); ^{1}H NMR (400 MHz, $CDCl_3$) δ 7.93 (dd, *J* = 15.4, 8.0 Hz, 3H), 7.61 (dt, *J* = 15.2, 8.1 Hz, 2H), 7.54-7.50 (m, 1H), 7.47 (t, *J* = 7.0 Hz, 1H), 7.38 (t, *J* = 7.7 Hz, 1H), 7.30 (d, *J* = 8.2 Hz, 2H), 3.65 (s, 3H), 2.37 (s, 3H); ^{13}C NMR (151 MHz, $CDCl_3$) δ 158.7, 141.9, 133.3, 132.7, 131.1, 129.7, 129.6, 129.1 128.7, 128.4, 126.5, 126.0, 125.4, 125.3, 120.6, 116.0, 100.1, 56.6, 43.7; HRMS (ESI) Calcd. for $C_{18}H_{17}O_3S$ $[M+H]^+$: 313.0898; Found: 313.0891; Enantiomeric excess was determined by chiral HPLC (Chiralcel IC-3, 25 °C, flow rate: 0.8 mL/min, hexanes/isopropanol: 70/30, 290 nm, 32.83 min (minor isomer), 40.57 min (major isomer)).

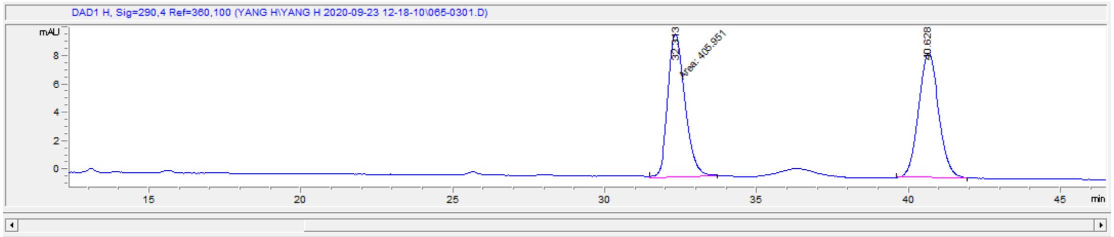

#	Time 时间	Area 峰面积	Height 峰高	Width 峰宽	Symmetry factor 对称因子	Area% 峰面积 %	Area%
1	32.313	406	10.1	0.6669	0.764	49.702	49.702
2	40.628	410.8	8.8	0.6642	0.909	50.298	50.298

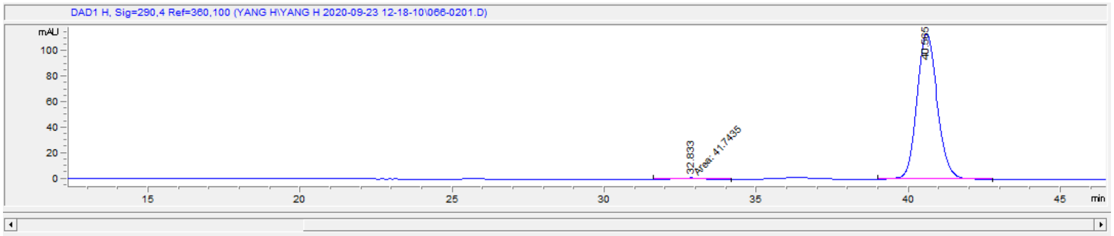

#	Time 时间	Area 峰面积	Height 峰高	Width 峰宽	Symmetry factor 对称因子	Area% 峰面积 %	Area%
1	32.833	41.7	9.8E-1	0.7112	1.065	0.771	0.771
2	40.565	5375.5	113.9	0.7286	0.831	99.229	99.229

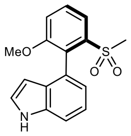

3g

(*S*)-4-(2-methoxy-6-(methylsulfonyl)phenyl)-1H-indole (3g)

Compound **3g** was obtained from the coupling reaction between **S29** and indole-4-boronic acid pinacol ester[9] in 82% yield and 85% ee. $[\alpha]^{25}_D$ = -30.0 (c = 0.5, $CHCl_3$); ^{1}H NMR (400 MHz, $CDCl_3$) δ 8.23 (s, 1H), 7.91 (d, J = 8.0 Hz, 1H), 7.57 (t, J = 8.2 Hz, 1H), 7.47 (d, J = 8.2 Hz, 1H), 7.33 (d, J = 7.4 Hz, 1H), 7.29 (d, J = 6.2 Hz, 2H), 7.19 (dd, J = 5.6, 1.7 Hz, 2H), 6.06 (s, 1H), 3.70 (s, 3H), 2.40 (s, 3H); ^{13}C NMR (151 MHz, $CDCl_3$) δ 158.7, 141.7, 135.4, 129.0, 129.0, 128.3, 125.8, 124.7, 123.3, 121.9, 120.2, 116.1, 111.6, 102.1, 56.7, 43.4; HRMS (ESI) Calcd. for $C_{16}H_{16}NO_3S$ $[M+H]^+$: 302.0851; Found: 302.0858; Enantiomeric excess was determined by chiral HPLC (Chiralcel OD-3, 25 °C, flow rate: 0.8 mL/min, hexanes/isopropanol: 70/30, 290 nm, 9.31 min (minor isomer), 10.84 min (major isomer)).

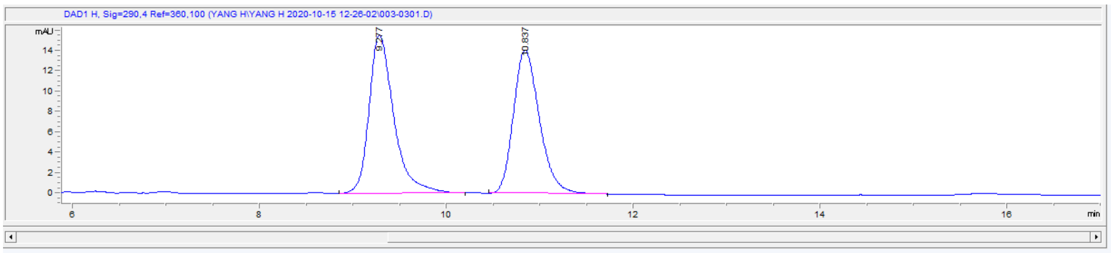

#	Time 时间	Area 峰面积	Height 峰高	Width 峰宽	Symmetry factor 对称因子	Area% 峰面积 %	Area%
1	9.277	286.5	15.6	0.2764	0.673	51.213	51.213
2	10.837	272.9	14.1	0.296	0.761	48.787	48.787

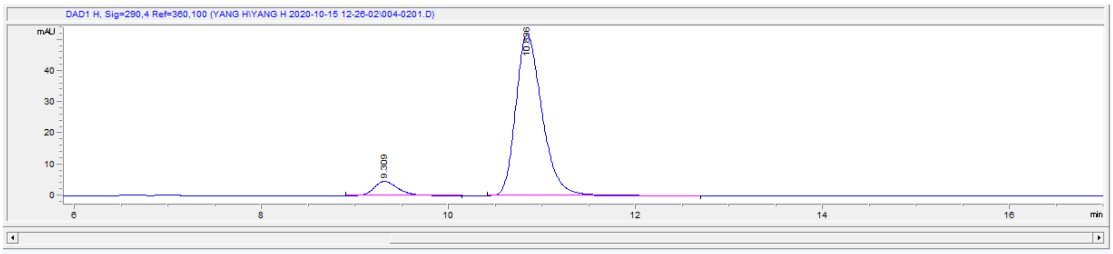

#	Time 时间	Area 峰面积	Height 峰高	Width 峰宽	Symmetry factor 对称因子	Area% 峰面积 %	Area%
1	9.309	85.1	4.6	0.2822	0.71	7.739	7.739
2	10.836	1014.6	51.6	0.3034	0.703	92.261	92.261

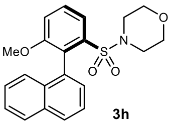

(*S*)-4-((3-methoxy-2-(naphthalen-1-yl)phenyl)sulfonyl)morpholine (3h)

Compound **3h** was obtained from the coupling reaction between **S30** and 1-naphthalene boronic acid in 80% yield and 88% ee. $[\alpha]^{25}_D$ = -23.0 (*c* = 0.5, $CHCl_3$); ^1H NMR (500 MHz, $CDCl_3$) δ 7.88 (dd, *J* = 8.4, 0.6 Hz, 2H), 7.53 (dd, *J* = 8.2, 7.0 Hz, 1H), 7.48-7.39 (m, 4H), 7.37-7.31 (m, 2H), 6.85 (dd, *J* = 7.5, 1.8 Hz, 1H), 3.66-3.63 (m, 4H), 3.63 (s, 3H), 2.85 (td, *J* = 3.9, 1.9 Hz, 4H); ^{13}C NMR (126 MHz, $CDCl_3$) δ 157.9, 142.1, 133.7, 133.4, 132.0, 129.0, 128.4, 128.4, 128.1, 126.0, 125.9, 125.7, 125.5, 124.1, 116.5), 107.9, 68.0, 56.2, 56.0; HRMS (ESI) Calcd. for $C_{21}H_{21}NNaO_4S$ $[M+Na]^+$: 406.1089; Found: 406.1080; Enantiomeric excess was determined by chiral HPLC (Chiralcel OJ-3, 25 °C, flow rate: 0.7 mL/min, hexanes/isopropanol: 95/5, 230 nm, 24.49 min (minor isomer), 27.34 min (major isomer)).

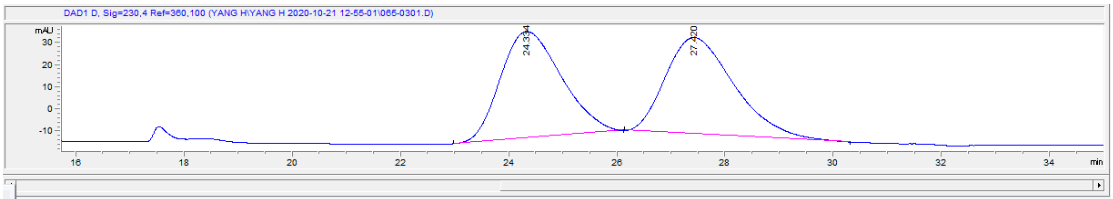

#	Time 时间	Area 峰面积	Height 峰高	Width 峰宽	Symmetry factor 对称因子	Area% 峰面积 %	Area%
1	24.334	3772.3	48.2	1.1196	0.744	49.428	49.428
2	27.42	3859.6	43.6	1.1604	0.671	50.572	50.572

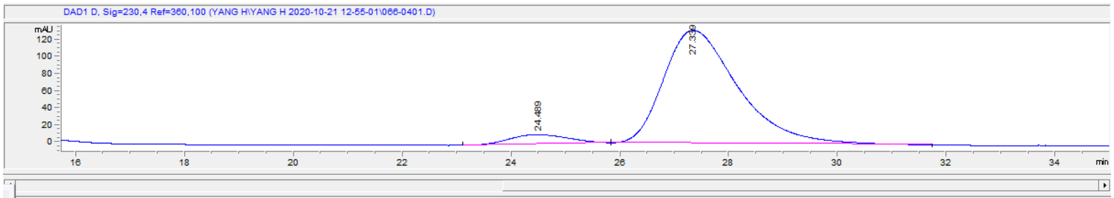

#	Time 时间	Area 峰面积	Height 峰高	Width 峰宽	Symmetry factor 对称因子	Area% 峰面积 %	Area%
1	24.489	816.6	11	0.8798	0.936	6.104	6.104
2	27.339	12561	132.3	1.388	0.619	93.896	93.896

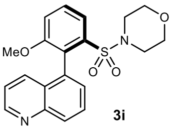

(*S*)-4-((3-methoxy-2-(quinolin-5-yl)phenyl)sulfonyl)morpholine (3i)

Compound **3i** was obtained from the coupling reaction between **S30** and quinolin-5-yl-5-boronic acid in 79% yield and 81% ee. $[\alpha]^{25}_D$ = -17.9 (*c* = 0.5, $CHCl_3$); ^1H NMR (500 MHz, $CDCl_3$) δ 8.90 (d, *J* = 2.4 Hz, 1H), 8.14 (d, *J* = 8.5 Hz, 1H), 7.76 (t, *J* = 8.0 Hz, 2H), 7.51-7.37 (m, 3H), 6.85 (d, *J* = 7.7 Hz, 1H), 3.68-3.59 (m, 7H), 2.85 (d, *J* = 3.9 Hz, 4H); ^{13}C NMR (151 MHz, $CDCl_3$) δ 157.8, 150.4, 148.5 142.1, 134.2, 133.8, 129.7, 129.4, 129.1, 128.8, 127.3, 122.6, 121.2,

116.6, 107.9, 68.0, 56.3, 55.9; HRMS (ESI) Calcd. for $C_{20}H_{21}N_2O_4S$ $[M+H]^+$: 385.1222; Found: 385.1229; Enantiomeric excess was determined by chiral HPLC (Chiralcel AD-3, 25 °C, flow rate: 0.8 mL/min, hexanes/isopropanol: 70/30, 254 nm, 7.40 min (minor isomer), 9.16 min (major isomer)).

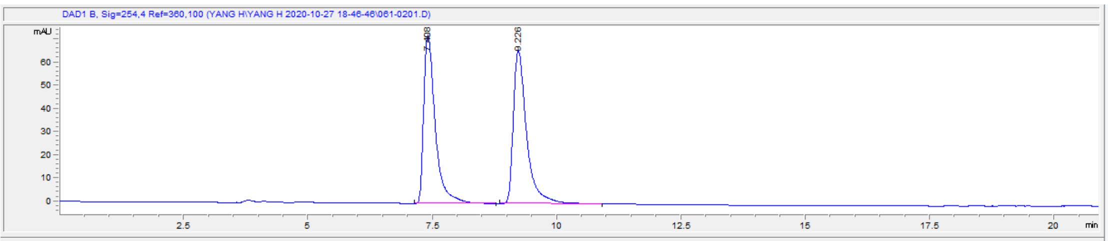

#	Time 时间	Area 峰面积	Height 峰高	Width 峰宽	Symmetry factor 对称因子	Area% 峰面积 %	Area%
1	7.408	1177.1	72.6	0.2451	0.526	48.993	48.993
2	9.226	1225.5	66.2	0.278	0.546	51.007	51.007

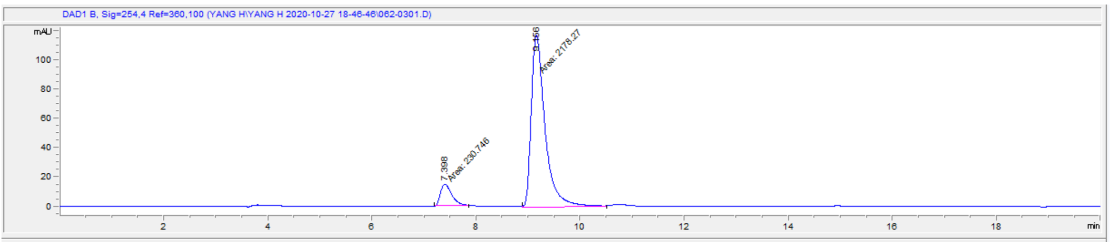

#	Time 时间	Area 峰面积	Height 峰高	Width 峰宽	Symmetry factor 对称因子	Area% 峰面积 %	Area%
1	7.398	230.7	15	0.2564	0.632	9.578	9.578
2	9.156	2178.3	117.2	0.3098	0.56	90.422	90.422

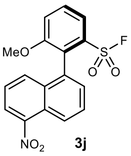

3j

(*S*)-3-methoxy-2-(5-nitronaphthalen-1-yl)benzenesulfonyl fluoride (3j) Compound **3j** was obtained from the coupling reaction between **S23** and 5-nitronaphthalene-1-boronic acid in 90% yield and 99% ee. $[\alpha]^{25}_D$ = -81.4 (c = 0.5, $CHCl_3$); ^{1}H NMR (600 MHz, $CDCl_3$) δ 8.62 (d, J = 8.8 Hz, 1H), 8.19 (dd, J = 7.5, 1.0 Hz, 1H), 7.89 (dd, J = 8.1, 0.6 Hz, 1H), 7.79 (dd, J = 8.8, 7.1 Hz, 1H), 7.71 (td, J = 8.3, 1.0 Hz, 1H), 7.55 (d, J = 8.4 Hz, 1H), 7.51 (d, J = 7.1 Hz, 1H), 7.46-7.40 (m, 2H), 3.69 (s, 3H); ^{13}C NMR (151 MHz, $CDCl_3$) δ 158.9, 147.4, 134.9 (d, J = 21.7 Hz), 133.4, 131.8, 131.8, 130.5, 129.6, 128.6, 125.2, 124.6, 124.1, 123.9, 121.9, 117.7, 56.6; ^{19}F NMR (376 MHz, $CDCl_3$) δ 65.6; HRMS (EI) Calcd. for $C_{17}H_{12}FNO_5S$ $[M]^+$: 361.0420; Found: 361.0424; Enantiomeric excess was determined by chiral HPLC (Chiralcel OD-3, 25 °C, flow rate: 0.8 mL/min, hexanes/isopropanol: 90/10, 210 nm, 17.00 min (minor isomer), 17.96 min (major isomer)).

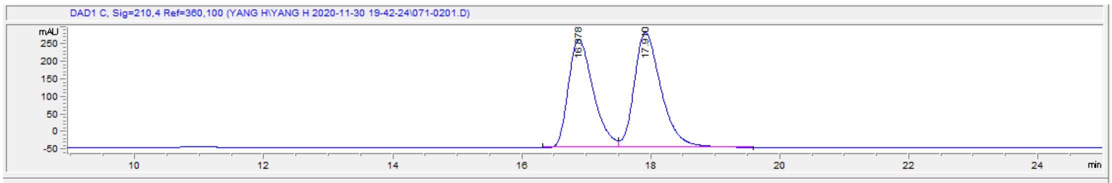

#	Time 时间	Area 峰面积	Height 峰高	Width 峰宽	Symmetry factor 对称因子	Area% 峰面积 %	Area%
1	16.878	8281.6	308.9	0.4148	0.676	46.378	46.378
2	17.91	9575.2	328.7	0.4478	0.658	53.622	53.622

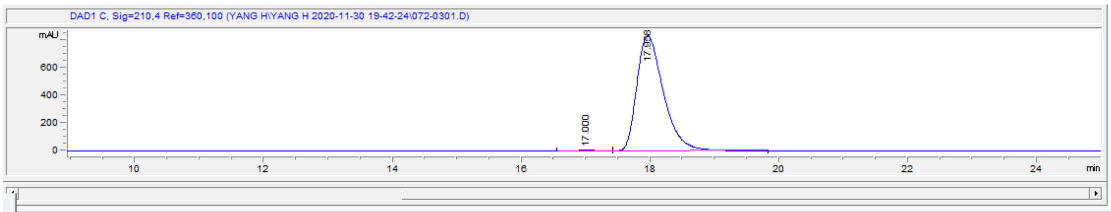

#	Time 时间	Area 峰面积	Height 峰高	Width 峰宽	Symmetry factor 对称因子	Area% 峰面积 %	Area%
1	17	135.5	5.6	0.3068	0.85	0.557	0.557
2	17.958	24202.1	838.1	0.4449	0.631	99.443	99.443

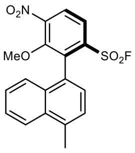

3k

(*S*)-3-methoxy-2-(4-methylnaphthalen-1-yl)-4-nitrobenzenesulfonyl fluoride (3k)

Compound **3k** was obtained from the coupling reaction between **S24** and 4-methylnaphthalene-1-boronic acid in 90% yield and 94% ee. $[\alpha]^{25}_{D}$ = -48.1 (c = 0.5, $CHCl_3$); ^{1}H NMR (500 MHz, $CDCl_3$) δ 8.08 (d, J = 8.6 Hz, 1H), 7.98 (d, J = 8.9 Hz, 1H), 7.58-7.51 (m, 1H), 7.47-7.38 (m, 2H), 7.34-7.28 (m, 2H), 7.28-7.26 (m, 4H), 3.74 (s, 3H), 2.77 (s, 3H); ^{13}C NMR (151 MHz, $CDCl_3$) δ 161.2, 143.5, 136.8, 133.4, 132.6, 131.7, 129.7 (d, J = 23.8 Hz), 128.5, 128.1, 126.4 (d, J = 13.4 Hz), 126.1 (d, J = 8.6 Hz), 125.7, 124.9, 119.6, 118.0, 114.9, 57.1 (d, J = 29.1 Hz), 19.9; ^{19}F NMR (376 MHz, $CDCl_3$) δ 67.8; HRMS (EI) Calcd. for $C_{18}H_{14}FNO_5S$ $[M]^+$: 375.0577; Found: 375.0571; Enantiomeric excess was determined by chiral HPLC (Chiralcel AD-3, 25 ºC, flow rate: 0.8 mL/min, hexanes/isopropanol: 70/30, 230 nm, 10.11 min (minor isomer), 14.22 min (major isomer)).

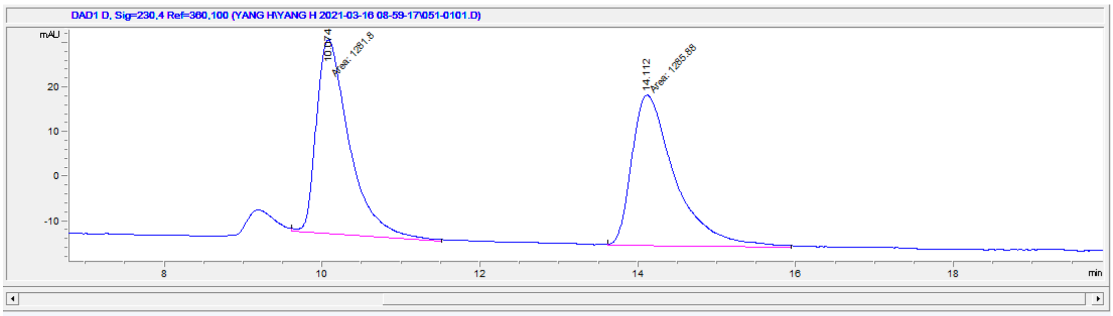

#	Time 时间	Area 峰面积	Height 峰高	Width 峰宽	Symmetry factor 对称因子	Area% 峰面积 %	Area%
1	10.074	1281.8	43.7	0.4891	0.545	49.921	100.000
2	14.112	1285.9	33.8	0.6348	0.524	50.079	0.000

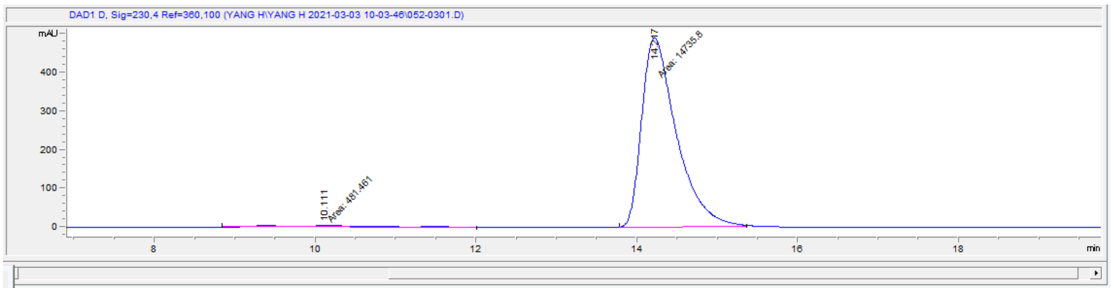

#	Time 时间	Area 峰面积	Height 峰高	Width 峰宽	Symmetry factor 对称因子	Area% 峰面积 %	Area%
1	10.111	481.5	5.3	1.5134	0.814	3.164	3.164
2	14.217	14735.8	489.2	0.5021	0.559	96.836	96.836

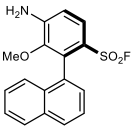

3l

(*S*)-4-amino-3-methoxy-2-(naphthalen-1-yl)benzenesulfonyl fluoride (3l)

Compound **3l** was obtained from the coupling reaction between **S25** and 1-naphthalene boronic acid in 92% yield and 97% ee. $[\alpha]^{25}_{D}$ = -98.1 (*c* = 0.5, $CHCl_3$); ^{1}H NMR (500 MHz, $CDCl_3$) δ 7.89 (t, *J* = 7.2 Hz, 2H), 7.53 (t, *J* = 7.7 Hz, 1H), 7.46 (ddd, *J* = 5.6, 5.1, 2.9 Hz, 1H), 7.37 (s, 2H), 7.32 (d, *J* = 6.9 Hz, 1H), 7.25 (d, *J* = 2.1 Hz, 2H), 6.92 (d, *J* = 9.1 Hz, 1H), 5.14 (s, 2H), 3.50 (s, 3H); ^{13}C NMR (151 MHz, $CDCl_3$) δ 150.0, 142.7, 133.3, 132.8, 132.6, 130.5, 128.6, 128.4, 127.5, 126.2, 125.8, 125.5, 1251, 122.6, 119.2, 115.2 (d, *J* = 20.1 Hz), 57.8; ^{19}F NMR (376 MHz, $CDCl_3$) δ 76.8; HRMS (EI) Calcd. for $C_{17}H_{14}FNO_3S$ $[M]^+$: 331.0678; Found: 331.0670; Enantiomeric excess was determined by chiral HPLC (Chiralcel AD-3, 25 ºC, flow rate: 0.8 mL/min, hexanes/isopropanol: 80/20, 290 nm, 18.13 min (minor isomer), 20.08 min (major isomer).

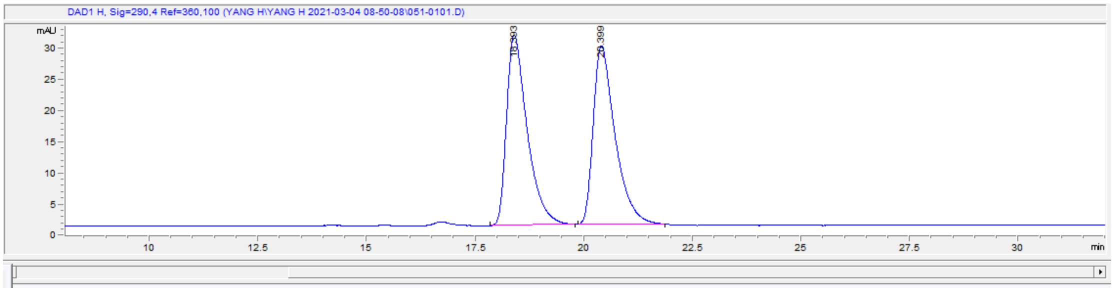

#	Time 时间	Area 峰面积	Height 峰高	Width 峰宽	Symmetry factor 对称因子	Area% 峰面积 %	Area%
1	18.393	1006.8	30.5	0.4837	0.549	50.153	50.153
2	20.399	1000.7	28.6	0.5179	0.554	49.847	49.847

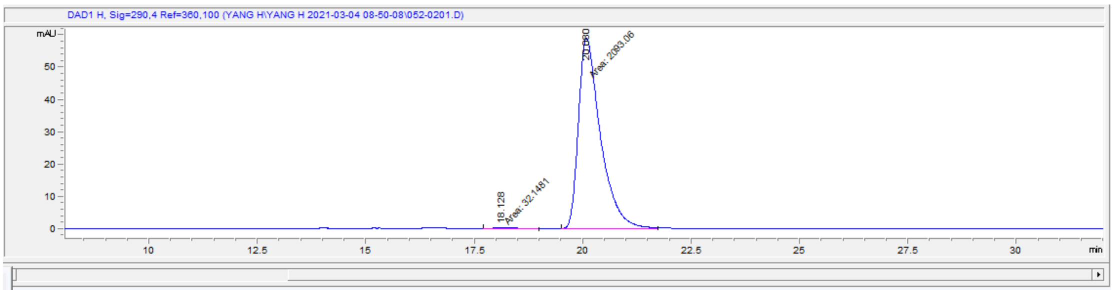

#	Time 时间	Area 峰面积	Height 峰高	Width 峰宽	Symmetry factor 对称因子	Area% 峰面积 %	Area%
1	18.128	32.1	6.4E-1	0.8361	0.486	1.513	1.513
2	20.08	2093.1	59.1	0.5904	0.552	98.487	98.487

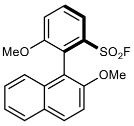

3m

(*R*)-3-methoxy-2-(2-methoxynaphthalen-1-yl)benzenesulfonyl fluoride (3m)

Compound **3m** was obtained from the coupling reaction (at 60 ºC) between **S23** and 2-methoxy-1-naphthaleneboronic acid in 77% yield and 98% ee. $[\alpha]^{25}_{D}$ = -49.7 (*c* = 0.5, $CHCl_3$); ^{1}H NMR (400 MHz, $CDCl_3$) δ 7.96 (d, *J* = 9.1 Hz, 1H), 7.87 (d, *J* = 8.1 Hz, 1H), 7.85-7.79 (m, 1H), 7.65 (t, *J* = 8.2 Hz, 1H), 7.39 (dd, *J* = 12.7, 8.7 Hz, 2H), 7.35-7.29 (m, 2H), 7.08-7.00 (m, 1H), 3.85 (s, 3H), 3.69 (s, 3H); ^{13}C NMR (151 MHz, $CDCl_3$) δ 159.2, 154.8, 135.2 (d, *J* = 21.8 Hz), 133.6, 130.9, 129.7, 128.2 126.8, 124.2, 123.7, 122.2, 117.8, 115.8, 113.2, 56.8, 56.6; ^{19}F NMR (376 MHz, $CDCl_3$) δ 61.9; HRMS (EI) Calcd. for $C_{18}H_{15}FO_4S$ $[M]^+$: 346.0675; Found: 346.0669; Enantiomeric excess was determined by chiral HPLC (Chiralcel OJ-3, 25 ºC, flow rate: 0.8 mL/min, hexanes/isopropanol: 80/20, 230 nm, 24.98 min (major isomer),

32.21 min (minor isomer).

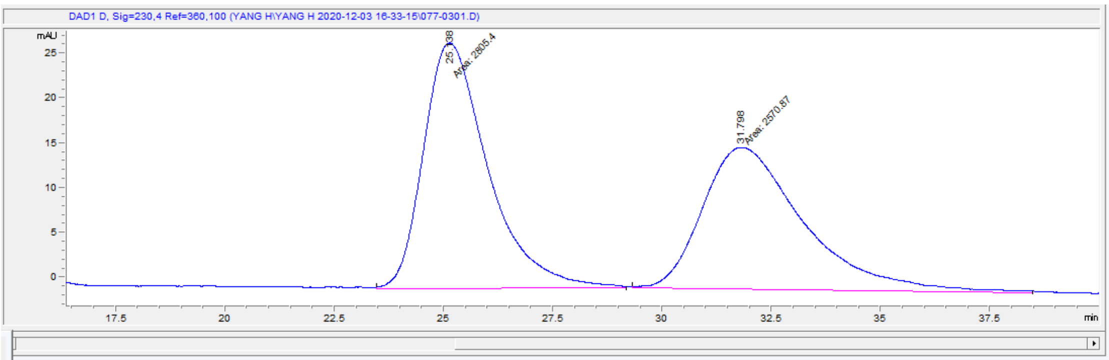

#	Time 时间	Area 峰面积	Height 峰高	Width 峰宽	Symmetry factor 对称因子	Area% 峰面积 %	Area%
1	25.138	2805.4	27.4	1.7076	0.665	52.181	52.181
2	31.798	2570.9	15.8	2.7067	0.589	47.819	47.819

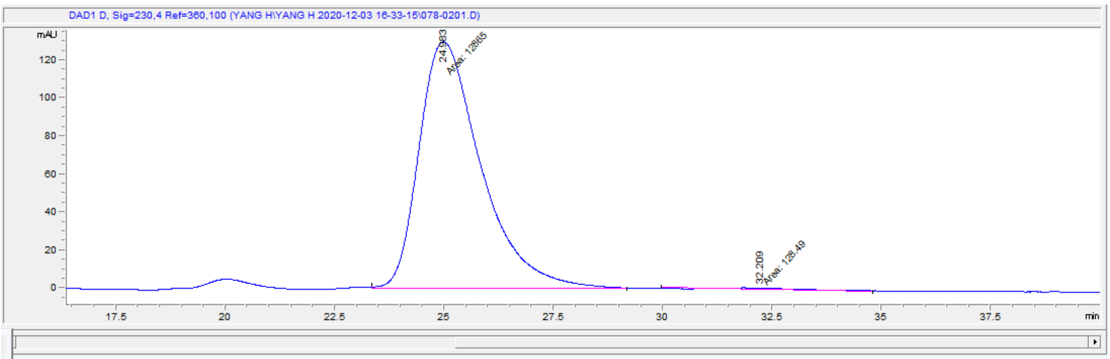

#	Time 时间	Area 峰面积	Height 峰高	Width 峰宽	Symmetry factor 对称因子	Area% 峰面积 %	Area%
1	24.983	12865	129.9	1.651	0.617	99.011	99.011
2	32.209	128.5	9.3E-1	2.2996	0.364	0.989	0.989

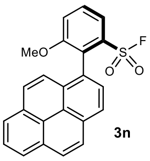

3n

(S)-3-methoxy-2-(pyren-1-yl)benzenesulfonyl fluoride (3n)

Compound **3n** was obtained from the coupling reaction between **S23** and 1-pyrenylboronic acid in 81% yield and 96% ee. $[\alpha]^{25}_{D}$ = -102.9 (c = 0.5, $CHCl_3$); ^{1}H NMR (500 MHz, $CDCl_3$) δ 8.26 (d, J = 7.8 Hz, 1H), 8.22 (dd, J = 7.6, 0.9 Hz, 1H), 8.17 (d, J = 7.4 Hz, 1H), 8.03-7.96 (m, 2H), 7.93 (dd, J = 8.1, 0.8 Hz, 1H), 7.89 (dd, J = 7.8, 1.4 Hz, 1H), 7.71 (td, J = 8.3, 1.3 Hz, 1H), 7.49 (d, J = 9.1 Hz, 1H), 7.46-7.42 (m, 1H), 3.66 (s, 3H); ^{13}C NMR (151 MHz, $CDCl_3$) δ 159.3, 135.2, 135.1, 131.8, 131.5, 131.0, 130.5, 130.1, 130.0, 128.0, 127.9, 127.9, 127.7, 126.2, 125.6, 125.5, 124.8, 124.8, 124.7, 124.3, 121.8, 117.7, 56.6; ^{19}F NMR (376 MHz, $CDCl_3$) δ 64.7; HRMS (ESI) Calcd. for $C_{23}H_{16}FO_3S$ $[M+H]^+$: 391.0804; Found: 391.0811; Enantiomeric excess was determined by chiral HPLC (Chiralcel AD-3, 25 °C, flow rate: 0.7 mL/min, hexanes/isopropanol: 93/7, 254 nm, 14.47 min (minor isomer), 15.41 min (major isomer).

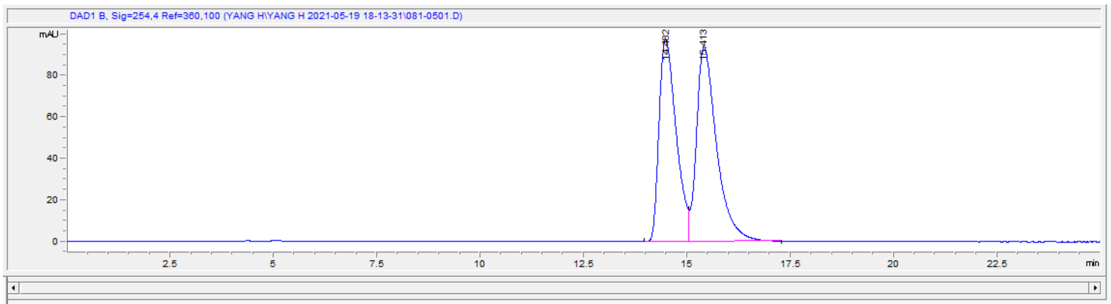

#	Time 时间	Area 峰面积	Height 峰高	Width 峰宽	Symmetry factor 对称因子	Area% 峰面积 %	Area%
1	14.482	2816.7	97.5	0.4431	0.587	46.897	46.897
2	15.413	3189.4	93.9	0.5105	0.551	53.103	53.103

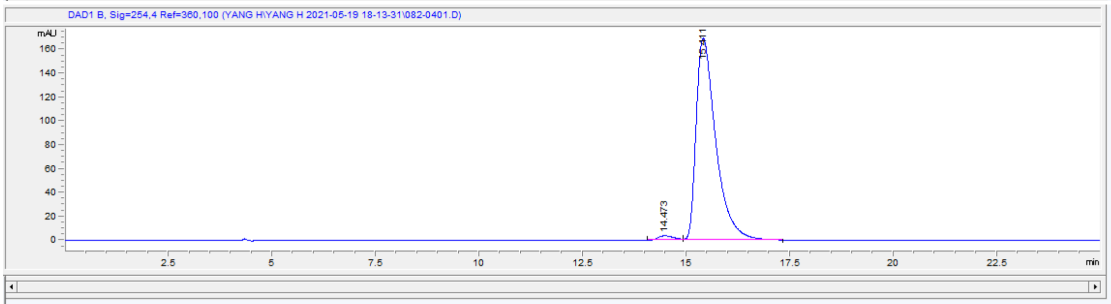

#	Time 时间	Area 峰面积	Height 峰高	Width 峰宽	Symmetry factor 对称因子	Area% 峰面积 %	Area%
1	14.473	102.6	3.7	0.3495	0.624	1.807	1.807
2	15.411	5574.1	169.3	0.4961	0.524	98.193	98.193

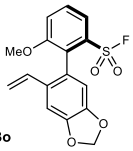

3o

(*S*)-3-methoxy-2-(6-vinylbenzo[d][1,3]dioxol-5-yl)benzenesulfonyl fluoride (3o)

Compound **3o** was obtained from the coupling reaction between **S23** and **S18** in 87% yield and 85% ee. $[\alpha]^{25}_{D}$ = -8.6 (c = 0.5, $CHCl_3$); ^1H NMR (500 MHz, $CDCl_3$) δ 7.78 (d, J = 8.1 Hz, 1H), 7.57 (t, J = 8.2 Hz, 1H), 7.31 (d, J = 8.4 Hz, 1H), 7.14 (s, 1H), 6.60 (d, J = 1.0 Hz, 1H), 6.08 (dd, J = 17.3, 10.9 Hz, 1H), 6.02 (dd, J = 3.9, 1.4 Hz, 2H), 5.51 (d, J = 17.3 Hz, 1H), 4.99 (d, J = 11.0 Hz, 1H), 3.77 (s, 3H); ^{13}C NMR (151 MHz, $CDCl_3$) δ 158.8, 148.6, 147.2, 134.6 (d, J = 21.1 Hz), 133.9, 131.5, 130.1, 129.7, 125.0, 121.7, 117.7, 113.5, 110.4, 104.7, 101.6, 56.8; ^{19}F NMR (376 MHz, $CDCl_3$) δ 64.8; HRMS (EI) Calcd. for $C_{16}H_{13}FO_5S$ $[M]^+$: 336.0468; Found: 336.0471; Enantiomeric excess was determined by chiral HPLC (Chiralcel IC-3, 25 °C, flow rate: 0.8 mL/min, hexanes/isopropanol: 90/10, 210 nm, 17.00 min (minor isomer), 17.96 min (major isomer)).

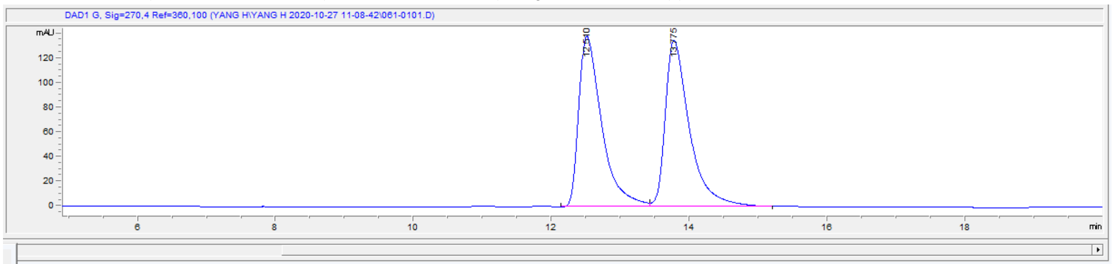

#	Time 时间	Area 峰面积	Height 峰高	Width 峰宽	Symmetry factor 对称因子	Area% 峰面积 %	Area%
1	12.51	3201.4	138.3	0.3501	0.489	49.205	49.205
2	13.775	3304.9	134.8	0.366	0.521	50.795	50.795

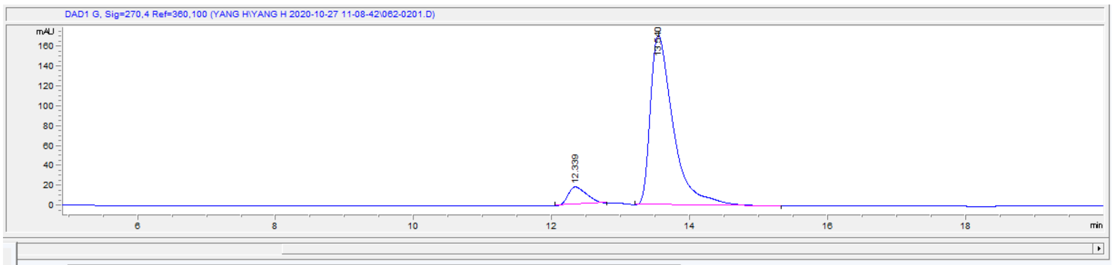

#	Time 时间	Area 峰面积	Height 峰高	Width 峰宽	Symmetry factor 对称因子	Area% 峰面积 %	Area%
1	12.339	334.2	17.9	0.2875	0.621	7.898	7.898
2	13.54	3897.1	170.6	0.3426	0.483	92.102	92.102

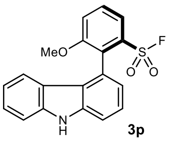

3p

(*S*)-2-(9H-carbazol-4-yl)-3-methoxybenzenesulfonyl fluoride (3p)
Compound **3p** was obtained from the coupling reaction between **S23** and 4-(4,4,5,5-tetramethyl-1,3,2-dioxaborolan-2-yl)-9H-carbazole in 83% yield and 97% ee. $[\alpha]^{25}_{D}$ = -82.7 (c = 0.5, $CHCl_3$); ^1H NMR (500 MHz, $CDCl_3$) δ 8.13 (s, 1H), 7.92 (d, J = 8.2 Hz, 1H), 7.72 (t, J = 8.2 Hz, 1H), 7.49 (dd, J = 7.3, 4.4 Hz, 2H), 7.44 (d, J = 8.4 Hz, 1H), 7.39 (d, J = 8.1 Hz, 1H), 7.31 (t, J = 7.7 Hz, 1H), 7.11 (dd, J = 7.4, 4.1 Hz, 1H), 6.91 (t, J = 7.5 Hz, 1H), 6.69 (d, J = 7.9 Hz, 1H), 3.65 (s, 4H); ^{13}C NMR (151 MHz, $CDCl_3$) δ 159.06 (s), 139.77 (s), 139.35 (s), 130.36 (d, J = 56.6 Hz), 129.85 (s), 127.21 (s), 125.83 (s), 125.34 (s), 123.10 (s), 121.91 (s), 121.62 (s), 120.96 (s), 120.54 (s), 119.50 (s), 117.91 (s), 117.80 (s), 111.12 (s), 110.75 (s), 56.81 (s); ^{19}F NMR (376 MHz, $CDCl_3$) δ 64.2; HRMS (ESI) Calcd. for $C_{19}H_{15}FNO_3S$ $[M+H]^+$: 356.0757; Found: 356.0758; Enantiomeric excess was determined by chiral HPLC (Chiralcel OD-3, 25 °C, flow rate: 0.5 mL/min, hexanes/isopropanol: 80/20, 260 nm, 15.57 min (minor isomer), 17.80 min (major isomer)).

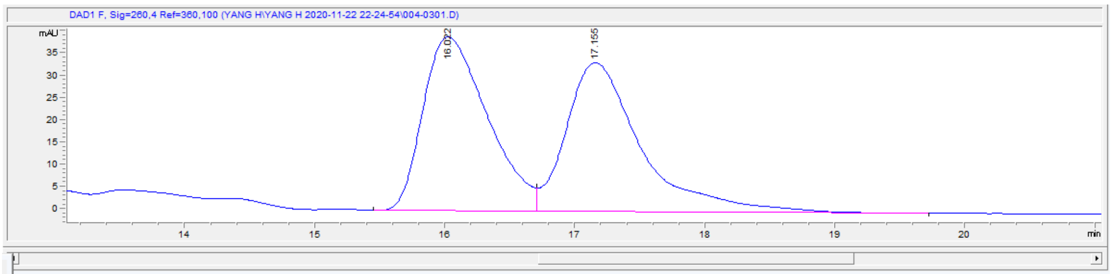

#	Time 时间	Area 峰面积	Height 峰高	Width 峰宽	Symmetry factor 对称因子	Area% 峰面积 %	Area%
1	16.022	1348.4	39	0.523	0.6	50.160	50.160
2	17.155	1339.8	33.4	0.596	0.579	49.840	49.840

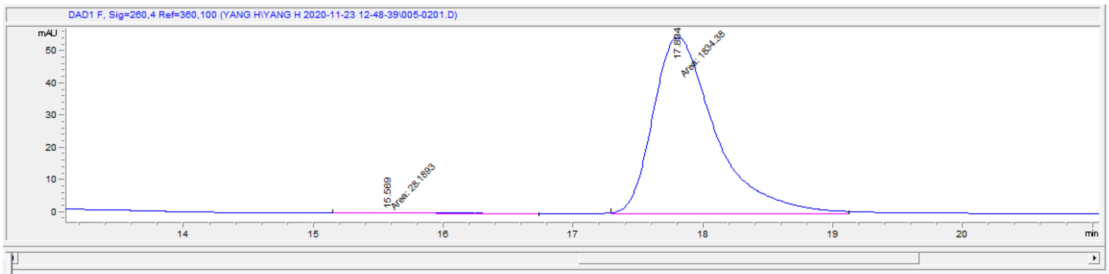

#	Time 时间	Area 峰面积	Height 峰高	Width 峰宽	Symmetry factor 对称因子	Area% 峰面积 %	Area%
1	15.569	28.2	4.2E-1	1.1275	0.342	1.513	1.513
2	17.804	1834.4	55	0.5562	0.629	98.487	98.487

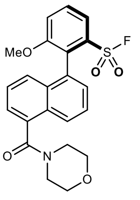

3q

(*S*)-3-methoxy-2-(5-(morpholine-4-carbonyl)naphthalen-1-yl)benzenesulfonyl fluoride (3q)

Compound **3q** was obtained from the coupling reaction between **S23** and **S10** in 80% yield and 98% ee. $[\alpha]^{25}_{D}$ = -52.4 (*c* = 0.5, $CHCl_3$); ^{1}H NMR (400 MHz, $CDCl_3$) δ 7.93 (t, *J* = 10.0 Hz, 1H), 7.87 (dd, *J* = 7.7, 3.9 Hz, 1H), 7.68 (t, *J* = 8.2 Hz, 1H), 7.61 (dd, *J* = 14.2, 6.9 Hz, 1H), 7.45-7.34 (m, 4H), 7.29 (s, 6H), 3.98 (t, *J* = 4.3 Hz, 2H), 3.87 (dd, *J* = 10.0, 7.1 Hz, 2H), 3.70 (s, 1H), 3.65 (s, 2H), 3.58 (t, *J* = 4.7 Hz, 2H), 3.29-3.21 (m, 2H); ^{13}C NMR (151 MHz, $CDCl_3$) (major rotamer) δ 207.1, 169.8, 159.1, 134.8 (d, *J* = 21.2 Hz), 134.1, 132.7, 131.7, 130.1, 129.6, 128.7, 126.9, 126.4, 125.6, 125.6, 124.0, 121.7, 117.6, 67.3, 56.5, 47.8, 42.4, 31.1; ^{19}F NMR (376 MHz, $CDCl_3$) δ 65.6 (major rotamer), 64.9 (minor rotamer); HRMS (ESI) Calcd. for $C_{22}H_{21}FNO_5S$ $[M+H]^+$: 430.1124; Found: 430.1126; Enantiomeric excess was determined by converting **3q** to **S31** upon treatment with *n*BuLi.

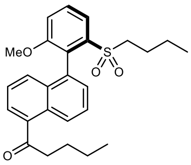

S31

(*S*)-1-(5-(2-(butylsulfonyl)-6-methoxyphenyl)naphthalen-1-yl)pentan-1-one (S31)

$[\alpha]^{25}_{D}$ = -106.5 (*c* = 0.5, $CHCl_3$); ^{1}H NMR (400 MHz, $CDCl_3$) δ 8.58 (d, *J* = 8.6 Hz, 1H), 7.91 (d, *J* = 7.9 Hz, 1H), 7.80 (d, *J* = 6.5 Hz, 1H), 7.68-7.60 (m, 2H), 7.51 (d, *J* = 7.0 Hz, 1H), 7.43 (d, *J* = 8.3 Hz, 1H), 7.40-7.35 (m, 1H), 7.30 (d, *J* = 8.4 Hz, 2H), 3.64 (s, 3H), 3.07 (t, *J* = 7.5 Hz, 2H), 2.62-2.44 (m, 1H), 2.37-2.20 (m, 1H), 1.89-1.67 (m, 2H), 1.45 (dt, *J* = 14.6, 7.3 Hz, 3H), 1.37-1.23 (m, 2H), 0.98 (t, *J* = 7.3 Hz, 4H), 0.64 (t, *J* = 7.3 Hz, 3H); ^{13}C NMR (151 MHz, $CDCl_3$) δ 205.5, 158.7, 140.1, 137.4, 133.2, 131.7, 130.1, 129.7, 128.4, 126.9, 126.9, 126.7, 124.7, 121.9, 116.0, 56.5, 54.8, 42.4, 26.9, 24.7, 22.7, 21.3, 14.1, 13.4; HRMS (ESI) Calcd. for $C_{26}H_{31}O_4S$ $[M+H]^+$: 439.1943; Found: 439.1947; Enantiomeric excess was determined by chiral HPLC (Chiralcel AD-3, 25 °C, flow rate: 0.7 mL/min, hexanes/isopropanol: 80/20, 250 nm, 17.90 min (minor isomer), 21.51 min (major isomer).

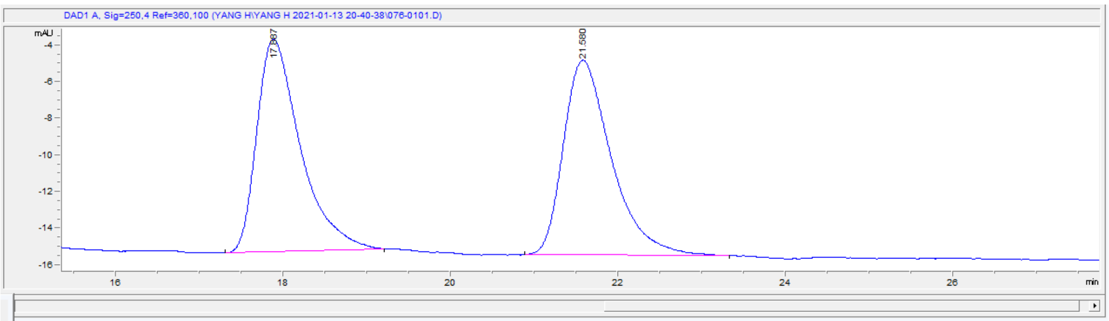

#	Time 时间	Area 峰面积	Height 峰高	Width 峰宽	Symmetry factor 对称因子	Area% 峰面积 %	Area%
1	17.887	420.1	11.6	0.5162	0.586	49.727	49.727
2	21.58	424.8	10.6	0.5779	0.639	50.273	50.273

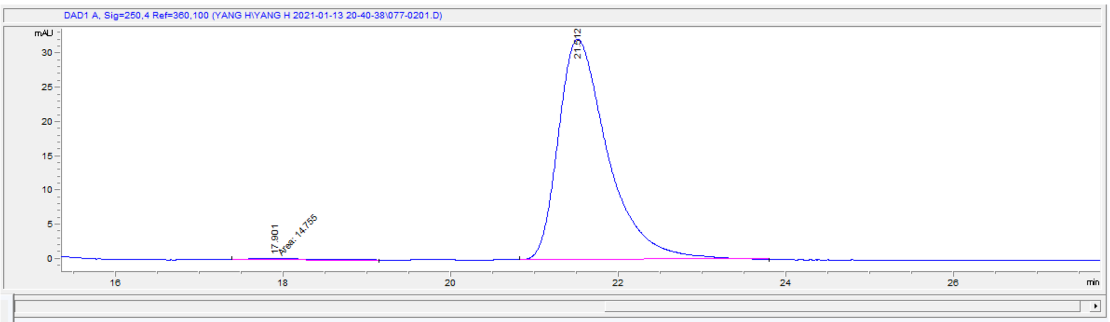

#	Time 时间	Area 峰面积	Height 峰高	Width 峰宽	Symmetry factor 对称因子	Area% 峰面积 %	Area%
1	17.901	14.8	2.5E-1	0.9769	0.339	1.105	1.105
2	21.512	1320.2	32.2	0.6147	0.593	98.895	98.895

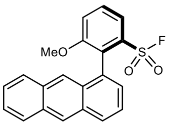

3r

(*S*)- 2-(anthracen-1-yl)-3-methoxybenzenesulfonyl fluoride (3r) Compound **3r** was obtained from the coupling reaction between **S23** and anthracene-1-yl boronic acid in 92% yield and 92% ee. $[\alpha]^{25}_{D}$ = -70.4 (*c* = 0.5, $CHCl_3$); ^{1}H NMR (500 MHz, $CDCl_3$) δ 8.50 (s, 1H), 8.11 (d, *J* = 8.5 Hz, 1H), 8.02 (d, *J* = 8.4 Hz, 1H), 7.93 (d, *J* = 8.1 Hz, 1H), 7.8-7.76 (m, 2H), 7.70 (t, *J* = 8.2 Hz, 1H), 7.60-7.51 (m, 1H), 7.43 (dt, *J* = 19.1, 8.7 Hz, 4H), 3.64 (s, 3H); ^{13}C NMR (151 MHz, $CDCl_3$) δ 159.2, 135.2, 135.0, 131.9, 131.7, 131.6, 131.1, 130.7, 130.0, 129.9, 129.6, 128.5, 128.2, 127.6, 127.0, 125.6, 125.5, 124.6, 124.0, 121.8, 117.7, 56.6; ^{19}F NMR (376 MHz, $CDCl_3$) δ 66.4; HRMS (ESI) Calcd. for $C_{21}H_{16}FO_3S$ $[M+H]^+$: 367.0804; Found: 367.0806; Enantiomeric excess was determined by chiral HPLC (Chiralcel AD-3, 25 °C, flow rate: 0.7 mL/min, hexanes/isopropanol: 95/5, 230 nm, 16.08 min (major isomer), 17.18 min (minor isomer).

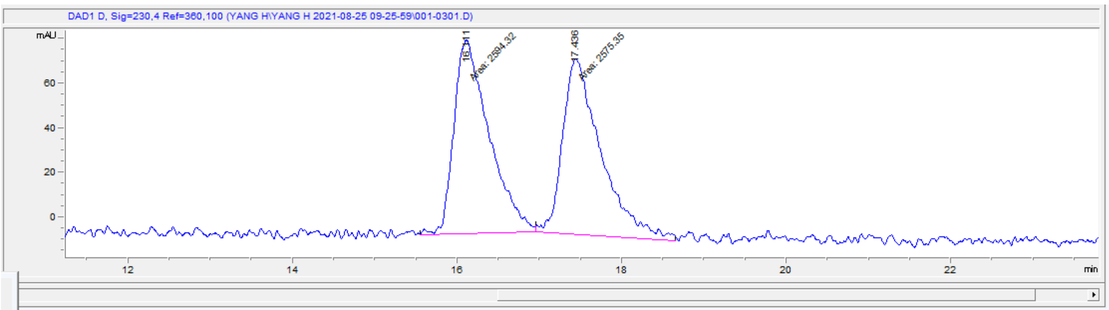

#	Time 时间	Area 峰面积	Height 峰高	Width 峰宽	Symmetry factor 对称因子	Area% 峰面积 %	Area%
1	16.111	2594.3	86.8	0.4982	0.541	50.184	50.184
2	17.436	2575.3	78.9	0.5437	0.498	49.816	49.816

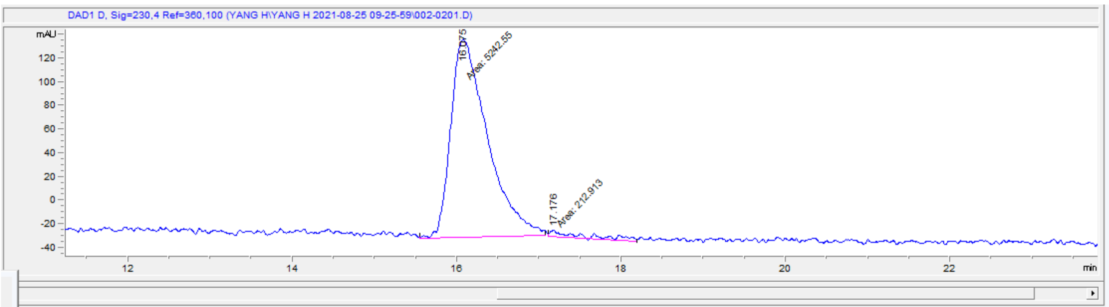

#	Time 时间	Area 峰面积	Height 峰高	Width 峰宽	Symmetry factor 对称因子	Area% 峰面积 %	Area%
1	16.075	5242.5	167.5	0.5216	0.494	96.097	96.097
2	17.176	212.9	6.2	0.5719	9.44E-2	3.903	3.903

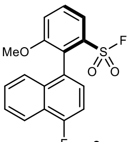

3s

(*S*)-2-(4-fluoronaphthalen-1-yl)-3-methoxybenzenesulfonyl fluoride (3s)

Compound **3s** was obtained from the coupling reaction between **S23** and 4-fluoronaphthalene-1-boronic acid in 88% yield and 96% ee. $[\alpha]^{25}_{D}$ = -61.7 (*c* = 0.5, $CHCl_3$); ^{1}H NMR (400 MHz, $CDCl_3$) δ 8.17 (d, *J* = 8.4 Hz, 1H), 7.87 (d, *J* = 7.8 Hz, 1H), 7.67 (td, *J* = 8.3, 1.3 Hz, 1H), 7.54 (t, *J* = 7.3 Hz, 1H), 7.46-7.41 (m, 1H), 7.39 (d, *J* = 8.4 Hz, 1H), 7.31 (ddd, *J* = 7.1, 5.4, 1.5 Hz, 1H), 7.23 (dd, *J* = 10.0, 7.9 Hz, 1H), 3.68 (s, 1H); ^{13}C NMR (151 MHz, $CDCl_3$) δ 159.3 (d, *J* = 253.6 Hz), 159.2, 135.2 (d, *J* = 21.2 Hz), 133.9 (d, *J* = 4.9 Hz), 130.1, 129.2, 127.9 (d, *J* = 8.6 Hz), 127.3, 126.7 (d, *J* = 4.6 Hz), 126.3 (d, *J* = 1.7 Hz), 125.3 (d, *J* = 2.6 Hz), 123.7 (d, *J* = 16.6 Hz), 121.8, 121.0 (d, *J* = 5.4 Hz), 117.6, 109.0 (d, *J* = 20.4 Hz), 56.6; ^{19}F NMR (376 MHz, $CDCl_3$) δ 65.5, 57.5, -121.5.; HRMS (EI) Calcd. for $C_{17}H_{12}F_2O_3S$ $[M]^{+}$: 334.0475; Found: 334.0471; Enantiomeric excess was determined by chiral HPLC (Chiralcel AD-3, 25 °C, flow rate: 0.6 mL/min, hexanes/isopropanol: 99/1, 254 nm, 19.76 min (major isomer), 22.14 min (minor isomer)).

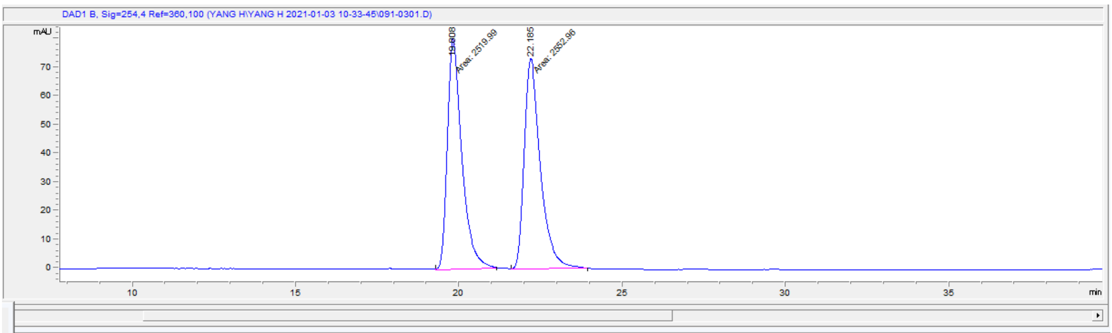

#	Time 时间	Area 峰面积	Height 峰高	Width 峰宽	Symmetry factor 对称因子	Area% 峰面积 %	Area%
1	19.808	2520	80.5	0.5215	0.626	49.675	49.675
2	22.185	2553	73.5	0.579	0.624	50.325	50.325

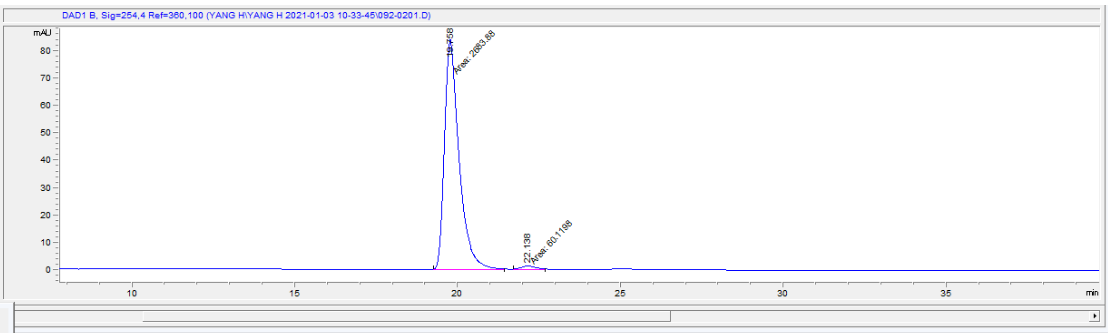

#	Time 时间	Area 峰面积	Height 峰高	Width 峰宽	Symmetry factor 对称因子	Area% 峰面积 %	Area%
1	19.758	2683.9	84.1	0.5317	0.61	97.809	97.809
2	22.138	60.1	1.6	0.6409	0.776	2.191	2.191

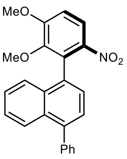

6a

(*R*)-1-(2,3-dimethoxy-6-nitrophenyl)-4-phenylnaphthalene (6a)

Compound **6a** was obtained from the coupling reaction between 2-bromo-3,4-dimethoxy-1-nitrobenzene and 4-phenylnaphthalene-1-boronic acid in 89% yield and 89% ee. $[\alpha]^{25}_{D}$ = -93.8 (*c* = 0.5, $CHCl_3$); ^{1}H NMR (500 MHz, $CDCl_3$) δ 7.98 (dd, *J* = 11.1, 8.5 Hz, 2H), 7.6-7.56 (m, 2H), 7.56-7.33 (m, 8H), 7.10 (d, *J* = 9.2 Hz, 1H), 4.03 (s, 3H), 3.50 (s, 3H); ^{13}C NMR (126 MHz, $CDCl_3$) δ 157.2, 147.8, 143.2, 140.8, 140.6, 132.5, 131.8, 131.2, 131.1, 130.4, 128.4, 127.4, 126.6, 126.3, 126.2, 126.0, 125.7, 125.5, 121.6, 110.9, 61.3, 56.4; HRMS

(ESI) Calcd. for $C_{24}H_{20}NO_4$ $[M+H]^+$: 386.1392; Found: 386.1397; Enantiomeric excess was determined by chiral HPLC (Chiralcel OD-3, 25 ºC, flow rate: 0.7 mL/min, hexanes/isopropanol: 80/20, 214 nm, 10.43 min (major isomer), 13.02 min (minor isomer)).

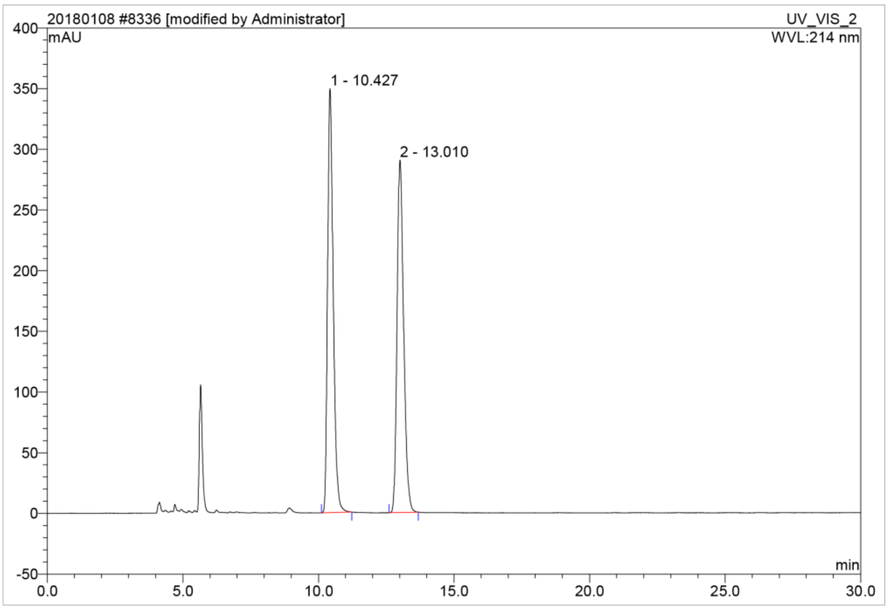

No.	Ret.Time min	Peak Name	Height mAU	Area mAU*min	Rel.Area %	Amount	Type
1	10.43	n.a.	349.575	84.329	50.65	n.a.	BMB
2	13.01	n.a.	290.615	82.150	49.35	n.a.	BMB
Total:			640.190	166.479	100.00	0.000	

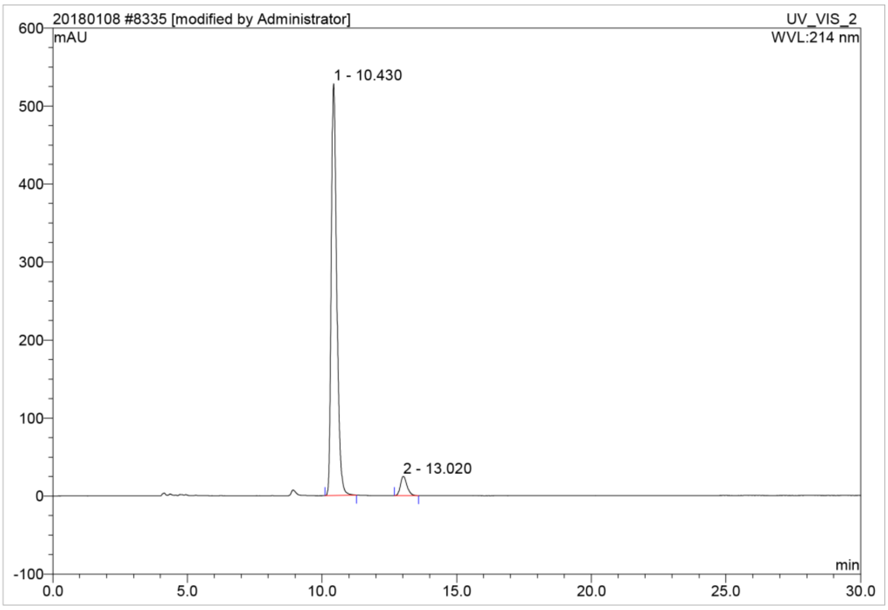

No.	Ret.Time min	Peak Name	Height mAU	Area mAU*min	Rel.Area %	Amount	Type
1	10.43	n.a.	528.087	125.459	94.73	n.a.	BMB
2	13.02	n.a.	24.633	6.976	5.27	n.a.	BMB
Total:			552.720	132.435	100.00	0.000	

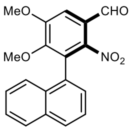

6b

(*R*)-4,5-dimethoxy-3-(naphthalen-1-yl)-2-nitrobenzaldehyde (6b)

Compound **6b** was obtained from the coupling reaction between 3-bromo-4,5-dimethoxy-2-nitrobenzaldehyde and 1-naphthalene boronic acid in 92% yield and 95% ee. $[\alpha]^{25}_D$ = -103.2 (c = 0.5, $CHCl_3$); ^1H NMR (500 MHz, $CDCl_3$) δ 9.95 (s, 1H), 7.92 (dd, J = 13.9, 8.2 Hz, 2H), 7.61 (s, 1H), 7.51 (ddd, J = 9.2, 6.0, 1.7 Hz, 2H), 7.47-7.42 (m, 2H), 7.35 (dd, J = 7.0, 1.1 Hz, 1H), 4.06 (s, 3H), 3.58 (s, 3H); ^{13}C NMR (151 MHz, $CDCl_3$) δ 186.2, 154.5, 152.5, 147.3, 133.5, 132.0, 129.7, 129.0, 128.8, 128.6, 127.3, 126.8, 126.4, 125.3, 125.2, 123.7, 110.8, 61.5, 56.6; HRMS (ESI) Calcd. for $C_{19}H_{16}NO_5$ $[M+H]^+$: 338.1028; Found: 338.1031; Enantiomeric excess was determined by chiral HPLC (Chiralcel AD-3, 25 °C, flow rate: 0.6 mL/min, hexanes/isopropanol: 80/20, 250 nm, 9.89 min (minor isomer), 10.74 min (major isomer)).

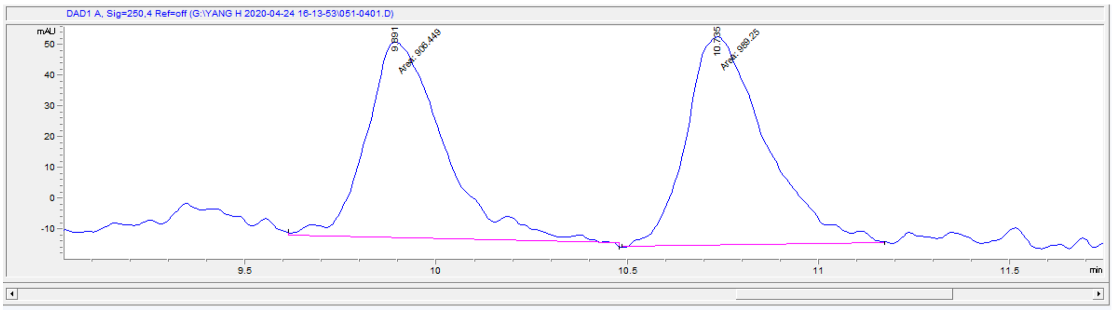

#	Time 时间	Area 峰面积	Height 峰高	Width 峰宽	Symmetry factor 对称因子	Area% 峰面积 %	Area%
1	9.891	906.4	63.8	0.2369	0.581	47.816	47.816
2	10.735	989.3	68	0.2425	0.631	52.184	52.184

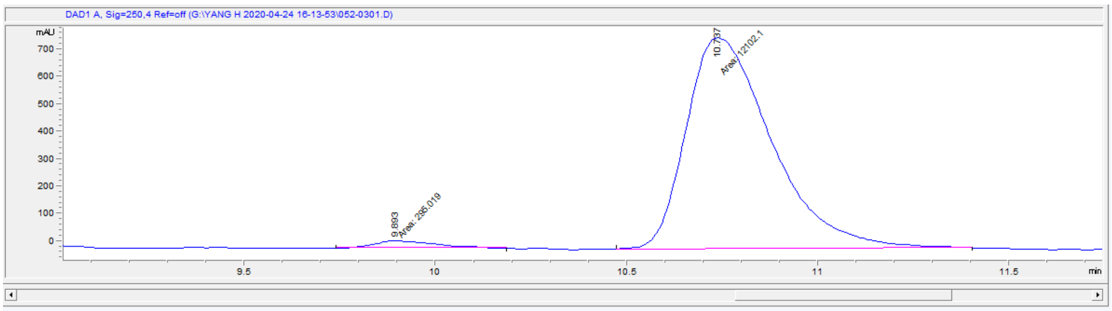

#	Time 时间	Area 峰面积	Height 峰高	Width 峰宽	Symmetry factor 对称因子	Area% 峰面积 %	Area%
1	9.893	295	27.1	0.1816	0.514	2.380	2.380
2	10.737	12102.1	773	0.2609	0.566	97.620	97.620

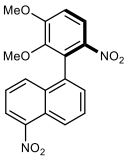

6c

(*R*)-1-(2,3-dimethoxy-6-nitrophenyl)-5-nitronaphthalene (6c)

Compound **6c** was obtained from the coupling reaction between 2-bromo-3,4-dimethoxy-1-nitrobenzene and 5-nitronaphthalene-1-boronic acid in 83% yield and 91% ee. $[\alpha]^{25}_D$ = -66.1 (c = 1.0, $CHCl_3$); ^1H NMR (500 MHz, $CDCl_3$) δ 8.58 (d, J = 8.8 Hz, 1H), 8.20 (dd, J = 7.6, 1.0 Hz, 1H), 8.04 (d, J = 9.2 Hz, 1H), 7.77-7.74 (m, 1H), 7.74-7.72 (m, 1H), 7.48-7.43 (m, 1H), 7.41 (dd, J = 7.0, 0.8 Hz, 1H), 7.13 (d, J = 9.2 Hz, 1H), 4.04 (s, 3H), 3.45 (s, 3H); ^{13}C NMR (126 MHz, $CDCl_3$) δ 157.4, 147.6, 147.4, 142.6, 133.2, 133.1 (d, J = 15.8 Hz), 133.0, 131.8, 130.1, 128.7, 127.4, 125.4, 124.7, 124.0, 123.4, 122.0, 111.4, 61.2, 56.5; HRMS (ESI) Calcd. for $C_{18}H_{15}N_2O_6$ $[M+H]^+$: 355.0930; Found: 355.0930; Enantiomeric excess was determined by chiral HPLC (Chiralcel OD-3, 25 °C, flow rate: 0.8 mL/min,

hexanes/isopropanol: 70/30, 290 nm, 11.12 min (major isomer), 13.66 min (minor isomer)).

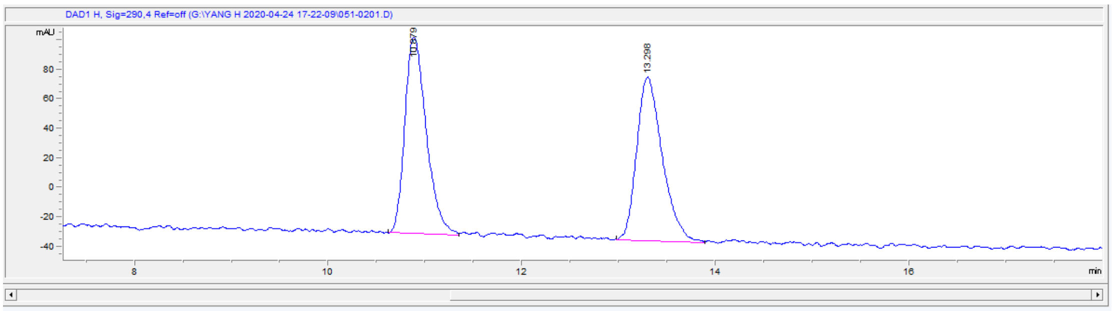

#	Time 时间	Area 峰面积	Height 峰高	Width 峰宽	Symmetry factor 对称因子	Area% 峰面积 %	Area%
1	10.879	2068.5	133.1	0.2274	0.663	50.116	50.116
2	13.298	2059	111.3	0.2599	0.715	49.884	49.884

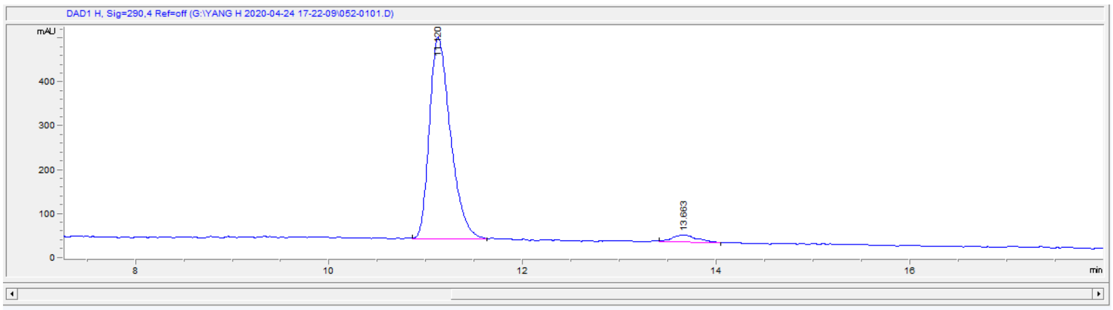

#	Time 时间	Area 峰面积	Height 峰高	Width 峰宽	Symmetry factor 对称因子	Area% 峰面积 %	Area%
1	11.12	7024	461.4	0.2296	0.635	95.667	95.667
2	13.663	318.1	17.5	0.2177	0.858	4.333	4.333

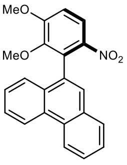

6d

(*R*)-9-(2,3-dimethoxy-6-nitrophenyl)phenanthrene (6d)

Compound **6d** was obtained from the coupling reaction between 2-bromo-3,4-dimethoxy-1-nitrobenzene and 9-phenanthreneboronic acid in 90% yield and 92% ee. $[\alpha]^{25}_{D}$ = -69.2 (c = 0.5, $CHCl_3$); ^{1}H NMR (500 MHz, $CDCl_3$) δ 8.76 (dd, J = 14.0, 8.3 Hz, 2H), 8.02 (dd, J = 9.1, 0.8 Hz, 1H), 7.85 (d, J = 7.9 Hz, 1H), 7.73-7.64 (m, 2H), 7.61 (t, J = 7.4 Hz, 1H), 7.57 (s, 1H), 7.54-7.46 (m, 2H), 7.09 (d, J = 9.1 Hz, 1H), 4.02 (s, 3H), 3.46 (s, 3H); ^{13}C NMR (126 MHz, $CDCl_3$) δ 157.3, 148.0, 143.2, 131.5, 131.4, 131.2, 130.8, 130.6, 130.4, 128.9, 127.0, 126.9, 126.8, 126.7, 126.7, 126.0, 123.1, 122.8, 121.7, 110.9, 61.3, 56.4; HRMS (ESI) Calcd. for $C_{22}H_{18}NO_4$ $[M+H]^+$: 360.1236; Found: 360.1239; Enantiomeric excess was determined by chiral HPLC (Chiralcel OD-3, 25 °C, flow rate: 0.7 mL/min, hexanes/isopropanol: 80/20, 214 nm, 21.10 min (minor isomer), 23.15 min (major isomer)).

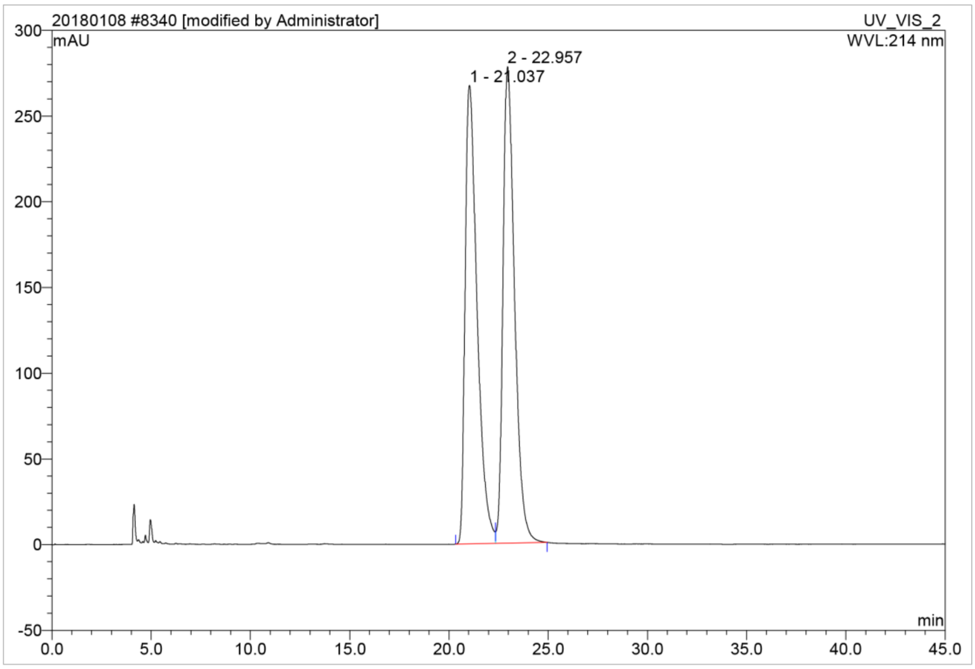

No.	Ret.Time min	Peak Name	Height mAU	Area mAU*min	Rel.Area %	Amount	Type
1	21.04	n.a.	267.527	187.025	49.78	n.a.	BM
2	22.96	n.a.	278.059	188.668	50.22	n.a.	MB
Total:			545.586	375.694	100.00	0.000	

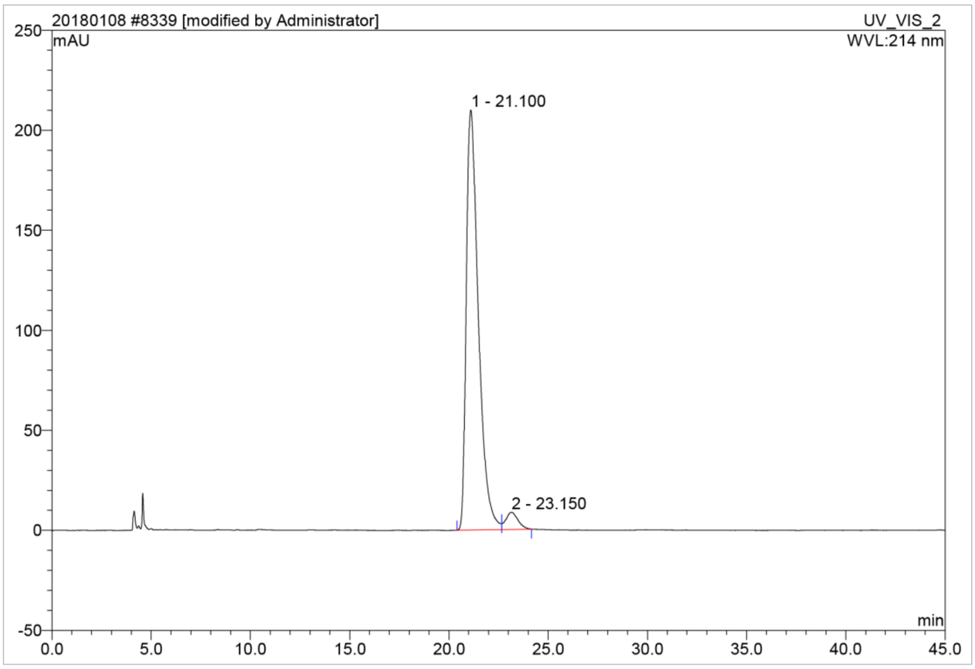

No.	Ret.Time min	Peak Name	Height mAU	Area mAU*min	Rel.Area %	Amount	Type
1	21.10	n.a.	210.033	148.435	96.03	n.a.	BM
2	23.15	n.a.	8.547	6.132	3.97	n.a.	MB
Total:			218.581	154.567	100.00	0.000	

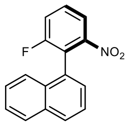

6e

(*R*)-1-(2-fluoro-6-nitrophenyl)naphthalene (6e)

Compound **6e** was obtained from the coupling reaction between 2-bromo-3-fluoronitrobenzene and 1-naphthalene boronic acid in 94% yield and 92% ee. $[\alpha]^{25}_{D}$ = -28.1 (c = 0.5, $CHCl_3$); ^{1}H NMR (500 MHz, $CDCl_3$) δ 7.95 (t, J = 9.2 Hz, 2H), 7.87 (d, J = 8.2 Hz, 1H), 7.62-7.43 (m, 7H), 7.38 (d, J = 7.0 Hz, 1H); ^{13}C NMR (126 MHz, $CDCl_3$) δ 160.5 (d, J = 250.3 Hz), 151.0 (d, J = 4.3 Hz), 133.6, 131.8, 129.9, 129.8, 128.7, 128.2 (d, J = 321.7 Hz), 128.2, 127.1, 126.3, 125.4, 124.7, 123.7 (d, J = 22.0 Hz), 120.3 (d, J = 23.4 Hz), 120.0 (d, J = 3.7 Hz); ^{19}F NMR (282 MHz, $CDCl_3$) δ -109.3; HRMS (ESI) Calcd. for $C_{16}H_{11}FNO_2$ $[M+H]^+$: 268.0774; Found: 268.0781; Enantiomeric excess was determined by chiral HPLC (Chiralcel OD-3, 25 °C, flow rate: 0.5 mL/min, hexanes/isopropanol: 99/1, 290 nm, 16.85 min (major isomer), 18.22 min (minor isomer)).

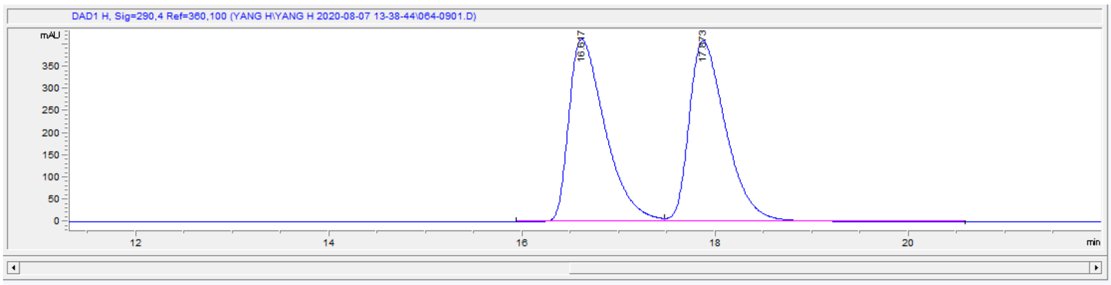

#	Time 时间	Area 峰面积	Height 峰高	Width 峰宽	Symmetry factor 对称因子	Area% 峰面积 %	Area%
1	16.617	10737.3	412.1	0.398	0.509	49.875	49.875
2	17.873	10791	408.1	0.4045	0.57	50.125	50.125

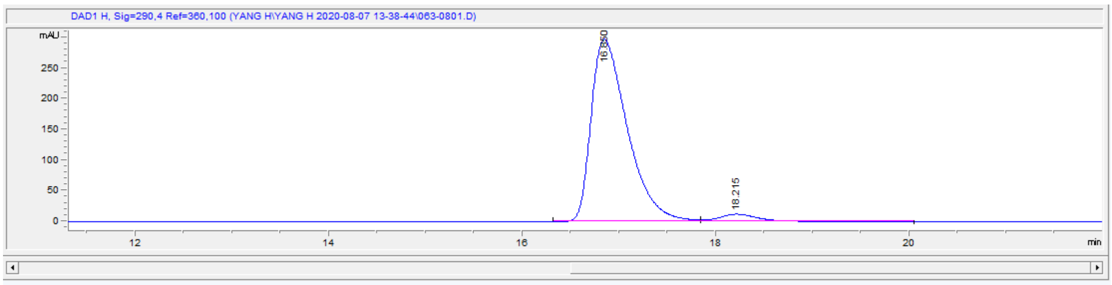

#	Time 时间	Area 峰面积	Height 峰高	Width 峰宽	Symmetry factor 对称因子	Area% 峰面积 %	Area%
1	16.85	7748.8	297.5	0.3958	0.529	95.750	95.750
2	18.215	343.9	12.1	0.4248	0.739	4.250	4.250

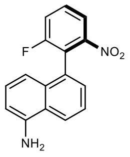

6f

(*R*)-5-(2-fluoro-6-nitrophenyl)naphthalen-1-amine (6f)

Compound **6f** was obtained from the coupling reaction between 2-bromo-1-fluoro-3-nitrobenzene and boronic acid **S21** in 91% yield and 87% ee. $[\alpha]^{25}_{D}$ = -6.2 (c = 0.3, $CHCl_3$); ^{1}H NMR (500 MHz, $CDCl_3$) δ 7.99 (d, J = 8.5 Hz, 1H), 7.87 (d, J = 8.2 Hz, 1H), 7.59 (td, J = 8.3, 5.4 Hz, 1H), 7.53 (dd, J = 8.5, 7.1 Hz, 1H), 7.47 (td, J = 8.3, 1.0 Hz, 1H), 7.36 (d, J = 7.0 Hz, 1H), 7.28-7.23 (m, 3H), 6.91 (d, J = 7.6 Hz, 2H); ^{13}C NMR (151 MHz, $CDCl_3$) δ 160.3 (d, J = 250.1 Hz), 150.7, 141.8, 132.5, 129.6 (d, J = 8.9 Hz), 128.6, 127.2, 127.1, 124.3, 124.0 (d, J = 22.1 Hz), 123.9, 122.2, 120.2 (d, J = 23.3 Hz), 119.9 (d, J = 3.5 Hz), 116.0, 110.8; ^{19}F NMR (376 MHz, $CDCl_3$) δ -108.6; HRMS (ESI) Calcd. for $C_{16}H_{11}N_2FNaO_2$ $[M+Na]^+$: 305.0702; Found: 305.0710; Enantiomeric excess was determined by chiral HPLC (Chiralcel IC, 25 °C, flow rate: 1.0 mL/min, hexanes/isopropanol: 70/30, 254 nm, 8.98 min (major isomer), 10.54 min (minor isomer)).

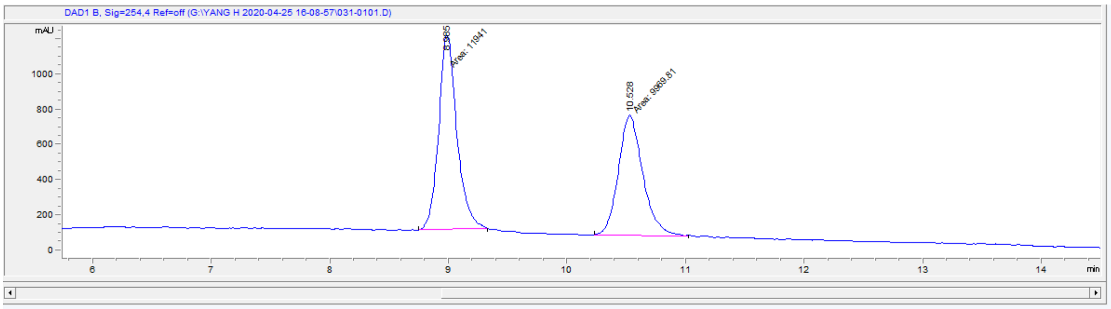

#	Time 时间	Area 峰面积	Height 峰高	Width 峰宽	Symmetry factor 对称因子	Area% 峰面积 %	Area%
1	8.985	11941	1102.1	0.1806	0.797	54.498	54.498
2	10.528	9969.8	687.1	0.2418	0.842	45.502	45.502

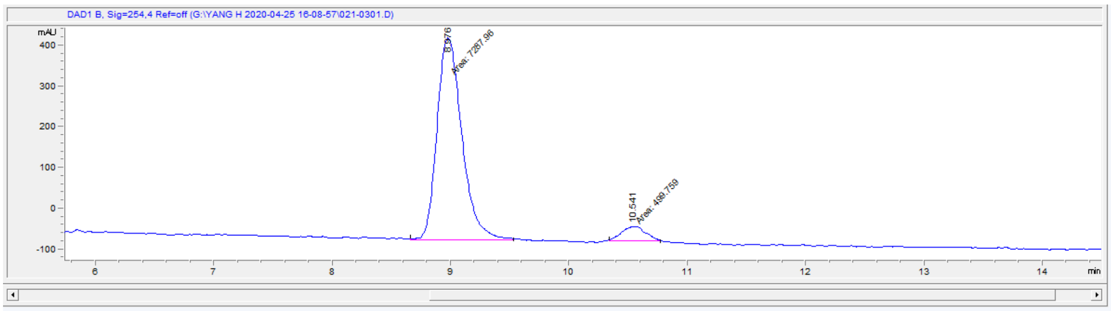

#	Time 时间	Area 峰面积	Height 峰高	Width 峰宽	Symmetry factor 对称因子	Area% 峰面积 %	Area%
1	8.976	7288	495.9	0.2449	0.748	93.583	93.583
2	10.541	499.8	35.4	0.2352	0.909	6.417	6.417

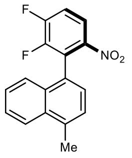

6g

(*R*)-1-(2,3-difluoro-6-nitrophenyl)-4-methylnaphthalene (6g)

Compound **6g** was obtained from the coupling reaction between 2-bromo-3,4-difluoro-1-nitrobenzene and 4-methylnaphthalene-1-boronic acid in 91% yield and 91% ee. $[\alpha]^{25}_{D}$ = -3.0 (c = 0.8, $CHCl_3$); ^1H NMR (500 MHz, $CDCl_3$) δ 8.11 (d, J = 8.4 Hz, 1H), 7.98-7.88 (m, 1H), 7.59 (dd, J = 8.2, 6.7 Hz, 1H), 7.53-7.34 (m, 4H), 7.30-7.26 (m, 1H), 2.78 (s, 3H); ^{13}C NMR (126 MHz, $CDCl_3$) δ 153.3 (dd, J = 259.4, 14.0 Hz), 148.6 (dd, J = 251.4, 13.2 Hz), 146.0, 136.5, 132.8, 131.4, 127.0 (d, J = 17.1 Hz), 126.8, 126.7, 126.3, 126.2, 125.6 (d, J = 1.8 Hz), 125.0, 124.9, 120.9 (dd, J = 8.0, 4.3 Hz), 116.8 (d, J = 19.1 Hz), 19.8; ^{19}F NMR (376 MHz, $CDCl_3$) δ -126.81--127.67 (m), -131.42 (dd, J = 21.7, 6.4 Hz); HRMS (ESI) Calcd. for $C_{17}H_{12}F_2NO_2$ $[M+H]^+$: 300.0836; Found: 300.0839; Enantiomeric excess was determined by chiral HPLC (Chiralcel OD-3, 25 ºC, flow rate: 0.5 mL/min, hexanes/isopropanol: 99/1, 290 nm, 20.64 min (major isomer), 21.59 min (minor isomer)).

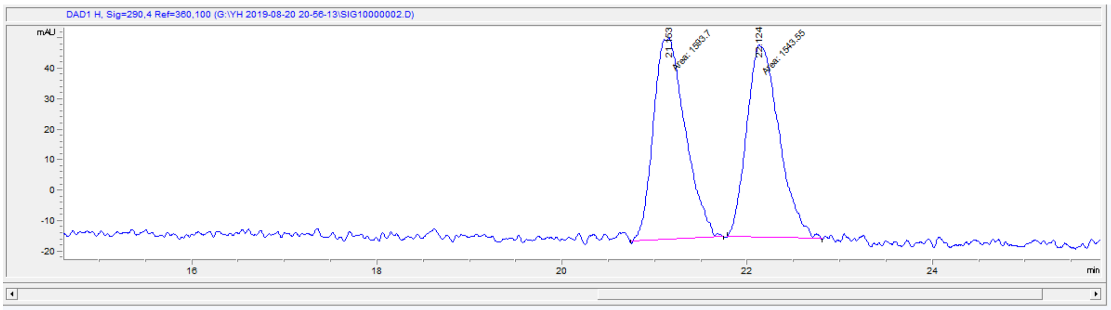

#	Time 时间	Area 峰面积	Height 峰高	Width 峰宽	Symmetry factor 对称因子	Area% 峰面积 %	Area%
1	21.153	1593.7	66.3	0.4006	0.918	50.799	50.799
2	22.124	1543.6	63.4	0.4058	0.612	49.201	49.201

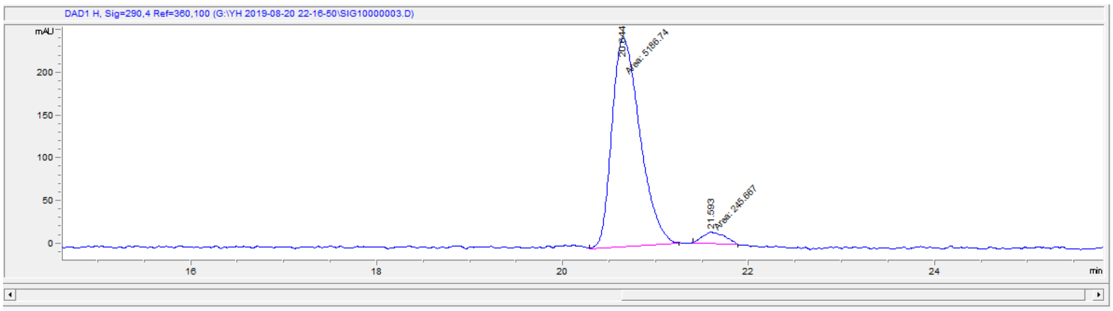

#	Time 时间	Area 峰面积	Height 峰高	Width 峰宽	Symmetry factor 对称因子	Area% 峰面积 %	Area%
1	20.644	5186.7	246	0.3514	0.629	95.478	95.478
2	21.593	245.7	14.1	0.2896	0.637	4.522	4.522

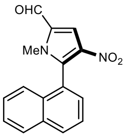

6h

(R)-1-methyl-5-(naphthalen-1-yl)-4-nitro-1H-pyrrole-2-carbaldehyde (6h)

Compound **6h** was obtained from the coupling reaction between 5-bromo-1-methyl-4-nitropyrrole-2-carbaldehyde and 1-naphthalene boronic acid in 80% yield and 88% ee. $[\alpha]^{25}_{D}$ = -21.6 (c = 0.5, $CHCl_3$); ^{1}H NMR (500 MHz, $CDCl_3$) δ 9.73 (s, 1H), 8.06 (d, J = 8.3 Hz, 1H), 7.97 (d, J = 8.2 Hz, 1H), 7.71 (s, 1H), 7.62 (dd, J = 8.2, 7.1 Hz, 1H), 7.59-7.54 (m, 1H), 7.51-7.46 (m, 2H), 7.29 (d, J = 8.3 Hz, 1H), 3.64 (s, 3H); ^{13}C NMR (151 MHz, $CDCl_3$) δ 180.4, 139.6, 135.7, 133.7, 131.8, 131.0, 130.3, 129.1, 128.7, 127.8 (d, J = 4.2 Hz), 126.9, 125.4, 125.2, 124.2, 119.0; HRMS (ESI) Calcd. for $C_{16}H_{13}N_2O_3$ $[M+H]^+$: 281.0926; Found: 281.0929; Enantiomeric excess was determined by chiral HPLC (Chiralcel OD-3, 25 °C, flow rate: 0.6 mL/min, hexanes/isopropanol: 80/20, 290 nm, 15.86 min (minor isomer), 18.03 min (major isomer)).

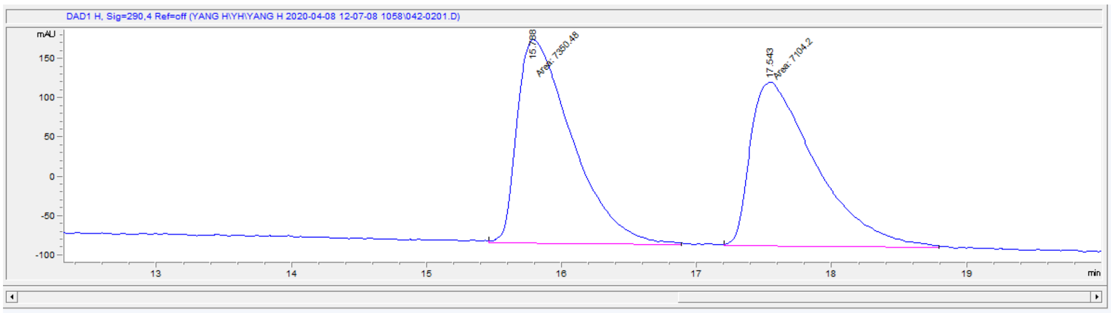

#	Time 时间	Area 峰面积	Height 峰高	Width 峰宽	Symmetry factor 对称因子	Area% 峰面积 %	Area%
1	15.788	7350.5	258.8	0.4733	0.406	50.852	50.852
2	17.543	7104.2	209.7	0.5646	0.419	49.148	49.148

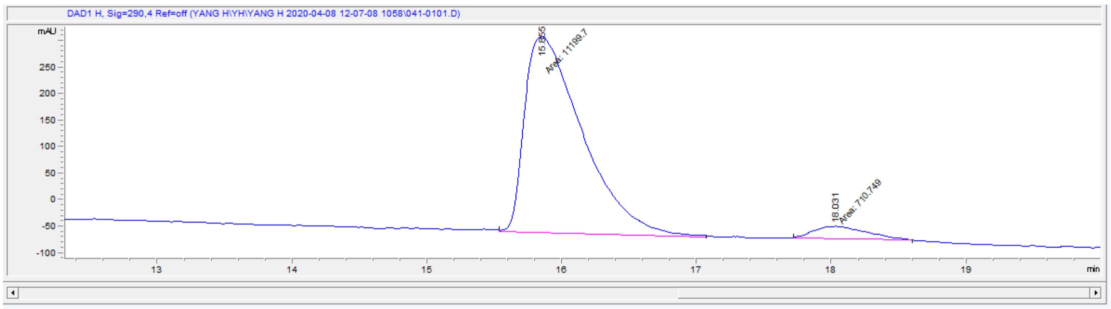

#	Time 时间	Area 峰面积	Height 峰高	Width 峰宽	Symmetry factor 对称因子	Area% 峰面积 %	Area%
1	15.855	11199.7	370.7	0.5035	0.429	94.033	94.033
2	18.031	710.7	24.8	0.4774	0.662	5.967	5.967

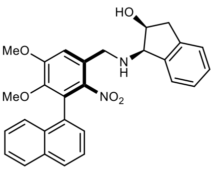

6i

(1*R*,2*S*)-1-(((*R*)-4,5-dimethoxy-3-(naphthalen-1-yl)-2-nitrobenzyl)amino)-2,3-dihydro-1H-inden-2-ol (6i)

Compound **6i** was obtained from the coupling reaction between **S20** and 1-naphthalene boronic acid in 64% yield and 92% de. $[\alpha]^{25}_{D}$ = -10.9 (c = 0.1, $CHCl_3$); ^{1}H NMR (400 MHz, $CDCl_3$) δ 7.89 (t, J = 7.5 Hz, 2H), 7.48 (dt, J = 17.5, 9.3 Hz, 4H), 7.39-7.28 (m, 3H), 7.20 (d, J = 9.6 Hz, 2H), 4.55-4.47 (m, 1H), 4.18 (dd, J = 5.0, 2.4 Hz, 1H), 4.02 (d, J = 4.4 Hz, 4H), 3.95 (dd, J = 24.9, 19.2 Hz, 1H), 3.45 (s, 3H), 3.10 (dd, J = 16.5, 5.3 Hz, 1H), 3.01 (d, J = 16.4 Hz, 1H); ^{13}C NMR (151 MHz, $CDCl_3$) δ 154.6 (d, J = 2.4 Hz), 146.9 (d, J = 7.4 Hz), 145.2 (d, J = 13.3 Hz), 141.9, 141.0 (d, J = 4.3 Hz), 132.3, 132.2, 130.4, 130.2, 129.2 (d, J = 5.9 Hz), 128.9 (d, J = 6.6 Hz), 128.4, 128.4 (d, J = 6.3 Hz), 127.4, 127.1, 126.9, 126.6 (d, J = 6.4 Hz), 126.2 (d, J = 4.6 Hz), 125.8, 125.6, 125.2, 124.1, 124.1, 113.0, 112.8, 71.1 (d, J = 16.8 Hz), 65.8 (d, J = 25.9 Hz), 61.2 (d, J = 2.8 Hz), 56.4, 49.1 (d, J = 49.4 Hz), 39.9 (d, J = 2.6 Hz); HRMS (ESI) Calcd. for $C_{28}H_{26}N_2NaO_5$ $[M+Na]^+$: 493.1739; Found: 493.1742; Enantiomeric excess was determined by chiral HPLC (Chiralcel OD-3, 25 ºC, flow rate: 0.7 mL/min, hexanes/isopropanol: 70/30, 230 nm, 15.93 min (minor isomer), 18.28 min (major isomer)).

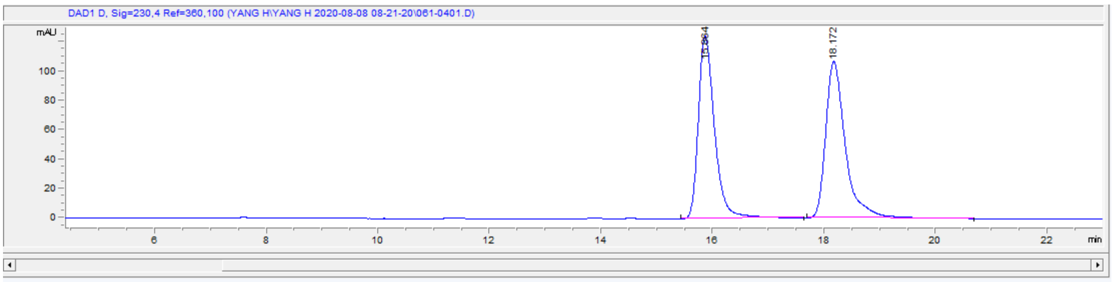

#	Time 时间	Area 峰面积	Height 峰高	Width 峰宽	Symmetry factor 对称因子	Area% 峰面积 %	Area%
1	15.864	2516.6	124.2	0.3083	0.649	48.998	48.998
2	18.172	2619.6	106.7	0.3705	0.632	51.002	51.002

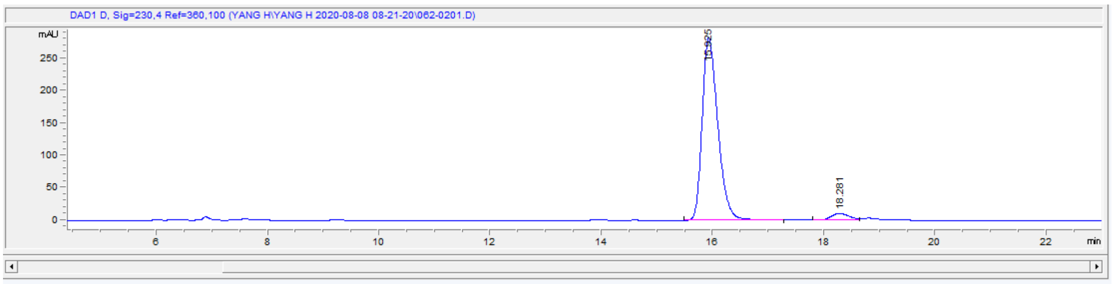

#	Time 时间	Area 峰面积	Height 峰高	Width 峰宽	Symmetry factor 对称因子	Area% 峰面积 %	Area%
1	15.925	5507.8	281.7	0.3001	0.672	95.958	95.958
2	18.281	232	10.4	0.3432	0.809	4.042	4.042

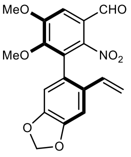

6j

(*R*)-4,5-dimethoxy-2-nitro-3-(6-vinylbenzo[d][1,3]dioxol-5-yl)benzaldehyde (6j)

Compound **6j** was obtained from the coupling reaction between 3-bromo-4,5-dimethoxy-2-nitrobenzaldehyde and **S18** in 89% yield and 94% ee. $[\alpha]^{25}_{D}$ = -20.7 (c = 0.5, $CHCl_3$); ^{1}H NMR (400 MHz, $CDCl_3$) δ 9.90 (s, 1H), 7.52 (s, 1H), 7.13 (s, 1H), 6.59 (s, 1H), 6.28 (dd, J = 17.3, 10.9 Hz, 1H), 6.02 (dd, J = 5.5, 1.3 Hz, 2H), 5.56 (d, J = 17.2 Hz, 1H), 5.10 (d, J = 11.2 Hz, 1H), 4.02 (s, 3H), 3.72 (s, 3H); ^{13}C NMR (151 MHz, $CDCl_3$) δ 186.1, 154.4, 152.3, 148.9, 147.5, 146.9, 133.6,

133.6, 131.5, 128.7, 123.4, 123.1, 114.5, 110.6, 109.6, 105.1, 101.7, 61.4, 56.6; HRMS (ESI) Calcd. for $C_{18}H_{16}NO_7$ $[M+H]^+$: 358.0927; Found: 358.0934; Enantiomeric excess was determined by chiral HPLC (Chiralcel OD-3, 25 °C, flow rate: 0.8 mL/min, hexanes/isopropanol: 80/20, 290 nm, 8.12 min (major isomer), 8.73 min (minor isomer)).

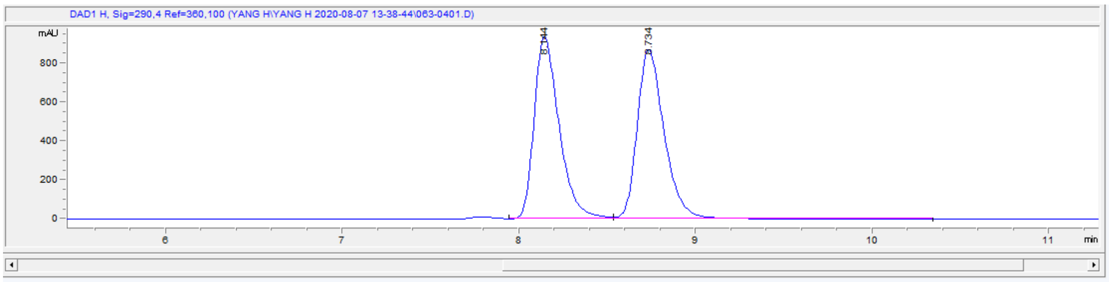

#	Time 时间	Area 峰面积	Height 峰高	Width 峰宽	Symmetry factor 对称因子	Area% 峰面积 %	Area%
1	8.144	9227.9	935.1	0.1511	0.647	49.848	49.848
2	8.734	9284	870.9	0.1624	0.683	50.152	50.152

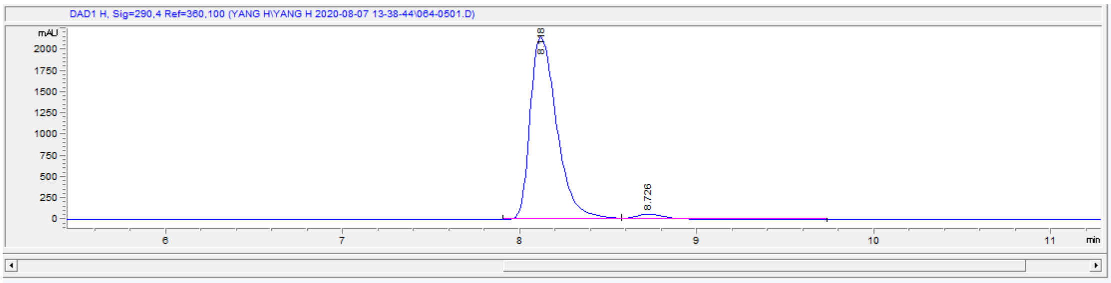

#	Time 时间	Area 峰面积	Height 峰高	Width 峰宽	Symmetry factor 对称因子	Area% 峰面积 %	Area%
1	8.118	22505.5	2149.3	0.1622	0.614	96.837	96.837
2	8.726	735.1	62.8	0.1726	0.712	3.163	3.163

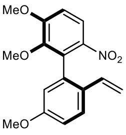

6k

(*R*)-2,3,5'-trimethoxy-6-nitro-2'-vinyl-1,1'-biphenyl (6k)

Compound **6k** was obtained from the coupling reaction between 2-bromo-3,4-dimethoxy-1-nitrobenzene and **S17** in 77% yield and 90% ee. $[\alpha]^{25}_D$ = -6.8 (c = 0.3, $CHCl_3$); ^1H NMR (500 MHz, $CDCl_3$) δ 7.90 (d, J = 9.1 Hz, 1H), 7.60 (d, J = 8.7 Hz, 1H), 7.00 (d, J = 9.1 Hz, 1H), 6.93 (dd, J = 8.7, 2.7 Hz, 1H), 6.63 (d, J = 2.6 Hz, 1H), 6.31 (dd, J = 17.4, 11.0 Hz, 1H), 5.53 (d, J = 17.4 Hz, 1H), 5.02 (d, J = 11.0 Hz, 1H), 3.99 (s, 3H), 3.80 (s, 3H), 3.55 (s, 3H); ^{13}C NMR (126 MHz, $CDCl_3$) δ 158.9, 157.2, 147.1, 142.6, 133.9, 133.7, 131.4, 129.2, 126.3, 121.5, 114.3, 114.1, 113.6, 110.6, 60.9, 56.4, 55.4; HRMS (ESI) Calcd. for $C_{17}H_{18}NO_5$ $[M+H]^+$: 316.1185; Found: 316.1189; Enantiomeric excess was determined by chiral HPLC (Chiralcel OD-3, 25 °C, flow rate: 0.7 mL/min, hexanes/isopropanol: 80/20, 214 nm, 11.79 min (major isomer), 15.14 min (minor isomer)).

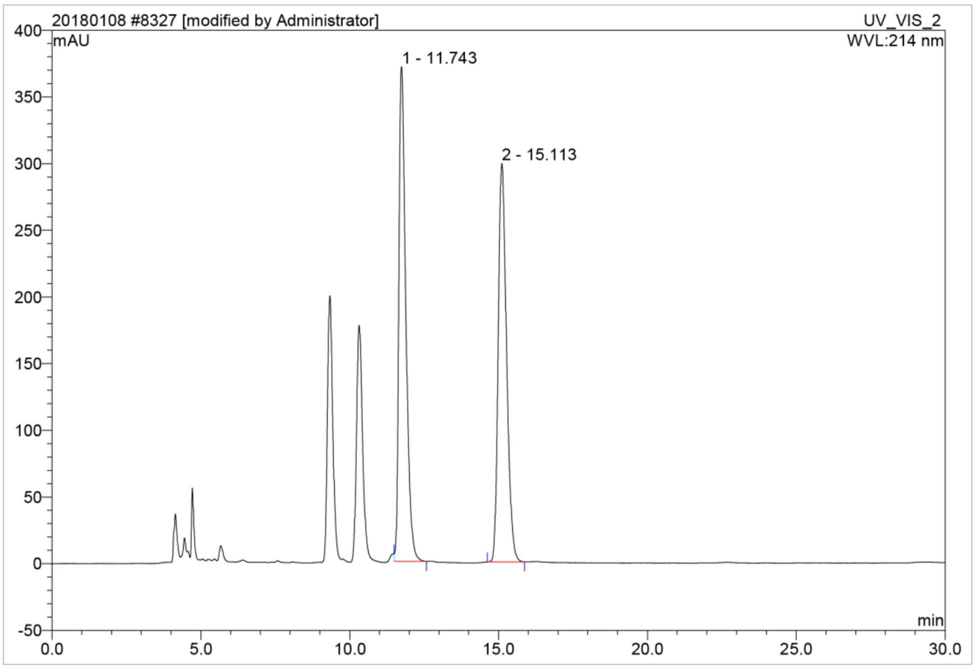

No.	Ret.Time min	Peak Name	Height mAU	Area mAU*min	Rel.Area %	Amount	Type
1	11.74	n.a.	370.884	100.784	50.93	n.a.	MB*
2	15.11	n.a.	298.951	97.086	49.07	n.a.	BMB
Total:			669.835	197.870	100.00	0.000	

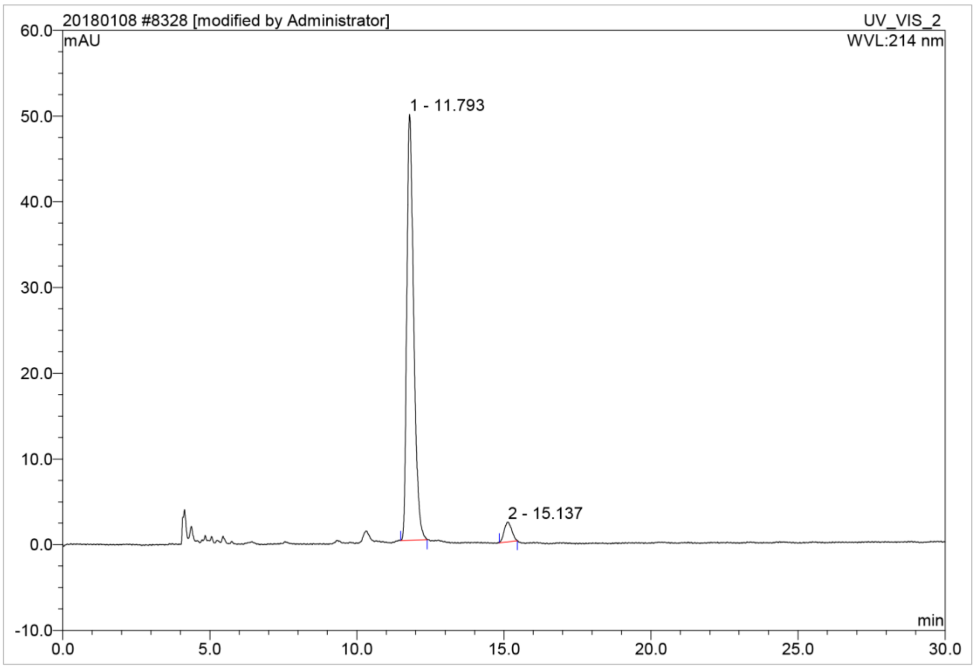

No.	Ret.Time min	Peak Name	Height mAU	Area mAU*min	Rel.Area %	Amount	Type
1	11.79	n.a.	49.704	13.503	95.21	n.a.	BMB
2	15.14	n.a.	2.299	0.679	4.79	n.a.	BMB
Total:			52.003	14.182	100.00	0.000	

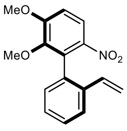

6l

(*R*)-2,3-dimethoxy-6-nitro-2'-vinyl-1,1'-biphenyl (6l)

Compound **6l** was obtained from the coupling reaction between 2-bromo-3,4-dimethoxy-1-nitrobenzene and (2-vinylphenyl)boronic acid in 88% yield and 90% ee. $[\alpha]^{25}_{D}$ = -33.2 (*c* = 1.0, $CHCl_3$); ^1H NMR (500 MHz, $CDCl_3$) δ 7.90 (d, *J* = 9.1 Hz, 1H), 7.67 (d, *J* = 7.8 Hz, 1H), 7.39 (t, *J* = 7.6 Hz, 1H), 7.31 (td, *J* = 7.5, 0.8 Hz, 1H), 7.10 (dd, *J* = 7.5, 0.8 Hz, 1H), 7.01 (d, *J* = 9.1 Hz, 1H), 6.41 (dd, *J* = 17.4, 11.0 Hz, 1H), 5.67 (d, *J* = 17.4 Hz, 1H), 5.14 (d, *J* = 11.0 Hz, 1H), 3.99 (s, 3H), 3.53 (s, 3H); ^{13}C NMR (126 MHz, $CDCl_3$) δ 157.2, 147.1, 142.6, 136.3, 134.5, 132.5, 131.5, 129.0, 128.3, 127.5, 125.1, 121.5, 115.7, 110.6, 60.8, 56.4; HRMS (ESI) Calcd. for $C_{16}H_{16}NO_4$ $[M+H]^+$: 286.1079; Found: 286.1080; Enantiomeric excess was determined by chiral HPLC (Chiralcel OD-3, 25 °C, flow rate: 0.7 mL/min, hexanes/isopropanol: 80/20, 250 nm, 9.86 min (major isomer), 10.91 min (minor isomer)).

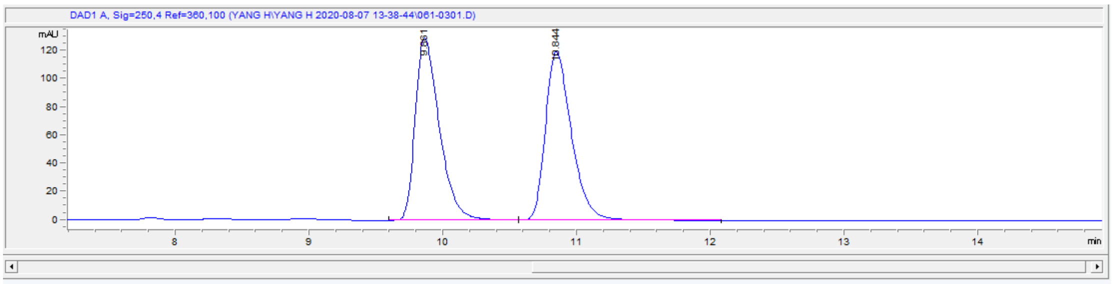

#	Time 时间	Area 峰面积	Height 峰高	Width 峰宽	Symmetry factor 对称因子	Area% 峰面积 %	Area%
1	9.861	1618.5	129.1	0.1903	0.595	50.127	50.127
2	10.844	1610.3	119.6	0.2051	0.657	49.873	49.873

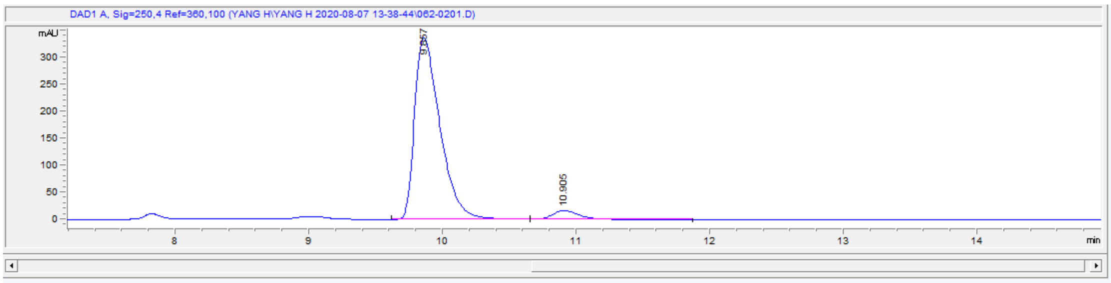

#	Time 时间	Area 峰面积	Height 峰高	Width 峰宽	Symmetry factor 对称因子	Area% 峰面积 %	Area%
1	9.857	4435	337.6	0.1993	0.518	95.115	95.115
2	10.905	227.8	16.5	0.209	0.713	4.885	4.885

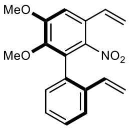

6m

(*R*)-2,3-dimethoxy-6-nitro-2',5-divinyl-1,1'-biphenyl (6m)

Compound **6m** was obtained from the coupling reaction between **S14** and (2-vinylphenyl)boronic acid in 79% yield and 89% ee. $[\alpha]^{25}_{D}$ = -54.5 (*c* = 0.5, $CHCl_3$); ^1H NMR (400 MHz, $CDCl_3$) δ 7.65 (d, *J* = 7.8 Hz, 1H), 7.38 (t, *J* = 7.3 Hz, 1H), 7.28 (d, *J* = 7.5 Hz, 1H), 7.17-7.09 (m, 2H), 6.66 (dd, *J* = 17.2, 11.0 Hz, 1H), 6.50 (dd, *J* = 17.5, 11.0 Hz, 1H), 5.80 (d, *J* = 17.2 Hz, 1H), 5.70 (d, *J* = 18.0 Hz, 1H), 5.46 (d, *J* = 11.0 Hz, 1H), 5.18 (d, *J* = 11.1 Hz, 1H), 3.99 (s, 3H), 3.56 (s, 3H); ^{13}C NMR (151 MHz, $CDCl_3$) δ 154.2, 146.8, 143.8, 137.0, 134.6, 130.9, 130.3, 129.7, 129.0, 128.6, 127.6, 126.2, 125.1, 118.6, 115.7, 108.4, 61.0, 56.3; HRMS (ESI) Calcd. for $C_{18}H_{18}NO_4$ $[M+H]^+$: 312.1236; Found: 312.1241; Enantiomeric excess was determined by chiral HPLC (Chiralcel OD-3, 25 °C, flow rate: 0.7 mL/min, hexanes/isopropanol: 80/20, 214 nm, 7.87 min (major isomer), 8.43 min (minor

isomer)).

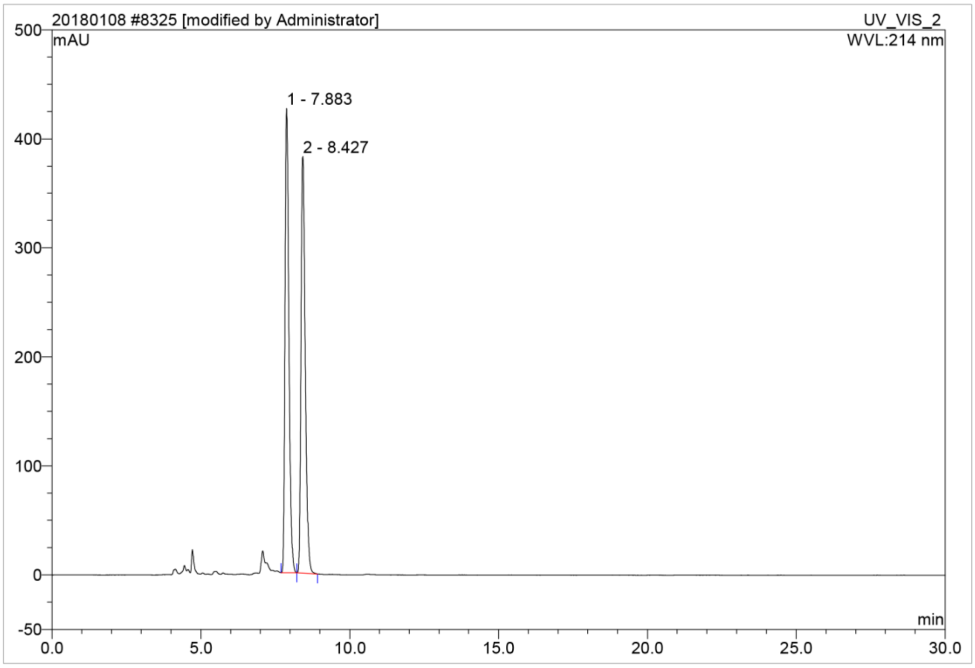

No.	Ret.Time min	Peak Name	Height mAU	Area mAU*min	Rel.Area %	Amount	Type
1	7.88	n.a.	426.113	63.828	50.45	n.a.	BMB
2	8.43	n.a.	382.369	62.687	49.55	n.a.	BMB
Total:			808.482	126.516	100.00	0.000	

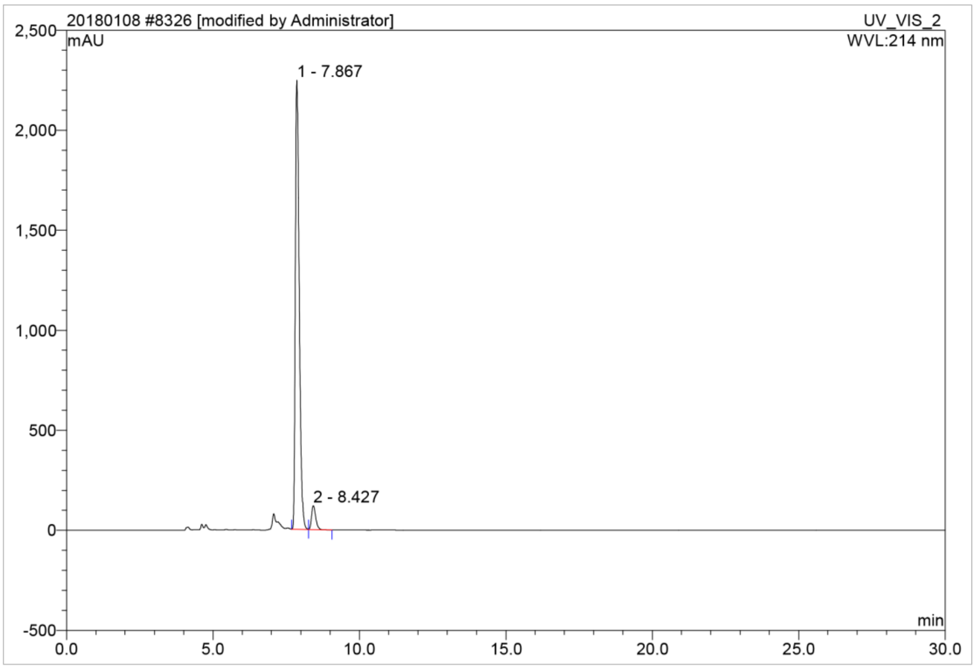

No.	Ret.Time min	Peak Name	Height mAU	Area mAU*min	Rel.Area %	Amount	Type
1	7.87	n.a.	2245.178	343.859	94.55	n.a.	BM *
2	8.43	n.a.	120.433	19.818	5.45	n.a.	MB*
Total:			2365.611	363.678	100.00	0.000	

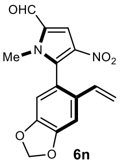

(*S*)-1-methyl-4-nitro-5-(6-vinylbenzo[d][1,3]dioxol-5-yl)-1H-pyrrole-2-carbaldehyde (6n)

Compound **6n** was obtained from the coupling reaction between 5-bromo-1-methyl-4-nitropyrrole-2-carbaldehyde and **S18** in 86% yield and 90% ee. $[\alpha]^{25}_{D}$ = 11.0 (*c* = 0.5, $CHCl_3$); ^{1}H NMR (500 MHz, $CDCl_3$) δ 9.65 (s, 1H), 7.59 (s, 1H), 7.16 (s, 1H), 6.66 (s, 1H), 6.11 (dd, *J* = 17.3, 11.0 Hz, 1H), 6.08 (s, 2H), 5.58 (d, *J* = 17.3 Hz, 1H), 5.16 (d, *J* = 11.0 Hz, 1H), 3.65 (s, 3H); ^{13}C NMR (151 MHz, $CDCl_3$) δ 180.3, 150.1, 148.0, 139.5, 132.8, 132.6, 130.0, 119.4, 118.9, 116.1, 109.6, 105.5, 102.1, 34.2; HRMS (ESI) Calcd. for $C_{15}H_{13}N_2O_5$ $[M+H]^+$: 301.0819; Found: 301.0815; Enantiomeric excess was determined by chiral HPLC (Chiralcel OD-3, 25 ºC, flow rate: 0.8 mL/min, hexanes/isopropanol: 70/30, 290 nm, 11.82 min (minor isomer), 12.72 min (major isomer)).

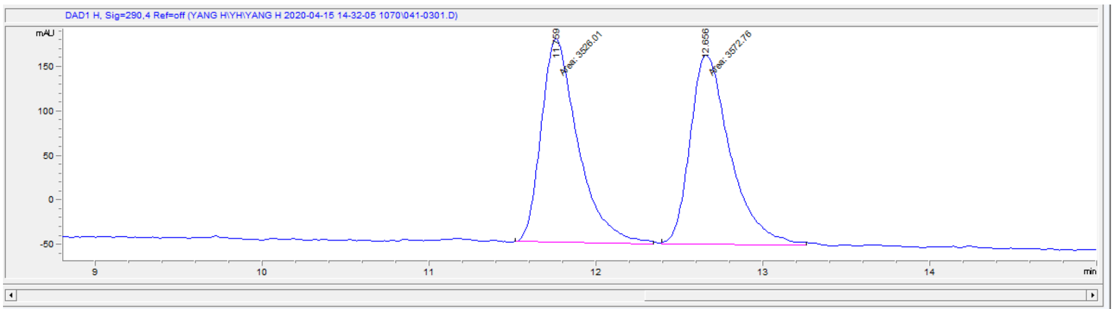

#	Time 时间	Area 峰面积	Height 峰高	Width 峰宽	Symmetry factor 对称因子	Area% 峰面积 %	Area%
1	11.759	3526	229.3	0.2562	0.649	49.671	49.671
2	12.656	3572.8	212.9	0.2796	0.63	50.329	50.329

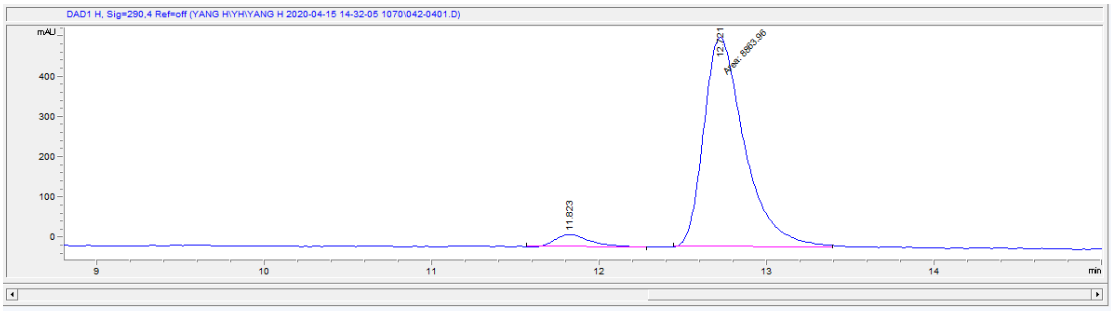

#	Time 时间	Area 峰面积	Height 峰高	Width 峰宽	Symmetry factor 对称因子	Area% 峰面积 %	Area%
1	11.823	460.9	30.9	0.2177	0.65	4.942	4.942
2	12.721	8864	520.3	0.284	0.654	95.058	95.058

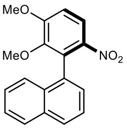

6o

(*R*)-1-(2,3-dimethoxy-6-nitrophenyl)naphthalene (6o)

Compound **6o** was obtained from the coupling reaction between 2-bromo-3,4-dimethoxy-1-nitrobenzene and 1-naphthalene boronic acid in 90% yield and 96% ee. $[\alpha]^{25}_{D}$ = -104.6 (*c* = 1.0, $CHCl_3$); ^{1}H NMR (500 MHz, $CDCl_3$) δ 7.98 (d, *J* = 9.1 Hz, 1H), 7.90 (d, *J* = 8.2 Hz, 2H), 7.52 (t, *J* = 7.6 Hz, 1H), 7.47 (t, *J* = 7.4 Hz, 1H), 7.45-7.35 (m, 2H), 7.31 (d, *J* = 7.0 Hz, 1H), 7.08 (d, *J* = 9.2 Hz, 1H), 4.02 (s, 3H), 3.42 (s, 3H); ^{13}C NMR (126 MHz, $CDCl_3$) δ 157.2, 147.7, 143.1, 133.5, 132.1, 131.7, 131.1, 128.5 (d, *J* = 2.5 Hz), 126.4, 126.2, 126.0, 125.2 (d, *J* = 16.1 Hz), 121.6, 110.8, 61.2, 56.4; HRMS (ESI) Calcd. for $C_{18}H_{16}NO_4$ $[M+H]^+$: 310.1079; Found: 310.1076; Enantiomeric excess was determined by chiral HPLC (Chiralcel OD-3, 25 ºC, flow rate: 0.7 mL/min, hexanes/isopropanol: 80/20, 214 nm,

12.20 min (major isomer), 13.50 min (minor isomer)).

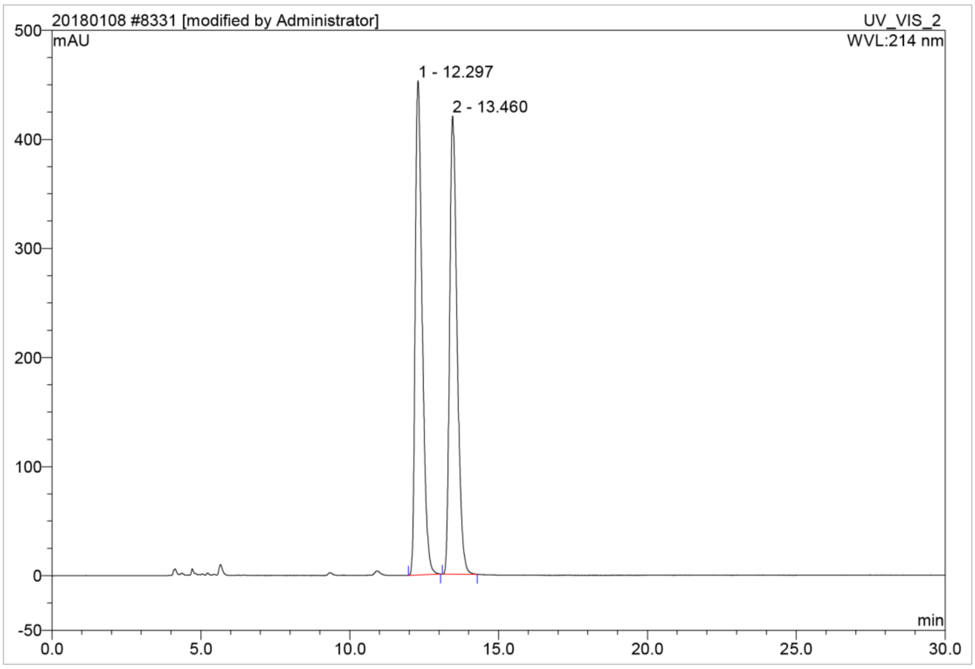

No.	Ret.Time min	Peak Name	Height mAU	Area mAU*min	Rel.Area %	Amount	Type
1	12.30	n.a.	453.053	123.394	50.18	n.a.	BMB
2	13.46	n.a.	420.145	122.522	49.82	n.a.	BMB
Total:			873.199	245.916	100.00	0.000	

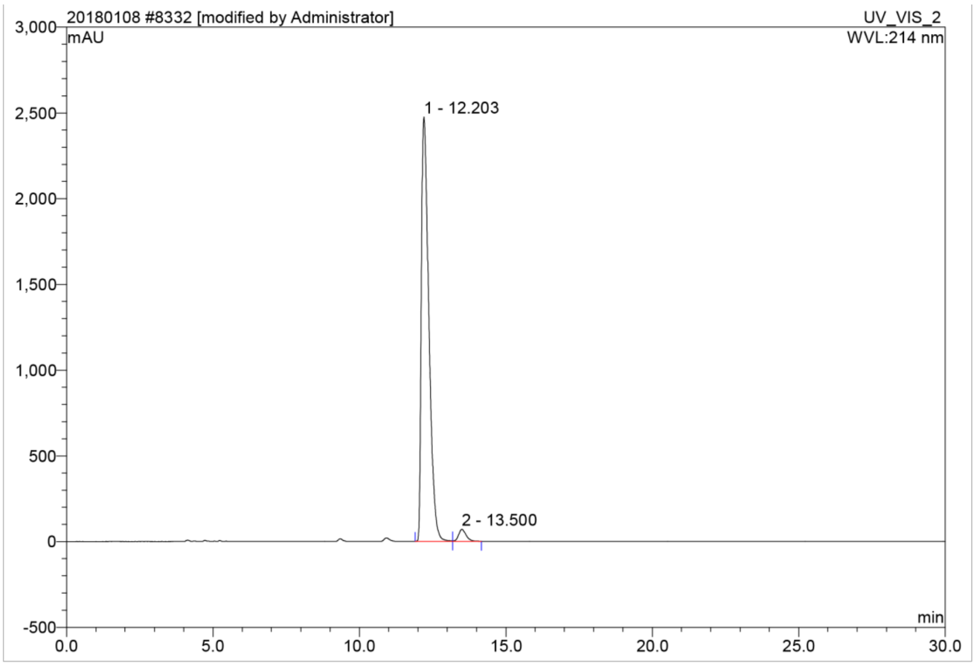

No.	Ret.Time min	Peak Name	Height mAU	Area mAU*min	Rel.Area %	Amount	Type
1	12.20	n.a.	2476.215	735.668	97.26	n.a.	BM
2	13.50	n.a.	69.789	20.713	2.74	n.a.	MB
Total:			2546.004	756.380	100.00	0.000	

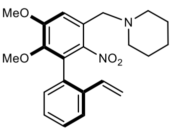

6p

(*R*)-1-((5,6-dimethoxy-2-nitro-2'-vinyl-[1,1'-biphenyl]-3-yl)methyl)piperidine (6p)

Compound **6p** was obtained from the coupling reaction between **S19** and (2-vinylphenyl)boronic acid in 70% yield and 92% ee. $[\alpha]^{25}_D$ = -24.2 (c = 0.1, $CHCl_3$); ^1H NMR (500 MHz, $CDCl_3$) δ 7.64 (d, J = 8.2 Hz, 1H), 7.36 (t, J = 7.3 Hz, 1H), 7.29-7.24 (m, 1H), 7.19 (s, 1H), 7.13 (dd, J = 7.6, 1.0 Hz, 1H), 6.47 (dd, J = 17.5, 11.0 Hz, 1H), 5.68 (d, J = 16.7 Hz, 1H), 5.16 (d, J = 11.0 Hz, 1H), 3.96 (s, 3H), 3.53 (s, 3H), 3.47 (s, 2H), 2.37 (s, 4H), 1.60-1.51 (m, 4H), 1.47-1.37 (m, 2H); ^{13}C NMR (126 MHz, $CDCl_3$) δ 154.1, 145.5, 144.3, 136.9, 134.7, 131.4, 129.8, 128.8, 128.7, 128.2, 127.5, 125.0, 115.5, 112.2, 60.9, 58.9, 56.2, 54.7, 26.1, 24.4; HRMS (ESI) Calcd. for $C_{22}H_{27}N_2O_4$ $[M+H]^+$: 383.1971; Found: 383.1970; Enantiomeric excess was determined by chiral HPLC (Chiralcel OD-3, 25 °C, flow rate: 0.8 mL/min, hexanes/isopropanol: 70/30, 290 nm, 7.13 min (major isomer), 8.18 min (minor isomer)).

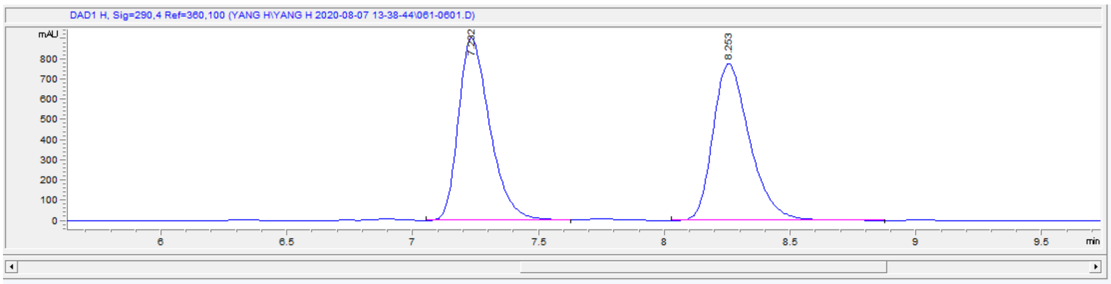

#	Time 时间	Area 峰面积	Height 峰高	Width 峰宽	Symmetry factor 对称因子	Area% 峰面积 %	Area%
1	7.232	7852.9	903.9	0.1334	0.662	49.934	49.934
2	8.253	7873.5	774.7	0.1546	0.681	50.066	50.066

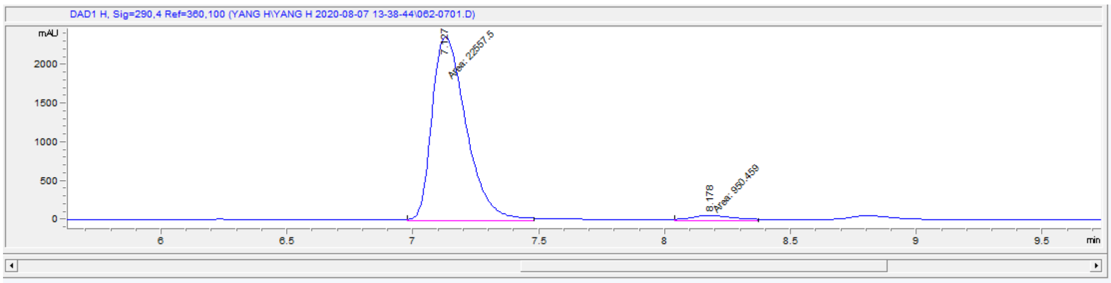

#	Time 时间	Area 峰面积	Height 峰高	Width 峰宽	Symmetry factor 对称因子	Area% 峰面积 %	Area%
1	7.127	22557.5	2374.8	0.1583	0.607	95.957	95.957
2	8.178	950.5	75.9	0.2088	0.827	4.043	4.043

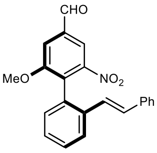

6q

(*R*,*E*)-2-methoxy-6-nitro-2'-styryl-[1,1'-biphenyl]-4-carbaldehyde (6q)

Compound **6q** was obtained from the coupling reaction between **S27** and 2-((*E*)-2-phenylethenyl)phenylboronic acid[10] in 80% yield and 82% ee. $[\alpha]^{25}_D$ = -199.8 (c = 1.0, $CHCl_3$); ^1H NMR (500 MHz, $CDCl_3$) δ 10.08 (s, 1H), 8.01 (s, 1H), 7.79 (d, J = 7.9 Hz, 1H), 7.70 (s, 1H), 7.45 (t, J = 7.6 Hz, 1H), 7.33 (t, J = 7.5 Hz, 1H), 7.28 (d, J = 4.2 Hz, 4H), 7.24 – 7.19 (m, 1H), 7.09 (d, J = 7.6 Hz, 1H), 7.04 (d, J = 16.1 Hz, 1H), 6.70 (d, J = 16.1 Hz, 1H), 3.84 (s, 3H); ^{13}C NMR (126 MHz, $CDCl_3$) δ 189.7, 158.9, 151.3, 137.3, 137.1, 136.4, 131.0, 131.0, 130.6, 129.2, 128.9, 128.7, 128.0, 127.7, 126.7, 125.8, 125.6, 118.5, 112.4, 57.0; HRMS (ESI) Calcd. for $C_{22}H_{18}NO_4$ $[M+H]^+$: 360.1236; Found: 360.1241; Enantiomeric excess was determined by chiral HPLC (Chiralcel AD-3, 25 °C, flow rate: 0.7 mL/min,

hexanes/isopropanol: 70/30, 290 nm, 11.52 min (minor isomer), 14.62 min (major isomer)).

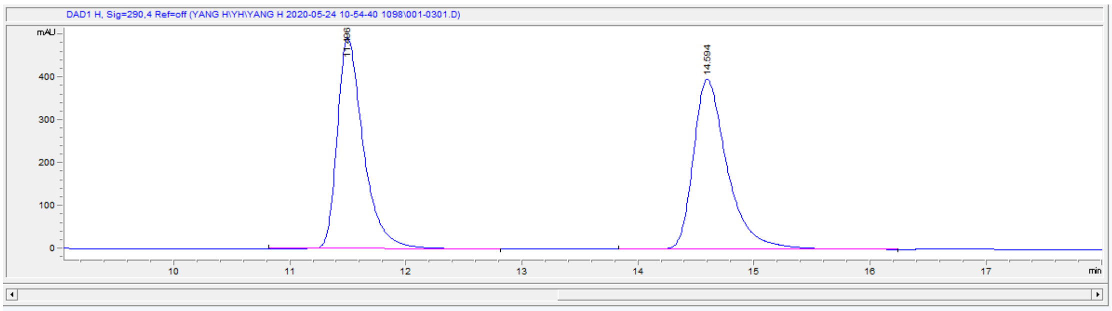

#	Time 时间	Area 峰面积	Height 峰高	Width 峰宽	Symmetry factor 对称因子	Area% 峰面积 %	Area%
1	11.496	7696.3	492.8	0.2342	0.619	48.850	48.850
2	14.594	8058.8	397.4	0.3046	0.633	51.150	51.150

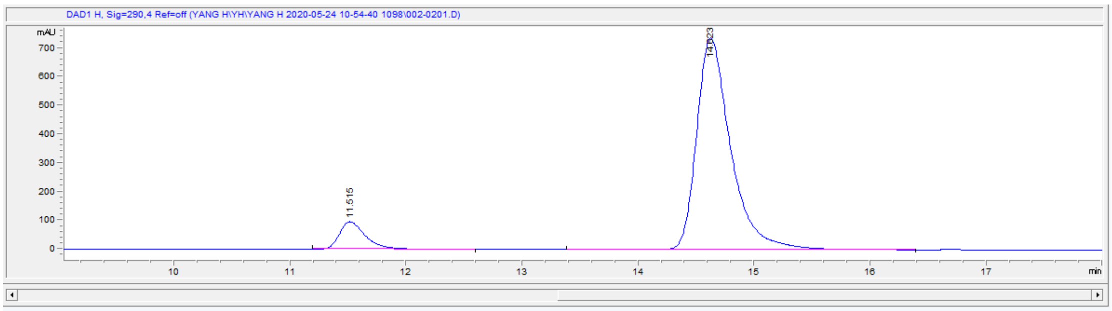

#	Time 时间	Area 峰面积	Height 峰高	Width 峰宽	Symmetry factor 对称因子	Area% 峰面积 %	Area%
1	11.515	1491.5	95.8	0.2337	0.623	9.040	9.040
2	14.623	15007.6	736.5	0.3057	0.639	90.960	90.960

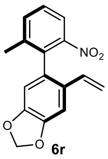

(*S*)-5-(2-methyl-6-nitrophenyl)-6-vinylbenzo[d][1,3]dioxole (6r)

Compound **6r** was obtained from the coupling reaction between 2-bromo-1-methyl-3-nitrobenzene and **S18** in 92% yield and 86% ee. $[\alpha]^{25}_{D}$ = -29.8 (*c* = 0.2, $CHCl_3$); ^1H NMR (500 MHz, $CDCl_3$) δ 7.71 (d, *J* = 8.0 Hz, 1H), 7.48 (d, *J* = 7.5 Hz, 1H), 7.40 (t, *J* = 7.9 Hz, 1H), 7.13 (s, 1H), 6.51 (s, 1H), 6.16 (dd, *J* = 17.4, 10.9 Hz, 1H), 6.01 (dd, *J* = 10.3, 1.2 Hz, 2H), 5.53 (d, *J* = 17.3 Hz, 1H), 5.03 (d, *J* = 11.0 Hz, 1H), 2.06 (s, 3H); ^{13}C NMR (151 MHz, $CDCl_3$) δ 140.3, 134.0, 134.0, 133.5, 130.4, 128.3, 121.4, 114.1, 108.7, 105.0, 101.6, 20.5; HRMS (ESI) Calcd. for $C_{16}H_{14}NO_4$ $[M+H]^+$: 284.0923; Found: 284.0931; Enantiomeric excess was determined by chiral HPLC (Chiralcel OD-3, 25 °C, flow rate: 0.7 mL/min, hexanes/isopropanol: 80/20, 214 nm, 8.86 min (minor isomer), 9.88 min (major isomer)).

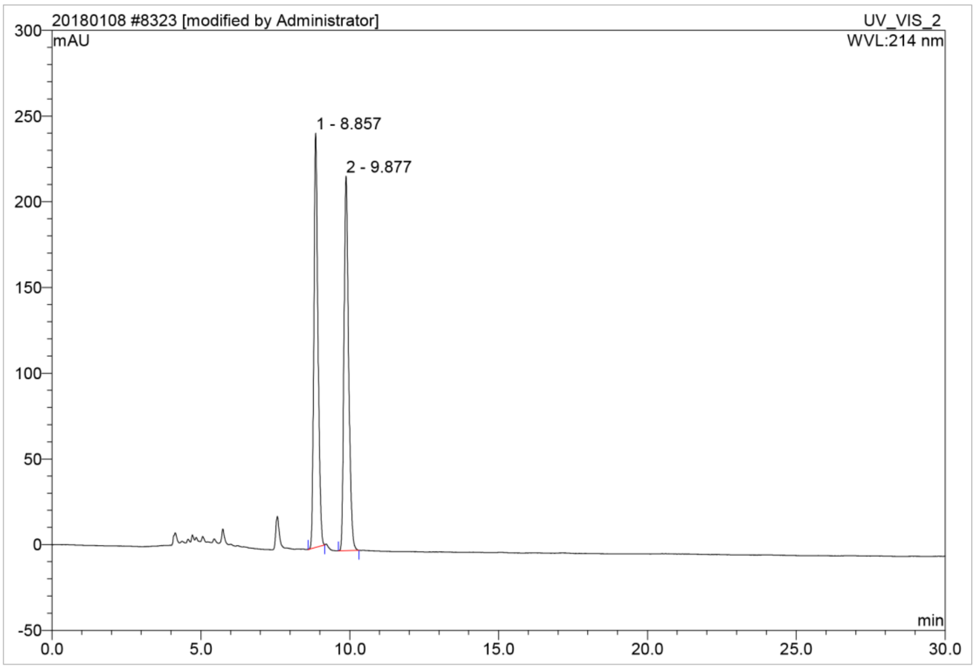

No.	Ret.Time min	Peak Name	Height mAU	Area mAU*min	Rel.Area %	Amount	Type
1	8.86	n.a.	241.746	38.726	49.42	n.a.	BMB*
2	9.88	n.a.	218.624	39.638	50.58	n.a.	BMB
Total:			460.371	78.364	100.00	0.000	

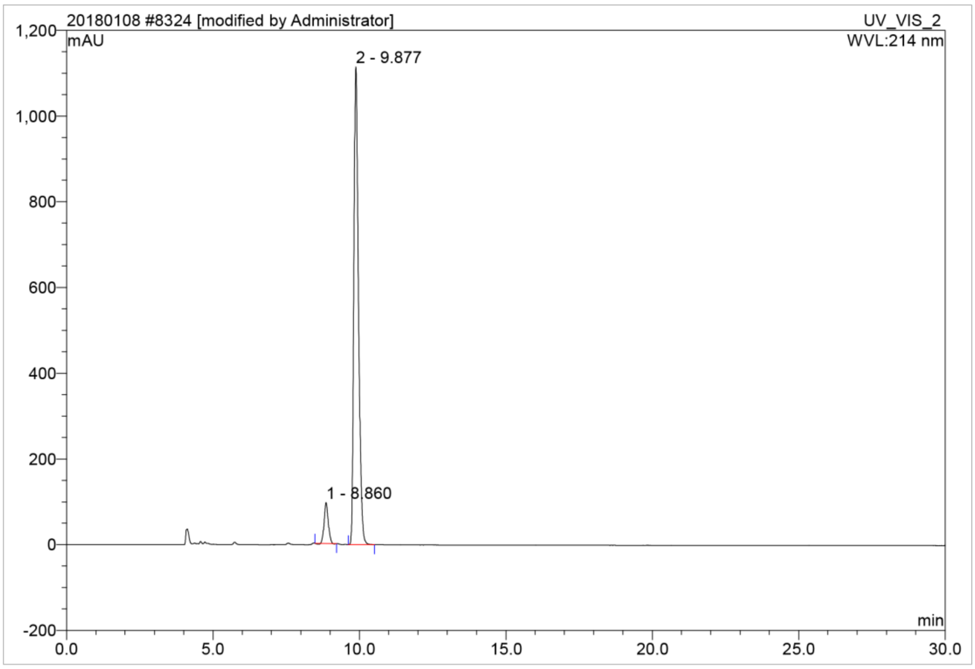

No.	Ret.Time min	Peak Name	Height mAU	Area mAU*min	Rel.Area %	Amount	Type
1	8.86	n.a.	95.878	15.343	7.00	n.a.	BMB*
2	9.88	n.a.	1115.248	203.863	93.00	n.a.	BMB
Total:			1211.126	219.206	100.00	0.000	

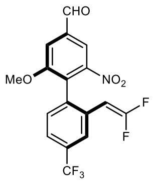

6s

(R)-2'-(2,2-difluorovinyl)-2-methoxy-6-nitro-4'-(trifluoromethyl)-[1,1'-biphenyl]-4-carbaldehyde (6s)

Compound **6s** was obtained from the coupling reaction between **S27** and **S11** in 88% yield and 82% ee. $[\alpha]^{25}_{D}$ = -81.9 (c = 0.5, $CHCl_3$); ^1H NMR (400 MHz, $CDCl_3$) δ 10.09 (s, 1H), 8.07 (d, J = 1.2 Hz, 1H), 7.86 (s, 1H), 7.73 (d, J = 1.1 Hz, 1H), 7.56 (d, J = 7.9 Hz, 1H), 7.19 (d, J = 8.1 Hz, 1H), 5.05-4.63 (m, 1H), 3.88 (s, 3H); ^{13}C NMR (151 MHz, $CDCl_3$) δ 189.3, 158.6, 156.9 (t, J = 295.2 Hz), 150.6, 137.9, 135.1, 131.5 (q, J = 32.7 Hz), 130.5, 129.3, 128.7, 125.0 (dd, J = 8.6, 4.8 Hz), 124.2 (d, J = 3.5 Hz), 123.9 (d, J = 272.4 Hz), 118.6, 113.0, 79.1 (dd, J = 23.0, 22.0 Hz), 57.1; ^{19}F NMR (376 MHz, $CDCl_3$) δ -62.9, -80.5 (d, J = 2.6 Hz), -80.6; HRMS (ESI) Calcd. for $C_{17}H_{11}F_5NO_4$ $[M+H]^+$: 388.0608; Found: 388.0612; Enantiomeric excess was determined by chiral HPLC (Chiralcel OD-3, 25 °C, flow rate: 0.7 mL/min, hexanes/isopropanol: 90/10, 260 nm, 6.06 min (major isomer), 6.77 min (minor isomer)).

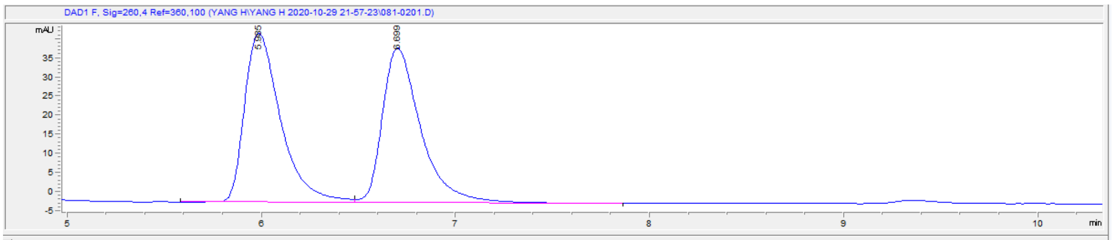

#	Time 时间	Area 峰面积	Height 峰高	Width 峰宽	Symmetry factor 对称因子	Area% 峰面积 %	Area%
1	5.985	572.5	44.2	0.1992	0.594	50.677	50.677
2	6.699	557.2	40.4	0.2088	0.594	49.323	49.323

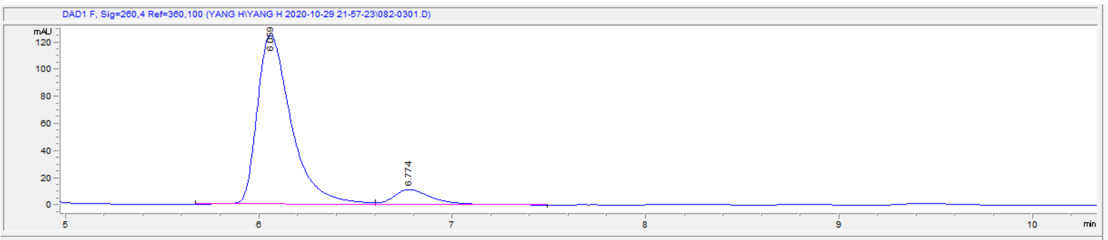

#	Time 时间	Area 峰面积	Height 峰高	Width 峰宽	Symmetry factor 对称因子	Area% 峰面积 %	Area%
1	6.059	1530.2	124.6	0.1873	0.585	90.916	90.916
2	6.774	152.9	11.2	0.2055	0.617	9.084	9.084

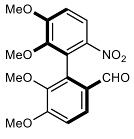

6t

(S)-2',3',5,6-tetramethoxy-6'-nitro-[1,1'-biphenyl]-2-carbaldehyde (6t)

Compound **6t** was obtained from the coupling reaction (at 70 °C) between 2-bromo-3,4-dimethoxy-1-nitrobenzene and 3,4-Dimethoxy-2-(trifluoro-λ^4-boraneyl)benzaldehyde, potassium salt[4] at 70 °C in 78% yield and 81% ee. $[\alpha]^{25}_{D}$ = -19.8 (c = 0.3, $CHCl_3$); ^1H NMR (500 MHz, $CDCl_3$) δ 9.64 (s, 1H), 8.05 (d, J = 9.2 Hz, 1H), 7.77 (d, J = 8.6 Hz, 1H), 7.09 (d, J = 8.6 Hz, 1H), 7.05 (d, J = 9.2 Hz, 1H), 4.00 (s, 3H), 3.98 (s, 3H), 3.61 (s, 3H), 3.55 (s, 3H); ^{13}C NMR (151 MHz, $CDCl_3$) δ 190.2, 157.5, 157.3, 146.9, 145.8, 142.0, 131.0, 127.9, 126.1, 121.9, 111.6, 111.0, 60.7, 60.6, 56.3, 56.1; HRMS (ESI) Calcd. for $C_{17}H_{18}NO_7$ $[M+H]^+$: 348.1078; Found: 348.1073; Enantiomeric excess was determined by chiral HPLC (Chiralcel AD-3, 25 °C, flow rate: 0.8 mL/min, hexanes/isopropanol: 70/30, 290 nm, 12.65 min (major isomer), 13.65 min (minor isomer)).

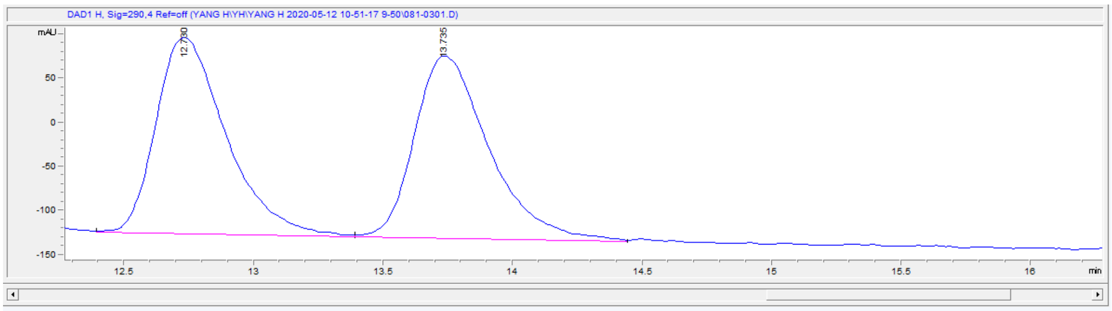

#	Time 时间	Area 峰面积	Height 峰高	Width 峰宽	Symmetry factor 对称因子	Area% 峰面积 %	Area%
1	12.73	4226	222.4	0.2856	0.628	50.330	50.330
2	13.735	4170.6	207.2	0.2948	0.634	49.670	49.670

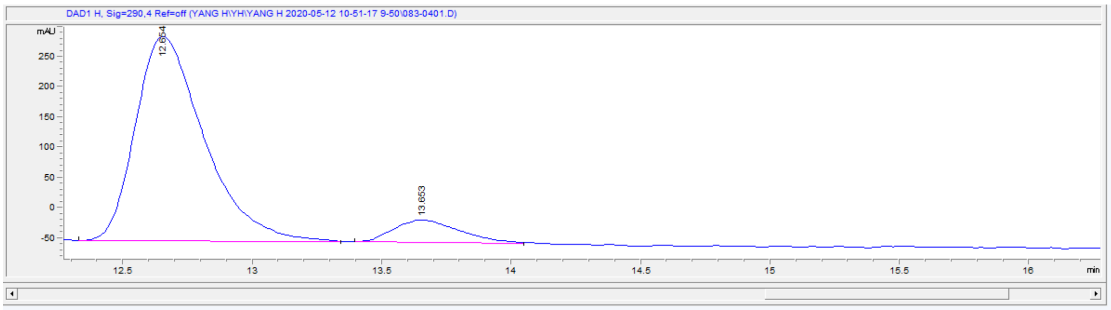

#	Time 时间	Area 峰面积	Height 峰高	Width 峰宽	Symmetry factor 对称因子	Area% 峰面积 %	Area%
1	12.654	6255.1	338.2	0.2758	0.633	90.344	90.344
2	13.653	668.6	37.7	0.223	0.679	9.656	9.656

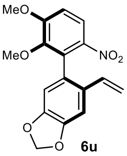

6u

(*R*)-5-(2,3-dimethoxy-6-nitrophenyl)-6-vinylbenzo[d][1,3]dioxole (6u) Compound **6u** was obtained from the coupling reaction between 2-bromo-3,4-dimethoxy-1-nitrobenzene and **S18** in 92% yield and 96% ee. $[\alpha]^{25}_{D}$ = 3.1 (*c* = 0.5, $CHCl_3$); ^1H NMR (600 MHz, $CDCl_3$) δ 7.87 (d, *J* = 9.1 Hz, 1H), 7.13 (s, 1H), 6.99 (d, *J* = 9.1 Hz, 1H), 6.58 (s, 1H), 6.25 (dd, *J* = 17.3, 11.0 Hz, 1H), 6.03 (d, *J* = 1.4 Hz, 1H), 6.00 (d, *J* = 1.4 Hz, 1H), 5.52 (dd, *J* = 17.3, 0.7 Hz, 1H), 5.02 (dd, *J* = 10.9, 0.7 Hz, 1H), 3.99 (s, 3H), 3.57 (s, 3H); ^{13}C NMR (151 MHz, $CDCl_3$) δ 157.1, 148.2, 147.4, 147.4, 143.0, 133.9 131.0, 130.5, 126.1, 121.4, 113.9, 110.7, 109.2, 104.9, 101.5, 60.9, 56.4; HRMS (ESI) Calcd. for $C_{17}H_{16}NO_6$ $[M+H]^+$: 330.0978; Found: 330.0981; Enantiomeric excess was determined by chiral HPLC (Chiralcel IC-3, 25 °C, flow rate: 0.6 mL/min, hexanes/isopropanol: 80/20, 230 nm, 20.89 min (major isomer), 22.19 min (minor isomer)).

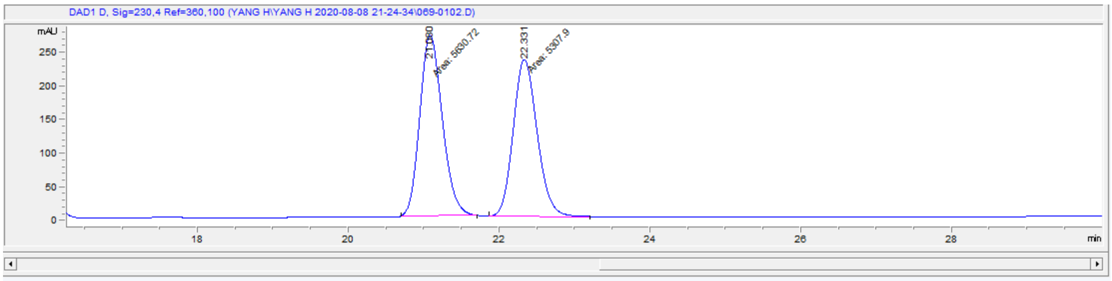

#	Time 时间	Area 峰面积	Height 峰高	Width 峰宽	Symmetry factor 对称因子	Area% 峰面积 %	Area%
1	21.08	5630.7	269.4	0.3483	0.824	51.476	51.476
2	22.331	5307.9	234.8	0.3768	0.832	48.524	48.524

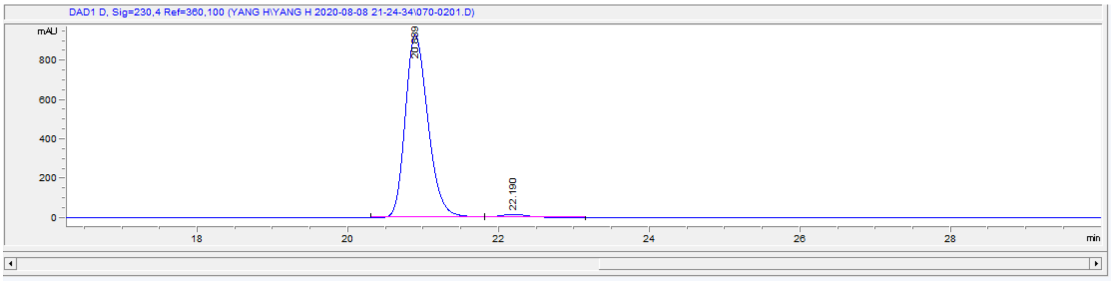

#	Time 时间	Area 峰面积	Height 峰高	Width 峰宽	Symmetry factor 对称因子	Area% 峰面积 %	Area%
1	20.889	19606.8	927.4	0.3266	0.744	97.961	97.961
2	22.19	408.1	16.3	0.3736	0.937	2.039	2.039

1.5 Recycling Experiment with MeO-PEG-BaryPhos

General procedure for Suzuki-Miyaura coupling

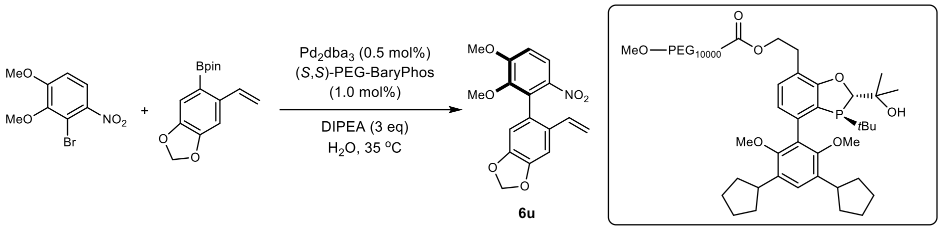

A Schlenk tube containing aryl halide (1.04 g, 4.0 mmol), arylboronic ester (1.42 g, 5.2 mmol), $Pd_2(dba)_3$ (18.3 mg, 0.020 mmol) and MeO-PEG$_{10000}$-(*S*,*S*)-BaryPhos (423.2 mg, 0.040 mmol, Pd/ligand mol ratio: 1/1) was evacuated and backfilled with N_2 for three times. Degassed DIPEA (2.10 mL, 12.0 mmol) and H_2O (10 mL) were added and the tube was sealed. The mixture was stirred at 35 ºC until aryl bromide substrate was consumed (>97% conversion by HPLC, around 15 h). The mixture was cooled to rt and degassed Et_2O (6 mL) was added. After vigorously stirring for 5 min, the mixture was allowed to settle down and the organic phase was collected with a syringe under N_2. The extraction procedure was repeated for 5 times to ensure the product was fully collected. The organic phases were combined, dried over Na_2SO_4, filtered and concentrated under reduced pressure. The crude coupling product **6u** was purified by column chromatography and the enantiomeric excess was determined by chiral HPLC (Chiralcel IC-3, 25 ºC, flow rate: 0.7 mL/min, hexanes/isopropanol: 80/20.
Another run of the asymmetric coupling reaction was conducted by adding the same amount of substrates, base and solvent to the Schlenk tube containing the aqueous phase with soluble catalyst under N_2. The above work-up procedure was repeated to determine the yield and ee of the reaction.

Supplementary Table 1 Recycling experiment

	With MeO-PEG$_{10000}$-BaryPhos					With 'free' BaryPhos
	1st	2nd	3rd	4th	5th	
yield	93%	90%	86%	82%	77%	94%
ee	94%	94%	93%	93%	89%	94%

HPLC traces of racemic sample of **6u**:

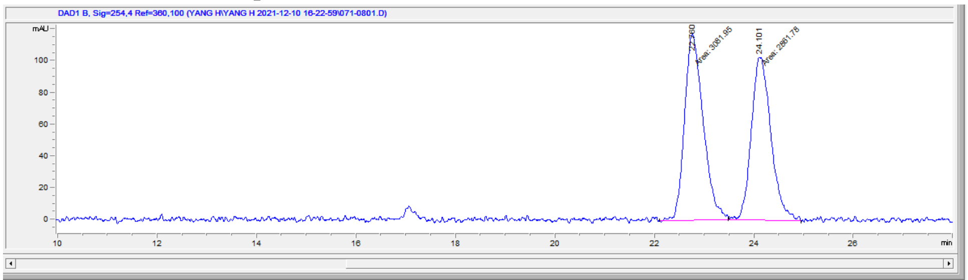

#	Time 时间	Area 峰面积	Height 峰高	Width 峰宽	Symmetry factor 对称因子	Area% 峰面积 %	Area%
1	22.76	3082	117.6	0.4366	0.726	51.852	100.000
2	24.101	2861.8	102.9	0.4633	0.666	48.148	0.000

HPLC traces of **6u** (1 st run):

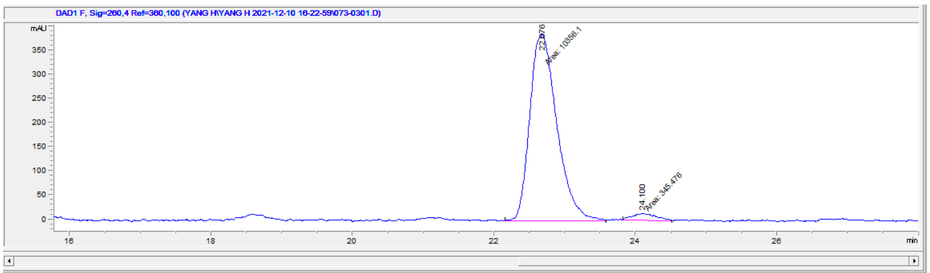

#	Time 时间	Area 峰面积	Height 峰高	Width 峰宽	Symmetry factor 对称因子	Area% 峰面积 %	Area%
1	22.676	10356.1	388.5	0.4443	0.778	96.772	100.000
2	24.1	345.5	14.2	0.4046	0.734	3.228	0.000

HPLC traces of **6u** (3 rd run):

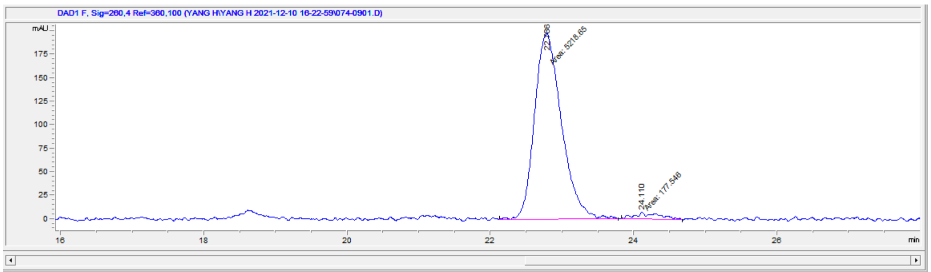

#	Time 时间	Area 峰面积	Height 峰高	Width 峰宽	Symmetry factor 对称因子	Area% 峰面积 %	Area%
1	22.786	5218.7	198.1	0.439	0.82	96.710	96.710
2	24.11	177.5	7.5	0.3969	0.464	3.290	3.290

HPLC traces of **6u** (5 th run):

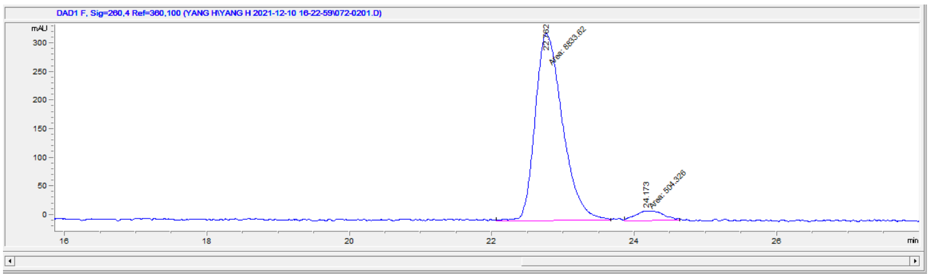

#	Time 时间	Area 峰面积	Height 峰高	Width 峰宽	Symmetry factor 对称因子	Area% 峰面积 %	Area%
1	22.762	8833.6	327.6	0.4494	0.666	94.599	0.000
2	24.173	504.3	17.7	0.4745	0.679	5.401	100.000

HPLC traces of **6u** using (*R*,*R*)-BaryPhos as ligand in Tol/H_2O (5:1) (94% ee):

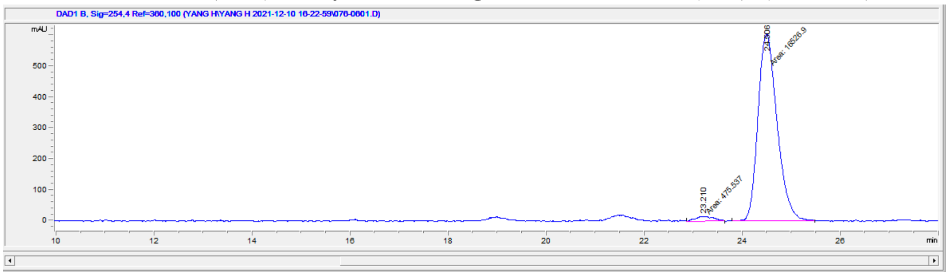

#	Time 时间	Area 峰面积	Height 峰高	Width 峰宽	Symmetry factor 对称因子	Area% 峰面积 %	Area%
1	23.21	475.5	16.9	0.4687	0.797	2.797	0.000
2	24.506	16526.9	604.8	0.4555	0.867	97.203	100.000

The recycling experiment was also studied using MeO-PEG_{2000}-BaryPhos. While comparable enantioselectivity (92% yield, 96% ee, 1 st run) was obtained, a noticeable decrease in reactivity after 2 runs was observed compared to the reactions using MeO-PEG_{10000}-BaryPhos, possibly due to the gradual loss of catalyst during the extraction process.

1.6 Comparison of Represent Chiral Ligands in Enantioselective Cross-Coupling

Several commercially available chiral ligands has been evaluated for the asymmetric synthesis of **6u** via cross-coupling reaction. Under the same reaction conditions, the preliminary results indicated that BaryPhos showed prominent catalytic property and provided the highest yield and ee value of the axially chiral biaryl product.

Supplementary Table 2 Comparison of Represent Chiral Ligands

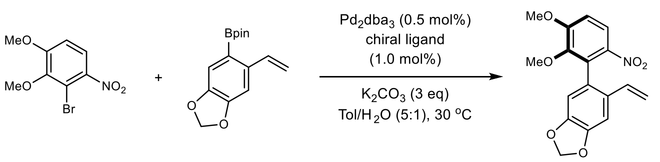

6u

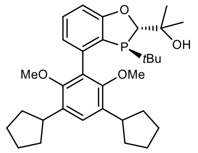	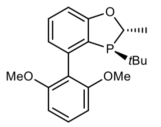	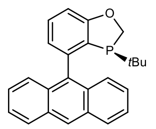	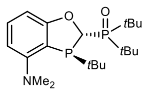
94% yield, 94% ee	79% yield, 51% ee	81% yield, 43% ee	19% yield, 33% ee
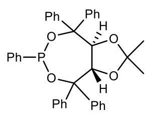	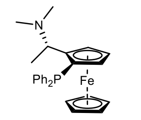	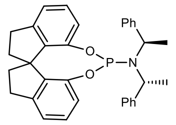	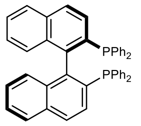
27% yield, 46% ee	low conversion, ee (n.d.)	25% yield, 52% ee	60% yield, 49% ee

1.7 Proposed stereochemical model and structure feature of substrates

Based on our results, BaryPhos is a suitable ligand for asymmetric Suzuki-Miyaura cross-coupling with aryl halide substrates containing an ortho functionality, such as nitro, sulfonyl, cyano, aldehyde, ester, and alkenyl group. The other ortho group of ary halide substrate can be an alkoxyl or alkyl group The aryl boronic acid/ester coupling partners include substituted 1-naphthaleneboronic acid/esters or related structures, ortho alkenyl-substituted aryl boronic acids/esters.

a) Suitable substrates:

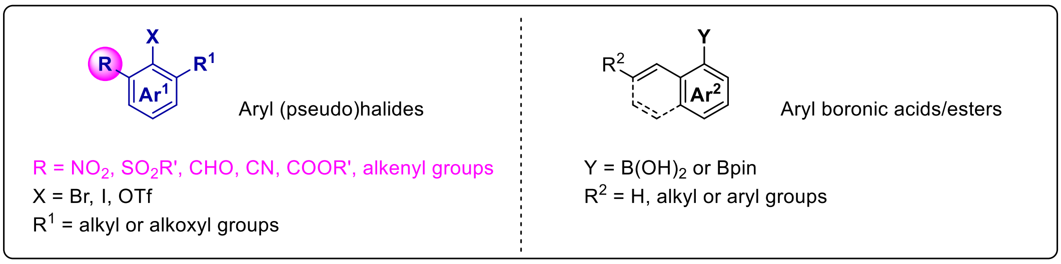

b) Stereochemical model:

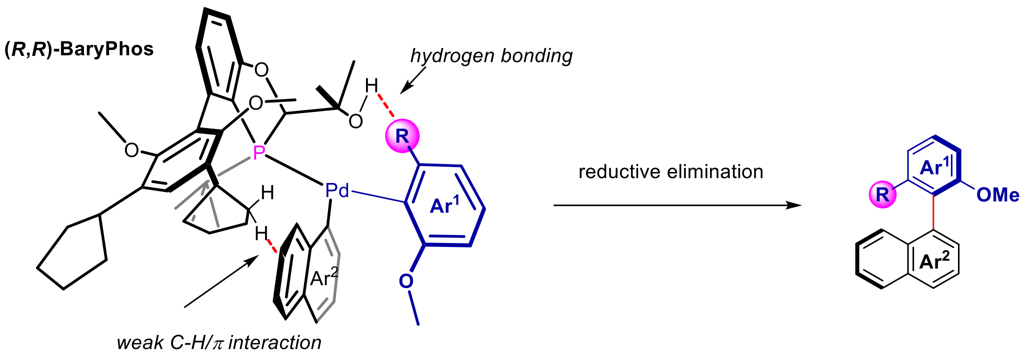

Supplementary Figure 2 a) Substrate feature. **b)** Proposed stereochemical model.

A stereochemical mode was proposed to explain the enantiocontrol of the coupling reaction and to predict the configuration of the products. It is believed that a hydrogen bonding between the tertiary alcohol of BaryPhos and the ortho functionality of aryl halide dominates the orientation of Ar^1. A C-H/π interaction between cyclopentyl group of ligand and Ar^2 exists presumably to set the conformation of this aryl coupling partner. Reduction elimination of the above intermediate delivers the chiral biaryl product with observed configuration.

1.8 Determination of racemization barrier of related products

The configurational stability of product **3d** and **3e** was studied. As demonstrated by the plots of ee as a function of time, both compounds were configurationally stable at the reaction temperatures (30 °C for **3d** and 35 °C for **3e**), as the ee values generally maintained constant within 2 days. The thermal racemization barriers of **3d** ($\Delta G^{\ddagger}$ = 27.6 kcal/mol, 60 °C) and **3e** ($\Delta G^{\ddagger}$ = 29.0 kcal/mol, 60 °C) were calculated based on the following functions.[11,12]

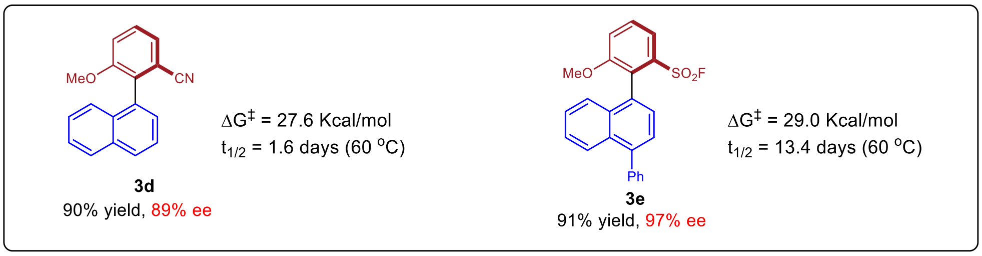

a) Determination of racemization barrier of **3d**

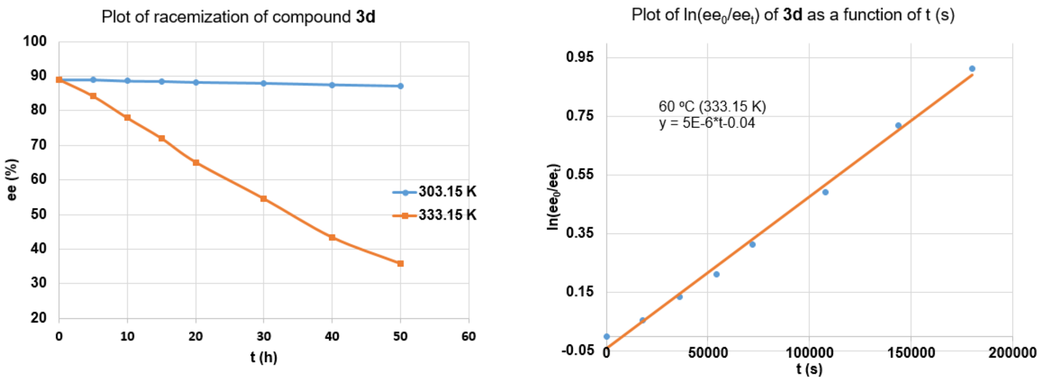

b) Determination of racemization barrier of **3e**

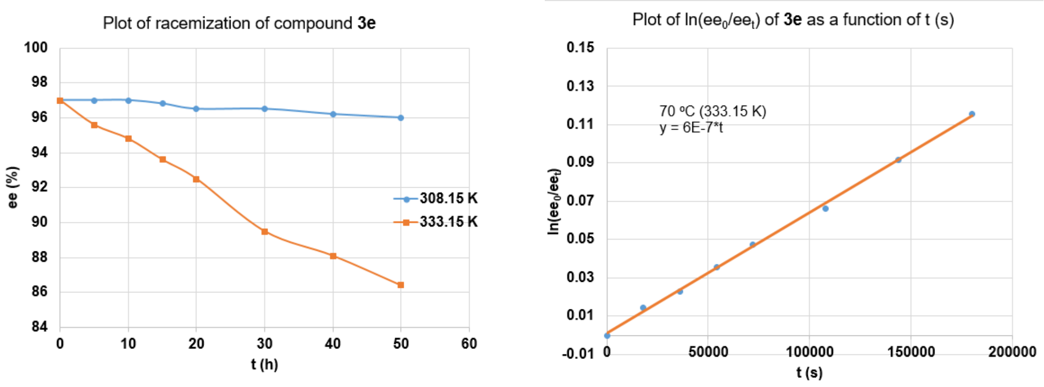

Supplementary Figure 3 Racemization experiments and data. a) Determination of racemization barrier of **3d**. **b)** Determination of racemization barrier of **3e**.

The energy ($\Delta G^{\ddagger}$) barrier and half life ($t_{1/2}$) for racemization were calculated from Eqn.1 and Eqn.2, respectively. Rate constants for racemization were obtained from Eqn.3.

Eqn.1: $\Delta G^{\ddagger} = -RT\ln(\frac{hk}{\kappa T k_B})$ (where gas constant R = 8.314 $J \cdot mol^{-1} \cdot K^{-1}$, Planck constant $h = 6.63\times10^{-34}$ J·s, transmission coefficient $\kappa = 0.5$ and Boltzmann constant $k_B = 1.38\times10^{-23}$ J/K)

Eqn.2: $t_{1/2} = 0.5\frac{\ln 2}{k}$

Eqn.3: $\ln(ee_0/ee_t) = 2k \times t$

While both products were considered to be configurationally stable under reaction conditions, the enantioselectivity for **3d** did not compete with that for **3e**, which might due to the weaker interaction between the cyano group of substrate and tertiary alcohol moiety of the ligand (than the case with sulfonyl substituted substrates), thus leading to decreased ee value of **3d**.

1.9 Transformation of Axially Chiral Biaryl Products

Synthesis of (*S*)-3,4-dimethoxy-2-(naphthalen-1-yl)aniline (6v)

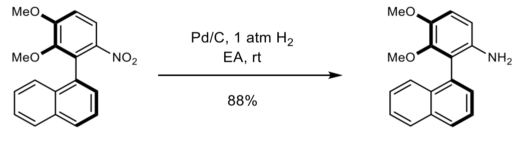

To a solution of **6o** (0.15 g, 0.49 mmol, 96% ee) in ethyl acetate (10 mL) was added 10% Pd/C (7.5 mg) and the reaction system was evacuated under reduced pressure and refilled with H_2 for three times. The mixture was stirred at rt under 1 atm H_2 for 5 h and then filtered through a layer of Celite. The catalyst was washed with EA (5 mL×3) and the filtrate was concentrated. The crude product was purified by column chromatography (eluent: EA/PE = 1/5) to afford **6v** as a colorless oil (0.12 g, 88% yield, 96% ee). $[\alpha]^{25}_{D}$ = -10.7 (*c* = 0.2, $CHCl_3$); ^1H NMR (500 MHz, $CDCl_3$) δ 7.91 (d, *J* = 7.9 Hz, 2H), 7.66-7.54 (m, 2H), 7.45 (ddd, *J* = 21.3, 15.0, 7.2 Hz, 3H), 6.91 (d, *J* = 8.7 Hz, 1H), 6.58 (d, *J* = 8.7 Hz, 1H), 3.88 (s, 3H), 3.46 (s, 3H), 2.99 (s, 2H); ^{13}C NMR (126 MHz, $CDCl_3$) δ 148.3, 145.8, 139.2, 134.0, 132.8, 132.2, 128.4, 128.2, 128.1, 126.4, 126.1, 125.9, 125.8, 121.7, 114.1, 110.4, 60.9, 56.9; HRMS (ESI) Calcd. for $C_{18}H_{17}NNaO_2$ $[M+Na]^+$: 302.1157; Found: 302.1161; Enantiomeric excess was determined by chiral HPLC (Chiralcel OD-3, 25 ºC, flow rate: 0.7 mL/min, hexanes/isopropanol: 80/20, 254 nm, 9.32 min (major isomer), 11.01 min (minor isomer)).

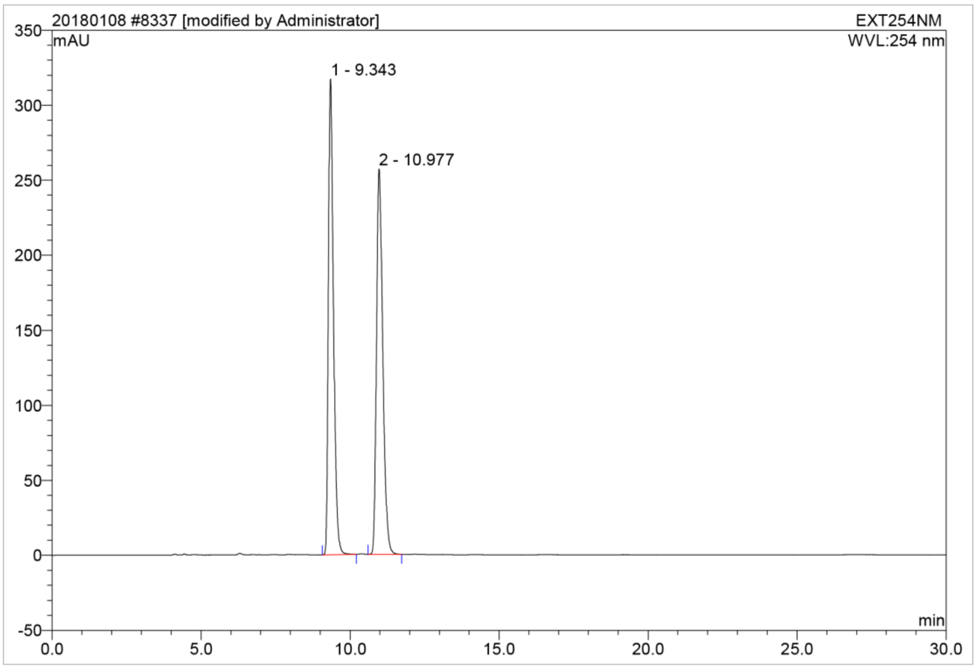

No.	Ret.Time min	Peak Name	Height mAU	Area mAU*min	Rel.Area %	Amount	Type
1	9.34	n.a.	317.145	62.860	50.02	n.a.	BMB
2	10.98	n.a.	257.135	62.804	49.98	n.a.	BMB
Total:			574.280	125.663	100.00	0.000	

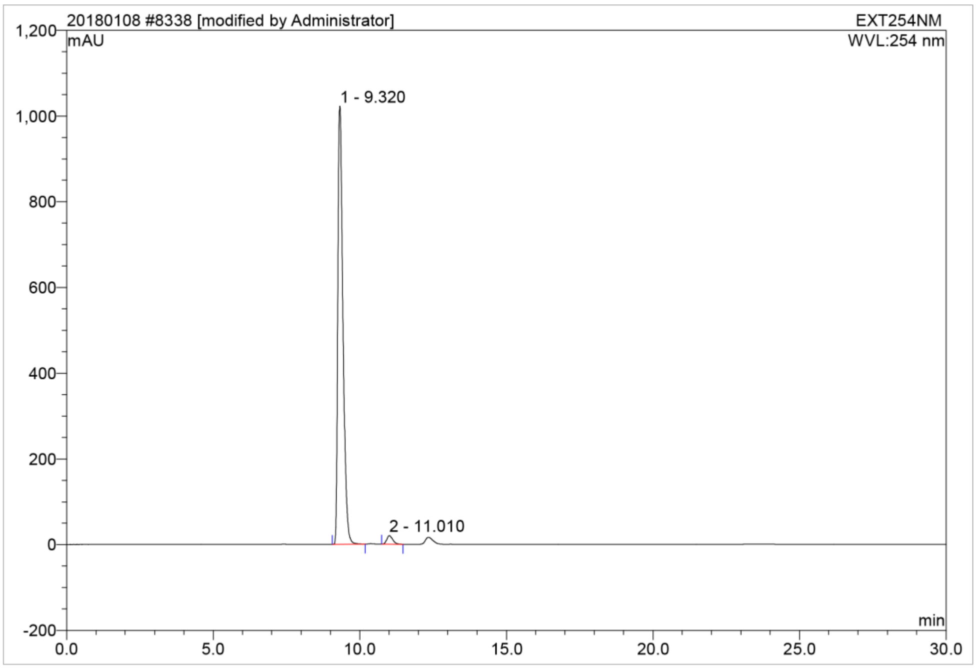

No.	Ret.Time min	Peak Name	Height mAU	Area mAU*min	Rel.Area %	Amount	Type
1	9.32	n.a.	1022.822	206.389	97.76	n.a.	BMB
2	11.01	n.a.	19.960	4.724	2.24	n.a.	BMB
Total:			1042.782	211.113	100.00	0.000	

Synthesis of (*R*)-2-(6-(2,3-dimethoxy-6-nitrophenyl)benzo[d][1,3]dioxol-5-yl)acetic acid (6w)

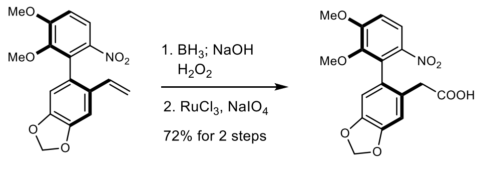

To a solution of **6u** (33 mg, 0.10 mmol) in anhydrous THF (5 mL) at 0 ºC was added a solution of BH_3·THF complex in THF (1.0 M, 0.15 mL, 0.15 mmol) and then the mixture was allowed to warm to rt and stirred for 3 h. Upon fully consumption of **6u**, the reaction was quenched by addition of H_2O (1 mL) and the mixture was cooled to 0 ºC before the addition of NaOH (12 mg, 0.30 mmol), followed by 30% H_2O_2 (60 µL, 0.50 mmol). The resulting mixture was allowed to warm to rt and stirred for 6 h. Saturated $Na_2S_2O_3$ (aq) (5 mL) was added and the mixture was extracted with EA (5 mL ×3). The organic phases were combined, dried over Na_2SO_4, filtered and concentrated under reduced pressure. The crude product was dissolved in MeCN (5 mL) and H_2O (1 mL). To the solution was added $RuCl_3$ (4 mg, 0.02 mmol) and $NaIO_4$ (86 mg, 0.40 mmol). The resulting mixture was stirred at rt for 8 h. Saturated NH_4Cl (aq) (10 mL) was added and the mixture was extracted with EA (5 mL ×3). The organic phases were combined, dried over Na_2SO_4, filtered and concentrated under reduced pressure. The crude product was purified by column chromatography (eluent: EA/PE = 1:1) to afford **6w** as a white solid (26 mg, 72% yield over 2 steps, 96% ee). $[\alpha]^{25}_{D}$ = -

49.6 (c = 0.5, $CHCl_3$); ^1H NMR (500 MHz, $CDCl_3$) δ 7.88 (dd, J = 9.1, 0.7 Hz, 1H), 7.01 (d, J = 8.7 Hz, 1H), 6.94 (s, 1H), 6.56 (s, 1H), 6.01 (d, J = 12.1 Hz, 2H), 3.99 (s, 3H), 3.57 (d, J = 0.7 Hz, 3H), 3.40 (d, J = 16.0 Hz, 1H), 3.31 (d, J = 16.1 Hz, 1H); ^{13}C NMR (151 MHz, $CDCl_3$) δ 173.5, 157.0, 148.1, 147.0, 146.8, 142.8, 130.6, 126.3, 125.9, 121.9, 111.1, 110.3, 109.1, 101.7, 61.1, 56.4, 38.7; HRMS (ESI) Calcd. for $C_{17}H_{15}NNaO_8$ $[M+Na]^+$: 384.0695; Found: 384.0699; Enantiomeric excess was determined by chiral HPLC (Chiralcel OJ-3, 25 °C, flow rate: 0.8 mL/min, hexanes/isopropanol: 80/20, 254 nm, 24.18 min (major isomer), 34.14 min (minor isomer)).

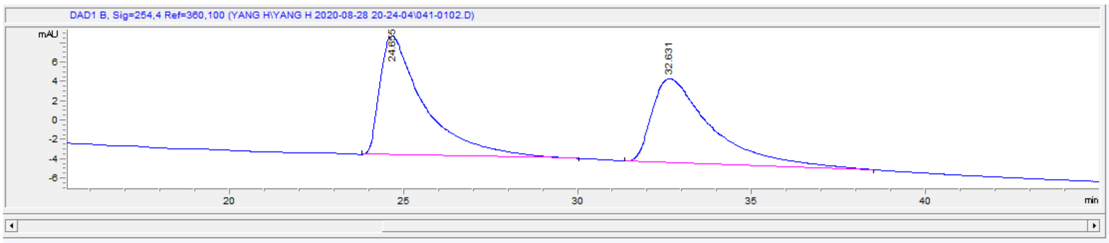

#	Time 时间	Area 峰面积	Height 峰高	Width 峰宽	Symmetry factor 对称因子	Area% 峰面积 %	Area%
1	24.655	1092.7	12.3	1.1617	0.346	51.355	51.355
2	32.631	1035	8.7	1.3993	0.405	48.645	48.645

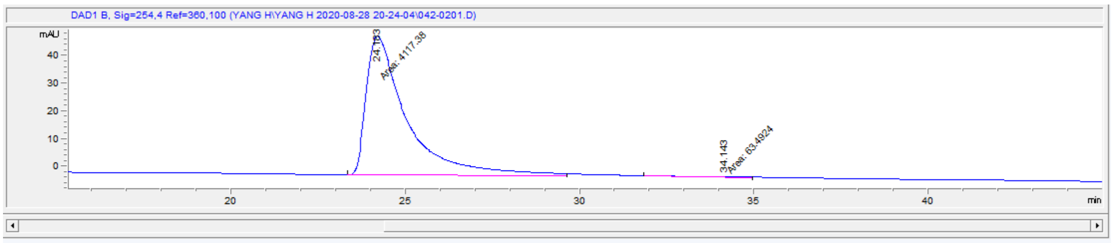

#	Time 时间	Area 峰面积	Height 峰高	Width 峰宽	Symmetry factor 对称因子	Area% 峰面积 %	Area%
1	24.183	4117.4	50.3	1.3641	0.359	98.481	98.481
2	34.143	63.5	4.3E-1	2.4339	2.046	1.519	1.519

Synthesis of (*R*)-2-(4-fluoronaphthalen-1-yl)-3-methoxy-N-(2-(4-methylpiperazin-1-yl)ethyl)benzenesulfonamide (3y)

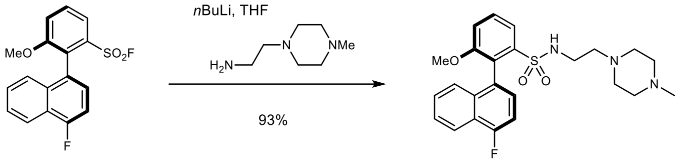

3s 3y

To a solution of 2-(4-methylpiperazin-1-yl)ethan-1-amine (0.10 mL, 0.67 mmol) in anhydrous THF (3.0 mL) at -78 °C was added *n*BuLi (2.5 M, 0.23 mL, 0.58 mmol) over 5 min. The resulting mixture was stirred at the same temperature for 30 min and then warmed up to 0 °C over 10 min. After being stirred for 30 min at 0 °C, the reaction solution was cooled to -78 °C and a solution of **3s** (0.15 g, 0.45 mmol, 96% ee) in anhydrous THF (2 mL) was added. The mixture was stirred at -78 °C for 1 h and then warmed up to 0 °C over 1 h. Upon completion, the reaction was quenched by addition of saturated NH_4Cl (10 mL).The mixture was extracted with EA (10 mL×3) and the organic phases were combined, washed with brine (10 mL), dried over Na_2SO_4, filtered and concentrated under reduced pressure. The crude product was purified by column chromatography (eluent: MeOH:EA = 1:4) to afford **3y** as a colorless oil (0.19 g, 93%

yield, 92% ee). $[\alpha]^{25}_{D}$ = -17.5 (*c* = 0.5, $CHCl_3$); ^{1}H NMR (600 MHz, $CDCl_3$) δ 8.16 (d, *J* = 8.4 Hz, 1H), 7.86 (d, *J* = 8.0 Hz, 1H), 7.57 (t, *J* = 8.2 Hz, 1H), 7.55-7.50 (m, 1H), 7.46 (dd, *J* = 7.8, 5.4 Hz, 1H), 7.42 (dd, *J* = 14.1, 6.2 Hz, 1H), 7.31-7.26 (m, 2H), 7.24 (d, *J* = 7.9 Hz, 2H), 3.93 (s, 1H), 3.63 (s, 3H), 2.74 (d, *J* = 5.4 Hz, 1H), 2.53 (s, 1H), 2.39-2.13 (m, 6H), 2.07-1.93 (m, 3H), 1.85 (ddd, *J* = 12.3, 7.0, 5.1 Hz, 1H), 1.38-1.26 (m, 2H), 0.88 (t, *J* = 6.9 Hz, 1H); ^{13}C NMR (151 MHz, $CDCl_3$) δ 158.9 (d, *J* = 253.3 Hz), 158.7, 141.4, 133.9 (d, *J* = 4.7 Hz), 129.4, 128.1 (d, *J* = 5.1 Hz), 127.1, 126.7, 126.2, 125.7, 123.7 (d, *J* = 16.4 Hz), 121.7, 121.1 (d, *J* = 5.4 Hz), 115.2, 109.3, 109.2; ^{19}F NMR (376 MHz, $CDCl_3$) δ -103.4; HRMS (ESI) Calcd. for $C_{24}H_{29}FN_3O_3S$ $[M+H]^+$: 458.1914; Found: 458.1919; Enantiomeric excess was determined by chiral HPLC (Chiralcel AD-3, 25 °C, flow rate: 0.8 mL/min, hexanes/isopropanol: 80/20, 290 nm, 23.62 min (major isomer), 27.65 min (minor isomer)).

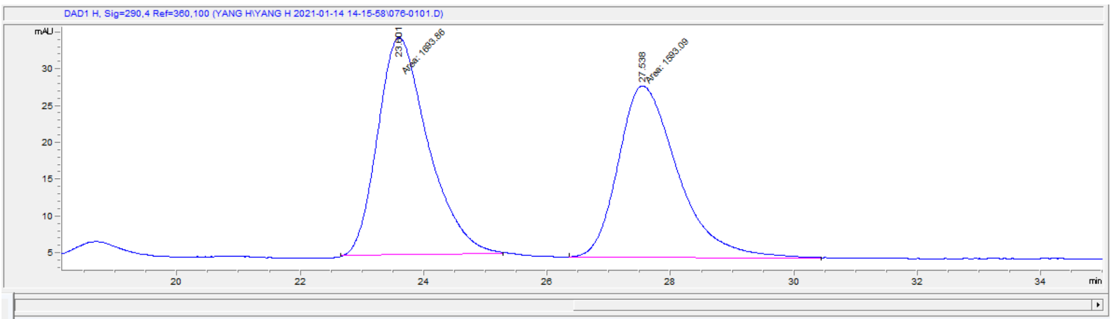

#	Time 时间	Area 峰面积	Height 峰高	Width 峰宽	Symmetry factor 对称因子	Area% 峰面积 %	Area%
1	23.601	1693.9	29.6	0.953	0.724	51.533	51.533
2	27.538	1593.1	23.4	1.1331	0.653	48.467	48.467

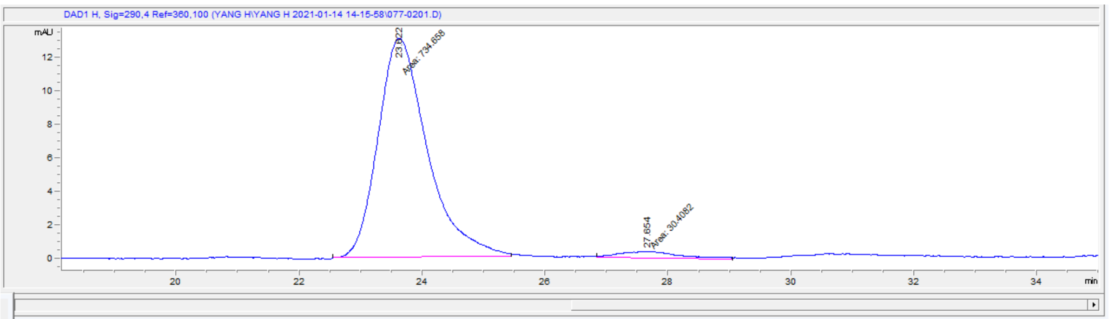

#	Time 时间	Area 峰面积	Height 峰高	Width 峰宽	Symmetry factor 对称因子	Area% 峰面积 %	Area%
1	23.622	734.7	13.1	0.9344	0.724	96.025	96.025
2	27.654	30.4	4.2E-1	1.2157	0.628	3.975	3.975

Synthesis of (*S*)-3,4-dimethoxy-2-(naphthalen-1-yl)aniline (3v)

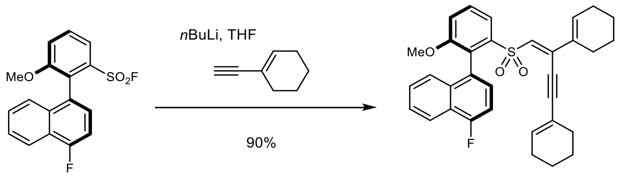

3s **3v**

To a solution of 1-ethynylcyclohex-1-ene (0.10 mL, 0.85 mmol) in anhydrous THF (5.0 mL) at -78 °C was added *n*BuLi (2.5 M, 0.28 mL, 0.71 mmol) over 10 min. The resulting mixture was stirred at the same temperature for 1 h and then transferred to a pre-cooled solution of **3s** (95 mg, 0.28 mmol, 96% ee) in anhydrous THF (2 mL) at -78 °C. The mixture was warmed up to 0 °C over 1 h. Upon completion, the reaction was

quenched by addition of saturated NH_4Cl (10 mL). The mixture was extracted with EA (10 mL×3) and the organic phases were combined, washed with brine (10 mL), dried over Na_2SO_4, filtered and concentrated under reduced pressure. The crude product was purified by column chromatography (eluent: EA:PE = 1:10) to afford **3v** as a colorless oil (132 mg, 90% yield, 91% ee). $[\alpha]^{25}_{D}$ = -94.0 (c = 0.5, $CHCl_3$); ^{1}H NMR (500 MHz, $CDCl_3$) δ 8.11-8.03 (m, 2H), 7.59 (t, J = 8.2 Hz, 1H), 7.48 (dd, J = 7.9, 5.5 Hz, 1H), 7.43 (t, J = 7.6 Hz, 1H), 7.23 (dd, J = 9.0, 6.3 Hz, 3H), 7.14 (d, J = 8.5 Hz, 1H), 6.22 (t, J = 3.9 Hz, 1H), 6.15-6.07 (m, 1H), 5.22 (s, 1H), 3.62 (s, 3H), 2.18-1.95 (m, 6H), 1.70-1.59 (m, 4H), 1.40-1.26 (m, 5H), 0.58 (d, J = 14.3 Hz, 1H); ^{13}C NMR (151 MHz, $CDCl_3$) δ 158.9 (d, J = 253.1 Hz), 158.5, 143.8, 137.7, 137.2, 136.8, 134.2 (d, J = 4.6 Hz), 133.0, 130.0 (d, J = 8.3 Hz), 128.8, 128.1, 127.6 (d, J = 4.5 Hz), 127.2), 127.1, 126.2 (d, J = 2.5 Hz), 125.9, 123.5 (d, J = 16.4 Hz), 121.3, 120.6 (d, J = 5.4 Hz), 120.5, 115.2, 109.2, 109.0, 106.5, 80.0, 56.5, 28.6, 26.4, 26.0, 23.9, 22.3, 22.1, 21.5, 21.4; ^{19}F NMR (376 MHz, $CDCl_3$) δ -122.8; HRMS (EI) Calcd. for $C_{33}H_{31}FO_3S$ $[M]^+$: 526.1978; Found: 526.1973; Enantiomeric excess was determined by chiral HPLC (Chiralcel AD-3, 25 ºC, flow rate: 0.8 mL/min, hexanes/isopropanol: 80/20, 290 nm, 10.73 min (major isomer), 14.89 min (minor isomer)).

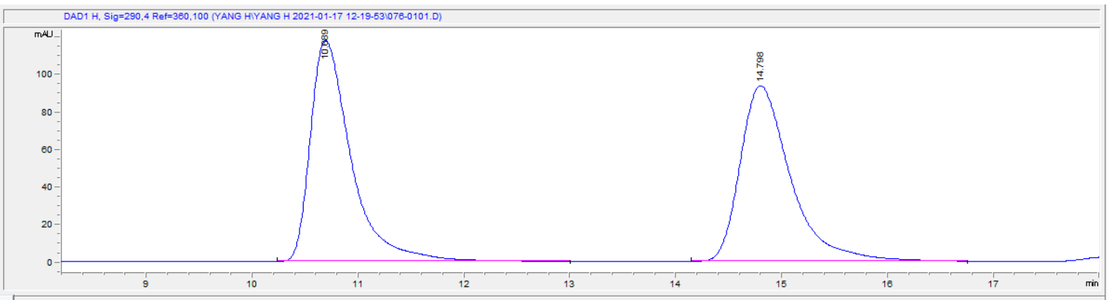

#	Time 时间	Area 峰面积	Height 峰高	Width 峰宽	Symmetry factor 对称因子	Area% 峰面积 %	Area%
1	10.689	3149.2	117.8	0.406	0.538	50.402	50.402
2	14.798	3099	93.6	0.5064	0.623	49.598	49.598

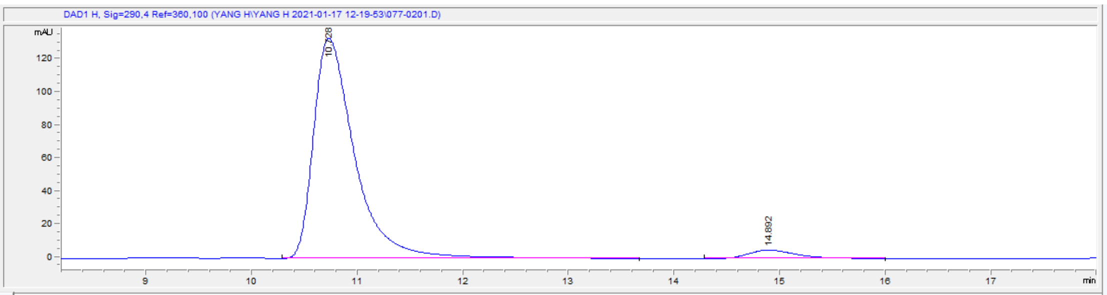

#	Time 时间	Area 峰面积	Height 峰高	Width 峰宽	Symmetry factor 对称因子	Area% 峰面积 %	Area%
1	10.728	3629.2	133.4	0.4134	0.531	95.685	95.685
2	14.892	163.7	5.1	0.4494	0.685	4.315	4.315

Synthesis of 2,2,2-trifluoroethyl (*R*)-2-(4-fluoronaphthalen-1-yl)-3-methoxy-Benzenesulfonate (3u)

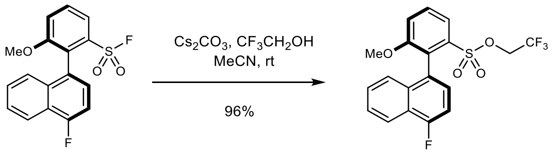

3s **3u**

To a solution of **3s** (20 mg, 0.06 mmol, 96% ee) in MeCN (2 mL) was added Cs_2CO_3 (39 mg, 0.12 mmol) and trifluoroethanol (23 μL, 0.30 mmol). The mixture was stirred at rt for 12 h before being partitioned between EA (5 mL) and H_2O (5 mL). The organic phase was separated and the aqueous phase was extracted with EA (5 mL×2). The organic phases were combined, washed with brine (10 mL), dried over Na_2SO_4, filtered and concentrated under reduced pressure. The crude product was purified by column chromatography (eluent: EA:PE = 1:6) to afford **3u** as a colorless oil (24 mg, 96% yield, 88% ee). $[\alpha]^{25}_{D}$ = -120.6 (*c* = 0.4, $CHCl_3$); ^1H NMR (500 MHz, $CDCl_3$) δ 8.17 (d, *J* = 8.4 Hz, 1H), 7.85 (d, *J* = 8.1 Hz, 1H), 7.64 (t, *J* = 8.2 Hz, 1H), 7.54 (t, *J* = 7.6 Hz, 1H), 7.42 (t, *J* = 7.7 Hz, 1H), 7.35 (d, *J* = 8.4 Hz, 1H), 7.31 (dd, *J* = 7.9, 5.4 Hz, 1H), 7.22 (dd, *J* = 10.1, 8.0 Hz, 2H), 3.93 (dq, *J* = 12.1, 8.0 Hz, 1H), 3.73-3.59 (m, 4H); ^{13}C NMR (151 MHz, $CDCl_3$) δ 159.2, 159.1 (d, *J* = 253.5 Hz), 136.9, 133.9 (d, *J* = 4.8 Hz), 129.8, 128.7, 128.1 (d, *J* = 8.4 Hz), 127.3, 127.1 (d, *J* = 4.7 Hz), 126.2, 125.4 (d, *J* = 2.6 Hz), 123.7, 123.6, 121.9, 121.8 (dd, *J* = 555.9, 278.0 Hz), 121.0 (d, *J* = 5.4 Hz), 116.9, 108.9 (d, *J* = 20.2 Hz), 63.8 (q, *J* = 38.2 Hz), 56.6; ^{19}F NMR (376 MHz, acetone-d_6) δ -75.0 (t, *J* = 7.5 Hz), -123.2; HRMS (ESI) Calcd. for $C_{19}H_{15}F_4O_4S$ $[M+H]^+$: 415.0627; Found: 415.0630; Enantiomeric excess was determined by chiral HPLC (Chiralcel IC-3, 25 ºC, flow rate: 0.8 mL/min, hexanes/isopropanol: 80/20, 260 nm, 8.93 min (minor isomer), 9.79 min (major isomer)).

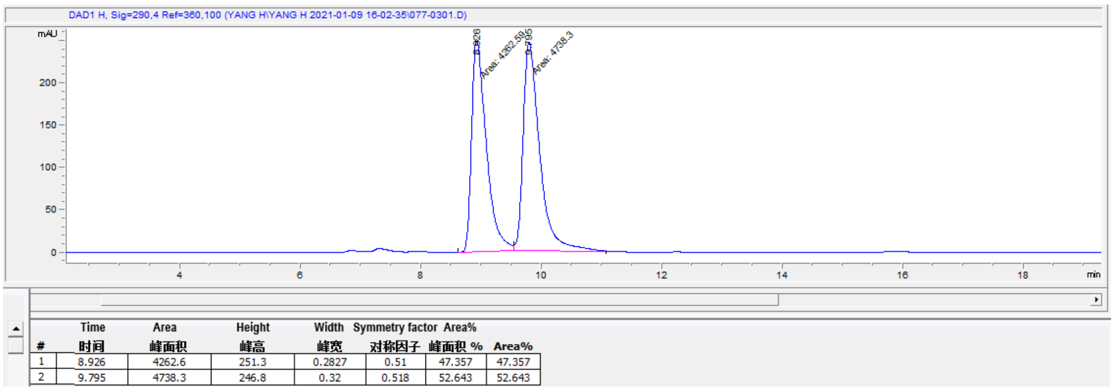

#	Time 时间	Area 峰面积	Height 峰高	Width 峰宽	Symmetry factor 对称因子	Area% 峰面积 %	Area%
1	8.926	4262.6	251.3	0.2827	0.51	47.357	47.357
2	9.795	4738.3	246.8	0.32	0.518	52.643	52.643

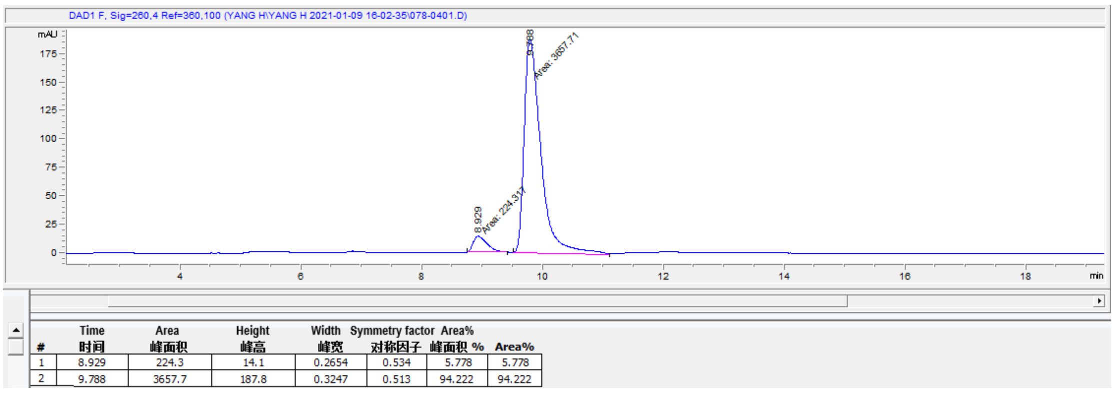

#	Time 时间	Area 峰面积	Height 峰高	Width 峰宽	Symmetry factor 对称因子	Area% 峰面积 %	Area%
1	8.929	224.3	14.1	0.2654	0.534	5.778	5.778
2	9.788	3657.7	187.8	0.3247	0.513	94.222	94.222

Synthesis of (*R*)-2-(4-fluoronaphthalen-1-yl)-3-methoxybenzenesulfonic acid (3t)

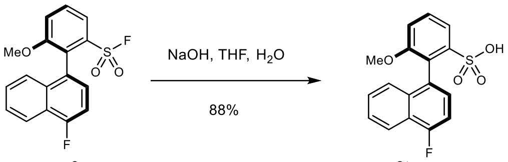

3s **3t**

To a solution of **3s** (20 mg, 0.06 mmol, 96% ee) in THF (4 mL) and H_2O (2 mL) was added NaOH (5.0 mg, 0.12 mmol) and the mixture was stirred at rt for 12 h. Upon full conversion of **3s**, the system was neutralized with 1 N HCl (2 mL) and the mixture was extracted with EA (5 mL×3). The organic phases were combined, dried over Na_2SO_4, filtered and concentrated under reduced pressure. The crude product was purified by column chromatography (eluent: MeOH:EA = 1:20) to afford **3t** as a colorless oil (18 mg, 88% yield, 90% ee). $[\alpha]^{25}_{D}$ = -43.9 (c = 0.5, $CHCl_3$); ^{1}H NMR (600 MHz, MeOD-d_4) δ 8.04 (d, J = 8.4 Hz, 1H), 7.80 (dd, J = 8.0, 0.9 Hz, 1H), 7.50 (t, J = 8.1 Hz, 1H), 7.45 (ddd, J = 8.2, 6.3, 1.6 Hz, 1H), 7.36-7.28 (m, 3H), 7.23-7.19 (m, 1H), 7.14 (dd, J = 10.8, 7.9 Hz, 1H), 3.57 (s, 3H); ^{13}C NMR (151 MHz, $CDCl_3$) δ 159.7, 159.5 (d, J = 248.6 Hz), 146.7, 135.8 (d, J = 4.7 Hz), 131.8 (d, J = 4.4 Hz), 129.6, 129.0 (d, J = 8.2 Hz), 128.0, 127.8 (d, J = 2.7 Hz), 127.1, 126.4, 124.5 (d, J = 16.4 Hz), 121.1, 120.8 (d, J = 5.6 Hz), 114.0, 109.5 (d, J = 20.0 Hz), 56.4; ^{19}F NMR (376 MHz, $CDCl_3$) δ -126.6; HRMS (EI) Calcd. for $C_{17}H_{13}FO_4S$ $[M]^+$: 332.0519; Found: 332.0511; Enantiomeric excess was determined by chiral HPLC (Chiralcel SB, 25 ºC, flow rate: 0.8 mL/min, hexanes/isopropanol: 70/30, 290 nm, 5.65 min (major isomer), 9.31 min (minor isomer)).

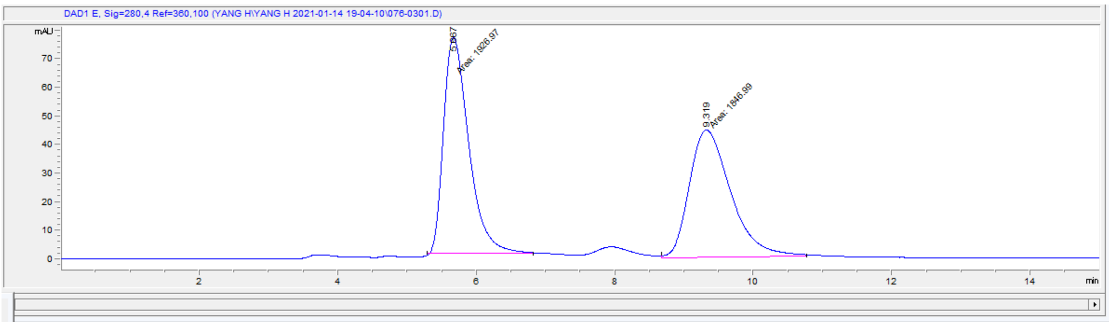

#	Time 时间	Area 峰面积	Height 峰高	Width 峰宽	Symmetry factor 对称因子	Area% 峰面积 %	Area%
1	5.667	1927	75.8	0.4236	0.617	51.060	51.060
2	9.319	1847	44.5	0.6911	0.677	48.940	48.940

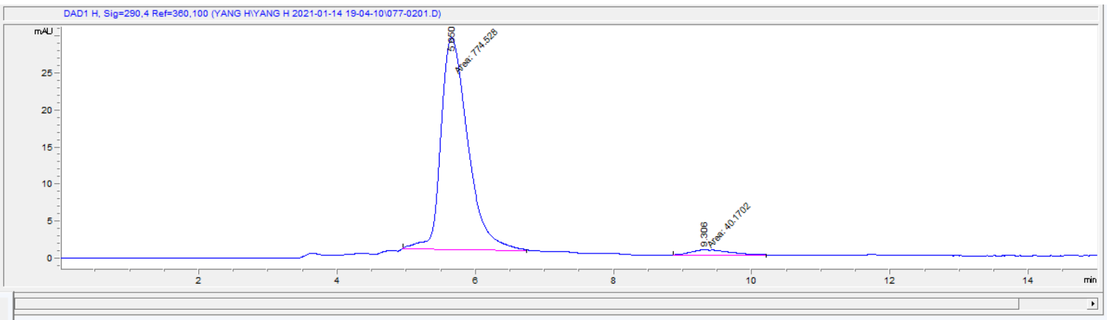

#	Time 时间	Area 峰面积	Height 峰高	Width 峰宽	Symmetry factor 对称因子	Area% 峰面积 %	Area%
1	5.65	774.5	28.7	0.4503	0.636	95.069	95.069
2	9.306	40.2	8.5E-1	0.7912	0.5	4.931	4.931

Synthesis of (*R*)-1-((3-methoxy-2-(pyren-1-yl)phenyl)sulfonyl)pyrene (*M*-17)

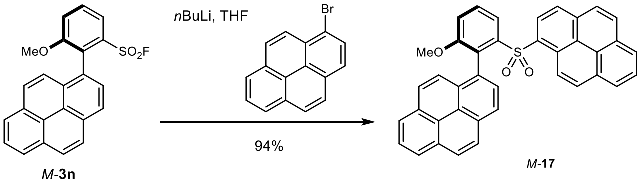

To a solution of 1-bromopyrene (0.72 g, 2.56 mmol) in anhydrous THF (10 mL) at -78 °C was added *n*BuLi (1.6 M, 1.60 mL, 2.56 mmol) over 10 min. The resulting mixture was stirred at the same temperature for 1 h and then transferred to a pre-cooled solution of **3n** (0.60 g, 1.54 mmol, 96% ee) in anhydrous THF (5 mL) at -78 °C. The mixture was warmed up to rt over 1 h and then stirred for 3 h. Upon completion, the reaction was quenched by addition of saturated NH_4Cl (10 mL). The mixture was extracted with EA (10 mL×3) and the organic phases were combined, washed with brine (10 mL), dried over Na_2SO_4, filtered and concentrated under reduced pressure. The crude product was purified by column chromatography (eluent: EA:PE = 1:5) to afford **17** as a gray solid (1.37 g, 94% yield, 96% ee). $[\alpha]^{25}_D$ = 62.5 (*c* = 0.2, $CHCl_3$); ^{1}H NMR (500 MHz, $CDCl_3$) δ 8.59 (d, *J* = 7.4 Hz, 1H), 8.45 (d, *J* = 9.3 Hz, 1H), 8.25-8.18 (m, 1H), 8.15 (d, *J* = 7.8 Hz, 1H), 8.04 (ddd, *J* = 13.3, 6.9, 4.6 Hz, 6H), 7.97 (d, *J* = 7.7 Hz, 1H), 7.86 (t, *J* = 8.3 Hz, 1H), 7.67 (t, *J* = 7.6 Hz, 1H), 7.3-7.27 (m, 2H), 7.20 (d, *J* = 8.9 Hz, 1H), 6.84 (d, *J* = 7.5 Hz, 1H), 6.45 (dd, *J* = 15.9, 8.5 Hz, 2H), 5.97 (d, *J* = 9.0 Hz, 1H), 5.64 (d, *J* = 9.1 Hz, 1H), 3.47 (s, 3H); ^{13}C NMR (151 MHz, $CDCl_3$) δ 158.5, 144.8, 133.2, 131.2, 130.6, 130.6, 130.0, 129.9, 129.6, 129.6, 129.4, 128.8, 128.6, 128.5, 127.8, 127.6, 127.2, 126.6, 126.4, 126.3, 125.6, 125.4, 125.2, 125.1, 124.8, 124.7, 123.8, 123.6, 123.6, 123.5, 123.3, 122.8, 122.2, 122.1, 120.1, 115.5, 56.3; HRMS (ESI) Calcd. for $C_{39}H_{25}NO_3S$ $[M+H]^+$: 573.1519; Found: 573.1521; Enantiomeric excess was determined by chiral HPLC (Chiralcel AD-3, 25 °C, flow rate: 0.8 mL/min, hexanes/isopropanol: 80/20, 254 nm, 15.21 min (major isomer), 16.46 min (minor isomer)).

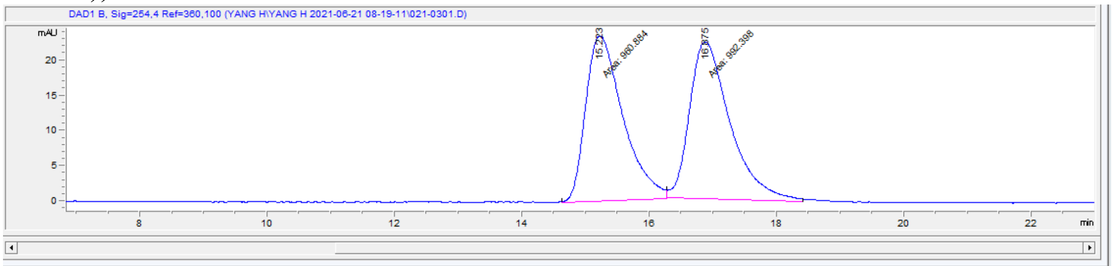

#	Time 时间	Area 峰面积	Height 峰高	Width 峰宽	Symmetry factor 对称因子	Area% 峰面积 %	Area%
1	15.223	960.9	23.5	0.682	0.596	49.193	49.193
2	16.875	992.4	22.3	0.7426	0.594	50.807	50.807

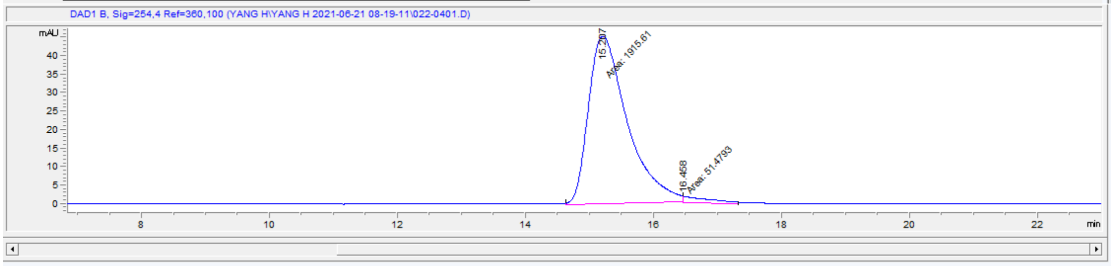

#	Time 时间	Area 峰面积	Height 峰高	Width 峰宽	Symmetry factor 对称因子	Area% 峰面积 %	Area%
1	15.207	1915.6	45.3	0.7043	0.576	97.383	97.383
2	16.458	51.5	1.8	0.4781	0	2.617	2.617

Synthesis of (*R*)-1-(2-((3,4-dimethoxyphenyl)sulfonyl)-6-methoxyphenyl)-4-fluoronaphthalene (3w)

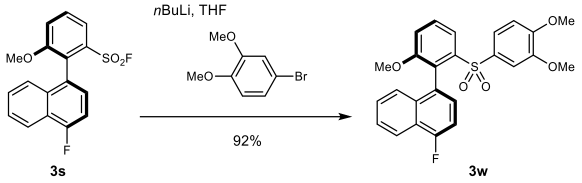

3w was synthesized following a similar procedure to that of **17** and was obtained in 92% yield, 91% ee). $[\alpha]^{25}_{D}$ = -16.3 (*c* = 0.2, $CHCl_3$); ^1H NMR (400 MHz, $CDCl_3$) δ 8.18 (d, *J* = 8.1 Hz, 1H), 7.98 (d, *J* = 8.3 Hz, 1H), 7.66 (t, *J* = 8.2 Hz, 1H), 7.36-7.30 (m, 2H), 7.25-7.16 (m, 2H), 7.04 (t, *J* = 7.6 Hz, 1H), 6.63 (d, *J* = 8.3 Hz, 1H), 6.58 (dd, *J* = 8.5, 1.7 Hz, 1H), 6.41 (d, *J* = 1.8 Hz, 1H), 6.17 (d, *J* = 8.5 Hz, 1H), 3.66 (s, 3H), 3.62 (s, 3H), 3.58 (s, 3H); ^{13}C NMR (151 MHz, $CDCl_3$) δ 159.0 (d, *J* = 252.9 Hz), 158.7, 152.0, 147.8, 143.2, 133.4 (d, *J* = 4.8 Hz), 131.4, 129.9 (d, *J* = 8.3 Hz), 129.4, 127.6, 126.8 (d, *J* = 4.3 Hz), 126.2, 125.6, 125.0 (d, *J* = 2.5 Hz), 123.1 (d, *J* = 16.2 Hz), 121.9, 120.5, 120.2 (d, *J* = 5.4 Hz), 115.7, 109.7 (d, *J* = 43.8 Hz), 108.9 (d, *J* = 19.9 Hz), 56.4, 55.9 55.8; ^{19}F NMR (376 MHz, $CDCl_3$) δ -122.8; HRMS (ESI) Calcd. for $C_{25}H_{22}FO_5S$ $[M+H]^+$: 453.1166; Found: 453.1162; Enantiomeric excess was determined by chiral HPLC (Chiralcel AD-3, 25 ºC, flow rate: 0.8 mL/min, hexanes/isopropanol: 80/20, 290 nm, 21.63 min (major isomer), 32.44 min (minor isomer)).

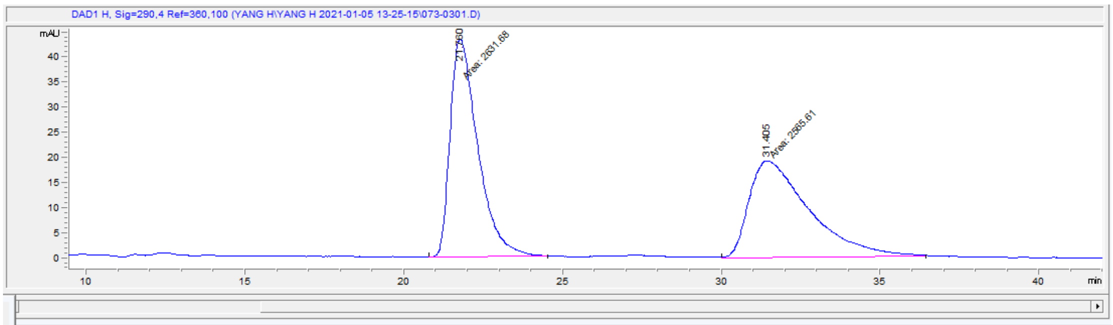

#	Time 时间	Area 峰面积	Height 峰高	Width 峰宽	Symmetry factor 对称因子	Area% 峰面积 %	Area%
1	21.76	2631.7	43.4	1.0107	0.55	50.636	50.636
2	31.405	2565.6	19.3	2.2122	0.404	49.364	49.364

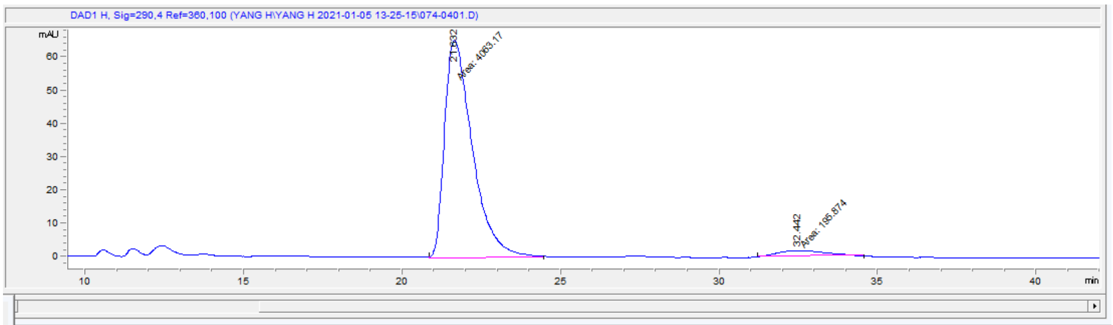

#	Time 时间	Area 峰面积	Height 峰高	Width 峰宽	Symmetry factor 对称因子	Area% 峰面积 %	Area%
1	21.632	4063.2	65.8	1.0292	0.516	95.401	95.401
2	32.442	195.9	1.8	1.8142	0.675	4.599	4.599

Synthesis of (*R*)-1-fluoro-4-(2-(hexylsulfonyl)-6-methoxyphenyl)naphthalene (3x)

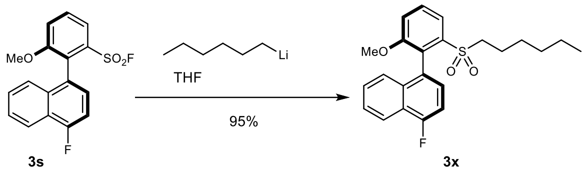

3x was synthesized by reacting **3s** with *n*-hexyllithium and was obtained in 95% yield, 92% ee). $[\alpha]^{25}_{D}$ = -27.9 (c = 0.5, $CHCl_3$); ^1H NMR (400 MHz, $CDCl_3$) δ 8.18 (d, J = 8.4 Hz, 1H), 7.91 (dd, J = 8.0, 0.9 Hz, 1H), 7.63 (t, J = 8.2 Hz, 1H), 7.57-7.50 (m, 1H), 7.46 (dd, J = 7.9, 5.4 Hz, 1H), 7.42 (ddd, J = 8.2, 6.9, 1.1 Hz, 1H), 7.32-7.28 (m, 1H), 7.28-7.22 (m, 2H), 3.65 (d, J = 6.5 Hz, 3H), 2.46 (ddd, J = 14.0, 11.7, 5.0 Hz, 1H), 2.15 (ddd, J = 14.0, 11.5, 4.7 Hz, 1H), 1.49-1.36 (m, 1H), 1.30-1.18 (m, 1H), 1.15-1.02 (m, 2H), 0.97-0.80 (m, 3H), 0.76 (t, J = 7.3 Hz, 3H), 0.72-0.60 (m, 1H).; ^{13}C NMR (151 MHz, $CDCl_3$) δ 159.1 (d, J = 253.7 Hz), 158.8, 140.4, 133.9 (d, J = 4.7 Hz), 129.7, 129.6, 127.7, 127.3, 126.8 (d, J = 4.6 Hz), 126.2 (d, J = 1.3 Hz), 125.3 (d, J = 2.5 Hz), 123.6 (d, J = 16.4 Hz), 121.8, 121.2 (d, J = 5.4 Hz), 116.0, 109.1 (d, J = 20.1 Hz), 56.5, 54.8, 31.0, 27.6, 22.7, 22.3, 14.0; ^{19}F NMR (376 MHz, $CDCl_3$) δ -122.0; HRMS (ESI) Calcd. for $C_{23}H_{26}FO_3S$ $[M+H]^+$: 401.1581; Found: 401.1583; Enantiomeric excess was determined by chiral HPLC (Chiralcel OD-3, 25 °C, flow rate: 0.8 mL/min, hexanes/isopropanol: 80/20, 260 nm, 20.22 min (major isomer), 21.59 min (minor isomer)).

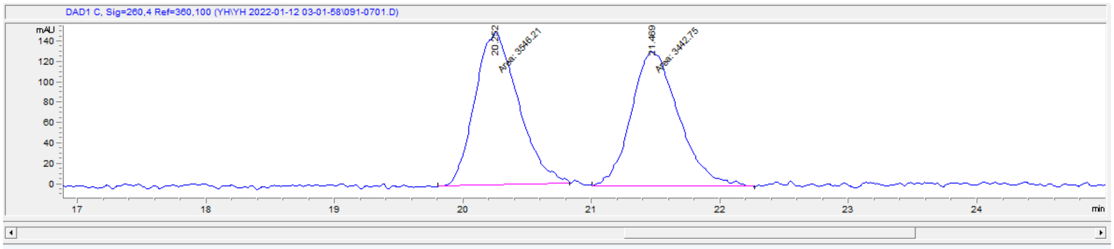

#	Time	Area	Height	Width	Area%	Symmetry
1	20.252	3546.2	149.5	0.3953	50.740	0.871
2	21.469	3442.7	132.6	0.4326	49.260	0.759

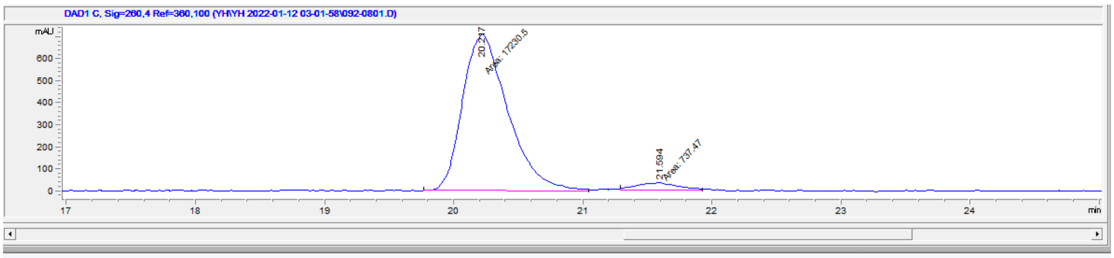

#	Time	Area	Height	Width	Area%	Symmetry
1	20.217	17230.5	709.4	0.4048	95.896	0.719
2	21.594	737.5	33	0.3727	4.104	1.235

Synthesis of 1,6-bis((*E*)-4-(((*R*)-3-methoxy-2-(pyren-1-yl)phenyl)sulfonyl)styryl)-Pyrene (*M*,*M*-18)

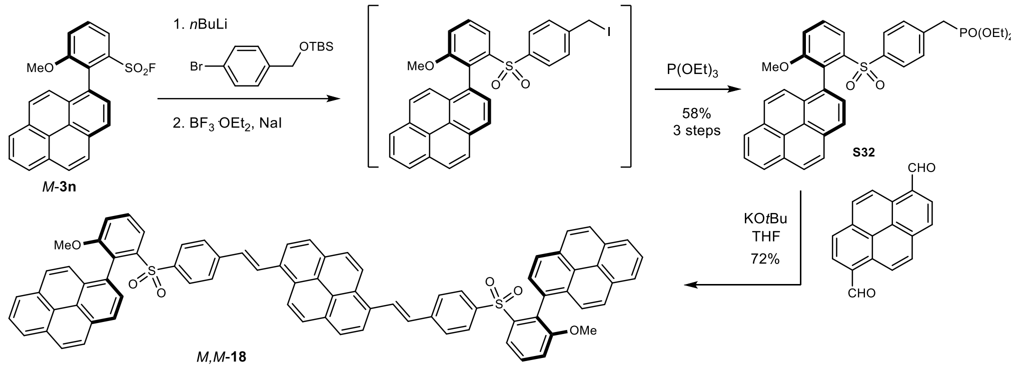

To a solution of ((4-bromobenzyl)oxy)(tert-butyl)dimethylsilane (1.50 g, 4.98 mmol) in anhydrous THF (20 mL) at -78 °C was added *n*BuLi (1.6 M, 2.85 mL, 4.57 mmol) over 10 min. The resulting mixture was stirred at the same temperature for 1 h and then transferred to a pre-cooled solution of **3n** (1.62 g, 4.15 mmol) in anhydrous THF (15 mL) at -78 °C. The mixture was warmed up to rt over 1 h and then stirred for 2 h. Upon completion, the reaction was quenched by addition of saturated NH_4Cl (10 mL). The mixture was extracted with EA (10 mL×3) and the organic phases were combined, washed with brine (10 mL), dried over Na_2SO_4, filtered and concentrated under reduced pressure.

The above intermediate was dissolved in anhydrous MeCN (15 mL) at 0 °C was added NaI (2.24 g, 14.94 mmol), followed by BF_3 • Et_2O (46.5 wt%, 1.90 mL, 14.94 mmol). The mixture was stirred at 0 °C for 5 h and then the reaction was quenched by slow addition of H_2O (5 mL). The mixture was concentrated under reduced pressure and the residue was dissolved in EA (30 mL) and H_2O (10 mL). The organic phase was separated and washed with brine (10 mL). The organic layer was dried over Na_2SO_4, filtered and concentrated to provide iodide intermediate as a brown oil.

The crude iodide intermediate was dissolved in THF (5 mL), followed by addition of $P(OEt)_3$ (4.30 mL, 24.90 mmol). The resulting mixture was stirred at rt for 20 h and quenched by addition of H_2O (15 mL). The mixture was extracted with EA (15 mL×3). The organic phases were combined, dried over Na_2SO_4, filtered and concentrated under reduced pressure. The crude product was purified by column chromatography (eluent: EA/PE = 1:1) to afford **S32** as a white foam (1.72 g, 58% yield over 3 steps).

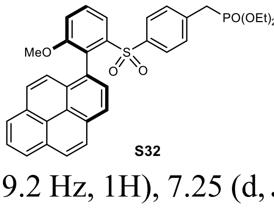

$[\alpha]^{25}_D$ = -15.8 (*c* = 1.0, $CHCl_3$); ^{1}H NMR (400 MHz, $CDCl_3$) δ 8.22 (d, *J* = 8.0 Hz, 1H), 8.17 (dd, *J* = 7.4, 4.9 Hz, 2H), 8.10-8.03 (m, 2H), 8.02 (d, *J* = 7.4 Hz, 1H), 7.93 (t, *J* = 7.6 Hz, 1H), 7.87 (d, *J* = 7.8 Hz, 1H), 7.65 (t, *J* = 8.2 Hz, 1H), 7.54 (d, *J* = 9.2 Hz, 1H), 7.25 (d, *J* = 7.8 Hz, 1H), 6.88 (d, *J* = 9.1 Hz, 1H), 6.66 (d, *J* = 8.0 Hz, 2H), 6.12 (dd, *J* = 8.3, 2.2 Hz, 2H), 3.82-3.67 (m, 4H), 3.50 (s, 3H), 1.96 (dd, *J* = 21.9, 14.9 Hz, 1H), 1.56 (dd, *J* = 21.7, 14.9 Hz, 1H), 1.05 (dd, *J* = 12.4, 7.0 Hz, 6H); ^{13}C NMR (151 MHz, $CDCl_3$) δ 158.7, 142.9, 137.8 (d, *J* = 3.9 Hz), 136.4 (d, *J* = 9.3 Hz), 131.4, 131.1, 130.7, 130.0, 129.6, 129.5, 128.9, 128.9, 128.8, 128.1, 127.7 (d, *J* = 1.4 Hz), 127.1, 127.0, 127.0, 126.0, 125.4, 125.0, 124.9, 124.4, 124.2, 124.2, 120.5, 115.9, 62.2 (t, *J* = 6.7 Hz), 56.5, 32.4 (d, *J* = 137.1 Hz), 16.3 (d, *J* = 2.8 Hz), 16.3 (d, *J* = 2.9 Hz);

^{31}P NMR (162 MHz, $CDCl_3$) δ 24.4; HRMS (ESI) Calcd. for $C_{34}H_{32}O_6PS$ $[M+H]^+$: 599.1652; Found: 599.1655.

To a solution of **S32** (110 mg, 0.184 mmol) and pyrene-1,6-dicarbaldehyde (21 mg, 0.081 mmol) in THF (10 mL) was added KO*t*Bu (36 mg, 0.324 mmol) and the resulting mixture was stirred at rt for 15 h. Upon completion, the reaction was quenched by addition of saturated NH_4Cl (10 mL) and the mixture was extracted with DCM (10 mL×3). The organic phases were combined, dried over Na_2SO_4, filtered and concentrated under reduced pressure. The crude product was purified by column chromatography (eluent: EA/DCM = 1:50) to afford **18** as a yellow solid (66 mg, 72% yield). $[\alpha]^{25}_D = 66.3$ ($c = 0.2$, $CHCl_3$); ^1H NMR (600 MHz, $CDCl_3$) δ 8.33-8.28 (m, 4H), 8.25-8.18 (m, 8H), 8.14 (d, $J = 8.8$ Hz, 2H), 8.02 (d, $J = 7.7$ Hz, 2H), 7.97 (dd, $J = 15.5$, 7.7 Hz, 4H), 7.90 (d, $J = 7.4$ Hz, 2H), 7.76 (t, $J = 8.4$ Hz, 2H), 7.57 (d, $J = 9.1$ Hz, 2H), 7.52 (t, $J = 7.5$ Hz, 2H), 7.39-7.34 (m, 2H), 7.27 (d, $J = 7.4$ Hz, 2H), 6.89 (d, $J = 9.0$ Hz, 2H), 6.74 (d, $J = 8.2$ Hz, 4H), 6.46 (d, $J = 8.2$ Hz, 4H), 6.31 (d, $J = 15.9$ Hz, 2H), 3.61 (s, 6H); ^{13}C NMR (151 MHz, $CDCl_3$) δ 158.8, 141.2, 137.8, 131.5, 131.4, 131.1, 131.1, 130.8, 130.1, 129.8, 129.5, 129.0, 129.0, 128.9, 128.1, 127.9, 127.8, 127.7, 127.6, 127.4, 127.3, 125.9, 125.6, 125.5, 125.4, 125.4, 125.3, 124.6, 124.5, 124.3, 124.2, 124.0, 123.3, 120.6, 115.9, 56.6; HRMS (ESI) Calcd. for $C_{78}H_{51}O_6S_2$ $[M+H]^+$: 1147.3122; Found: 1147.3121.

1.10 Enantioselective Synthesis of Isoplagiochin D

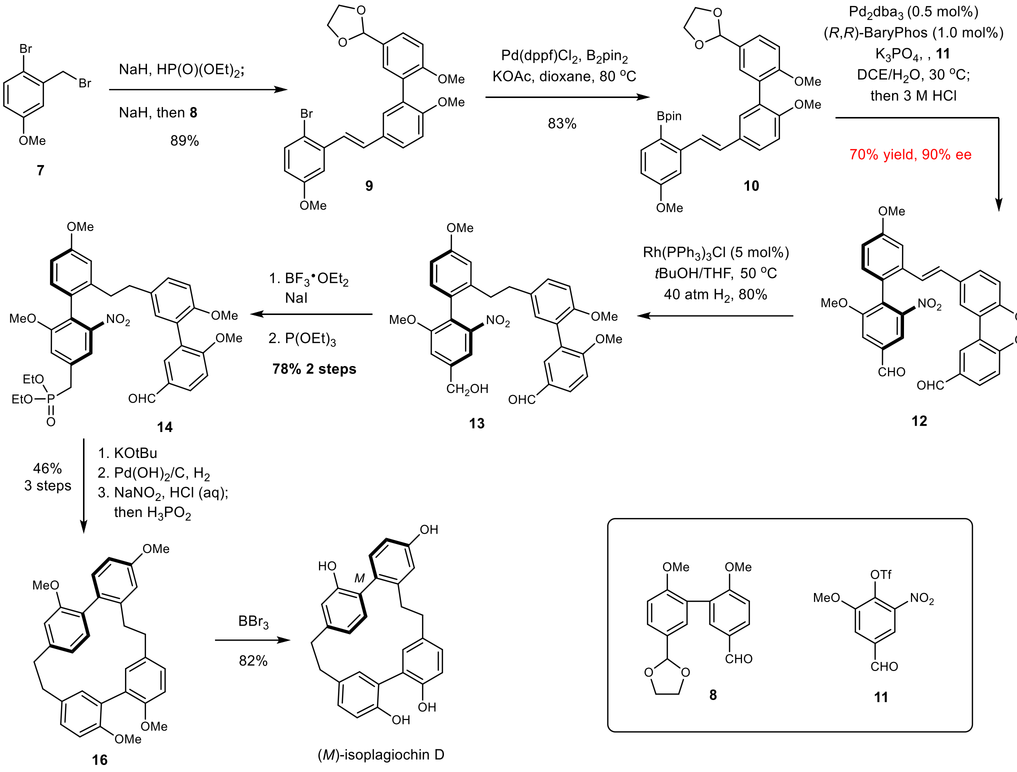

Supplementary Figure 4 Synthetic route of isoplagiochin D

Synthesis of 5'-(1,3-dioxolan-2-yl)-2',6-dimethoxy-[1,1'-biphenyl]-3-carbaldehyde (8)

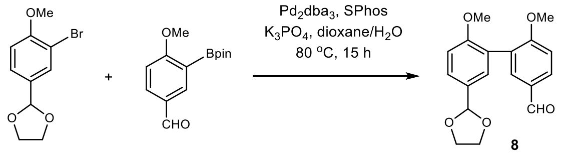

To a mixture of 2-(3-bromo-4-methoxyphenyl)-1,3-dioxolane (10.7 g, 41.3 mmol), 4-methoxy-3-(4,4,5,5-tetramethyl-1,3,2-dioxaborolan-2-yl)benzaldehyde (13.0 g, 49.6 mmol), potassium phosphate (26.3 g, 123.9 mmol), $Pd_2(dba)_3$ (0.75 g, 0.82 mmol) and SPhos (0.67 g, 1.64 mmol) under N_2 was charged degassed dioxane (50 mL)/H_2O (10 mL). The mixture was stirred at 80 ºC for 15 h. Upon full consumption of aryl bromide, the mixture was concentrated under reduced pressure. The residue was partitioned between ethyl acetate (50 mL) and water (30 mL). The organic phase was separated and the aqueous phase was extracted with EA (20 mL × 3). The organic layers were combined, dried over Na_2SO_4, filtered and concentrated. The crude product was purified by silica gel flash column chromatography (eluent: PE:EA = 6:1) to afford **8** as a colorless oil (11.5 g, 89% yield). ^{1}H NMR (400 MHz, $CDCl_3$) δ 9.90 (s, 1H), 7.88 (dd, J = 8.5, 2.1 Hz, 1H), 7.78 (d, J = 2.1 Hz, 1H), 7.47 (dd, J = 8.5, 2.1 Hz, 1H), 7.35 (d, J = 2.1 Hz, 1H), 7.06 (d, J = 8.5 Hz, 1H), 6.97 (d, J = 8.5 Hz, 1H), 5.79 (s, 1H), 4.17-4.08 (m, 2H), 4.06-3.97 (m, 2H), 3.83 (s, 3H), 3.77 (s, 3H); ^{13}C NMR (126 MHz, $CDCl_3$) δ 191.1, 162.3, 157.9, 133.5, 131.4, 129.9, 129.6, 128.5, 127.7, 126.5, 110.9, 110.8, 103.7, 65.4, 56.0, 55.9, 24.9; HRMS (ESI) Calcd. for $C_{18}H_{19}O_5$ $[M+H]^+$:

315.1232; Found: 315.1235.

Synthesis of (E)-2-(5'-(2-bromo-5-methoxystyryl)-2',6-dimethoxy-[1,1'-biphenyl]-3-yl)-1,3-dioxolane (9)

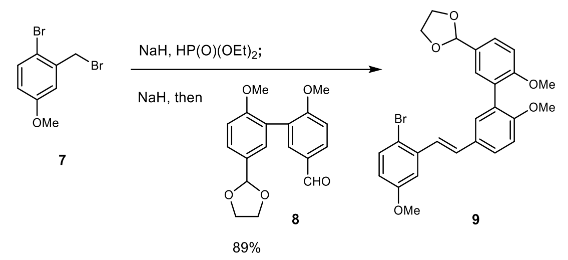

To a solution of diethyl phosphite (6.9 mL, 53.6 mmol) in anhydrous THF (30 mL) was added NaH (60 wt%, 2.2 g, 55.0 mmol) in three portions at room temperature and the resulting mixture was stirred for 30 min. The mixture was cooled to 0 °C and a solution of 1-bromo-2-(bromomethyl)-4-methoxybenzene (15.0 g, 53.6 mmol) in anhydrous THF (20 mL) was added over 20 min. The reaction system was allowed to warm to room temperature and stirred for 8 h. Upon consumption of the corresponding benzyl bromide, The mixture was cooled to 0 °C and NaH (60 wt%, 2.2 g, 55.0 mmol) was added. The mixture was stirred at 0 °C for 30 min before the addition of a solution of **8** (16.8 g, 53.6 mmol) in anhydrous THF (15 mL). The reaction system was allowed to warm to room temperature and stirred for 12 h. Upon completion, the mixture was concentrated under reduced pressure and the residue was dissolved in EA (80 mL) and H_2O (20 mL). The organic phase was separated and washed with brine (30 mL). The organic layer was separated, dried over Na_2SO_4, filtered and concentrated. The crude product was purified by silica gel flash column chromatography (eluent: PE:EA = 3:1) to afford **9** as a colorless oil (23.7 g, 89% yield). ^1H NMR (500 MHz, $CDCl_3$) δ 7.53 (dd, J = 8.5, 2.3 Hz, 1H), 7.50-7.44 (m, 3H), 7.40 (d, J = 2.2 Hz, 1H), 7.30 (d, J = 16.1 Hz, 1H), 7.18 (d, J = 3.0 Hz, 1H), 7.02 (d, J = 11.2 Hz, 1H), 6.99 (d, J = 7.1 Hz, 1H), 6.98 (d, J = 12.1 Hz, 1H), 6.69 (dd, J = 8.8, 3.0 Hz, 1H), 5.83 (s, 1H), 4.17-4.11 (m, 2H), 4.07-3.99 (m, 2H), 3.83 (s, 3H), 3.81 (s, 3H), 3.79 (s, 3H); ^{13}C NMR (126 MHz, $CDCl_3$) δ 159.1, 158.0, 157.4, 138.3, 133.6, 131.2, 130.0, 129.8, 129.8, 129.5, 128.1, 127.7, 127.6, 127.2, 125.5, 114.9, 114.8, 111.6, 111.3, 110.9, 103.9, 65.4, 56.0, 55.9, 55.6; HRMS (ESI) Calcd. for $C_{26}H_{25}BrNaO_5$ $[M+Na]^+$: 519.0783; Found: 519.0789.

Synthesis of (*E*)-2-(2-(2-(5'-(1,3-dioxolan-2-yl)-2',6-dimethoxy-[1,1'-biphenyl]-3-yl)vinyl)-4-methoxyphenyl)-4,4,5,5-tetramethyl-1,3,2-dioxaborolane (10)

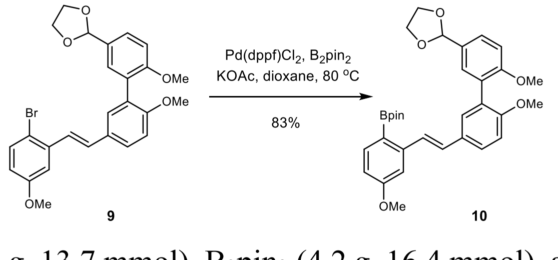

To a mixture of **9** (6.8 g, 13.7 mmol), B_2pin_2 (4.2 g, 16.4 mmol), and potassium acetate (4.0 g, 41.1 mmol) was added Pd(dppf)Cl_2 (0.30 g, 0.41 mmol) and the system was

evacuated and back-filled with N_2 for three times. Degassed dioxane (40 mL) was added and the mixture was stirred at 80 °C for 20 h. Upon full consumption of aryl bromide, the mixture was concentrated under reduced pressure. The residue was partitioned between ethyl acetate (40 mL) and water (20 mL). The organic phase was separated and the aqueous phase was extracted with EA (20 mL × 3). The organic layers were combined, dried over Na_2SO_4, filtered and concentrated. The crude product was purified by silica gel flash column chromatography (eluent: PE:EA = 8:1) to afford **10** as a colorless oil (7.5 g, 83% yield). ^{1}H NMR (500 MHz, $CDCl_3$) δ 7.97 (d, *J* = 16.3 Hz, 1H), 7.76 (d, *J* = 8.3 Hz, 1H), 7.52 (dd, *J* = 8.5, 2.3 Hz, 1H), 7.45 (dd, *J* = 8.4, 2.2 Hz, 1H), 7.43 (d, *J* = 2.2 Hz, 1H), 7.40-7.38 (m, 1H), 7.22 (d, *J* = 2.4 Hz, 1H), 7.00 (d, *J* = 11.8 Hz, 1H), 6.98-6.95 (m, 2H), 6.78 (dd, *J* = 8.3, 2.5 Hz, 1H), 5.81 (d, *J* = 3.6 Hz, 1H), 4.15-4.11 (m, 2H), 4.04-4.00 (m, 2H), 3.87 (s, 3H), 3.79 (s, 3H), 3.79 (s, 3H), 1.34 (s, 12H); ^{13}C NMR (126 MHz, $CDCl_3$) δ 162.0, 158.0, 156.9, 146.1, 138.1, 130.6, 130.3, 129.9, 129.9, 129.0, 128.0, 128.0, 127.1, 126.9, 112.5, 111.4, 110.9, 109.3, 103.9, 83.5, 65.4, 56.0, 56.0, 553, 25.1; HRMS (EI) Calcd. for $C_{32}H_{37}BO_7$ $[M]^+$: 544.2632; Found: 544.2629.

Synthesis of (*R*,*E*)-5'-(2-(4'-formyl-2',4-dimethoxy-6'-nitro-[1,1'-biphenyl]-2-yl)vinyl)-2',6-dimethoxy-[1,1'-biphenyl]-3-carbaldehyde (12)

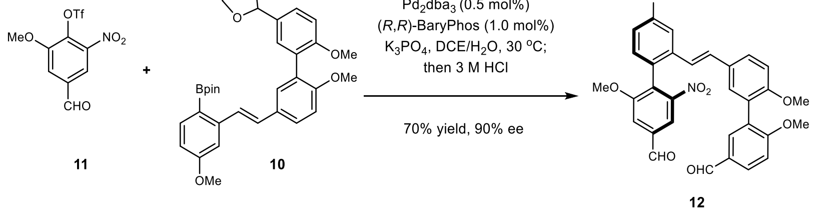

To a mixture of **11** (2.0 g, 6.1 mmol), **10** (4.0 g, 7.3 mmol), potassium phosphate (3.9 g, 18.3 mmol), $Pd_2(dba)_3$ (28.0 mg, 0.031 mmol) and (*R*, *R*)-BaryPhos (32.6 mg, 0.062 mmol) under N_2 was charged degassed DCE (10 mL)/H_2O (2 mL). The mixture was stirred at 30 °C for 15 h. Upon completion, the mixture was partitioned between DCM (15 mL) and water (10 mL). The organic phase was separated and the aqueous phase was extracted with DCM (10 mL × 3). The organic layers were combined, dried over Na_2SO_4, filtered and concentrated. The crude product was purified by silica gel flash column chromatography (eluent: PE:EA = 1:1) to afford **12** as a colorless oil (2.4 g, 70% yield, 90% ee). $[\alpha]^{25}_D$ = 86.4 (*c* = 0.5, $CHCl_3$); ^{1}H NMR (500 MHz, $CDCl_3$) δ 10.04 (s, 1H), 9.90 (s, 1H), 7.95 (d, *J* = 1.3 Hz, 1H), 7.88 (dd, *J* = 8.5, 2.2 Hz, 1H), 7.75 (d, *J* = 2.1 Hz, 1H), 7.65 (d, *J* = 1.2 Hz, 1H), 7.29 (dd, *J* = 8.6, 2.3 Hz, 1H), 7.24 (d, *J* = 2.5 Hz, 1H), 7.17 (d, *J* = 2.2 Hz, 1H), 7.07 (d, *J* = 8.5 Hz, 1H), 7.00 (d, *J* = 8.4 Hz, 1H), 6.97 (d, *J* = 16.0 Hz, 1H), 6.90 (d, *J* = 8.6 Hz, 1H), 6.86 (dd, *J* = 8.4, 2.6 Hz, 1H), 6.55 (d, *J* = 16.1 Hz, 1H), 3.89 (s, 3H), 3.84 (s, 3H), 3.84 (s, 4H), 3.75 (s, 3H); ^{13}C NMR (151 MHz, $CDCl_3$) δ 191.1, 189.8, 162.2, 160.2, 159.2, 157.0, 151.8, 138.2, 136.9, 133.2, 131.8, 130.7, 130.6, 130.2, 129.9, 129.8, 129.7, 128.4, 127.6, 126.7, 124.2, 123.0, 118.4, 113.4, 112.3, 111.3, 111.0, 110.7, 57.0, 56.1, 55.9, 55.4; HRMS (ESI) Calcd. for $C_{32}H_{28}NO_8$ $[M+H]^+$: 554.1815; Found: 554.1817. Enantiomeric excess was determined by chiral HPLC (Chiralcel AD-3, 25 °C, flow rate: 0.8 mL/min, hexanes/isopropanol:

70/30, 290 nm, 35.92 min (minor isomer), 40.36 min (major isomer)).

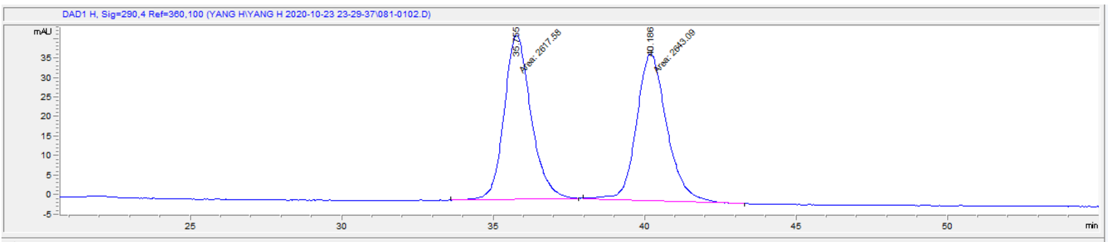

#	Time 时间	Area 峰面积	Height 峰高	Width 峰宽	Symmetry factor 对称因子	Area% 峰面积 %	Area%
1	35.755	2617.6	42	1.0381	0.815	49.757	49.757
2	40.186	2643.1	37.6	1.1718	0.839	50.243	50.243

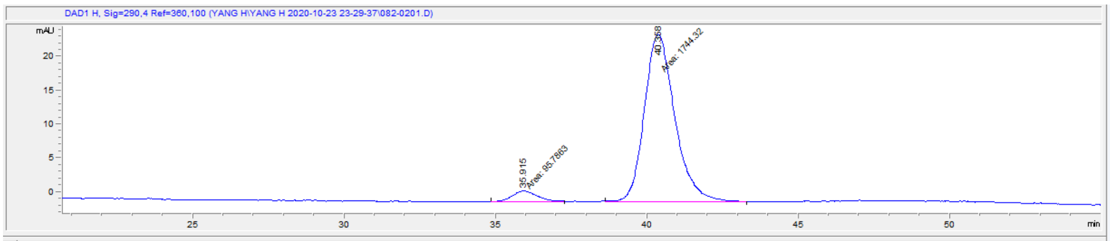

#	Time 时间	Area 峰面积	Height 峰高	Width 峰宽	Symmetry factor 对称因子	Area% 峰面积 %	Area%
1	35.915	95.8	1.6	1.0002	0.794	5.205	5.205
2	40.358	1744.3	24.8	1.1722	0.772	94.795	94.795

Synthesis of (*R*)-5'-(2-(4'-(hydroxymethyl)-2',4-dimethoxy-6'-nitro-[1,1'-biphenyl]-2-yl)ethyl)-2',6-dimethoxy-[1,1'-biphenyl]-3-carbaldehyde (13)

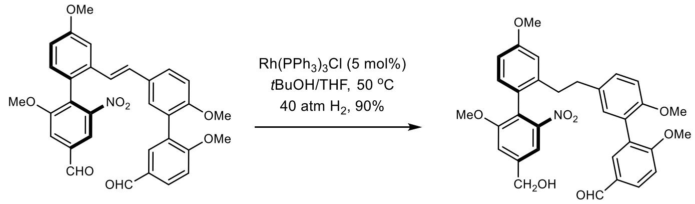

12 **13**

To a solution of **12** (1.6 g, 2.9 mmol) in degassed *t*BuOH (10 mL) and THF (10 mL) in glove box was added $Rh(PPh_3)_3Cl$ (80.5 mg, 0.087 mmol). The reaction vial contained in high pressure reaction vessel was purged with H_2 and then stirred under 25 atm H_2 at room temperature for 48 h. Upon completion, the mixture was concentrated under reduced pressure and the crude product was purified by silica gel flash column chromatography (eluent: EA:PE = 1:2) to afford **13** as a colorless oil (1.4 g, 90% yield). $[\alpha]^{25}_{D}$ = 59.8 (*c* = 0.5, $CHCl_3$); ^1H NMR (500 MHz, $CDCl_3$) δ 9.91 (s, 1H), 7.86 (dd, *J* = 8.5, 2.1 Hz, 1H), 7.69 (d, *J* = 2.1 Hz, 1H), 7.40 (s, 1H), 7.20 (s, 1H), 7.09-7.02 (m, 2H), 6.96 (d, *J* = 8.4 Hz, 1H), 6.84 (dd, *J* = 6.6, 2.1 Hz, 3H), 6.77 (dd, *J* = 8.4, 2.6 Hz, 1H), 4.77 (s, 2H), 3.85 (s, 3H), 3.80 (s, 3H), 3.77 (s, 3H), 3.72 (s, 3H), 2.77 (dd, *J* = 10.3, 6.2 Hz, 2H), 2.68-2.61 (m, 2H), 2.42 (s, 1H); ^{13}C NMR (151 MHz, $CDCl_3$) δ 191.5, 162.5, 159.6, 158.3, 155.4, 151.4, 143.2, 142.3, 134.2, 132.5, 132.4, 131.3, 130.4, 129.6, 129.1, 126.4, 124.3, 123.5, 114.7, 113.2, 112.2, 111.4, 111.1, 110.9, 64.2, 56.5, 56.1, 55.9, 55.2, 36.2, 35.7; HRMS (ESI) Calcd. for $C_{32}H_{32}NO_8$ $[M+H]^+$: 558.2128; Found: 558.2133.

Synthesis of diethyl (R)-((2'-(2-(5'-formyl-2',6-dimethoxy-[1,1'-biphenyl]-3-yl)ethyl)-2,4'-dimethoxy-6-nitro-[1,1'-biphenyl]-4-yl)methyl)phosphonate (14)

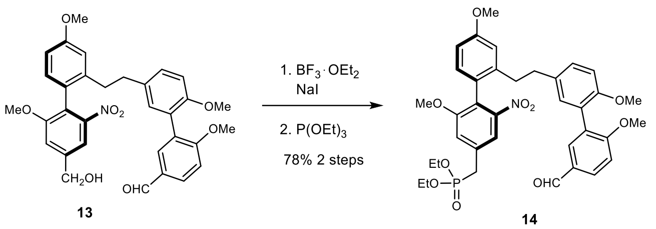

To a solution of **13** (1.4 g, 2.5 mmol) in anhydrous MeCN (15 mL) at 0 °C was added NaI (0.75 g, 5.0 mmol), followed by BF_3· Et_2O (46.5 wt%, 0.65 mL, 5.0 mmol). The mixture was stirred at 0 °C for 2 h and then the reaction was quenched by slow addition of H_2O (5 mL). The mixture was concentrated under reduced pressure and the residue was dissolved in EA (30 mL) and H_2O (10 mL). The organic phase was separated and washed with brine (10 mL). The organic layer was dried over Na_2SO_4, filtered and concentrated to provide **14** as a brown oil (78% yield over 2 steps). $[\alpha]^{25}_{D}$ = 30.3 (*c* = 0.5, $CHCl_3$); ^1H NMR (400 MHz, $CDCl_3$) δ 9.91 (s, 1H), 7.87 (dd, *J* = 8.5, 2.1 Hz, 1H), 7.71 (d, *J* = 2.1 Hz, 1H), 7.34 (s, 1H), 7.14 (s, 1H), 7.06 (d, *J* = 8.5 Hz, 1H), 7.02-6.98 (m, 1H), 6.95 (d, *J* = 8.2 Hz, 1H), 6.91 (d, *J* = 2.2 Hz, 1H), 6.83 (d, *J* = 8.4 Hz, 1H), 6.78 (dt, *J* = 8.3, 2.5 Hz, 2H), 4.13-4.03 (m, 4H), 3.84 (s, 3H), 3.78 (s, 3H), 3.77 (s, 3H), 3.72 (s, 3H), 3.25-3.14 (m, 2H), 2.79-2.70 (m, 2H), 2.68-2.60 (m, 2H), 1.27 (q, *J* = 7.2 Hz, 7H); ^{13}C NMR (151 MHz, $CDCl_3$) δ 191.1, 162.3, 159.6, 158.1 (d, *J* = 3.0 Hz), 155.3, 151.3 (d, *J* = 3.3 Hz), 142.2, 134.0, 133.7 (d, *J* = 8.9 Hz), 133.3, 131.6, 131.3, 130.4, 129.7, 129.0, 128.9, 126.3, 124.0, 123.3 (d, *J* = 3.5 Hz), 116.7 (d, *J* = 7.1 Hz), 115.8 (d, *J* = 5.8 Hz), 114.8, 111.4, 111.1, 111.0, 62.6 (t, *J* = 7.6 Hz), 56.5, 56.1, 55.9, 55.2, 36.0, 35.5, 33.8 (d, *J* = 138.8 Hz), 16.5 (d, *J* = 5.8 Hz); ^{31}P NMR (162 MHz, $CDCl_3$) δ 20.8; HRMS (ESI) Calcd. for $C_{36}H_{41}NO_{10}P$ $[M+H]^+$: 678.2463; Found: 678.2460.

Synthesis of 1^4,2^2,5^4,6^6-tetramethoxy-1(1,2),2(1,4),5,6(1,3)-tetrabenzenacyclooctaphane (16)

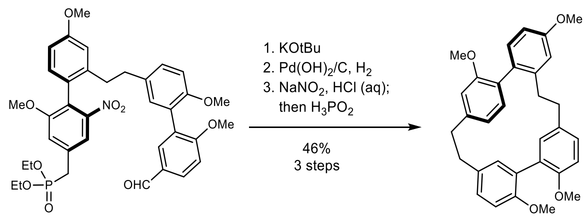

To a solution of **14** (68 mg, 0.10 mmol) in anhydrous THF (5 mL) was added KO*t*Bu (22 mg, 0.20 mmol) and the mixture was stirred at rt for 20 h. Upon completion, H_2O (10 mL) was added and the mixture was extracted with EA (10 mL×3). The organic phases were combined and washed with brine (10 mL). The organic layer was dried over Na_2SO_4, filtered and concentrated give a light yellow oil.

The above obtained intermediate was dissolved in THF (10 mL) and 15% $Pd(OH)_2$ (20 mg, 15 wt%) was added to the solution. The mixture was stirred at 30 °C under 1 atm of H_2 for 24 h. Upon completion, the mixture was filtered through a layer of Celite and the catalyst was wash twice with THF (15 mL). The combined organic solvent was concentrated under reduced pressure and the residue was dissolved in 1,4-dioxane (2

mL) and 3 N HCl (aq) (5 mL). The solution was cooled to 0 °C and $NaNO_2$ (7.6 mg, 0.11 mmol) was added. The mixture was stirred at 0 °C for 2 h before the addition of H_3PO_2 (50 µL, 1.0 mmol). The mixture was stirred at 0 °C for 3 h and then warmed up to rt and further stirred for 5 h. The reaction solution was diluted with H_2O (10 mL) and the mixture was extracted with EA (10 mL×3). The organic layers were combined, dried over Na_2SO_4, filtered and concentrated. The crude product was purified by silica gel flash column chromatography (eluent: PE:EA = 6:1) to afford **16** as a colorless oil (22 mg, 46% yield over 3 steps). $[\alpha]^{25}_D$ = 20.9 (*c* = 0.5, $CHCl_3$); ^1H NMR (400 MHz, $CDCl_3$) δ 7.16 (dd, *J* = 14.3, 5.5 Hz, 1H), 7.12-6.96 (m, 4H), 6.92-6.84 (m, 2H), 6.80 (dd, *J* = 8.4, 2.6 Hz, 1H), 6.74 (dd, *J* = 8.2, 3.0 Hz, 1H), 6.40 (d, *J* = 7.0 Hz, 0.5H), 6.34 (d, *J* = 2.3 Hz, 0.5H), 6.30 (s, 1H), 6.26 (d, *J* = 2.3 Hz, 0.5H), 6.22 (s, 0.5H), 3.85 (s, 3H), 3.80 (s, 1.5H), 3.78 (s, 1.5H), 3.76 (s, 1.5H), 3.76 (s, 1.5H), 3.72 (s, 1.5H), 3.49 (s, 1.5H), 3.28 (dd, *J* = 8.7, 4.2 Hz, 1H), 3.01 (d, *J* = 12.2 Hz, 1H), 2.88 (d, *J* = 13.2 Hz, 1H), 2.84-2.65 (m, 2H), 2.53 (ddt, *J* = 24.5, 21.1, 11.6 Hz, 3H); ^{13}C NMR (151 MHz, $CDCl_3$) δ 158.9, 158.9, 157.3, 156.6, 155.3, 155.3, 155.2, 155.2, 143.6, 143.2, 142.2, 142.0, 135.6, 135.4, 134.4, 134.1, 134.0, 133.9, 133.0, 132.9, 131.7, 131.7, 131.4, 130.9, 130.7, 130.5, 129.0, 128.8, 128.2, 128.1, 127.3, 127.2, 127.0, 126.8, 123.2, 120.6, 114.1, 113.9, 113.8, 111.1, 111.0, 111.0, 110.9, 110.6, 110.4, 110.1, 55.9, 55.9, 55.8, 55.7, 55.6, 55.4, 55.2, 39.1, 38.8, 38.5, 37.6, 36.9, 36.6, 36.0; HRMS (ESI) Calcd. for $C_{32}H_{32}NaO_4$ $[M+Na]^+$: 503.2193; Found: 503.2190.

Synthesis of (*M*)-isoplagiochin D

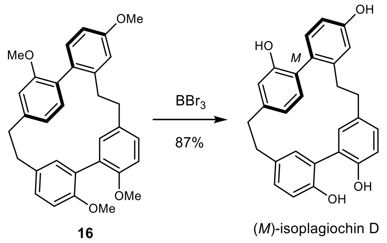

To a solution of **16** (22 mg, 0.046 mmol) in anhydrous DCM at -78 °C was added BBr_3 (44 µL, 0.46 mmol) dropwise. Then the mixture was allowed to warm to rt and stirred for 8 h. MeOH (1 mL) was added to quench the reaction, followed by addition of H_2O (10 mL). The mixture was extracted with DCM (10 mL×3). The organic layers were combined, dried over Na_2SO_4, filtered and concentrated. The crude product was purified by silica gel flash column chromatography (eluent: PE:EA = 1:1) to afford (*M*)-isoplagiochin D as a white film (17 mg, 82% yield, 90% ee). $[\alpha]^{25}_D$ = 54.3 (*c* = 0.5, MeOH); ^1H NMR (400 MHz, MeOD-d_4) δ 7.09 (dd, *J* = 8.1, 2.2 Hz, 1H), 7.03 (d, *J* = 7.6 Hz, 1H), 6.99-6.92 (m, 2H), 6.81 (d, *J* = 8.1 Hz, 1H), 6.77 (d, *J* = 2.6 Hz, 1H), 6.69 (ddd, *J* = 10.7, 9.2, 5.1 Hz, 3H), 6.64 (s, 1H), 6.45 (d, *J* = 2.1 Hz, 1H), 6.36 (d, *J* = 2.1 Hz, 1H), 3.07-2.85 (m, 3H), 2.82-2.74 (m, 1H), 2.74-2.62 (m, 3H), 2.55-2.43 (m, 1H); ^{13}C NMR (151 MHz, MeOD-d_4) δ 156.4, 154.3, 151.3, 150.7, 143.3, 142.0, 135.5, 133.5, 133.3, 133.1, 131.5, 131.3, 129.3, 128.4, 127.1, 126.9, 126.8, 125.4, 120.6, 116.6, 116.0, 115.0 (d, *J* = 3.0 Hz), 112.3, 38.5, 37.9, 37.5, 35.7; HRMS (ESI) Calcd. for $C_{28}H_{24}O_4Na$ $[M+Na]^+$: 447.1567; Found: 447.1556. Enantiomeric excess was determined by chiral HPLC (Chiralcel OD-H, 25 °C, flow rate: 0.9 mL/min,

hexanes/isopropanol: 80/20, 290 nm, 13.10 min (major isomer), 32.73 min (minor isomer)). The obtained analytical data were in full agreement with the previously reported data.[13,14]

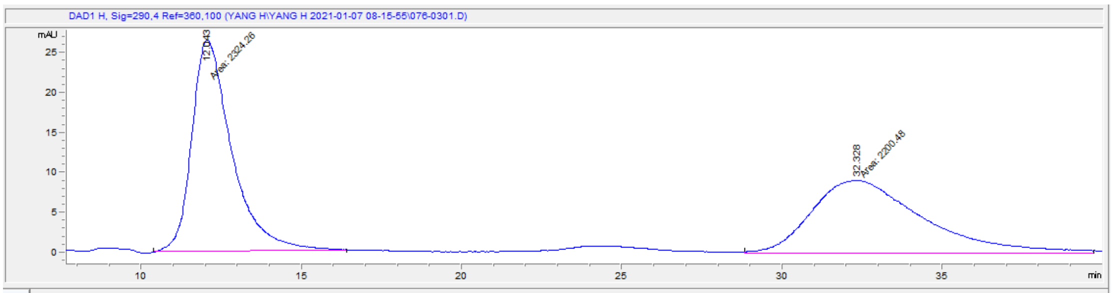

#	Time 时间	Area 峰面积	Height 峰高	Width 峰宽	Symmetry factor 对称因子	Area% 峰面积 %	Area%
1	12.043	2324.3	26.5	1.4612	0.601	51.368	51.368
2	32.328	2200.5	9.1	4.0127	0.671	48.632	48.632

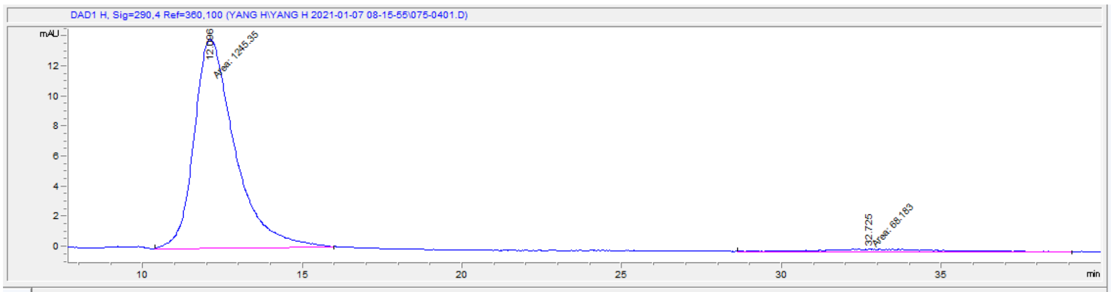

#	Time 时间	Area 峰面积	Height 峰高	Width 峰宽	Symmetry factor 对称因子	Area% 峰面积 %	Area%
1	12.096	1245.4	13.9	1.4894	0.628	94.809	94.809
2	32.725	68.2	2.1E-1	5.4928	0.695	5.191	5.191

1.11 Determination of the Absolution Configuration of 6a and *P*-17

The absolute configurations of the chiral products **6a** (CCDC 2130064) and **17** (CCDC 2130065) were determined by single crystal X-ray diffraction. The absolute stereochemistries of other products were tentatively assigned by analogy.

Supplementary Figure 5 X-ray structure of *M*-6a. with 50% probability ellipsoids.

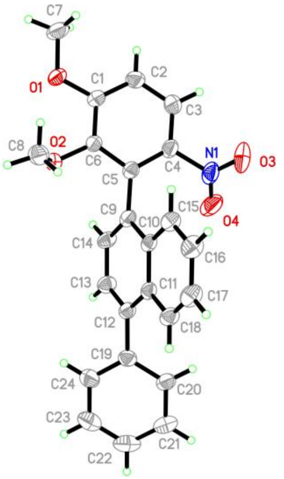

Crystal data and structure refinement.

Bond precision:	C-C = 0.0065 A		Wavelength=1.54178
Cell:	a=23.0578(5) b=23.0578(5) c=10.1350(2)		
	alpha=90 beta=90 gamma=120		
Temperature:	297 K		
	Calculated		Reported
Volume	4666.5(2)		4666.5(2)
Space group	R 3		R 3 :H
Hall group	R 3		R 3
Moiety formula	C24 H19 N O4, O		C24 H19 N O4, O
Sum formula	C24 H19 N O5		C24 H19 N O5
Mr	401.40		401.40
Dx,g cm-3	1.286		1.286
Z	9		9
Mu (mm-1)	0.745		0.745
F000	1890.0		1890.0
F000'	1896.15		
h,k,lmax	27,27,12		27,27,12
Nref	3798[1899]		3772
Tmin,Tmax	0.862,0.894		

Tmin'	0.862	
Correction method= Not given		
Data completeness= 1.99/0.99	Theta(max)= 68.269	
R(reflections)= 0.0571(3402)		wR2(reflections)= 0.1585(3772)
S = 1.064	Npar= 274	

B-level alerts are shown in IUCr's CheckCIF report of chiral products **6a** (CCDC 2130064). These alerts are all related to a water molecule coexistence with **6a** in the crystal sample.

Supplementary Figure 6 X-ray structure of *P*-17. with 50% probability ellipsoids.

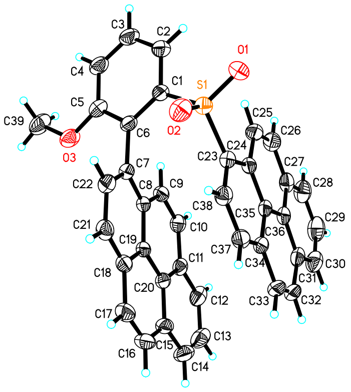

Crystal data and structure refinement.

Empirical formula	C40 H26 Cl2 O3 S	
Formula weight	657.57	
Temperature	293(2) K	
Wavelength	0.71073 Å	
Crystal system	Orthorhombic	
Space group	P 21 21 21	
Unit cell dimensions	a = 11.5498(5) Å	α= 90°.
	b = 15.9048(6) Å	β= 90°.
	c = 16.9151(7) Å	γ = 90°.
Volume	3107.3(2) $Å^3$	
Z	4	
Density (calculated)	1.406 Mg/m^3	
Absorption coefficient	0.317 mm^{-1}	
F(000)	1360	

Crystal size	0.200 x 0.150 x 0.120 mm^3
Theta range for data collection	2.135 to 25.985°.
Index ranges	-14<=h<=14, -19<=k<=19, -20<=l<=20
Reflections collected	31558
Independent reflections	6065 [R(int) = 0.0335]
Completeness to theta = 25.242°	99.3 %
Absorption correction	Semi-empirical from equivalents
Max. and min. transmission	0.7456 and 0.6300
Refinement method	Full-matrix least-squares on F^2
Data / restraints / parameters	6065 / 40 / 444
Goodness-of-fit on F^2	1.042
Final R indices [I>2sigma(I)]	R1 = 0.0422, wR2 = 0.1028
R indices (all data)	R1 = 0.0516, wR2 = 0.1105
Absolute structure parameter	0.016(18)
Extinction coefficient	0.016(3)
Largest diff. peak and hole	0.247 and -0.316 e.$Å^{-3}$

1.12 Photophysical Property Studies

Structures of **17** and **18** :

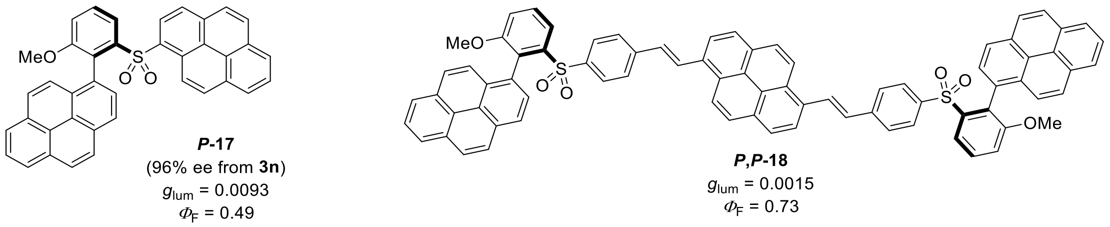

Supplementary Figure 7 Absorption and fluorescence spectra of **17** and **18** in CH_2Cl_2 (1.0×10^{-6} M), λ_{ex} = 363 nm (for **17**), λ_{ex} = 345 nm (for **18**) :

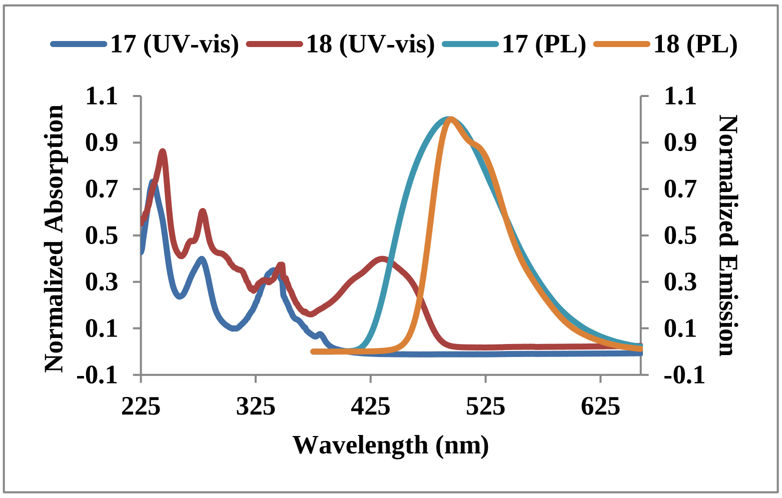

Supplementary Figure 8 CD spectra of *M*-**17** and *P*-**17** in CH_2Cl_2 (1.0×10^{-5} M) :

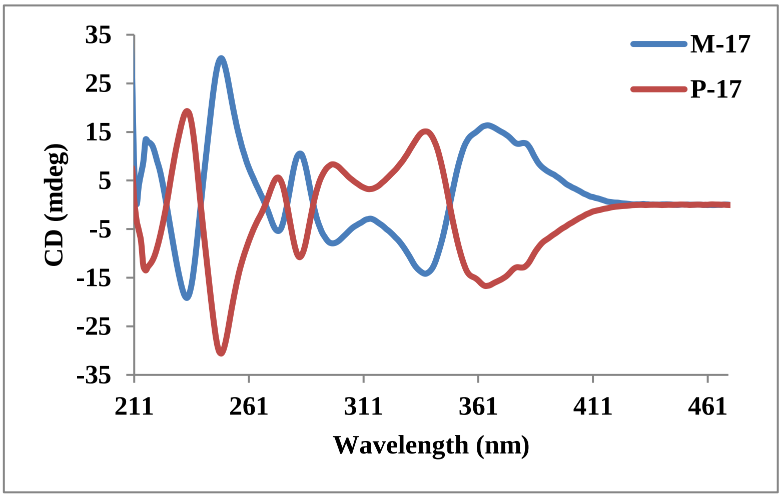

Supplementary Figure 9 CD spectra of *M,M*-**18** and *P,P*-**18** in CH_2Cl_2 (1.0×10^{-5} M) :

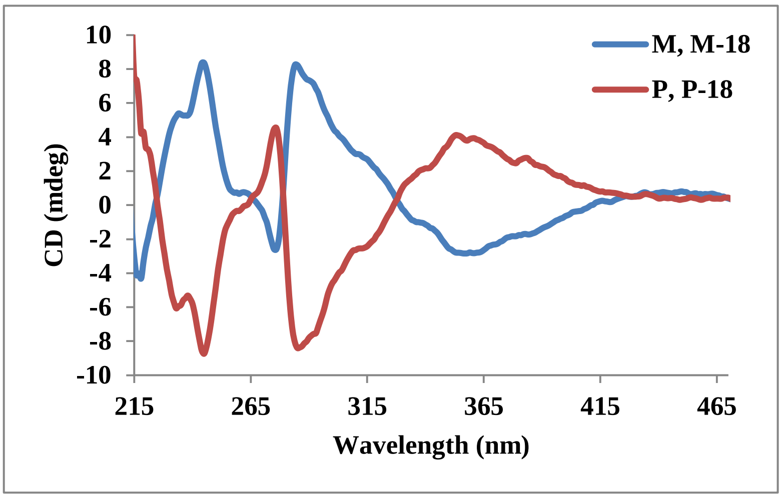

Supplementary Figure 10 CPL spectra of *M*-**17**, *P*-**17**, *M,M*-**18** and *P,P*-**18** in CH_2Cl_2 (1.0×10^{-4} M) :

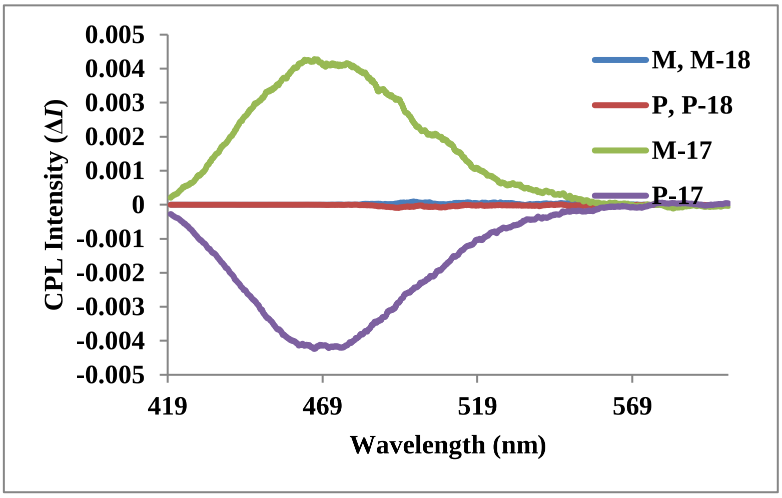

Supplementary Figure 11 g_{lum} values-wavelength curve of *M*-**17**, *P*-**17**, *M,M*-**18** and *P,P*-**18** :

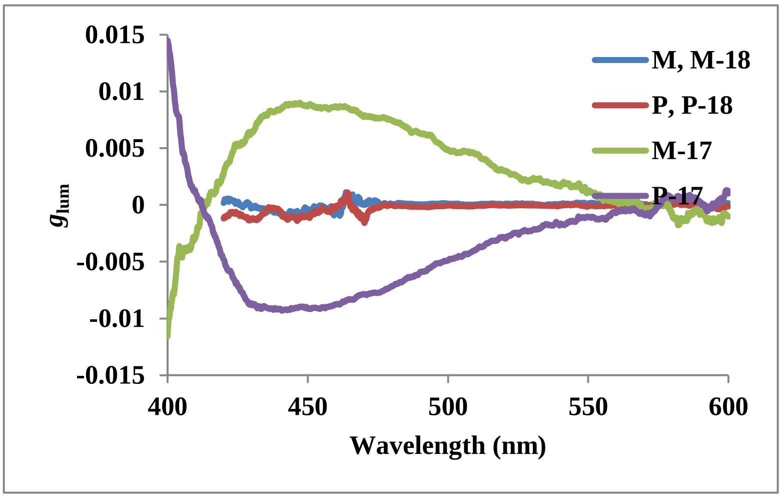

2. Supplementary Figures

2.1 NMR Spectra

Supplementary Figure 12 ^{1}H NMR spectrum of **S2** (400 MHz, $CDCl_3$)

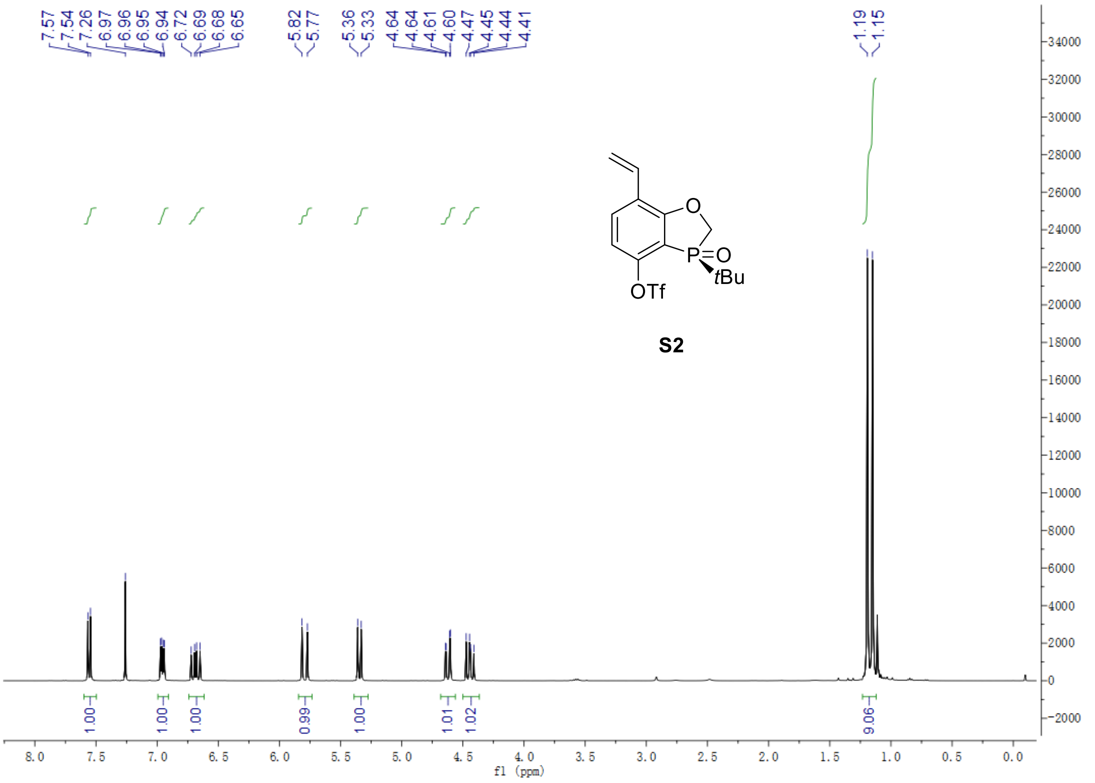

Supplementary Figure 13 ^{13}C NMR spectrum of **S2** (101 MHz, $CDCl_3$)

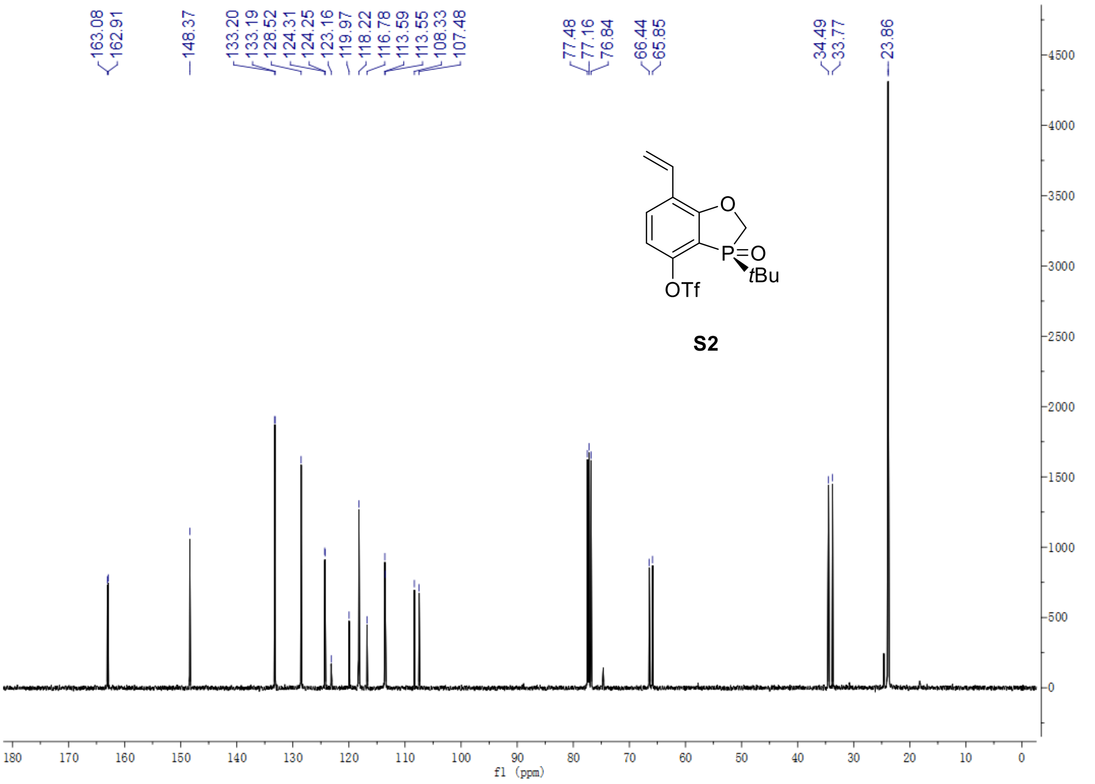

Supplementary Figure 14 ^{19}F NMR spectrum of **S2** (376 MHz, $CDCl_3$)

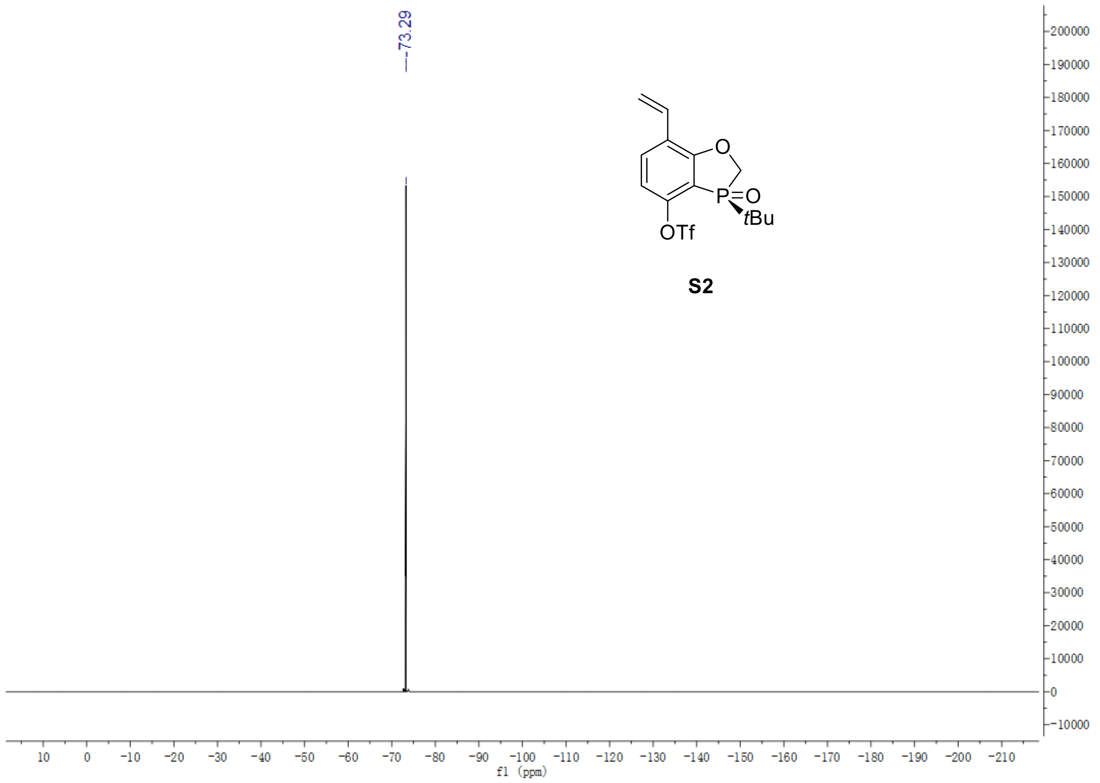

Supplementary Figure 15 ^{31}P NMR spectrum of **S2** (162 MHz, $CDCl_3$)

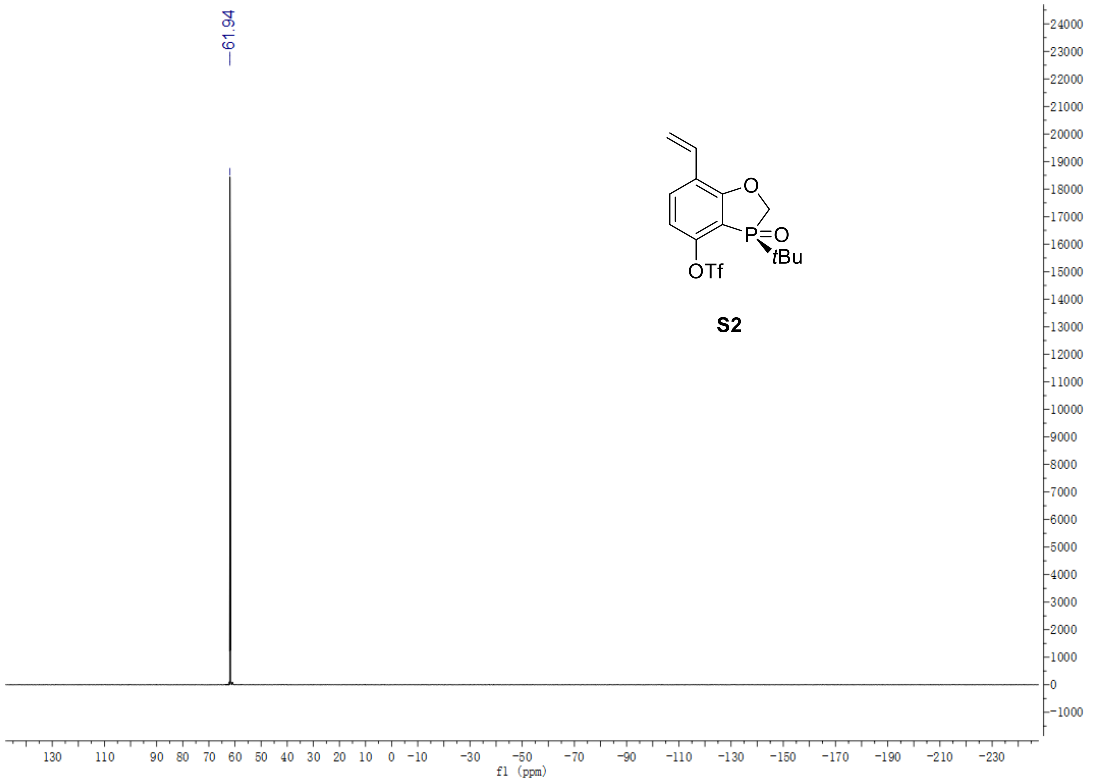

Supplementary Figure 16 ^{1}H NMR spectrum of **S3** (500 MHz, $CDCl_3$)

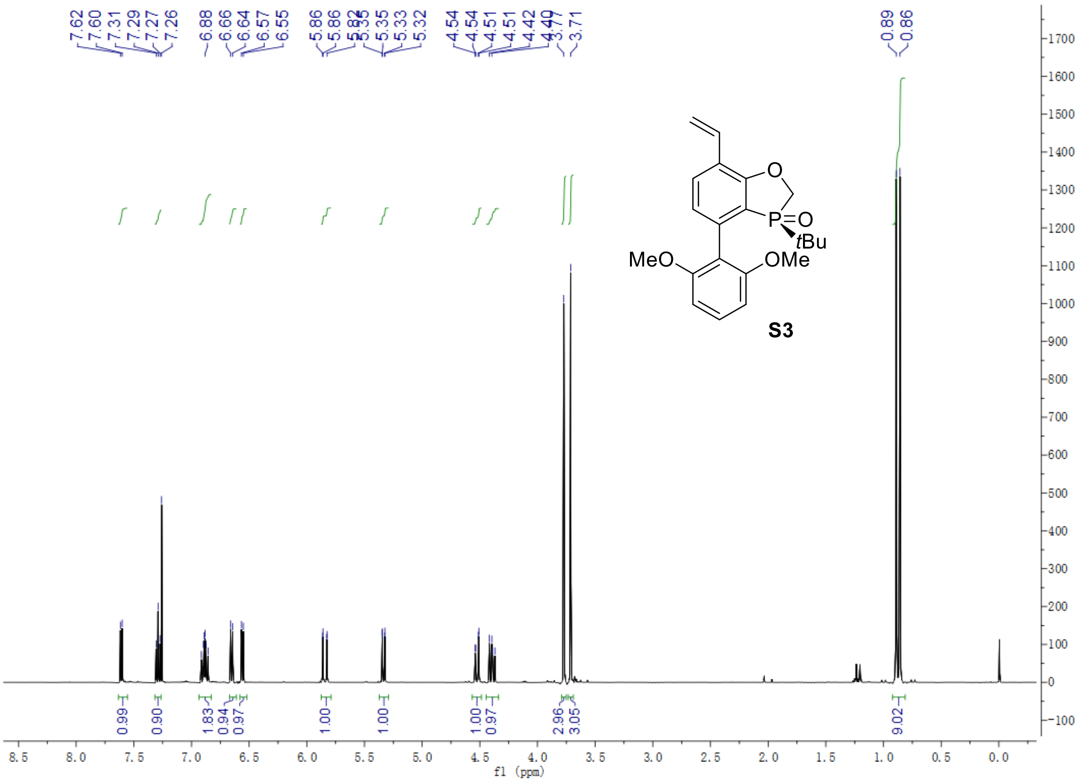

Supplementary Figure 17 ^{13}C NMR spectrum of **S3** (126 MHz, $CDCl_3$)

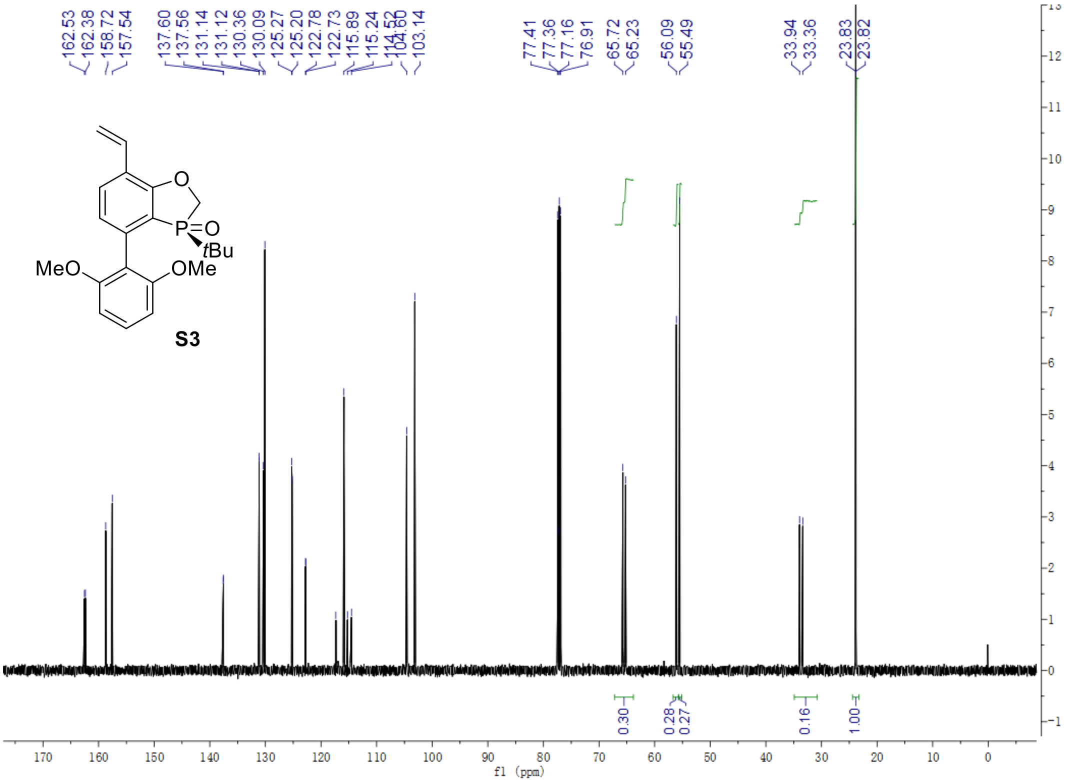

Supplementary Figure 18 ^{31}P NMR spectrum of **S3** (162 MHz, $CDCl_3$)

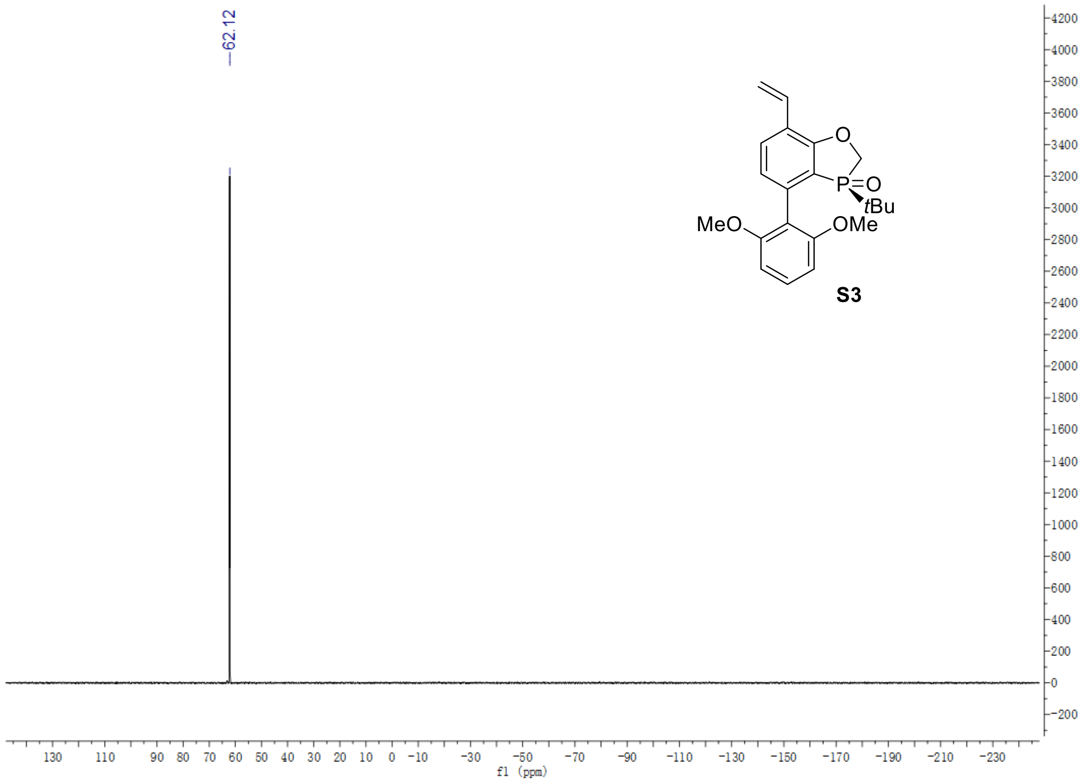

Supplementary Figure 19 ^{1}H NMR spectrum of **S4** (400 MHz, $CDCl_3$)

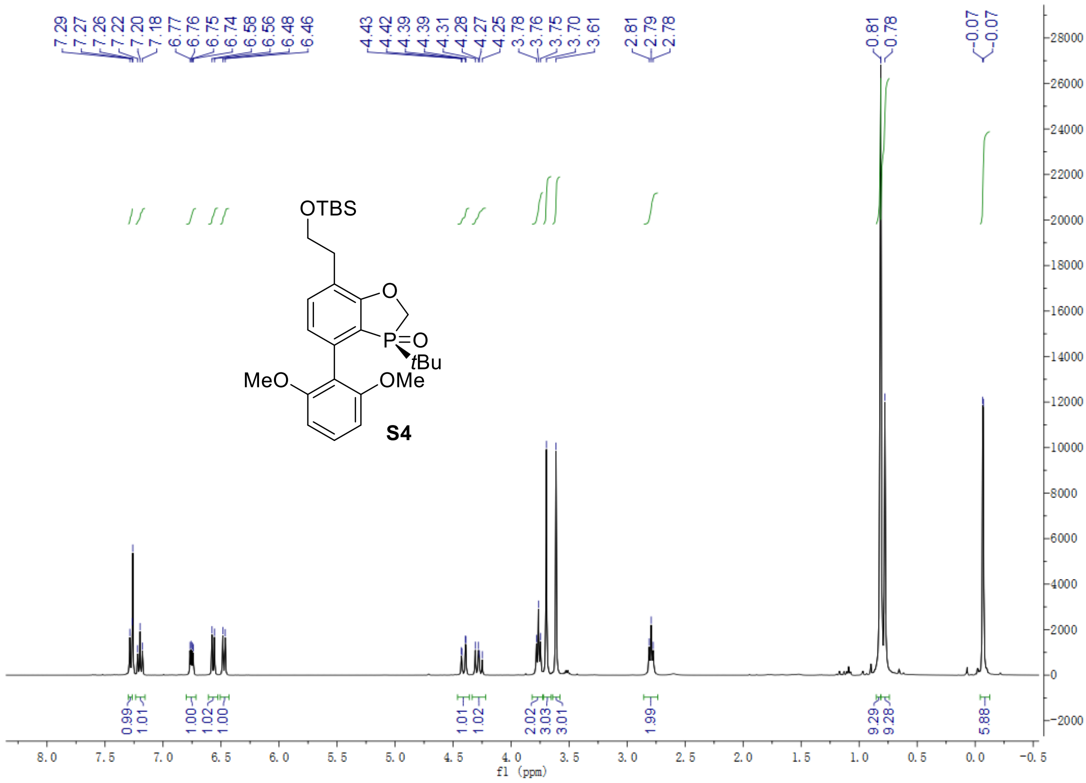

Supplementary Figure 20 ^{13}C NMR spectrum of **S4** (126 MHz, $CDCl_3$)

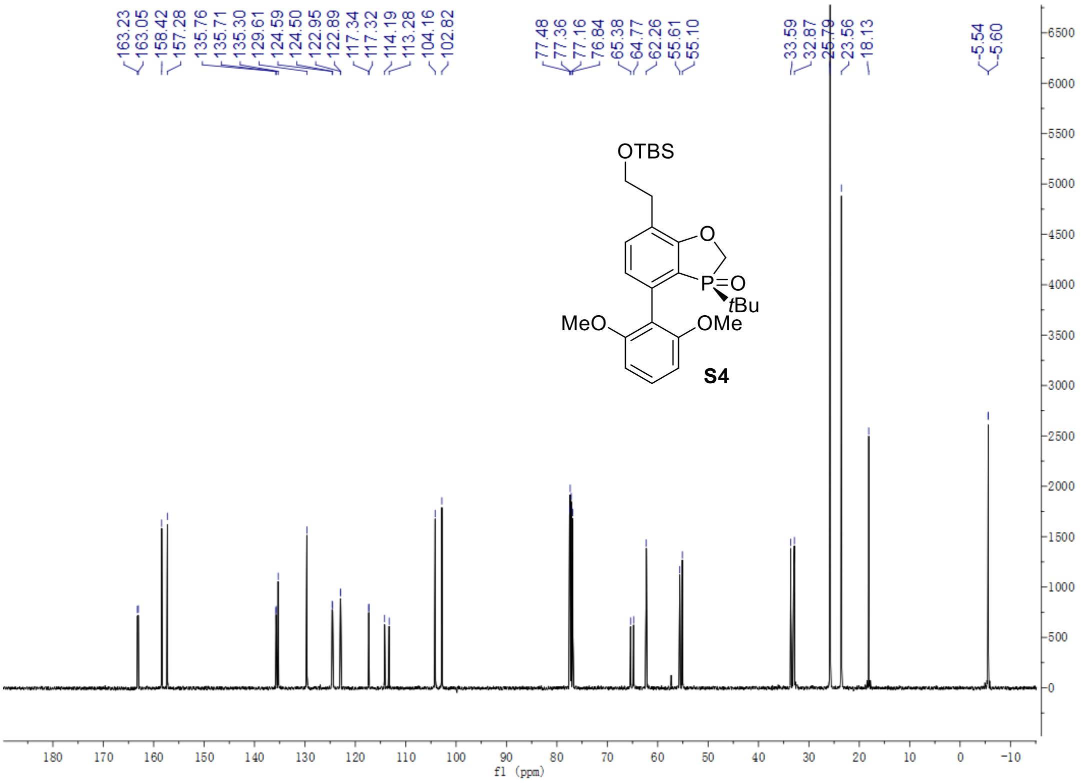

Supplementary Figure 21 ^{31}P NMR spectrum of **S4** (162 MHz, $CDCl_3$)

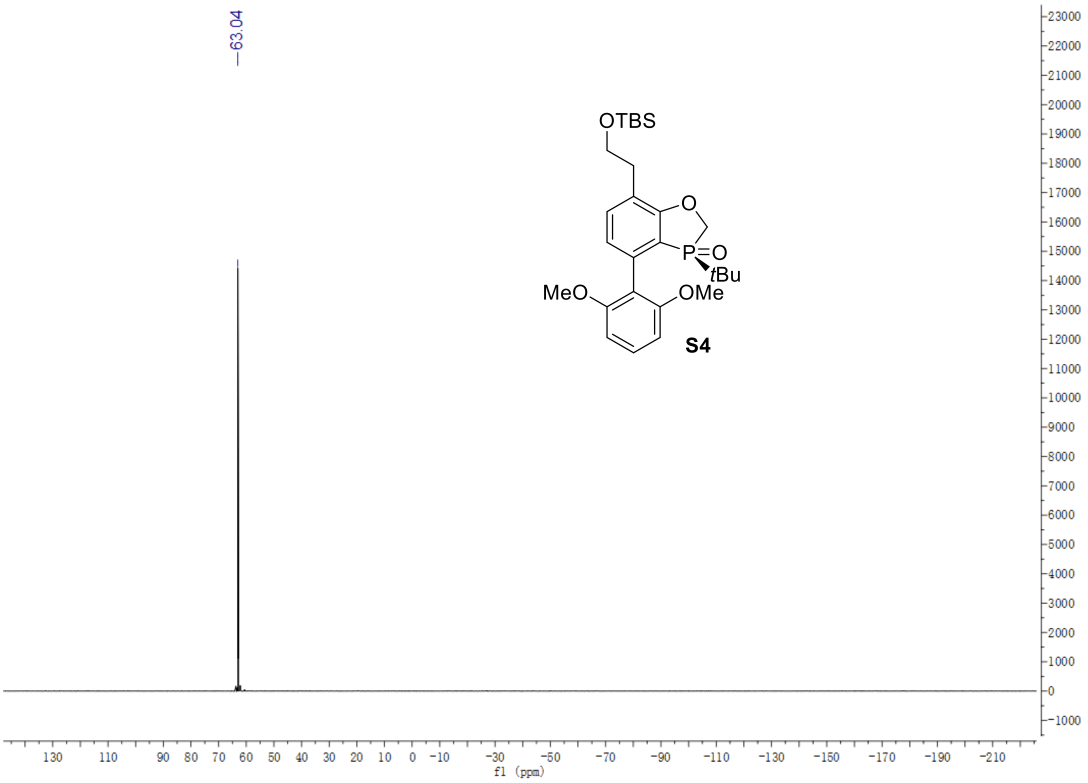

Supplementary Figure 22 ^{1}H NMR spectrum of **S8** (400 MHz, $CDCl_3$)

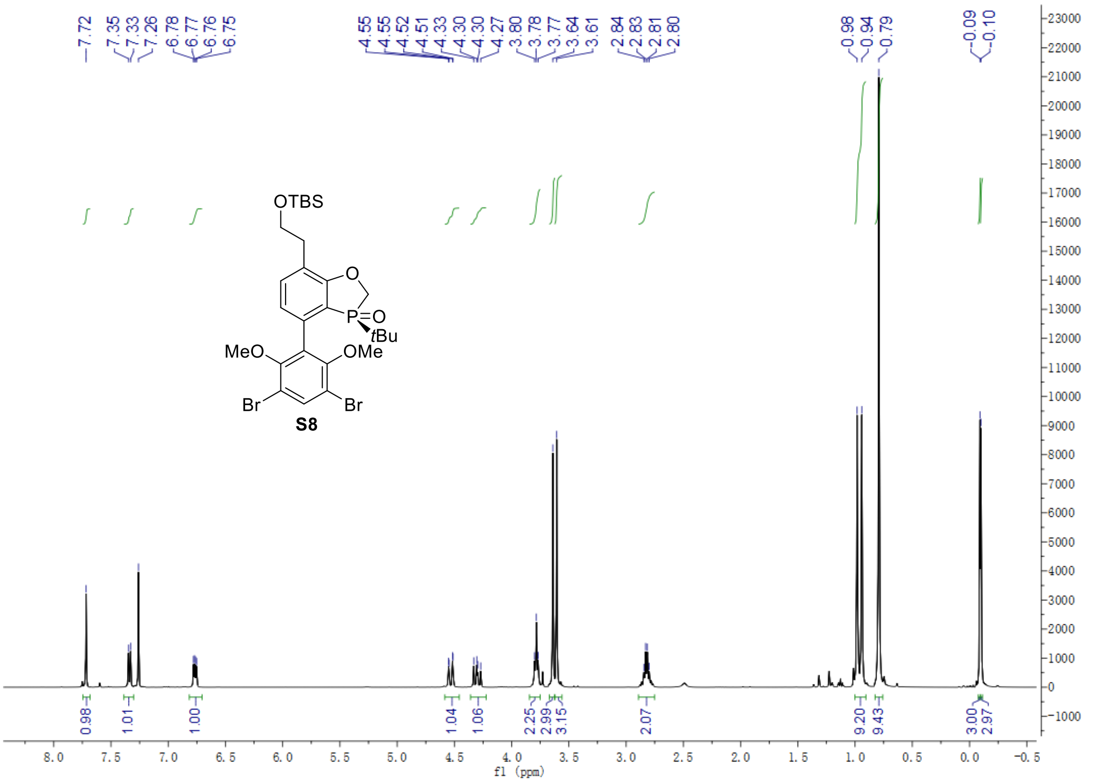

Supplementary Figure 23 ^{13}C NMR spectrum of **S8** (101 MHz, $CDCl_3$)

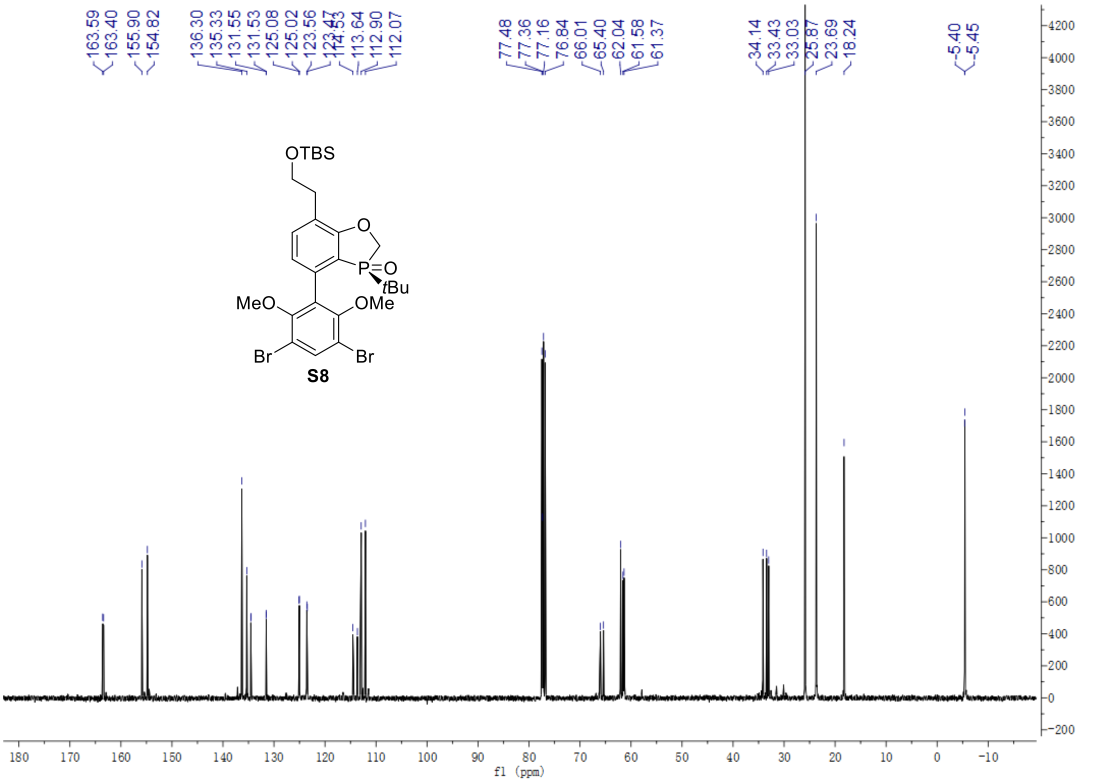

Supplementary Figure 24 ^{31}P NMR spectrum of **S8** (162 MHz, $CDCl_3$)

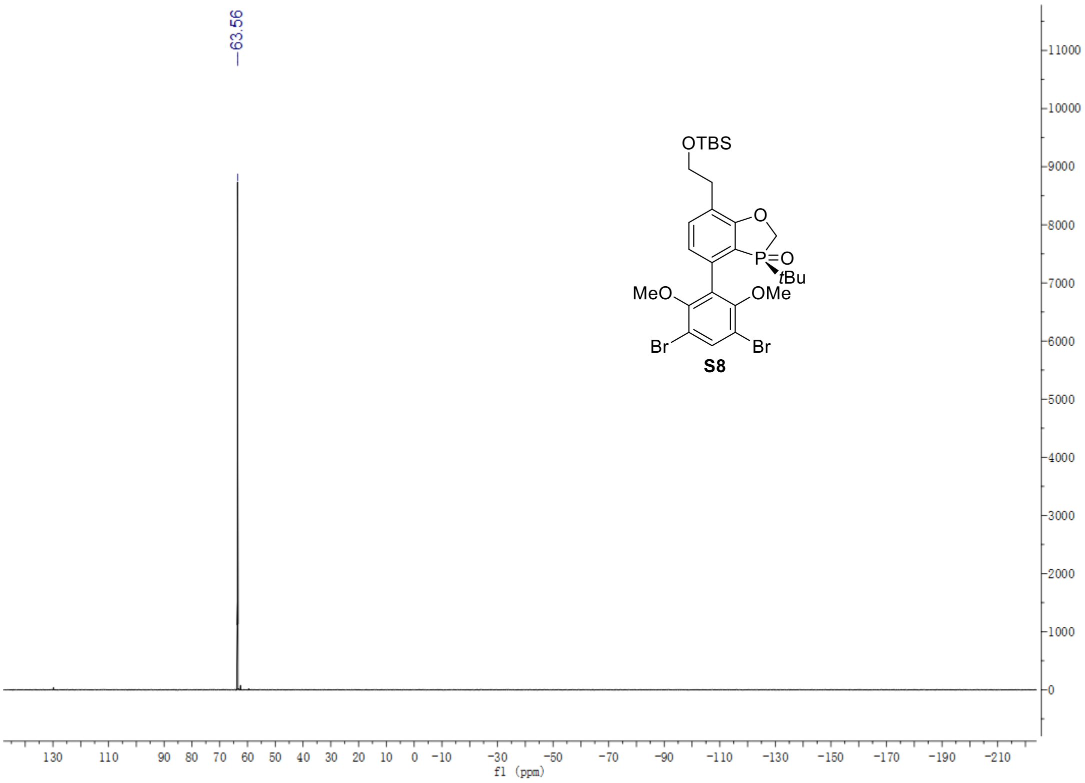

Supplementary Figure 25 ^{1}H NMR spectrum of **S5** (400 MHz, $CDCl_3$)

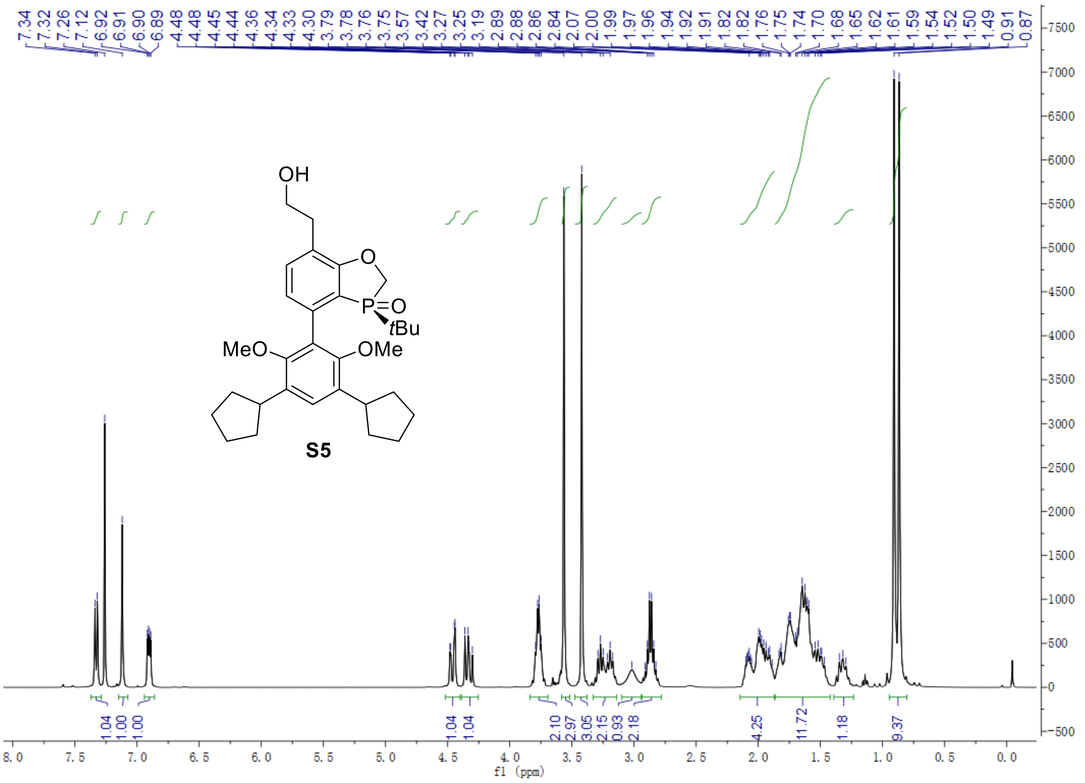

Supplementary Figure 26 ^{13}C NMR spectrum of **S5** (126 MHz, $CDCl_3$)

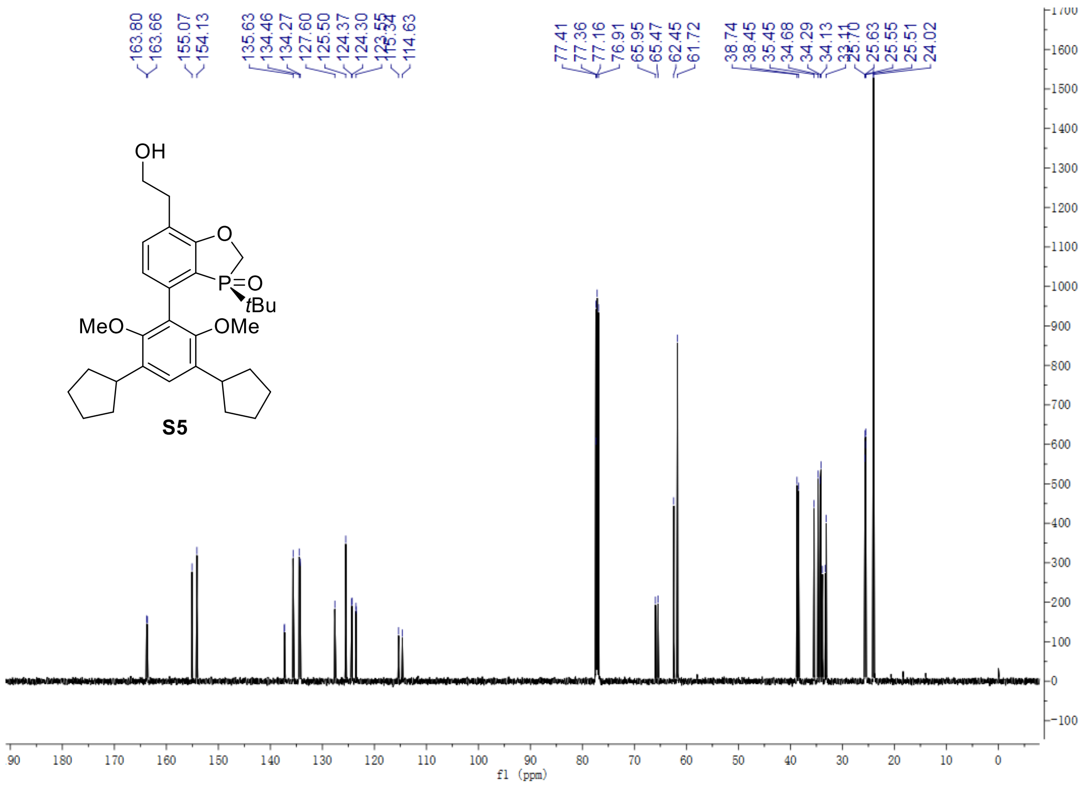

Supplementary Figure 27 ^{31}P NMR spectrum of **S5** (162 MHz, $CDCl_3$)

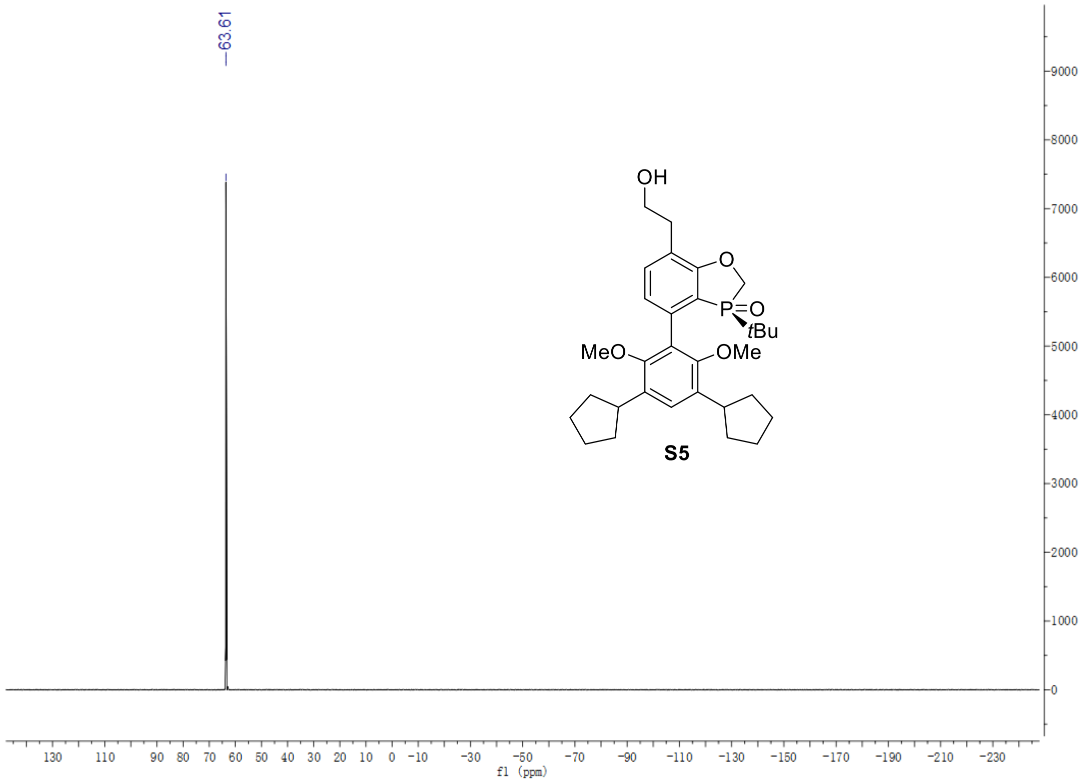

Supplementary Figure 28 ^{1}H NMR spectrum of **S6** (400 MHz, $CDCl_3$)

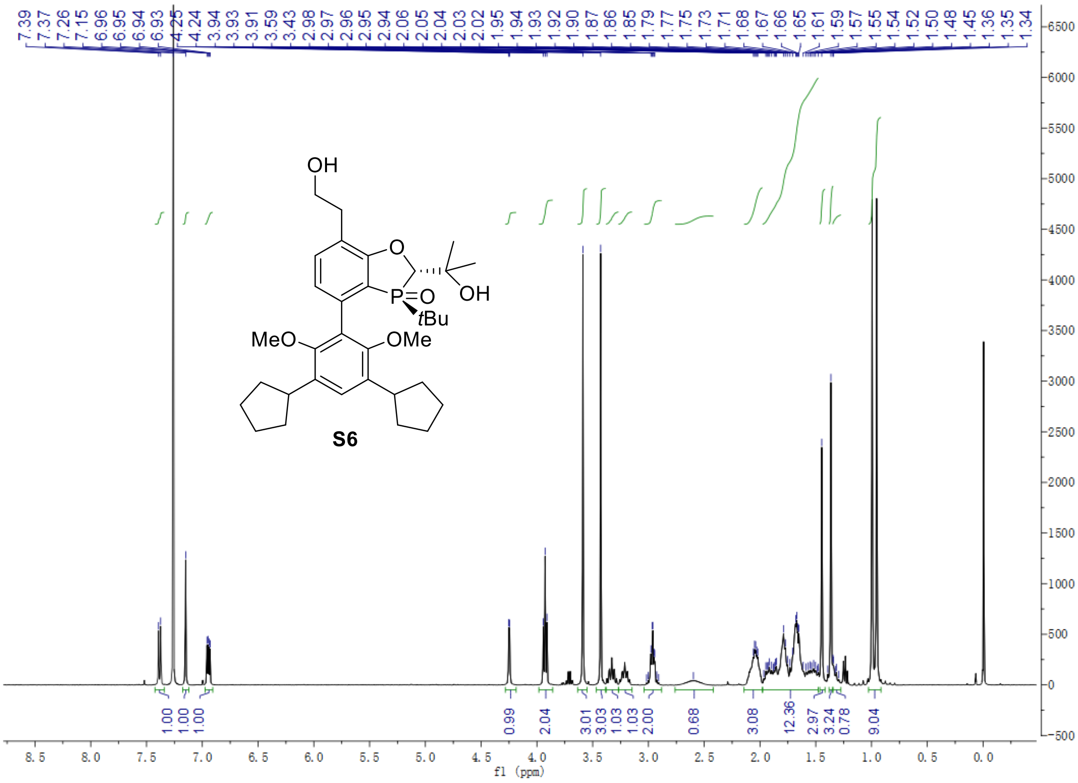

Supplementary Figure 29 ^{13}C NMR spectrum of **S6** (151 MHz, $CDCl_3$)

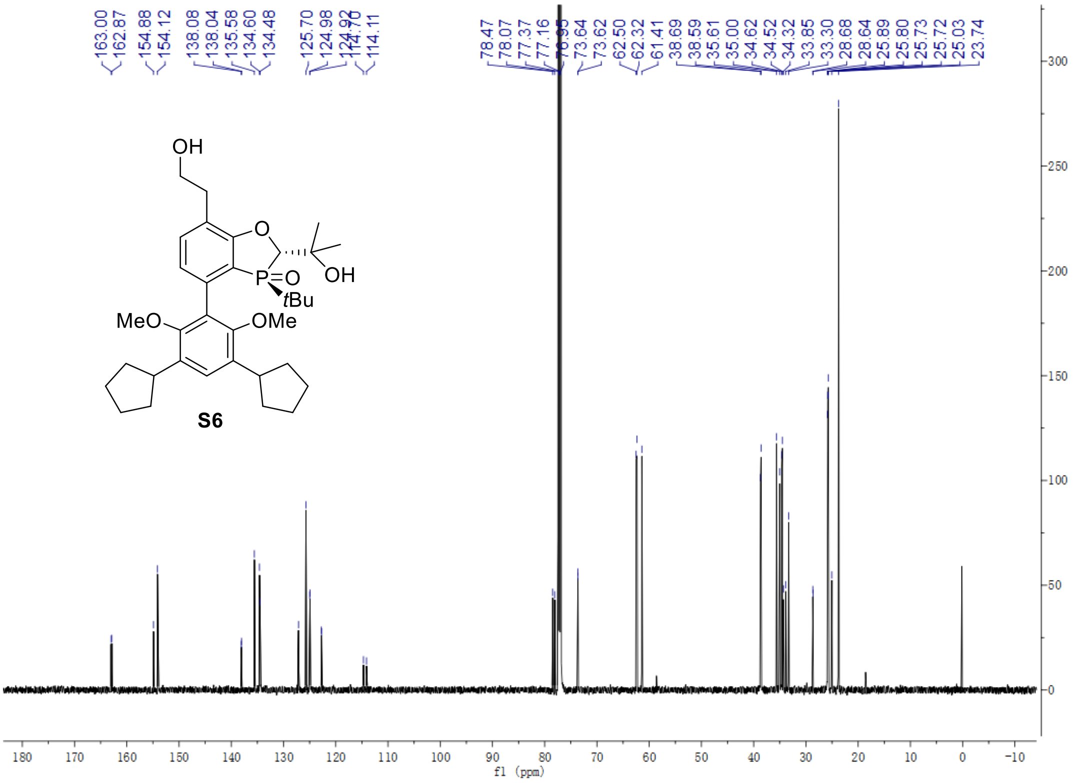

Supplementary Figure 30 ^{31}P NMR spectrum of **S6** (162 MHz, $CDCl_3$)

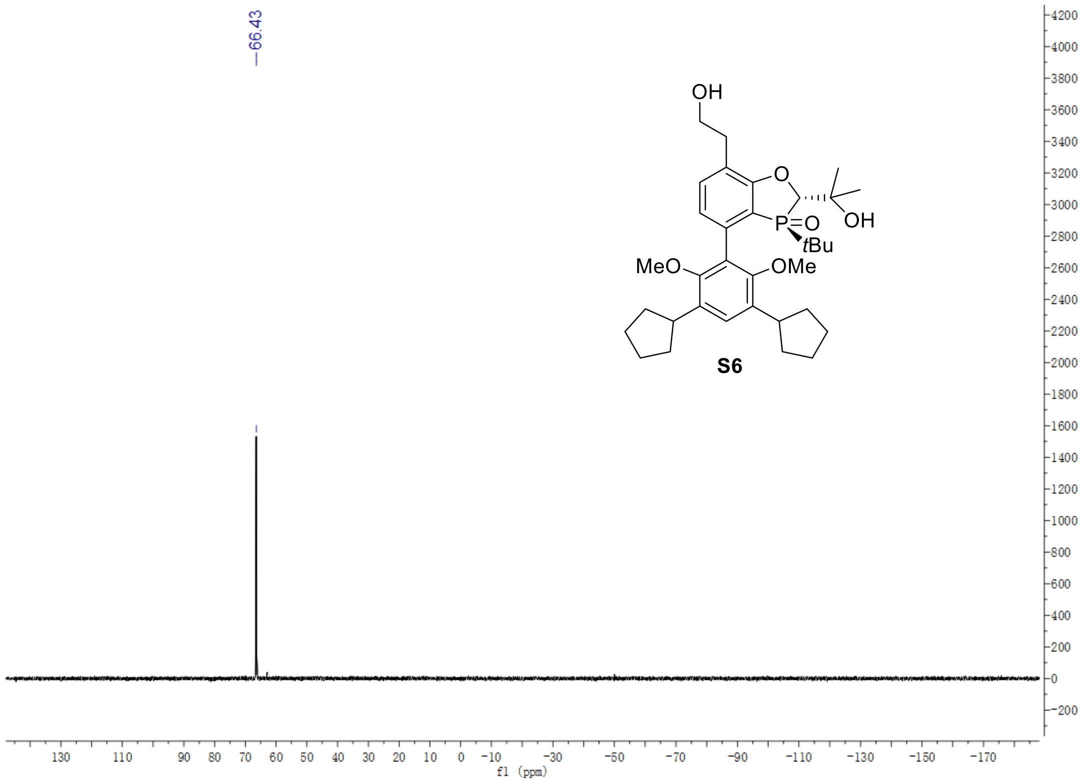

Supplementary Figure 31 ^{1}H NMR spectrum of **S7** (600 MHz, $CDCl_3$)

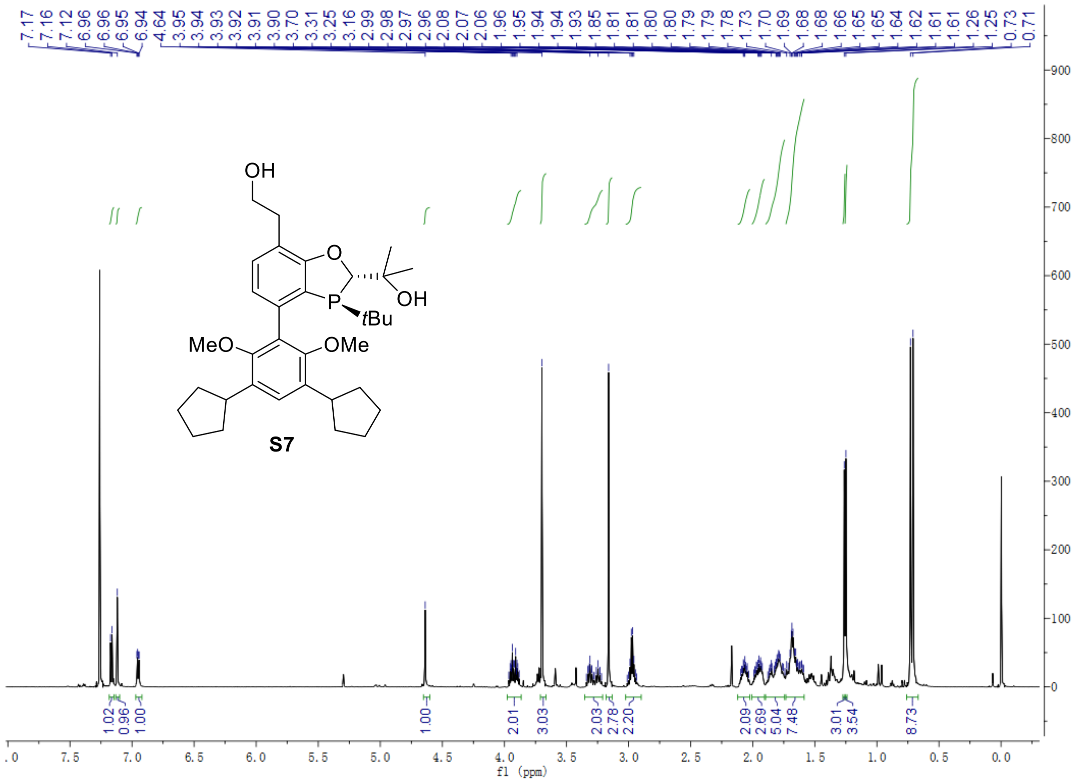

Supplementary Figure 32 ^{13}C NMR spectrum of **S7** (151 MHz, $CDCl_3$)

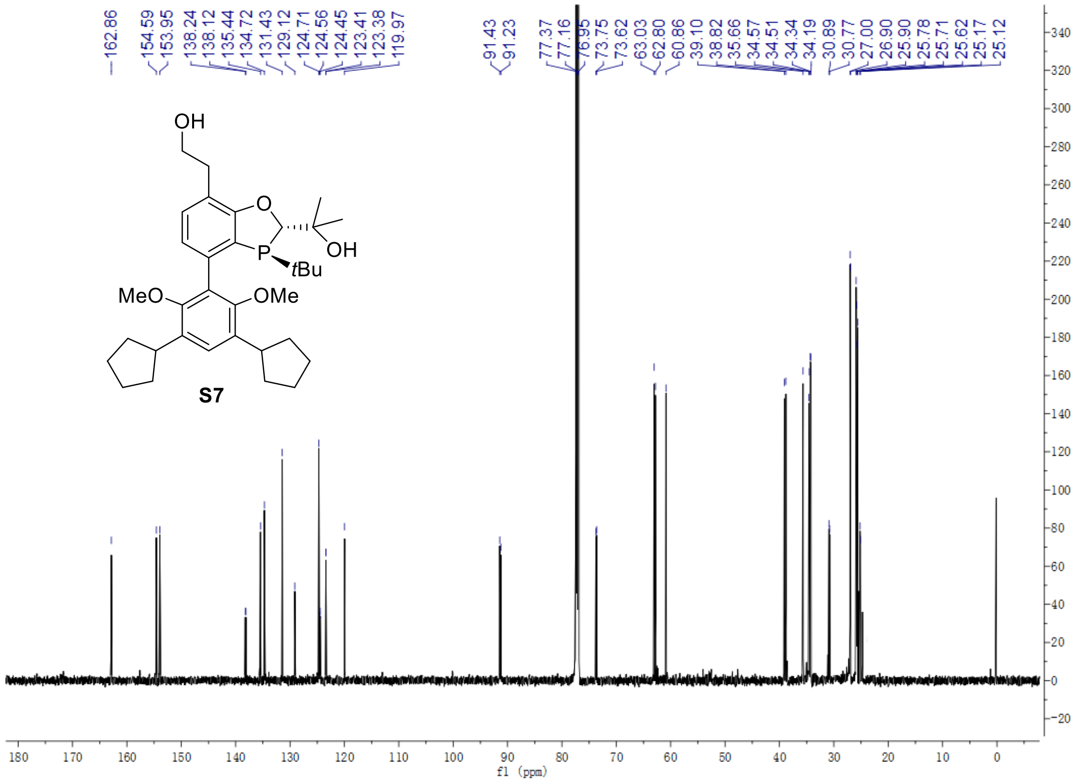

Supplementary Figure 33 ^{31}P NMR spectrum of **S7** (162 MHz, $CDCl_3$)

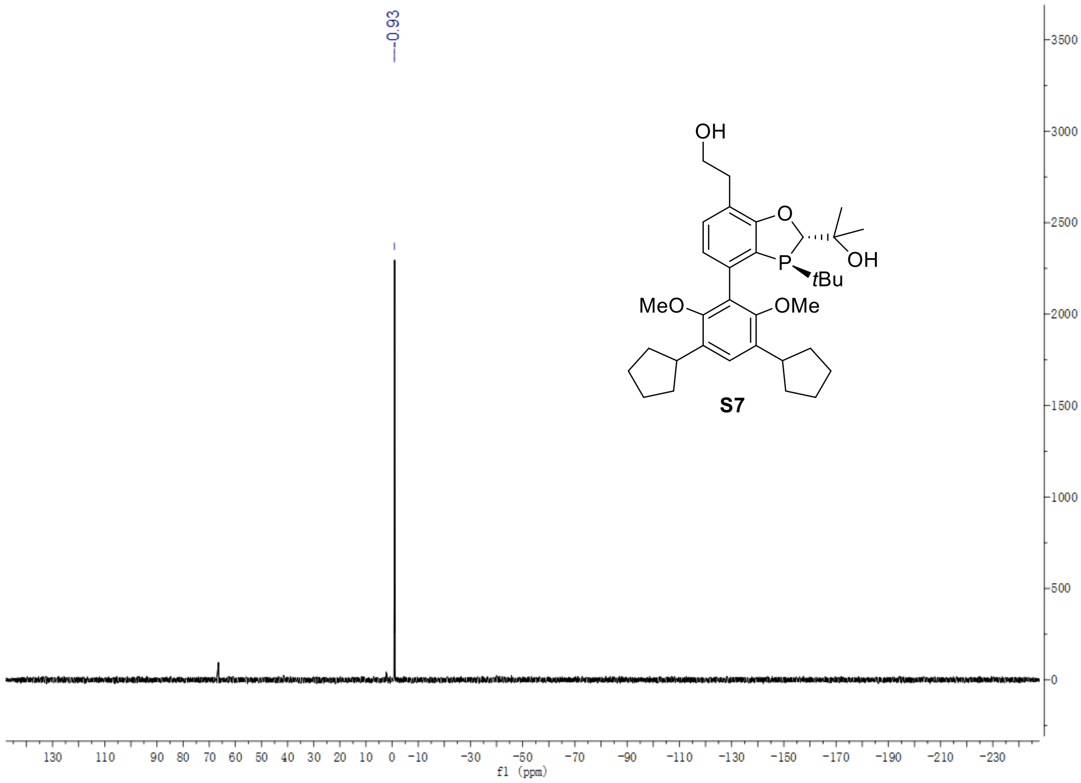

Supplementary Figure 34 ^{31}P NMR spectrum of **L2** (162 MHz, $CDCl_3$)

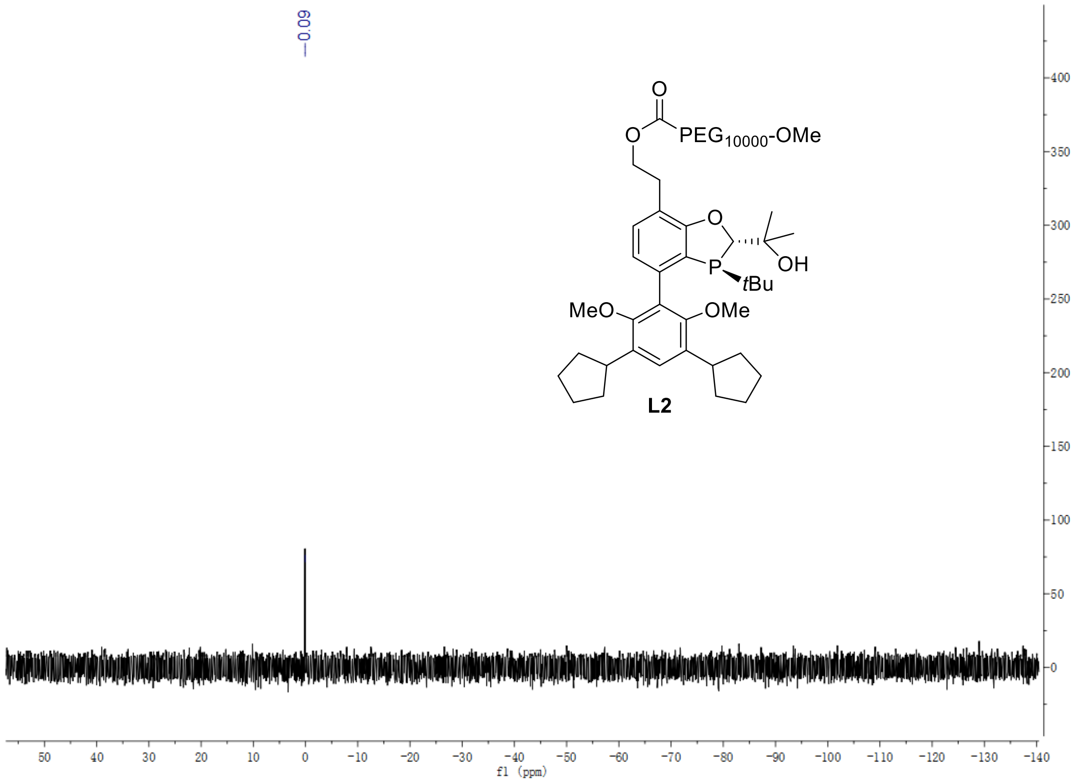

Supplementary Figure 35 ^{31}P NMR spectrum of **L3** (162 MHz, $CDCl_3$)

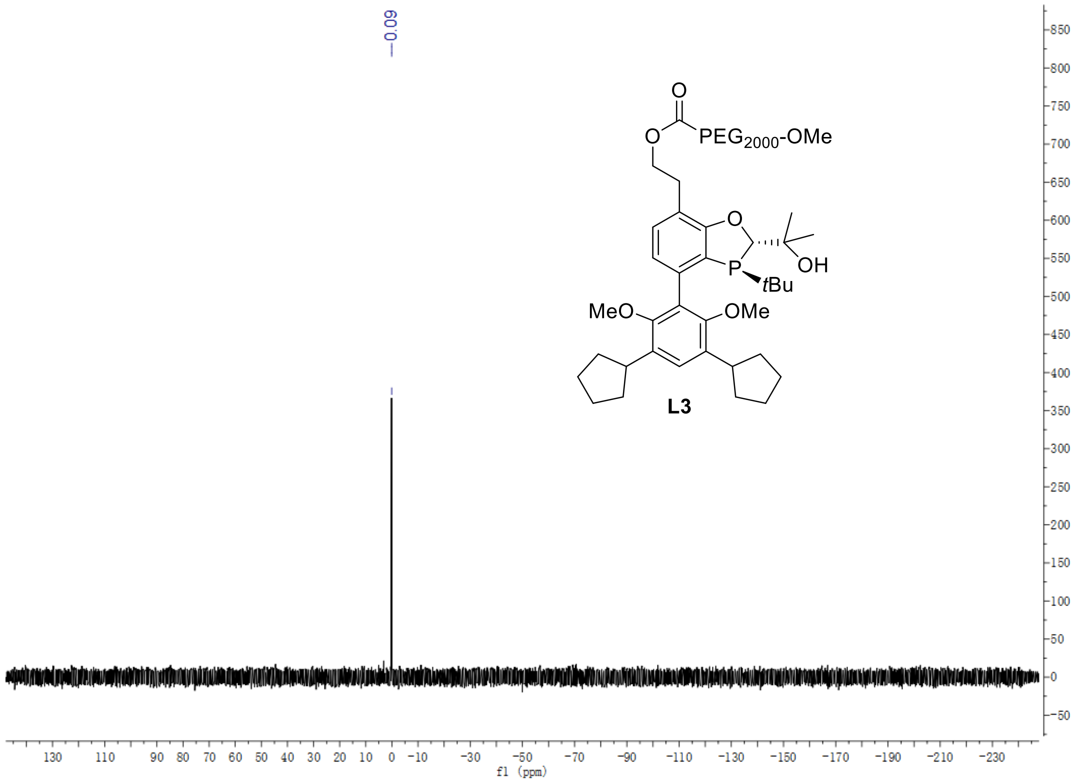

Supplementary Figure 36 ^{1}H NMR spectrum of **S9** (400 MHz, $CDCl_3$)

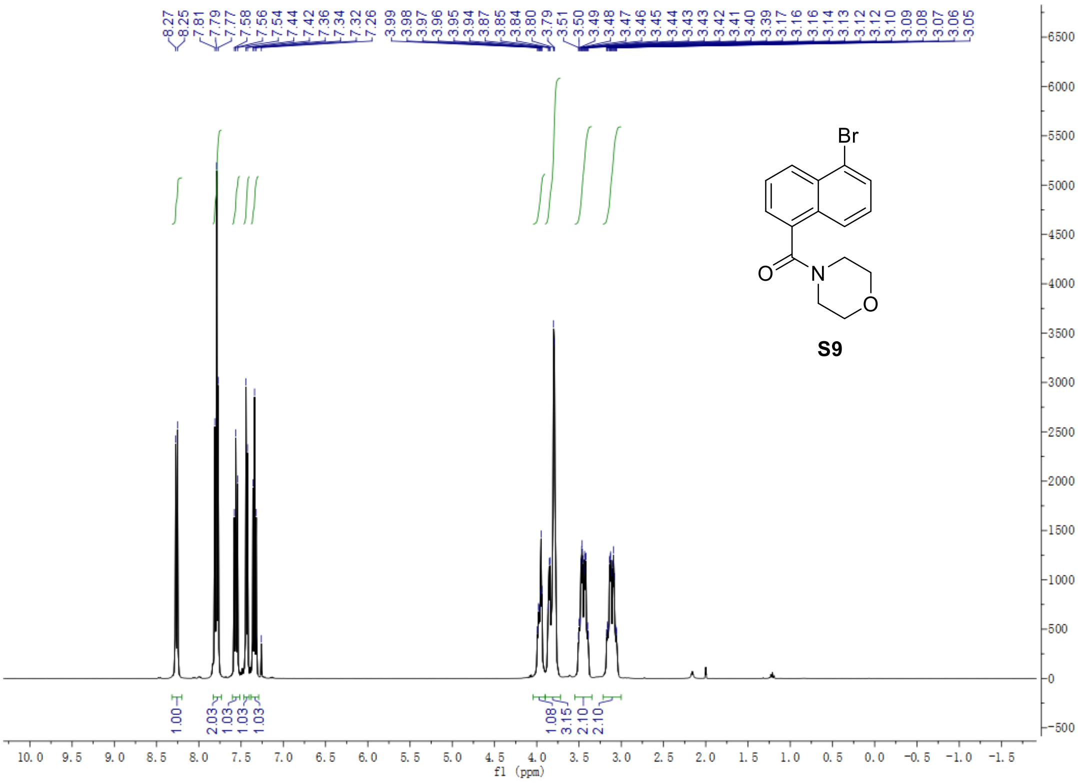

Supplementary Figure 37 ^{13}C NMR spectrum of **S9** (101 MHz, $CDCl_3$)

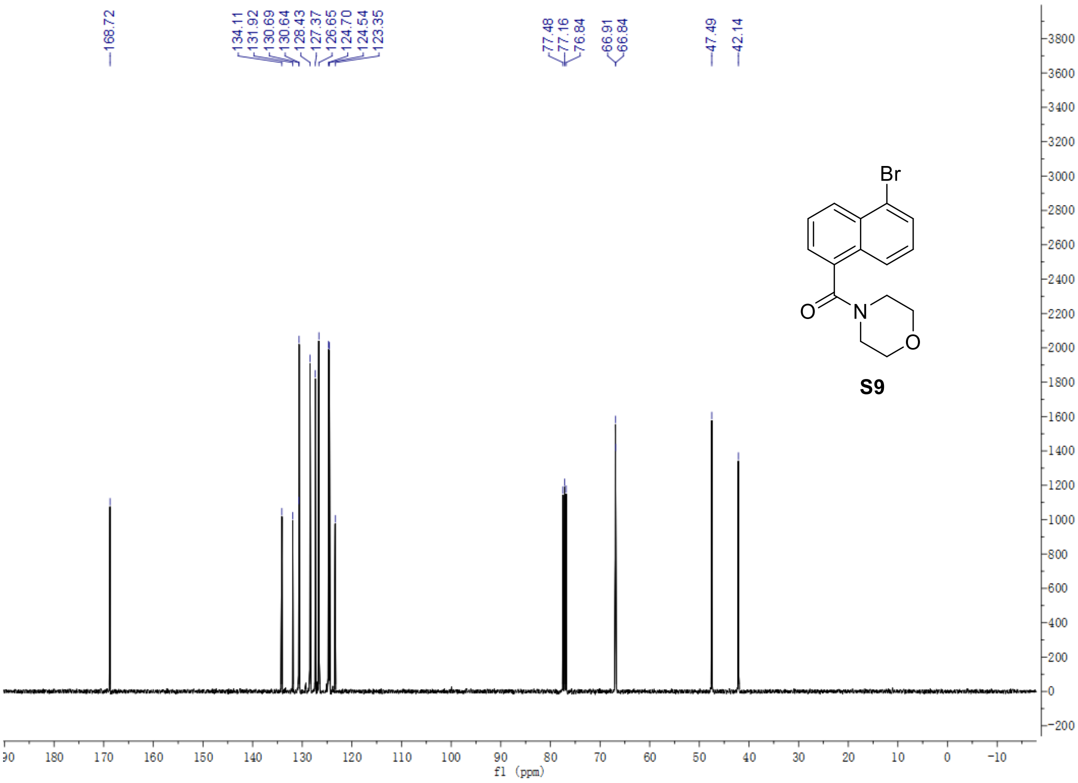

Supplementary Figure 38 ^{1}H NMR spectrum of **S10** (600 MHz, DMSO-d_6)

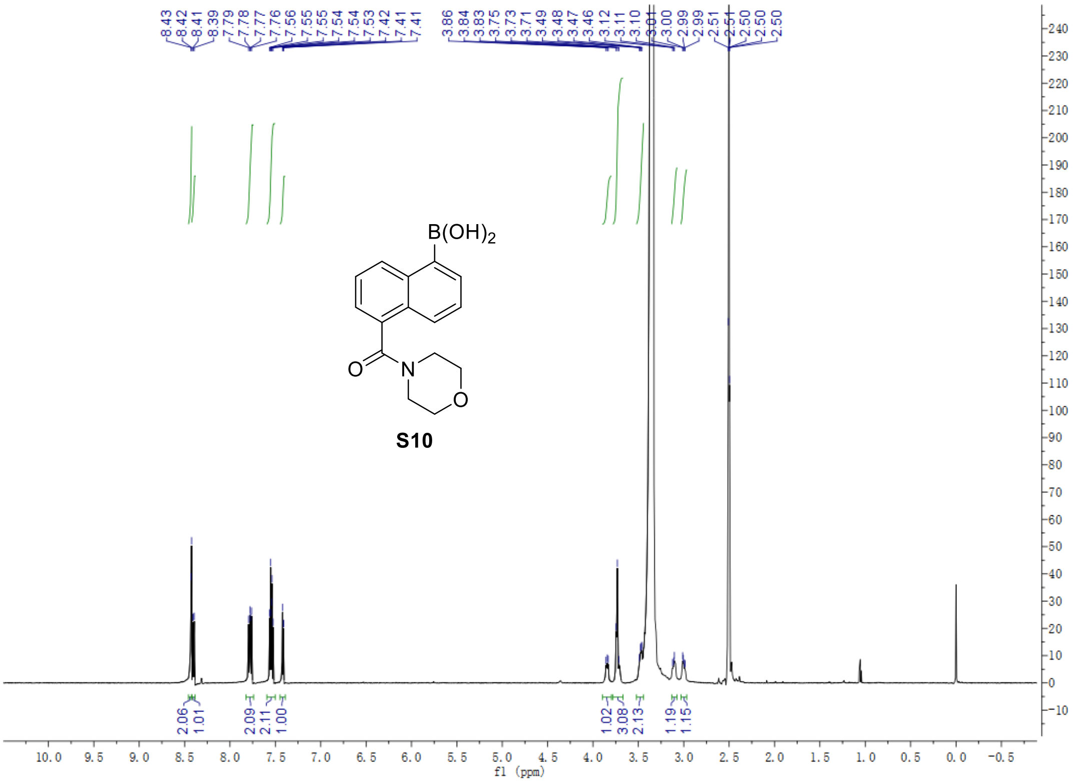

Supplementary Figure 39 ^{13}C NMR spectrum of **S10** (151 MHz, DMSO-d_6)

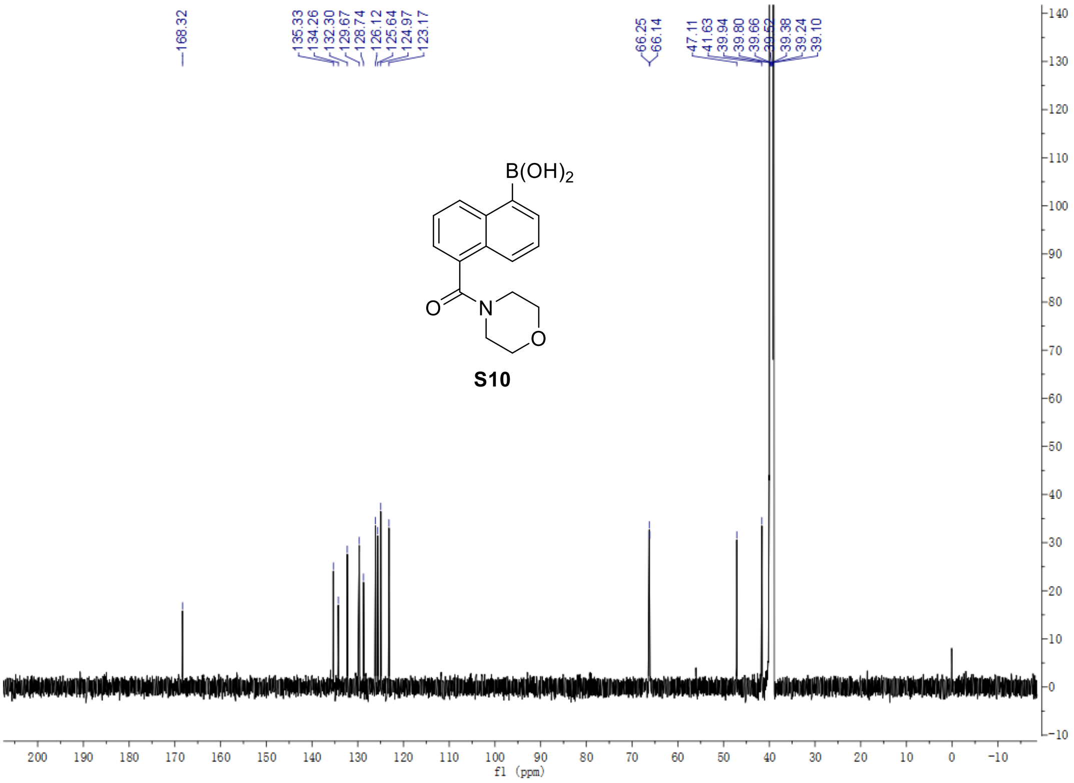

Supplementary Figure 40 ^{1}H NMR spectrum of **S11** (500 MHz, $CDCl_3$)

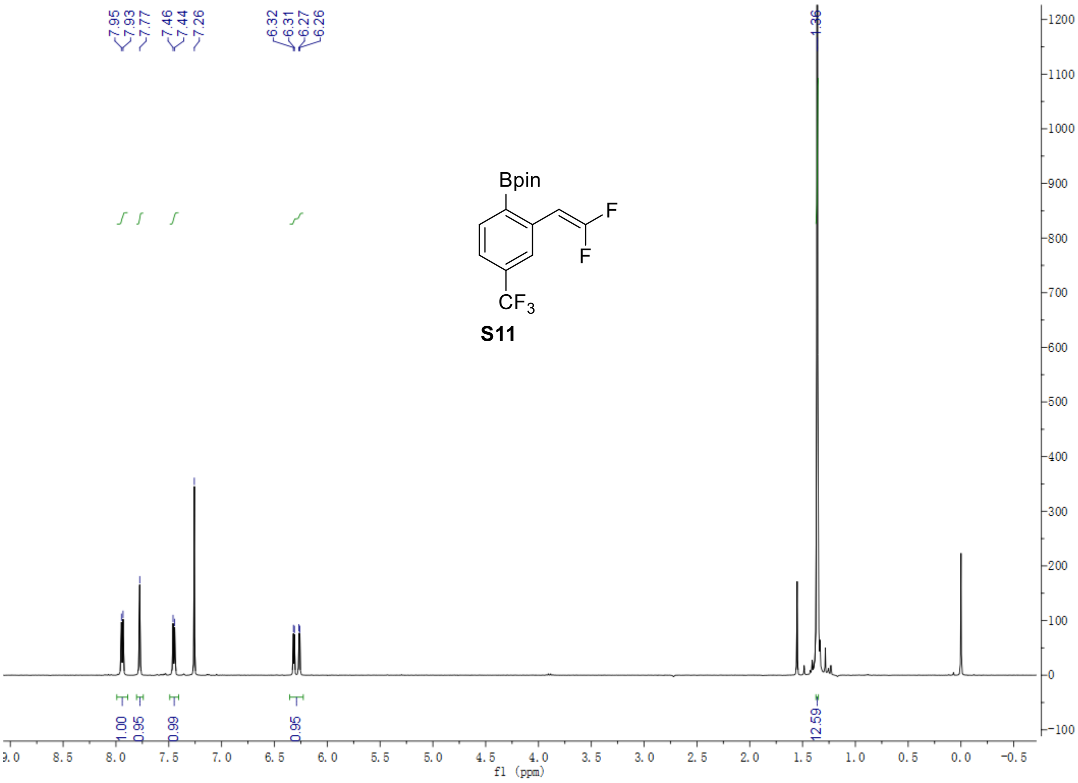

Supplementary Figure 41 ^{13}C NMR spectrum of **S11** (151 MHz, $CDCl_3$)

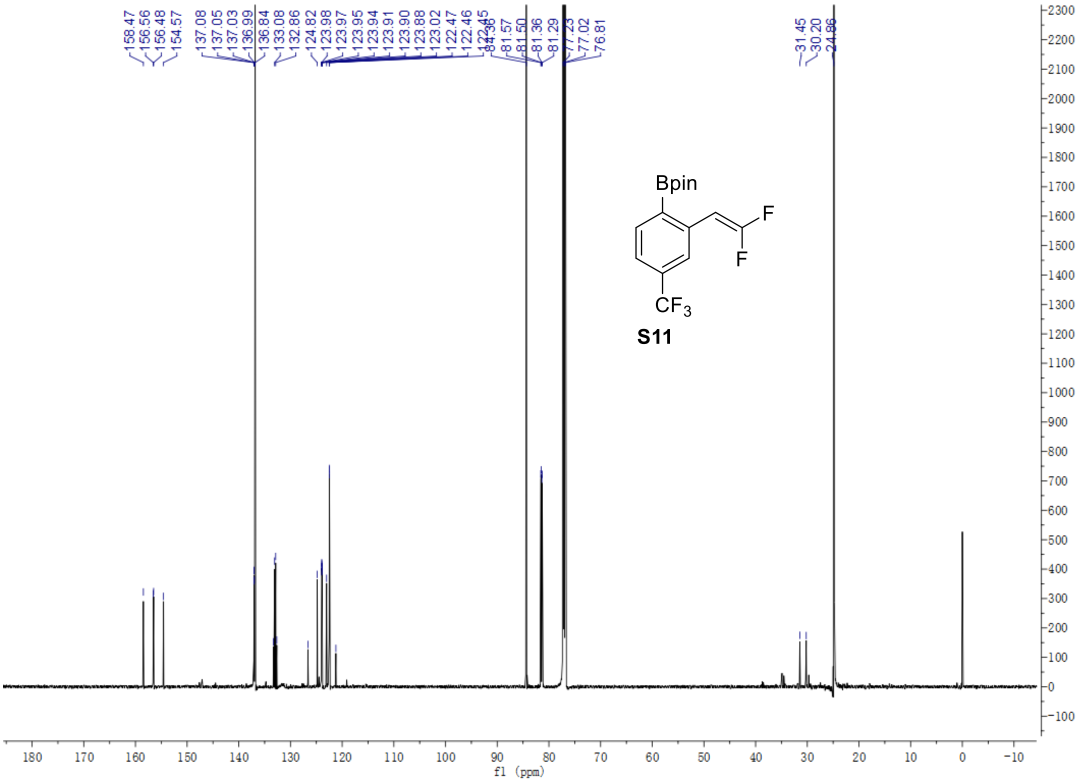

Supplementary Figure 42 ^{19}F NMR spectrum of **S11** (376 MHz, $CDCl_3$)

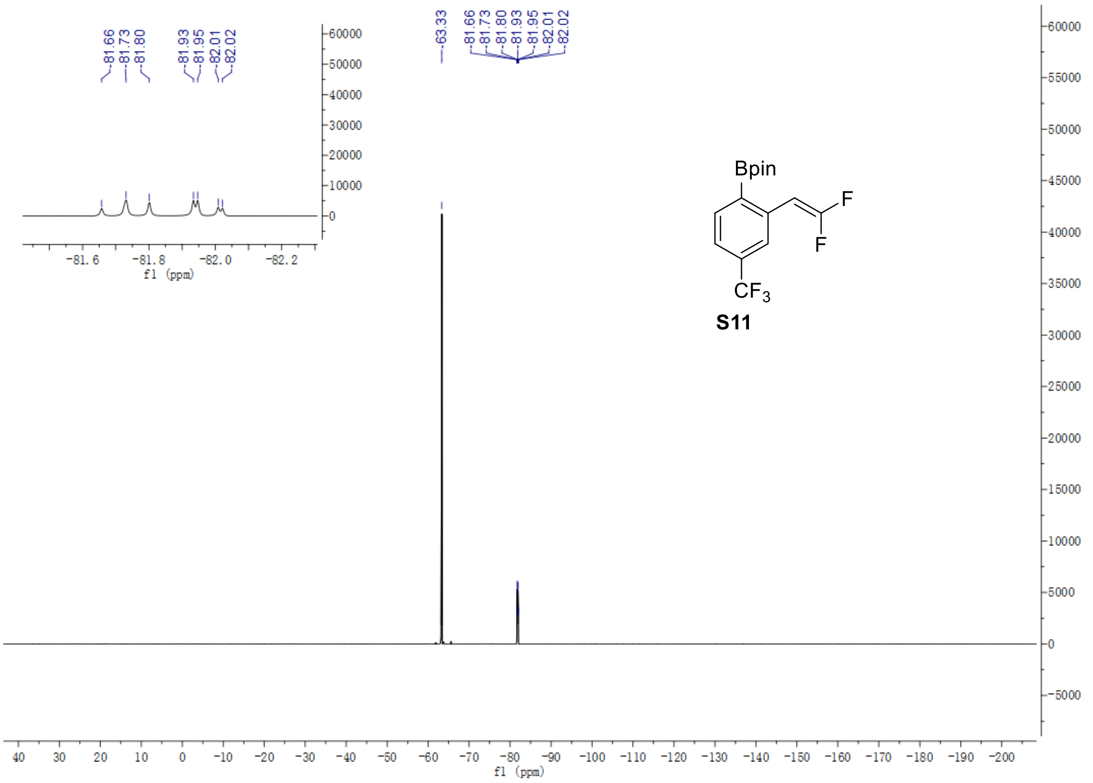

Supplementary Figure 43 ^{1}H NMR spectrum of **S14** (500 MHz, $CDCl_3$)

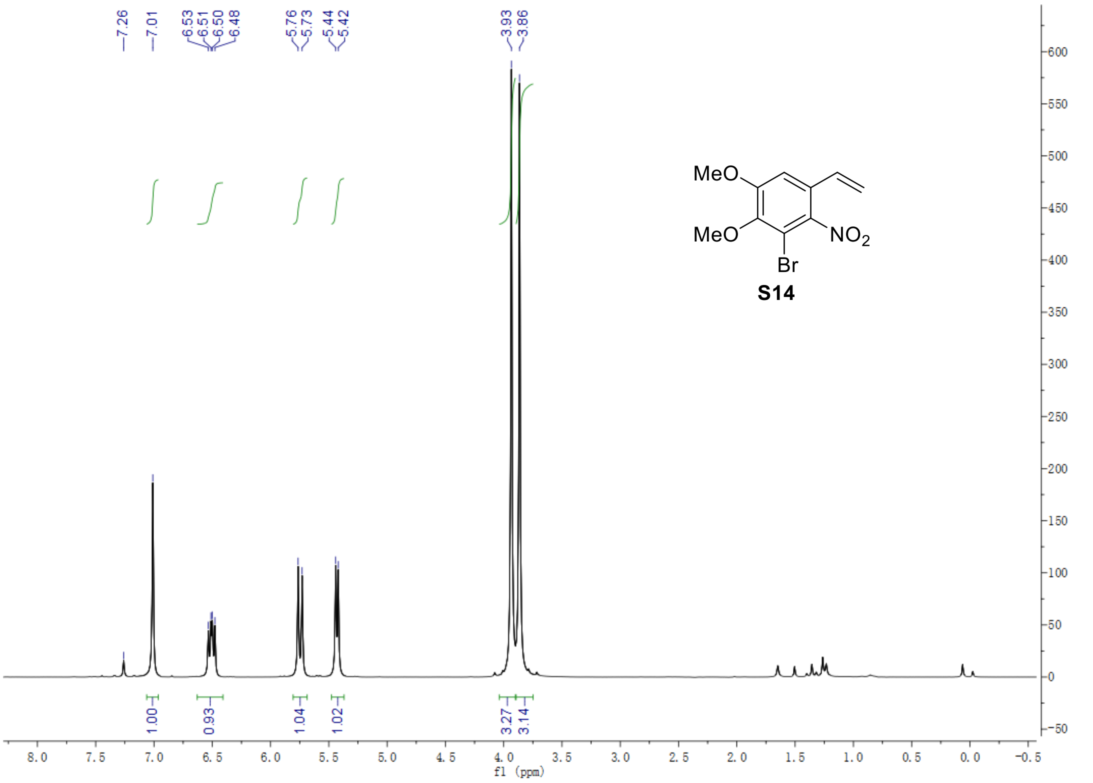

Supplementary Figure 44 ^{13}C NMR spectrum of **S14** (126 MHz, $CDCl_3$)

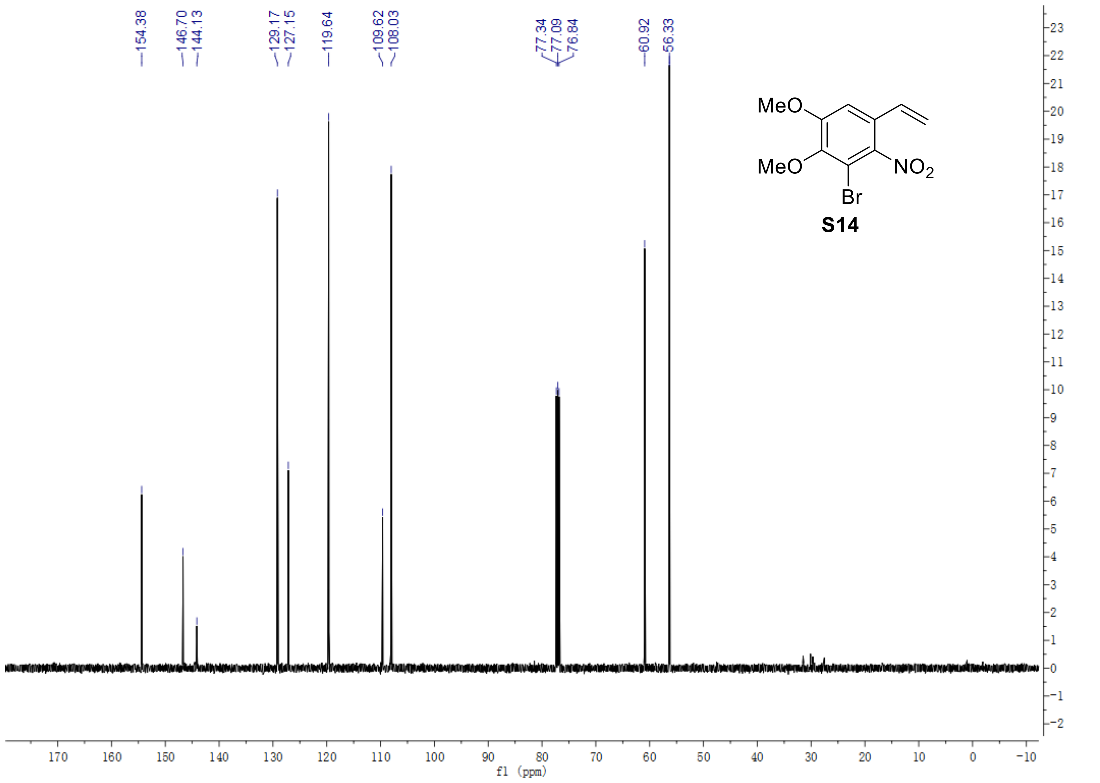

Supplementary Figure 45 ^{1}H NMR spectrum of **S15** (400 MHz, $CDCl_3$)

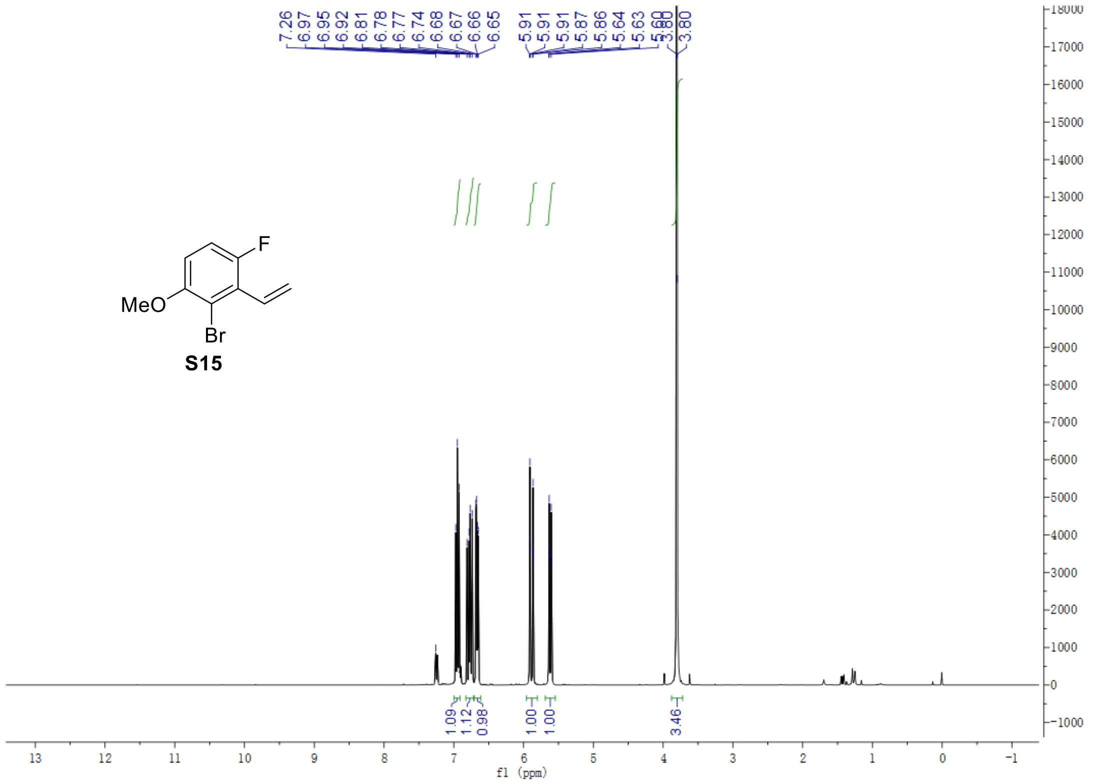

Supplementary Figure 46 ^{13}C NMR spectrum of **S15** (126 MHz, $CDCl_3$)

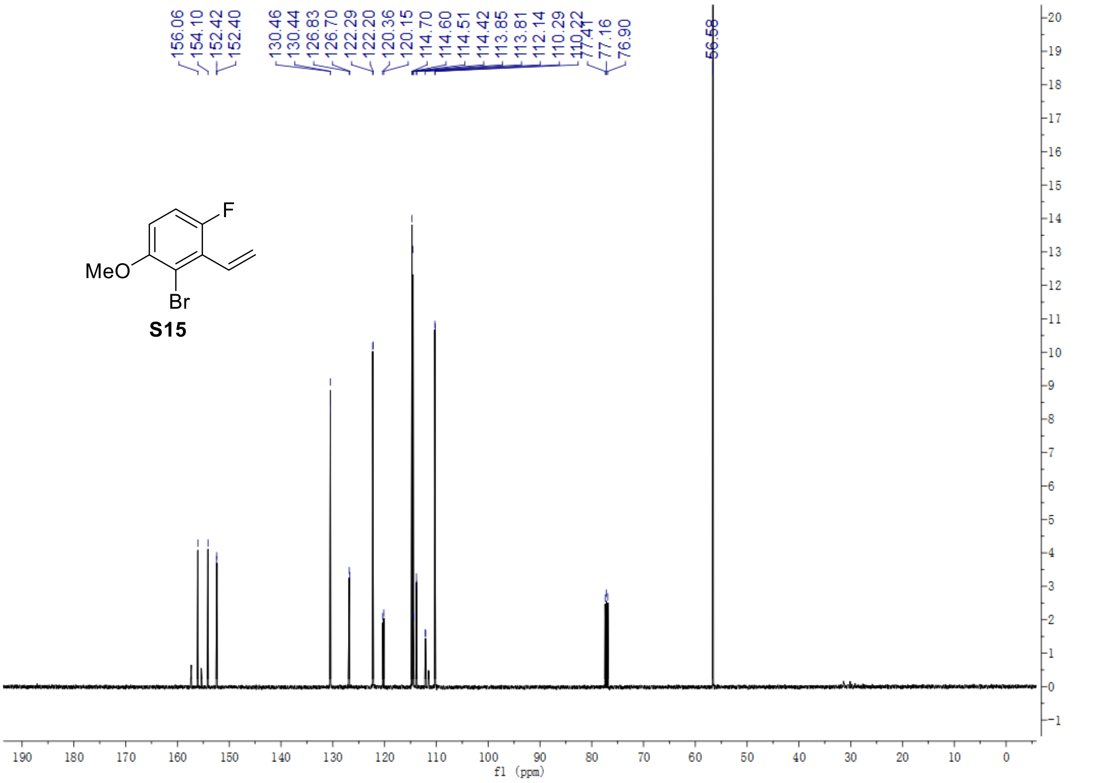

Supplementary Figure 47 ^{19}F NMR spectrum of **S15** (376 MHz, $CDCl_3$)

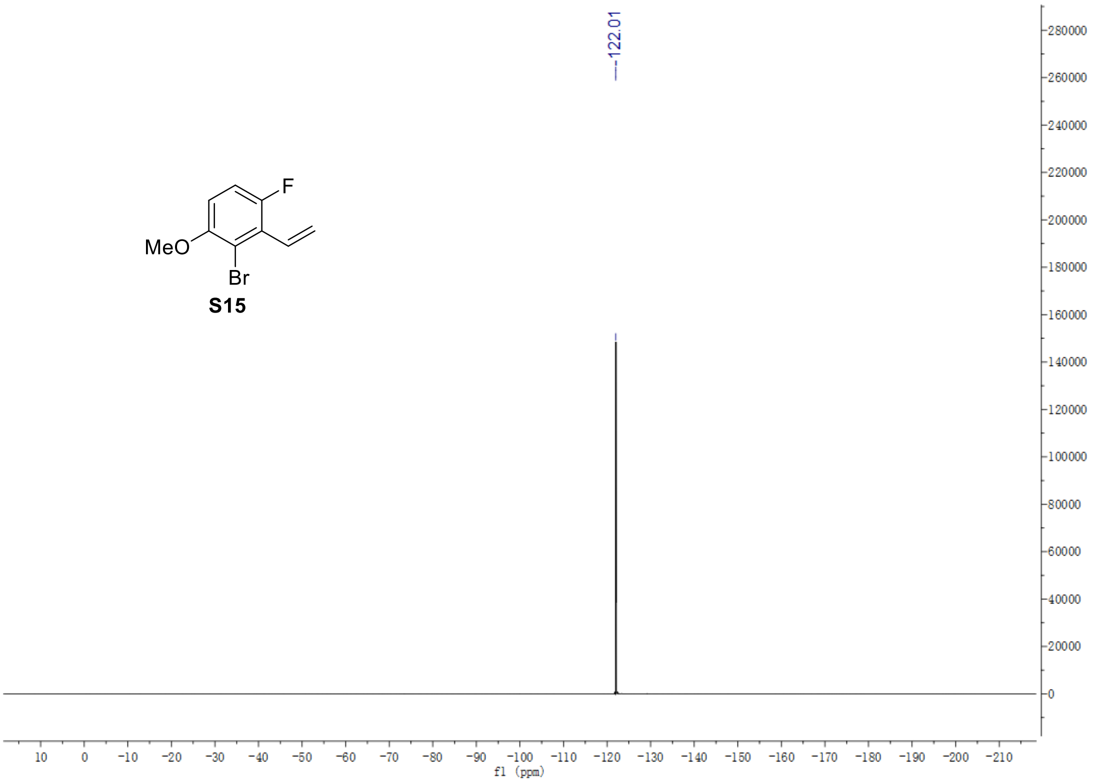

Supplementary Figure 48 ^{1}H NMR spectrum of **S17** (500 MHz, $CDCl_3$)

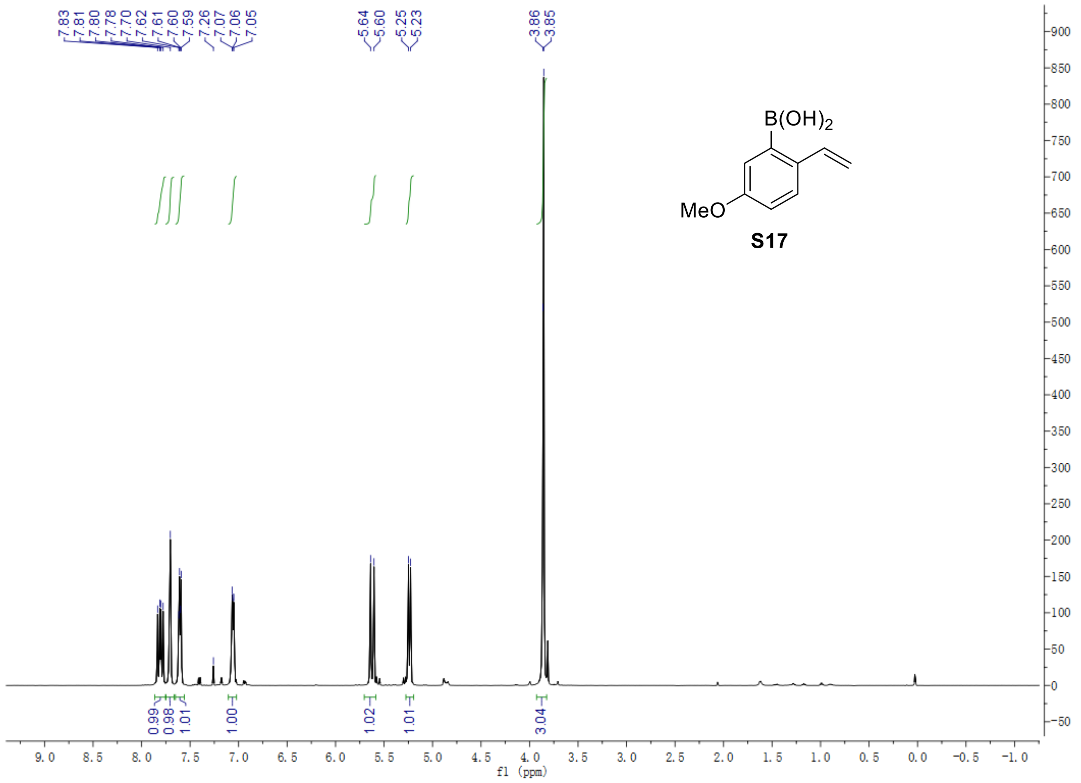

Supplementary Figure 49 ^{13}C NMR spectrum of **S17** (126 MHz, $CDCl_3$)

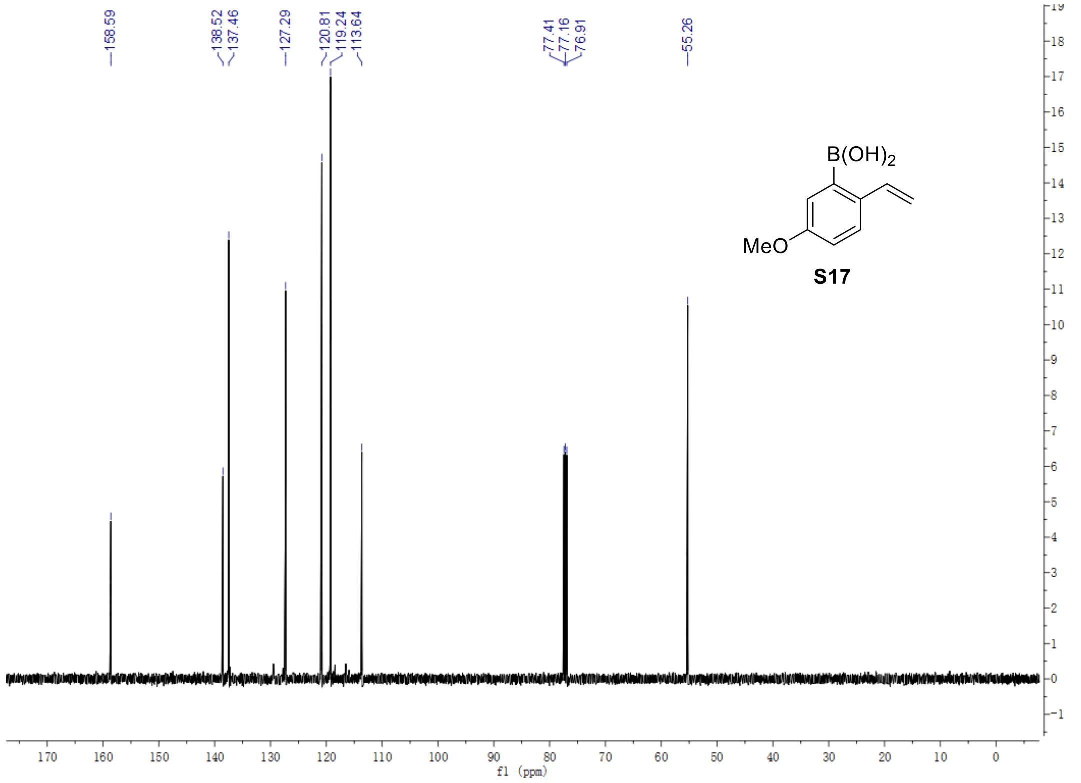

Supplementary Figure 50 ^{1}H NMR spectrum of **S18** (500 MHz, DMSO-d_6)

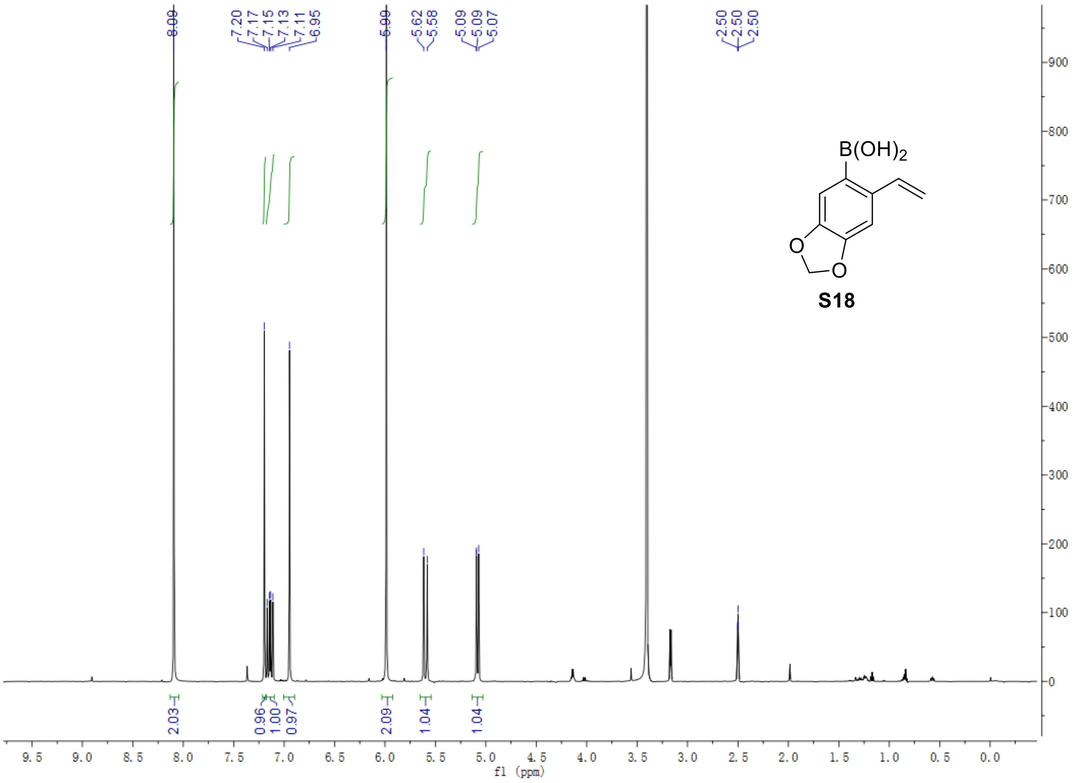

Supplementary Figure 51 ^{13}C NMR spectrum of **S18** (126 MHz, DMSO-d_6)

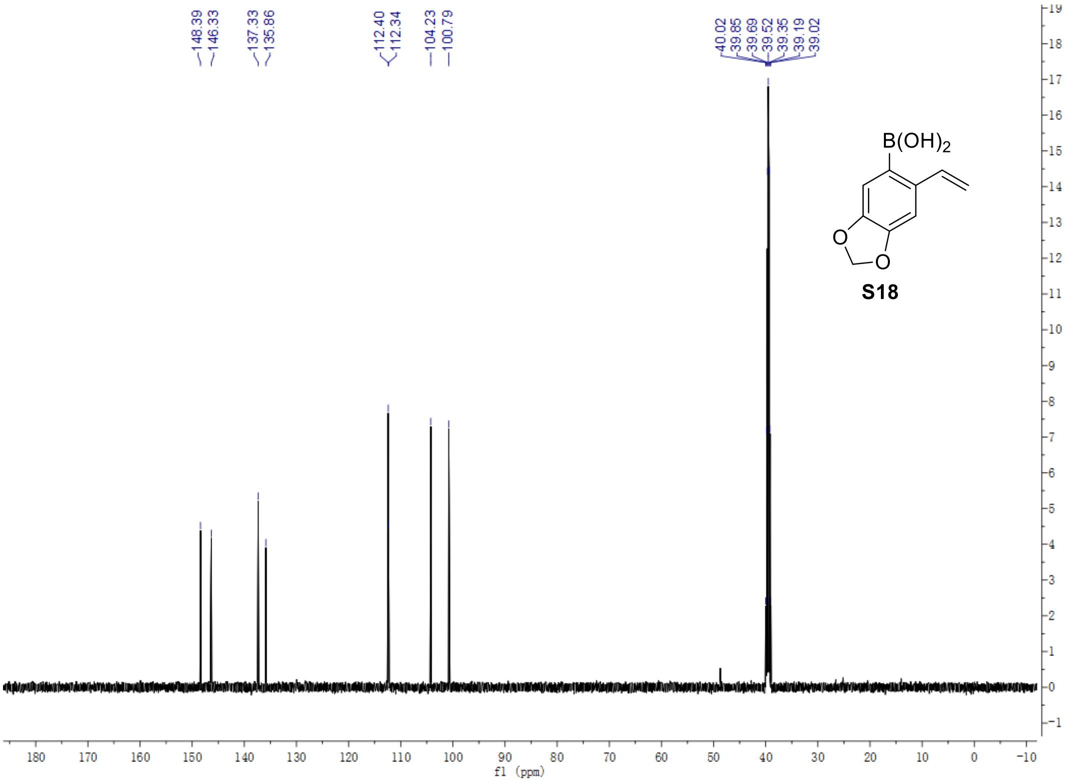

Supplementary Figure 52 ^{1}H NMR spectrum of **S19** (400 MHz, $CDCl_3$)

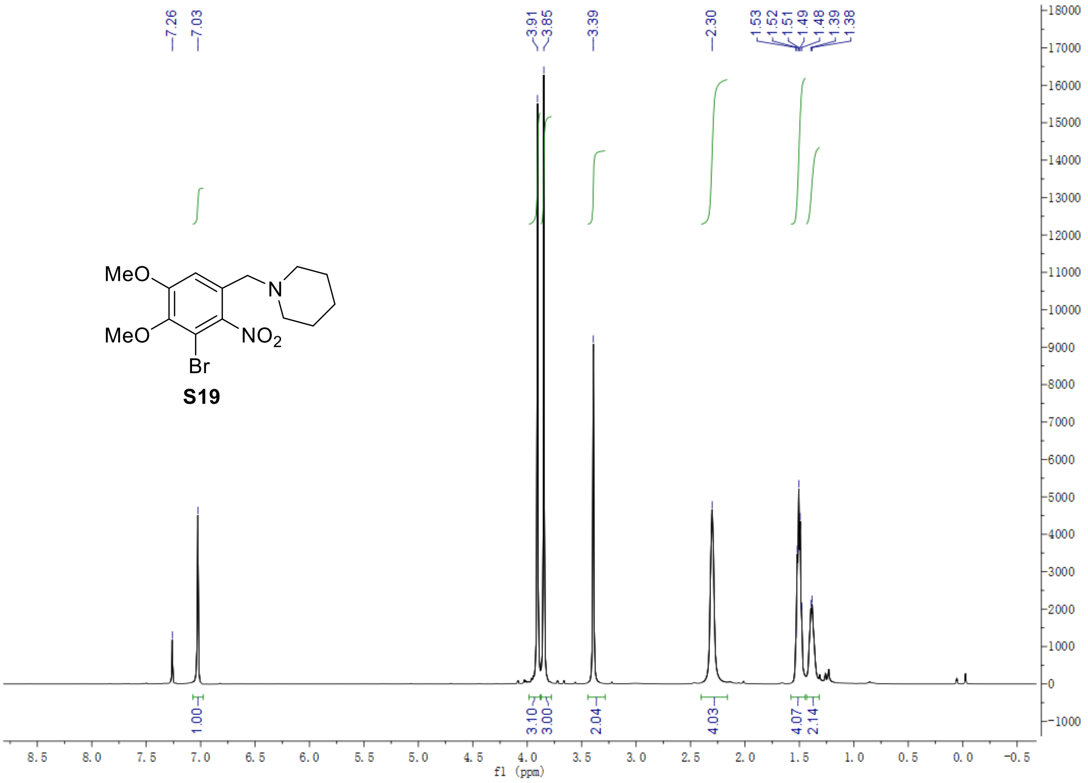

Supplementary Figure 53 ^{13}C NMR spectrum of **S19** (126 MHz, $CDCl_3$)

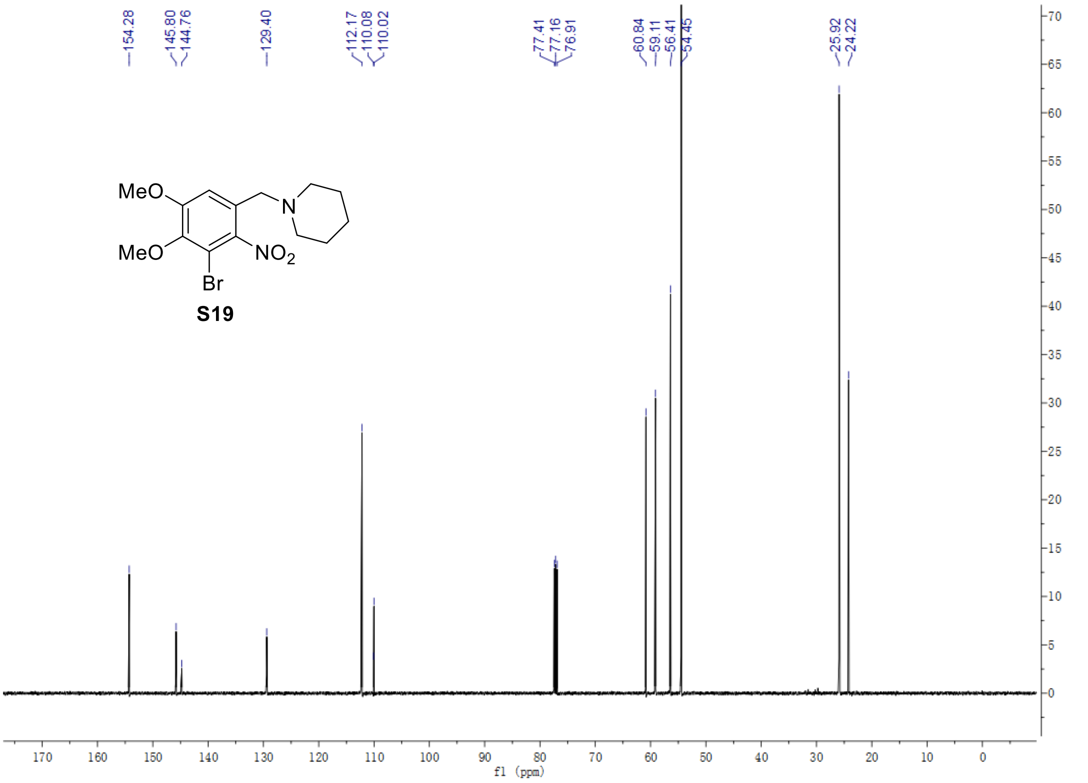

Supplementary Figure 54 ^{1}H NMR spectrum of **S20** (500 MHz, $CDCl_3$)

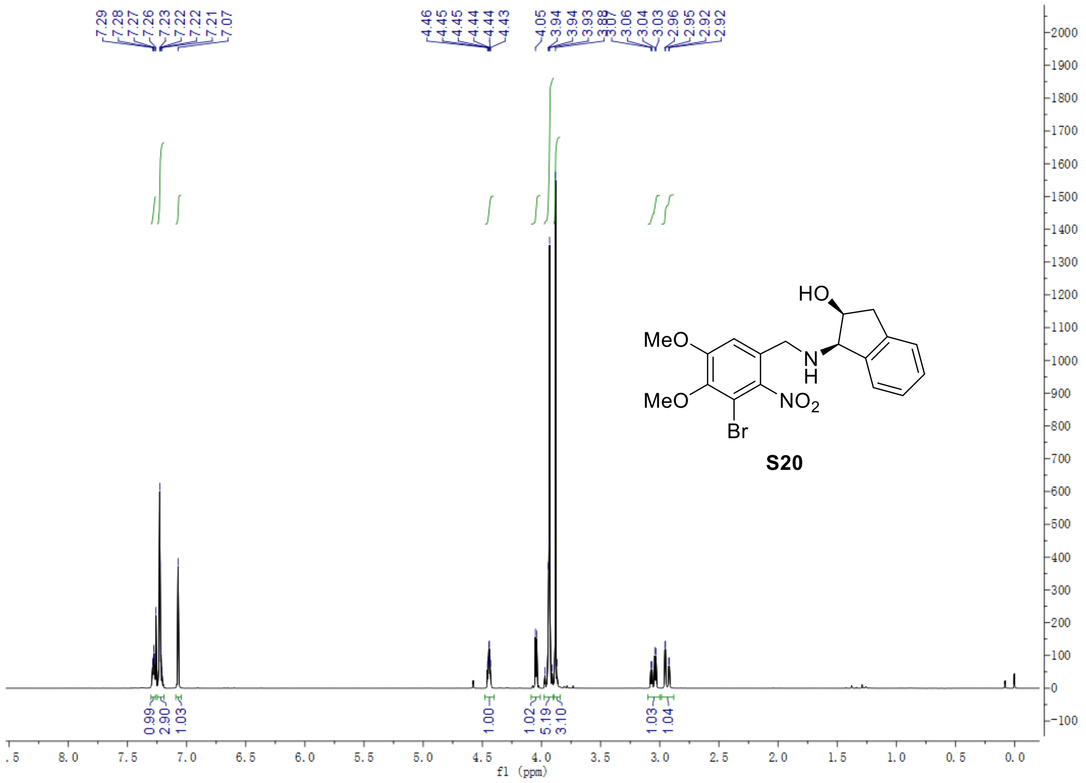

Supplementary Figure 55 ^{13}C NMR spectrum of **S20** (126 MHz, $CDCl_3$)

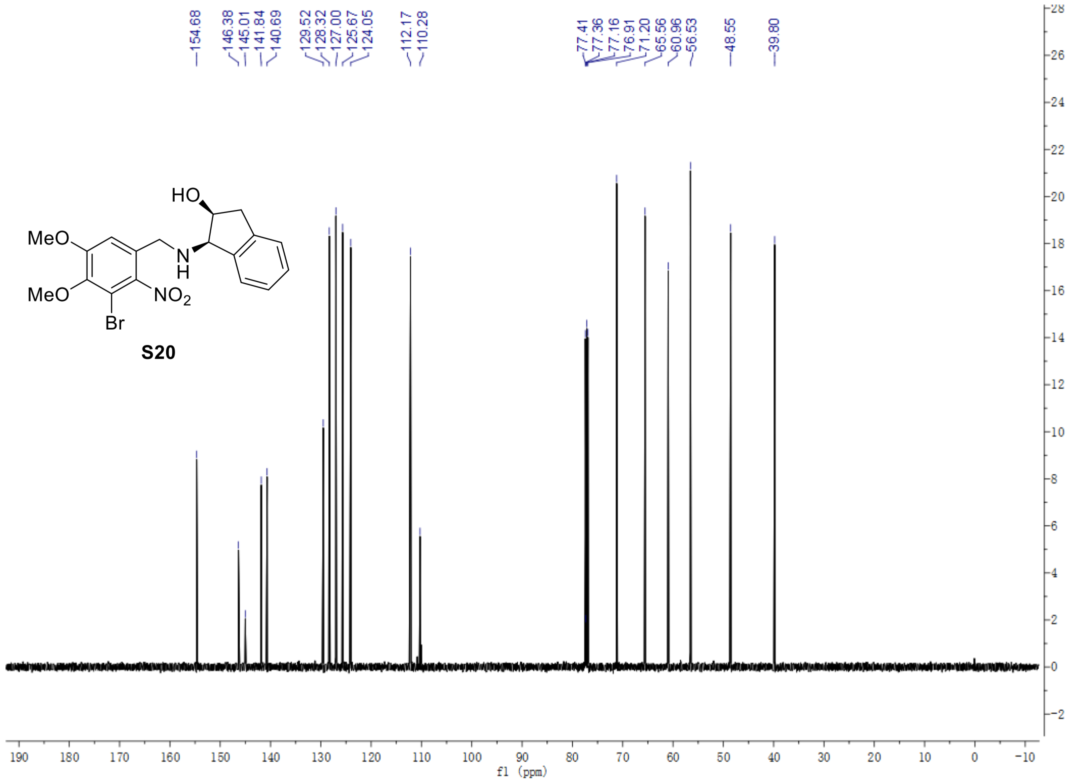

Supplementary Figure 56 ^{1}H NMR spectrum of **S21** (500 MHz, DMSO-d_6)

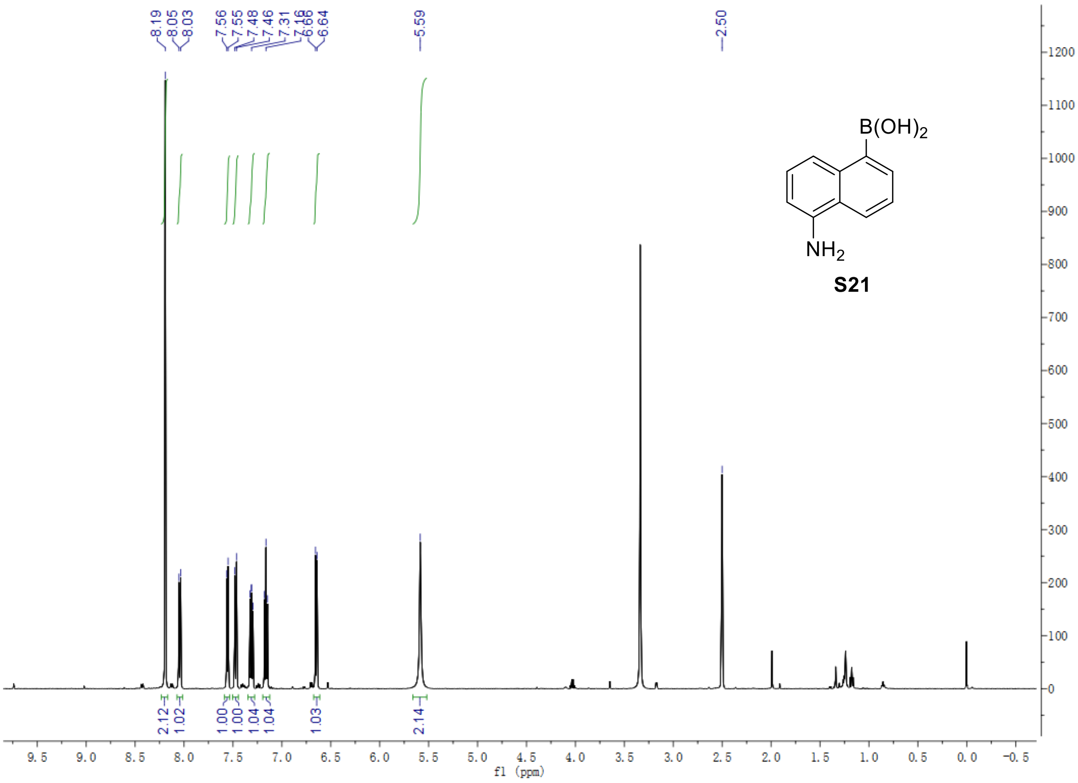

Supplementary Figure 57 ^{13}C NMR spectrum of **S21** (151 MHz, DMSO-d_6)

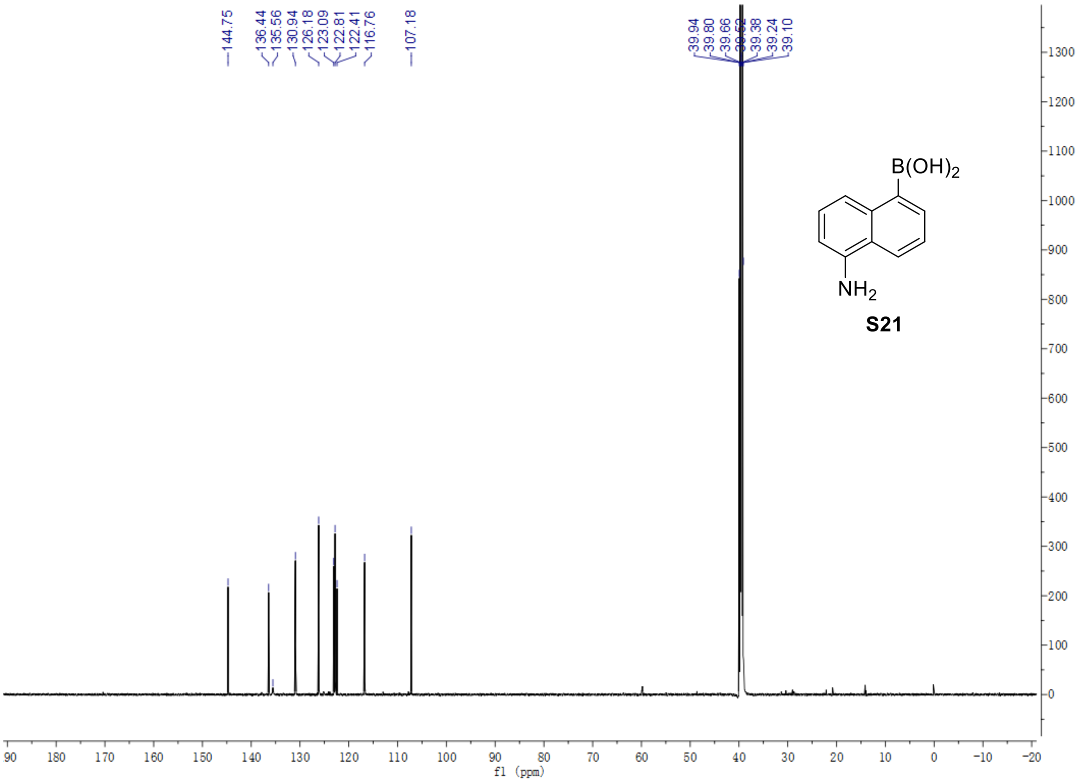

Supplementary Figure 58 ^{1}H NMR spectrum of **S22** (400 MHz, $CDCl_3$)

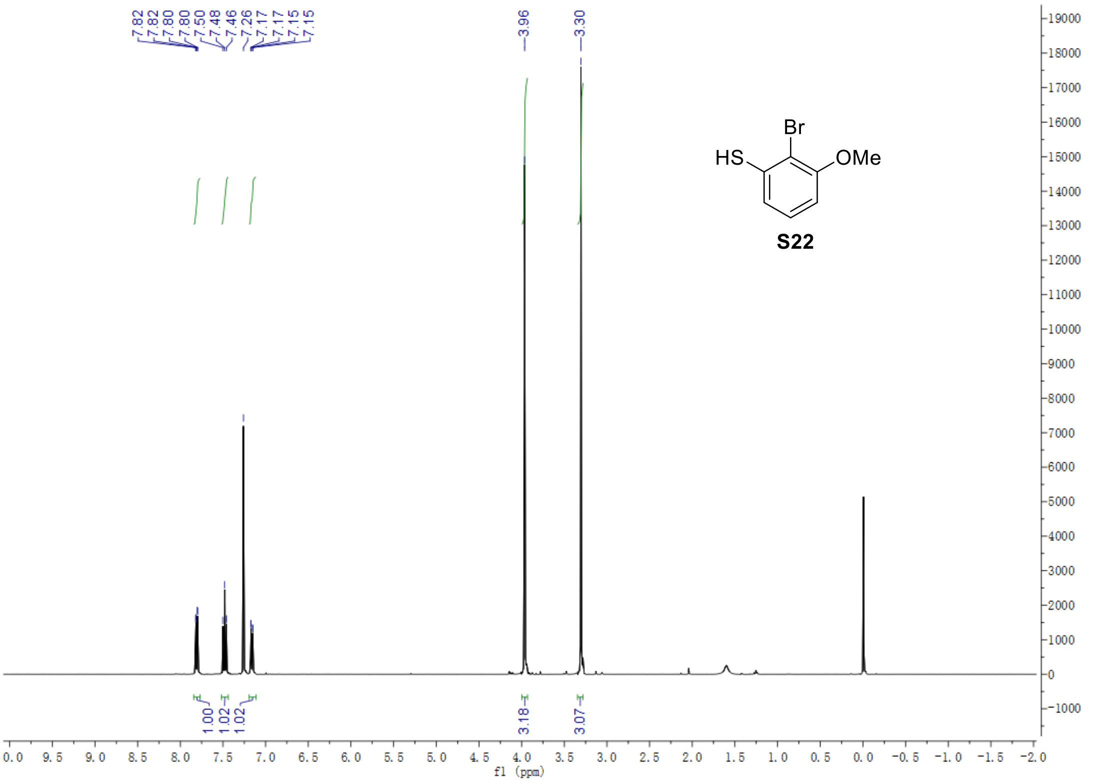

Supplementary Figure 59 ^{13}C NMR spectrum of **S22** (126 MHz, $CDCl_3$)

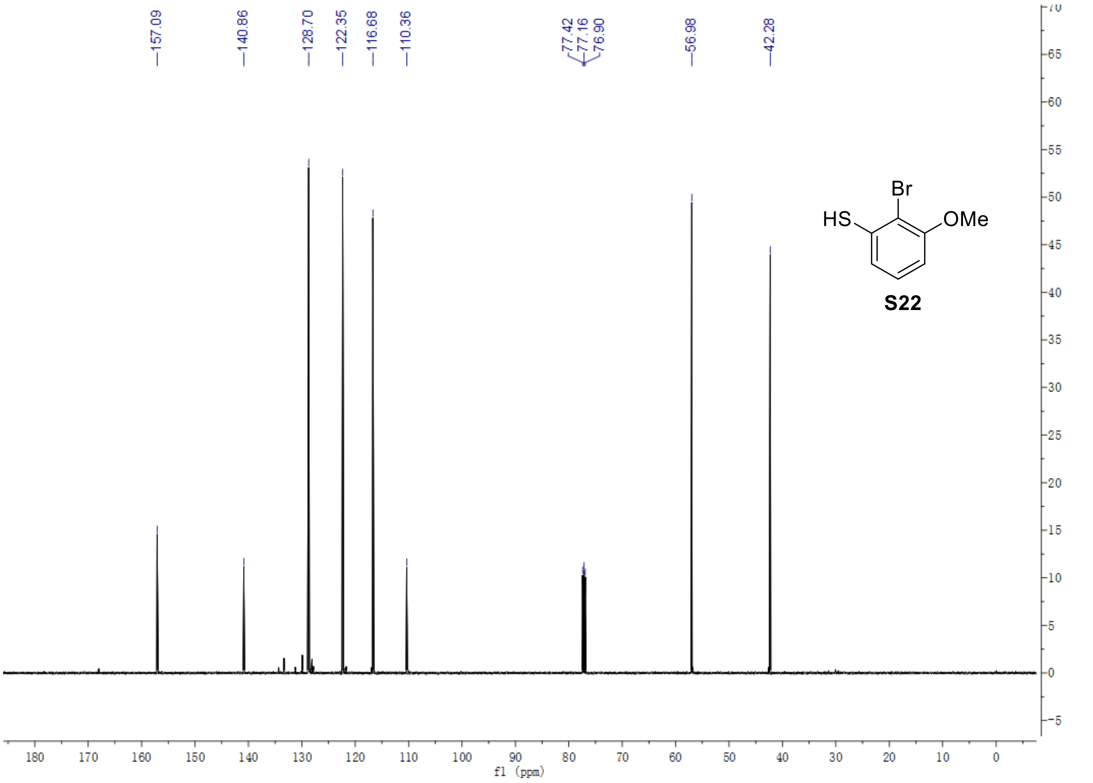

Supplementary Figure 60 ^{1}H NMR spectrum of **S23** (500 MHz, $CDCl_3$)

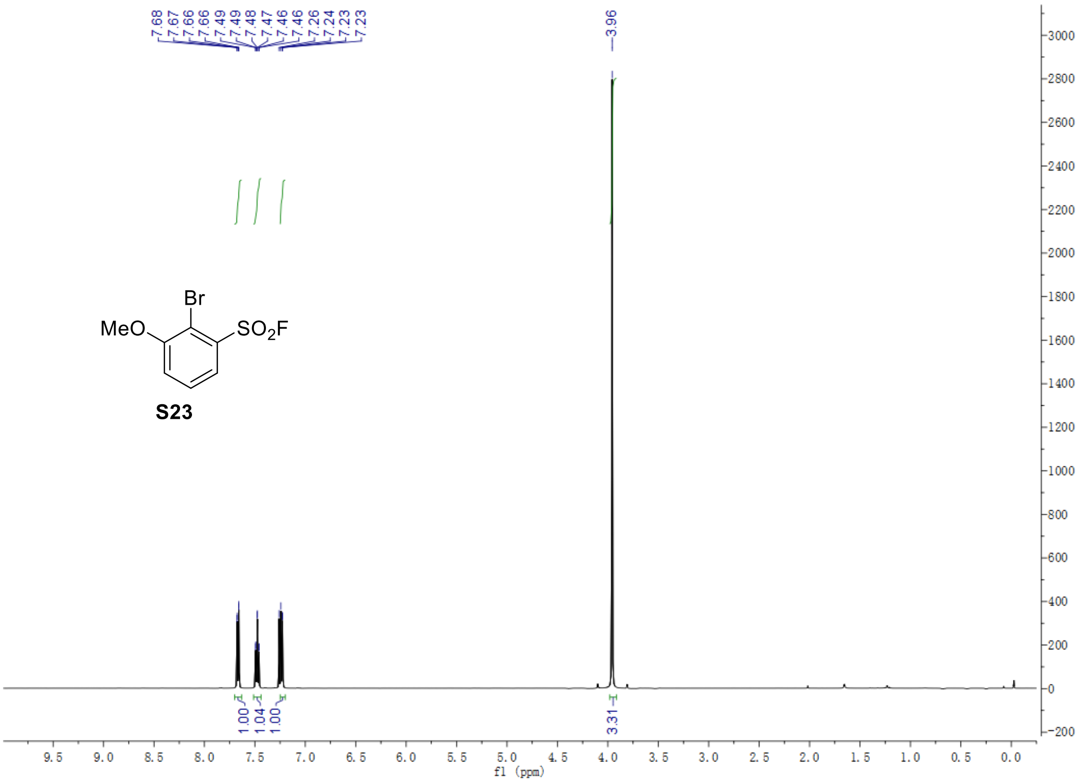

Supplementary Figure 61 ^{13}C NMR spectrum of **S23** (126 MHz, $CDCl_3$)

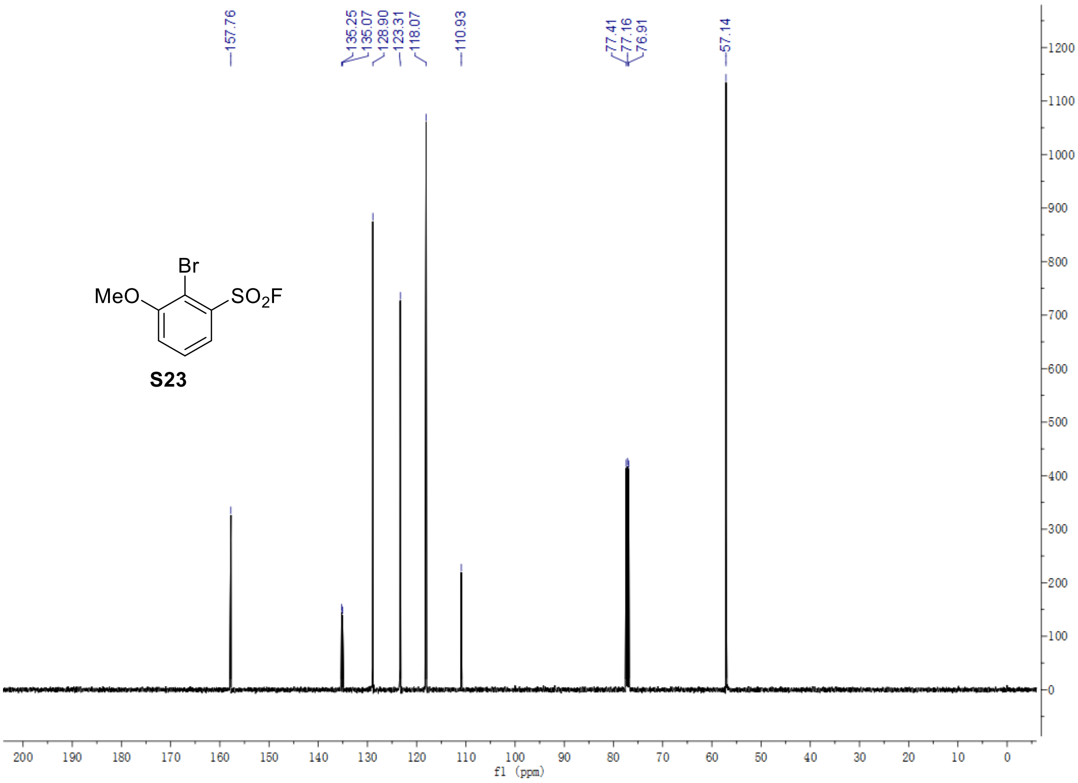

Supplementary Figure 62 ^{19}F NMR spectrum of **S23** (376 MHz, $CDCl_3$)

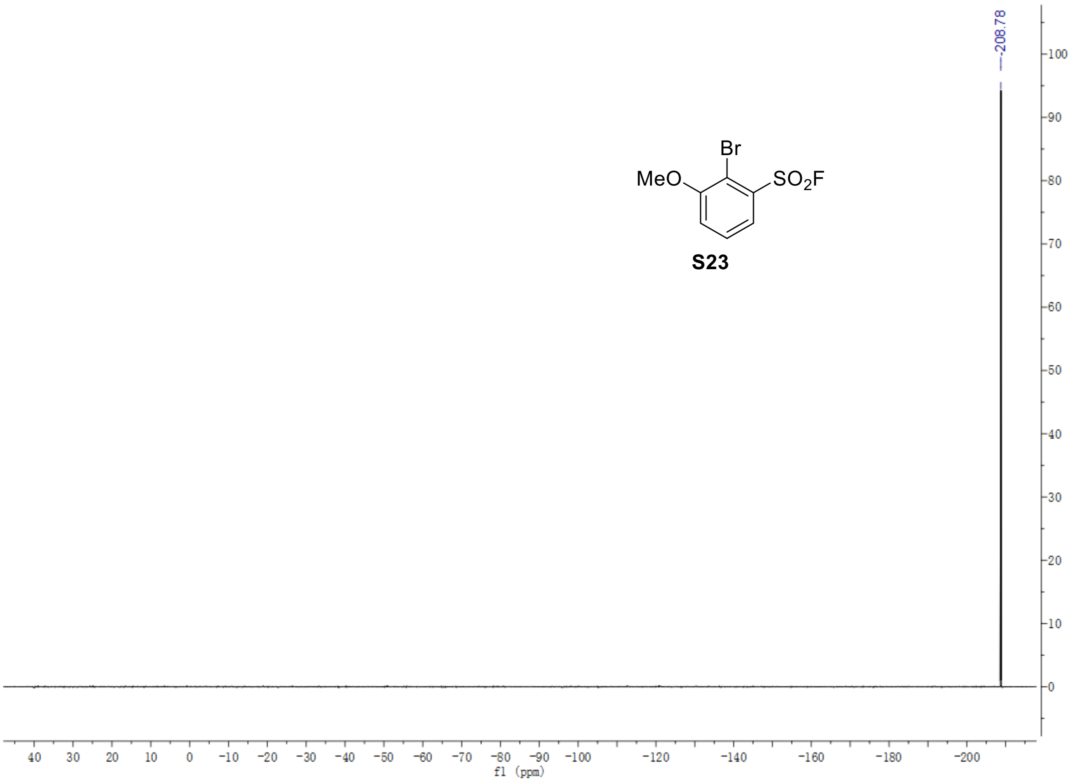

Supplementary Figure 63 ^{1}H NMR spectrum of **S24** (500 MHz, $CDCl_3$)

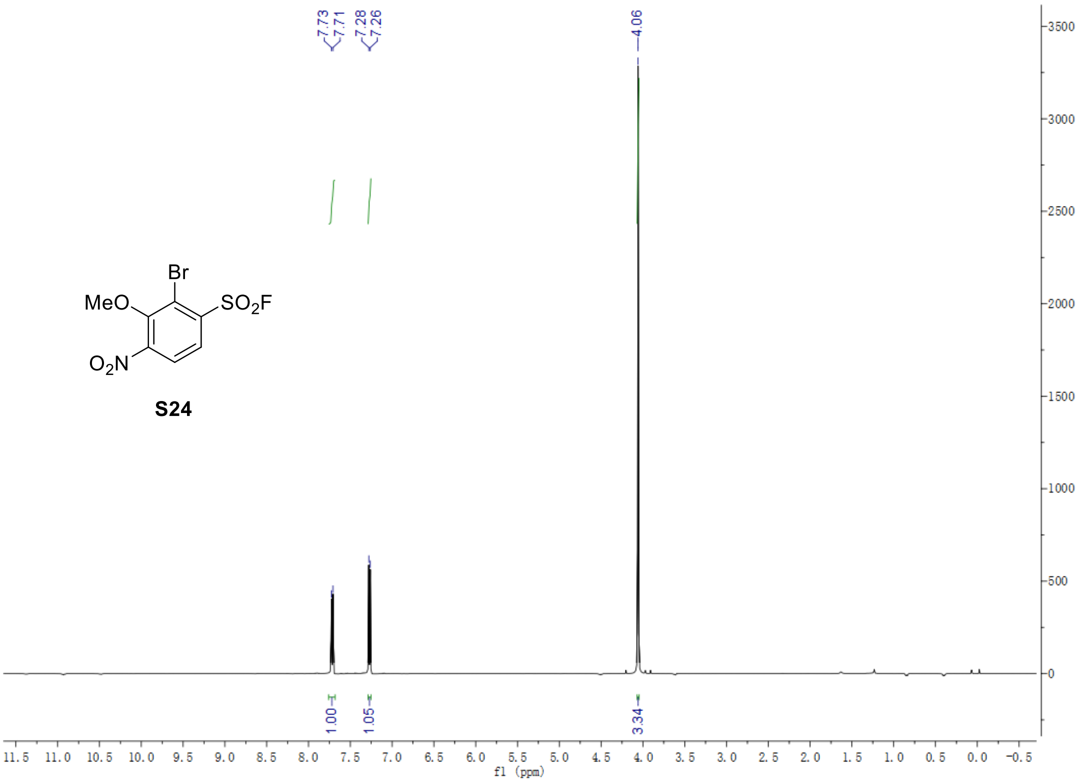

Supplementary Figure 64 ^{13}C NMR spectrum of **S24** (126 MHz, $CDCl_3$)

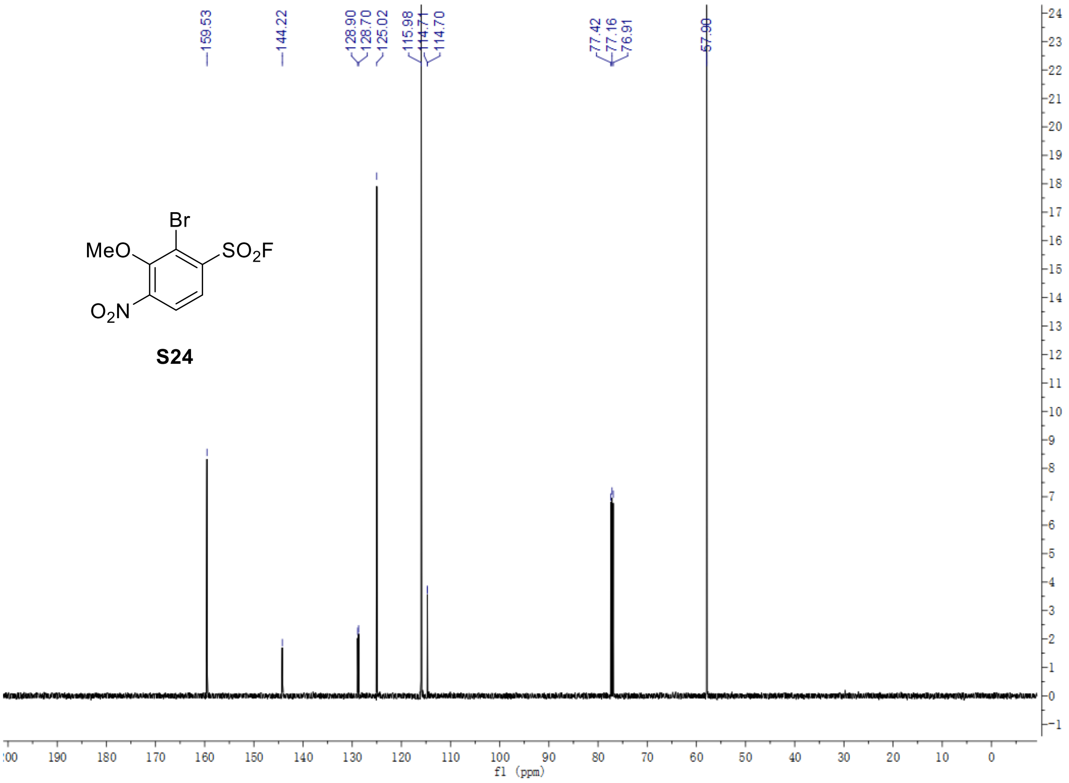

Supplementary Figure 65 ^{19}F NMR spectrum of **S24** (376 MHz, $CDCl_3$)

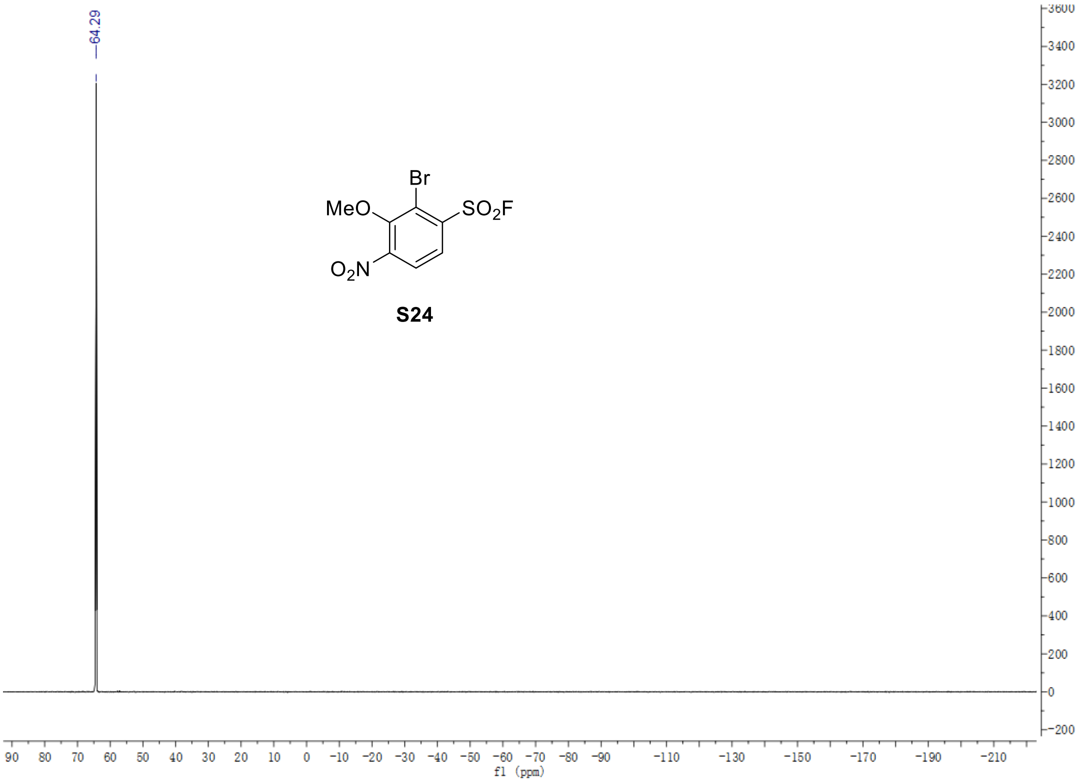

Supplementary Figure 66 ^{1}H NMR spectrum of **S25** (600 MHz, $CDCl_3$)

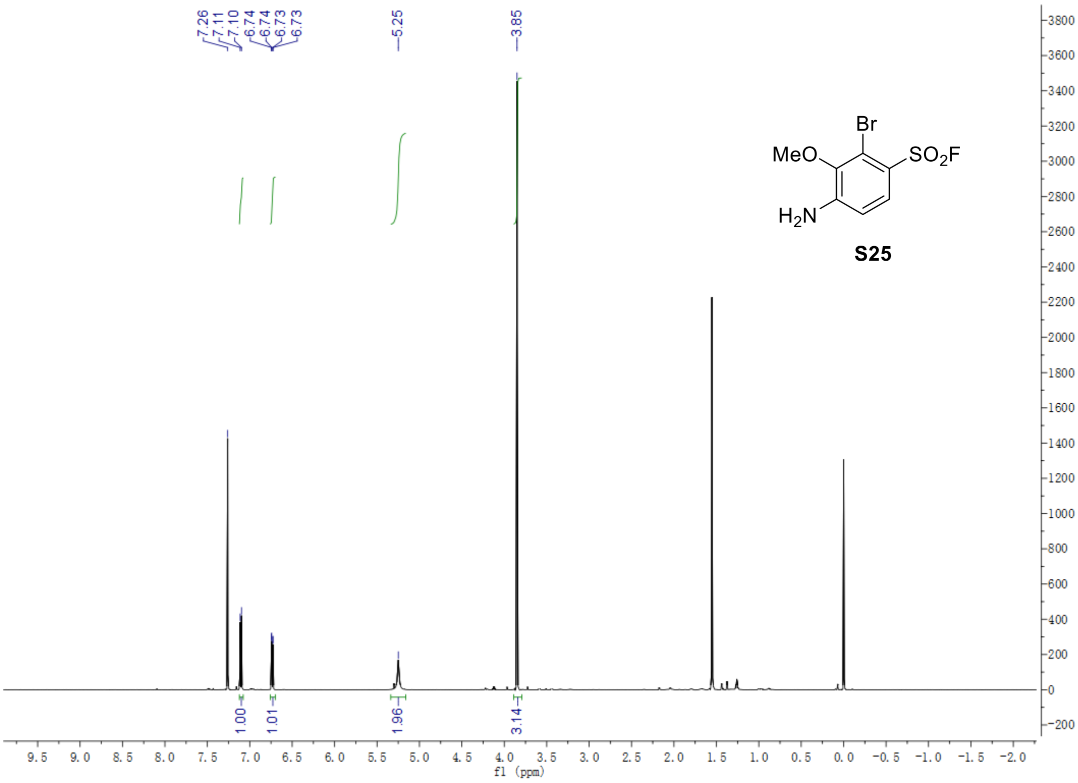

Supplementary Figure 67 ^{13}C NMR spectrum of **S25** (151 MHz, $CDCl_3$)

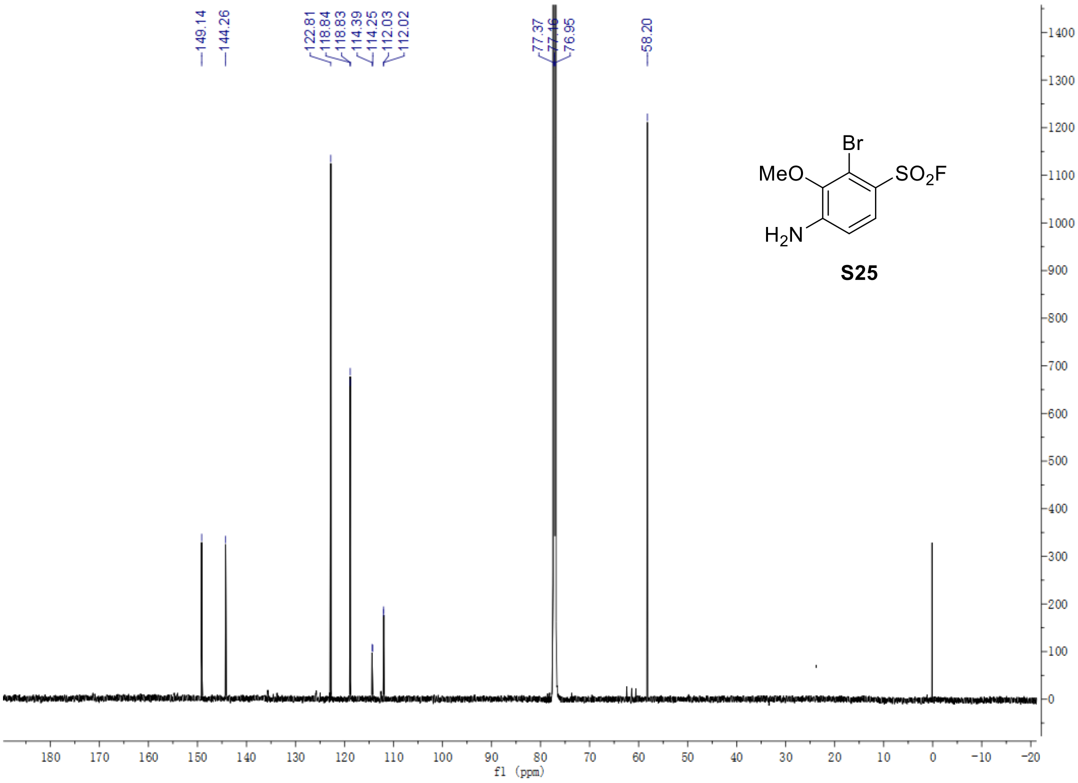

Supplementary Figure 68 ^{19}F NMR spectrum of **S25** (376 MHz, $CDCl_3$)

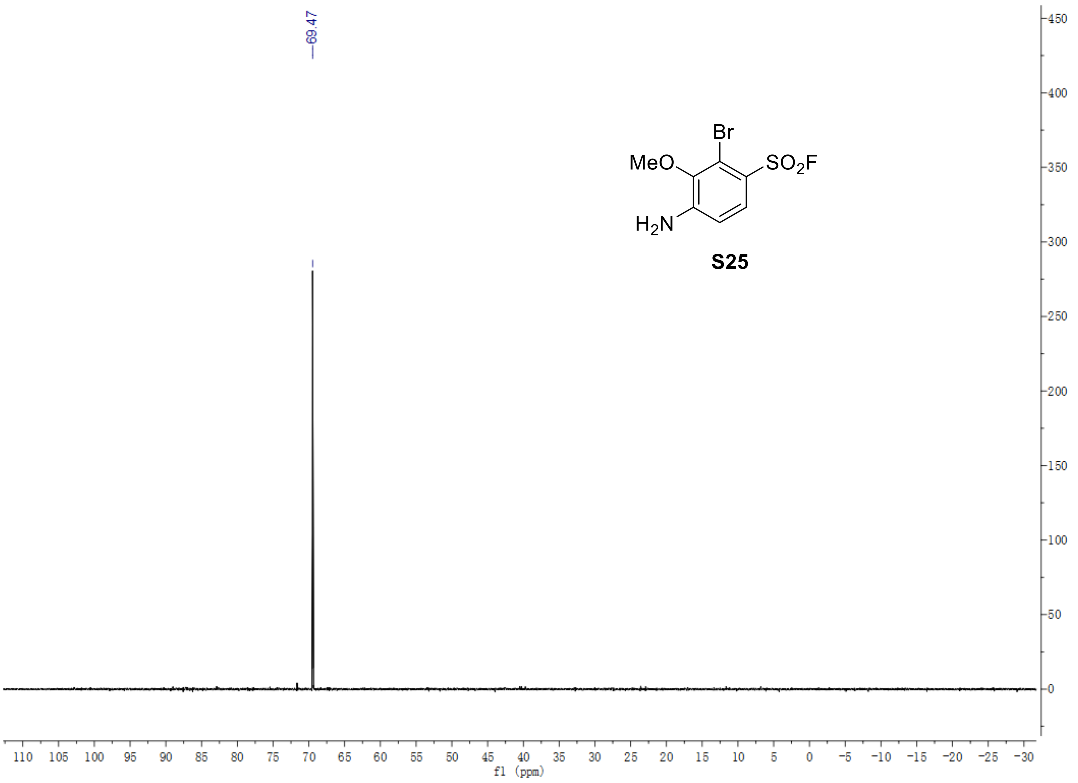

Supplementary Figure 69 ^{1}H NMR spectrum of **S26** (500 MHz, $CDCl_3$)

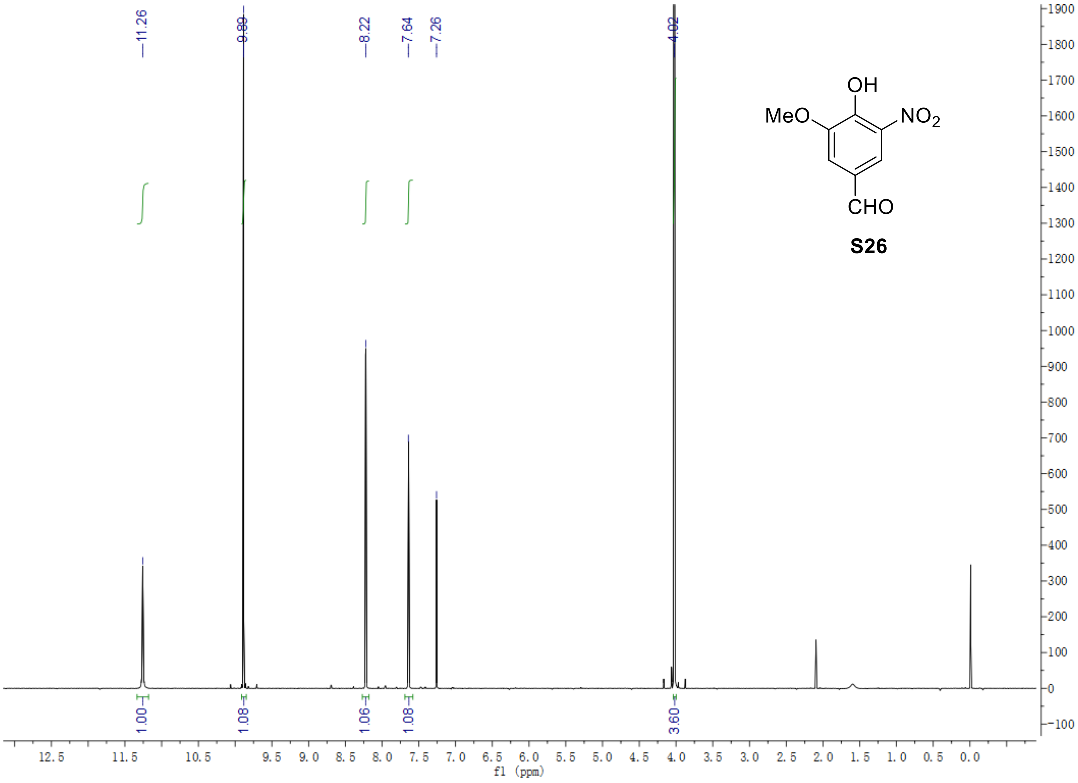

Supplementary Figure 70 ^{13}C NMR spectrum of **S26** (151 MHz, $CDCl_3$)

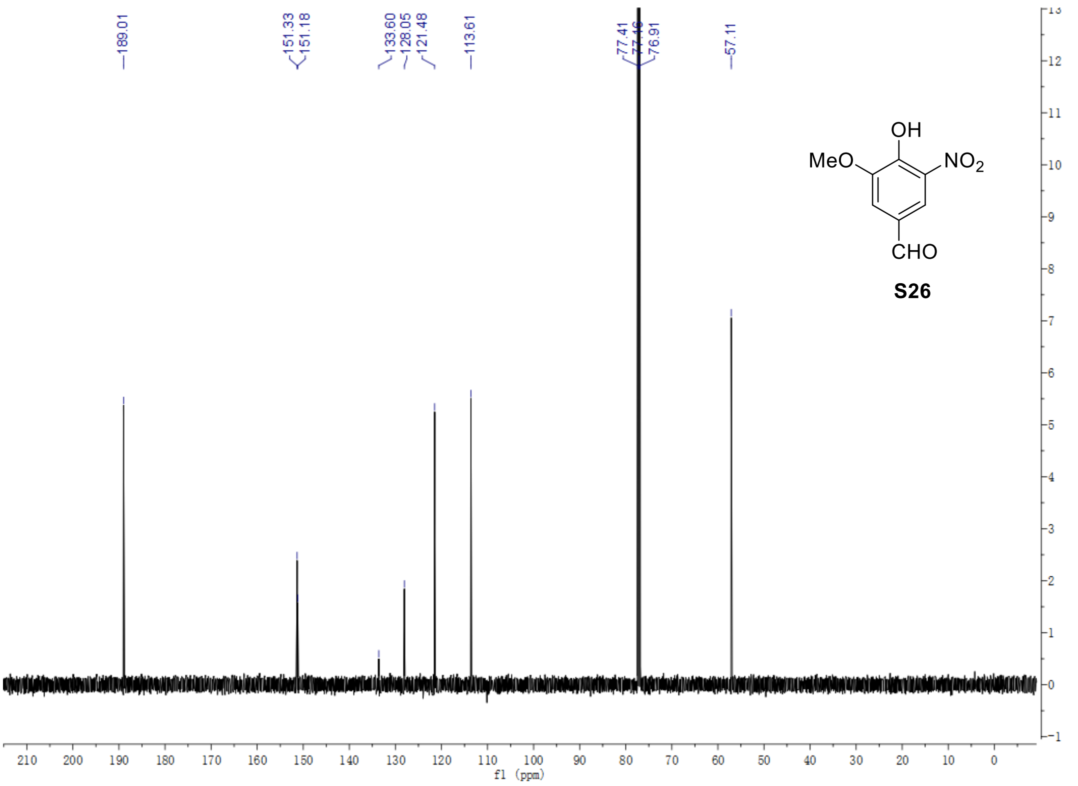

Supplementary Figure 71 ^{1}H NMR spectrum of **S27** (500 MHz, $CDCl_3$)

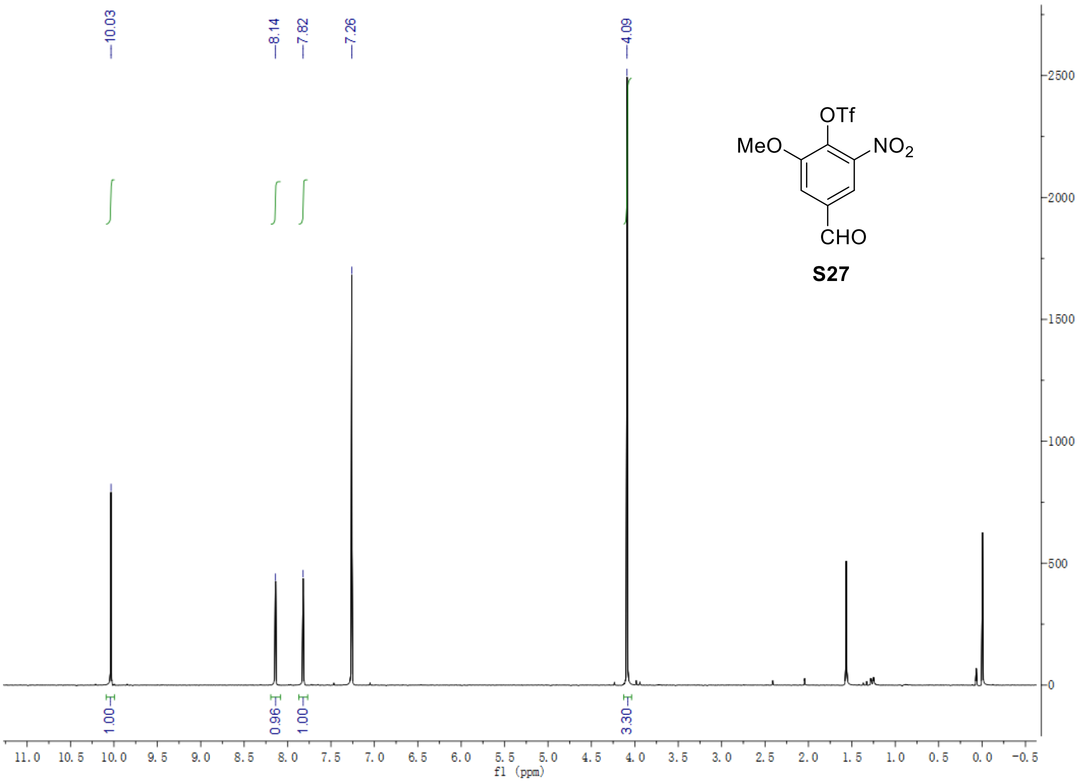

Supplementary Figure 72 ^{13}C NMR spectrum of **S27** (126 MHz, $CDCl_3$)

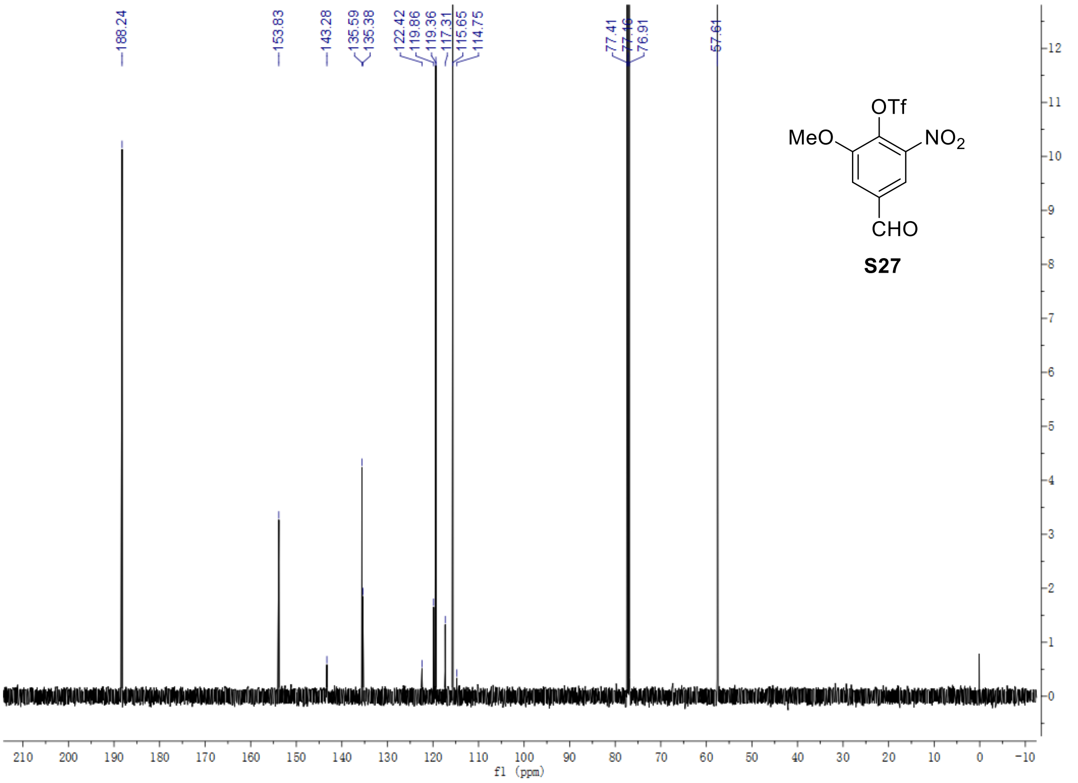

Supplementary Figure 73 ^{19}F NMR spectrum of **S27** (376 MHz, $CDCl_3$)

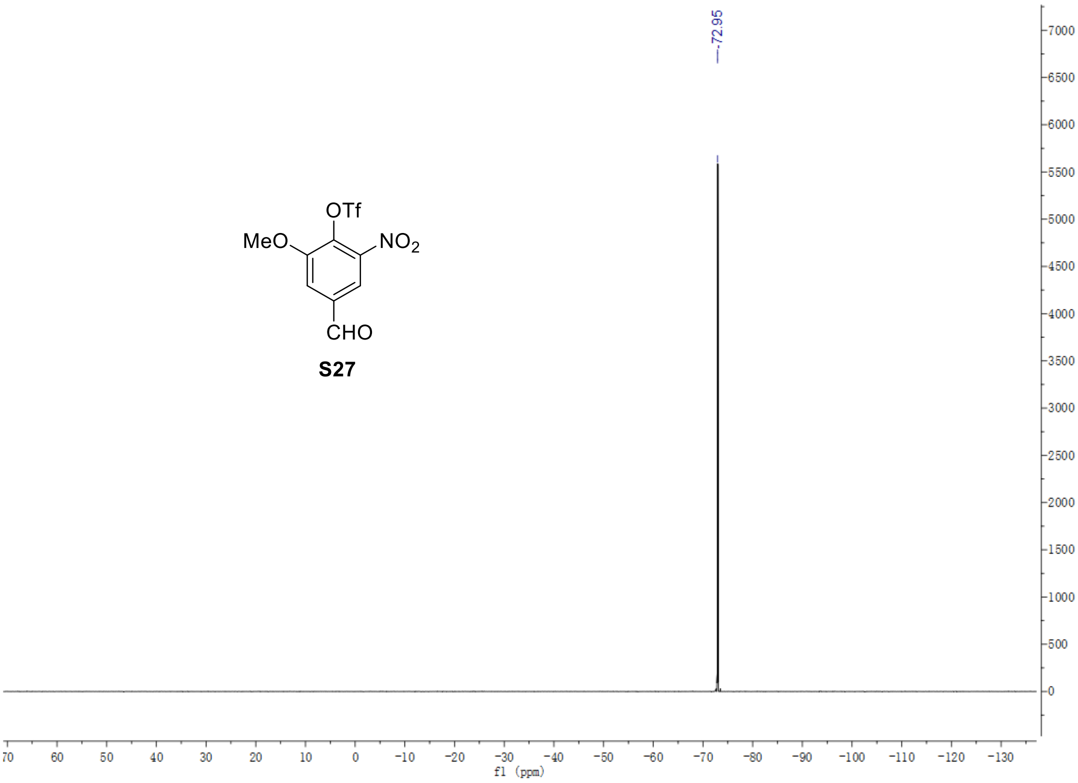

Supplementary Figure 74 ^{1}H NMR spectrum of **S29** (500 MHz, $CDCl_3$)

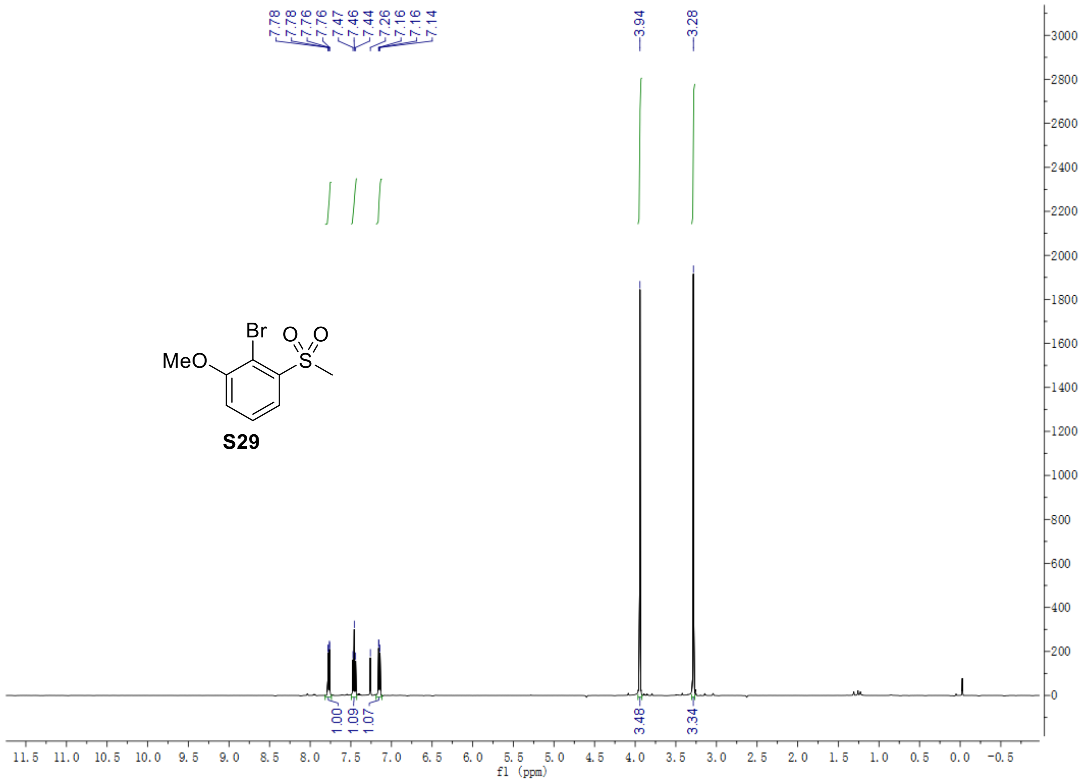

Supplementary Figure 75 ^{13}C NMR spectrum of **S29** (126 MHz, $CDCl_3$)

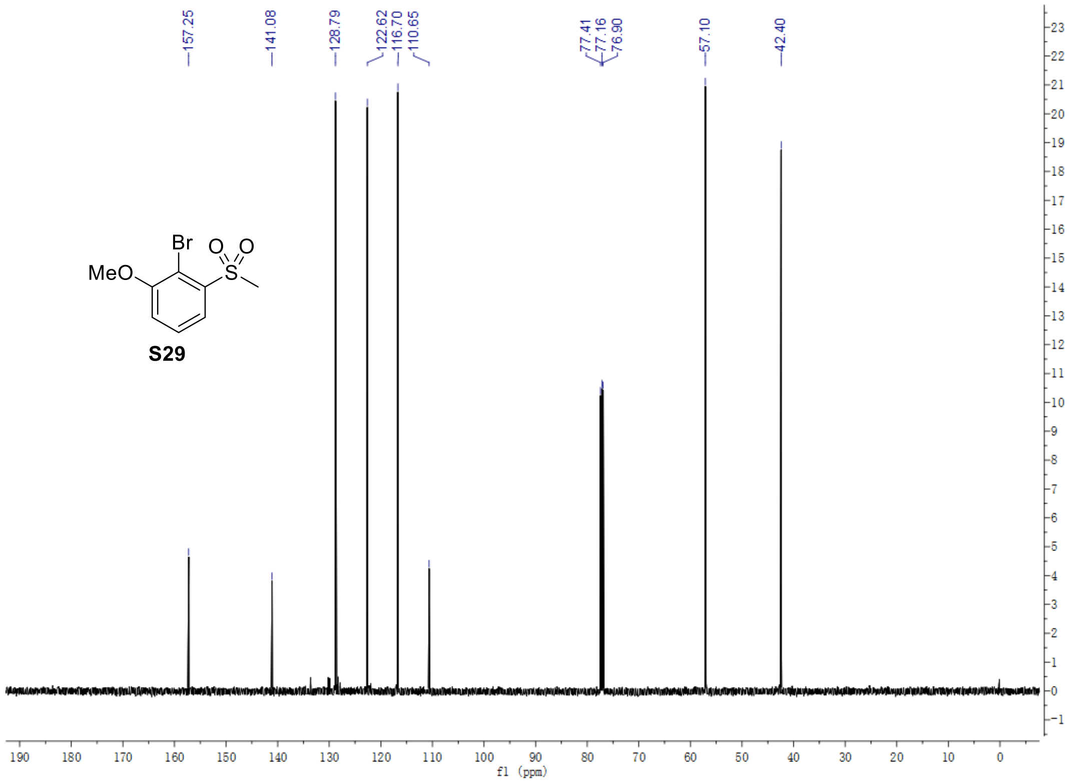

Supplementary Figure 76 ^{1}H NMR spectrum of **S30** (500 MHz, $CDCl_3$)

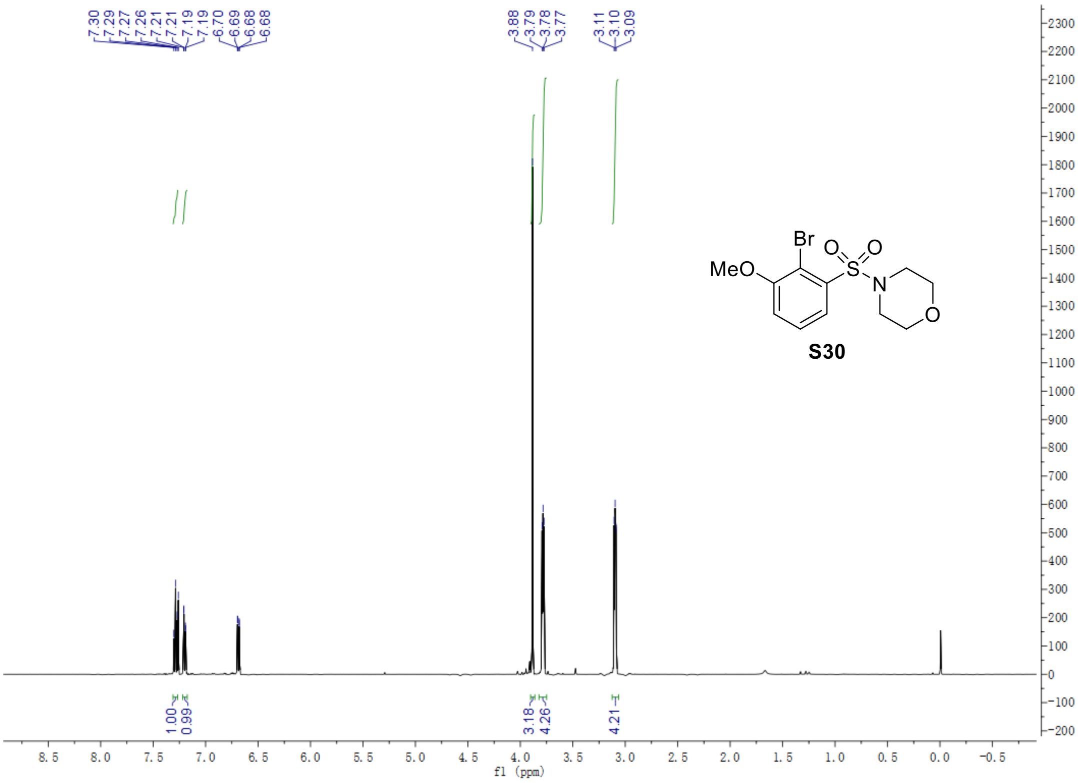

Supplementary Figure 77 ^{13}C NMR spectrum of **S30** (126 MHz, $CDCl_3$)

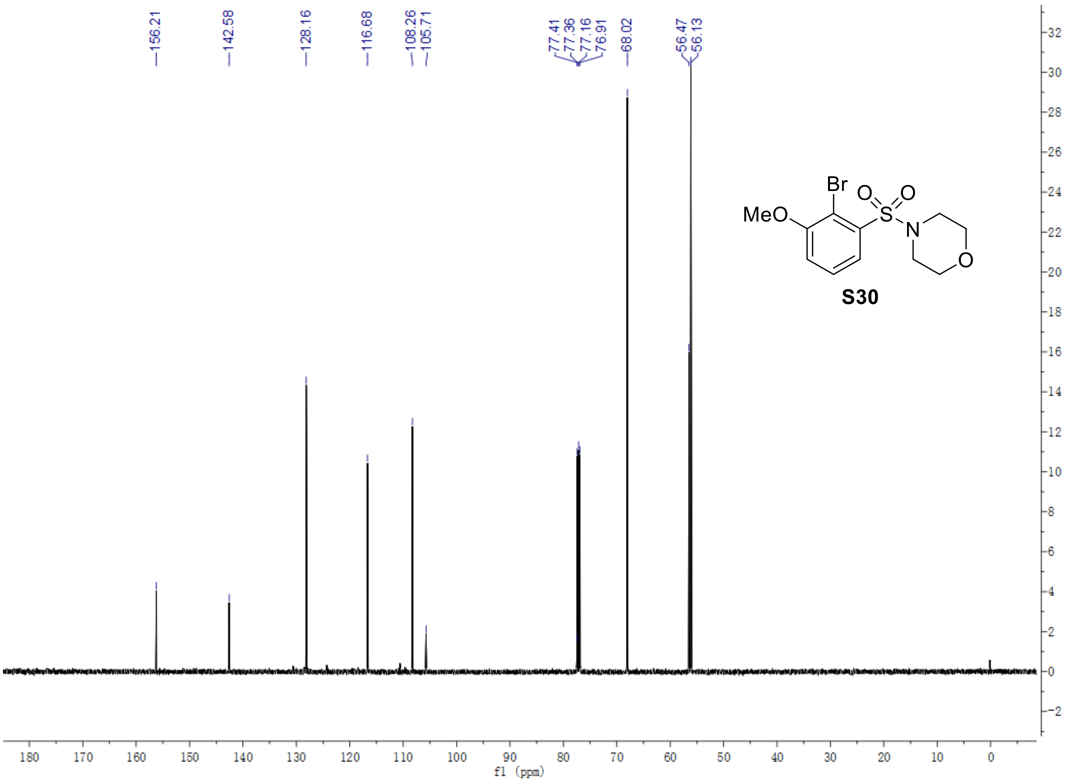

Supplementary Figure 78 ^{1}H NMR spectrum of **3a** (500 MHz, $CDCl_3$)

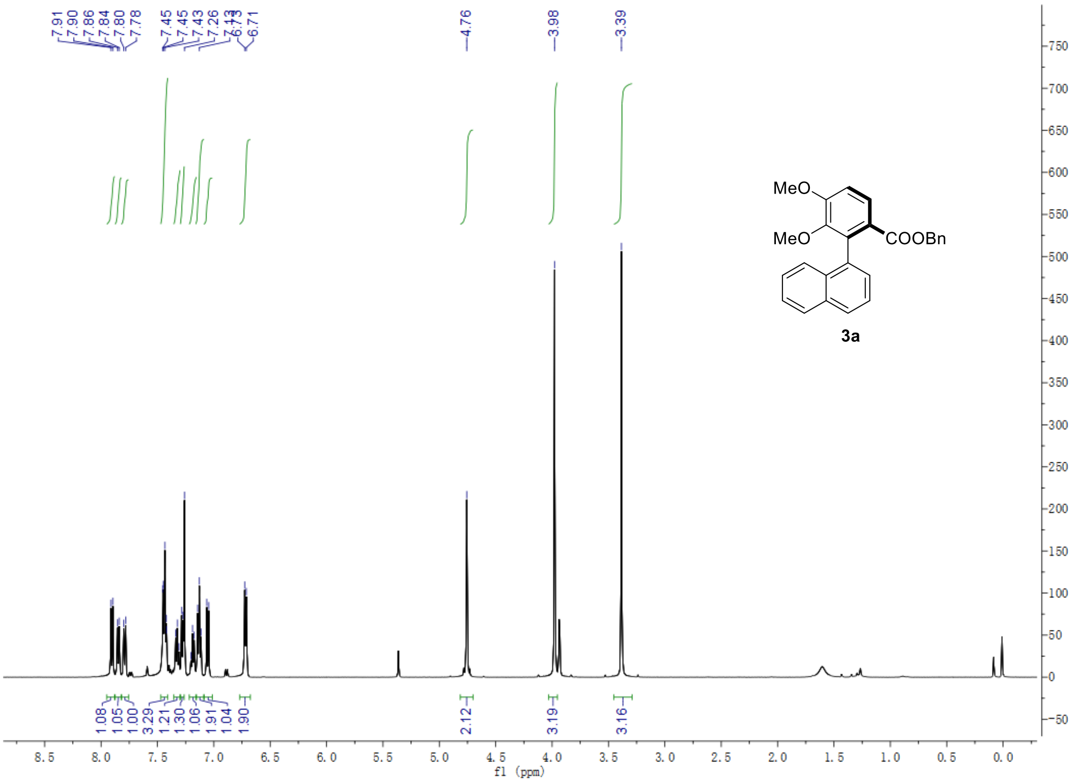

Supplementary Figure 79 ^{13}C NMR spectrum of **3a** (126 MHz, $CDCl_3$)

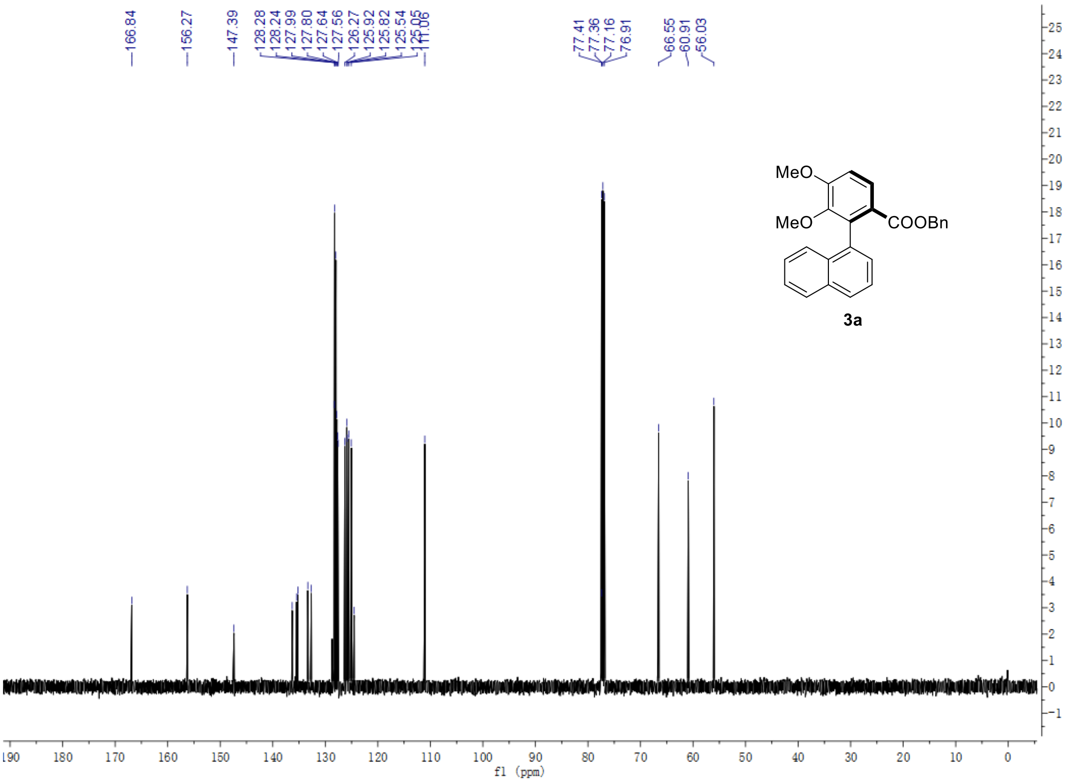

Supplementary Figure 80 ¹H NMR spectrum of **3b** (500 MHz, $CDCl_3$)

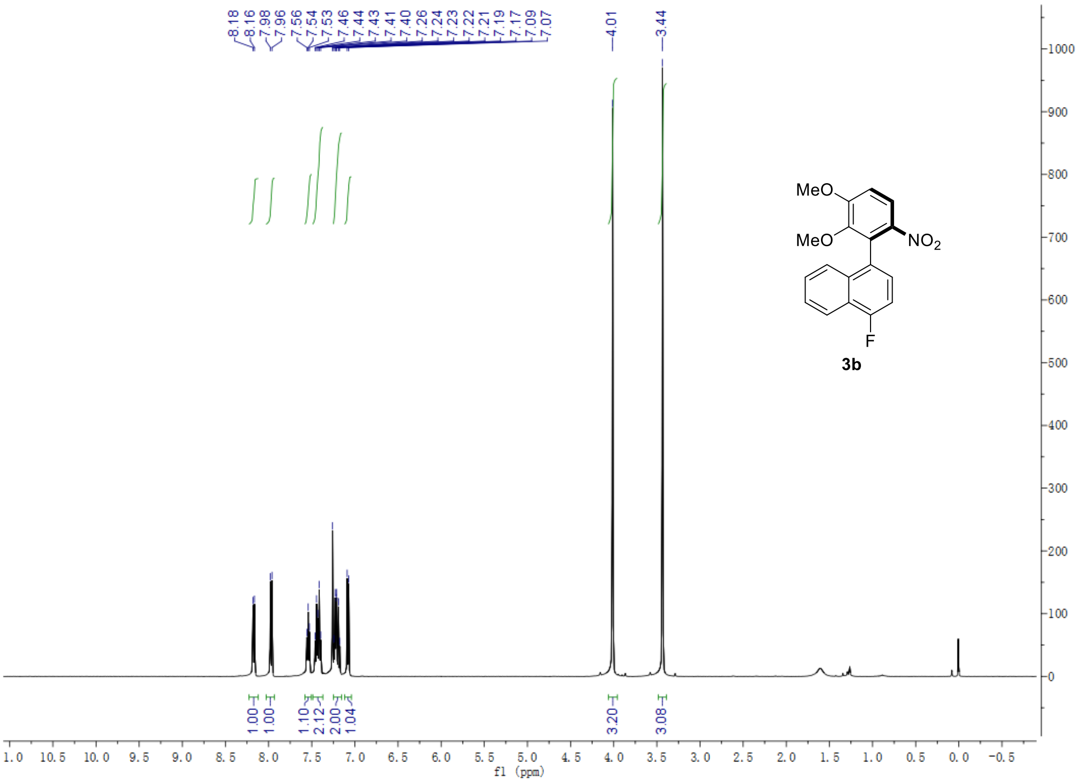

Supplementary Figure 81 ¹³C NMR spectrum of **3b** (126 MHz, $CDCl_3$)

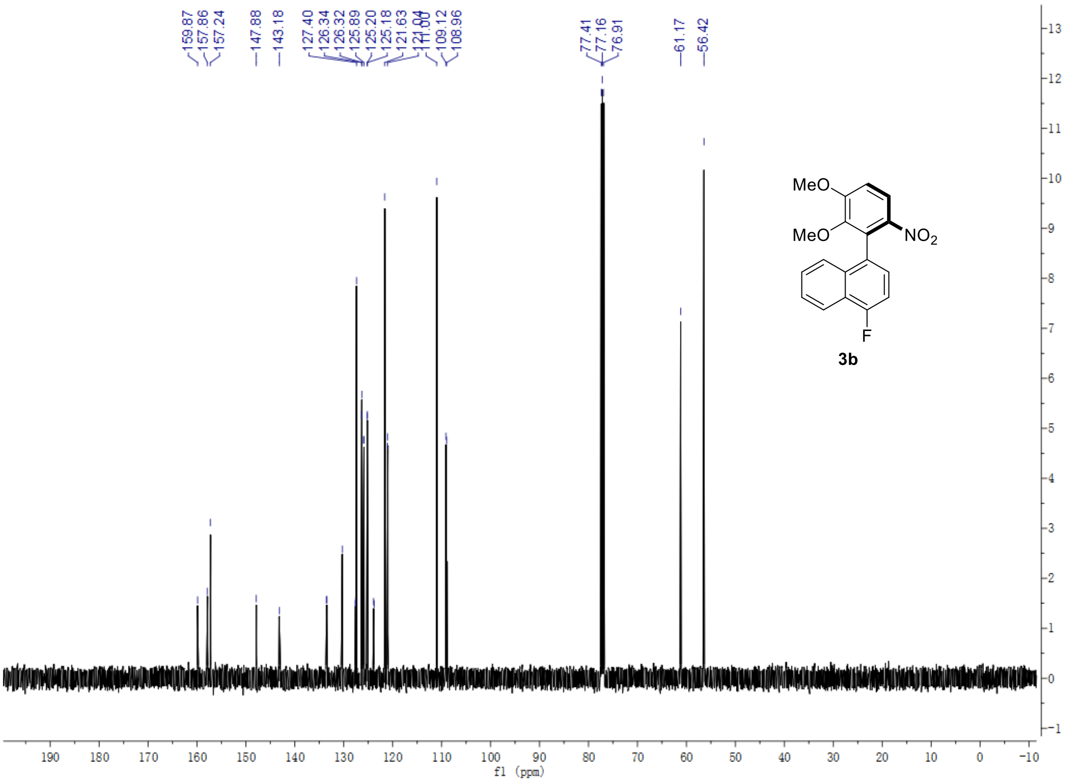

Supplementary Figure 82 ^{19}F NMR spectrum of **3b** (282 MHz, $CDCl_3$)

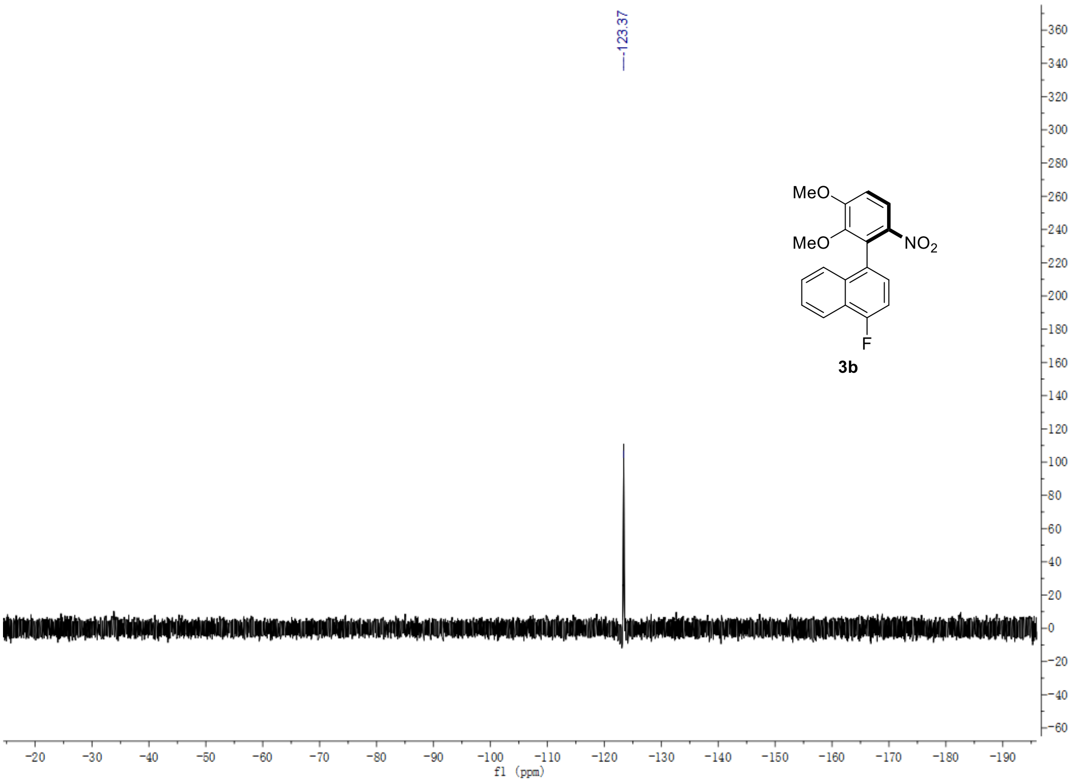

Supplementary Figure 83 ^{1}H NMR spectrum of **3c** (400 MHz, $CDCl_3$)

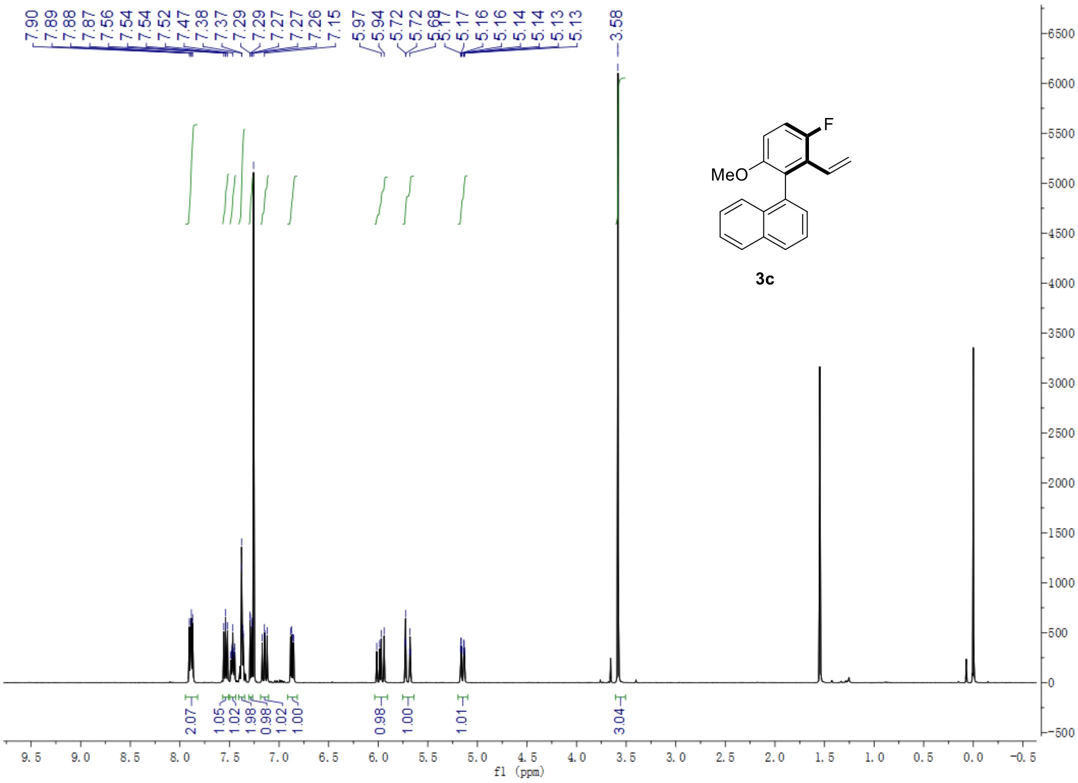

Supplementary Figure 84 ^{13}C NMR spectrum of **3c** (151 MHz, $CDCl_3$)

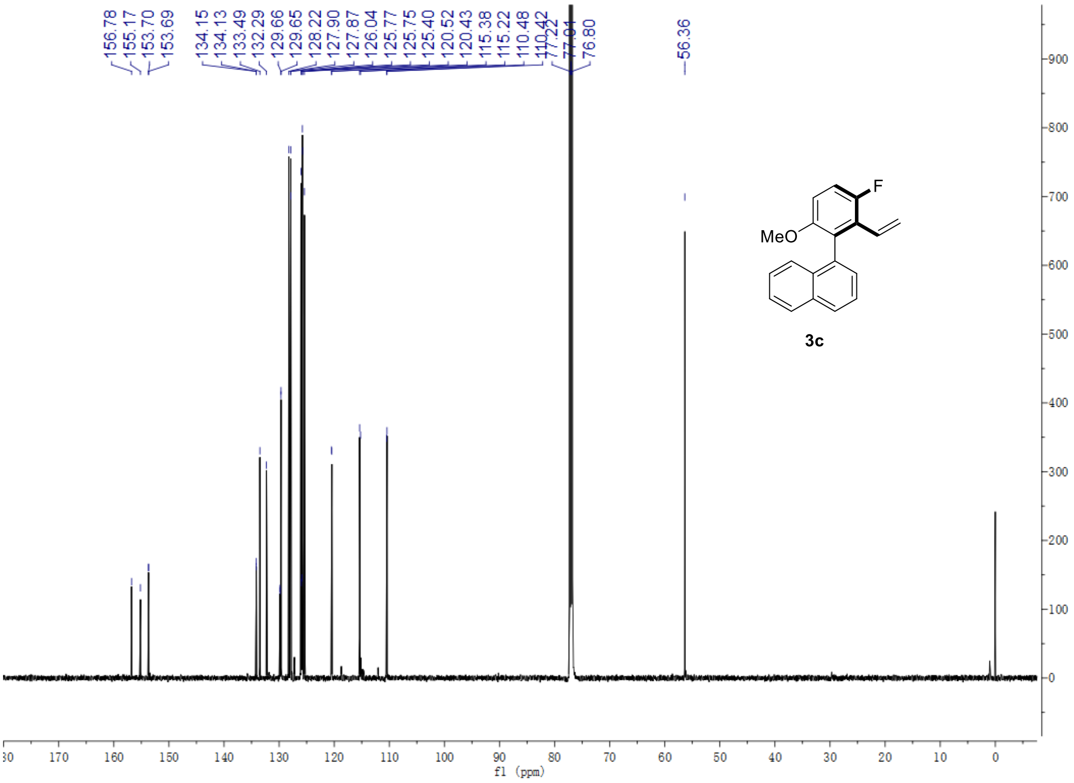

Supplementary Figure 85 ^{19}F NMR spectrum of **3c** (376 MHz, $CDCl_3$)

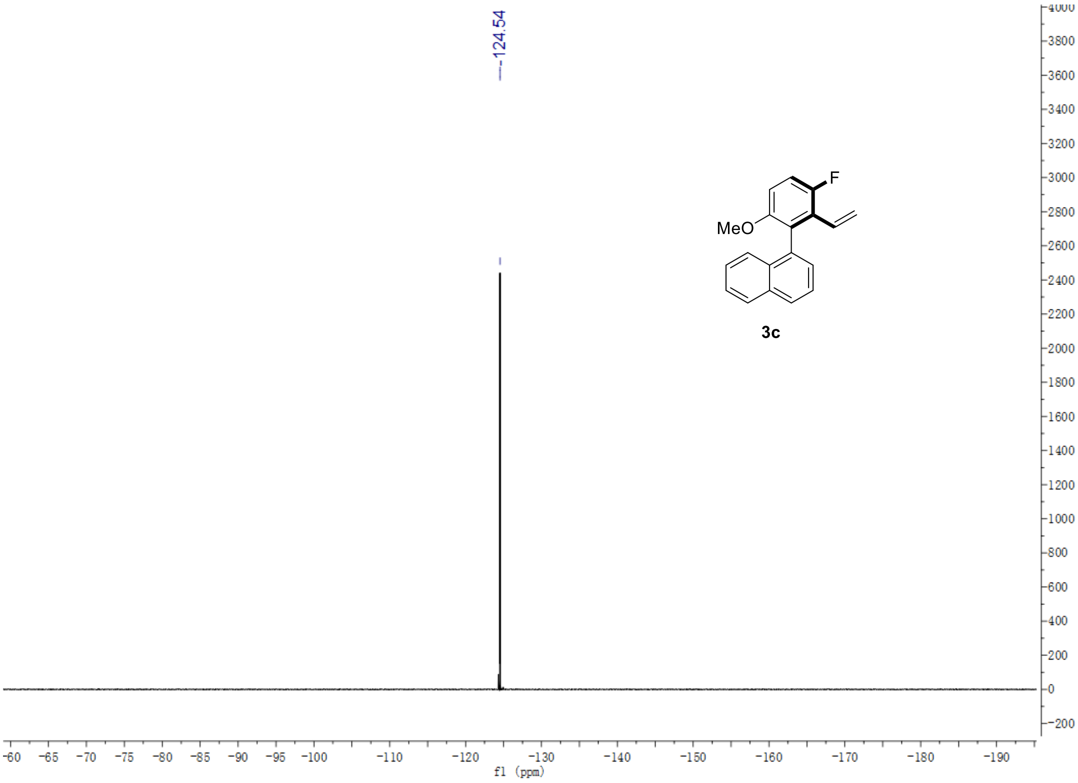

Supplementary Figure 86 ^{1}H NMR spectrum of **3d** (600 MHz, $CDCl_3$)

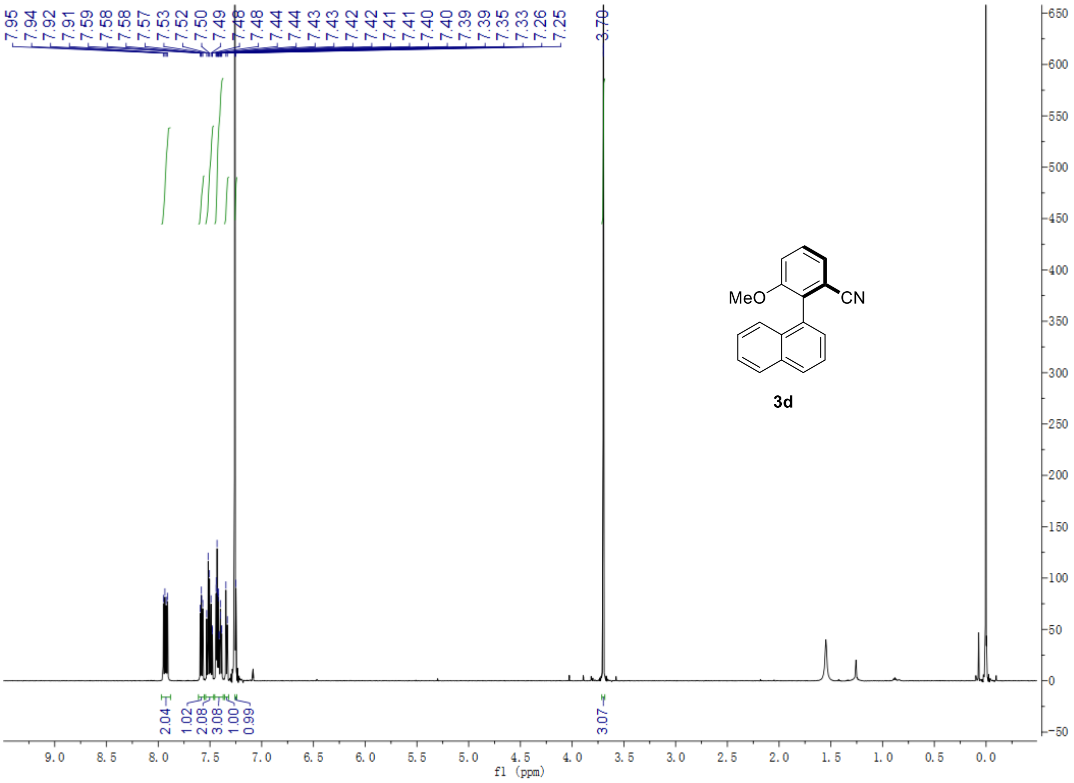

Supplementary Figure 87 ^{13}C NMR spectrum of **3d** (151 MHz, $CDCl_3$)

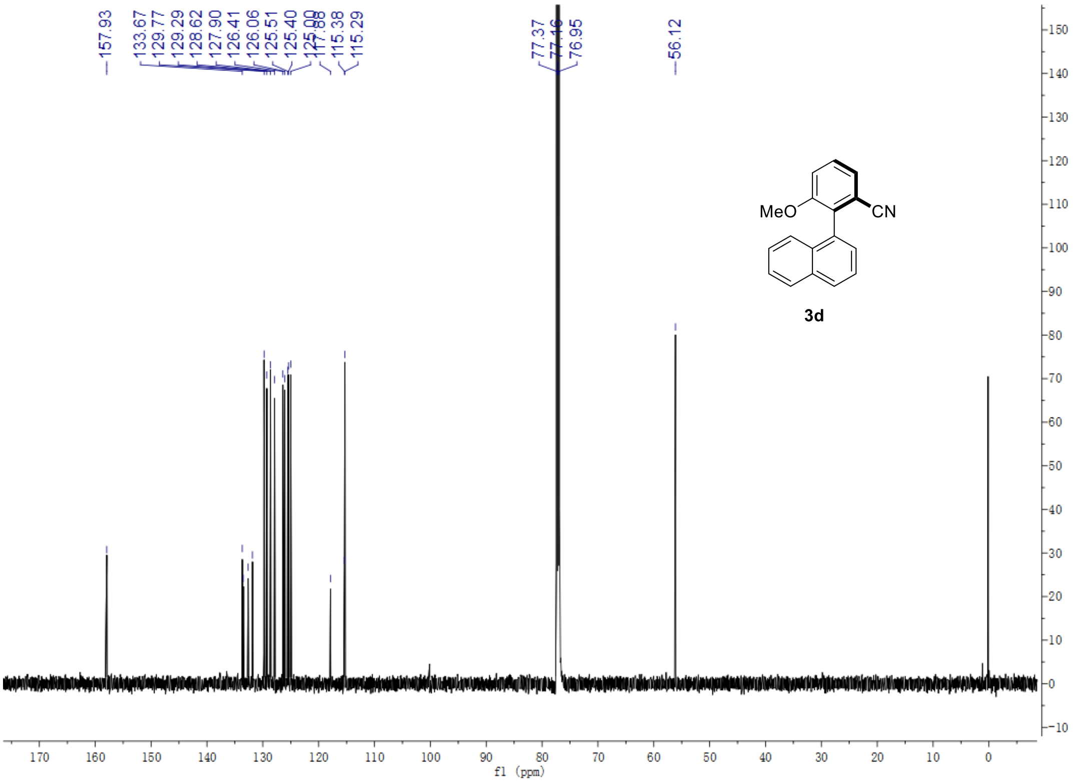

Supplementary Figure 88 ^{1}H NMR spectrum of **3e** (500 MHz, $CDCl_3$)

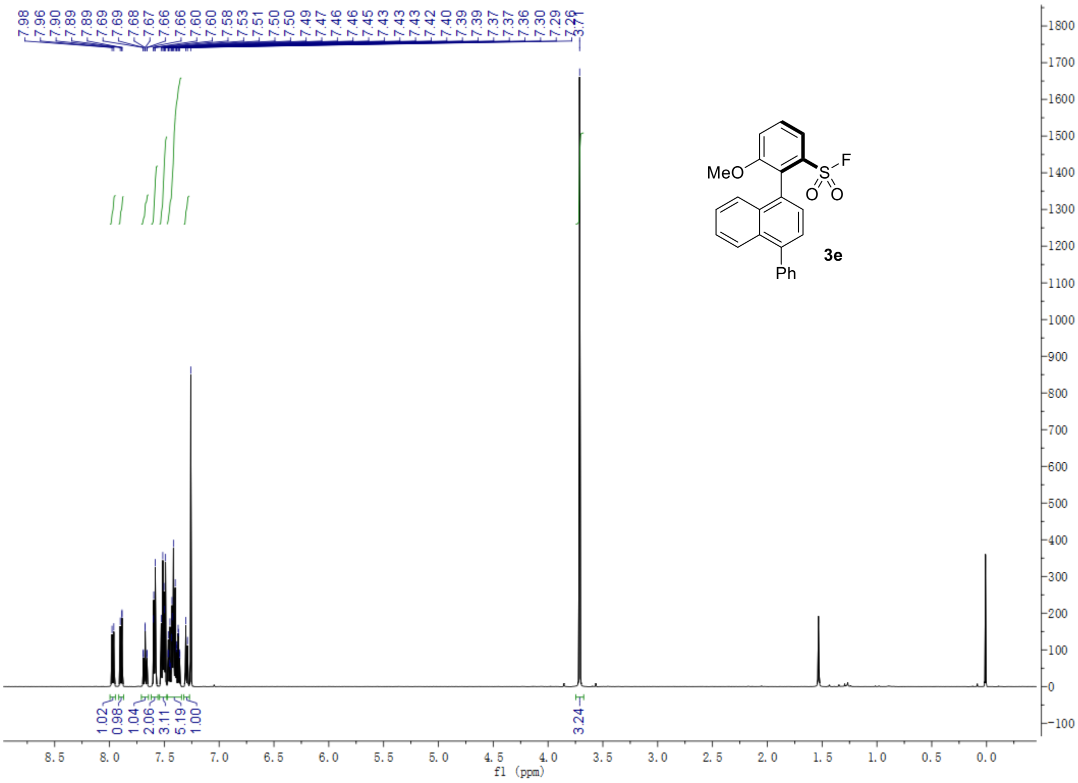

Supplementary Figure 89 ^{13}C NMR spectrum of **3e** (126 MHz, $CDCl_3$)

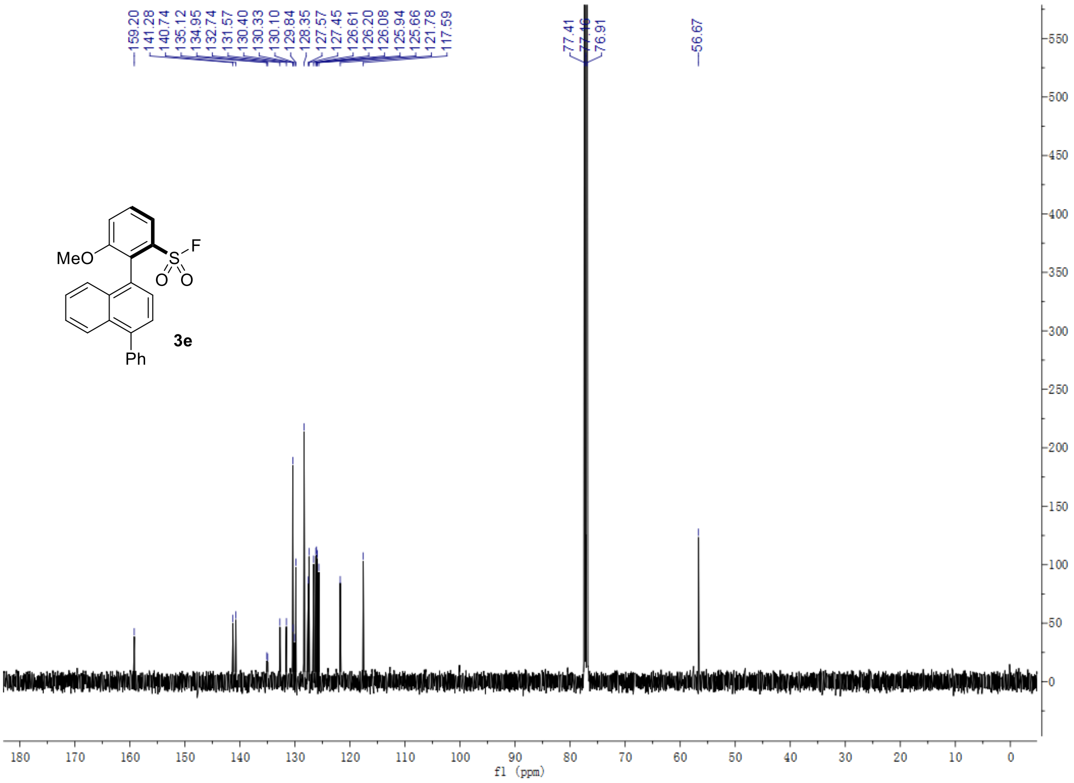

Supplementary Figure 90 ^{19}F NMR spectrum of **3e** (376 MHz, $CDCl_3$)

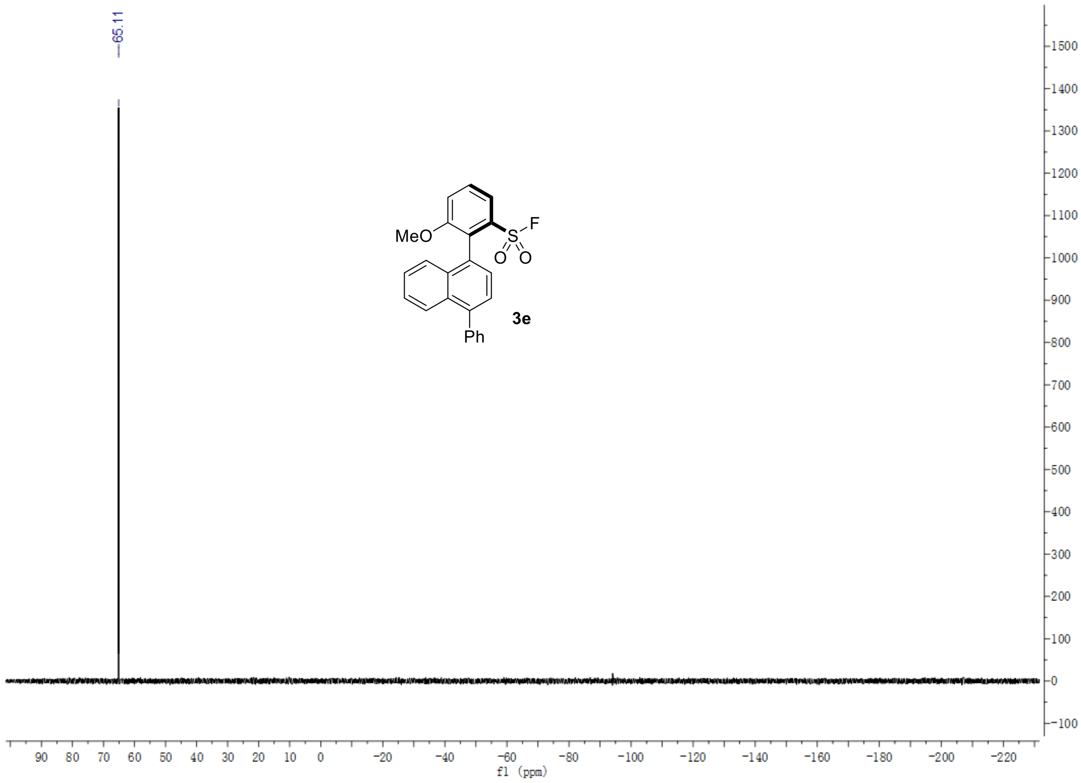

Supplementary Figure 91 ^{1}H NMR spectrum of **3f** (400 MHz, $CDCl_3$)

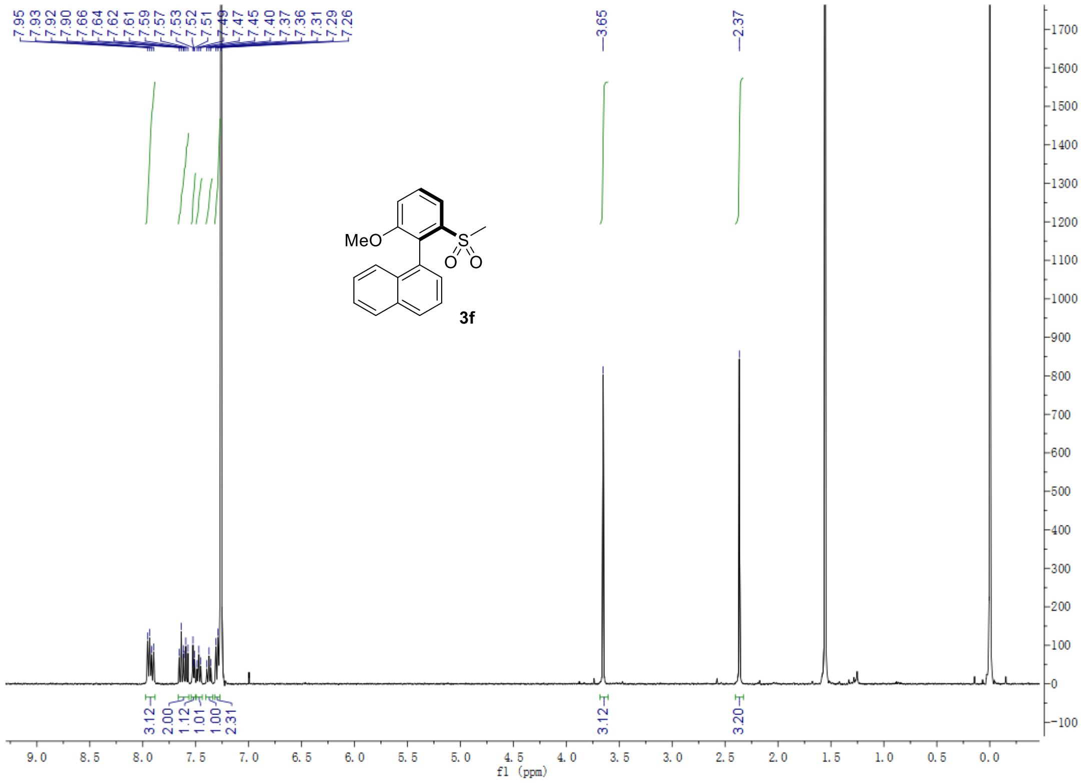

Supplementary Figure 92 ^{13}C NMR spectrum of **3f** (151 MHz, $CDCl_3$)

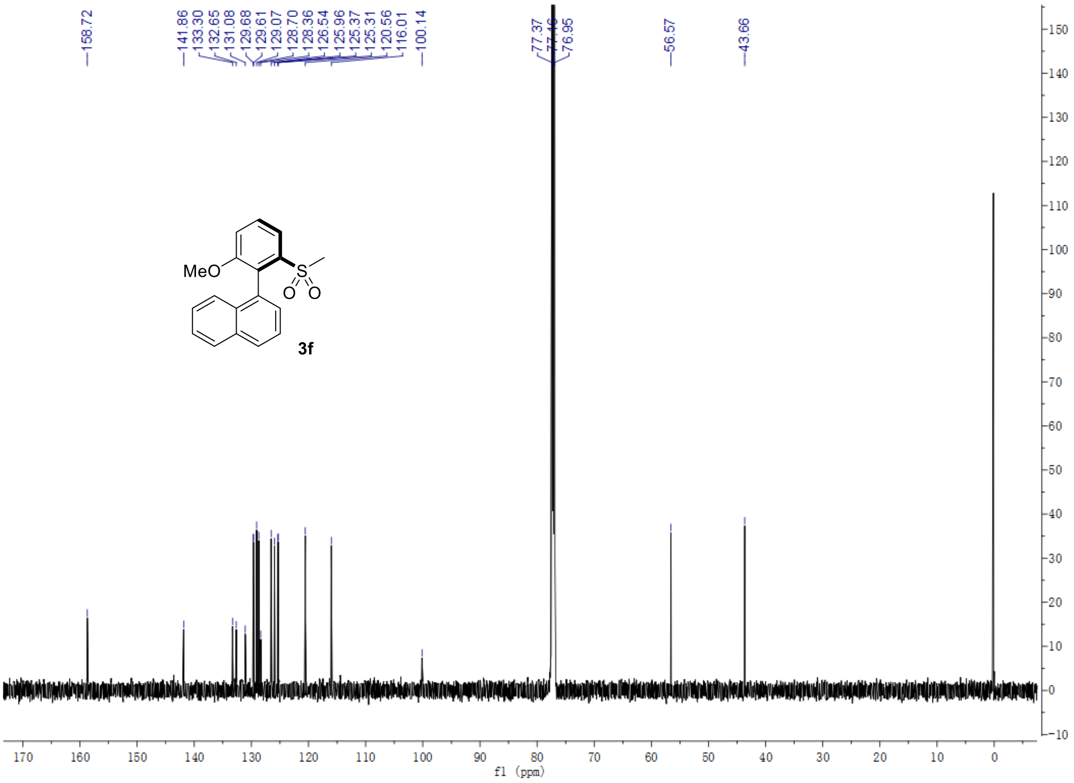

Supplementary Figure 93 ^{1}H NMR spectrum of **3g** (400 MHz, $CDCl_3$)

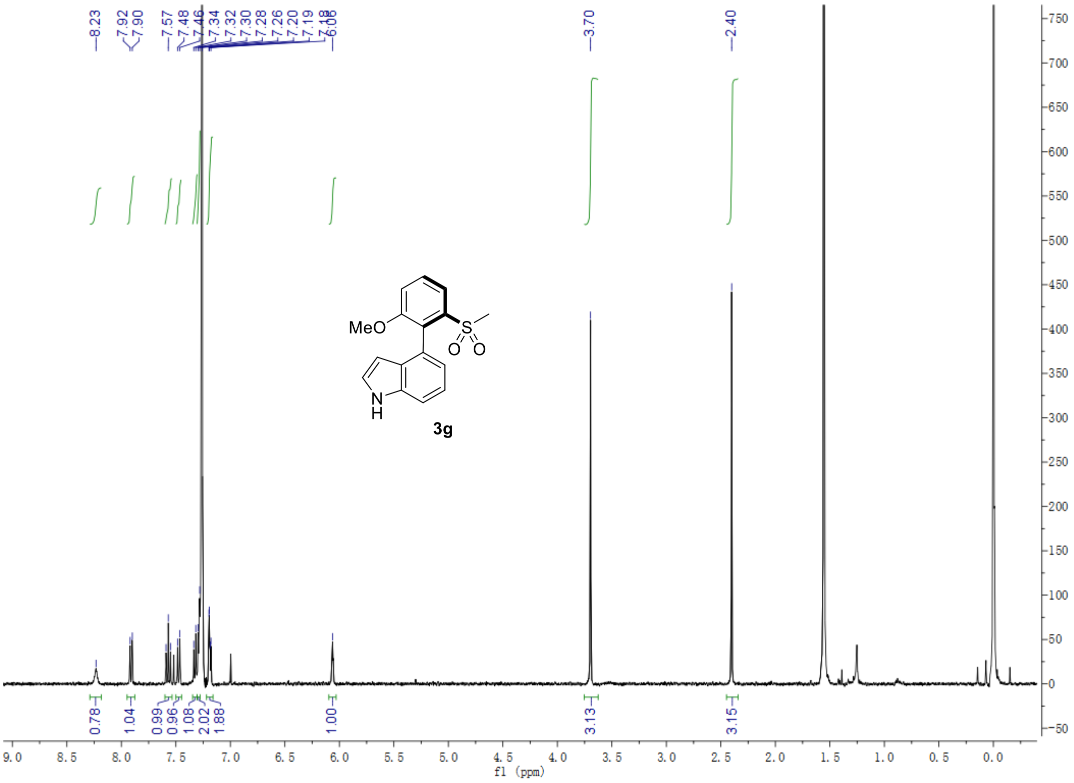

Supplementary Figure 94 ^{13}C NMR spectrum of **3g** (151 MHz, $CDCl_3$)

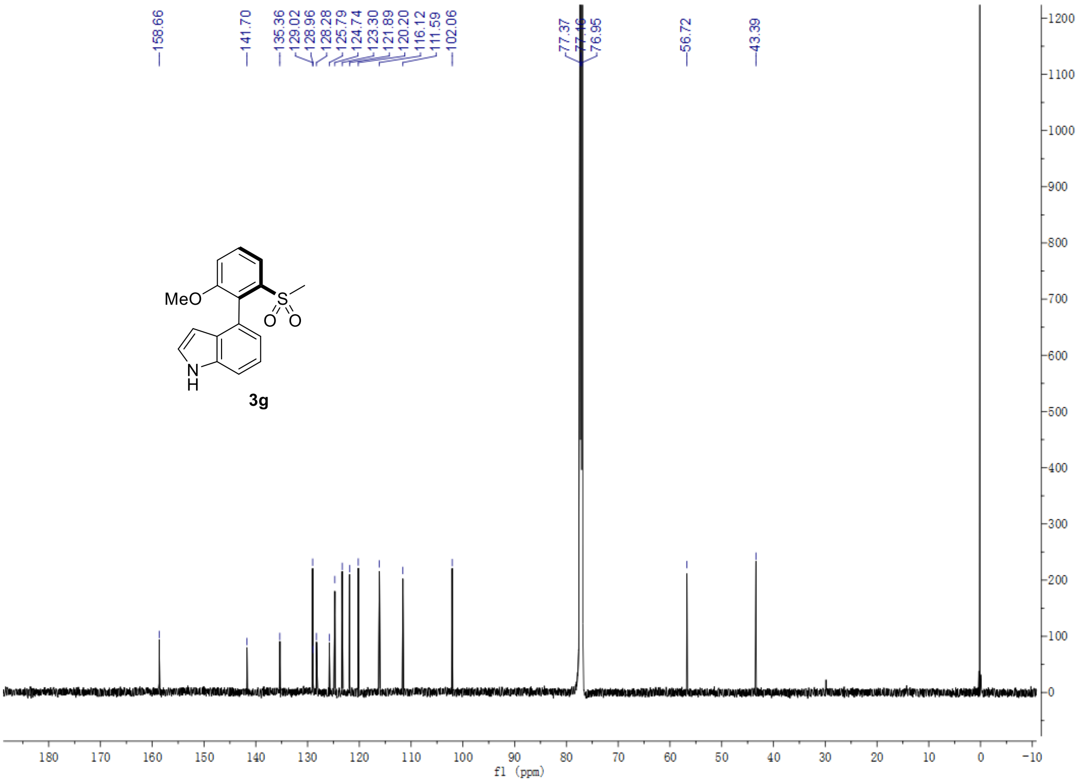

Supplementary Figure 95 ^{1}H NMR spectrum of **3h** (500 MHz, $CDCl_3$)

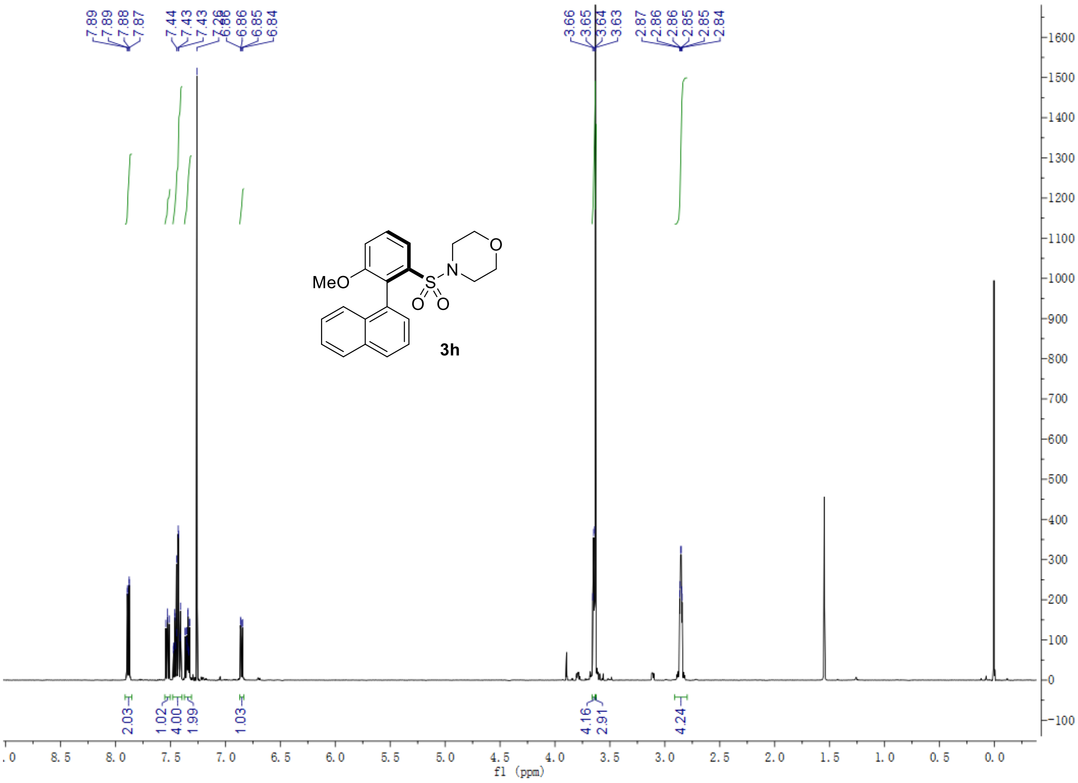

Supplementary Figure 96 ^{13}C NMR spectrum of **3h** (126 MHz, $CDCl_3$)

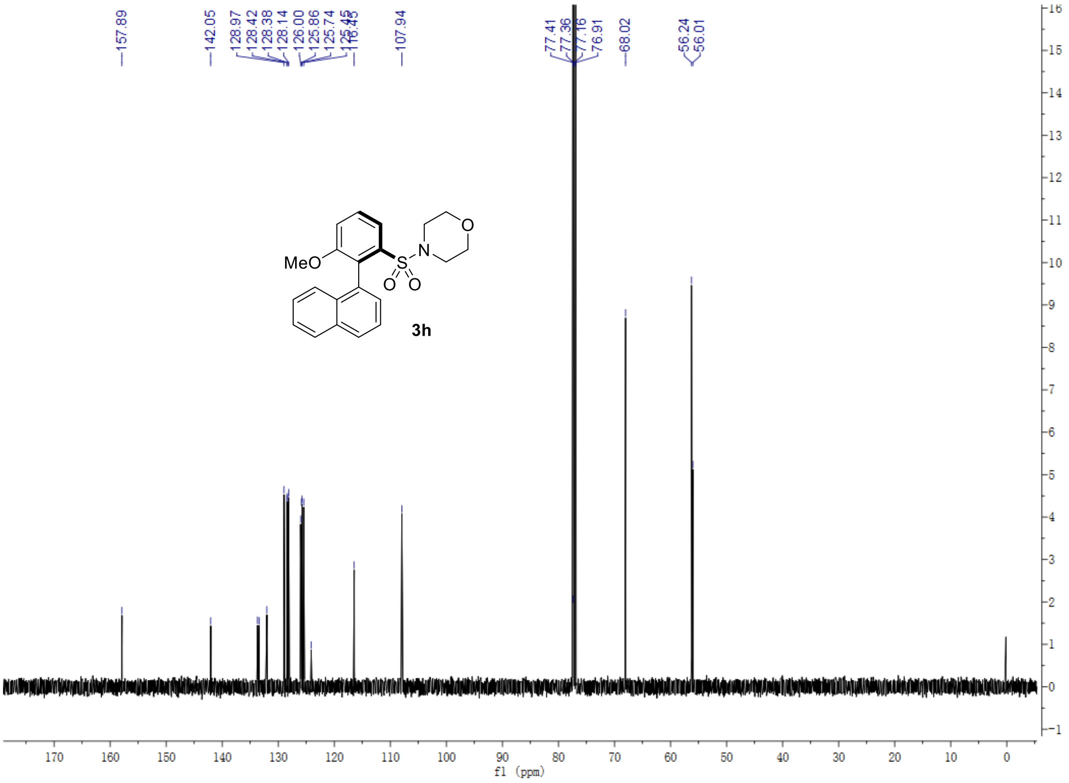

Supplementary Figure 97 ^{1}H NMR spectrum of **3i** (500 MHz, $CDCl_3$)

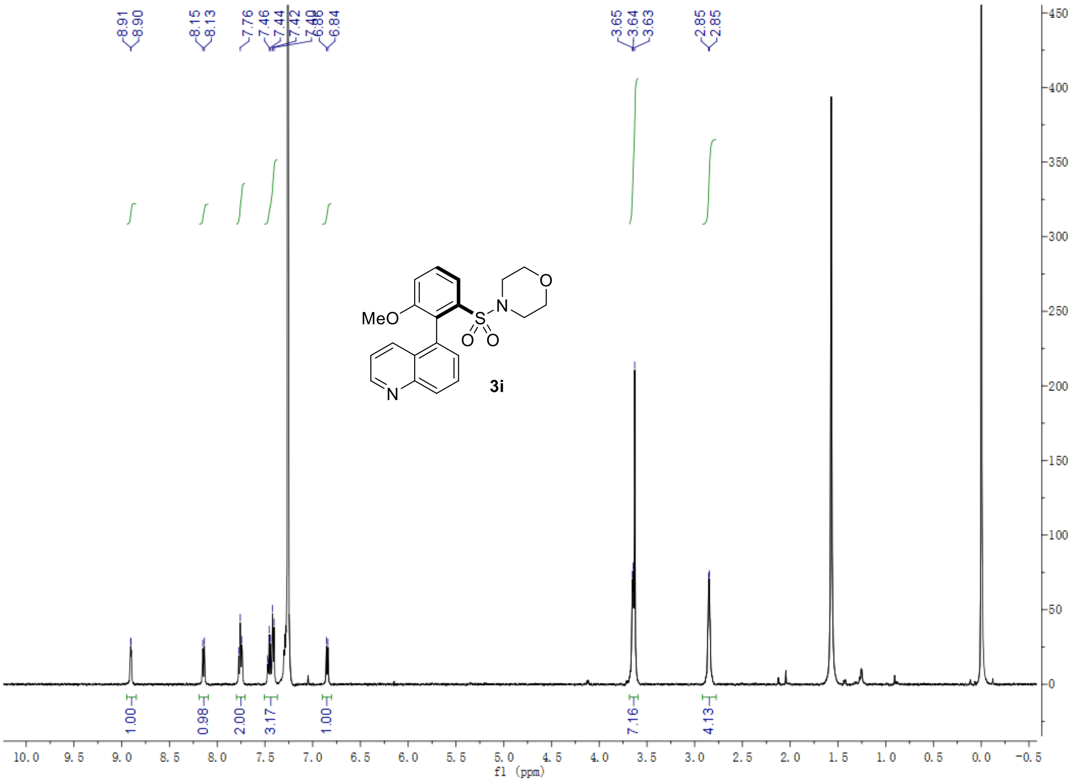

Supplementary Figure 98 ^{13}C NMR spectrum of **3i** (151 MHz, $CDCl_3$)

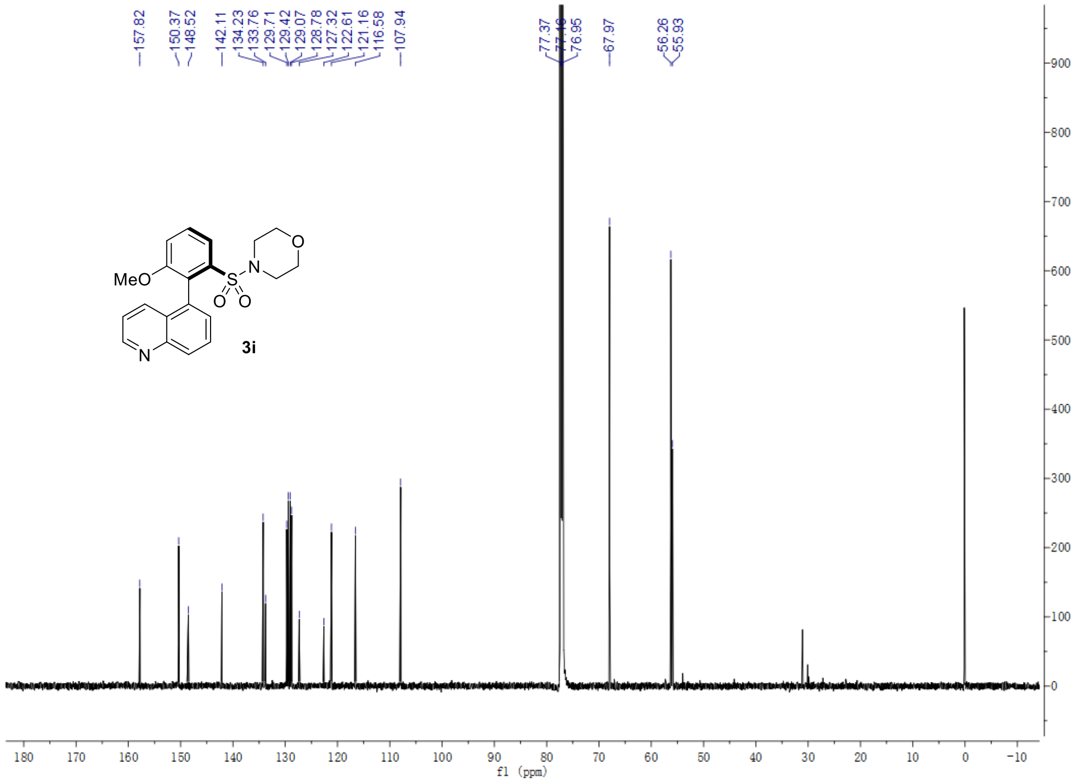

Supplementary Figure 99 ^{1}H NMR spectrum of **3j** (600 MHz, $CDCl_3$)

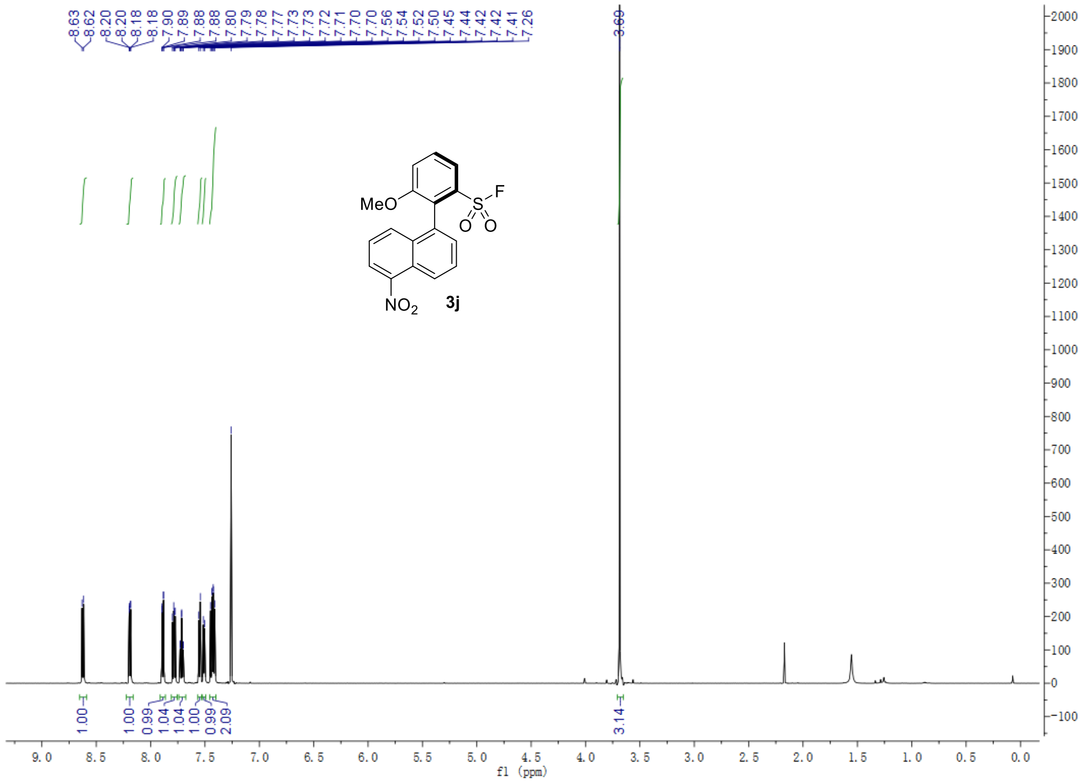

Supplementary Figure 100 ^{13}C NMR spectrum of **3j** (151 MHz, $CDCl_3$)

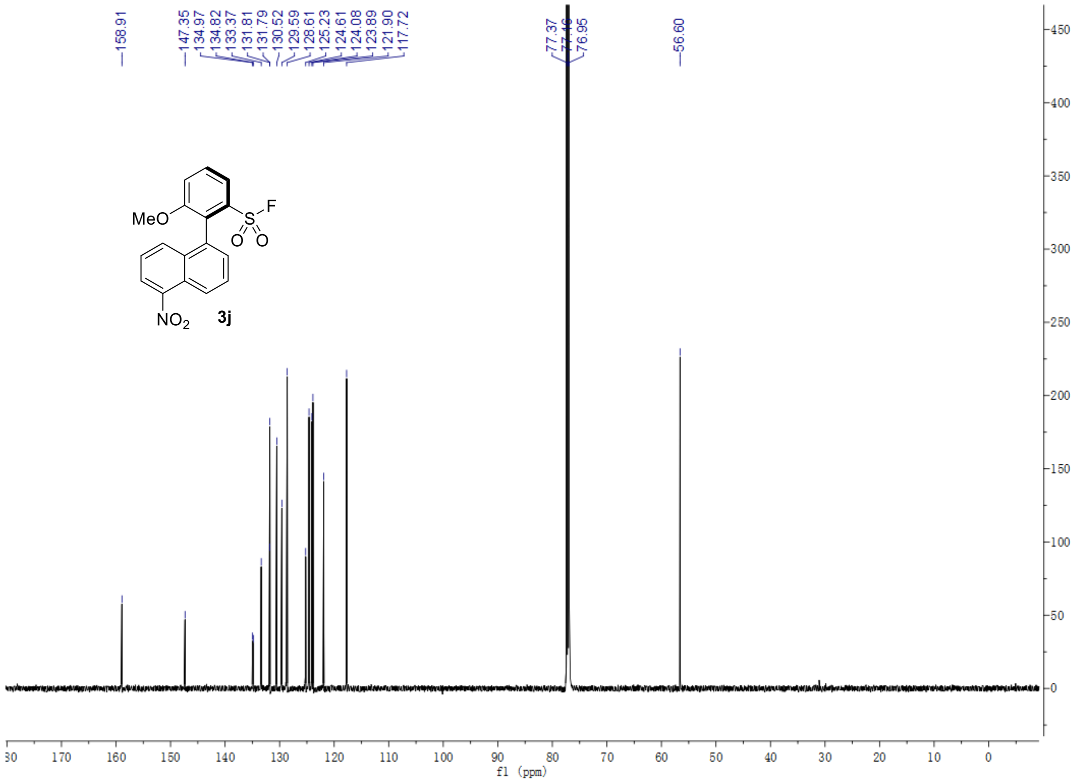

Supplementary Figure 101 ^{19}F NMR spectrum of **3j** (376 MHz, $CDCl_3$)

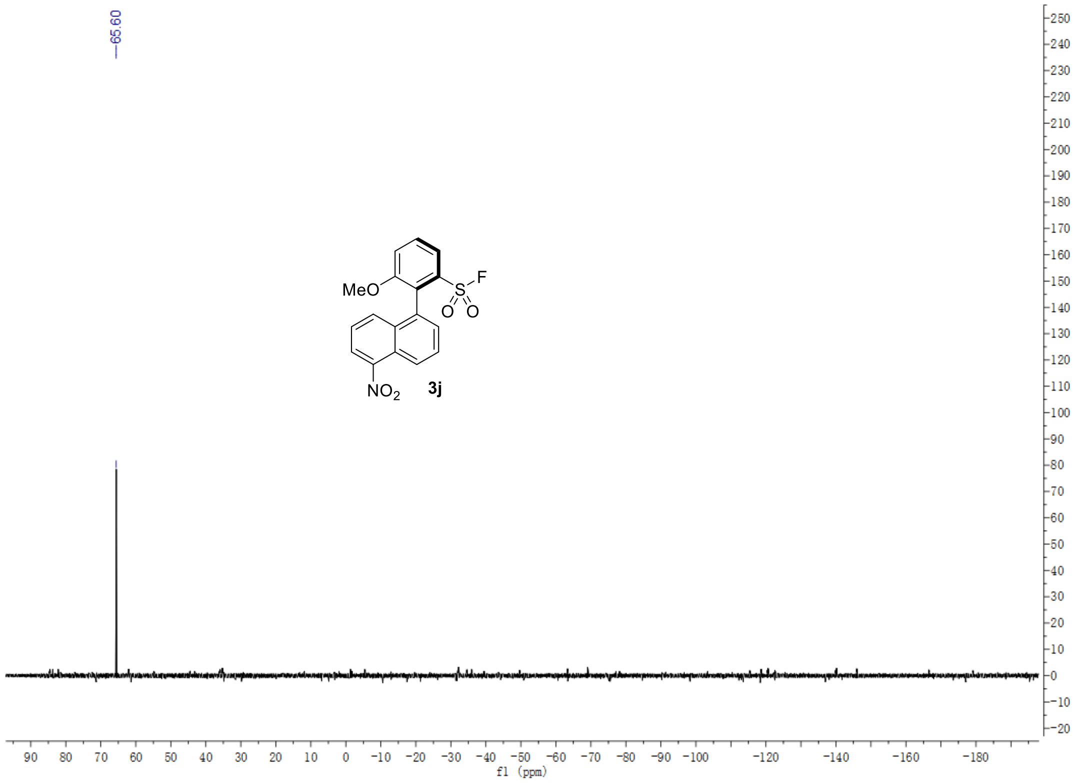

Supplementary Figure 102 ^{1}H NMR spectrum of **3k** (500 MHz, $CDCl_3$)

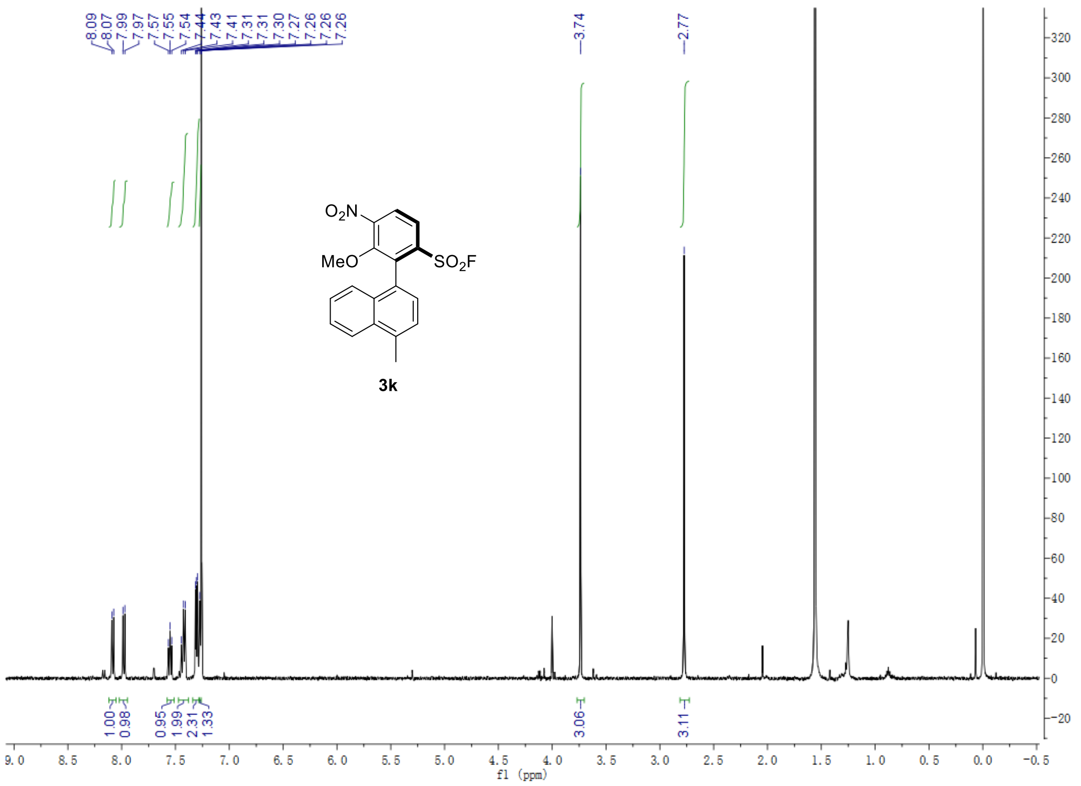

Supplementary Figure 103 ^{13}C NMR spectrum of **3k** (151 MHz, $CDCl_3$)

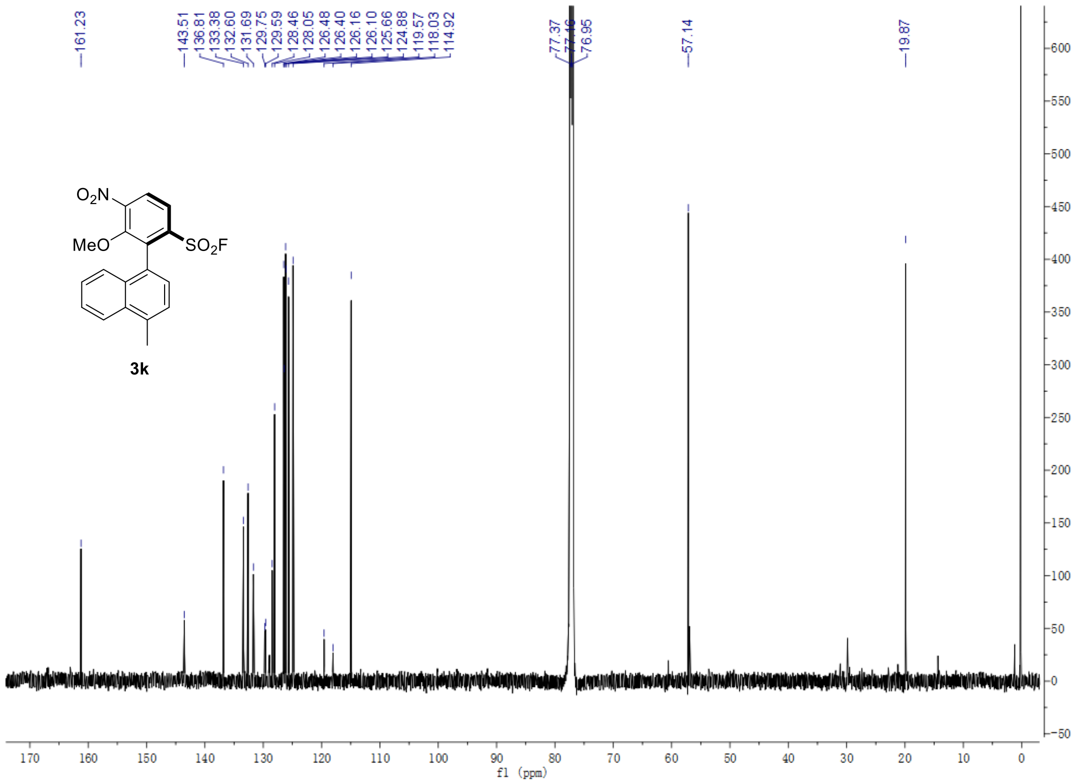

Supplementary Figure 104 ^{19}F NMR spectrum of **3k** (376 MHz, $CDCl_3$)

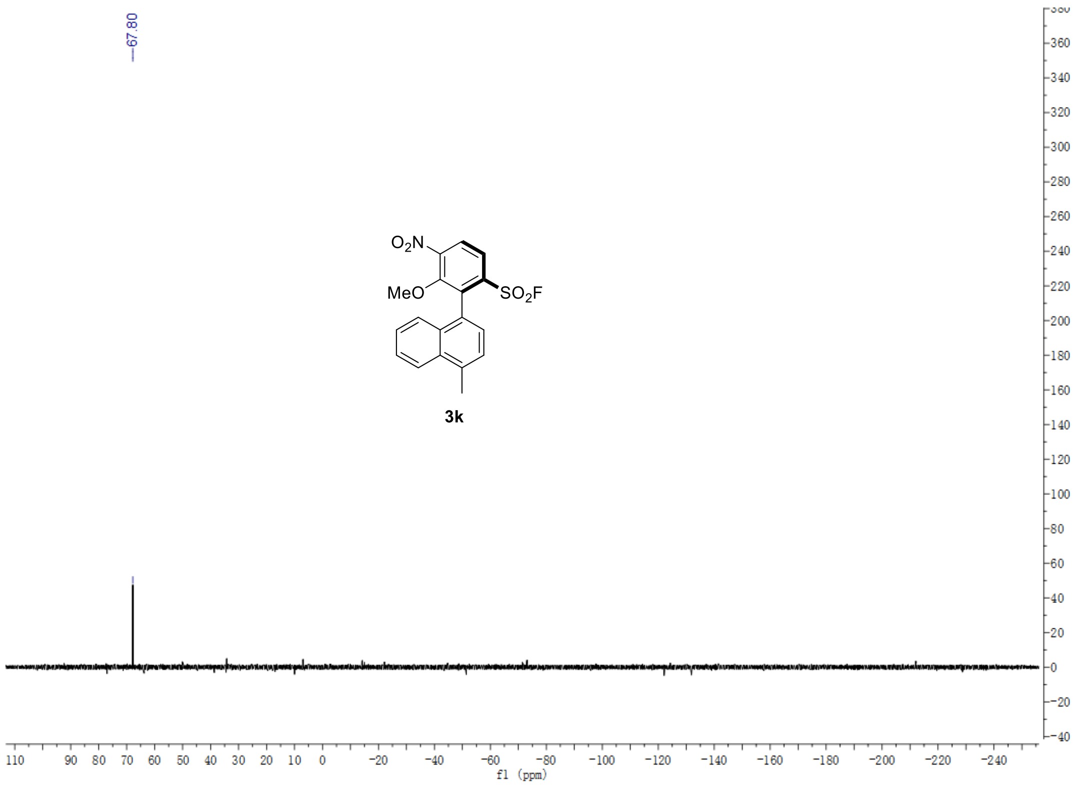

Supplementary Figure 105 ^{1}H NMR spectrum of **3l** (500 MHz, $CDCl_3$)

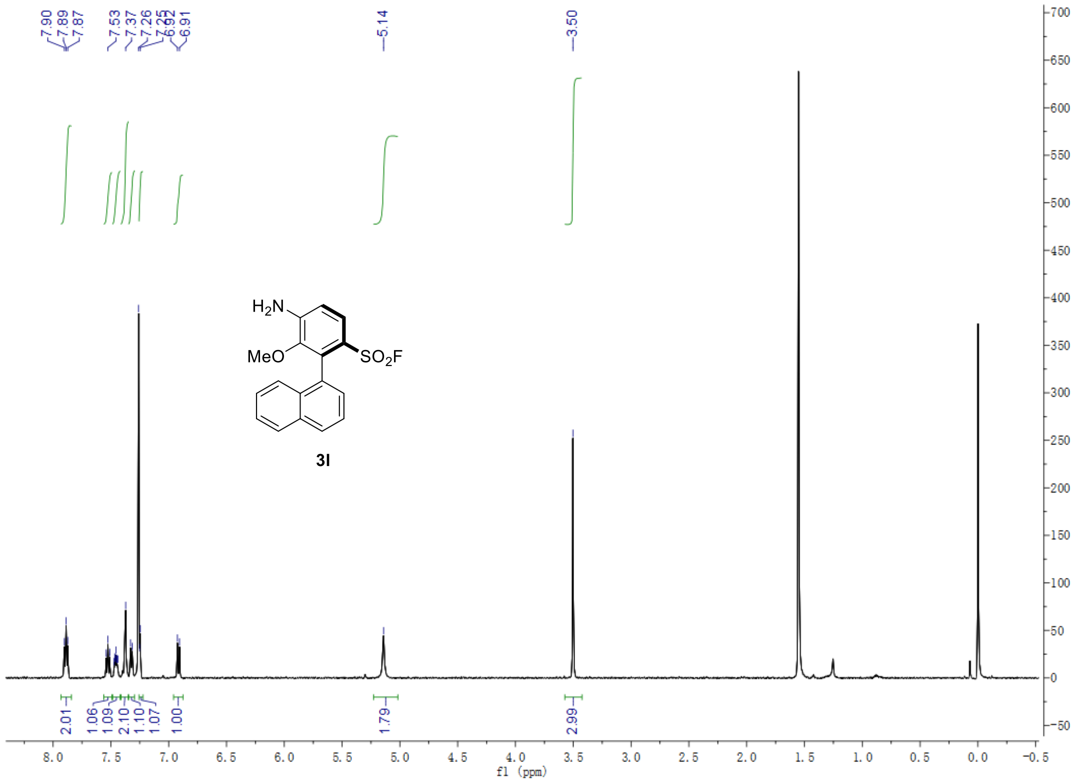

Supplementary Figure 106 ^{13}C NMR spectrum of **3l** (151 MHz, $CDCl_3$)

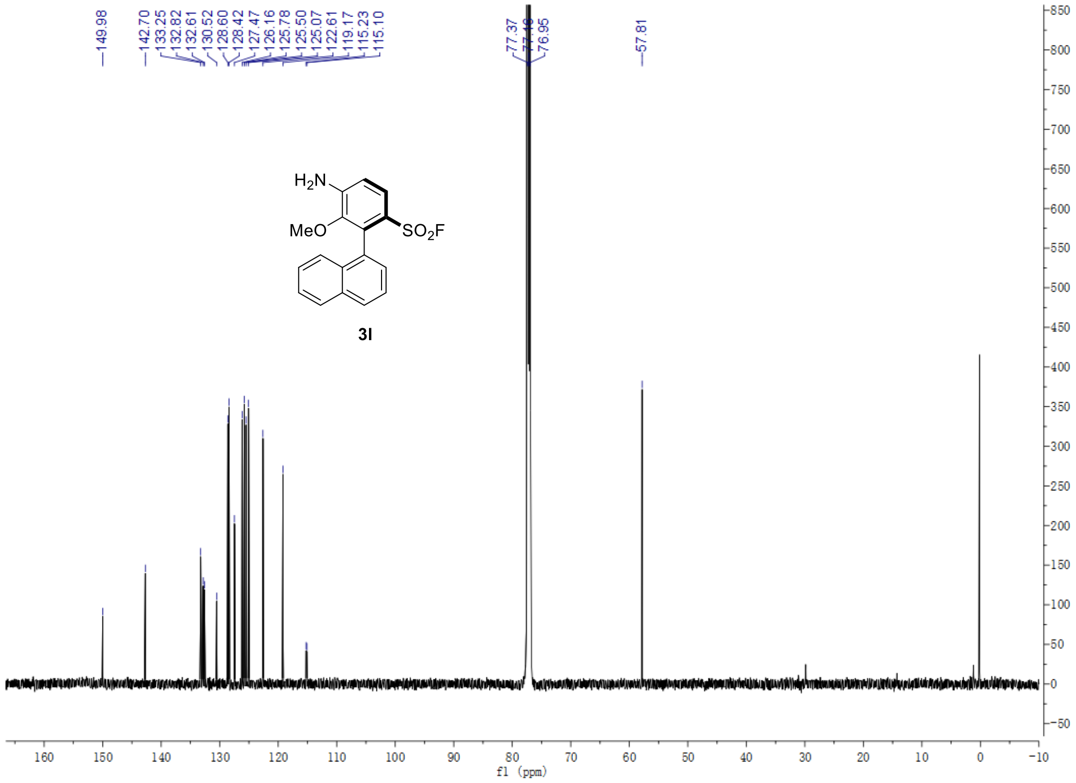

Supplementary Figure 107 ^{19}F NMR spectrum of **3l** (376 MHz, $CDCl_3$)

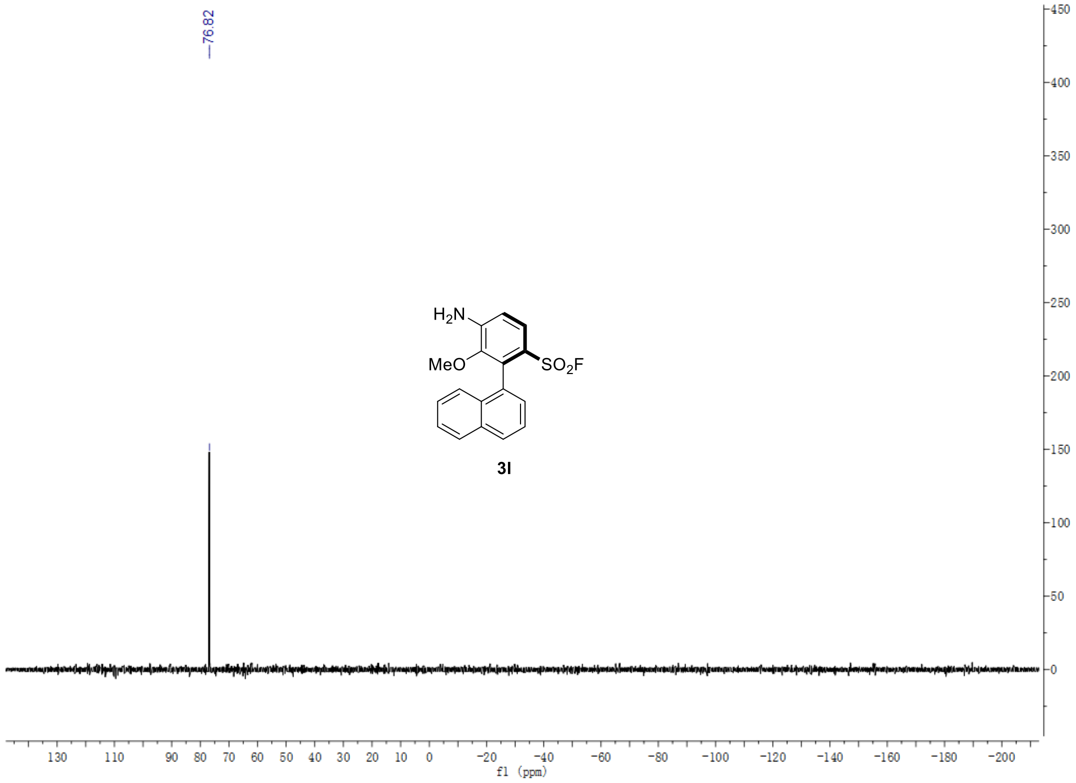

Supplementary Figure 108 ^{1}H NMR spectrum of **3m** (400 MHz, $CDCl_3$)

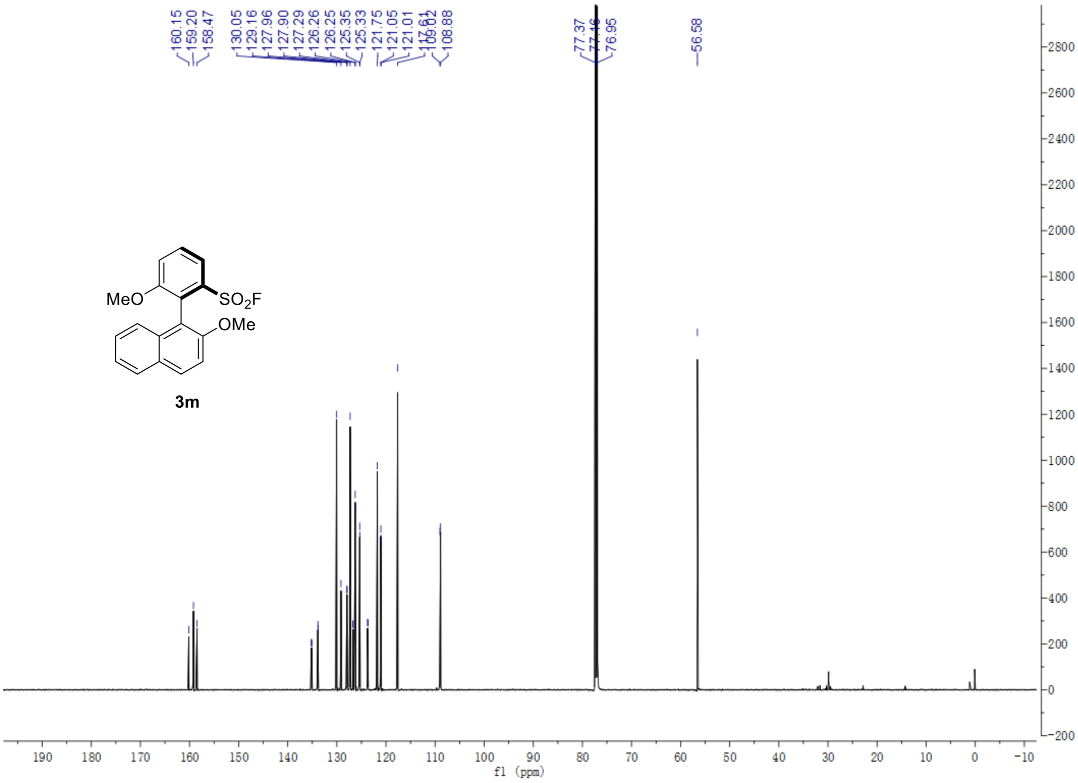

Supplementary Figure 109 ^{13}C NMR spectrum of **3m** (151 MHz, $CDCl_3$)

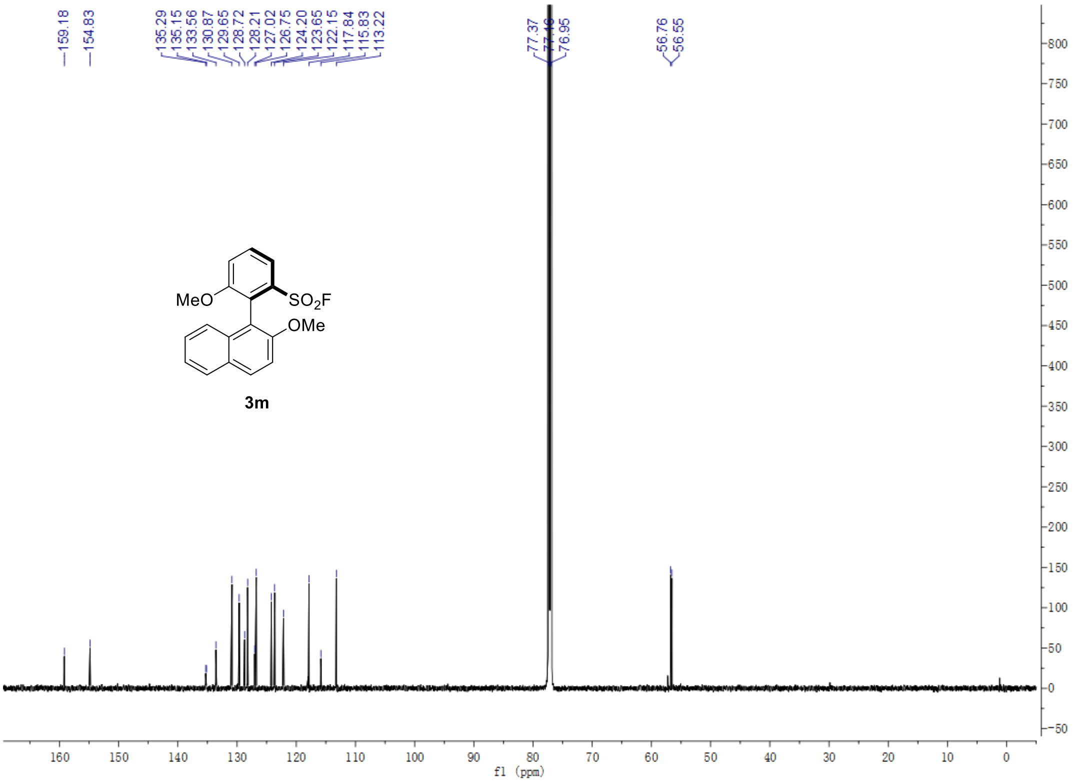

Supplementary Figure 110 ^{19}F NMR spectrum of **3m** (376 MHz, $CDCl_3$)

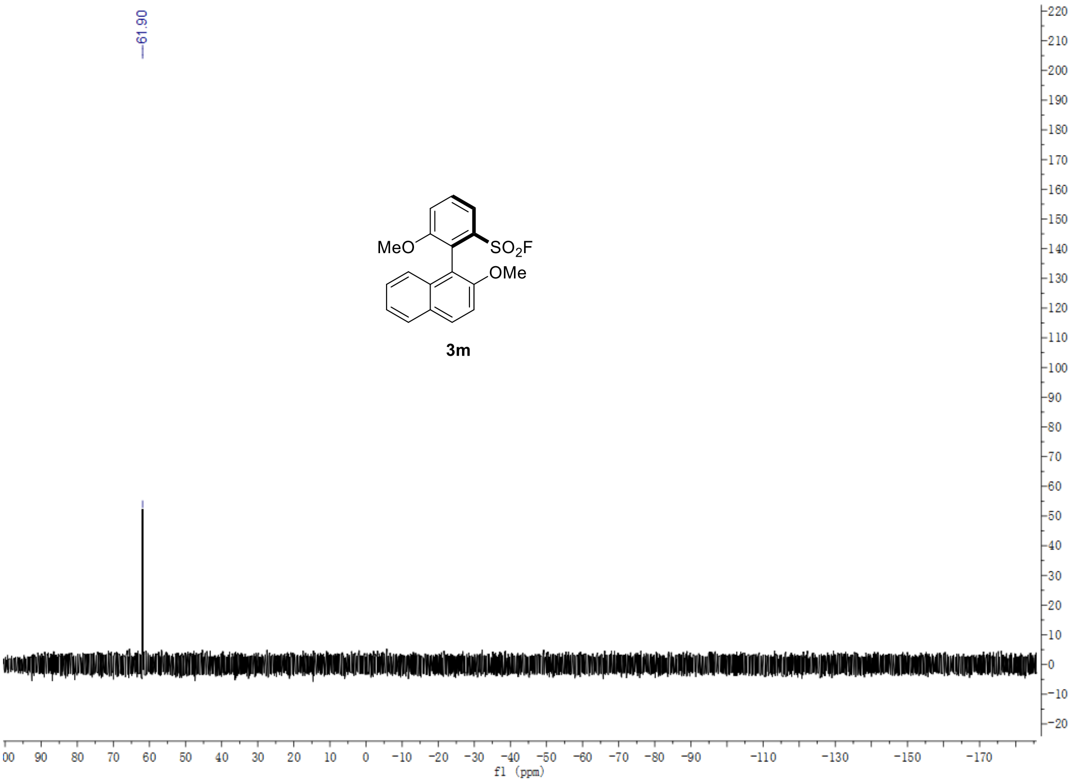

Supplementary Figure 111 ^{1}H NMR spectrum of **3n** (500 MHz, $CDCl_3$)

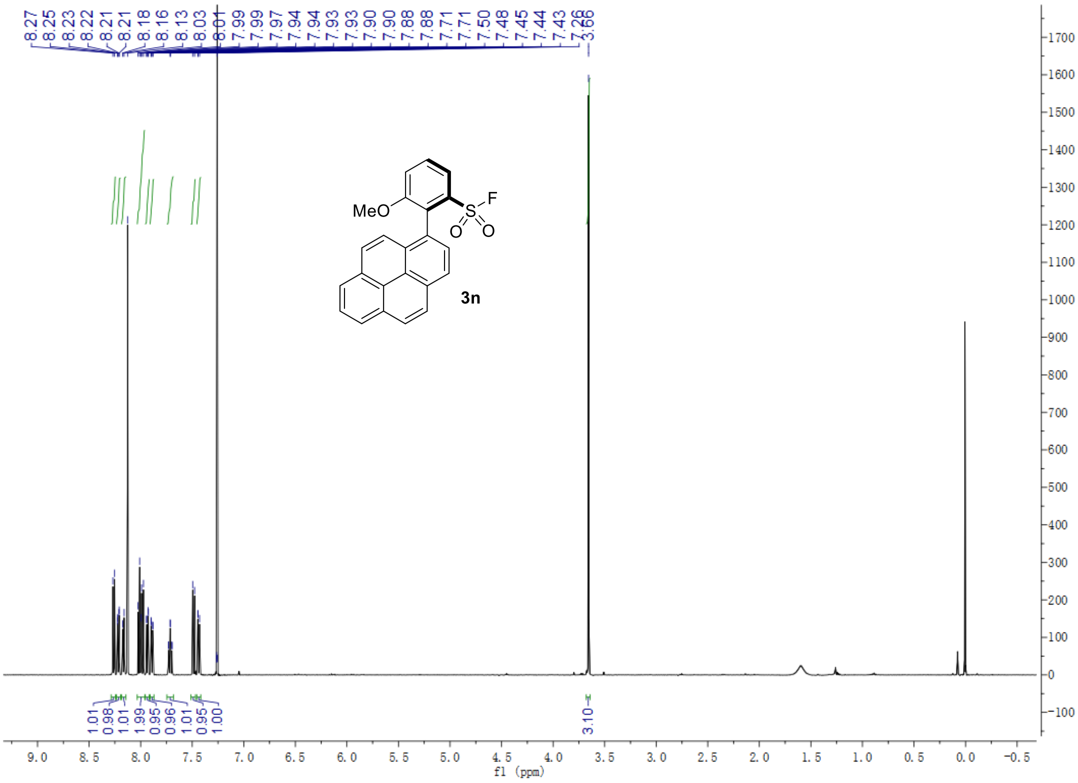

Supplementary Figure 112 ^{13}C NMR spectrum of **3n** (151 MHz, $CDCl_3$)

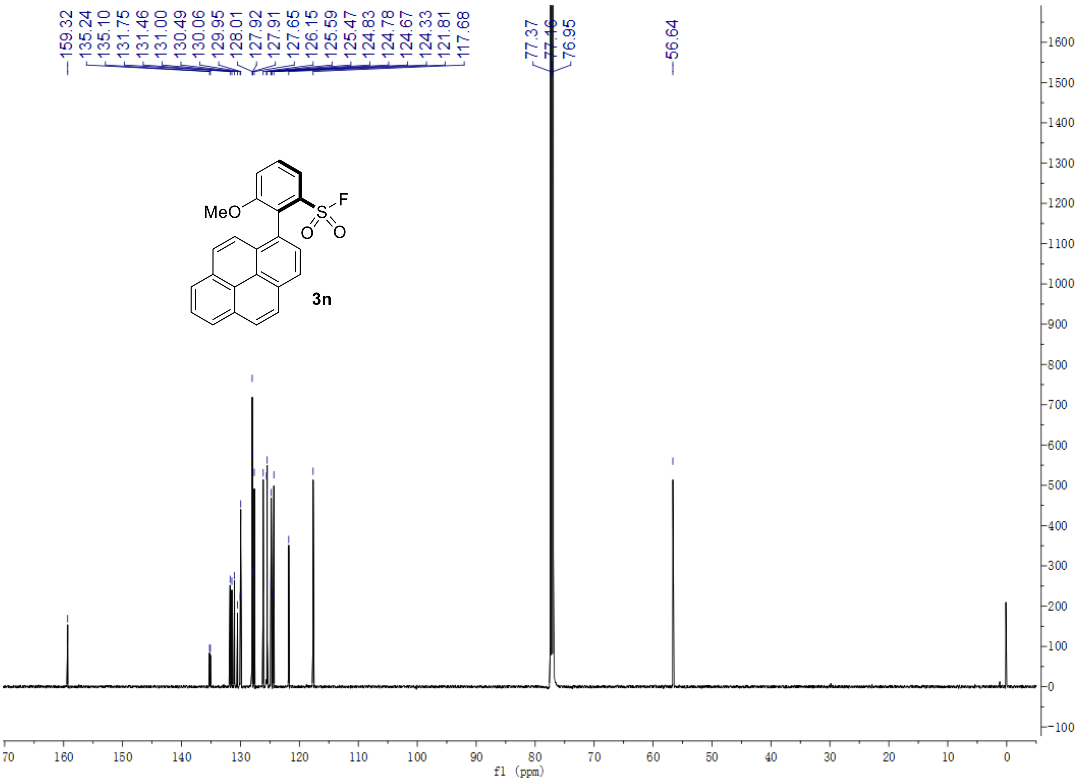

Supplementary Figure 113 ^{19}F NMR spectrum of **3n** (376 MHz, $CDCl_3$)

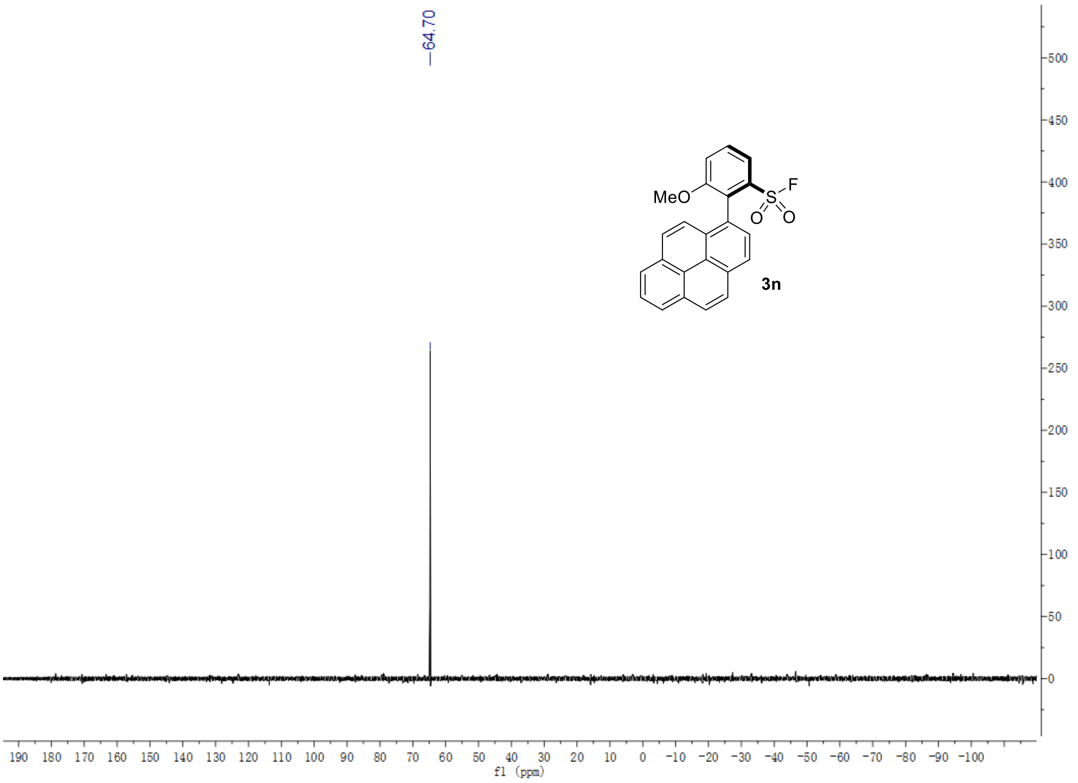

Supplementary Figure 114 ^{1}H NMR spectrum of **3o** (500 MHz, $CDCl_3$)

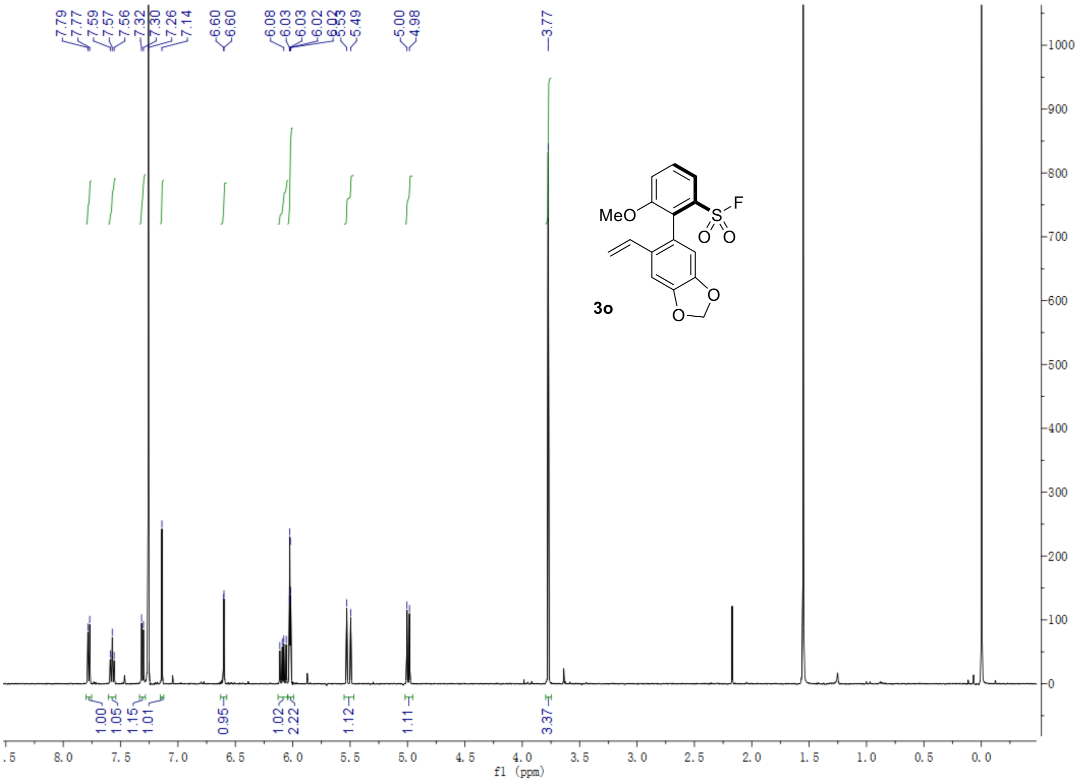

Supplementary Figure 115 ^{13}C NMR spectrum of **3o** (151 MHz, $CDCl_3$)

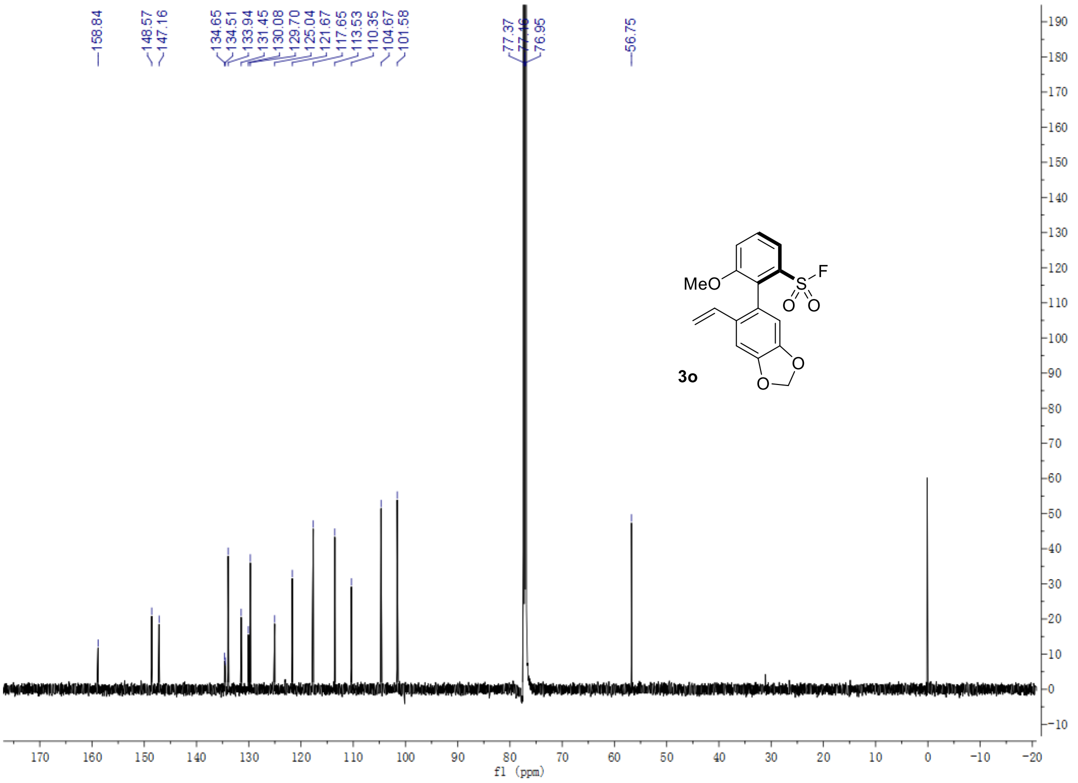

Supplementary Figure 116 ^{19}F NMR spectrum of **3o** (376 MHz, $CDCl_3$)

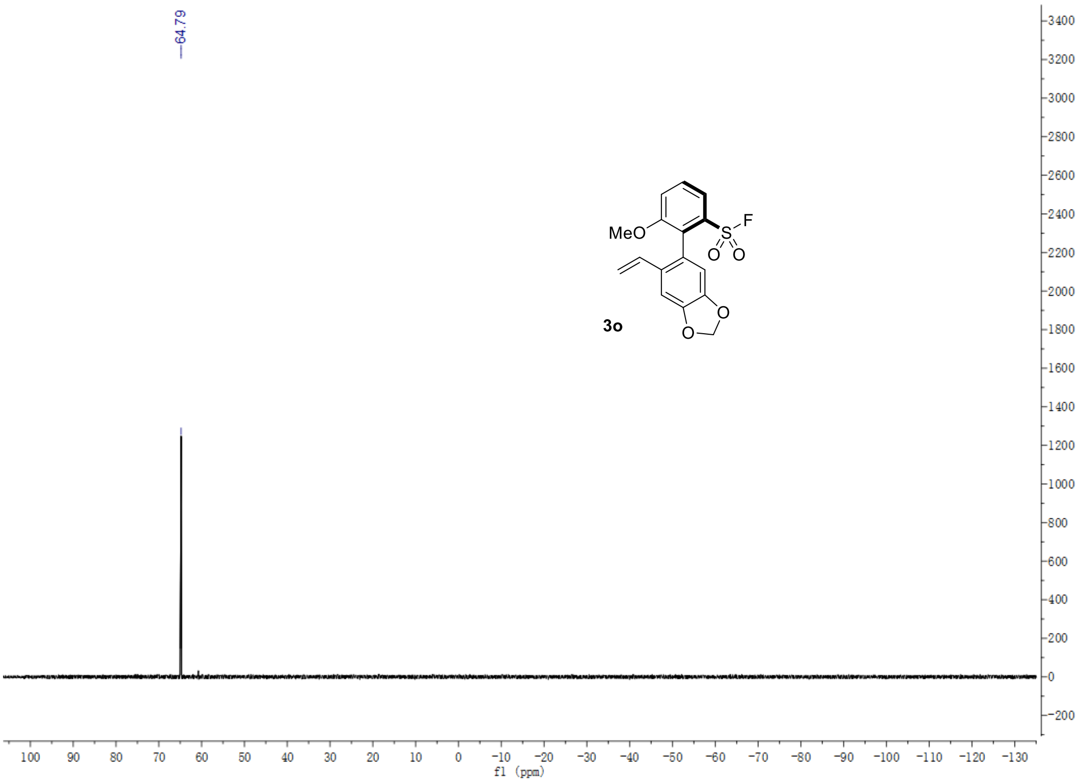

Supplementary Figure 117 ^{1}H NMR spectrum of **3p** (500 MHz, $CDCl_3$)

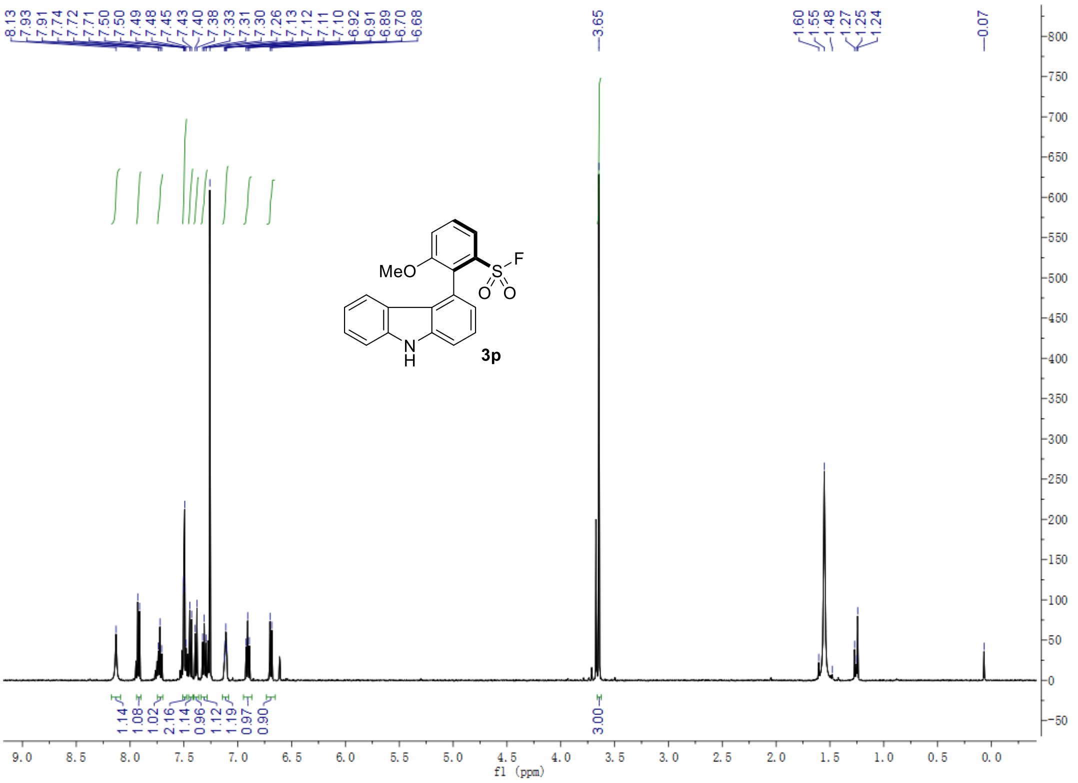

Supplementary Figure 118 ^{13}C NMR spectrum of **3p** (151 MHz, $CDCl_3$)

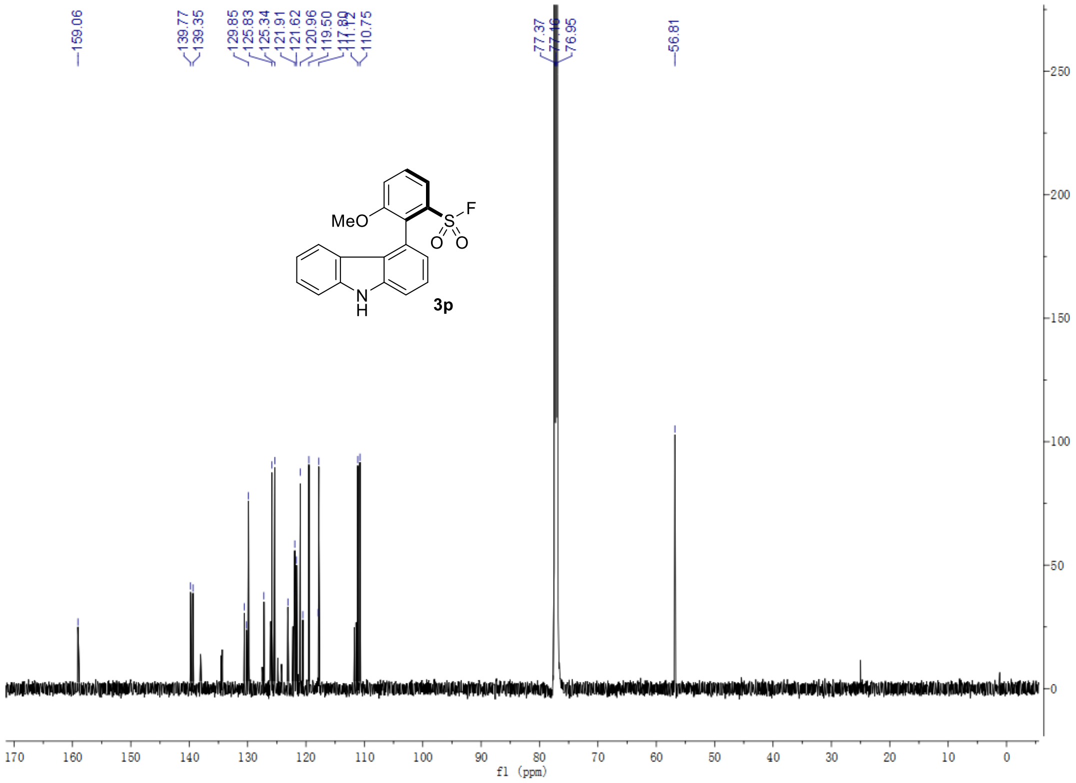

Supplementary Figure 119 ^{19}F NMR spectrum of **3p** (376 MHz, $CDCl_3$)

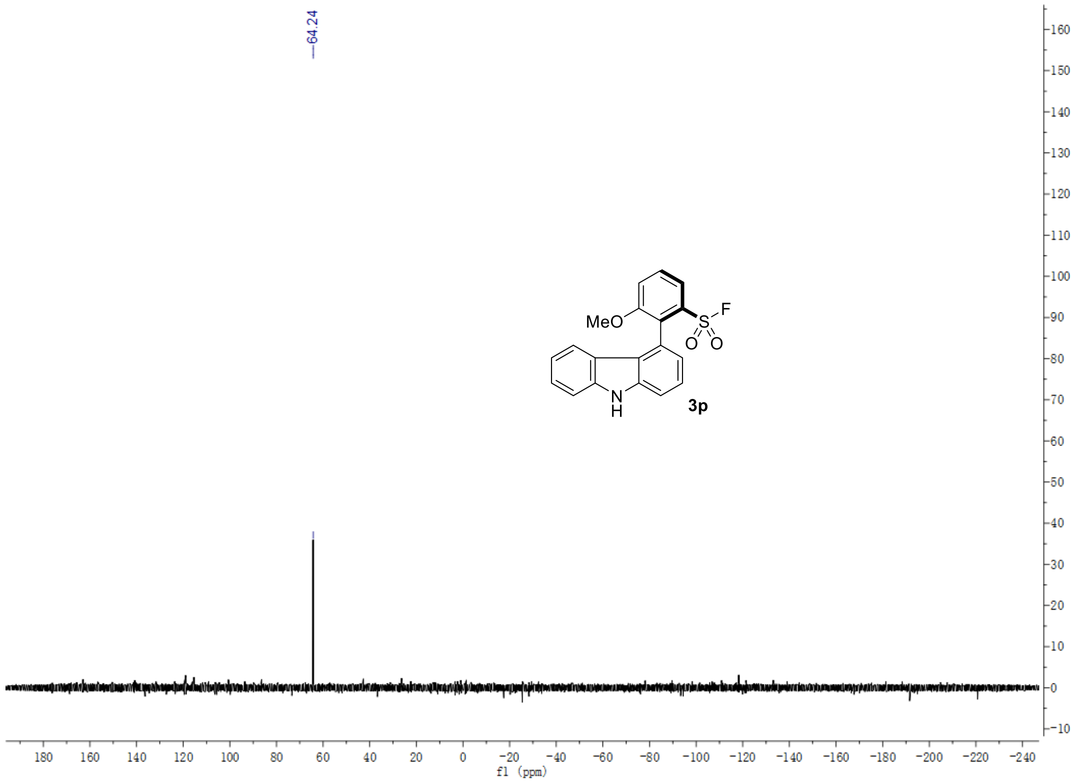

Supplementary Figure 120 ^{1}H NMR spectrum of **3q** (400 MHz, $CDCl_3$)

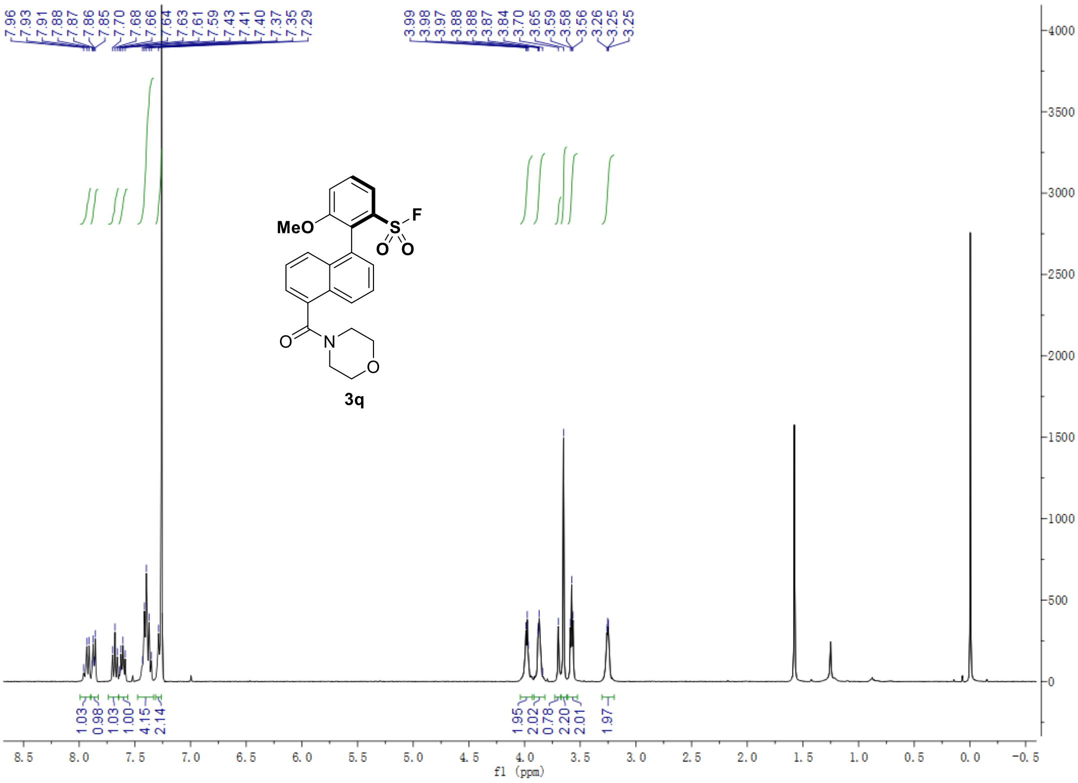

Supplementary Figure 121 ^{13}C NMR spectrum of **3q** (151 MHz, $CDCl_3$)

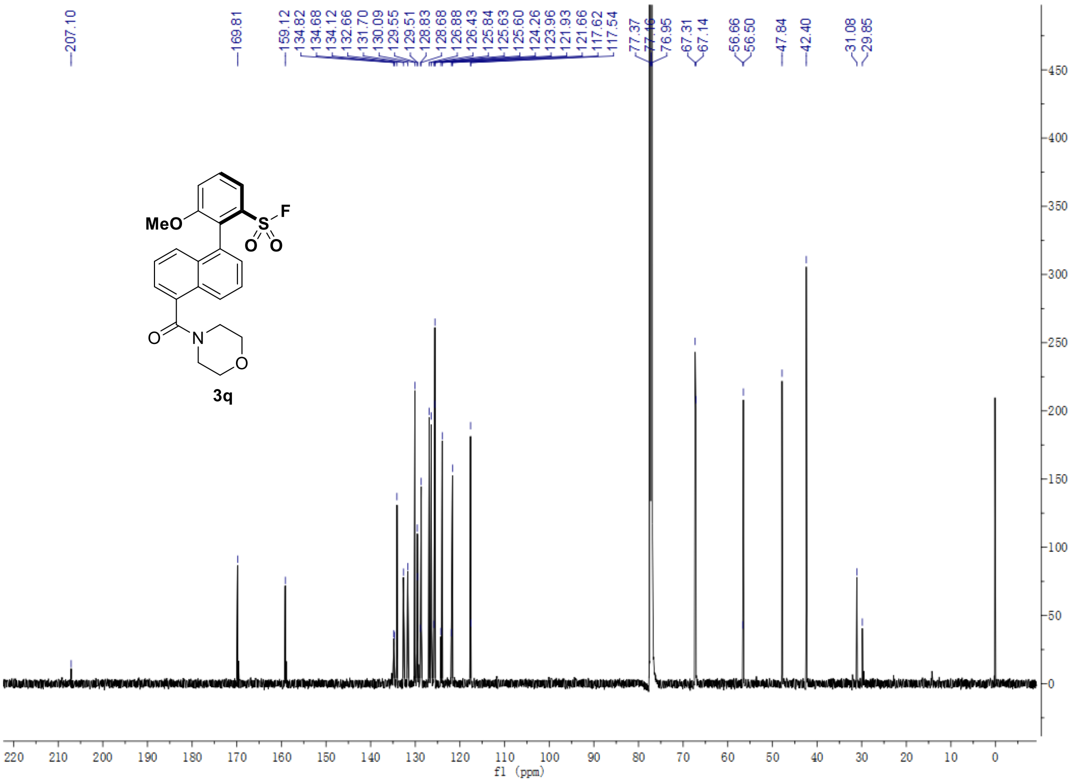

Supplementary Figure 122 ^{19}F NMR spectrum of **3q** (376 MHz, $CDCl_3$)

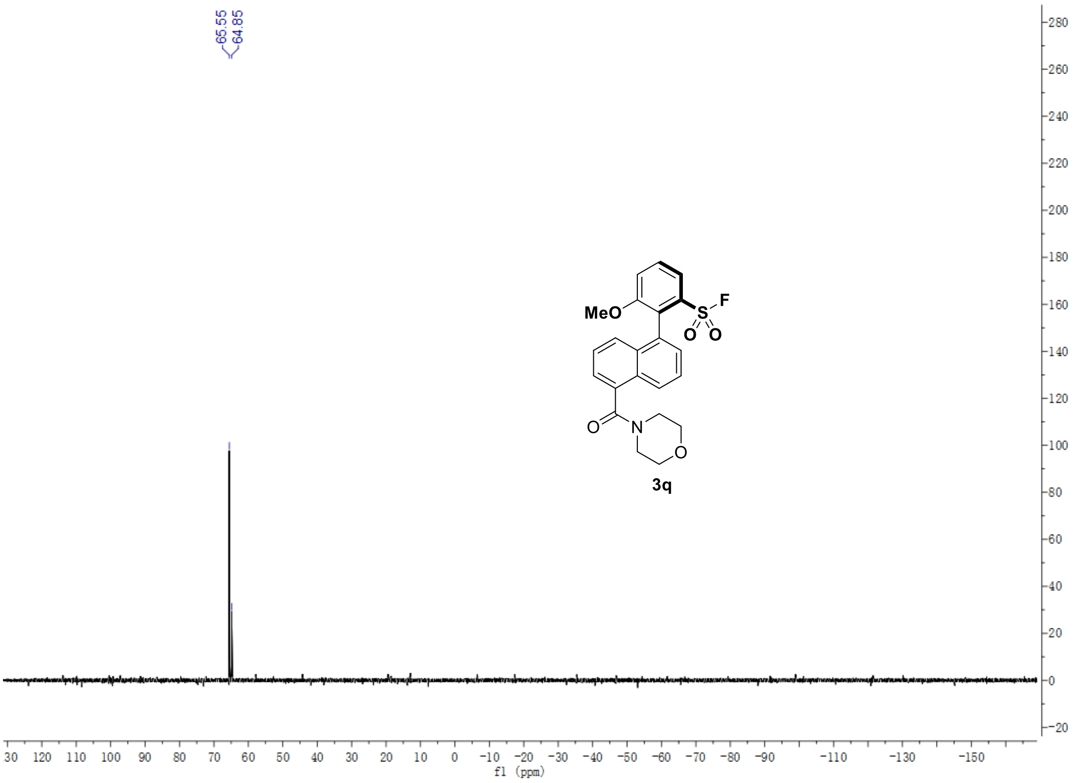

Supplementary Figure 123 ^{1}H NMR spectrum of **S31** (400 MHz, $CDCl_3$)

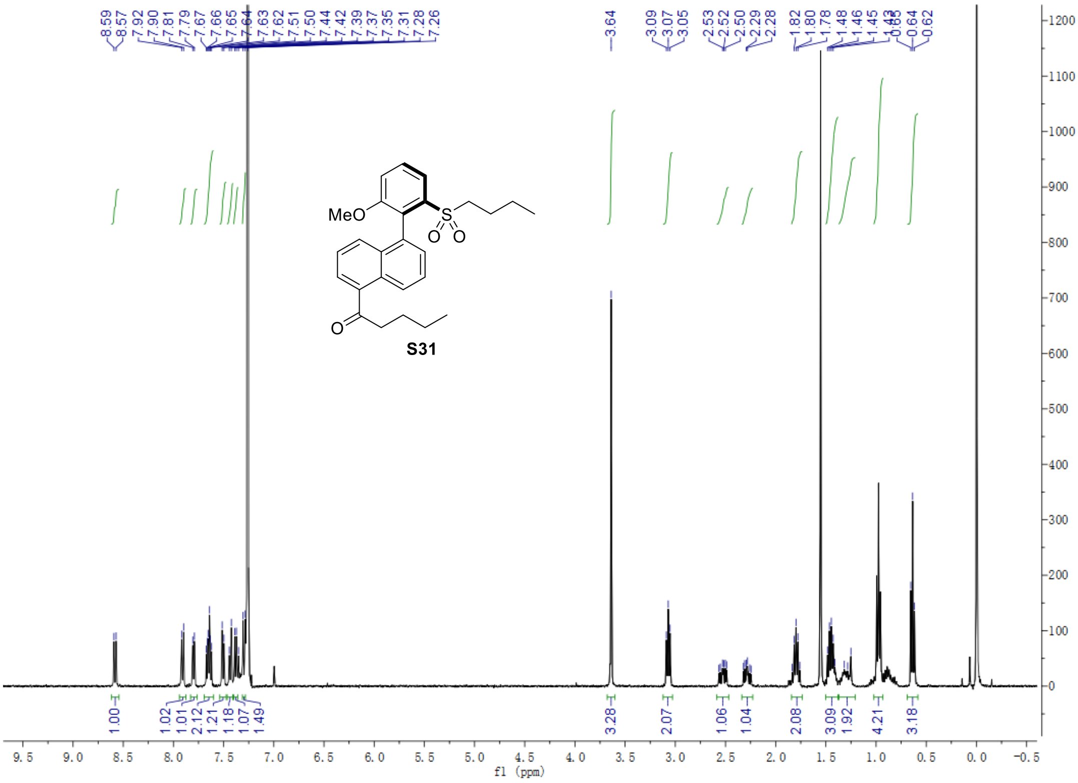

Supplementary Figure 124 ^{13}C NMR spectrum of **S31** (151 MHz, $CDCl_3$)

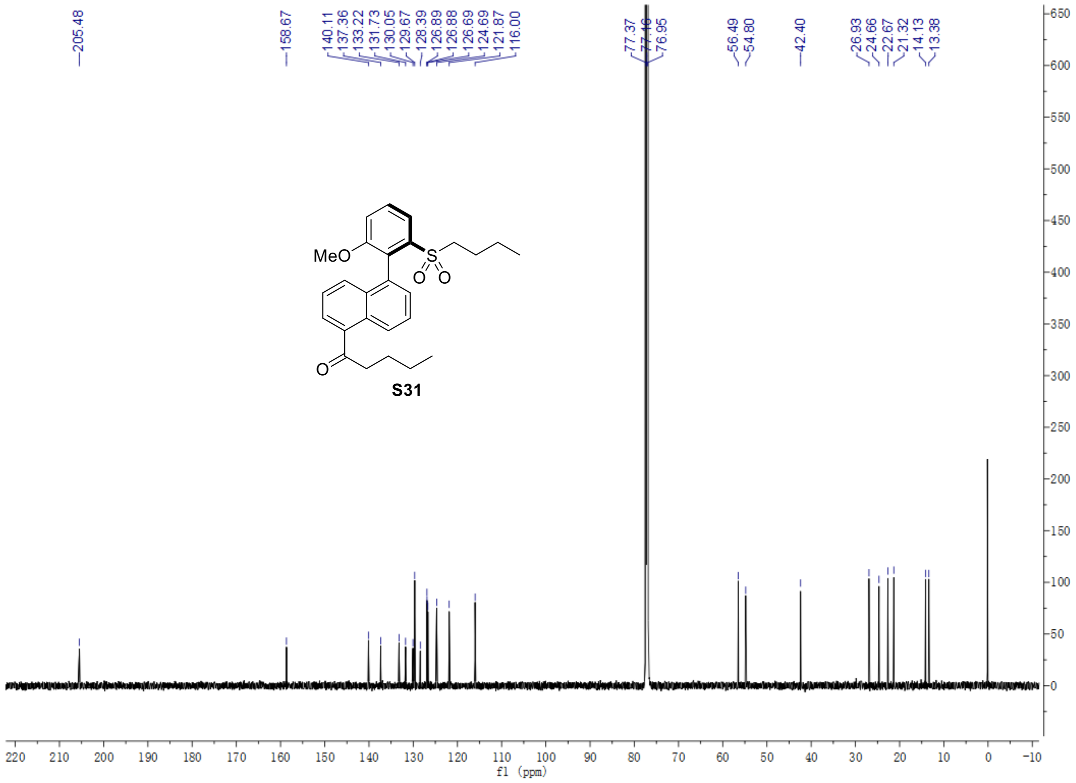

Supplementary Figure 125 ^{1}H NMR spectrum of **3r** (500 MHz, $CDCl_3$)

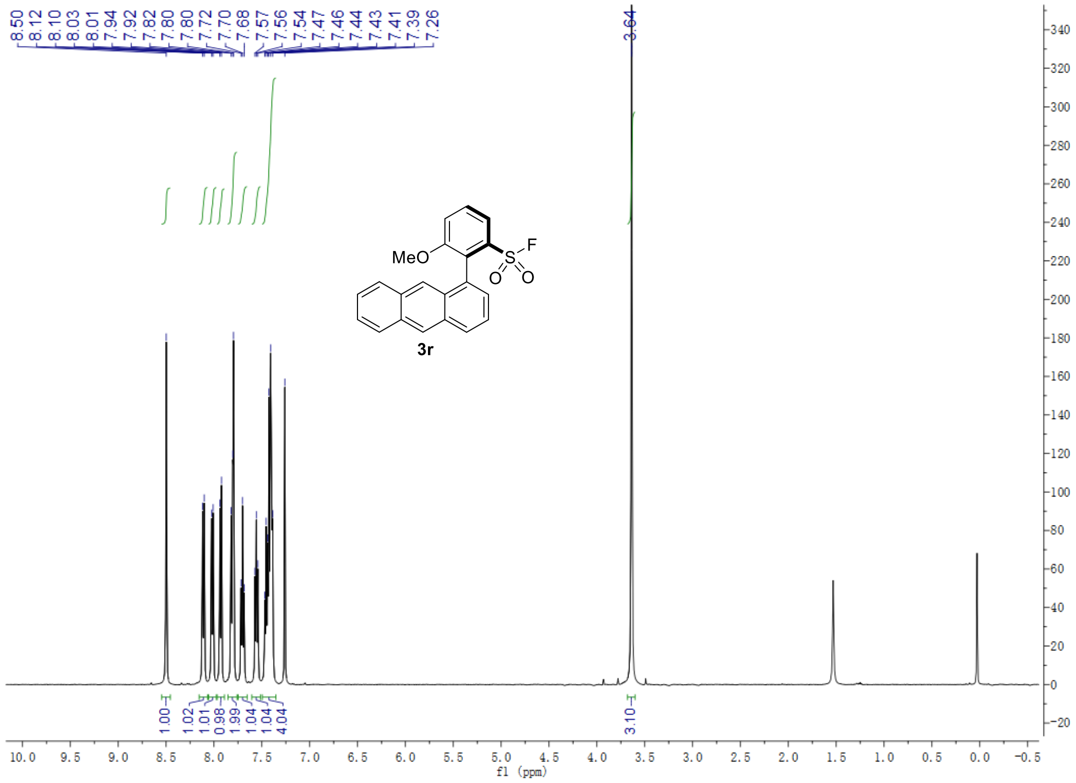

Supplementary Figure 126 ^{13}C NMR spectrum of **3r** (151 MHz, $CDCl_3$)

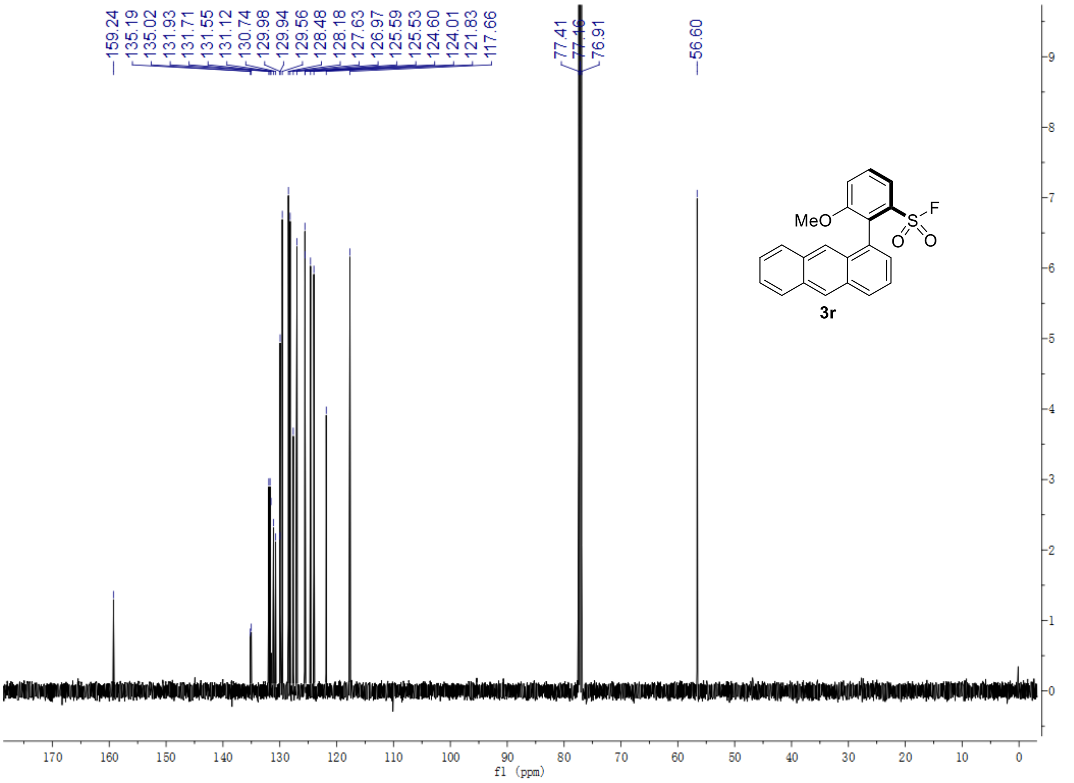

Supplementary Figure 127 ^{19}F NMR spectrum of **3r** (376 MHz, $CDCl_3$)

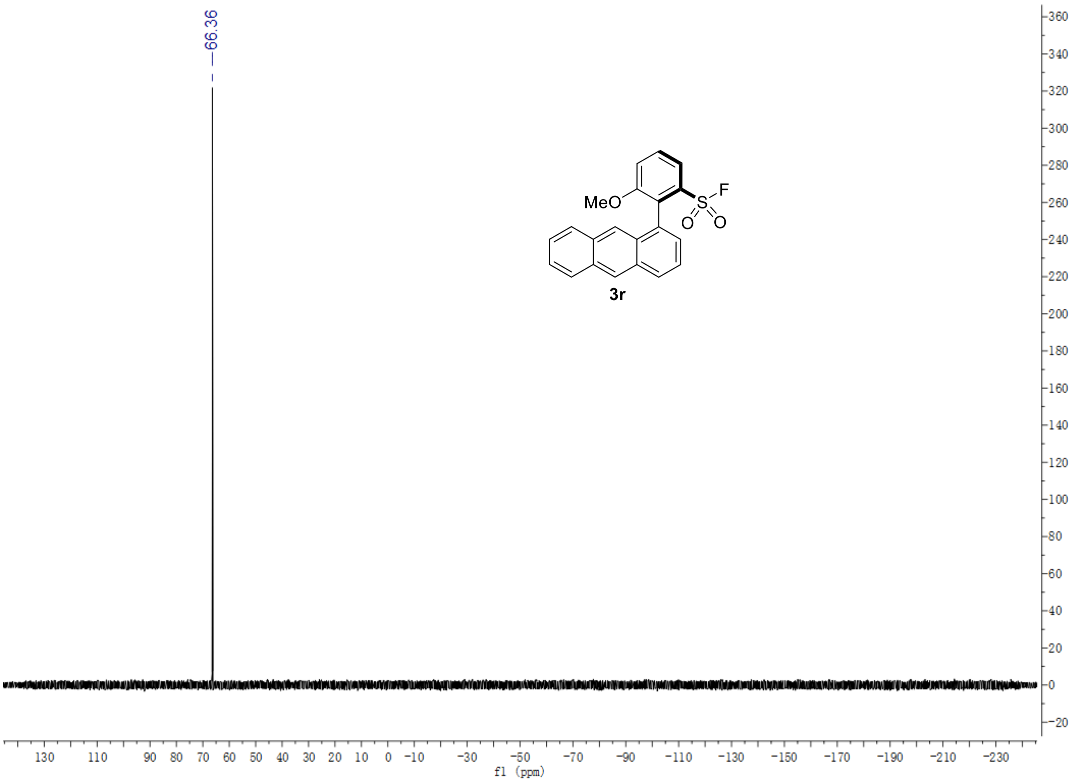

Supplementary Figure 128 ^{1}H NMR spectrum of **3s** (400 MHz, $CDCl_3$)

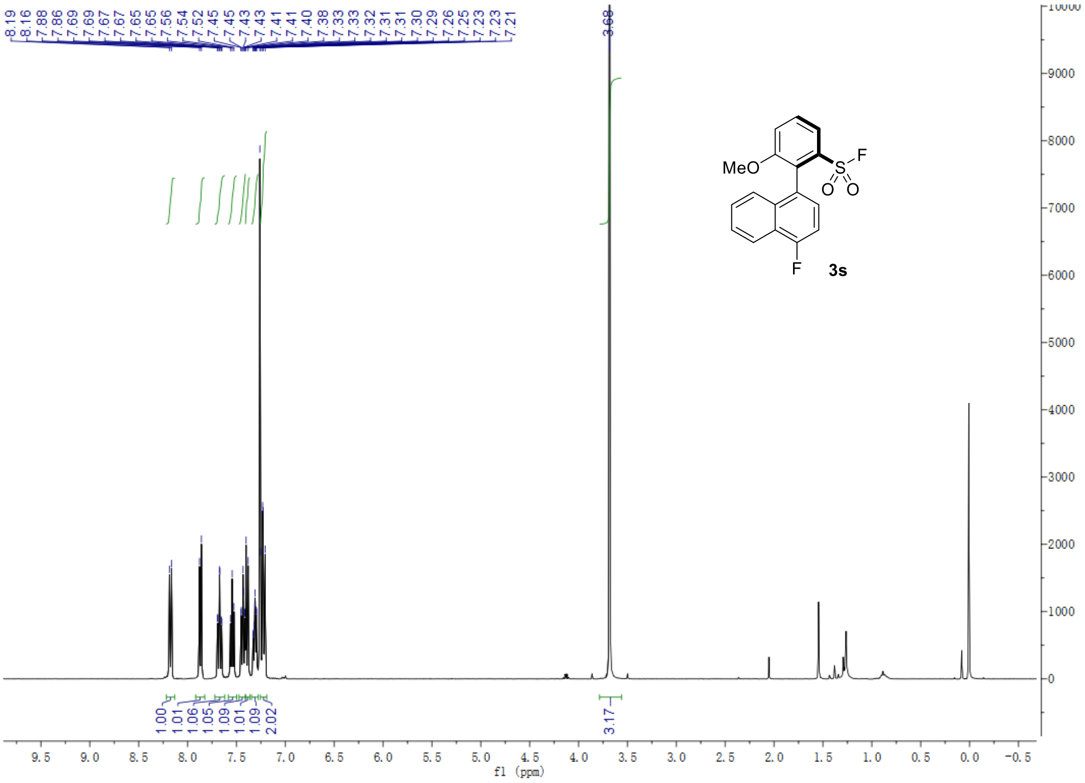

Supplementary Figure 129 ^{13}C NMR spectrum of **3s** (151 MHz, $CDCl_3$)

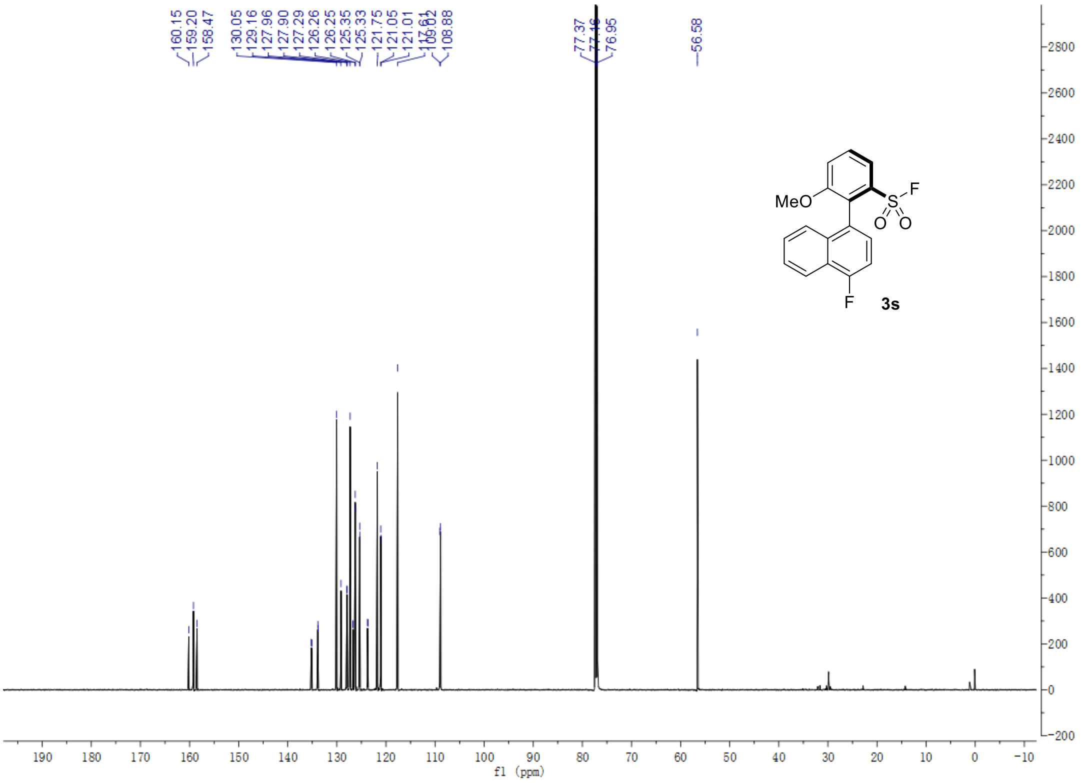

Supplementary Figure 130 ^{19}F NMR spectrum of **3s** (376 MHz, $CDCl_3$)

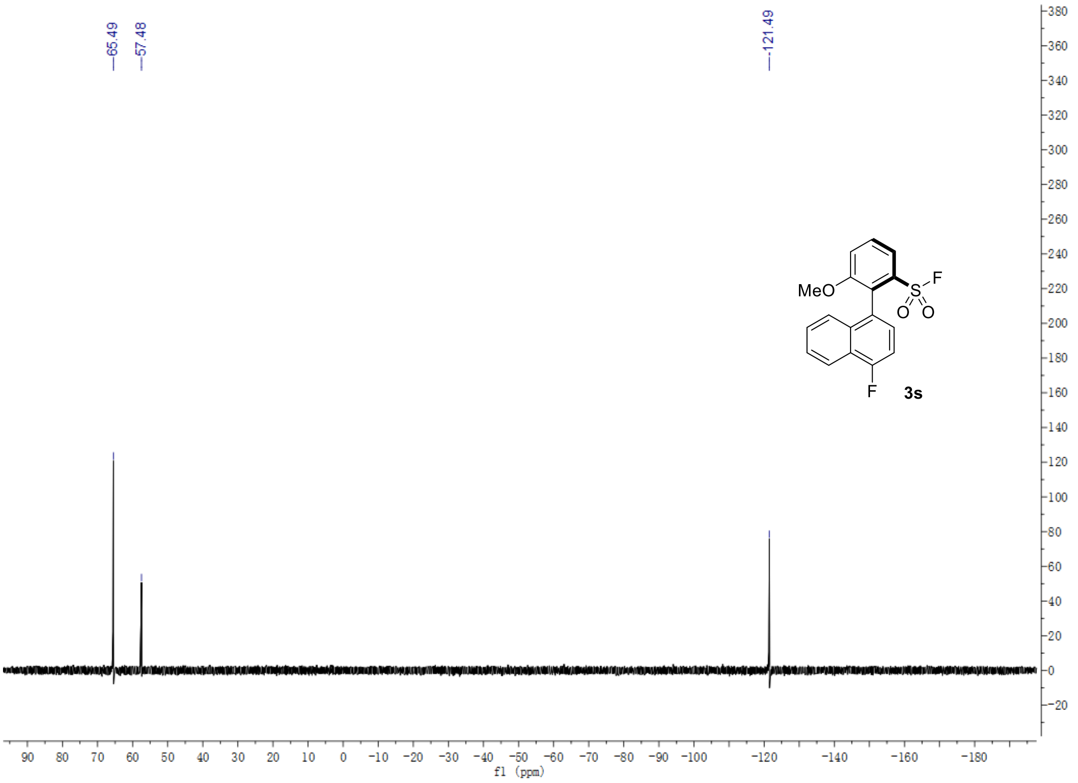

Supplementary Figure 131 ^{1}H NMR spectrum of **6a** (500 MHz, $CDCl_3$)

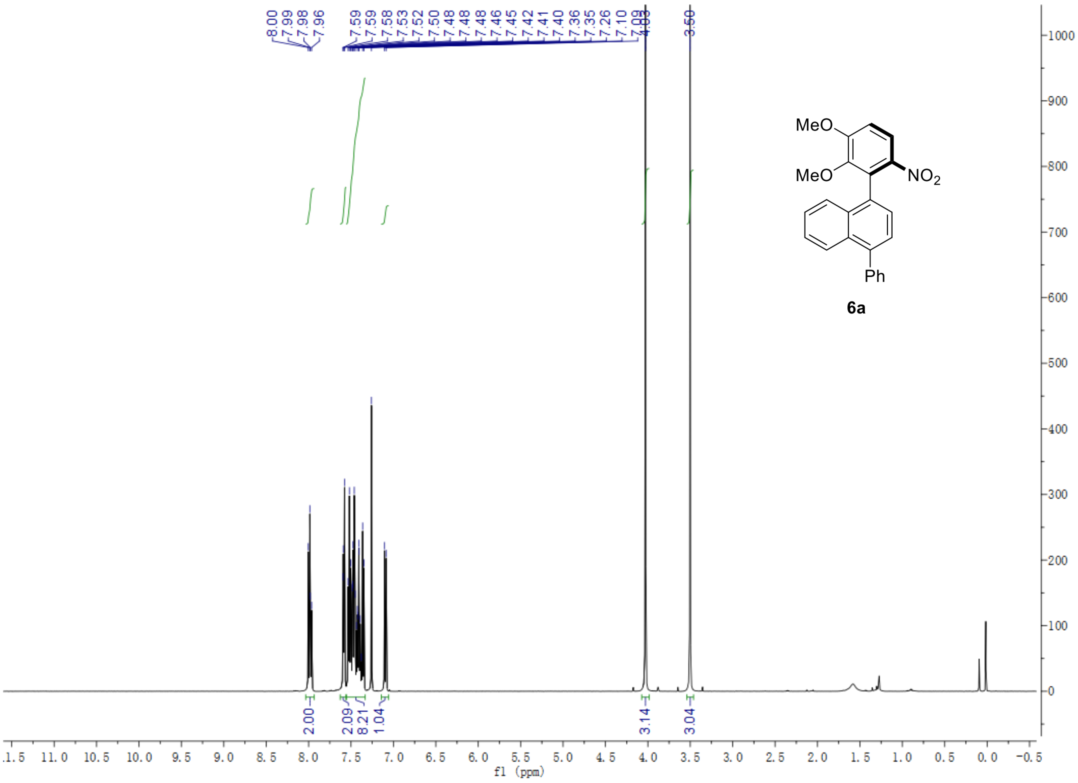

Supplementary Figure 132 ^{13}C NMR spectrum of **6a** (126 MHz, $CDCl_3$)

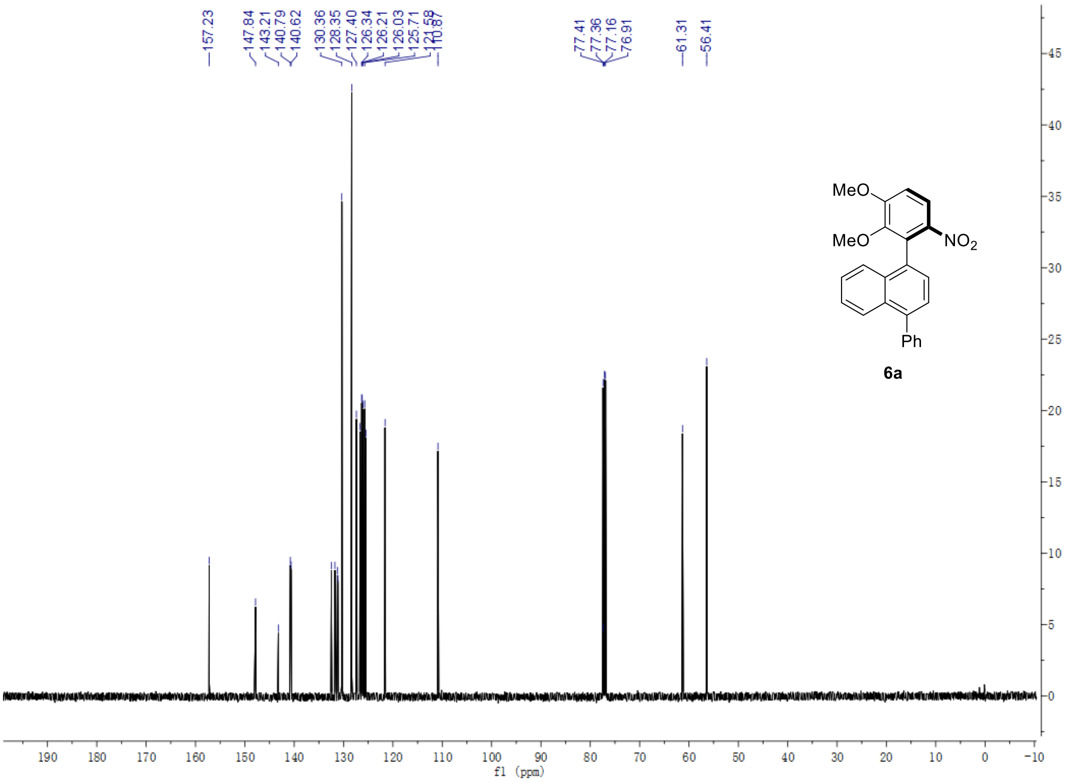

Supplementary Figure 133 ^{1}H NMR spectrum of **6b** (500 MHz, $CDCl_3$)

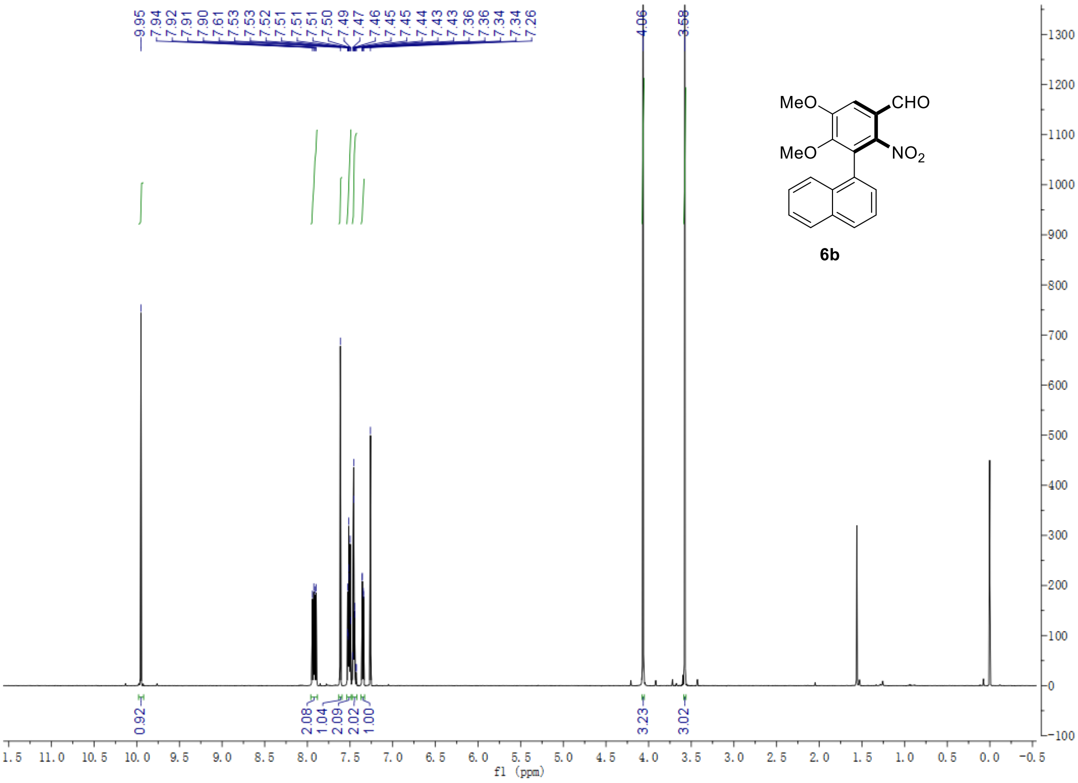

Supplementary Figure 134 ^{13}C NMR spectrum of **6b** (151 MHz, $CDCl_3$)

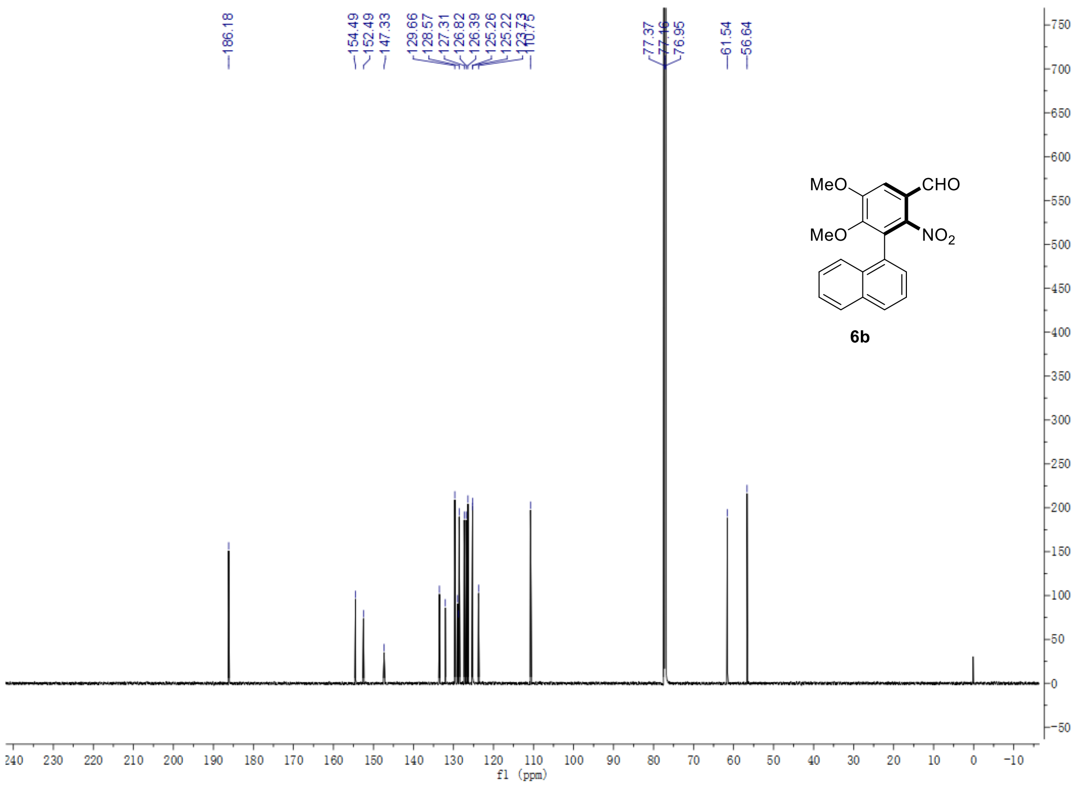

Supplementary Figure 135 ^{1}H NMR spectrum of **6c** (500 MHz, $CDCl_3$)

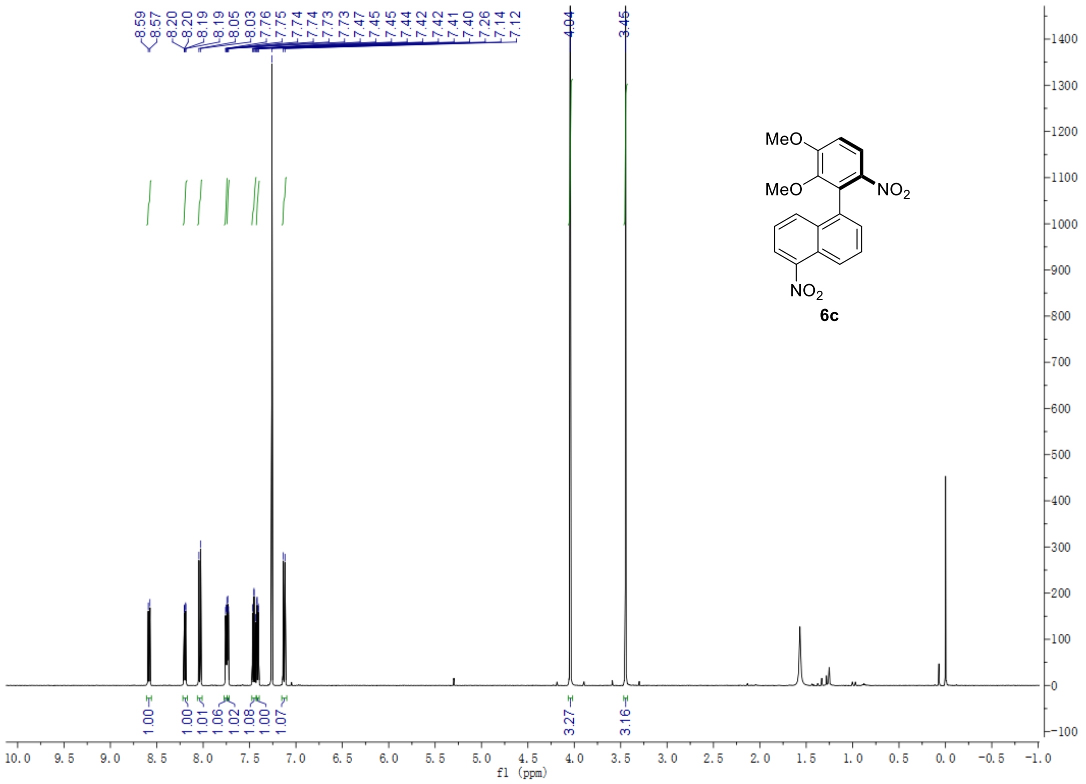

Supplementary Figure 136 ^{13}C NMR spectrum of **6c** (126 MHz, $CDCl_3$)

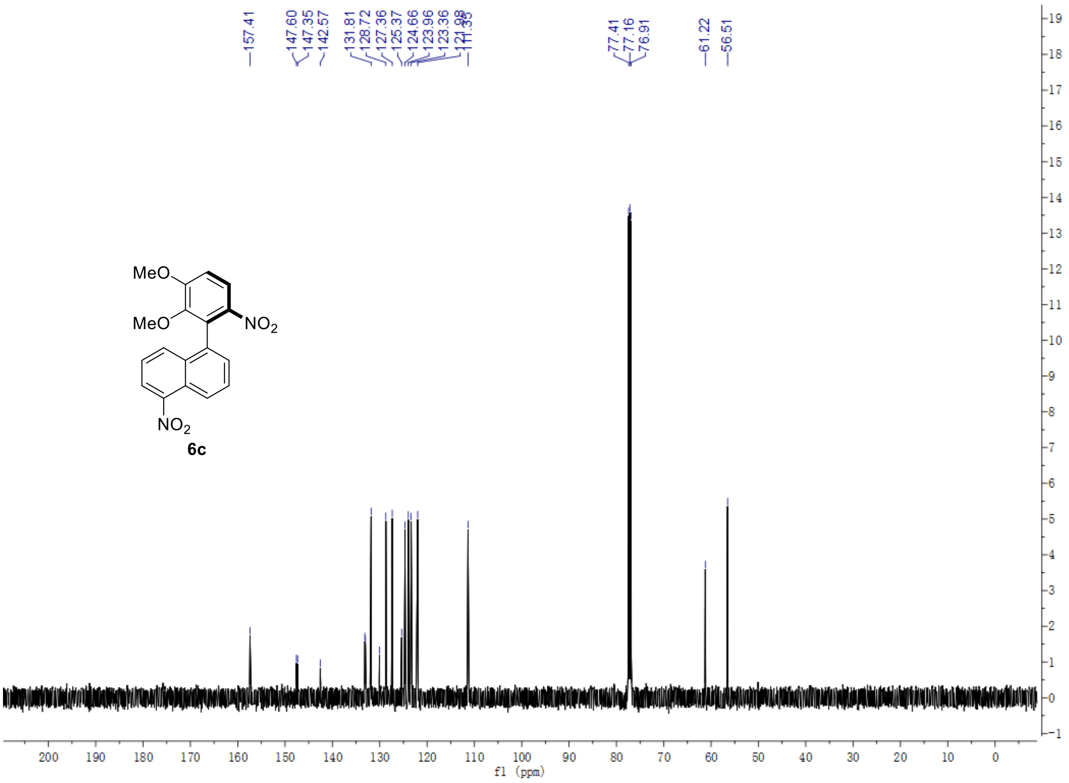

Supplementary Figure 137 ^{1}H NMR spectrum of **6d** (500 MHz, $CDCl_3$)

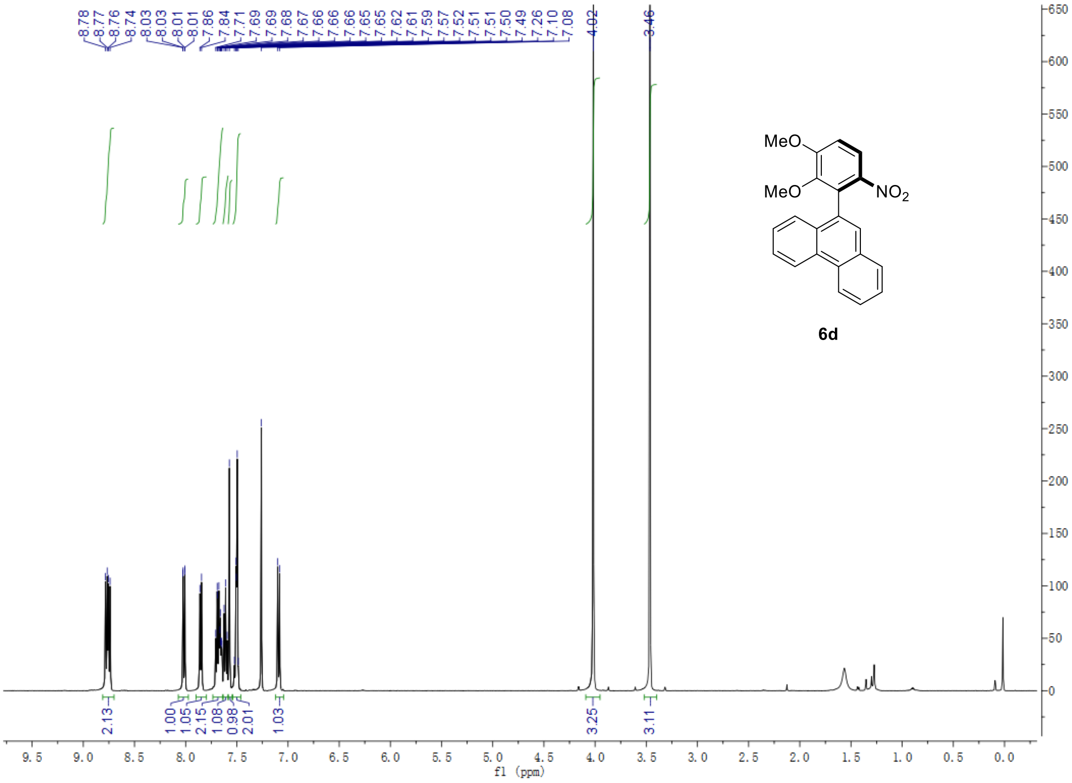

Supplementary Figure 138 ^{13}C NMR spectrum of **6d** (126 MHz, $CDCl_3$)

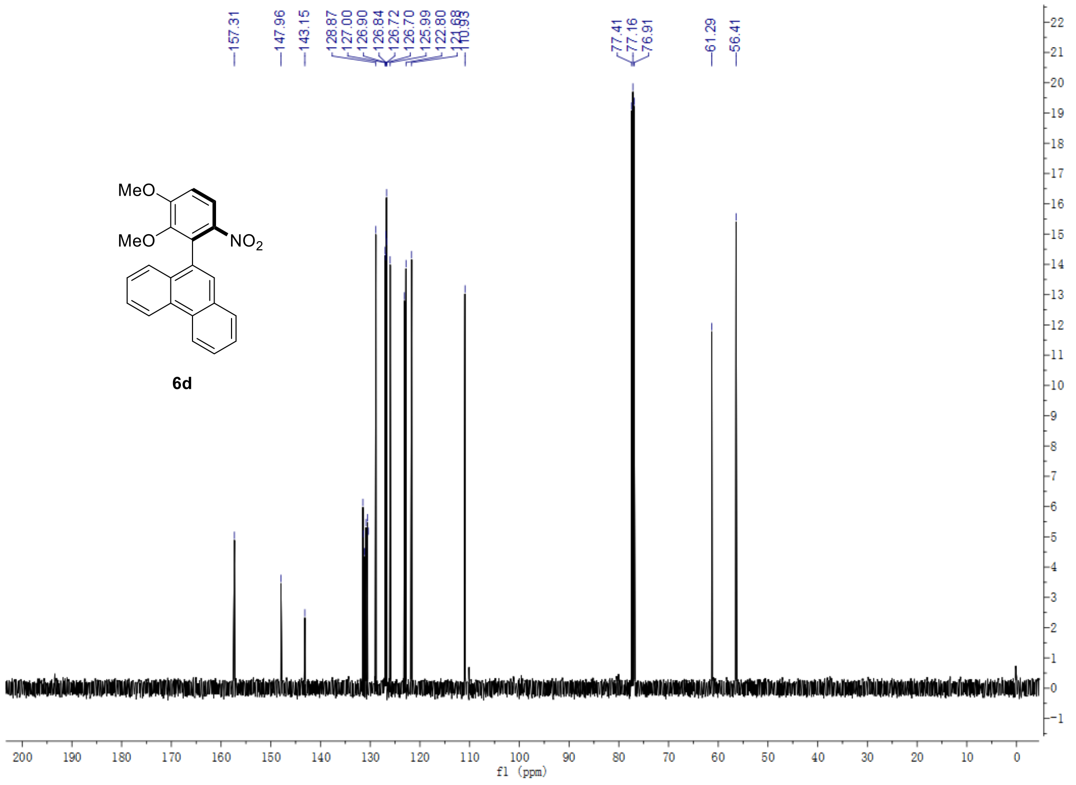

Supplementary Figure 139 ^{1}H NMR spectrum of **6e** (500 MHz, $CDCl_3$)

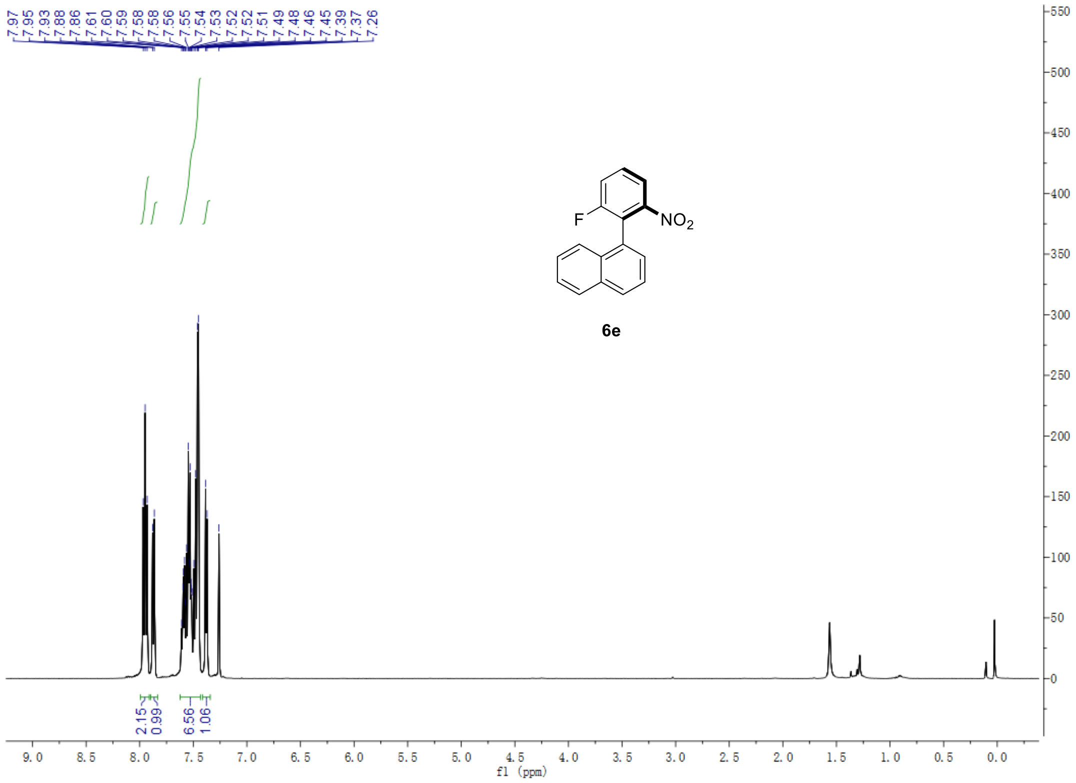

Supplementary Figure 140 ^{13}C NMR spectrum of **6e** (126 MHz, $CDCl_3$)

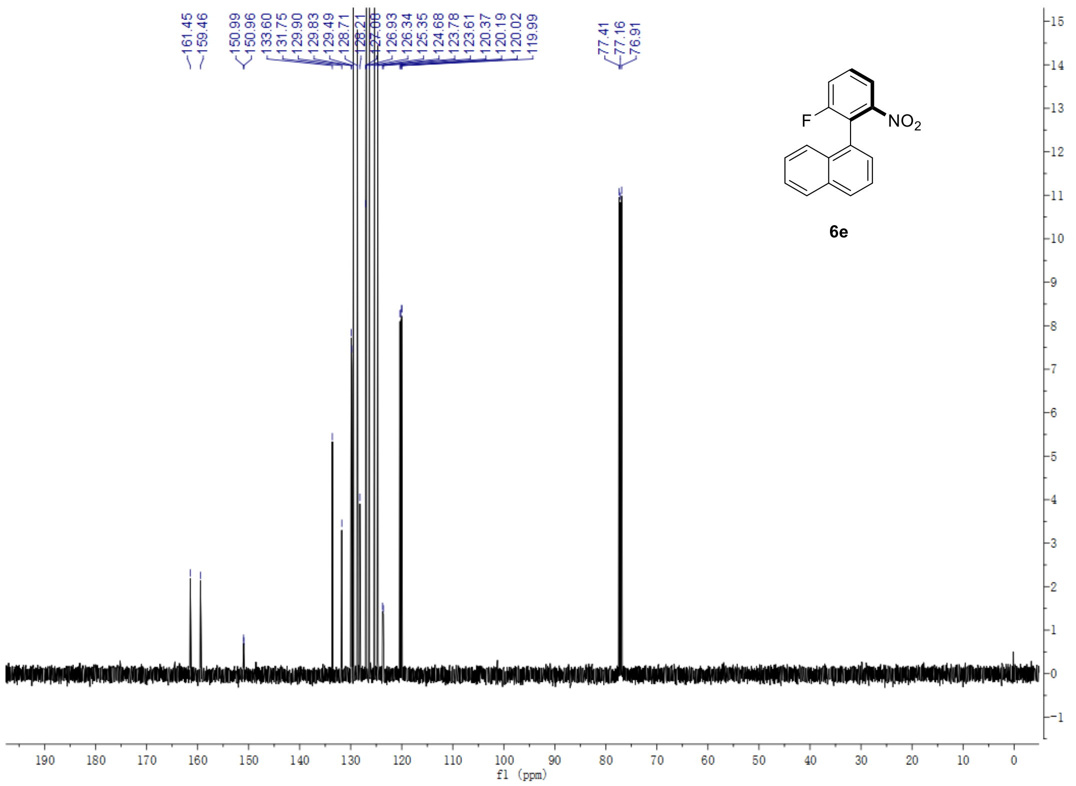

Supplementary Figure 141 ^{19}F NMR spectrum of **6e** (282 MHz, $CDCl_3$)

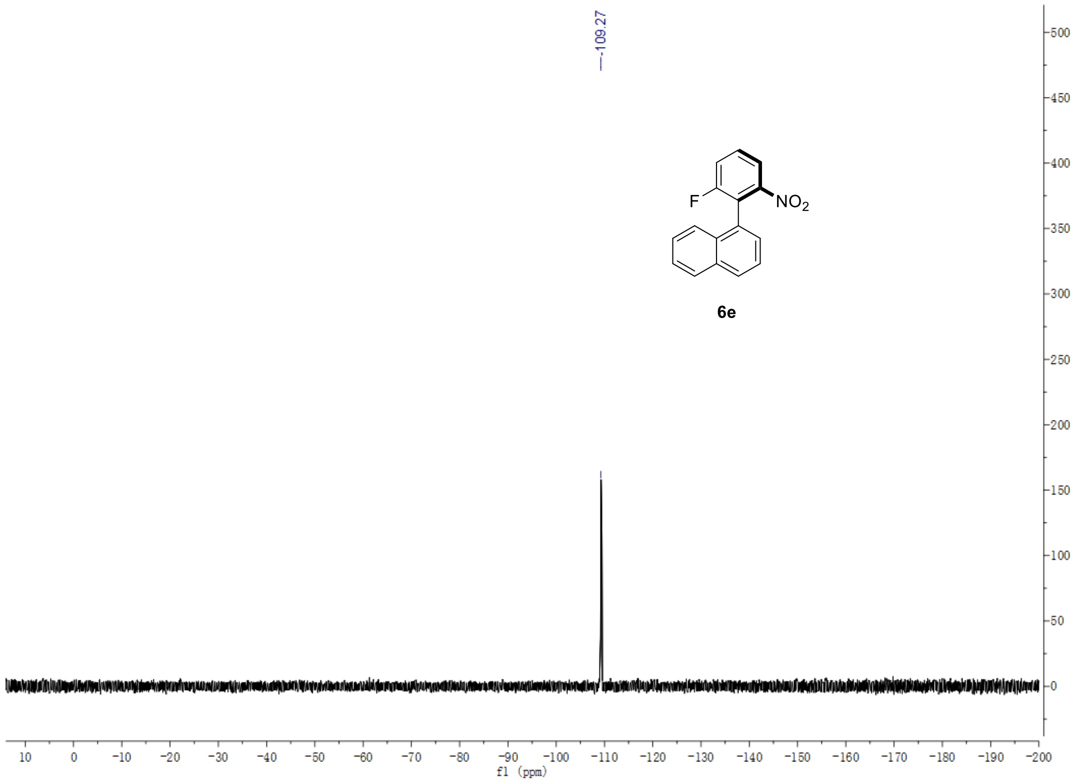

Supplementary Figure 142 ^{1}H NMR spectrum of **6f** (500 MHz, $CDCl_3$)

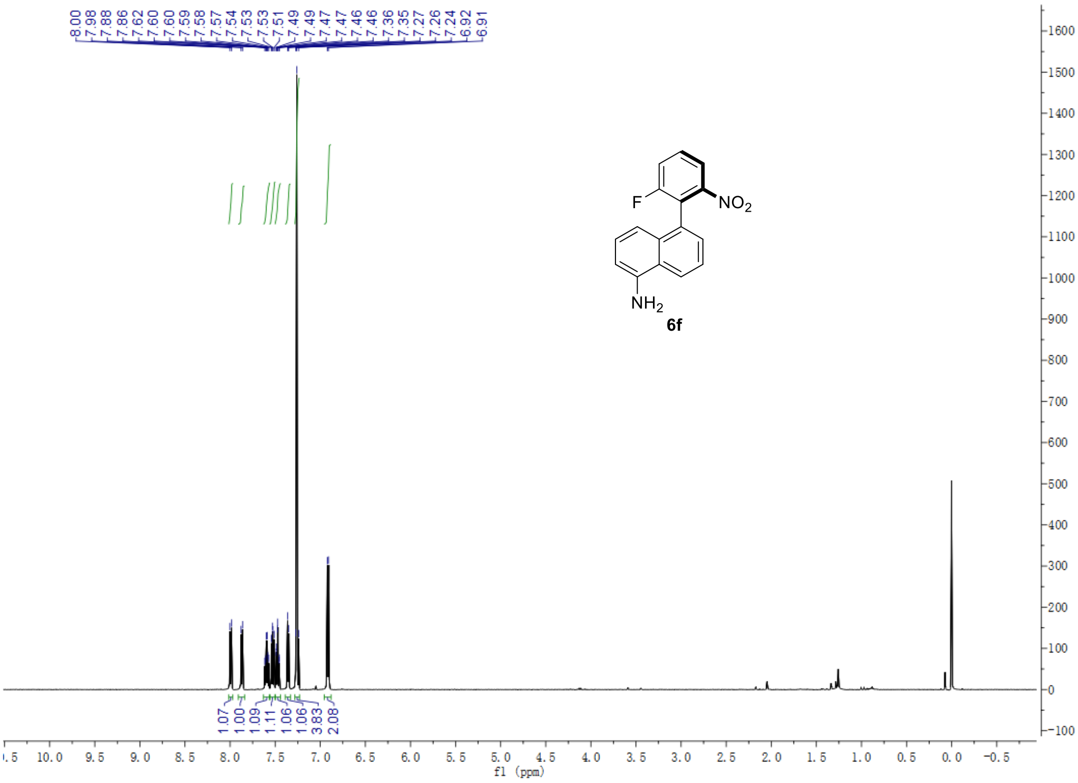

Supplementary Figure 143 ^{13}C NMR spectrum of **6f** (151 MHz, $CDCl_3$)

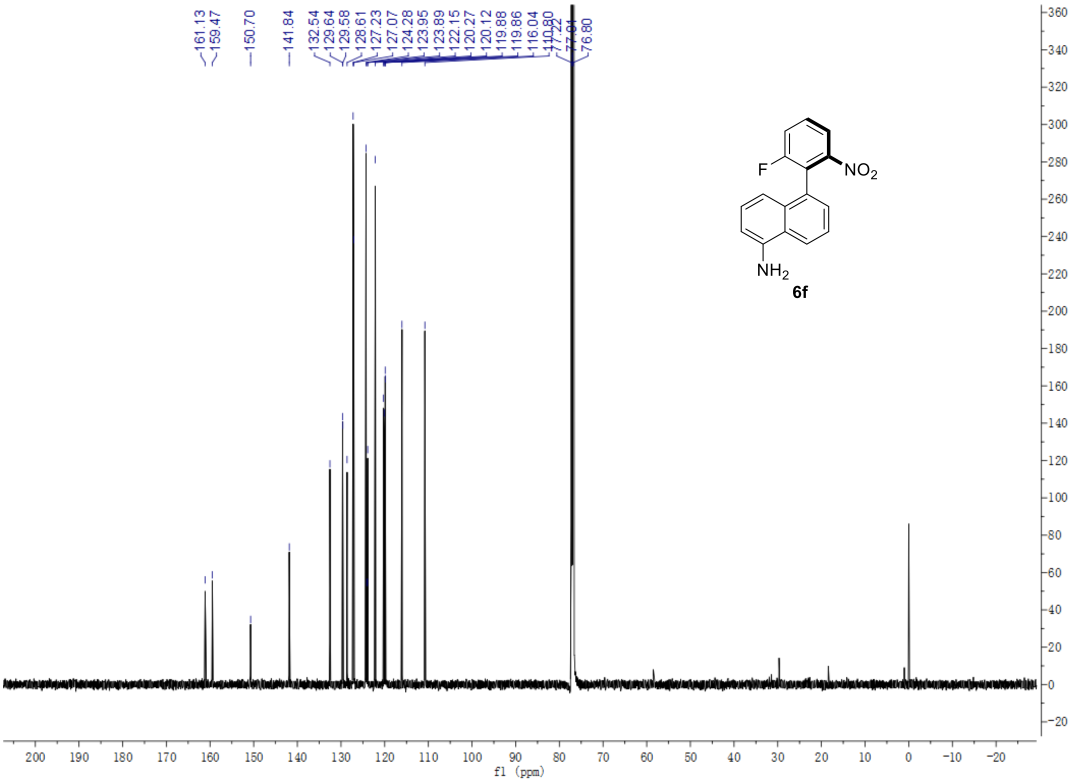

Supplementary Figure 144 ^{19}F NMR spectrum of **6f** (376 MHz, $CDCl_3$)

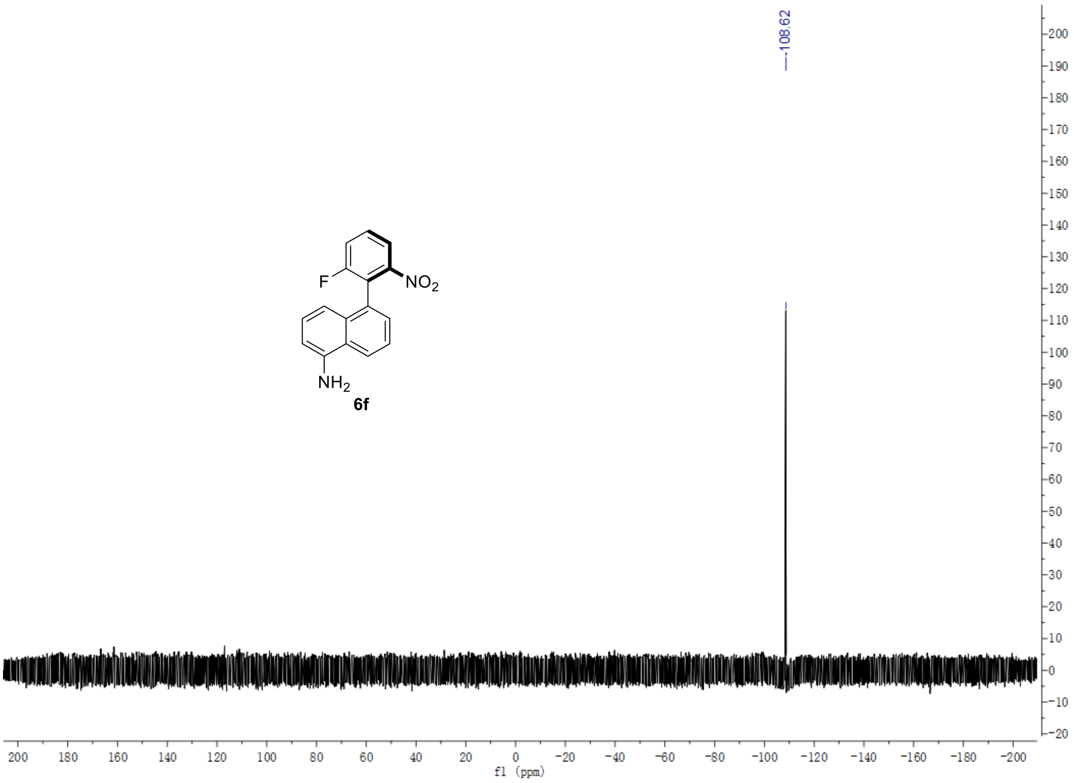

Supplementary Figure 145 ^{1}H NMR spectrum of **6g** (500 MHz, $CDCl_3$)

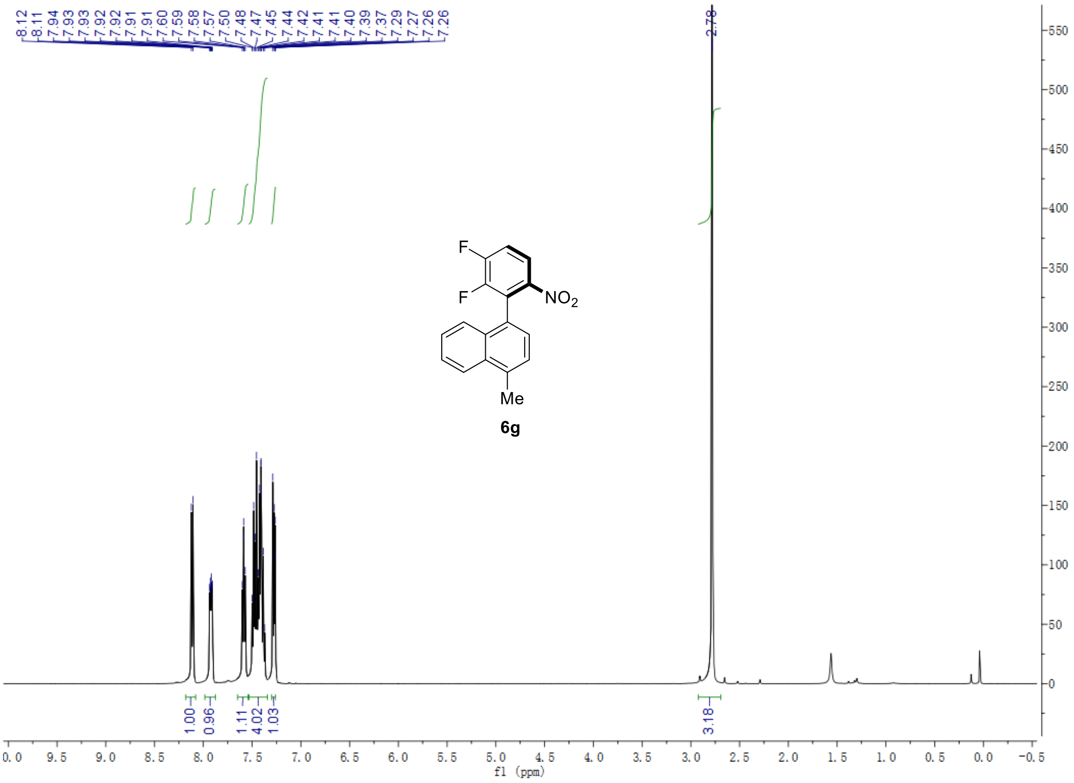

Supplementary Figure 146 ^{13}C NMR spectrum of **6g** (126 MHz, $CDCl_3$)

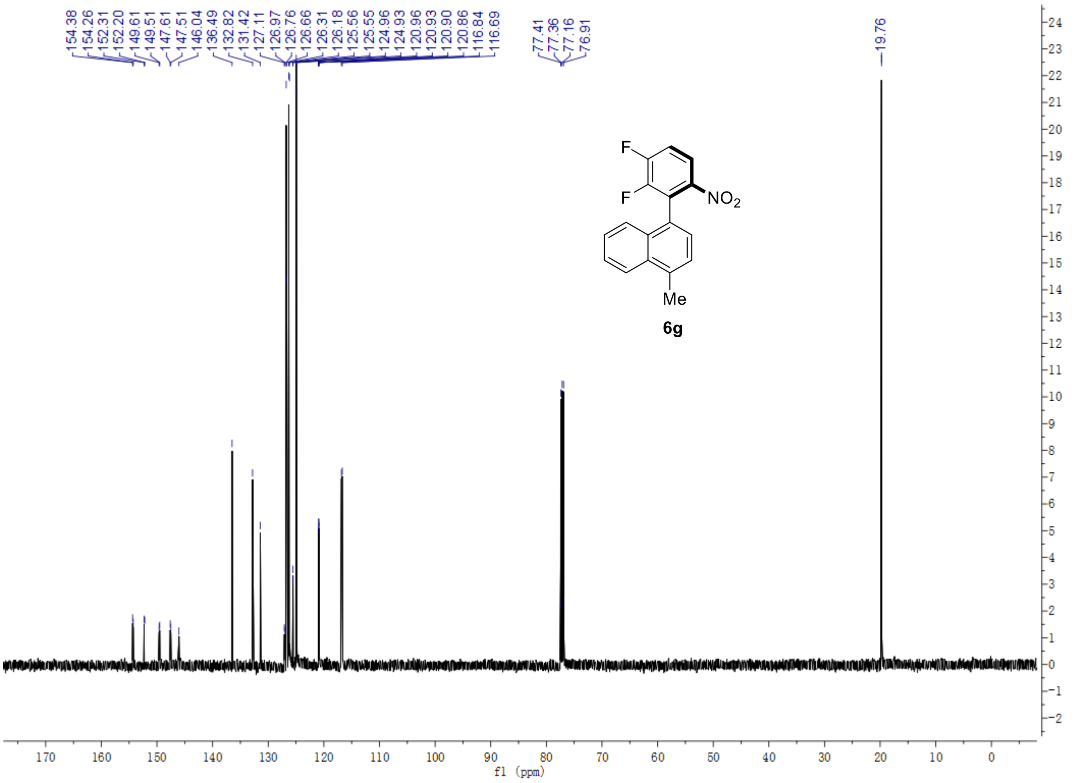

Supplementary Figure 147 ^{19}F NMR spectrum of **6g** (376 MHz, $CDCl_3$)

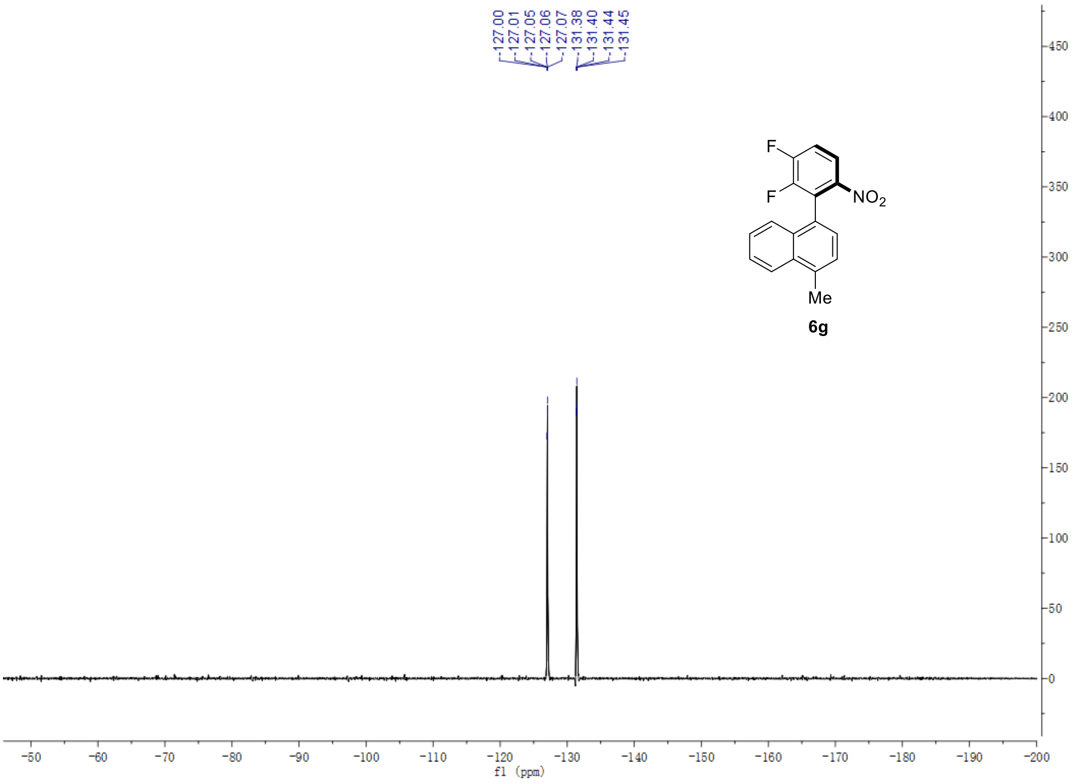

Supplementary Figure 148 ^{1}H NMR spectrum of **6h** (500 MHz, $CDCl_3$)

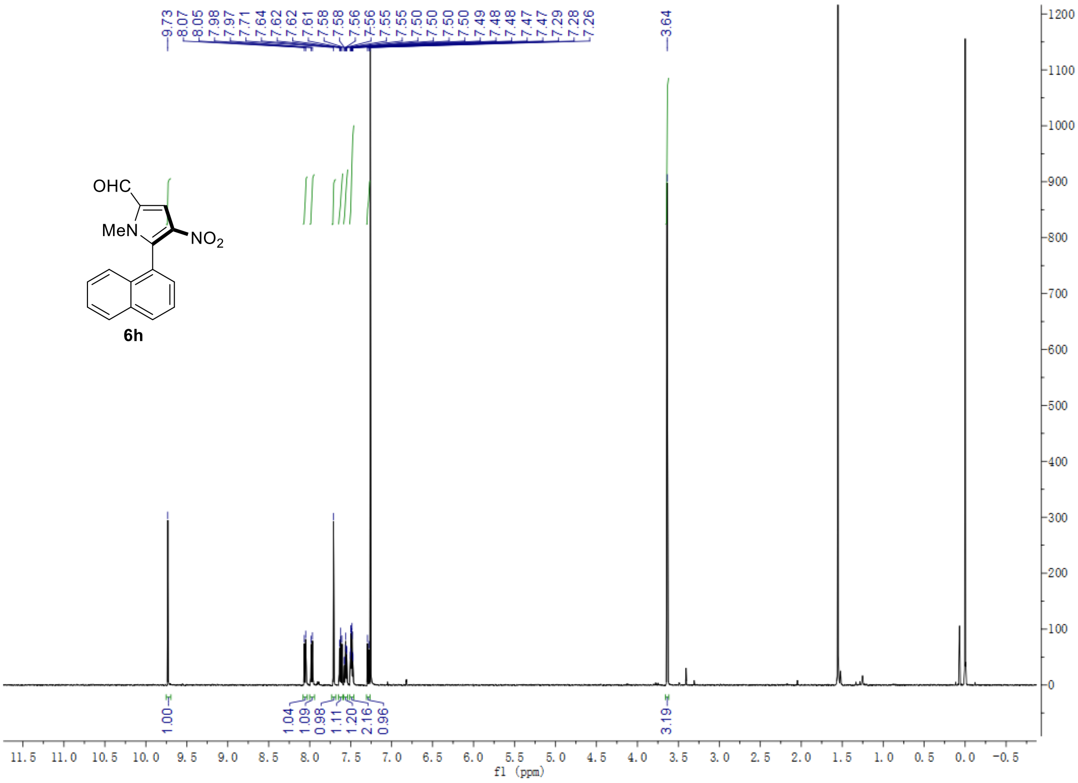

Supplementary Figure 149 ^{13}C NMR spectrum of **6h** (151 MHz, $CDCl_3$)

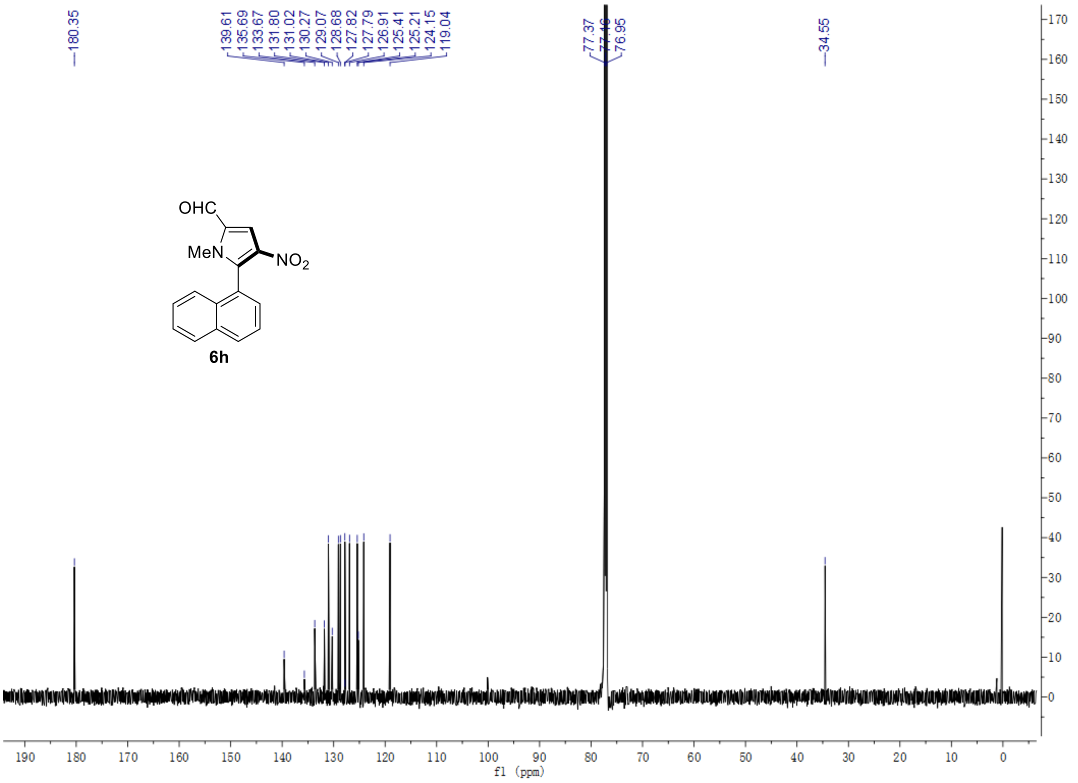

Supplementary Figure 150 ^{1}H NMR spectrum of **6i** (400 MHz, $CDCl_3$)

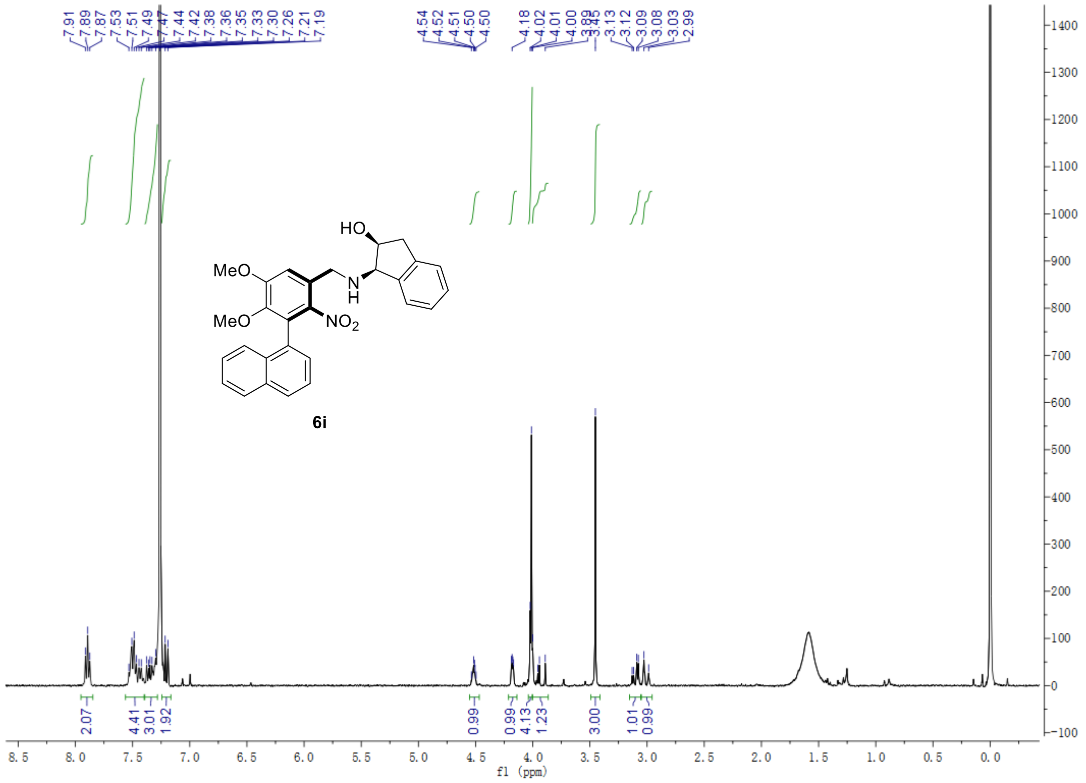

Supplementary Figure 151 ^{13}C NMR spectrum of **6i** (151 MHz, $CDCl_3$)

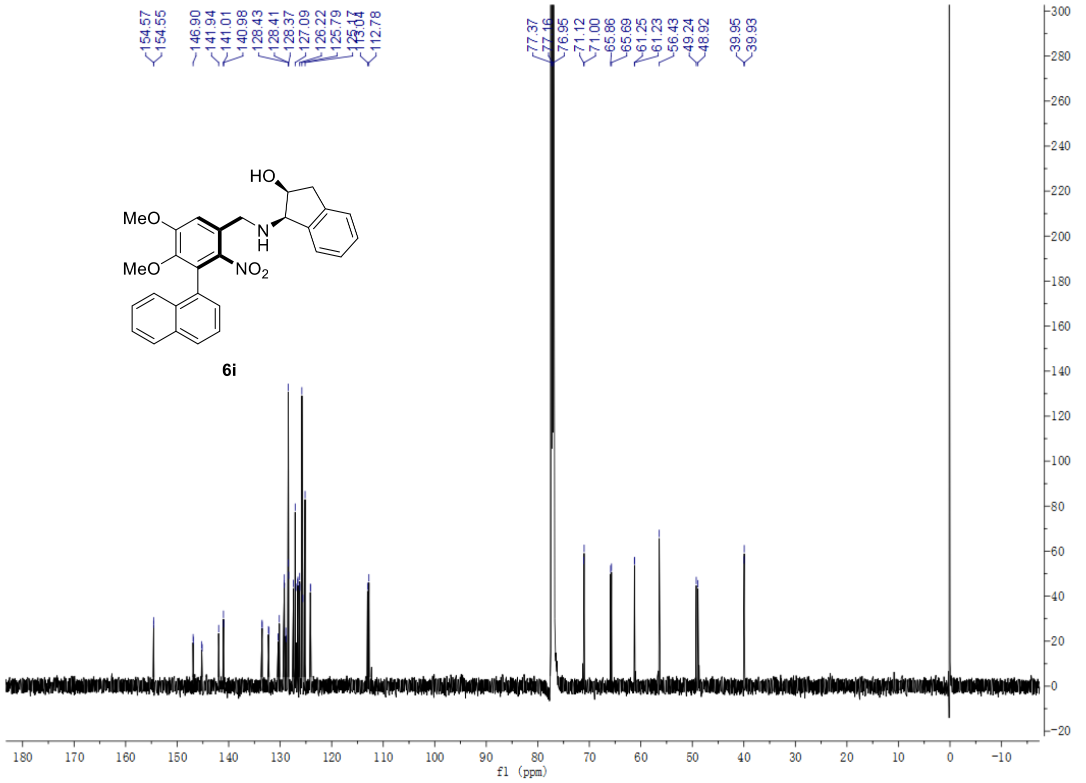

Supplementary Figure 152 ^{1}H NMR spectrum of **6j** (400 MHz, $CDCl_3$)

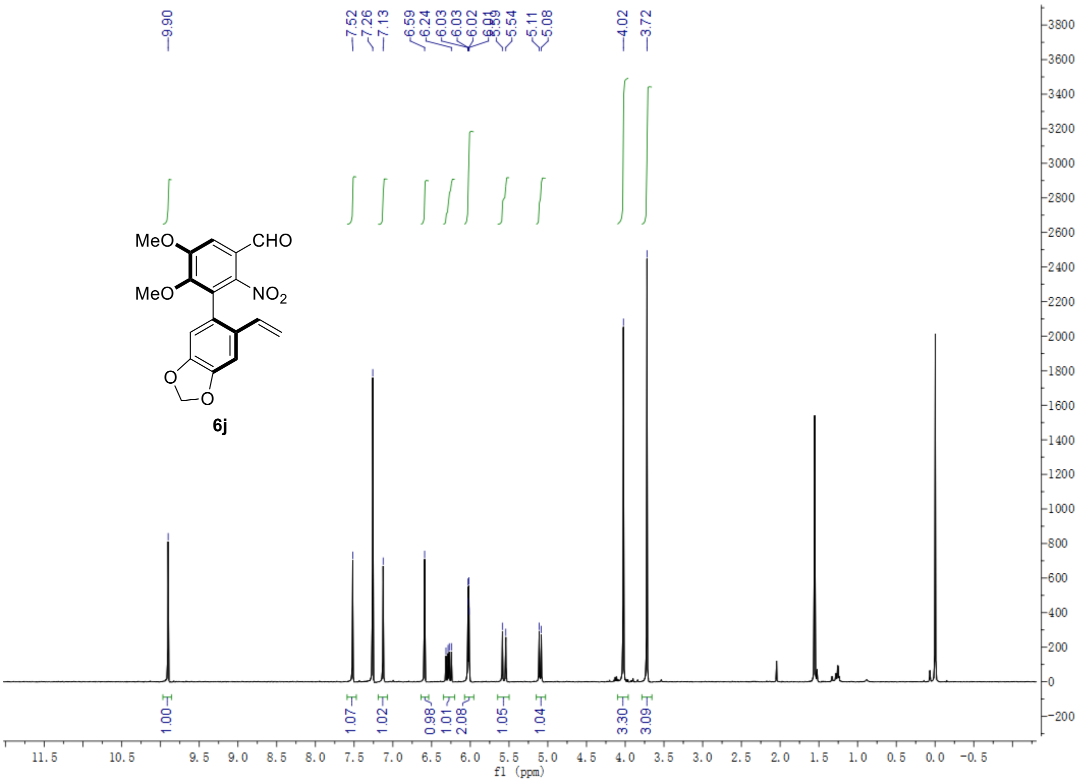

Supplementary Figure 153 ^{13}C NMR spectrum of **6j** (151 MHz, $CDCl_3$)

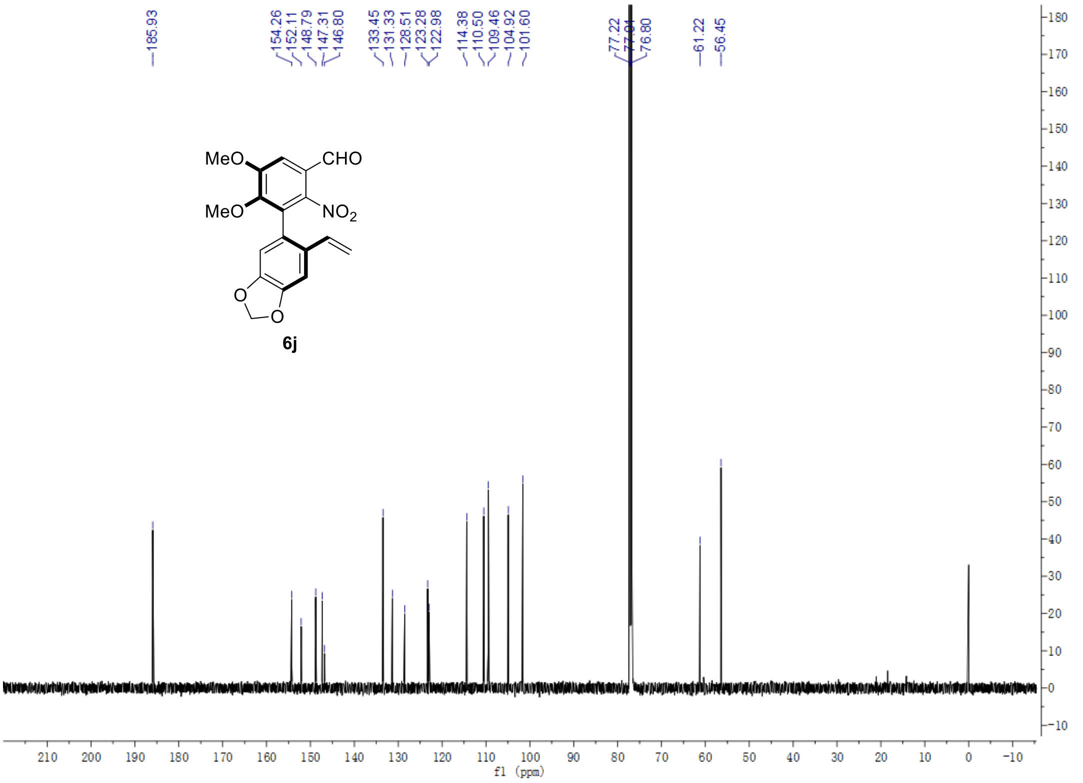

Supplementary Figure 154 ^{1}H NMR spectrum of **6k** (500 MHz, $CDCl_3$)

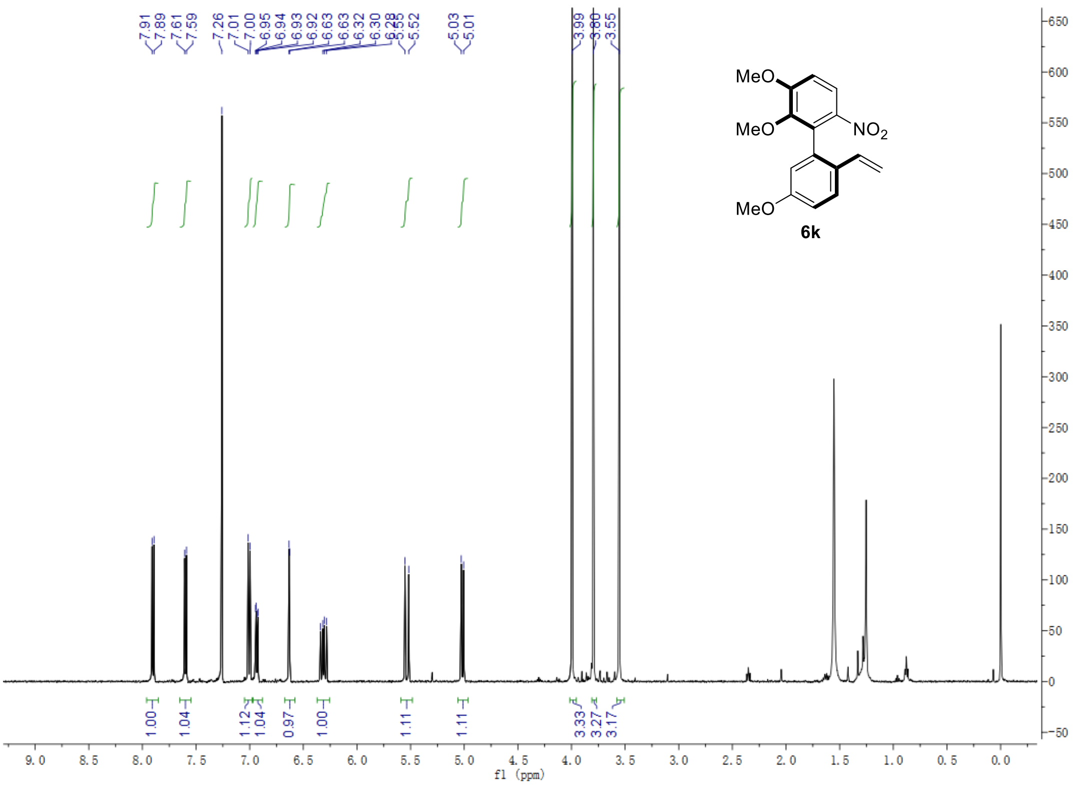

Supplementary Figure 155 ^{13}C NMR spectrum of **6k** (126 MHz, $CDCl_3$)

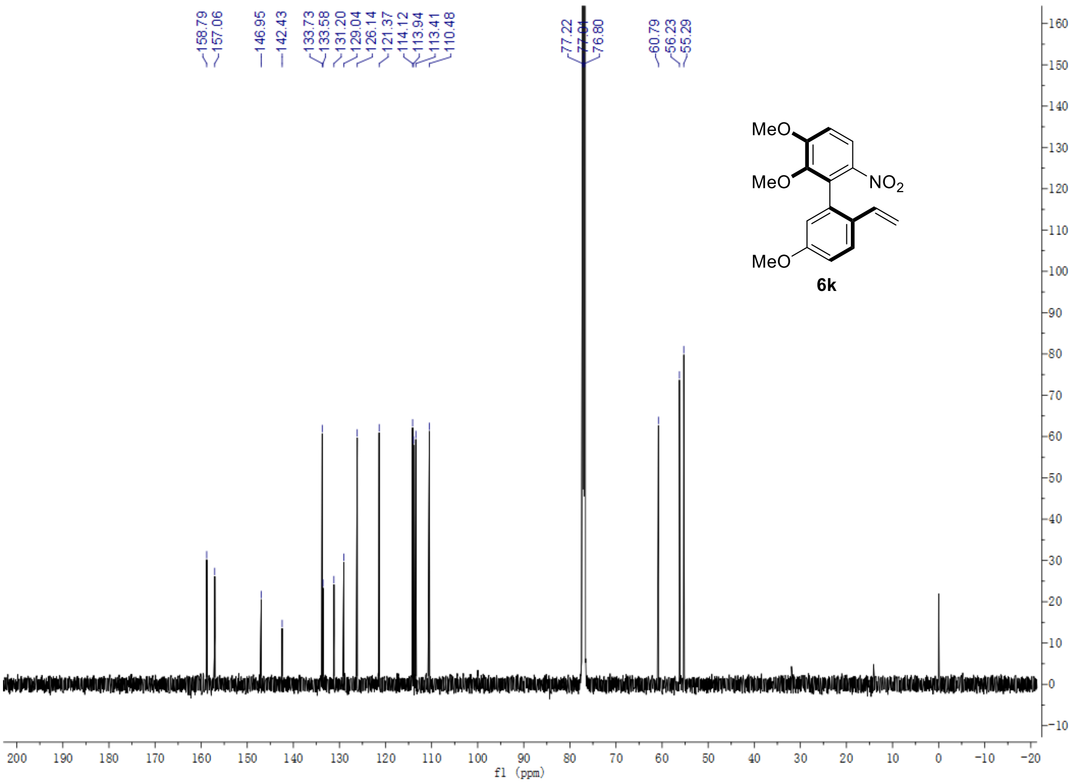

Supplementary Figure 156 ^{1}H NMR spectrum of **6l** (500 MHz, $CDCl_3$)

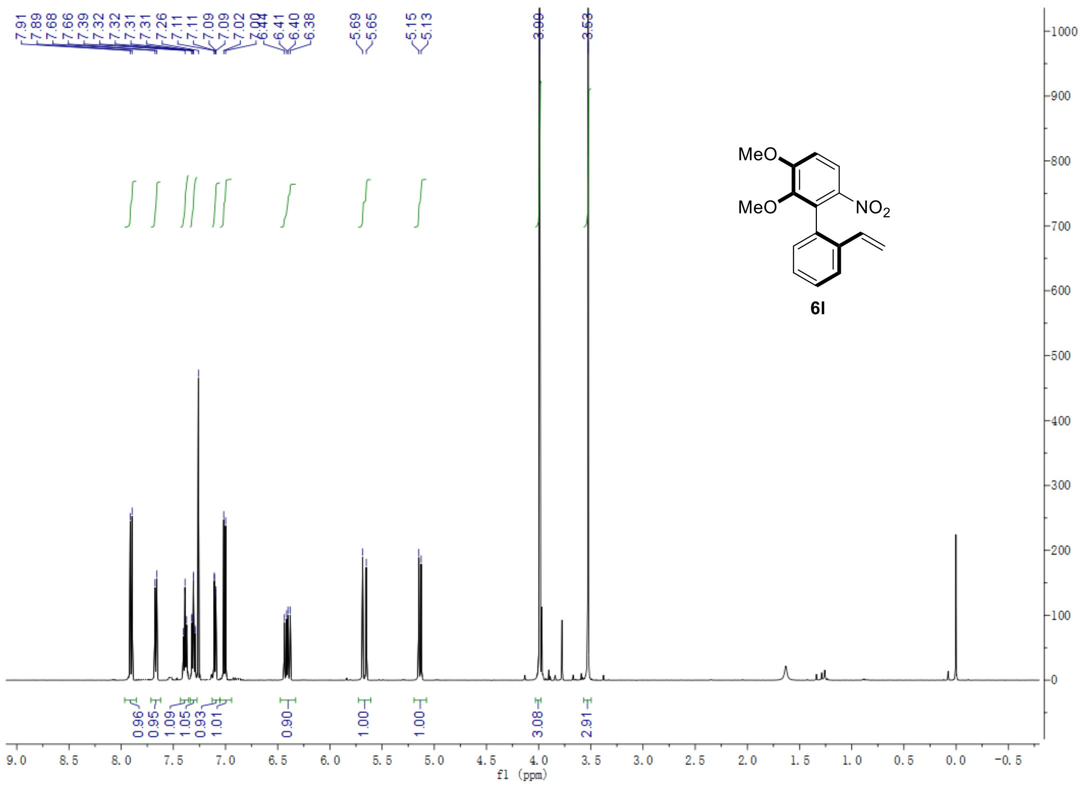

Supplementary Figure 157 ^{13}C NMR spectrum of **6l** (126 MHz, $CDCl_3$)

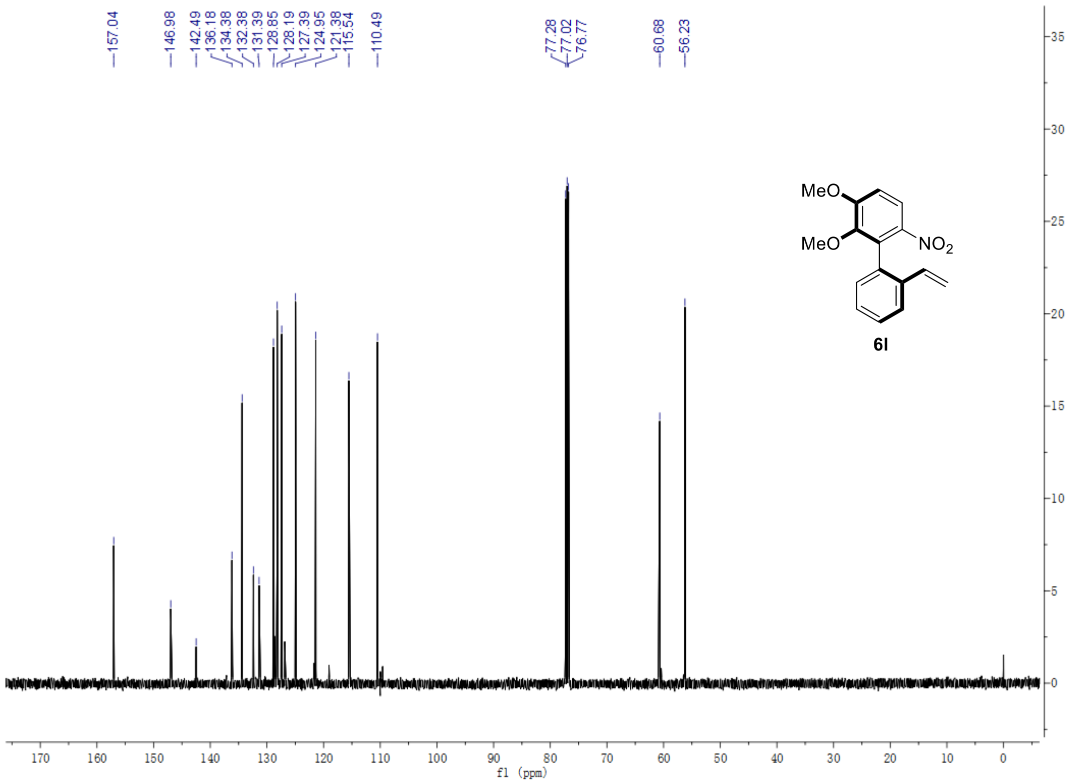

Supplementary Figure 158 ^{1}H NMR spectrum of **6m** (400 MHz, $CDCl_3$)

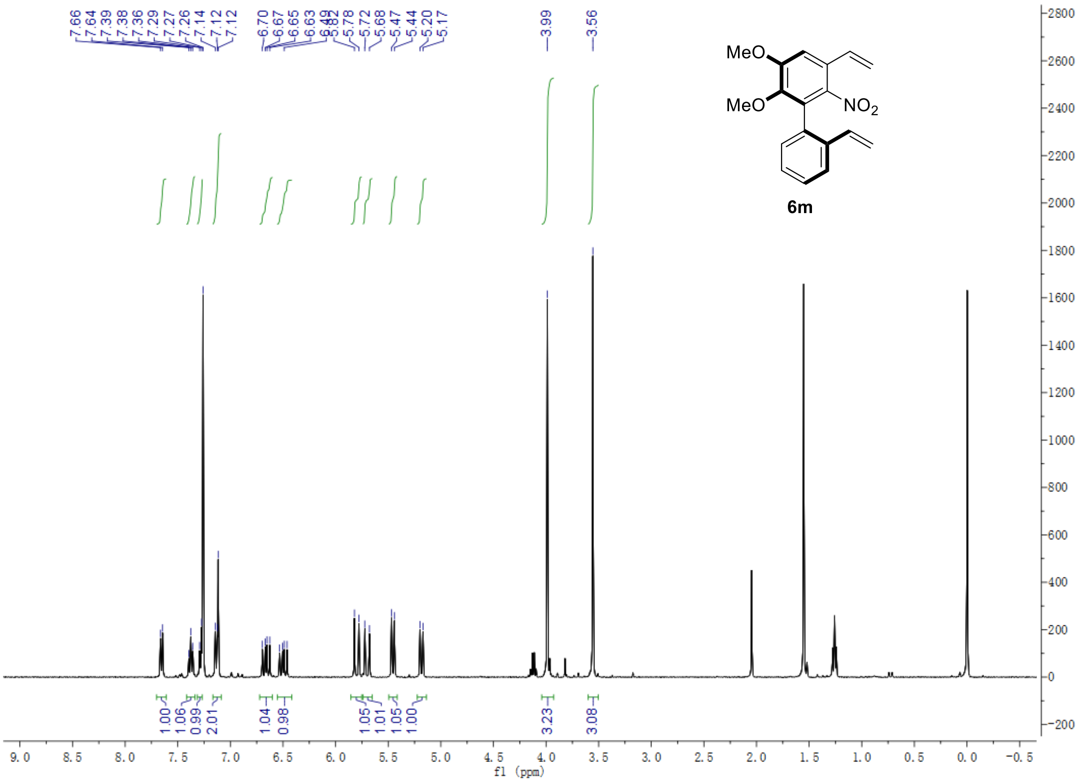

Supplementary Figure 159 ^{13}C NMR spectrum of **6m** (151 MHz, $CDCl_3$)

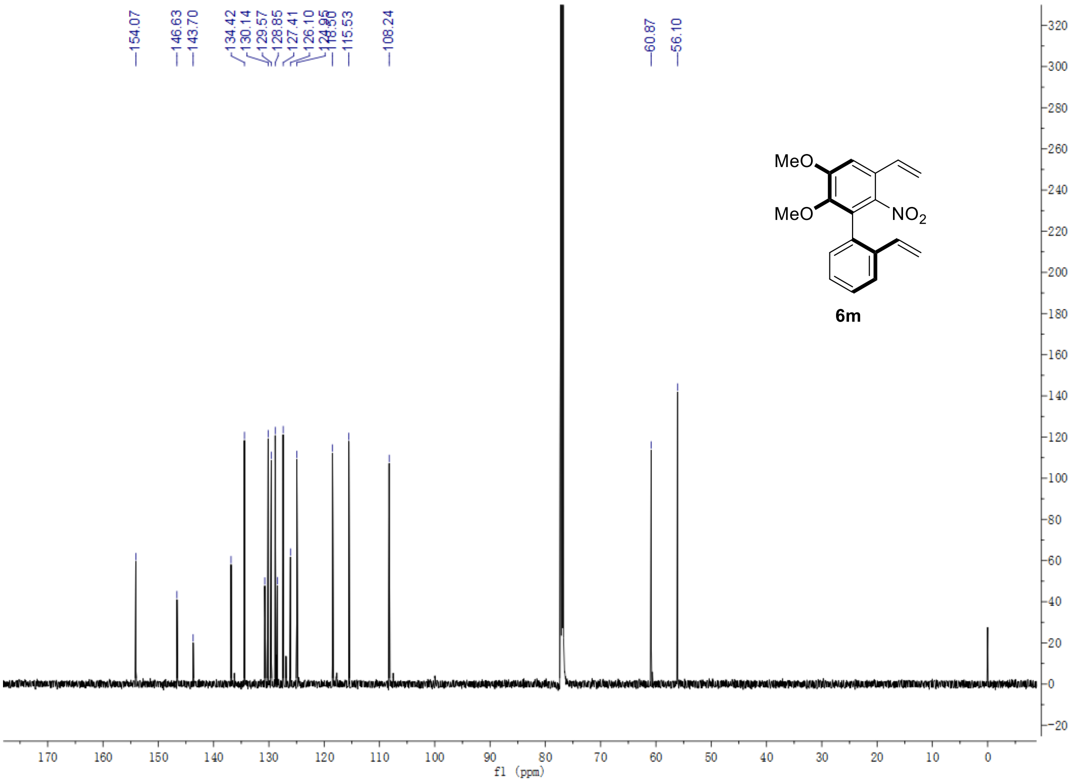

Supplementary Figure 160 ^{1}H NMR spectrum of **6n** (500 MHz, $CDCl_3$)

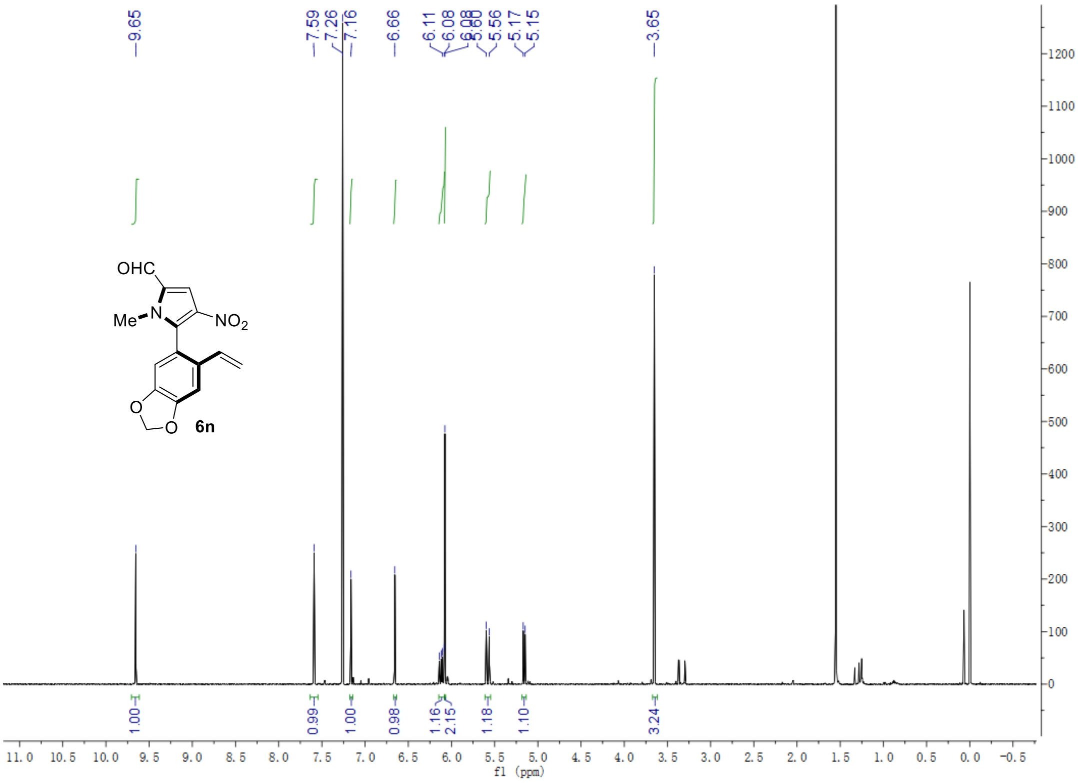

Supplementary Figure 161 ^{13}C NMR spectrum of **6n** (151 MHz, $CDCl_3$)

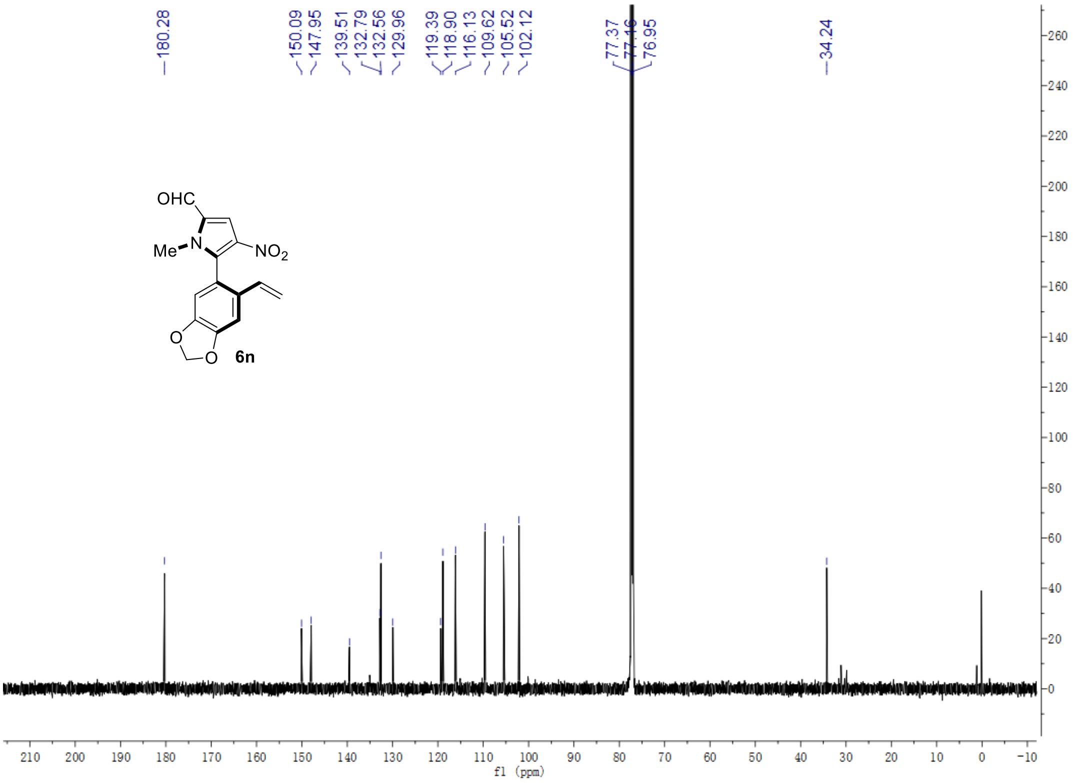

Supplementary Figure 162 ^{1}H NMR spectrum of **6o** (500 MHz, $CDCl_3$)

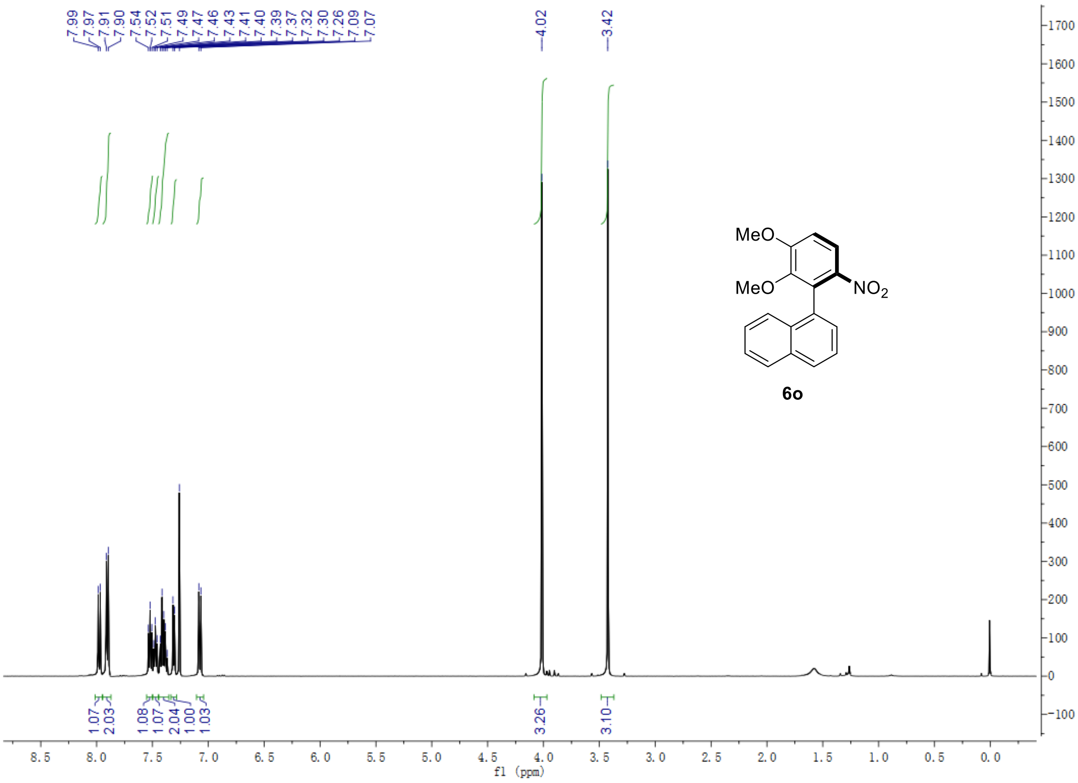

Supplementary Figure 163 ^{13}C NMR spectrum of **6o** (126 MHz, $CDCl_3$)

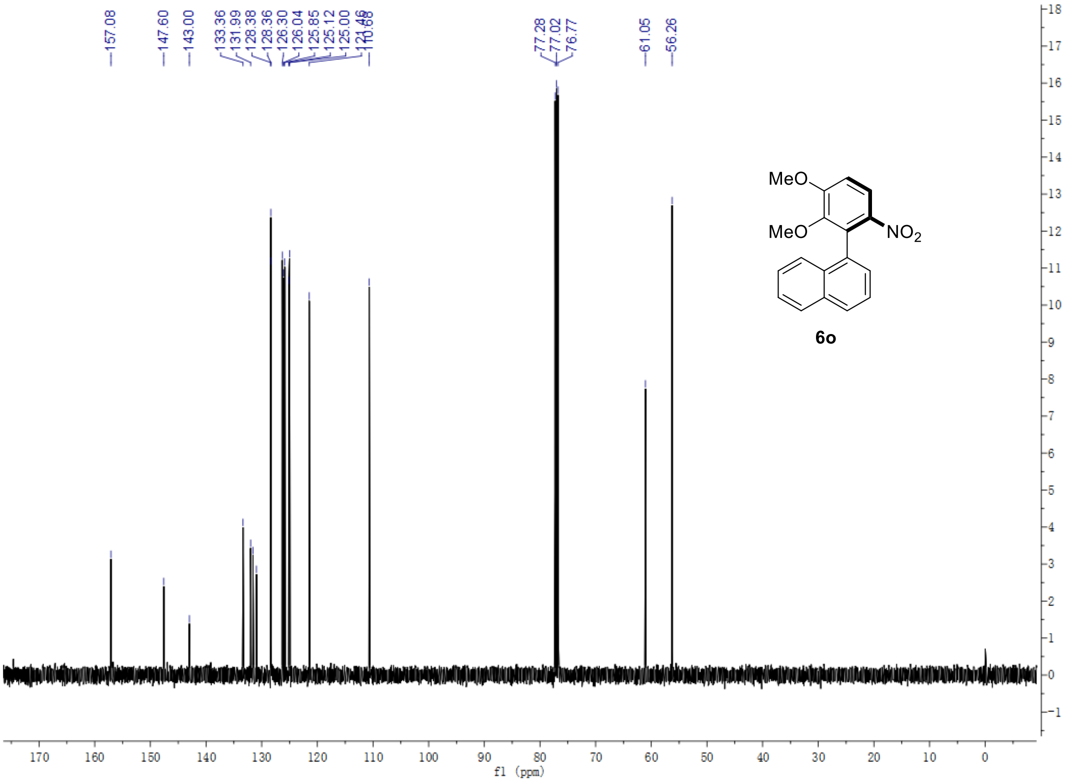

Supplementary Figure 164 ^{1}H NMR spectrum of **6p** (500 MHz, $CDCl_3$)

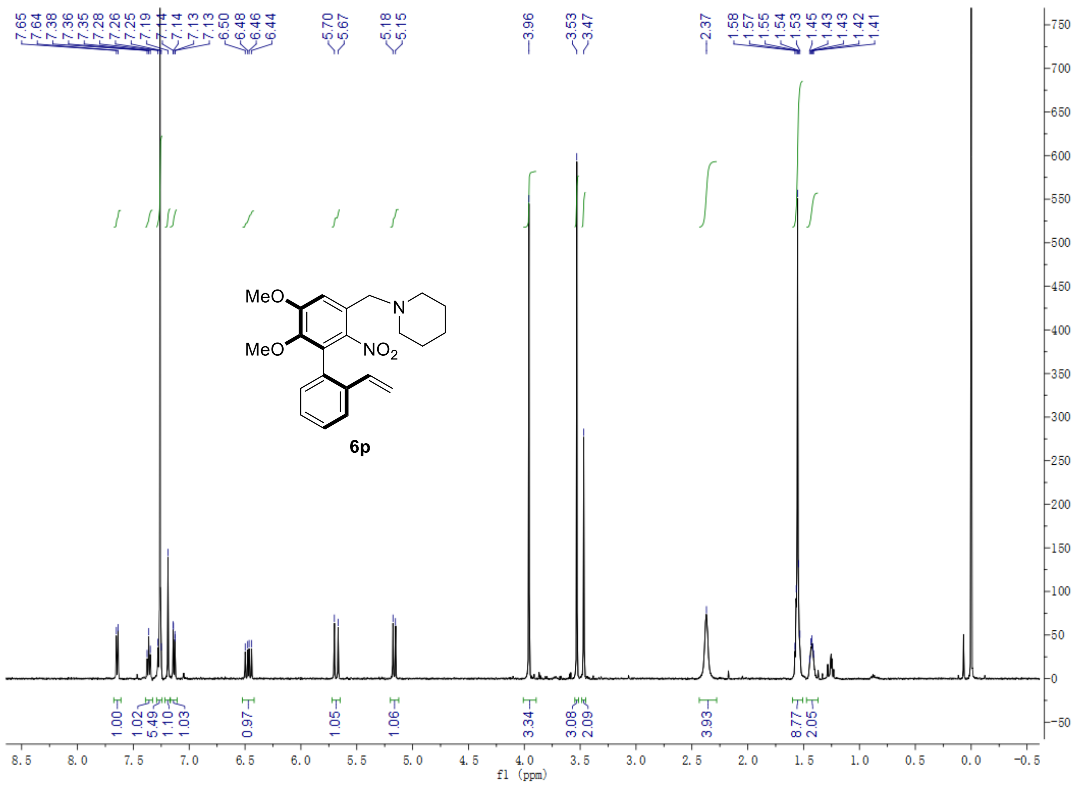

Supplementary Figure 165 ^{13}C NMR spectrum of **6p** (126 MHz, $CDCl_3$)

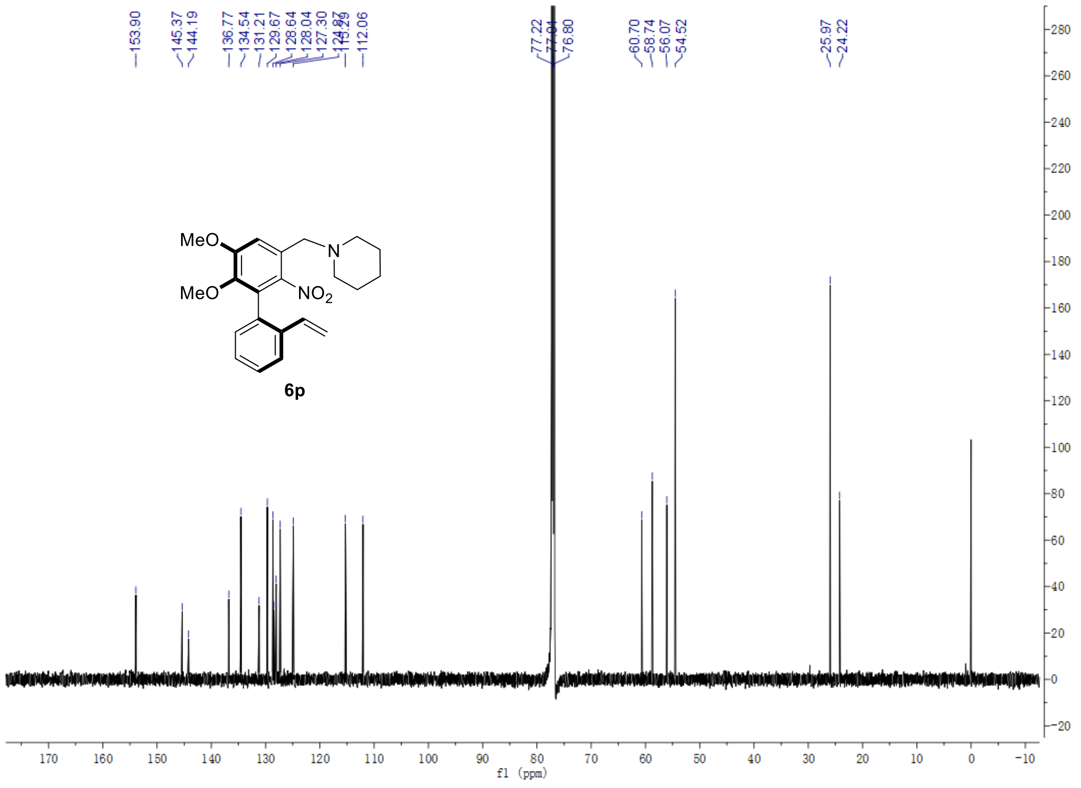

Supplementary Figure 166 ^{1}H NMR spectrum of **6q** (500 MHz, $CDCl_3$)

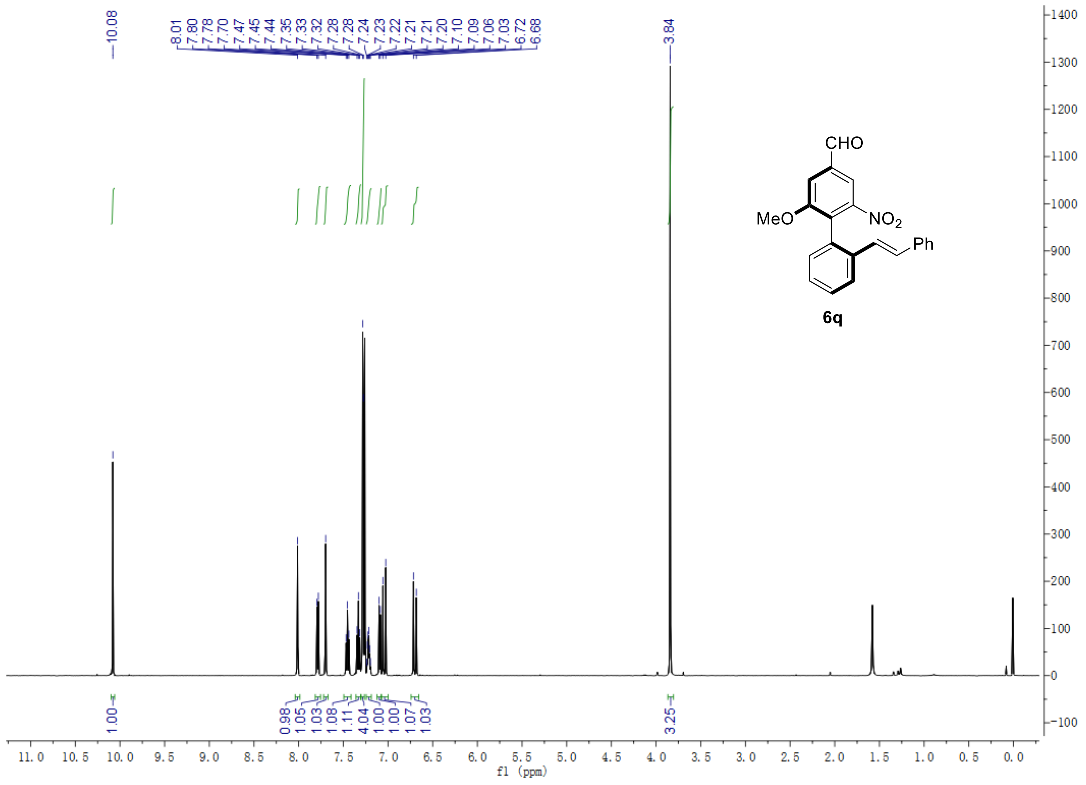

Supplementary Figure 167 ^{13}C NMR spectrum of **6q** (126 MHz, $CDCl_3$)

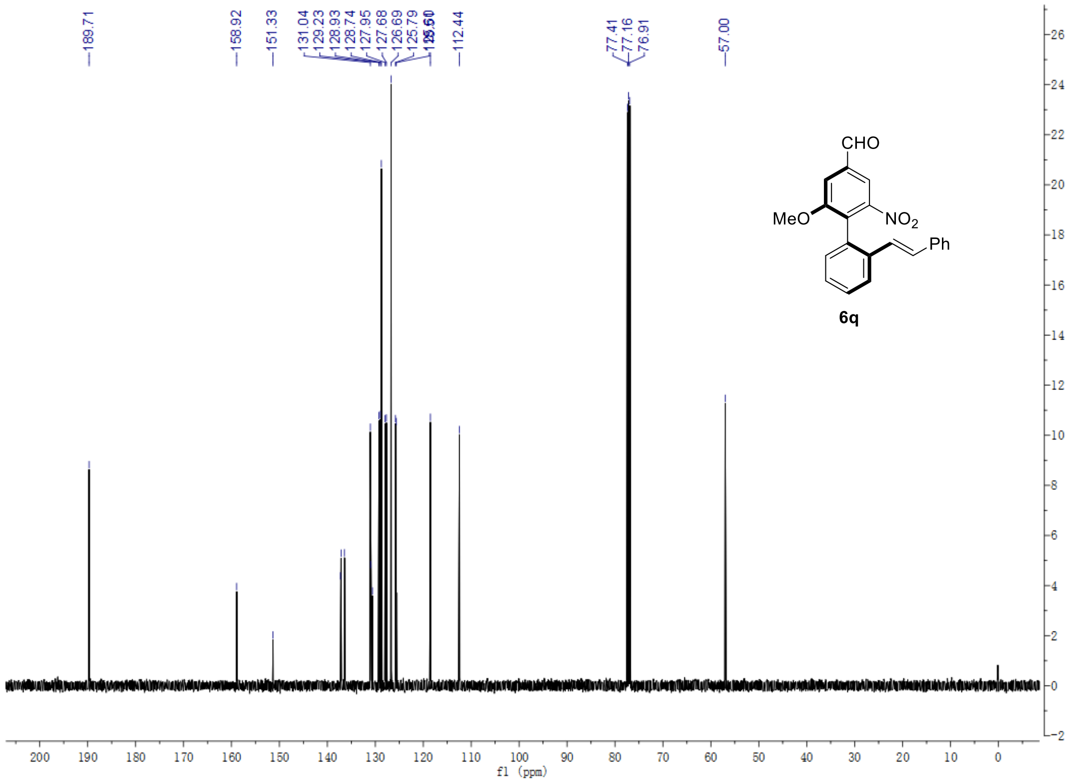

Supplementary Figure 168 ^{1}H NMR spectrum of **6r** (500 MHz, $CDCl_3$)

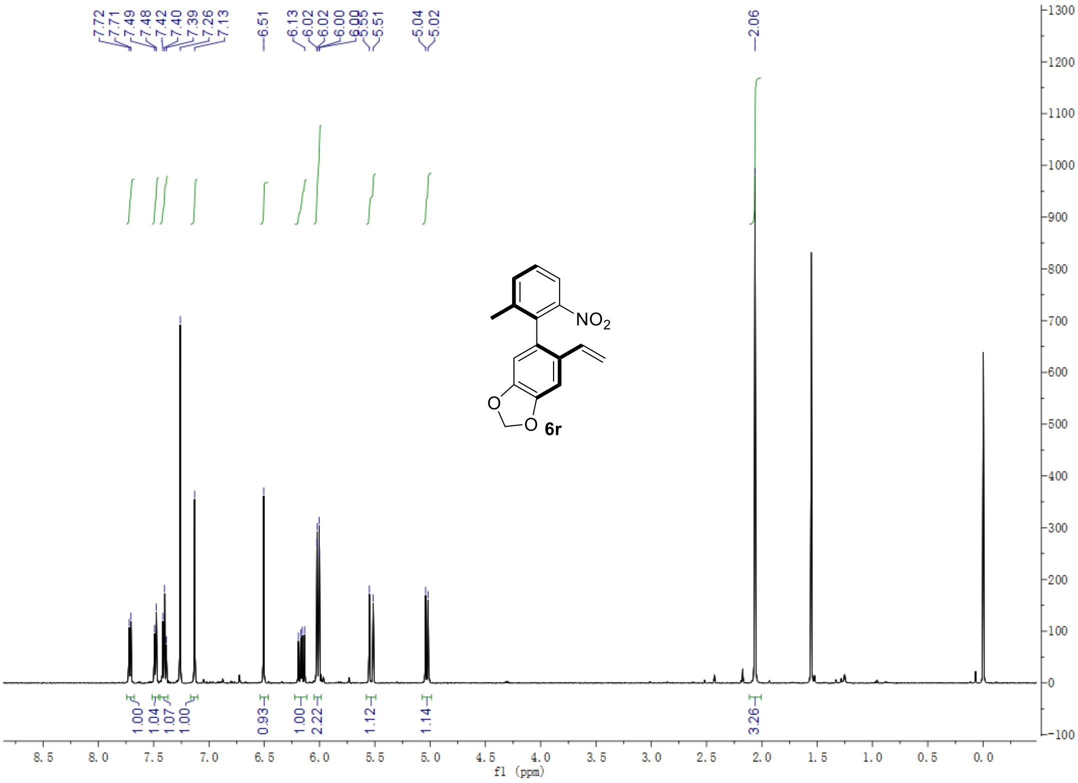

Supplementary Figure 169 ^{13}C NMR spectrum of **6r** (151 MHz, $CDCl_3$)

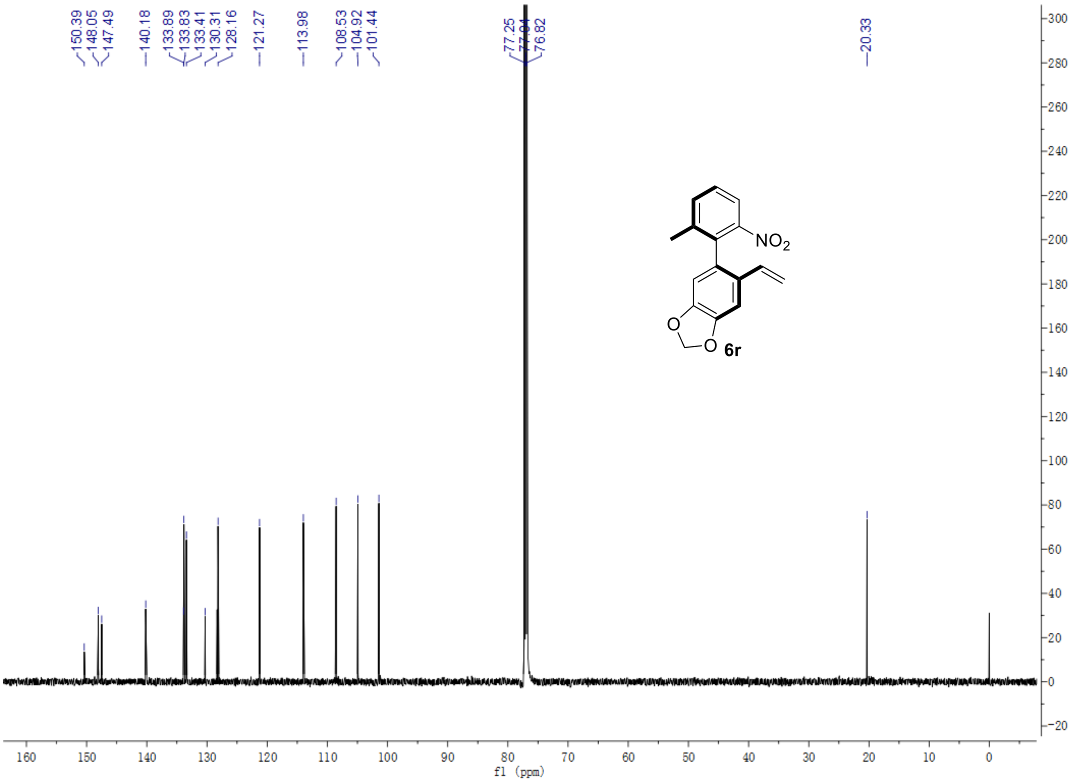

Supplementary Figure 170 ^{1}H NMR spectrum of **6s** (400 MHz, $CDCl_3$)

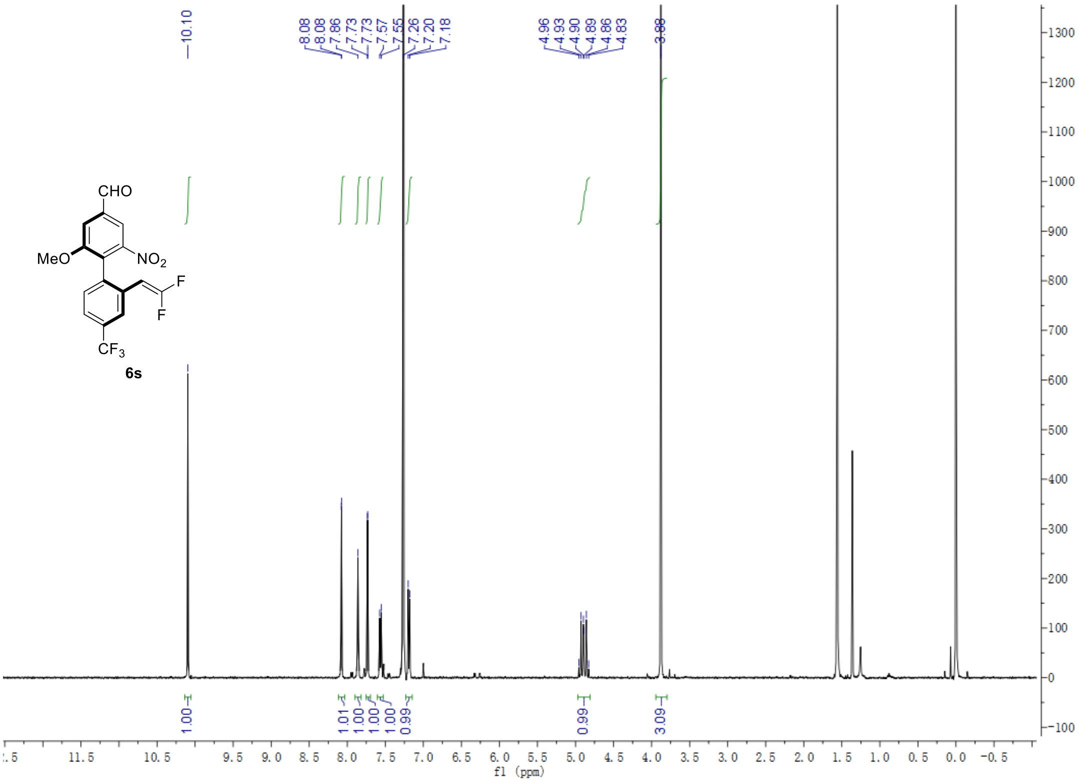

Supplementary Figure 171 ^{13}C NMR spectrum of **6s** (151 MHz, $CDCl_3$)

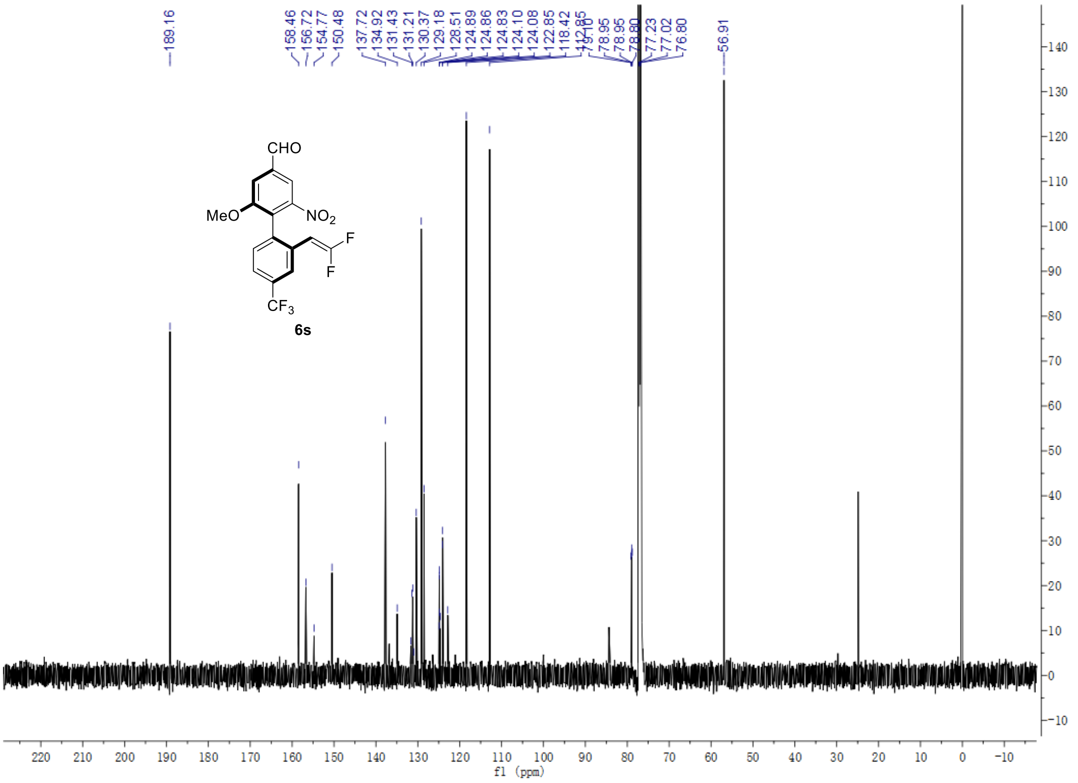

Supplementary Figure 172 ^{19}F NMR spectrum of **3s** (376 MHz, $CDCl_3$)

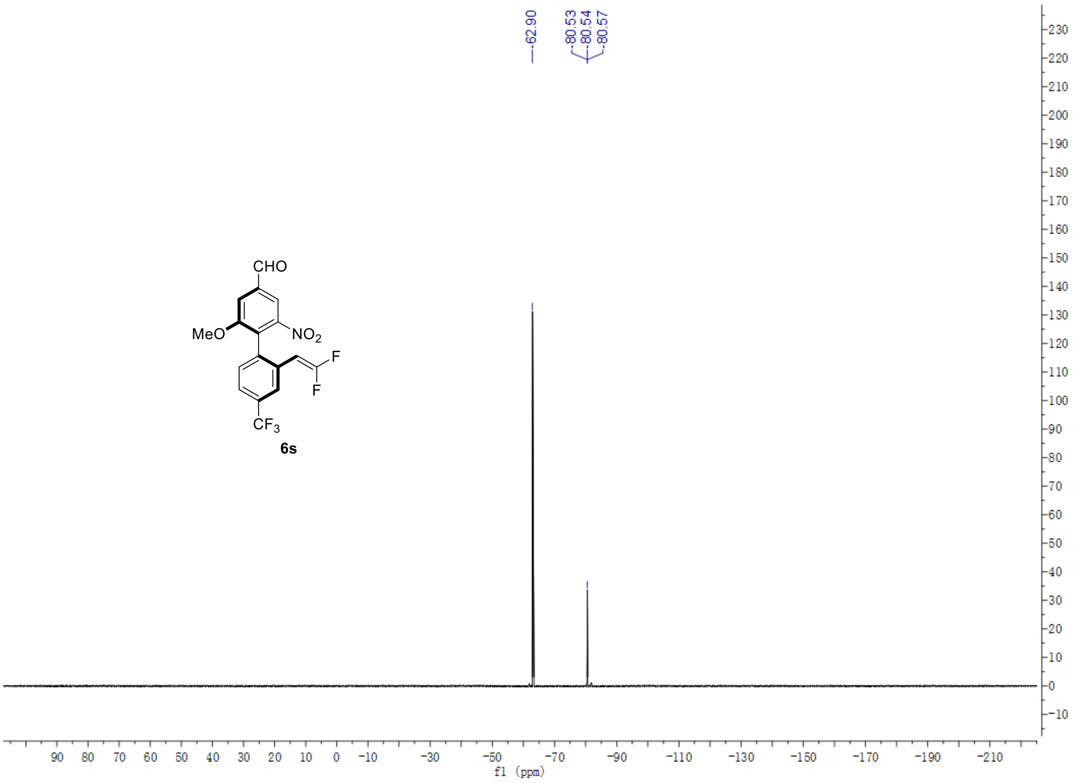

Supplementary Figure 173 ^{1}H NMR spectrum of **6t** (500 MHz, $CDCl_3$)

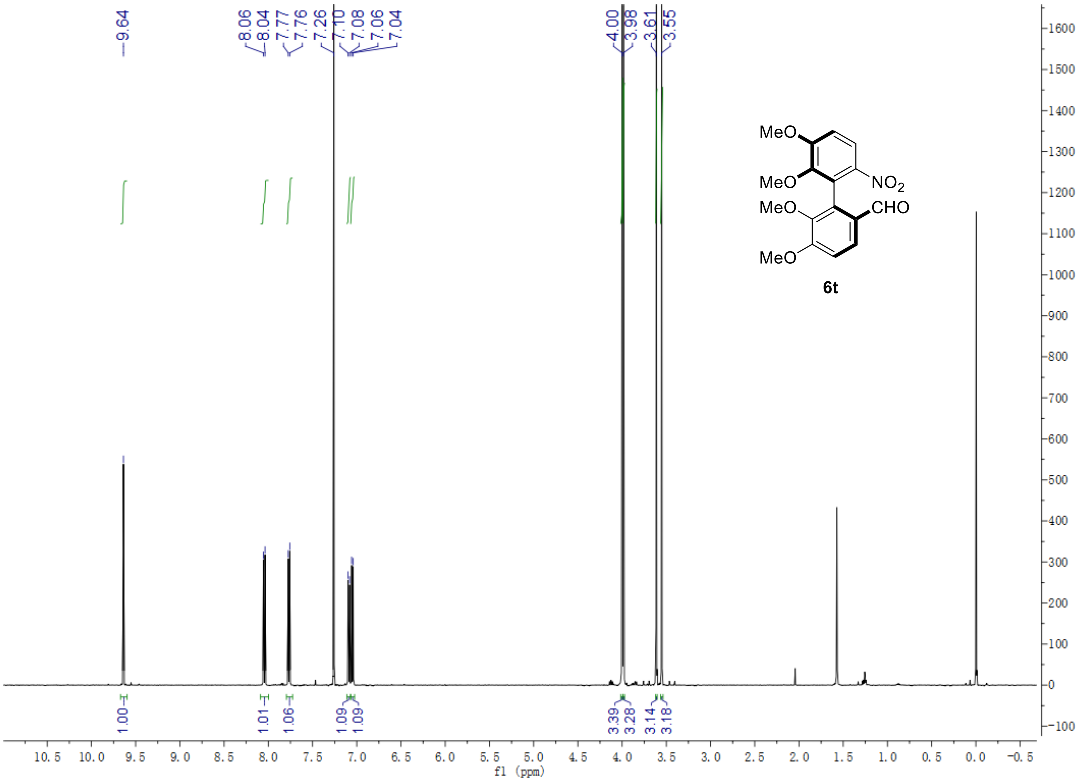

Supplementary Figure 174 ^{13}C NMR spectrum of **6t** (151 MHz, $CDCl_3$)

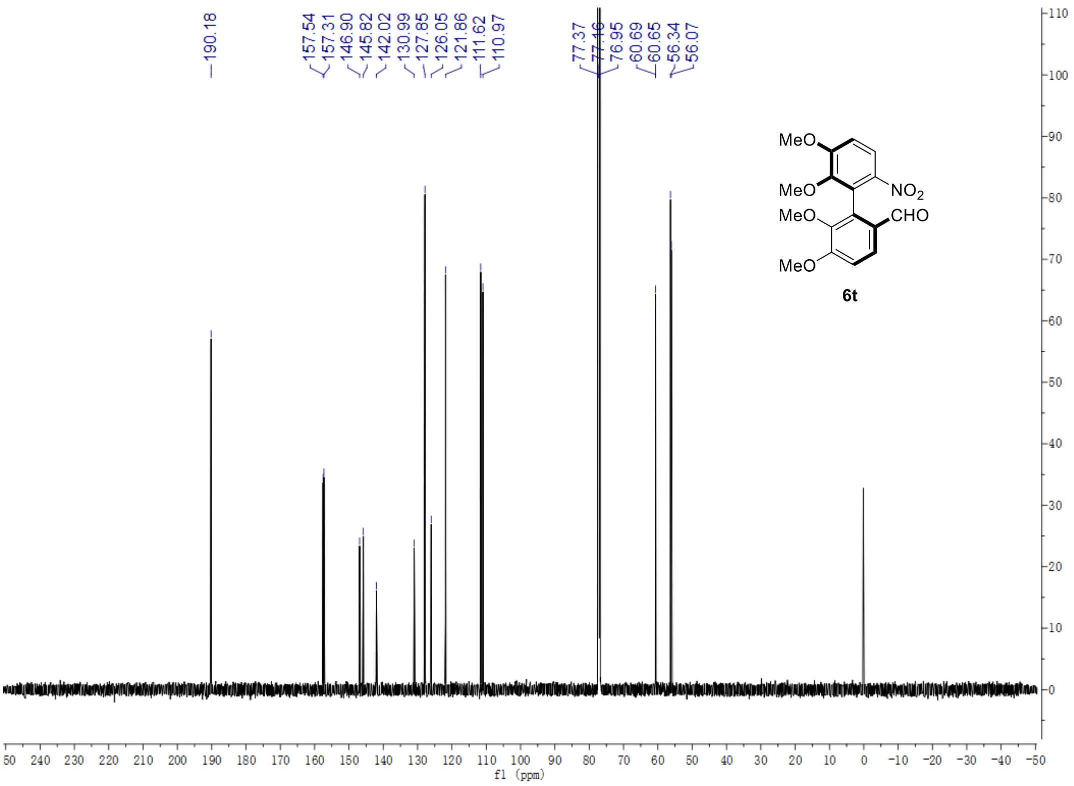

Supplementary Figure 175 ^{1}H NMR spectrum of **6u** (600 MHz, $CDCl_3$)

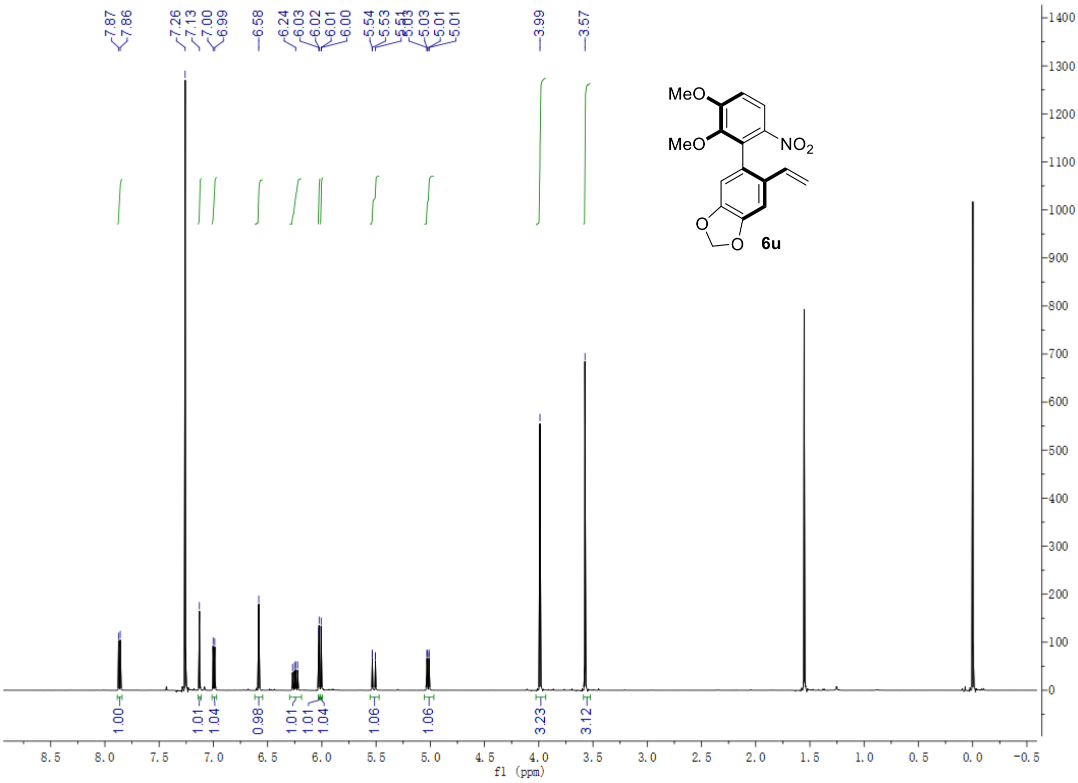

Supplementary Figure 176 ^{13}C NMR spectrum of **6u** (151 MHz, $CDCl_3$)

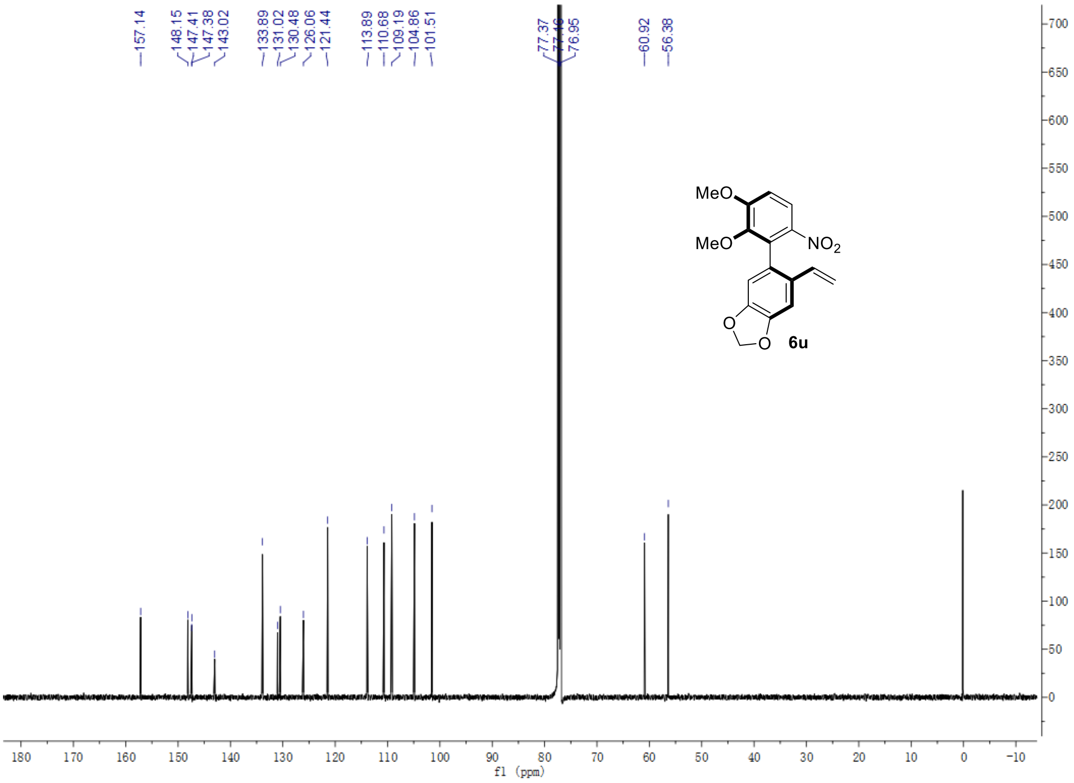

Supplementary Figure 177 ^{1}H NMR spectrum of **6v** (500 MHz, $CDCl_3$)

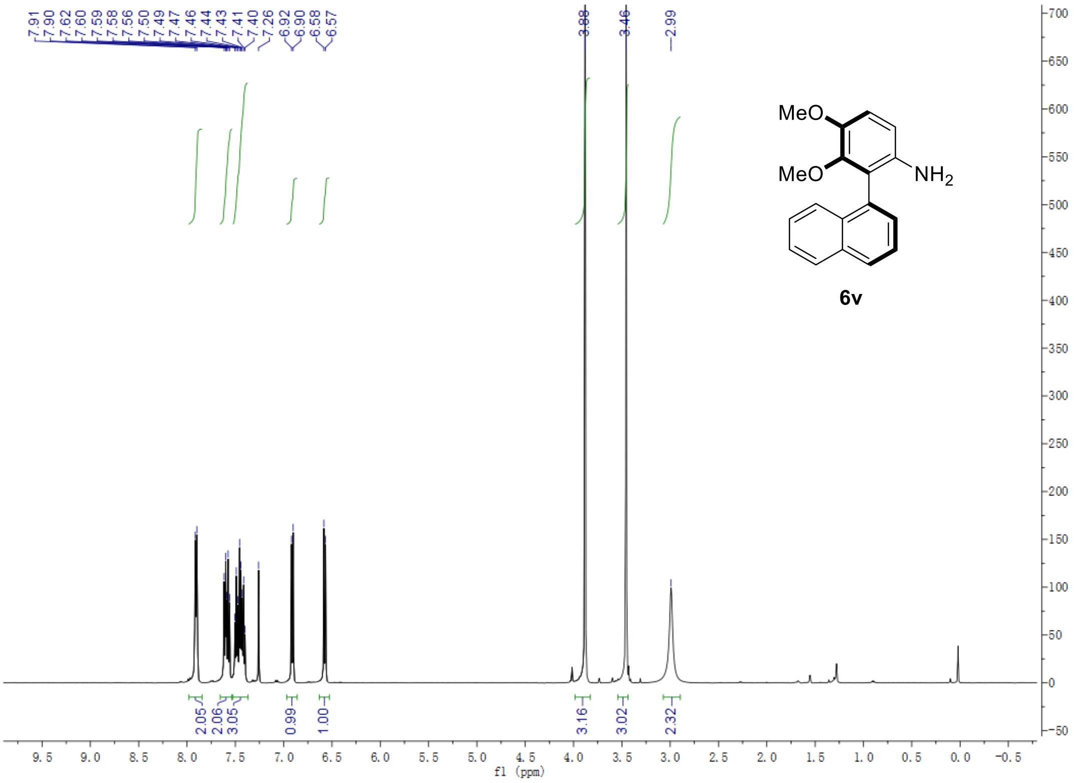

Supplementary Figure 178 ^{13}C NMR spectrum of **6v** (126 MHz, $CDCl_3$)

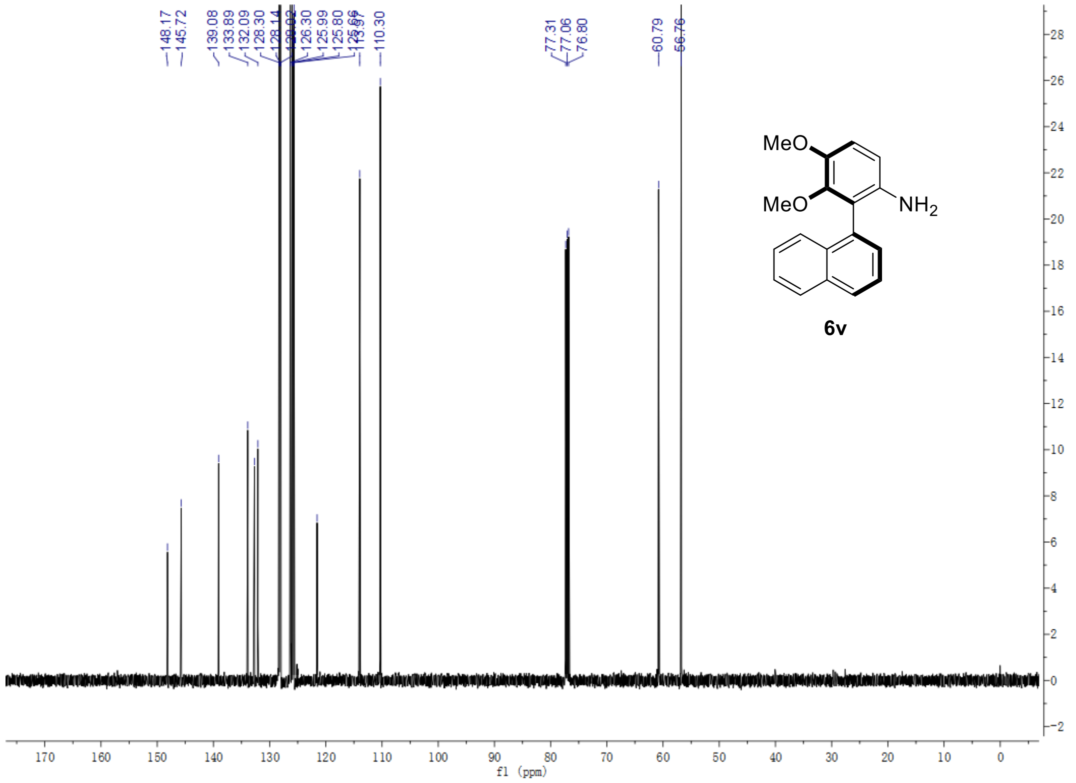

Supplementary Figure 179 ^{1}H NMR spectrum of **6w** (500 MHz, $CDCl_3$)

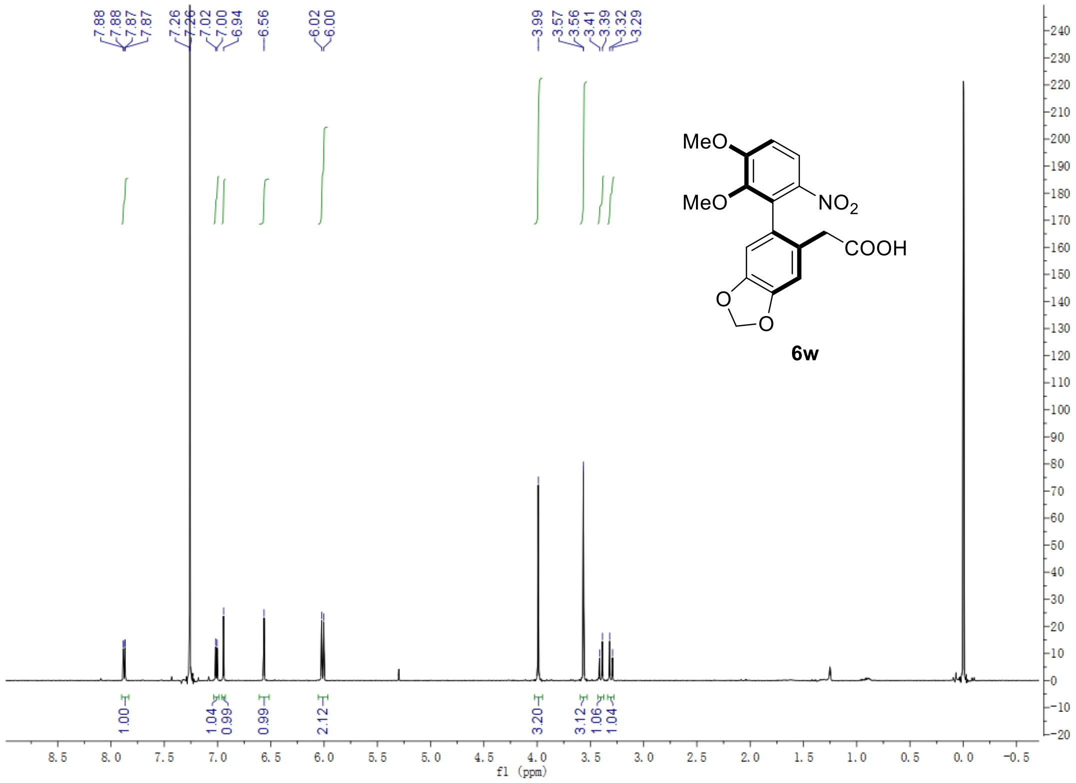

Supplementary Figure 180 ^{13}C NMR spectrum of **6w** (151 MHz, $CDCl_3$)

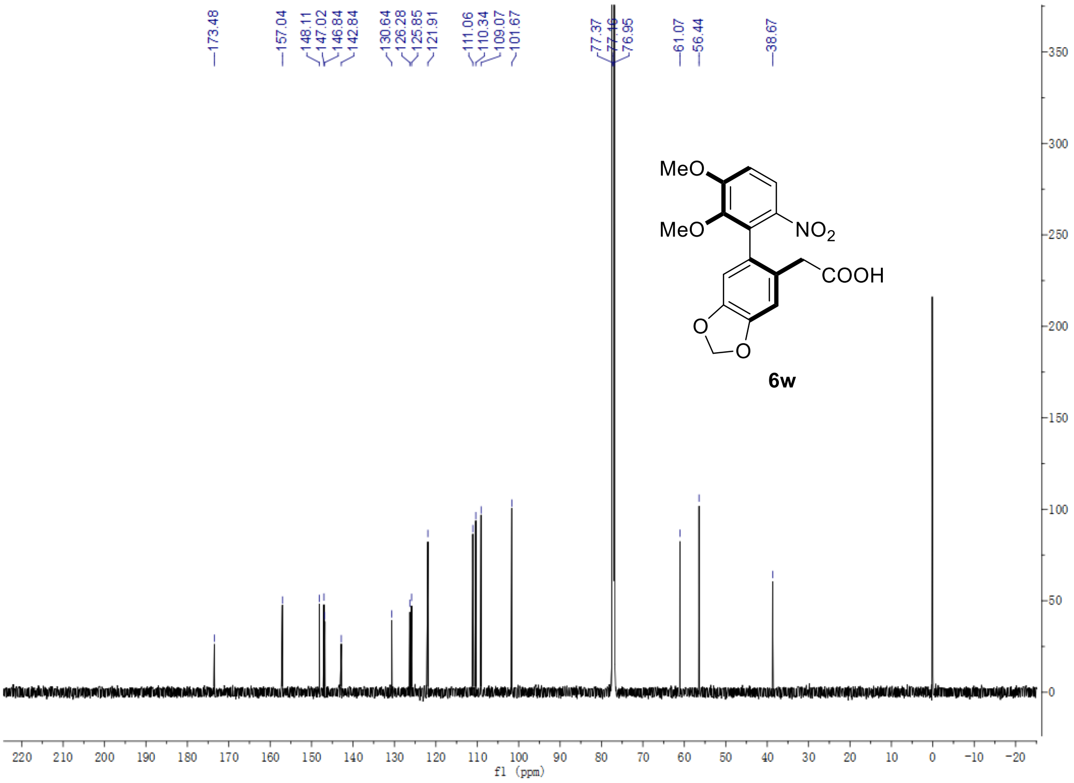

Supplementary Figure 181 ^{1}H NMR spectrum of **3y** (600 MHz, $CDCl_3$)

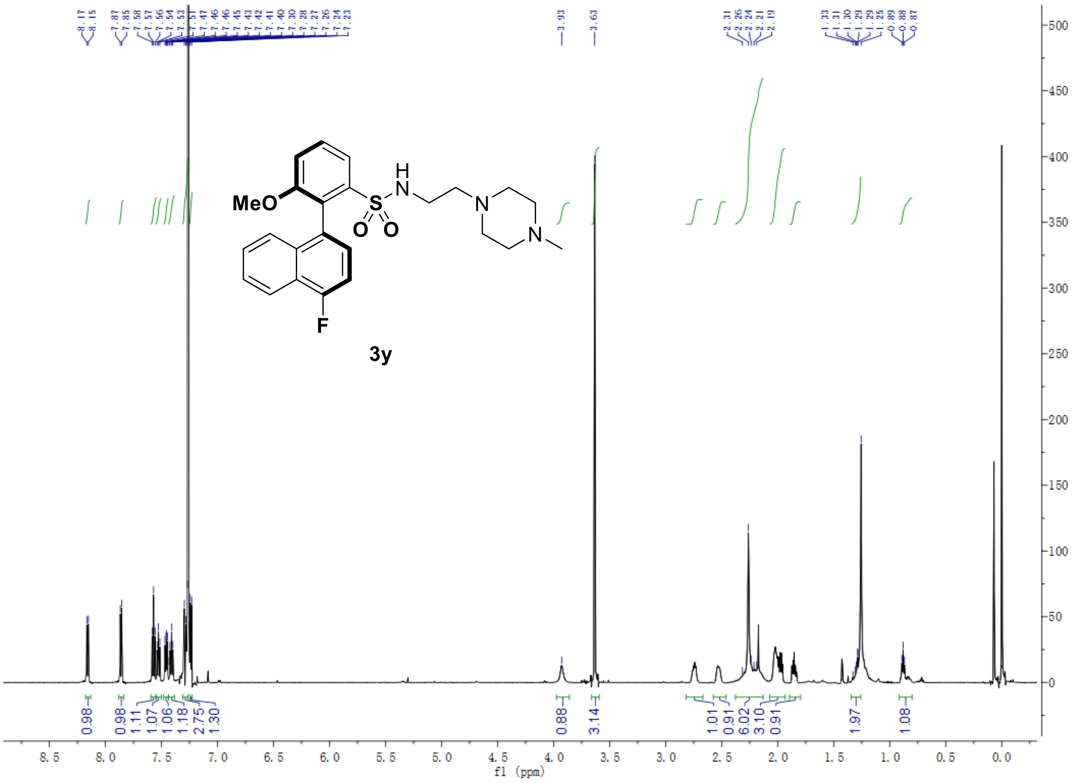

Supplementary Figure 182 ^{13}C NMR spectrum of **3y** (151 MHz, $CDCl_3$)

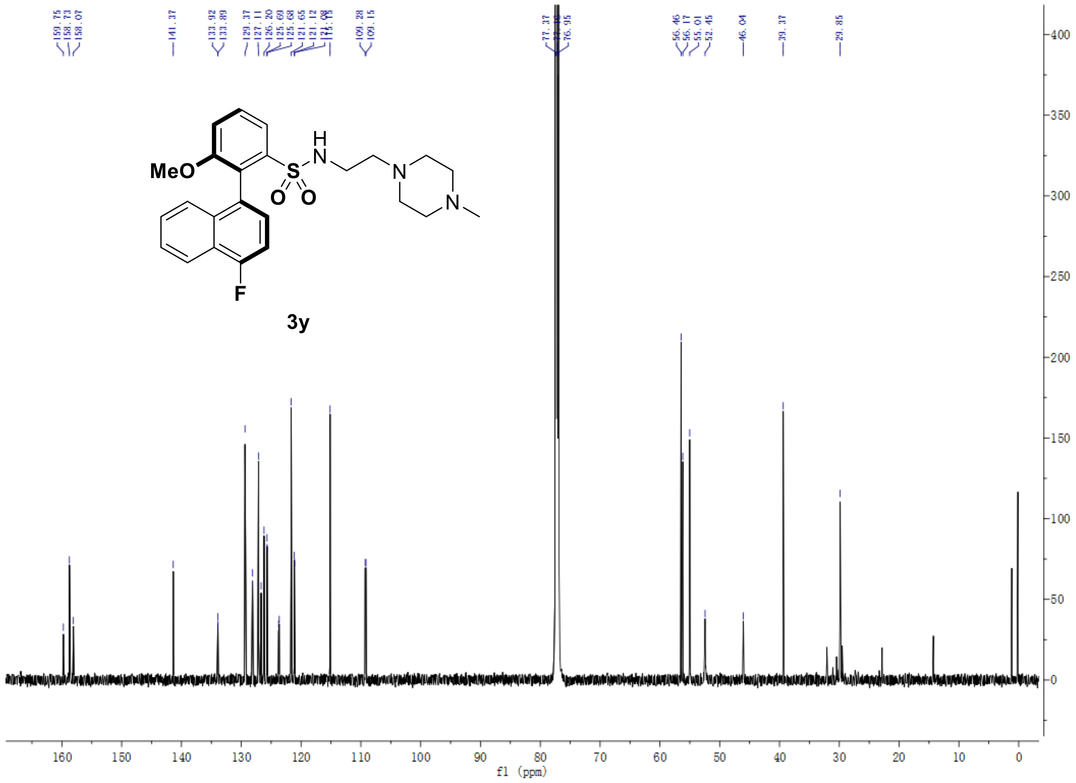

Supplementary Figure 183 ^{19}F NMR spectrum of **3y** (376 MHz, $CDCl_3$)

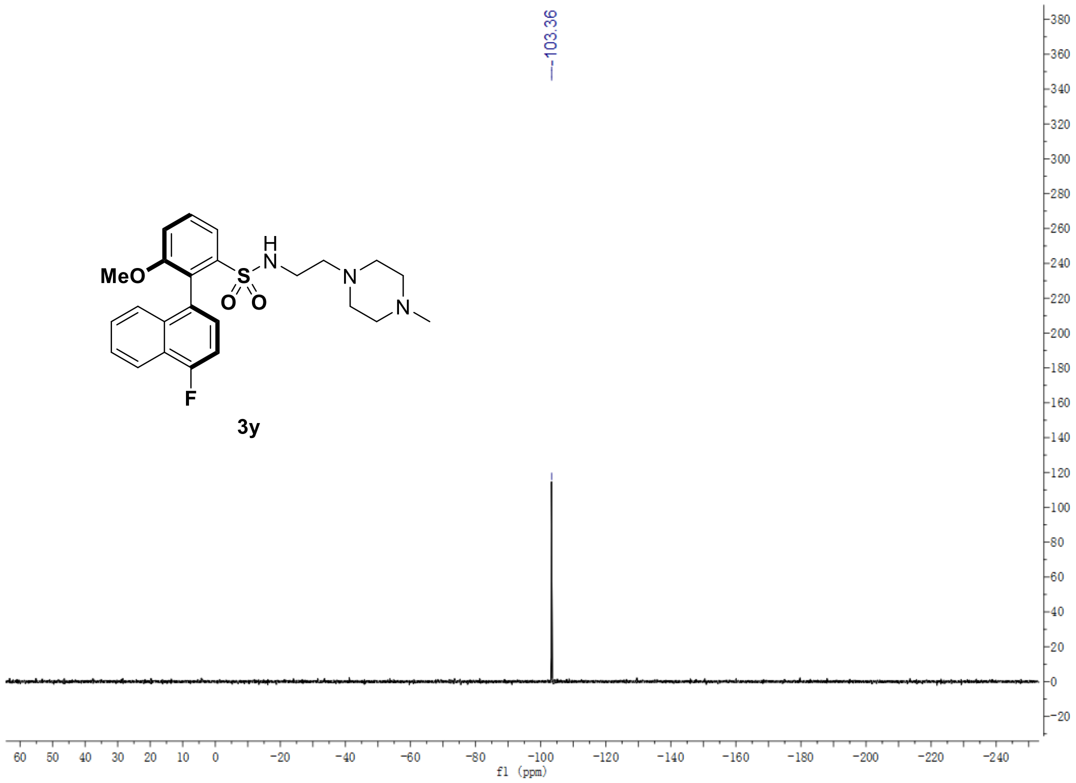

Supplementary Figure 184 ^{1}H NMR spectrum of **3v** (500 MHz, $CDCl_3$)

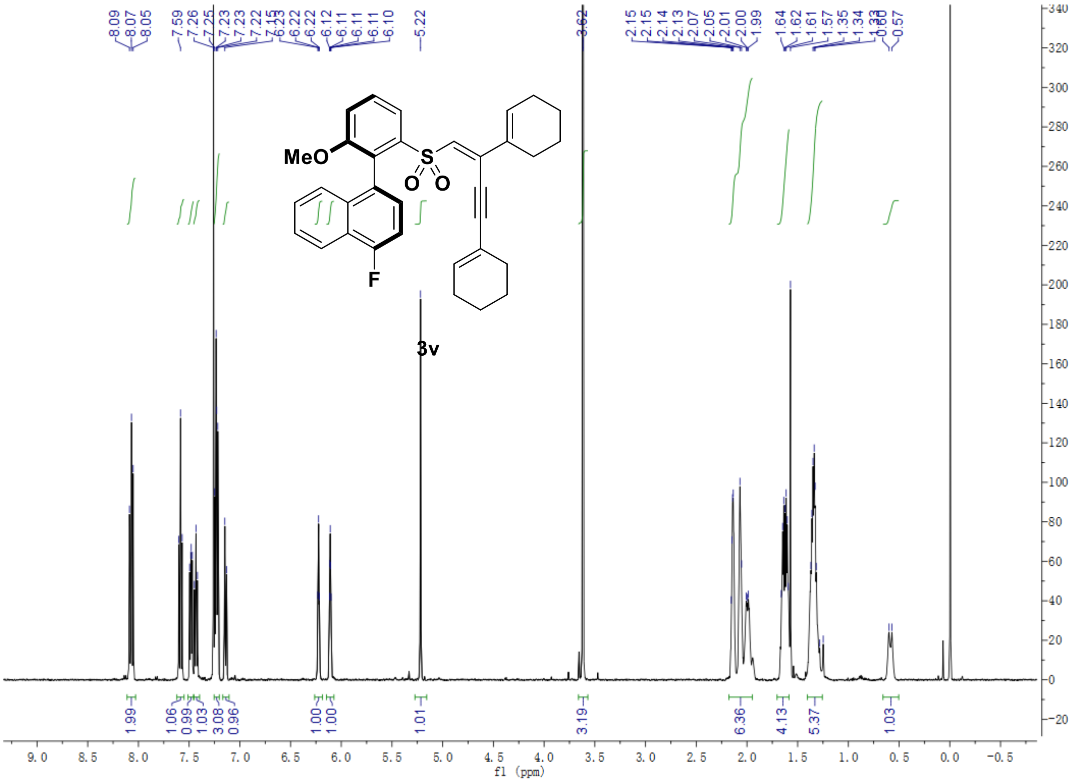

Supplementary Figure 185 ^{13}C NMR spectrum of **3v** (151 MHz, $CDCl_3$)

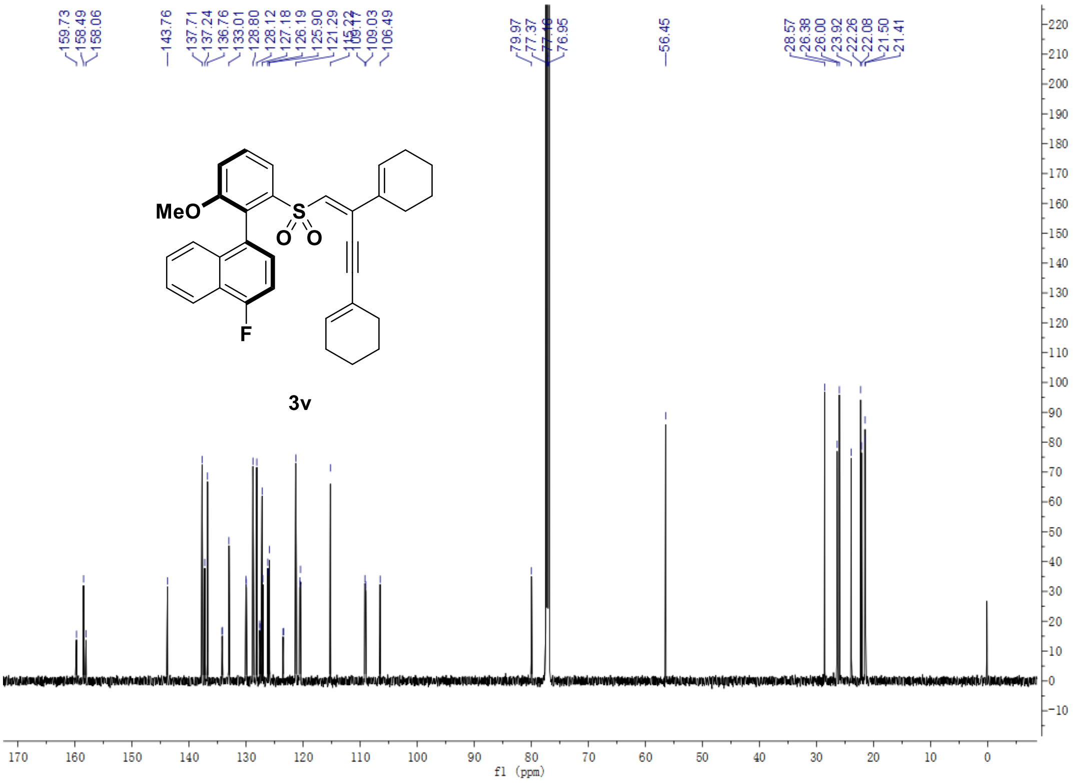

Supplementary Figure 186 ^{19}F NMR spectrum of **3v** (376 MHz, $CDCl_3$)

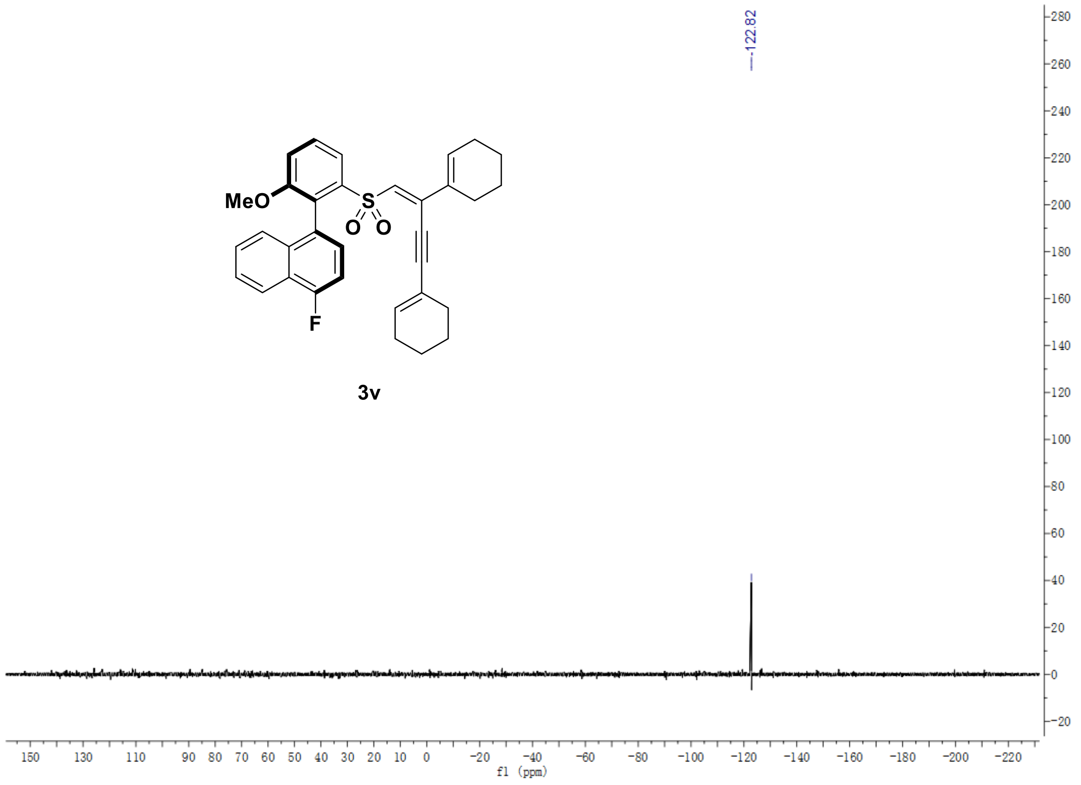

Supplementary Figure 187 ^{1}H NMR spectrum of **3u** (500 MHz, $CDCl_3$)

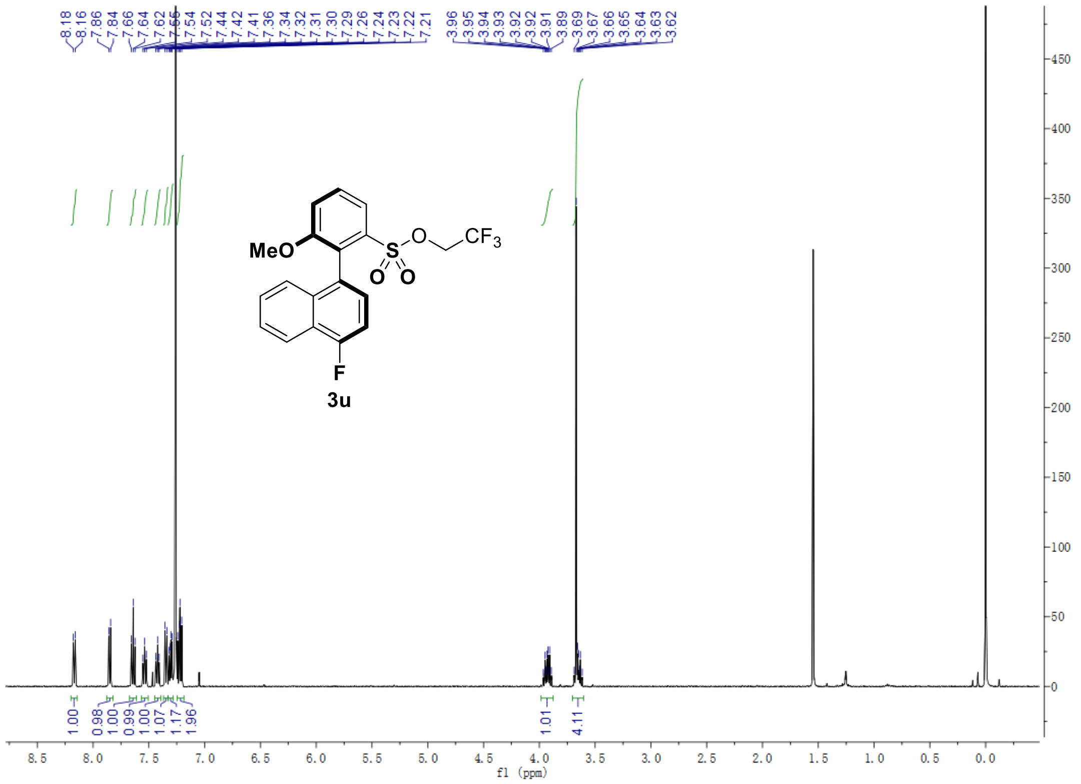

Supplementary Figure 188 ^{13}C NMR spectrum of **3u** (151 MHz, $CDCl_3$)

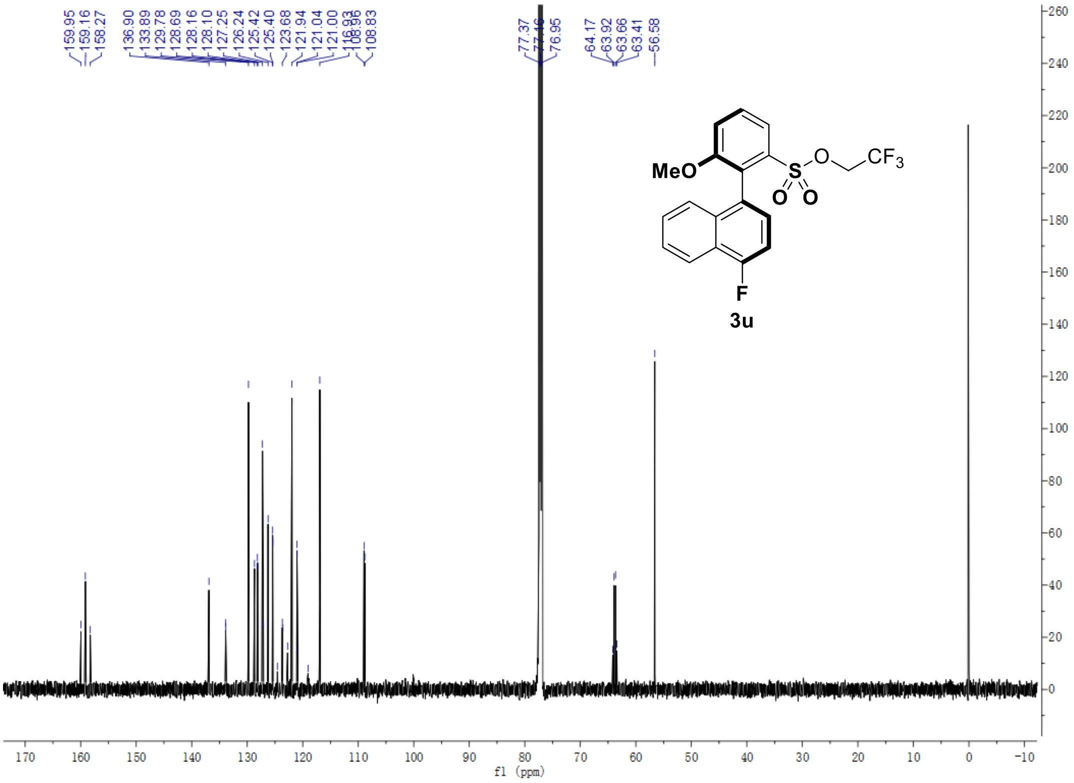

Supplementary Figure 189 ^{19}F NMR spectrum of **3u** (376 MHz, acetone-d_6)

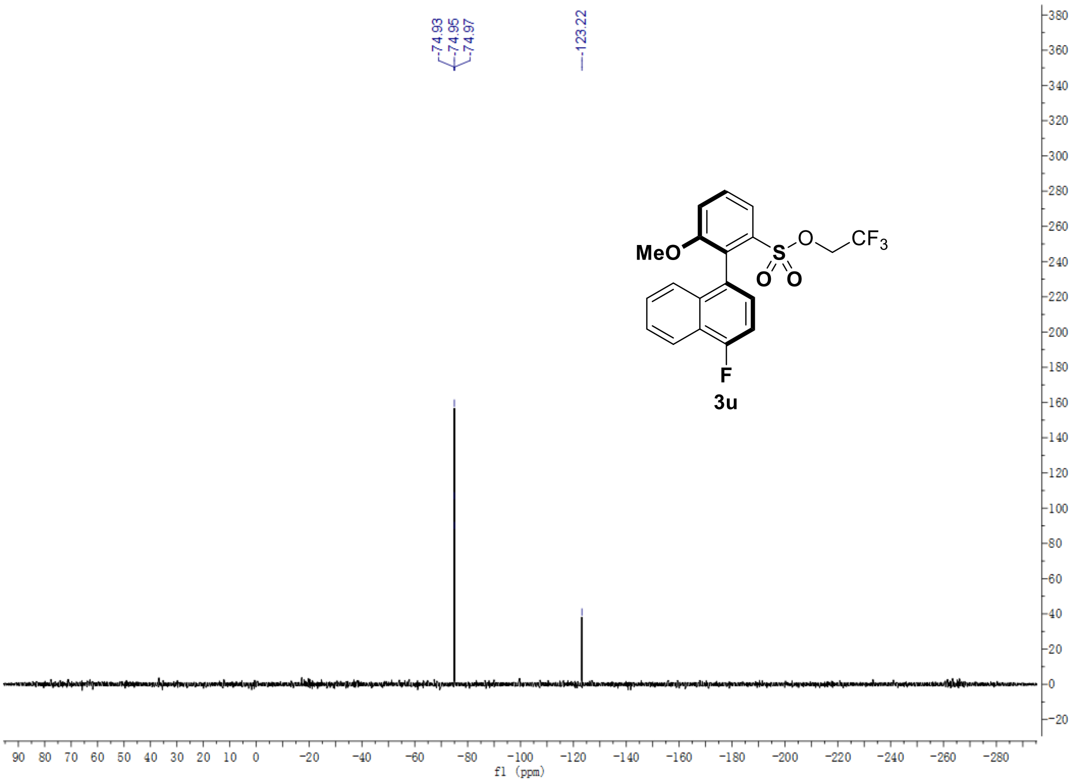

Supplementary Figure 190 ^{1}H NMR spectrum of **3t** (600 MHz, MeOD-d_4)

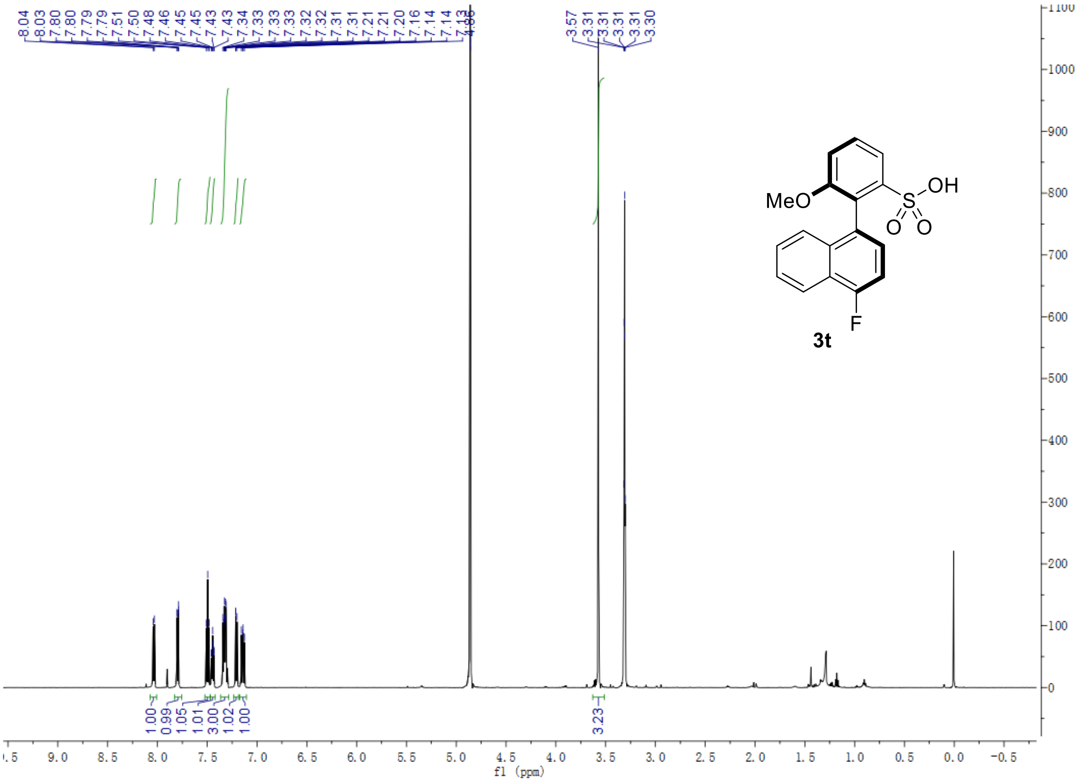

Supplementary Figure 191 ^{13}C NMR spectrum of **3t** (151 MHz, $CDCl_3$)

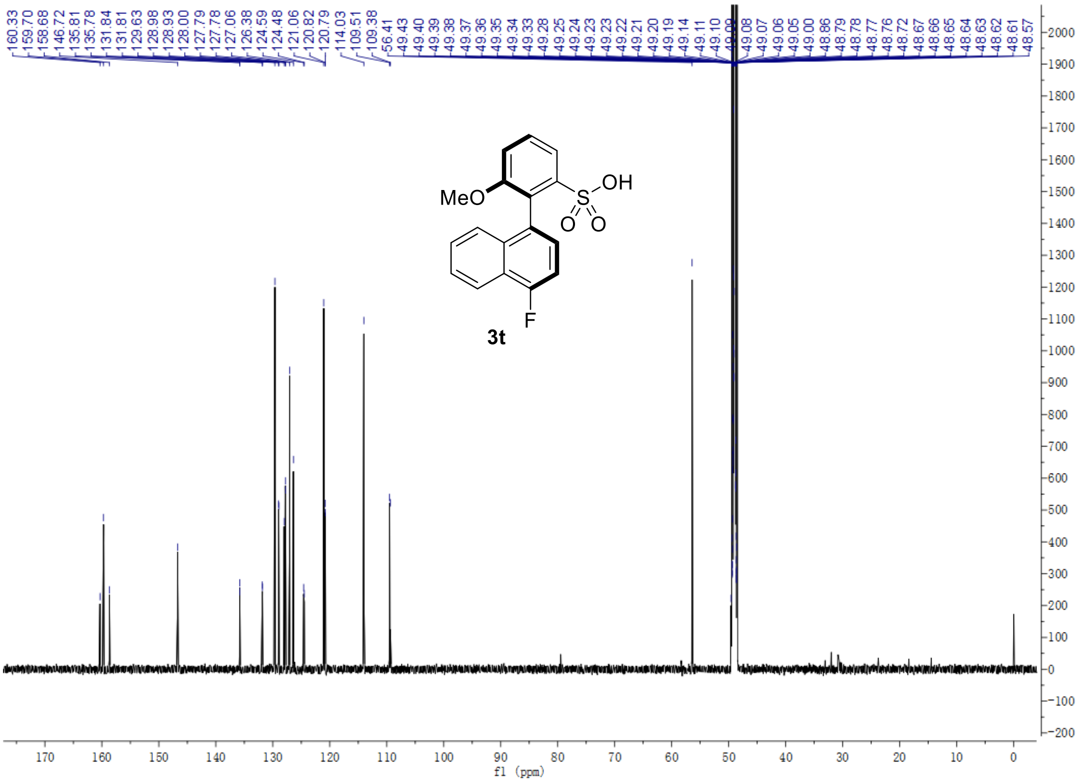

Supplementary Figure 192 ^{19}F NMR spectrum of **3t** (376 MHz, $CDCl_3$)

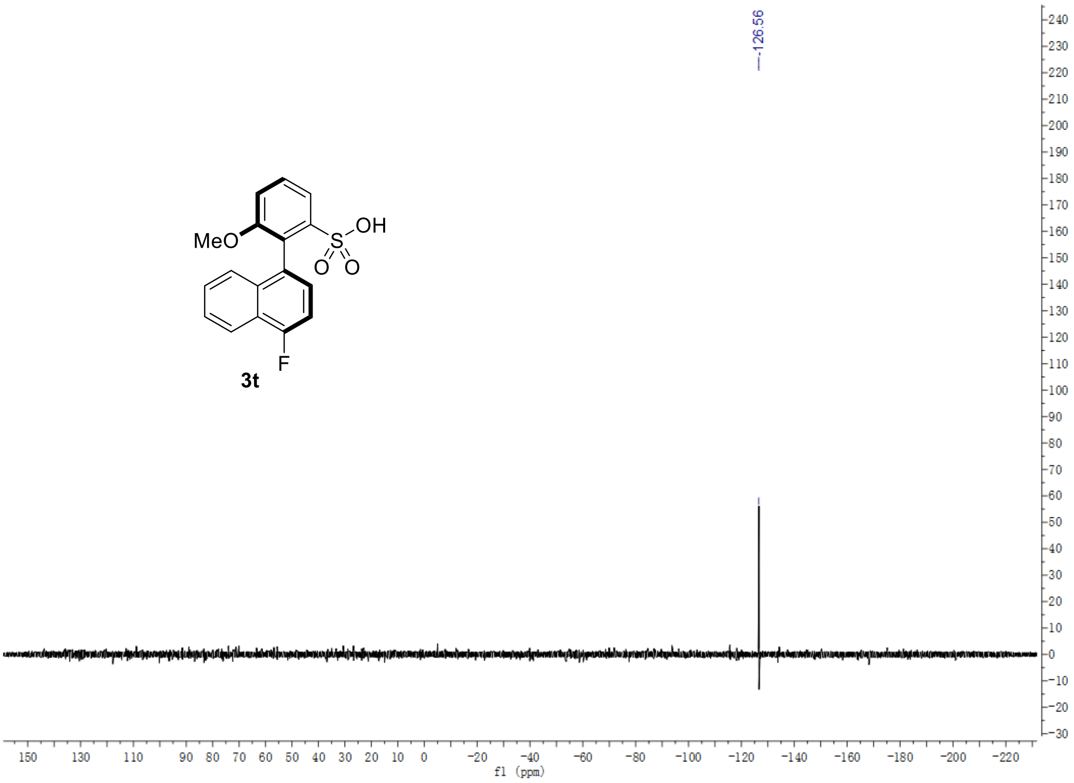

Supplementary Figure 193 ^{1}H NMR spectrum of **17** (500 MHz, $CDCl_3$)

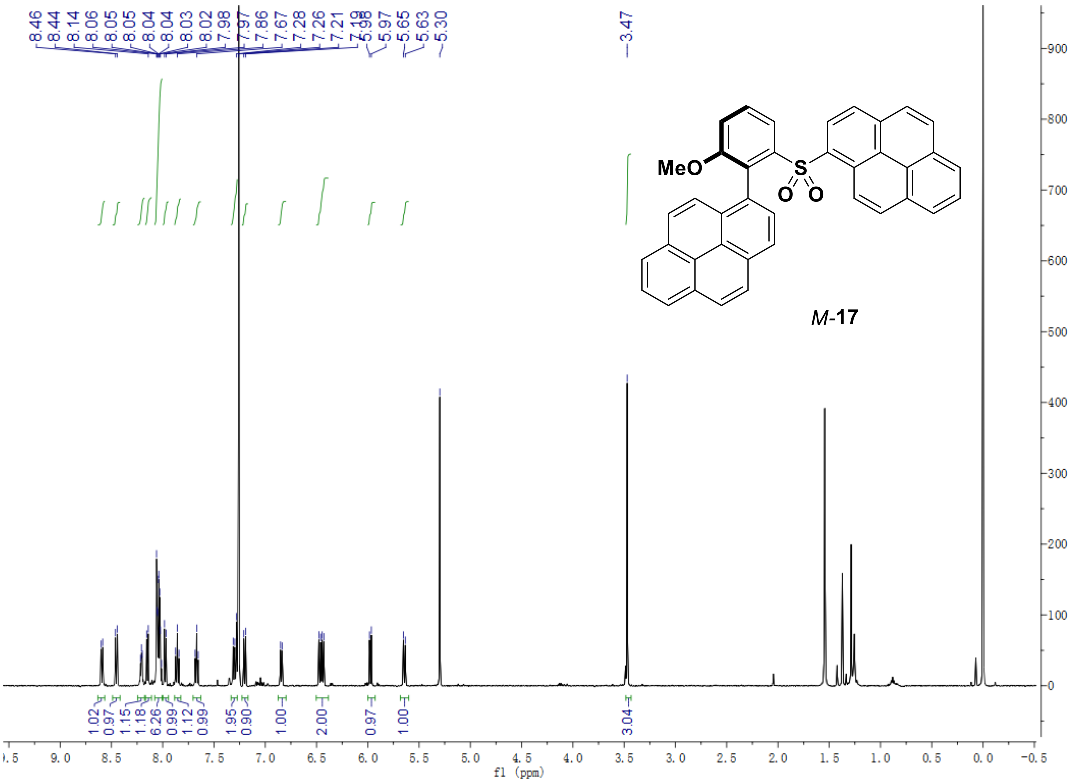

Supplementary Figure 194 ^{13}C NMR spectrum of **17** (151 MHz, $CDCl_3$)

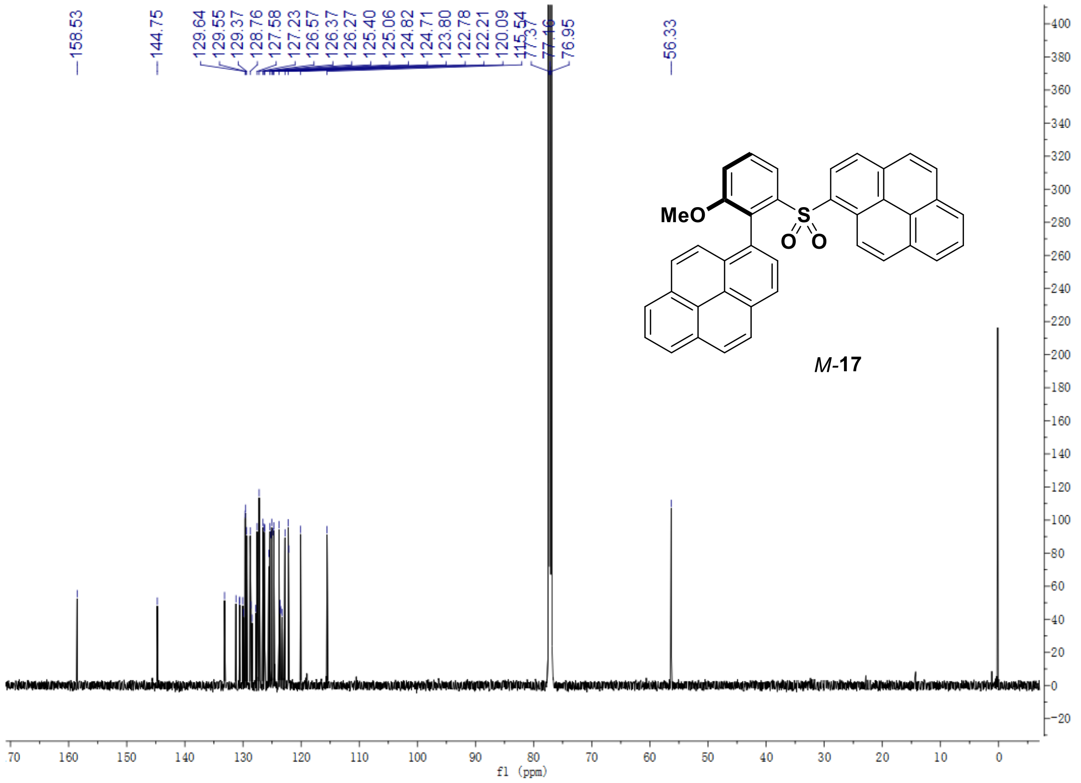

Supplementary Figure 195 ^{1}H NMR spectrum of **3w** (400 MHz, $CDCl_3$)

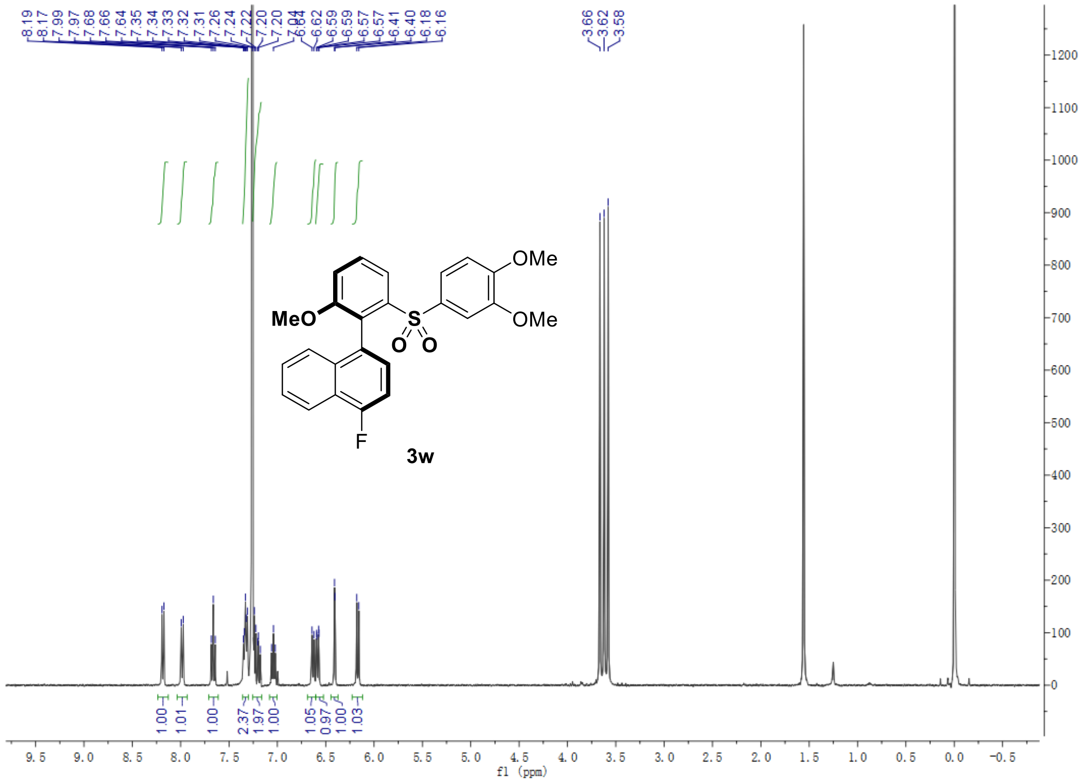

Supplementary Figure 196 ^{13}C NMR spectrum of **3w** (151 MHz, $CDCl_3$)

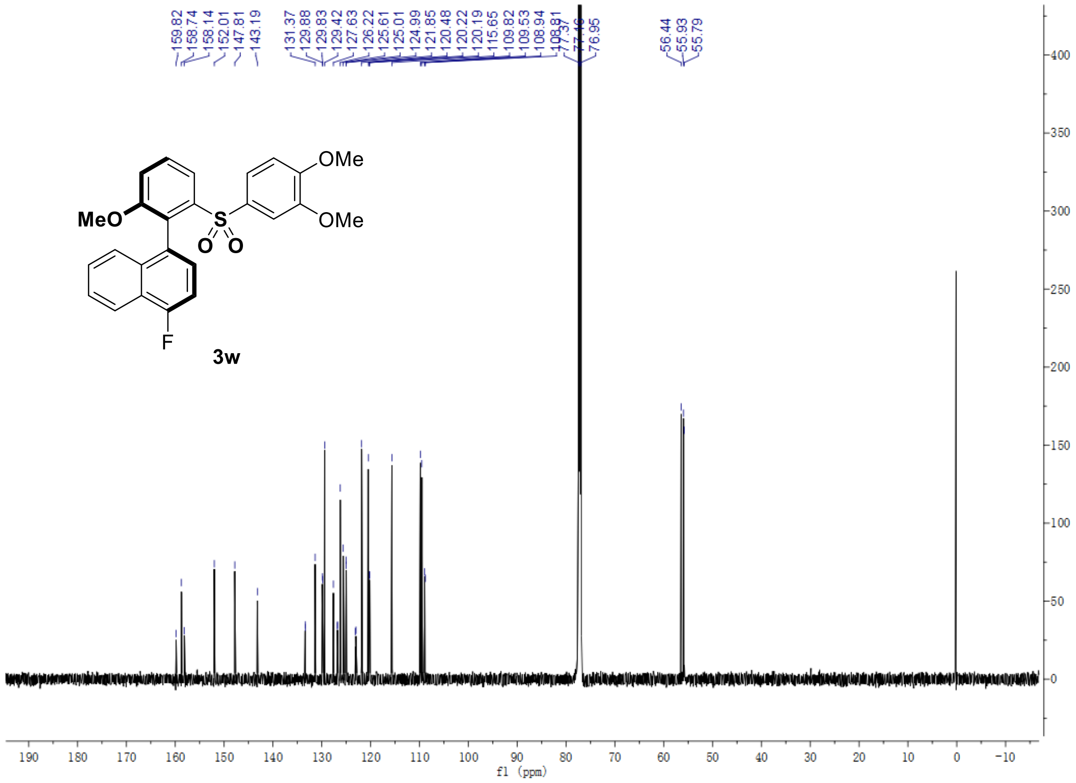

Supplementary Figure 197 ^{19}F NMR spectrum of **3w** (376 MHz, $CDCl_3$)

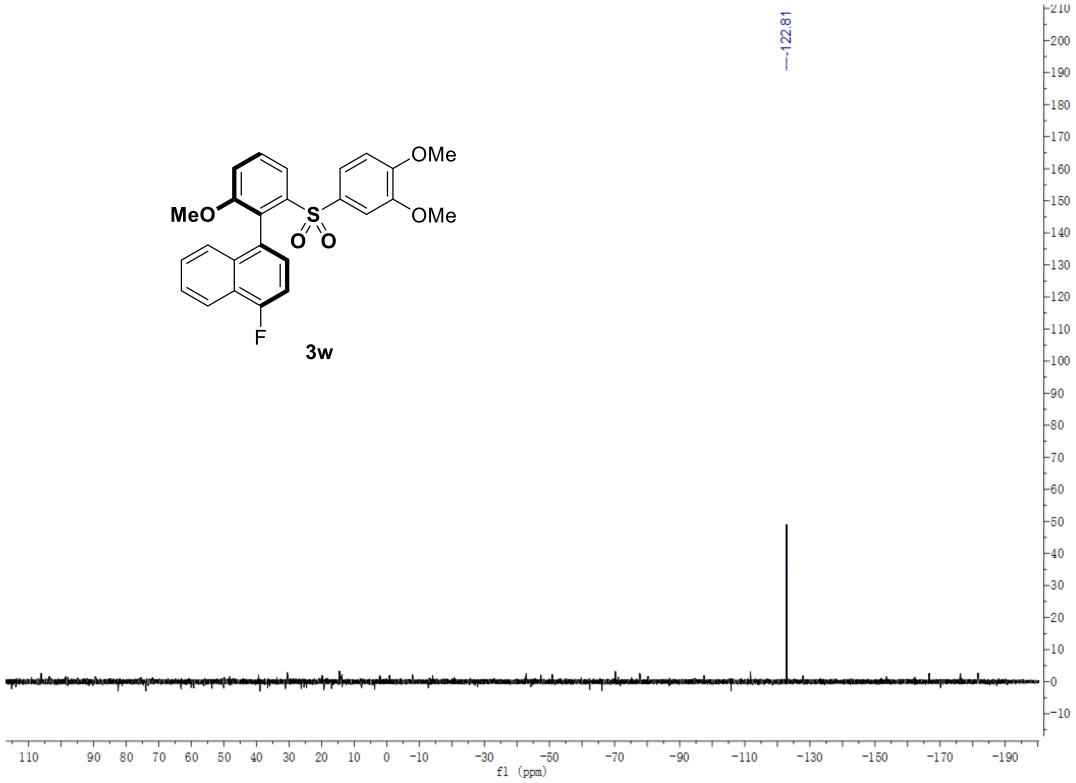

Supplementary Figure 198 ^{1}H NMR spectrum of **3x** (400 MHz, $CDCl_3$)

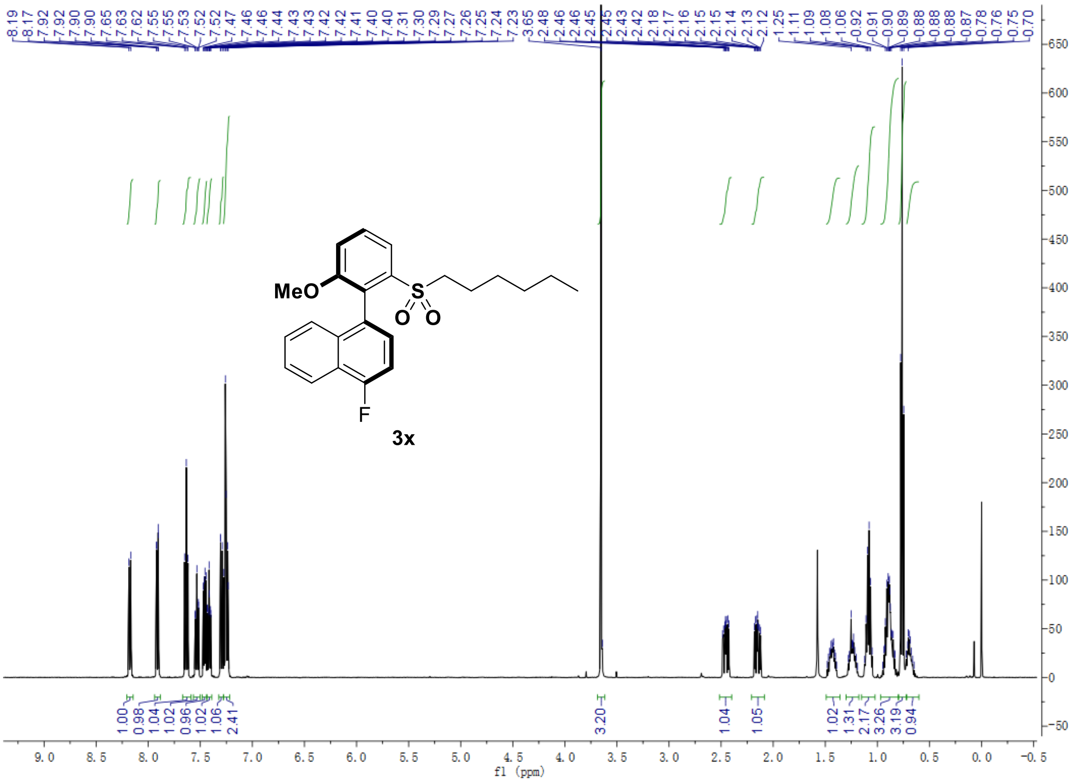

Supplementary Figure 199 ^{13}C NMR spectrum of **3x** (151 MHz, $CDCl_3$)

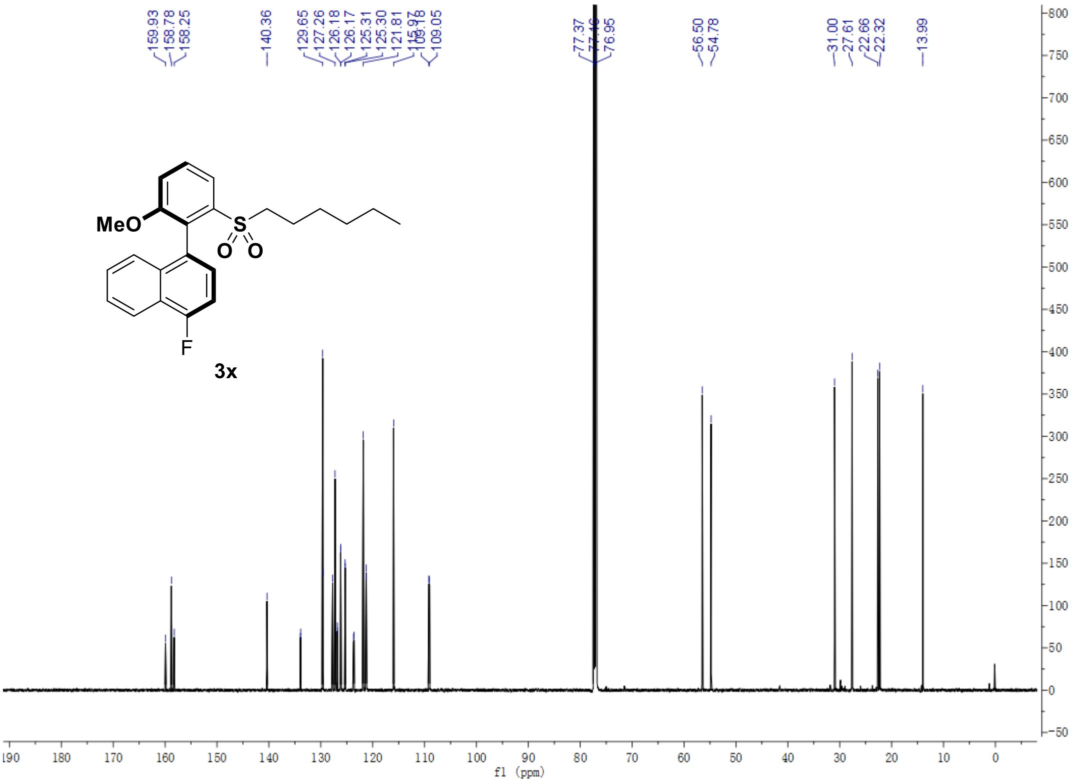

Supplementary Figure 200 ^{19}F NMR spectrum of **3x** (376 MHz, $CDCl_3$)

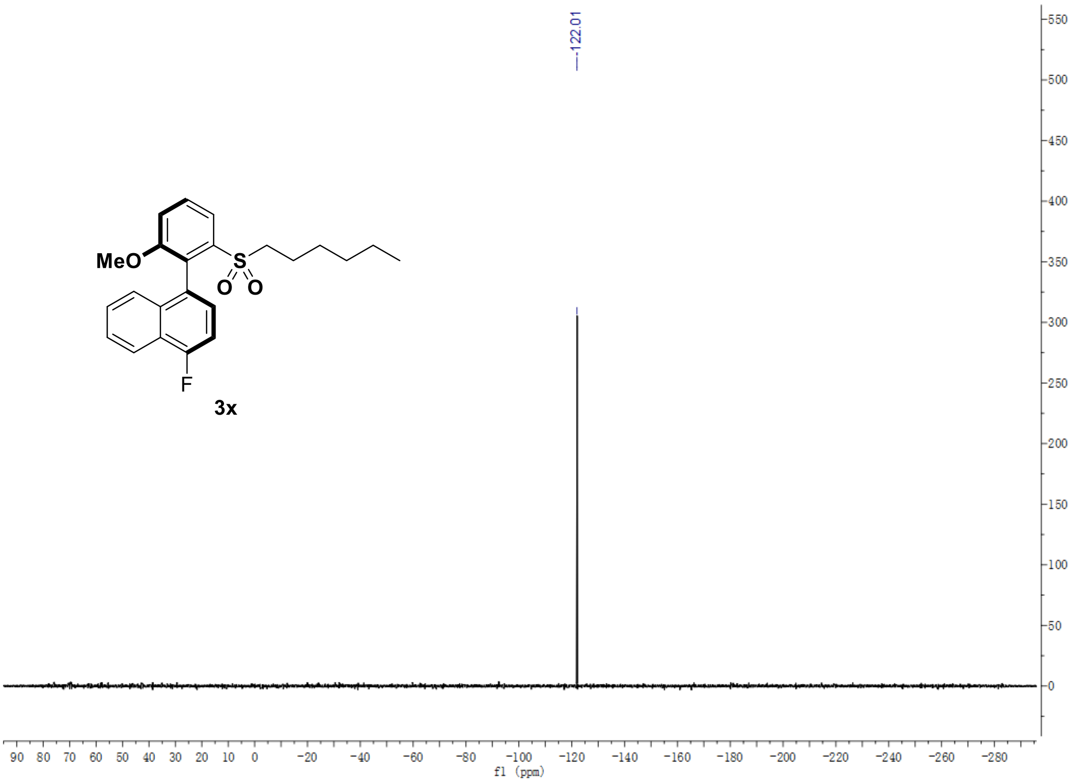

Supplementary Figure 201 ^{1}H NMR spectrum of **S32** (400 MHz, $CDCl_3$)

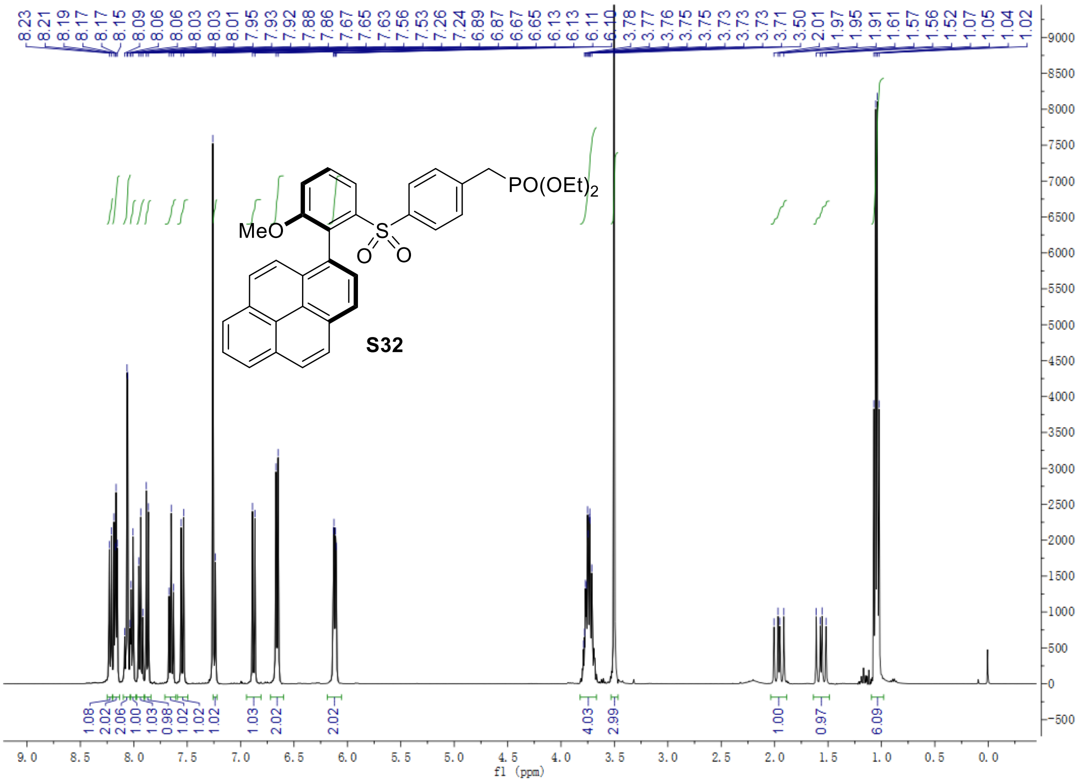

Supplementary Figure 202 ^{13}C NMR spectrum of **S32** (151 MHz, $CDCl_3$)

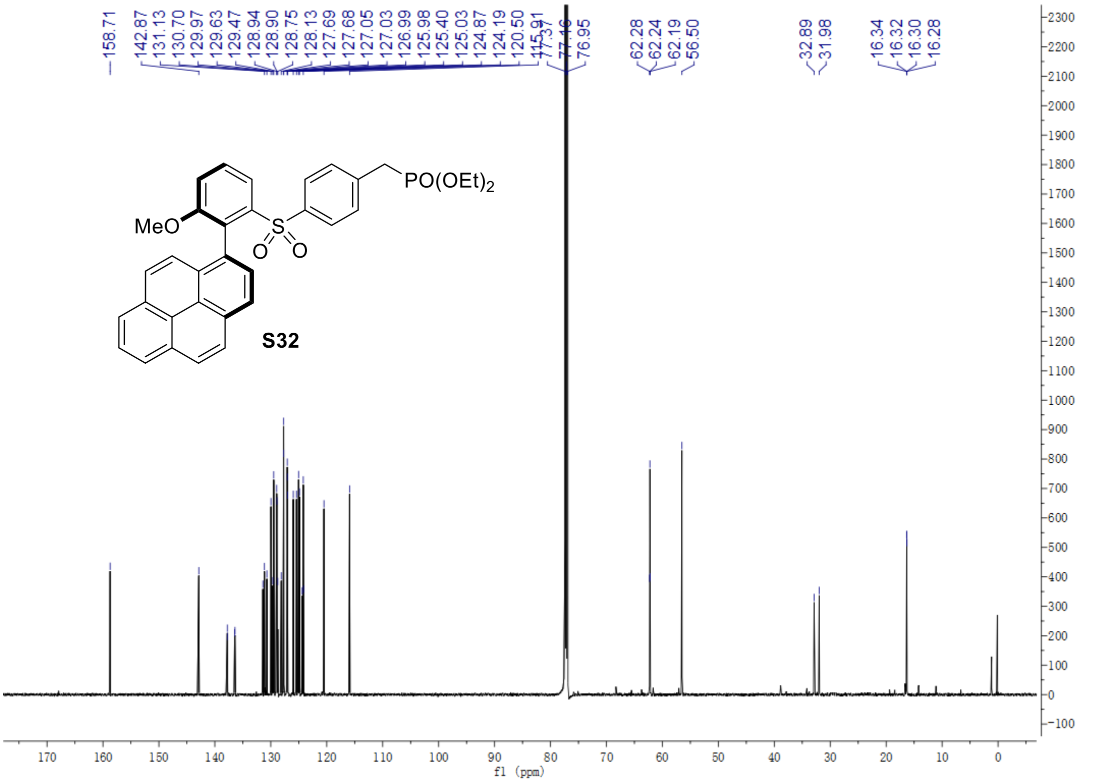

Supplementary Figure 203 ^{31}P NMR spectrum of **3r** (162 MHz, $CDCl_3$)

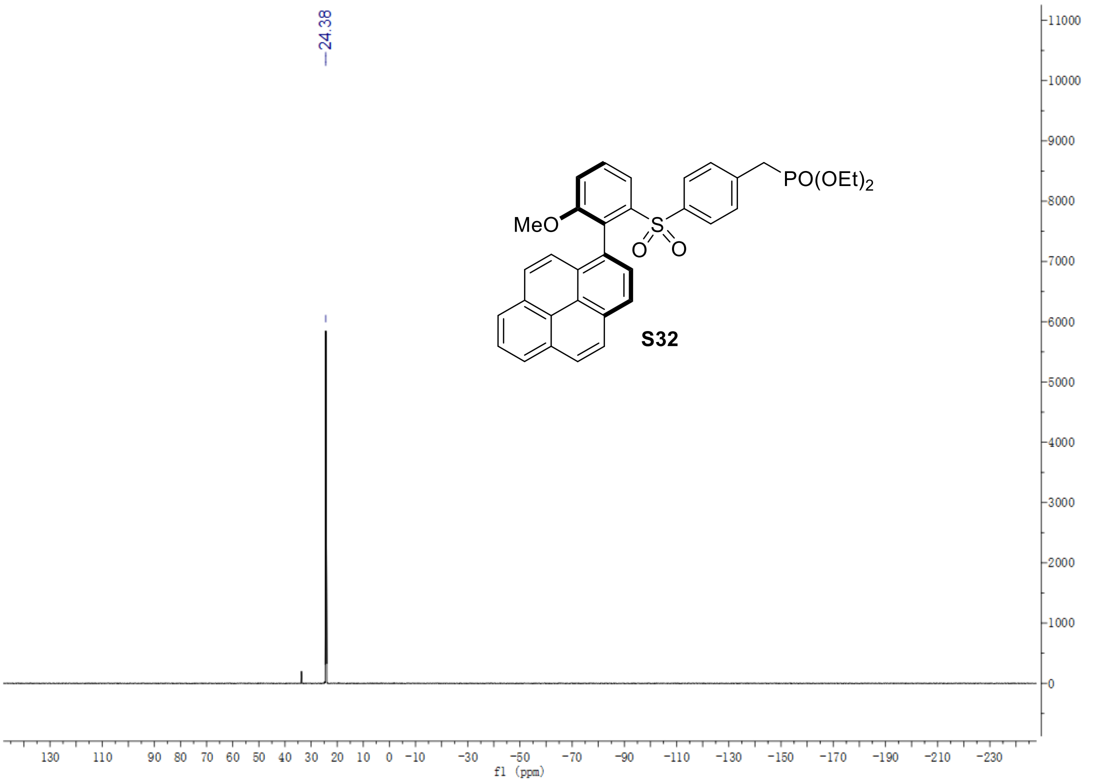

Supplementary Figure 204 ^{1}H NMR spectrum of **18** (600 MHz, $CDCl_3$)

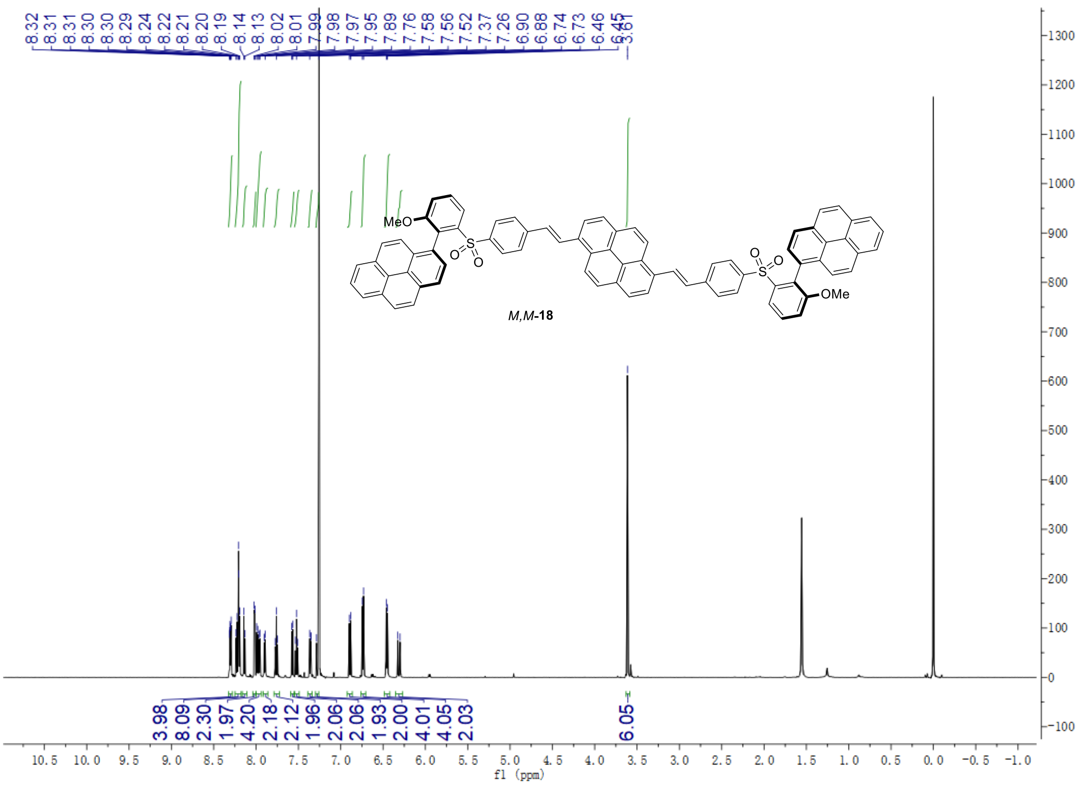

Supplementary Figure 205 ^{13}C NMR spectrum of **18** (151 MHz, $CDCl_3$)

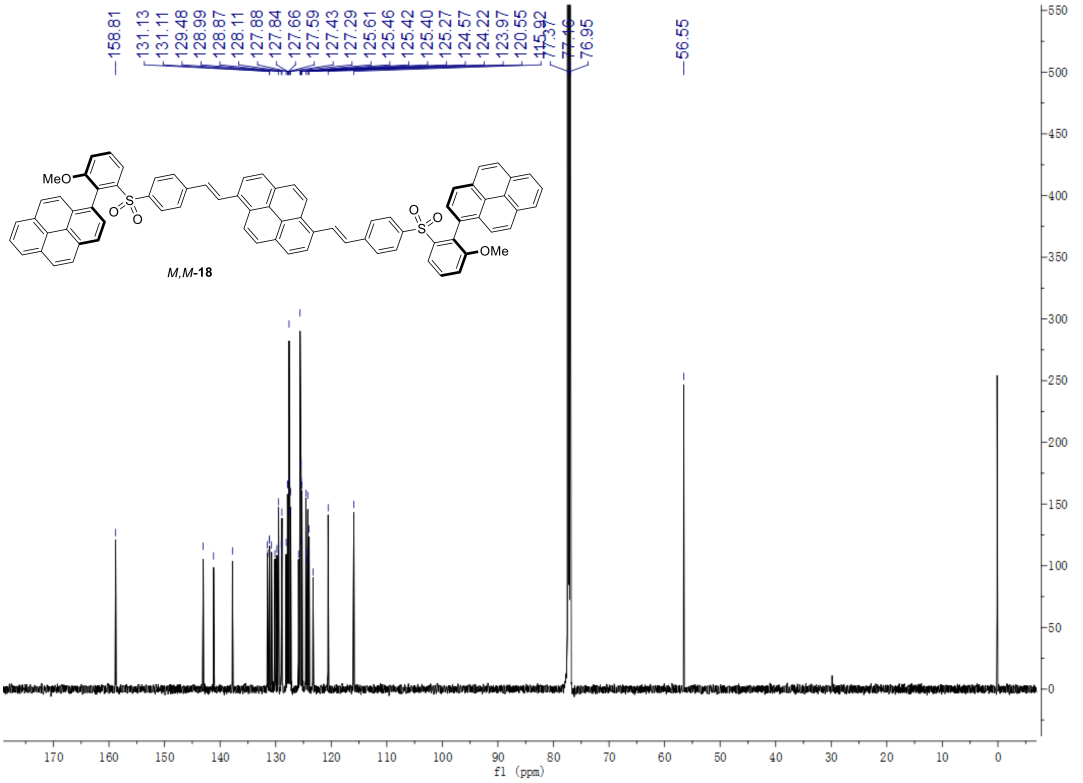

Supplementary Figure 206 ^{1}H NMR spectrum of **8** (400 MHz, $CDCl_3$)

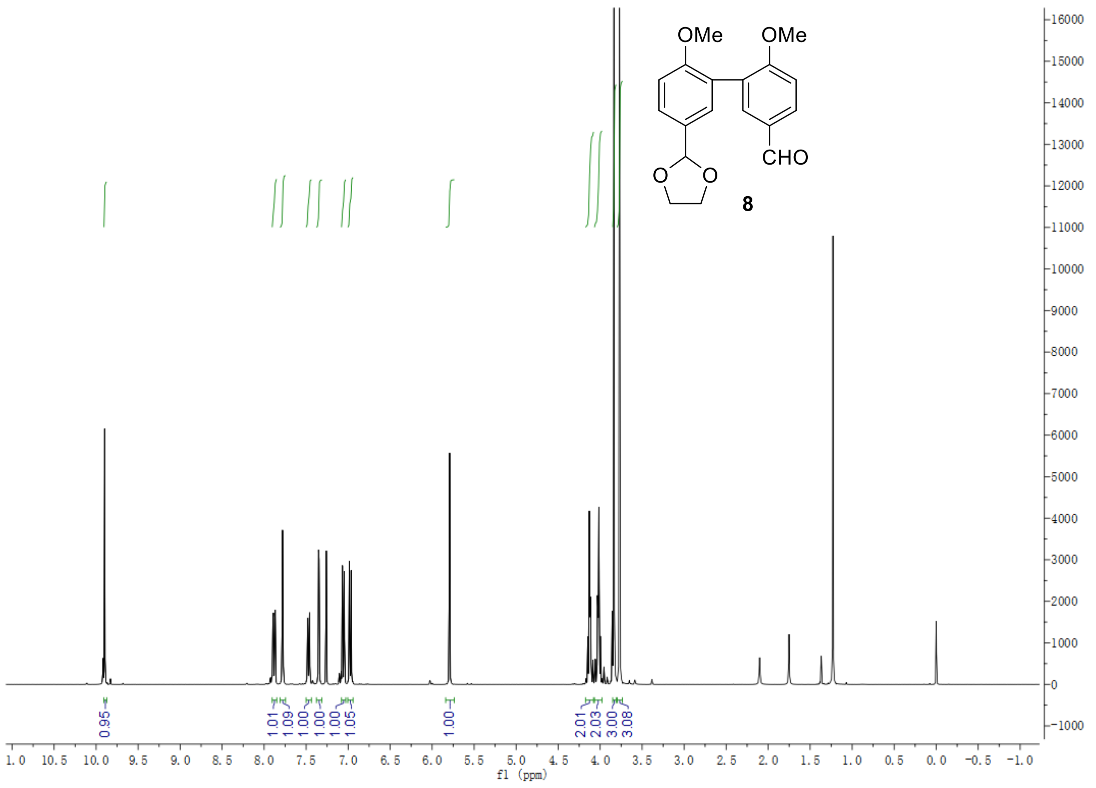

Supplementary Figure 207 ^{13}C NMR spectrum of **8** (126 MHz, $CDCl_3$)

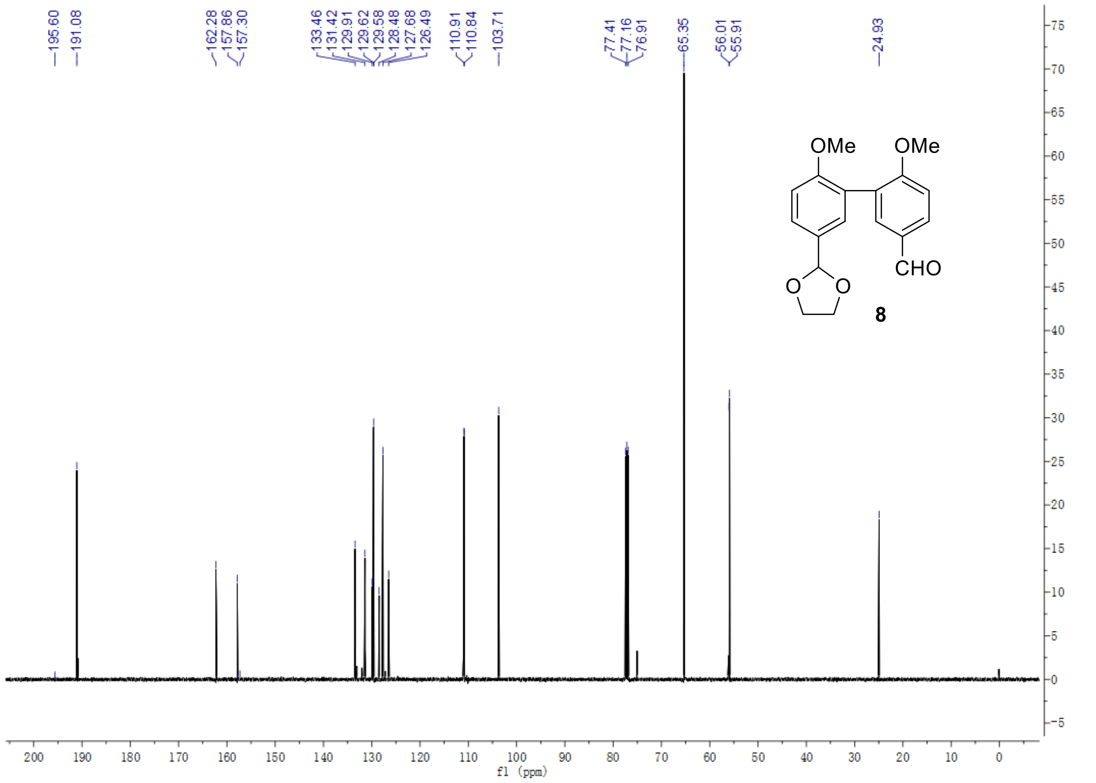

Supplementary Figure 208 ^{1}H NMR spectrum of **9** (500 MHz, $CDCl_3$)

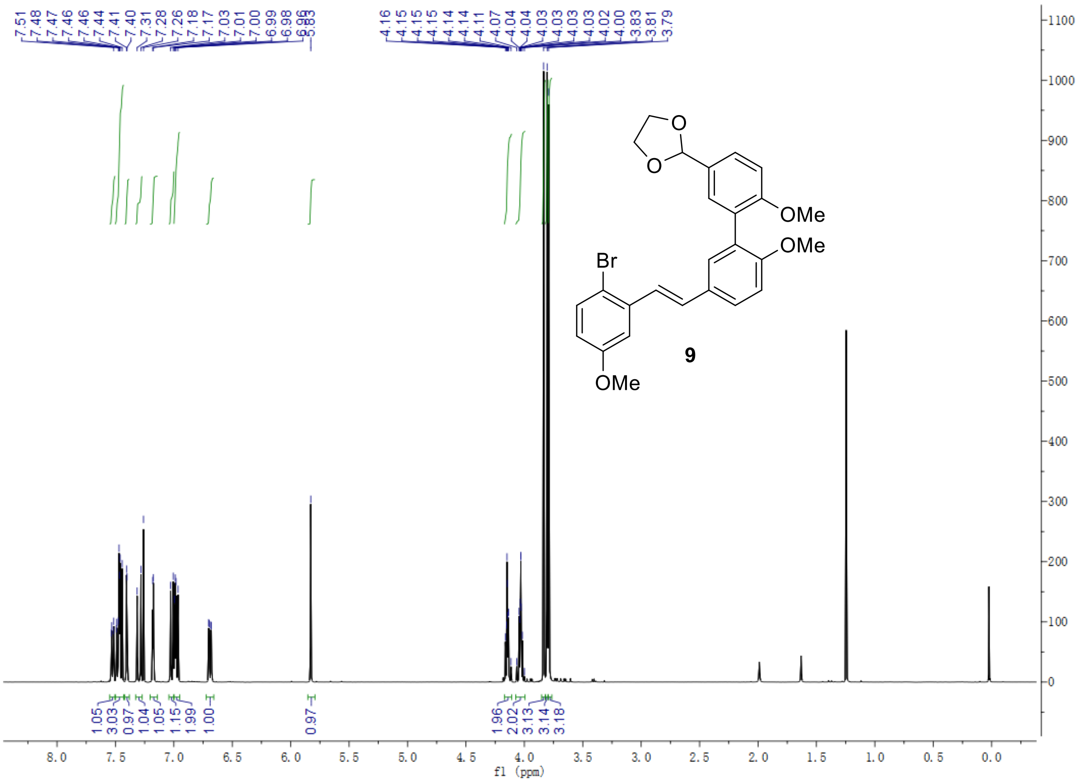

Supplementary Figure 209 ^{13}C NMR spectrum of **9** (126 MHz, $CDCl_3$)

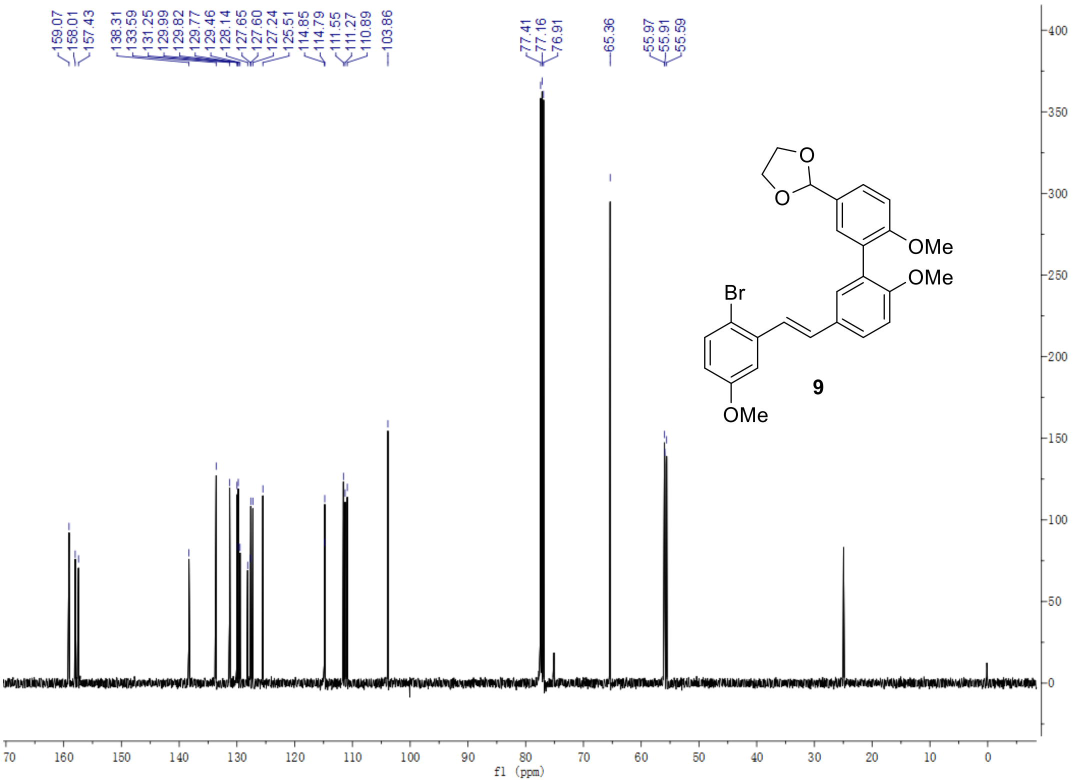

Supplementary Figure 210 ^{1}H NMR spectrum of **10** (500 MHz, $CDCl_3$)

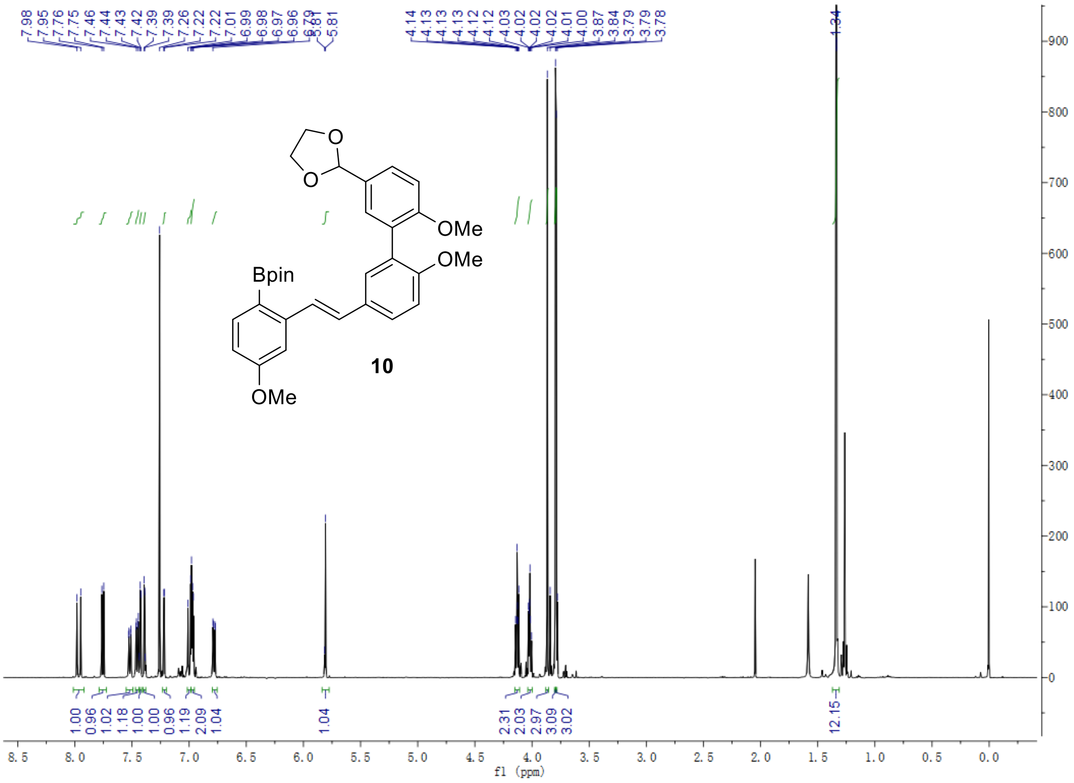

Supplementary Figure 211 ^{13}C NMR spectrum of **10** (126 MHz, $CDCl_3$)

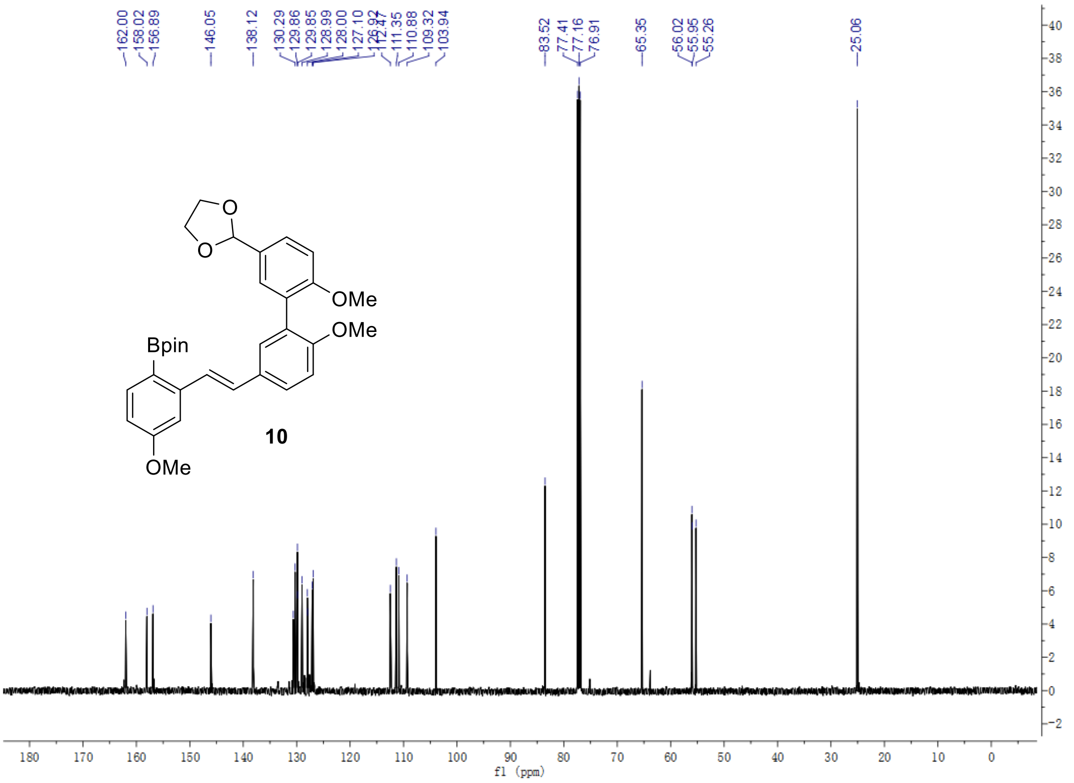

Supplementary Figure 212 ^{1}H NMR spectrum of **12** (500 MHz, $CDCl_3$)

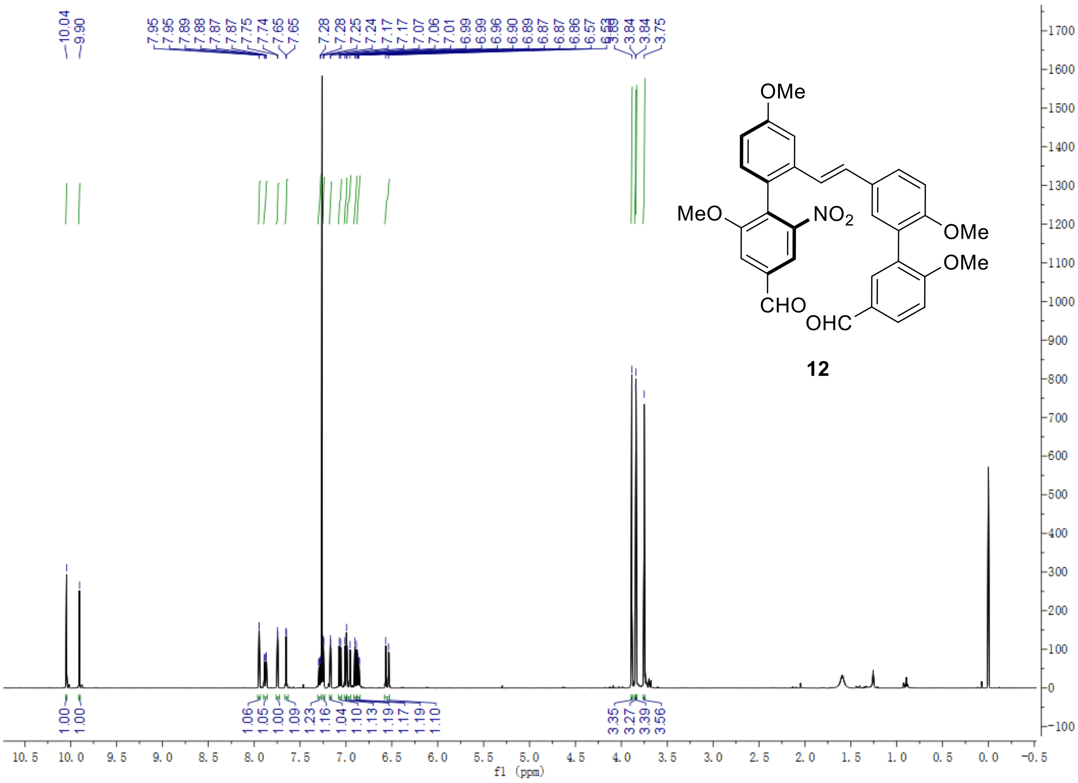

Supplementary Figure 213 ^{13}C NMR spectrum of **12** (151 MHz, $CDCl_3$)

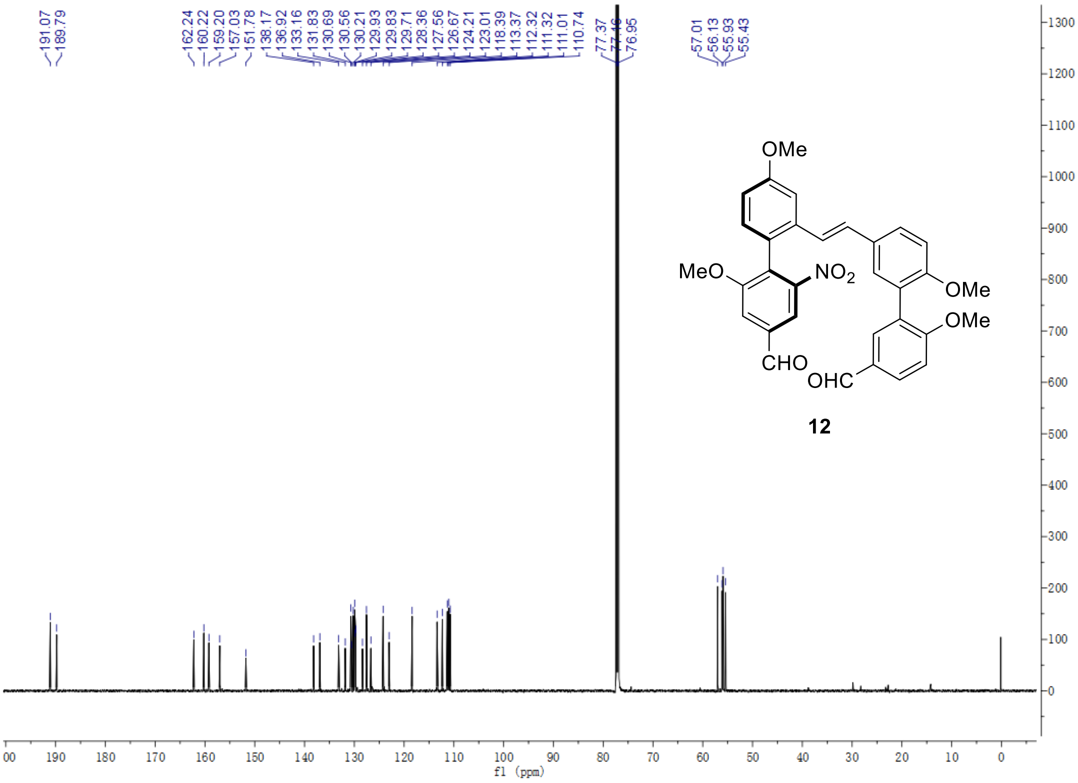

Supplementary Figure 214 ^{1}H NMR spectrum of **13** (500 MHz, $CDCl_3$)

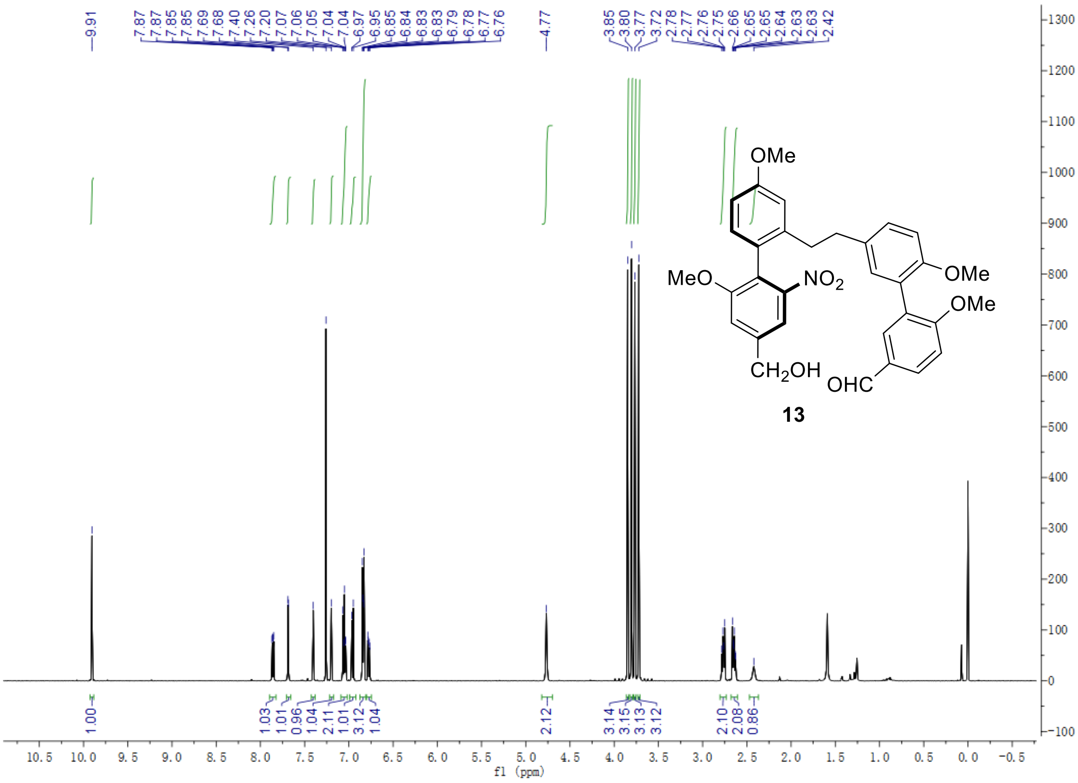

Supplementary Figure 215 ^{13}C NMR spectrum of **13** (151 MHz, $CDCl_3$)

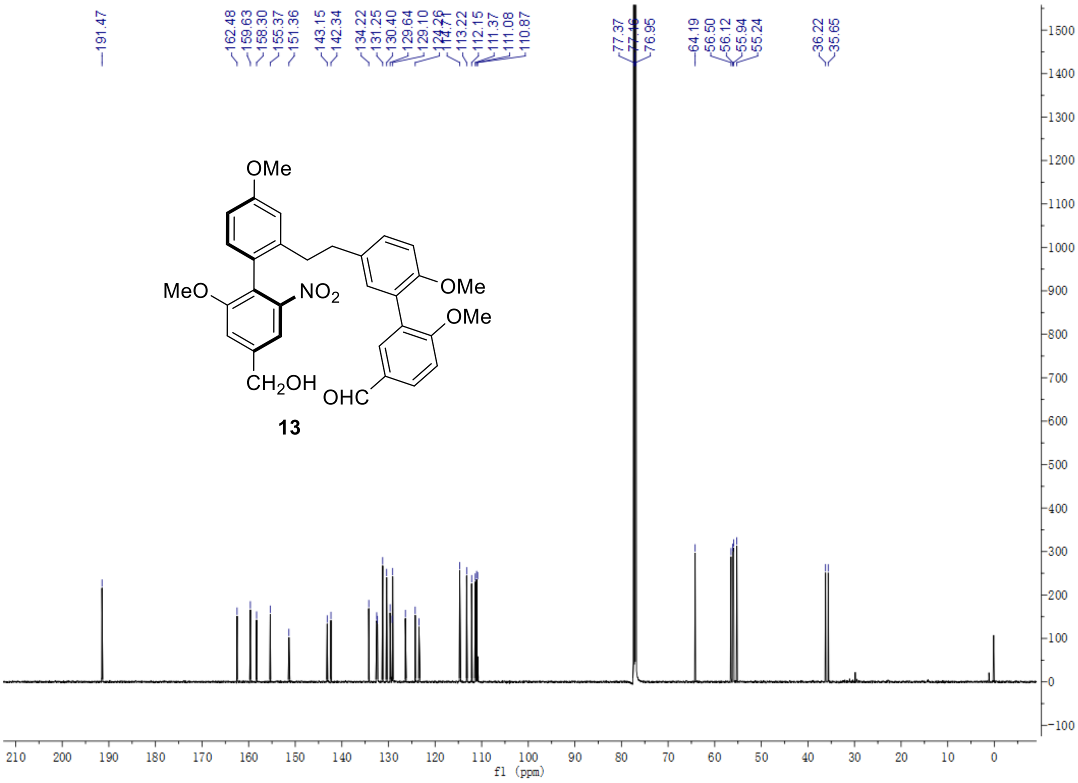

Supplementary Figure 216 ^{1}H NMR spectrum of **14** (400 MHz, $CDCl_3$)

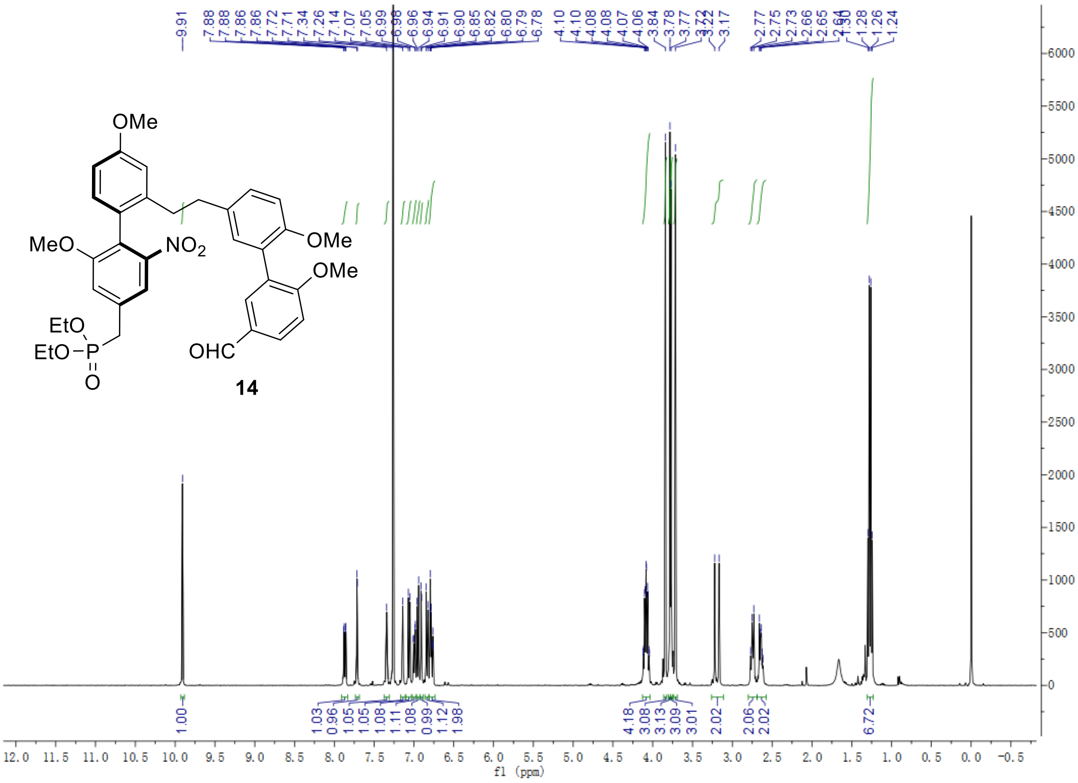

Supplementary Figure 217 ^{13}C NMR spectrum of **14** (151 MHz, $CDCl_3$)

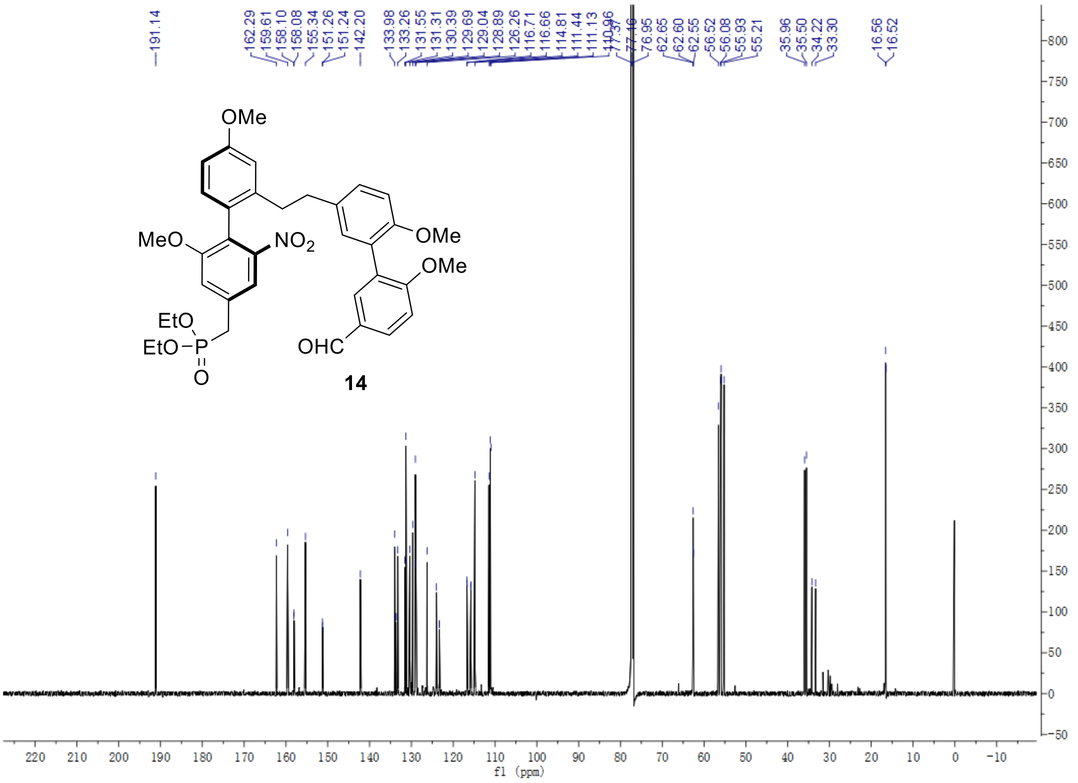

Supplementary Figure 218 ^{31}P NMR spectrum of **14** (162 MHz, $CDCl_3$)

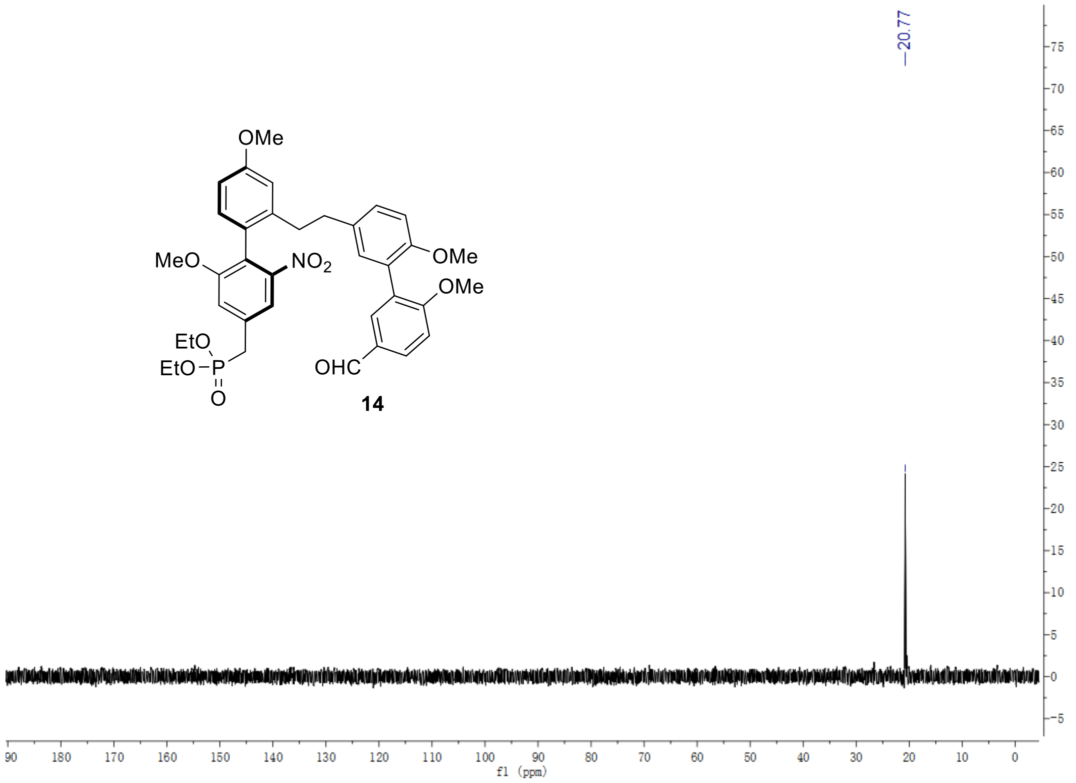

Supplementary Figure 219 ^{1}H NMR spectrum of **16** (400 MHz, $CDCl_3$)

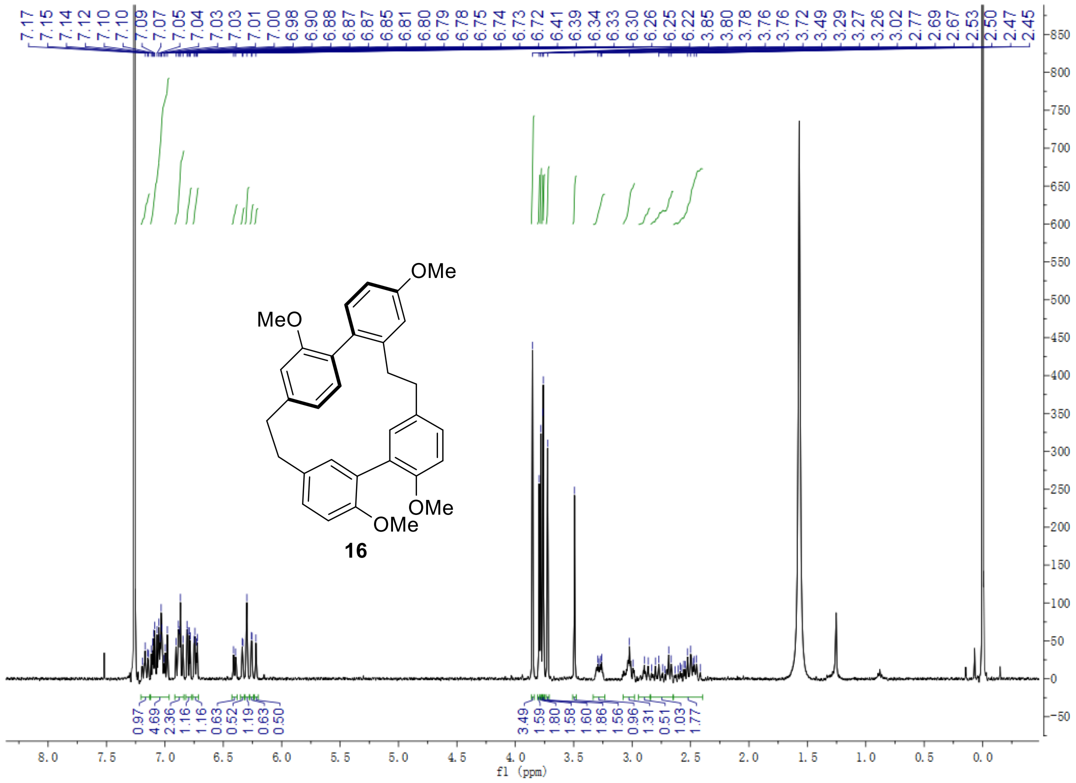

Supplementary Figure 220 ^{13}C NMR spectrum of **16** (151 MHz, $CDCl_3$)

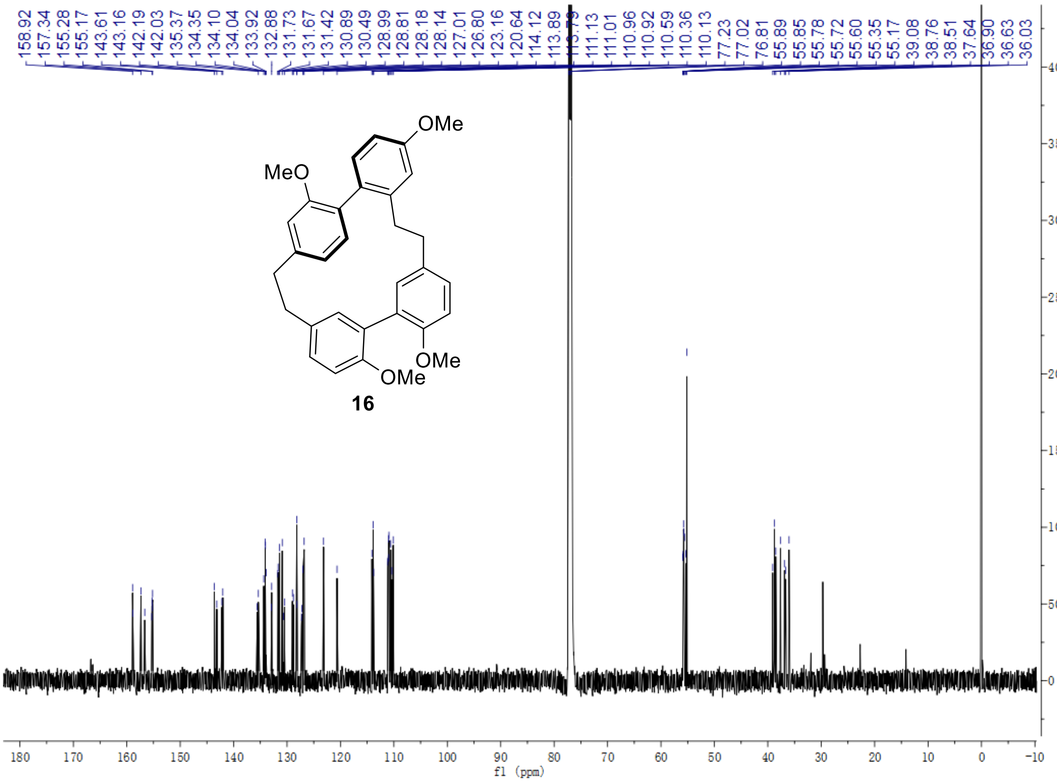

Supplementary Figure 221 ^{1}H NMR spectrum of isoplagiochin D (400 MHz, MeOD-d_4)

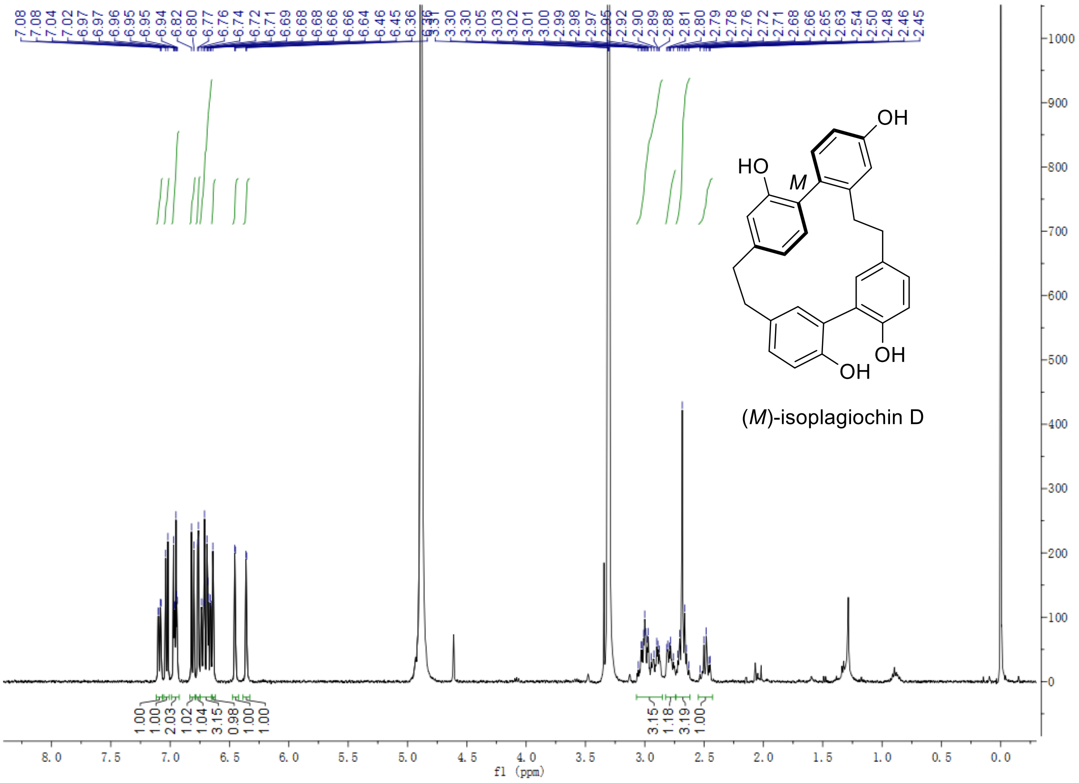

Supplementary Figure 222 ^{13}C NMR spectrum of isoplagiochin D (151 MHz, MeOD-d_4)

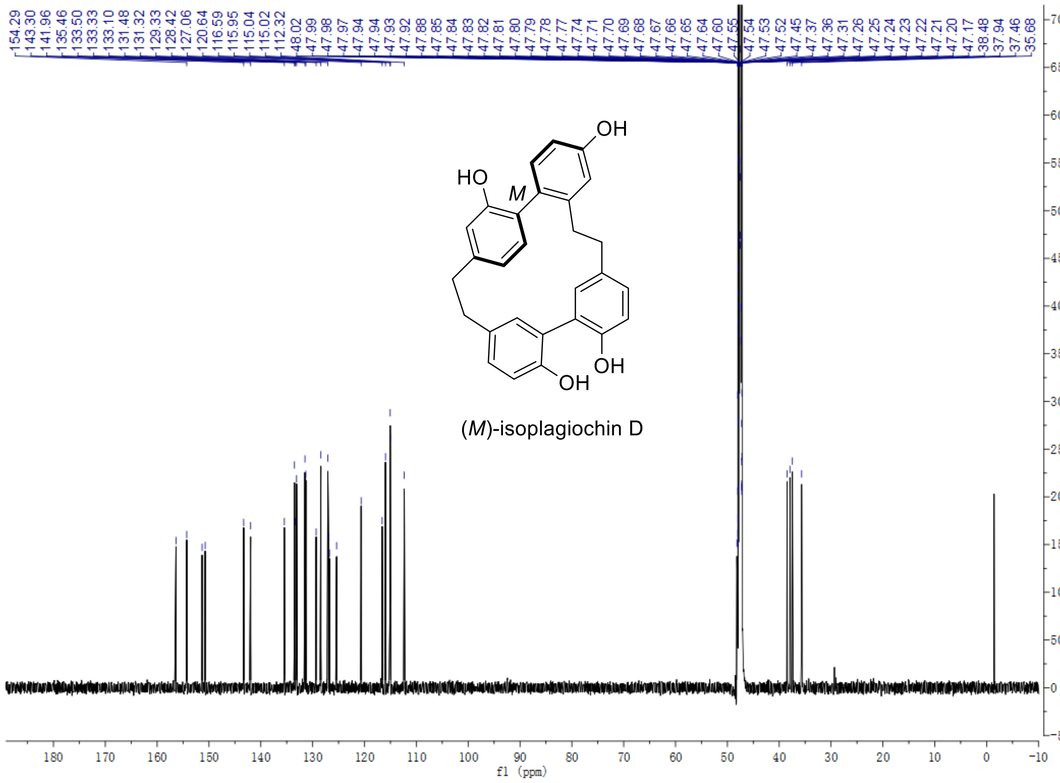

3. Supplementary References

[1] Liu, G. *et al*. Pyrrolidines and piperidines bearing chiral tertiary alcohols by nickel-catalyzed enantioselective reductive cyclization of N-alkynones. *Commun. Chem.* **1**, 90-96 (2018).

[2] Zhang, B., Zhang, X., Hao, J. & Yang, C. Direct Approach to N-Substituted-2-Fluoroindoles by Sequential Construction of C–N Bonds from gem-Difluorostyrenes. *Org. Lett.* **19**, 1780-1783 (2017).

[3] Huleatt, P. B. *et al*. Concise, efficient and practical assembly of bromo-5,6-dimethoxyindole building blocks. *Tetrahedron Lett.* **52**, 1339-1342 (2011).

[4] Yang, H., Sun, J., Gu, W. & Tang, W. Enantioselective Cross-Coupling for Axially Chiral Tetra-ortho-Substituted Biaryls and Asymmetric Synthesis of Gossypol. *J. Am. Chem. Soc.* **142**, 8036-8043 (2020).

[5] Yamaoka, Y., Taniguchi, M., Yamada, K. & Takasu, K. Total Synthesis of Phenanthroquinolizidine Alkaloid Cryptopleurine and Phenanthroindolizidine Alkaloid Tylophorine. *Heterocycles* **97**, 292-305 (2018).

[6] Pschirer, N. G., Kohl, C., Nolde, F., Qu, J. & Mullen, K. Pentarylene- and Hexarylenebis(dicarboximide)s: Near-Infrared-Absorbing Polyaromatic Dyes. *Angew. Chem. Int. Ed.* **45**, 1401-1404 (2006).

[7] Grenier, J. L., Cotelle, N., Catteau, J. P. & Cotelle, P. J. Synthesis and physico-chemical properties of nitrocaffeic acids. *J. Phys. Org. Chem.* **13**, 511-517 (2000).

[8] Kulkarni, N. N., Kulkarni, V. S., Lele, S. R. & Hosangadi, B. D. Synthetic studies on medium and large ring esters. *Tetrahedron* **44**, 5145-5150 (1988).

[9] Bartolucci, S., Bartoccini, F., Righi, M. & Piersanti, G. Direct, Regioselective, and Chemoselective Preparation of Novel Boronated Tryptophans by Friedel-Craft Alkylation. *Org. Lett.* **14**, 600-603 (2012).

[10] Sasaki, K. & Hayashi, T. Asymmetric conjugate addition of cis-2-arylethenylboronic acids catalyzed by chiral diene/rhodium complexes: 1,4-rhodium shift from alkenylrhodium to arylrhodium intermediates. *Tetrahedron* **23**, 373-380 (2012).

[11] Patel, D. C., Woods, R. M., Breitbach, Z. S., Berthod, A. & Armstrong, D. W. Thermal racemization of biaryl atropisomers. *Tetrahedron: Asymmetry* **28**, 1557-1561 (2017).

[12] Yao, Q.-J., Zhang, S., Zhan, B.-B. & Shi, B.-F. Atroposelective Synthesis of Axially Chiral Biaryls by Palladium-Catalyzed Asymmetric C−H Olefination Enabled by a Transient Chiral Auxiliary. *Angew. Chem. Int. Ed.* **56**, 6617-6621 (2017).

[13] Xi, J. & Gu, Z. Palladium-Catalzyed Atroposelective 16-Membered Macrocyclization: Total Synthesis of Isoplagiochin D. *Chin. J. Chem.* **38**, 1081-1085 (2020).

[14] Meidlinger, D. *et al*. Access to the Enantiopure Axially Chiral Cyclophane Isoplagiochin D through Atropo-diastereoselective Heck Coupling. *Angew. Chem. Int. Ed.* **57**, 9160-9164 (2018).
