# Supplementary material for: Enantioselective construction of ortho-sulfur- or nitrogen-substituted axially chiral biaryls and asymmetric synthesis of isoplagiochin D
Source: Nat Commun. 2022 Aug 5;13:4577. doi: 10.1038/s41467-022-32360-7 (PMC9355965; doi:10.1038/s41467-022-32360-7)

checkCIF/PLATON report

Structure factors have been supplied for datablock(s) d8v21987

THIS REPORT IS FOR GUIDANCE ONLY. IF USED AS PART OF A REVIEW PROCEDURE FOR PUBLICATION, IT SHOULD NOT REPLACE THE EXPERTISE OF AN EXPERIENCED CRYSTALLOGRAPHIC REFEREE.

No syntax errors found. CIF dictionary Interpreting this report

Datablock: d8v21987

Bond precision: C-C = 0.0050 A Wavelength=0.71073

Cell:	a=11.5498(5)	b=15.9048(6)	c=16.9151(7)
	alpha=90	beta=90	gamma=90
Temperature:	293 K		

	Calculated	Reported
Volume	3107.3(2)	3107.3(2)
Space group	P 21 21 21	P 21 21 21
Hall group	P 2ac 2ab	P 2ac 2ab
Moiety formula	C39 H24 O3 S, C H2 Cl2	?
Sum formula	C40 H26 Cl2 O3 S	C40 H26 Cl2 O3 S
Mr	657.57	657.57
Dx,g cm-3	1.406	1.406
Z	4	4
Mu (mm-1)	0.317	0.317
F000	1360.0	1360.0
F000’	1362.21	
h,k,lmax	14,19,20	14,19,20
Nref	6101[3425]	6065
Tmin,Tmax	0.945,0.963	0.630,0.746
Tmin’	0.939	

Correction method= # Reported T Limits: Tmin=0.630 Tmax=0.746
AbsCorr = MULTI-SCAN

Data completeness= 1.77/0.99 Theta(max)= 25.985

R(reflections)= 0.0422(5300) wR2(reflections)= 0.1105(6065)

S = 1.042 Npar= 444

The following ALERTS were generated. Each ALERT has the format
test-name_ALERT_alert-type_alert-level.
Click on the hyperlinks for more details of the test.

Alert level C

```
PLAT260_ALERT_2_C Large Average Ueq of Residue Including       Cl1      0.162 Check
PLAT260_ALERT_2_C Large Average Ueq of Residue Including      Cl1'      0.120 Check
PLAT340_ALERT_3_C Low Bond Precision on  C-C Bonds ...............      0.005 Ang.
PLAT911_ALERT_3_C Missing FCF Refl Between Thmin & STh/L=    0.600         21 Report
PLAT913_ALERT_3_C Missing # of Very Strong Reflections in FCF ....         16 Note
```

Alert level G

```
PLAT002_ALERT_2_G Number of Distance or Angle Restraints on AtSite          6 Note
PLAT003_ALERT_2_G Number of Uiso or Uij Restrained non-H Atoms ...          6 Report
PLAT172_ALERT_4_G The CIF-Embedded .res File Contains DFIX Records          1 Report
PLAT186_ALERT_4_G The CIF-Embedded .res File Contains ISOR Records          1 Report
PLAT199_ALERT_1_G Reported _cell_measurement_temperature ..... (K)        293 Check
PLAT200_ALERT_1_G Reported   _diffrn_ambient_temperature ..... (K)        293 Check
PLAT300_ALERT_4_G Atom Site Occupancy of Cl1        Constrained at        0.7 Check
PLAT300_ALERT_4_G Atom Site Occupancy of Cl2        Constrained at        0.7 Check
PLAT300_ALERT_4_G Atom Site Occupancy of C40        Constrained at        0.7 Check
PLAT300_ALERT_4_G Atom Site Occupancy of H40A       Constrained at        0.7 Check
PLAT300_ALERT_4_G Atom Site Occupancy of H40B       Constrained at        0.7 Check
PLAT300_ALERT_4_G Atom Site Occupancy of Cl1'       Constrained at        0.3 Check
PLAT300_ALERT_4_G Atom Site Occupancy of Cl2'       Constrained at        0.3 Check
PLAT300_ALERT_4_G Atom Site Occupancy of C40'       Constrained at        0.3 Check
PLAT300_ALERT_4_G Atom Site Occupancy of H40C       Constrained at        0.3 Check
PLAT300_ALERT_4_G Atom Site Occupancy of H40D       Constrained at        0.3 Check
PLAT302_ALERT_4_G Anion/Solvent/Minor-Residue Disorder (Resd  2  )       100% Note
PLAT302_ALERT_4_G Anion/Solvent/Minor-Residue Disorder (Resd  3  )       100% Note
PLAT304_ALERT_4_G Non-Integer Number of Atoms in ..... (Resd  2  )       3.50 Check
PLAT304_ALERT_4_G Non-Integer Number of Atoms in ..... (Resd  3  )       1.50 Check
PLAT335_ALERT_2_G Check Large C6 Ring C-C Range C31      -C36            0.22 Ang.
PLAT432_ALERT_2_G Short Inter X...Y Contact  Cl2'     ..C4       .       3.22 Ang.
                                                        x,y,z  =      1_555 Check
PLAT860_ALERT_3_G Number of Least-Squares Restraints ............          40 Note
PLAT883_ALERT_1_G No Info/Value for _atom_sites_solution_primary .     Please Do !
PLAT910_ALERT_3_G Missing # of FCF Reflection(s) Below Theta(Min).          1 Note
PLAT933_ALERT_2_G Number of HKL-OMIT Records in Embedded .res File          4 Note
PLAT965_ALERT_2_G The SHELXL WEIGHT Optimisation has not Converged     Please Check
PLAT967_ALERT_5_G Note: Two-Theta Cutoff Value in Embedded .res ..       52.0 Degree
PLAT978_ALERT_2_G Number C-C Bonds with Positive Residual Density.          5 Info
```

```
   0 ALERT level A = Most likely a serious problem - resolve or explain
   0 ALERT level B = A potentially serious problem, consider carefully
   5 ALERT level C = Check. Ensure it is not caused by an omission or oversight
  29 ALERT level G = General information/check it is not something unexpected

   3 ALERT type 1 CIF construction/syntax error, inconsistent or missing data
   9 ALERT type 2 Indicator that the structure model may be wrong or deficient
   5 ALERT type 3 Indicator that the structure quality may be low
  16 ALERT type 4 Improvement, methodology, query or suggestion
   1 ALERT type 5 Informative message, check
```

Validation response form

Please find below a validation response form (VRF) that can be filled in and pasted into your CIF.

```
# start Validation Reply Form
_vrf_PLAT260_d8v21987
;
PROBLEM: Large Average Ueq of Residue Including       Cl1      0.162 Check
RESPONSE: ...
;
_vrf_PLAT340_d8v21987
;
PROBLEM: Low Bond Precision on  C-C Bonds ...............      0.005 Ang.
RESPONSE: ...
;
_vrf_PLAT911_d8v21987
;
PROBLEM: Missing FCF Refl Between Thmin & STh/L=    0.600         21 Report
RESPONSE: ...
;
_vrf_PLAT913_d8v21987
;
PROBLEM: Missing # of Very Strong Reflections in FCF ....         16 Note
RESPONSE: ...
;
# end Validation Reply Form
```

It is advisable to attempt to resolve as many as possible of the alerts in all categories. Often the minor alerts point to easily fixed oversights, errors and omissions in your CIF or refinement strategy, so attention to these fine details can be worthwhile. In order to resolve some of the more serious problems it may be necessary to carry out additional measurements or structure refinements. However, the purpose of your study may justify the reported deviations and the more serious of these should normally be commented upon in the discussion or experimental section of a paper or in the "special_details" fields of the CIF. checkCIF was carefully designed to identify outliers and unusual parameters, but every test has its limitations and alerts that are not important in a particular case may appear. Conversely, the absence of alerts does not guarantee there are no aspects of the results needing attention. It is up to the individual to critically assess their own results and, if necessary, seek expert advice.

Publication of your CIF in IUCr journals

A basic structural check has been run on your CIF. These basic checks will be run on all CIFs submitted for publication in IUCr journals (*Acta Crystallographica*, *Journal of Applied Crystallography*, *Journal of Synchrotron Radiation*); however, if you intend to submit to *Acta Crystallographica Section C* or *E* or *IUCrData*, you should make sure that full publication checks are run on the final version of your CIF prior to submission.

Publication of your CIF in other journals

Please refer to the *Notes for Authors* of the relevant journal for any special instructions relating to CIF submission.

PLATON version of 18/05/2022; check.def file version of 17/05/2022

Datablock d8v21987 - ellipsoid plot

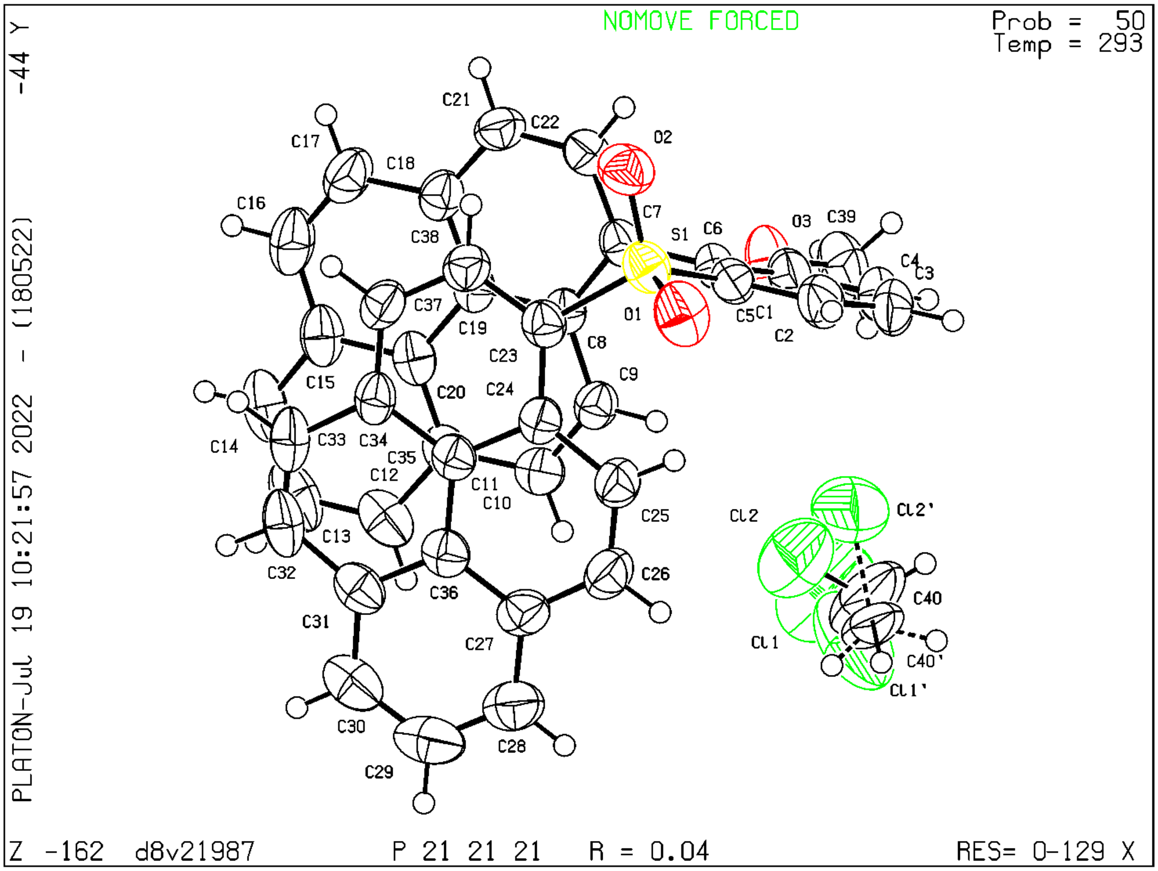

Supplement: Supplementary file 3 — Source Data [file 41467_2022_32360_MOESM3_ESM.zip › Inventory of Supporting Information/cif files/checkcif compound 17.pdf]
