# Supplementary material for: Enantioselective construction of ortho-sulfur- or nitrogen-substituted axially chiral biaryls and asymmetric synthesis of isoplagiochin D
Source: Nat Commun. 2022 Aug 5;13:4577. doi: 10.1038/s41467-022-32360-7 (PMC9355965; doi:10.1038/s41467-022-32360-7)

checkCIF/PLATON report

Structure factors have been supplied for datablock(s) t_a

THIS REPORT IS FOR GUIDANCE ONLY. IF USED AS PART OF A REVIEW PROCEDURE FOR PUBLICATION, IT SHOULD NOT REPLACE THE EXPERTISE OF AN EXPERIENCED CRYSTALLOGRAPHIC REFEREE.

No syntax errors found. CIF dictionary Interpreting this report

Datablock: t_a

```
Bond precision:  C-C = 0.0065 A          Wavelength=1.54178

Cell:            a=23.0578(5)     b=23.0578(5)     c=10.1350(2)
                 alpha=90         beta=90          gamma=120
Temperature:     297 K

                Calculated                  Reported
Volume          4666.5(3)                   4666.5(2)
Space group     R 3                         R 3 :h
Hall group      R 3                         R 3
Moiety formula  C24 H19 N O4, O             C24 H19 N O4, O
Sum formula     C24 H19 N O5                C24 H19 N O5
Mr              401.40                      401.40
Dx,g cm-3       1.286                       1.286
Z               9                           9
Mu (mm-1)       0.745                       0.745
F000            1890.0                      1890.0
F000'           1896.15
h,k,lmax        27,27,12                    27,27,12
Nref            3798[ 1899]                 3772
Tmin,Tmax       0.862,0.894
Tmin'           0.862

Correction method= Not given

Data completeness= 1.99/0.99      Theta(max)= 68.269

R(reflections)= 0.0571( 3402)     wR2(reflections)= 0.1585( 3772)

S = 1.064                   Npar= 274
```

The following ALERTS were generated. Each ALERT has the format
test-name_ALERT_alert-type_alert-level.
Click on the hyperlinks for more details of the test.

Alert level B

```
PLAT306_ALERT_2_B Isolated Oxygen Atom (H-atoms Missing ?) .......         O5 Check
PLAT430_ALERT_2_B Short Inter D...A Contact  O5       ..O5       .       2.72 Ang.
                                                   -y,x-y,z  =      2_555 Check
PLAT430_ALERT_2_B Short Inter D...A Contact  O5       ..O5       .       2.72 Ang.
                                                  -x+y,-x,z  =      3_555 Check
```

Alert level C

```
DIFMX02_ALERT_1_C  The maximum difference density is > 0.1*ZMAX*0.75
            The relevant atom site should be identified.
STRVA01_ALERT_4_C           Flack parameter is too small
           From the CIF: _refine_ls_abs_structure_Flack   -0.340
           From the CIF: _refine_ls_abs_structure_Flack_su    0.120
PLAT052_ALERT_1_C Info on Absorption Correction Method   Not Given     Please Do !
PLAT089_ALERT_3_C Poor Data / Parameter Ratio (Zmax < 18) ........       6.92 Note
PLAT094_ALERT_2_C Ratio of Maximum / Minimum Residual Density ....       3.94 Report
PLAT097_ALERT_2_C Large Reported Max.  (Positive) Residual Density       0.80 eA-3
PLAT242_ALERT_2_C Low    'MainMol' Ueq as Compared to Neighbors of         N1 Check
PLAT260_ALERT_2_C Large Average Ueq of Residue Including       O5      0.183 Check
PLAT340_ALERT_3_C Low Bond Precision on  C-C Bonds ...............    0.00652 Ang.
PLAT911_ALERT_3_C Missing FCF Refl Between Thmin & STh/L=    0.600          3 Report
PLAT918_ALERT_3_C Reflection(s) with I(obs) much Smaller I(calc) .          1 Check
```

Alert level G

```
PLAT072_ALERT_2_G SHELXL First  Parameter in WGHT  Unusually Large       0.12 Report
PLAT790_ALERT_4_G Centre of Gravity not Within Unit Cell: Resd.  #          2 Note
              O
PLAT883_ALERT_1_G No Info/Value for _atom_sites_solution_primary .     Please Do !
PLAT916_ALERT_2_G Hooft y and Flack x Parameter Values Differ by .       0.11 Check
PLAT978_ALERT_2_G Number C-C Bonds with Positive Residual Density.          2 Info
```

---

0 **ALERT level A** = Most likely a serious problem - resolve or explain
3 **ALERT level B** = A potentially serious problem, consider carefully
11 **ALERT level C** = Check. Ensure it is not caused by an omission or oversight
5 **ALERT level G** = General information/check it is not something unexpected

3 ALERT type 1 CIF construction/syntax error, inconsistent or missing data
10 ALERT type 2 Indicator that the structure model may be wrong or deficient
4 ALERT type 3 Indicator that the structure quality may be low
2 ALERT type 4 Improvement, methodology, query or suggestion
0 ALERT type 5 Informative message, check

It is advisable to attempt to resolve as many as possible of the alerts in all categories. Often the minor alerts point to easily fixed oversights, errors and omissions in your CIF or refinement strategy, so attention to these fine details can be worthwhile. In order to resolve some of the more serious problems it may be necessary to carry out additional measurements or structure refinements. However, the purpose of your study may justify the reported deviations and the more serious of these should normally be commented upon in the discussion or experimental section of a paper or in the "special_details" fields of the CIF. checkCIF was carefully designed to identify outliers and unusual parameters, but every test has its limitations and alerts that are not important in a particular case may appear. Conversely, the absence of alerts does not guarantee there are no aspects of the results needing attention. It is up to the individual to critically assess their own results and, if necessary, seek expert advice.

Publication of your CIF in IUCr journals

A basic structural check has been run on your CIF. These basic checks will be run on all CIFs submitted for publication in IUCr journals (*Acta Crystallographica*, *Journal of Applied Crystallography*, *Journal of Synchrotron Radiation*); however, if you intend to submit to *Acta Crystallographica Section C* or *E* or *IUCrData*, you should make sure that full publication checks are run on the final version of your CIF prior to submission.

Publication of your CIF in other journals

Please refer to the *Notes for Authors* of the relevant journal for any special instructions relating to CIF submission.

PLATON version of 03/05/2019; check.def file version of 29/04/2019

Datablock t_a - ellipsoid plot

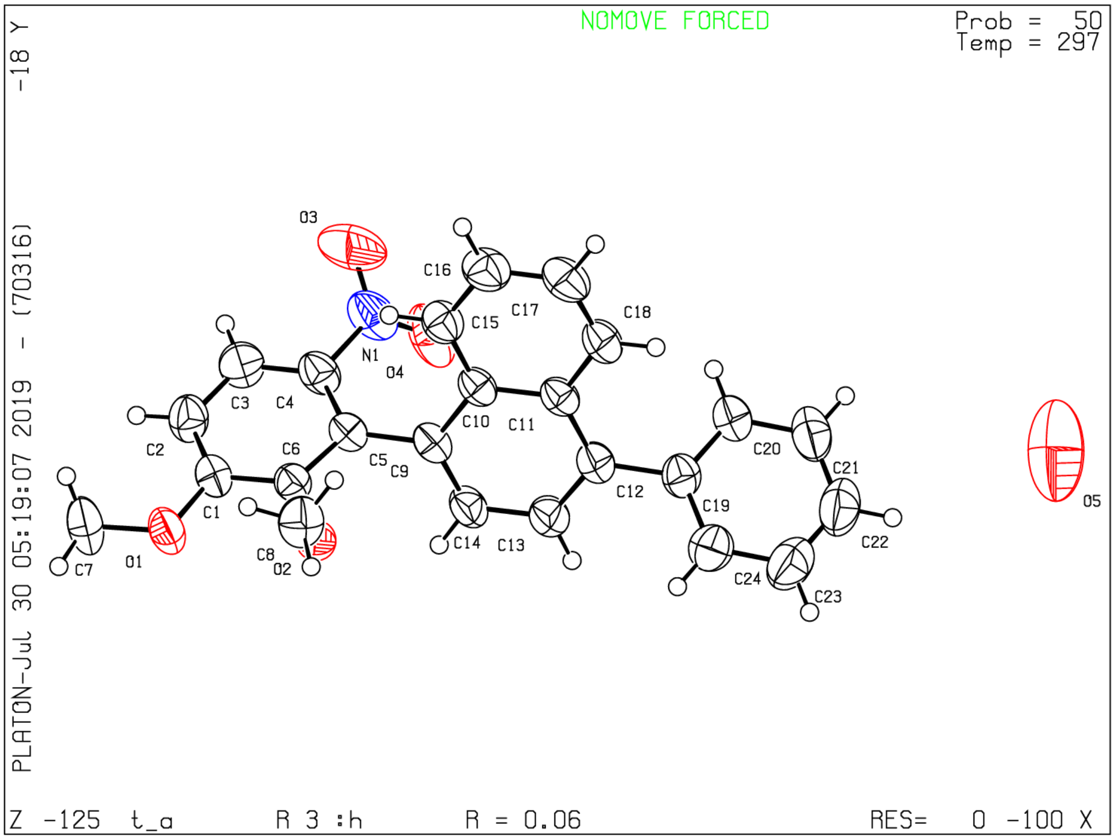

Supplement: Supplementary file 3 — Source Data [file 41467_2022_32360_MOESM3_ESM.zip › Inventory of Supporting Information/cif files/checkcif compound 6a.pdf]
